# Catalytic Enantioselective Intramolecular Oxa-Michael Reaction to α,β-Unsaturated Esters and Amides

**DOI:** 10.1021/jacs.3c03182

**Published:** 2023-05-30

**Authors:** Guanglong Su, Michele Formica, Ken Yamazaki, Trevor A. Hamlin, Darren J. Dixon

**Affiliations:** † Department of Chemistry, Chemistry Research Laboratory, University of Oxford, 12 Mansfield Road, OX1 3TA Oxford, U.K.; ‡ Department of Theoretical Chemistry, Amsterdam Institute of Molecular and Life Sciences (AIMMS), Amsterdam Center for Multiscale Modeling (ACMM), Vrije Universiteit Amsterdam, De Boelelaan 1083, 1081 HV Amsterdam, The Netherlands

## Abstract

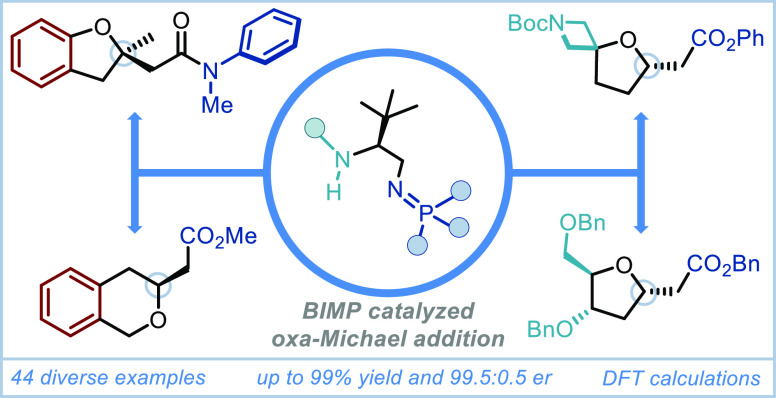

A bifunctional iminophosphorane (BIMP)-catalyzed, enantioselective intramolecular oxa-Michael reaction of alcohols to tethered, low electrophilicity Michael acceptors is described. Improved reactivity over previous reports (1 day vs 7 days), excellent yields (up to 99%), and enantiomeric ratios (up to 99.5:0.5 er) are demonstrated. The broad reaction scope, enabled by catalyst modularity and tunability, includes substituted tetrahydrofurans (THFs) and tetrahydropyrans (THPs), oxaspirocycles, sugar and natural product derivatives, dihydro-(iso)-benzofurans, and iso-chromans. A state-of-the-art computational study revealed that the enantioselectivity originates from the presence of several favorable intermolecular hydrogen bonds between the BIMP catalyst and the substrate that induce stabilizing electrostatic and orbital interactions. The newly developed catalytic enantioselective approach was carried out on multigram scale, and multiple Michael adducts were further derivatized to an array of useful building blocks, providing access to enantioenriched biologically active molecules and natural products.

## Introduction

**Scheme 1:**
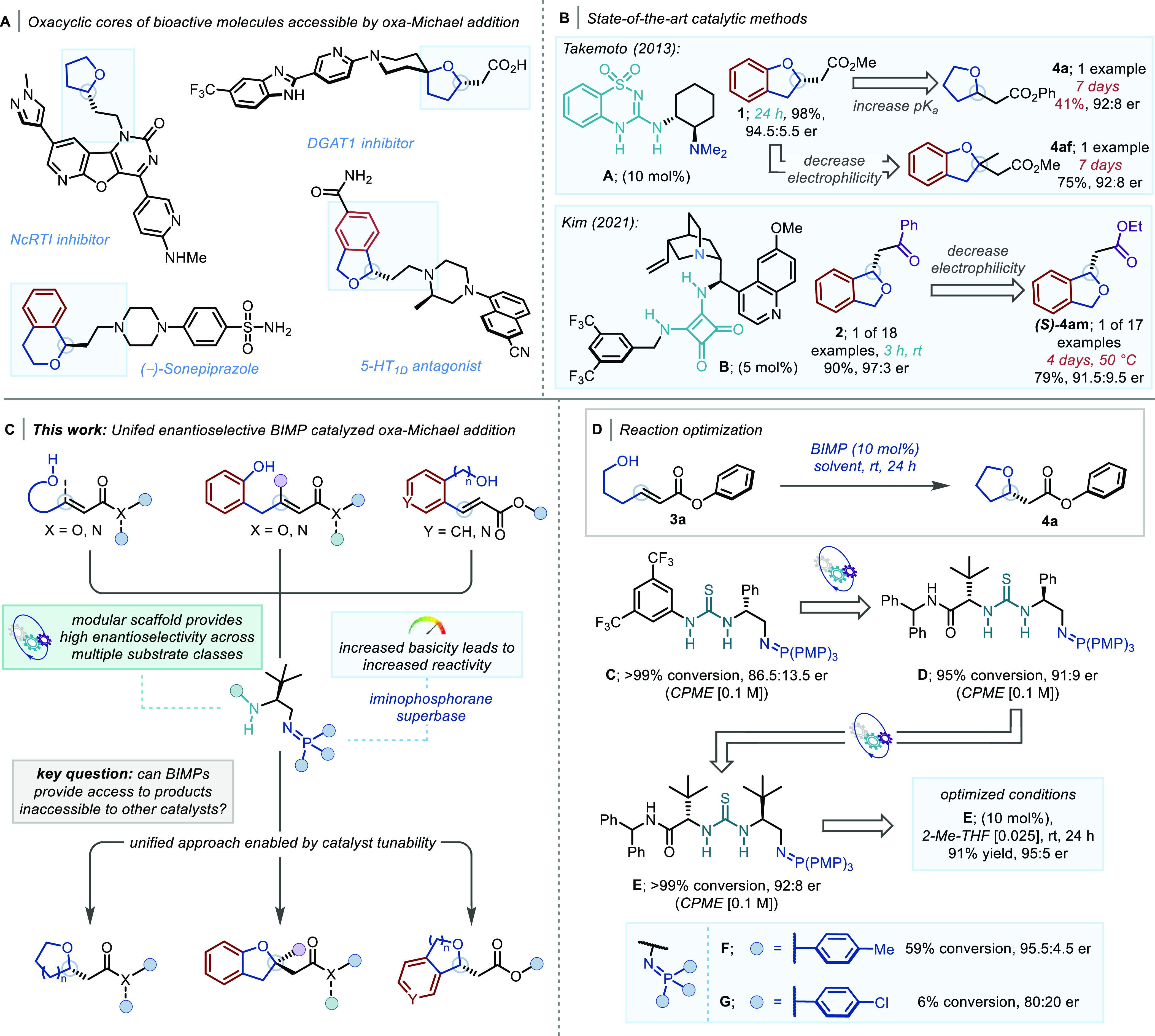
[A] Oxacyclic Cores of Bioactive Molecules (Highlighted in Blue) Accessible by Oxa-Michael Addition; [B] Limitations of Current State-of-the-Art Methods; [C] BIMP-Enabled Unified Approach to Enantioenriched Cyclic Ethers; and [D] Reaction Optimization

Saturated, α-substituted, chiral oxygen-containing heterocycles^1−5^ are among the most common structural motifs found in natural products^2d,4e,4f^ and pharmaceuticals, examples of which include the dopamine D4 antagonist (−)-sonepiprazole^5d^ (Scheme 1A). While multiple approaches to this important class of compounds exist, these are highly fragmented, with minor structural variations in the substrate often requiring completely different modes of catalysis, offering highly variable levels of performance and selectivity. The development and application of new catalytic systems enabling the enantioselective construction of these cyclic ether frameworks across a wide range of substrate classes are therefore of great importance.

Among the most direct strategies^6^ to access chiral cyclic ethers are enantioselective intramolecular oxa-Michael reactions. These transformations are typically very challenging due to the lower nucleophilicity^7^ or the higher p*K*_a_^8^ of alcohol nucleophiles when compared to the corresponding *C*-, *N*-, and *S* counterparts. Additionally, the chiral cyclic ethers obtained from these reactions are well-known to undergo retro-Michael in the presence of base leading to potential racemization.^9,10^ While additions of pronucleophilic alcohols to tethered, activated electrophiles such as β-substituted α,β-unsaturated aldehydes,^11a^ ketones,^11b,11c−11l,11m,11n^ thioesters,^11o,11p^ and imides/*N*-acyl pyrroles^11q^ are well developed, corresponding reactions to much less electrophilic^12^ (yet more attractive) α,β-unsaturated esters or amides remain largely unknown.^13^ Such reactions would provide direct access to the desired enantioenriched cyclic ethers without the need for extensive functional group manipulation to install and remove activating groups, which are prevalent in the literature due to the typical low activity of the catalysts being employed.

Current state-of-the-art approaches for enantioselective intramolecular oxa-Michael reactions to α,β-unsaturated esters or amides have relied heavily on the use of more acidic phenols as tethered nucleophiles as they can be partially deprotonated even by weaker 3° amine bases.^13^ A clear example of this can be seen in the work of Takemoto,^13e^ where novel bifunctional 3° amine catalyst **A**, bearing a highly acidic H-bond donor, could provide rapid access to a wide range of enantioenriched benzofurans (such as **1**) but was recalcitrant in affording THF product **4a** even after 7 days and in only 41% yield. Additionally, in the case of β-substituted benzofuran **4af**, 7 days were also required due to the lower electrophilicity of the disubstituted acceptor. In an analogous scenario,^13f^ squaramide-containing, bifunctional cinchona catalyst **B** was demonstrated by Kim to smoothly promote the enantioselective intramolecular oxa-Michael reaction of benzylic alcohols to afford enantioenriched 1-substituted phthalans (such as **2**) using α,β-unsaturated ketones as Michael acceptors. When the tethered α,β-unsaturated ketone acceptor was replaced with an α,β-unsaturated ester (**(*S*)**-**4am**) however, extended reaction times of 4 days at 50 °C were typical, once again highlighting the limitations of 3° amine base bifunctional catalysts in promoting challenging oxa-Michael reactions (Scheme 1B).^13^ Knowing that the bifunctional iminophosphorane (BIMP) superbase catalysts developed in our group often vastly outperform 3° amine catalysts in multiple scenarios, thanks to the increased basicity of the iminophosphorane group,^14^ it was very likely this catalyst class could perform these challenging enantioselective reactions in only a fraction of the time.

While enabling faster reactions was already a worthwhile goal, the true challenge lay in enabling intramolecular oxa-Michael reactions far beyond the reach of current methods. We envisaged that the enhanced Brønsted basicity of the BIMP system could potentially provide synergistic activation for much less reactive and more hindered nucleophiles such as secondary and tertiary alcohols, in concert with tethers of varying length and low electrophilicity, β-substituted, α,β-unsaturated amide and ester acceptors. Moreover, thanks to the tuneability and modularity of the BIMP catalyst system, we believed that it could provide the ideal platform to perform enantioselective oxa-Michael reactions across a wide range of substrate classes, providing a truly unified and broad scope approach to the synthesis of enantioenriched cyclic ethers and herein we sought to present our findings (Scheme 1C).

## Results and Discussion

In our initial exploration, we were soon pleased to find that first-generation BIMP catalyst **C** could smoothly promote the cyclization of demanding precursor **3a** to enantioenriched THF **4a** with full conversion and good enantioselectivity (86.5:13.5 er) in only 24 h. The enantioselectivity was subsequently improved to 91:9 er by using second-generation BIMP catalyst **D**, which bears an additional stereocenter flanking the thiourea. Exchanging the phenyl substituent on the stereocenter proximal to the iminophosphorane to a *tert*-butyl group further enhanced enantioselectivity to 92:8 and, following a systematic screen of solvents, reaction temperature, and concentration (see the Supporting Information for full optimization), we were delighted to find that when the reaction was carried out in 2-MeTHF (0.025 M), **4a** could be obtained in 91% isolated yield and 95:5 er. Significantly, when using derivatives of catalyst **E** carrying a less Brønsted basic iminophosphorane motif (**F** and **G**), the oxa-Michael reaction was found to be much less efficient, highlighting the importance and advantages of employing a superbase catalyst (Scheme 1D).

**Scheme 2:**
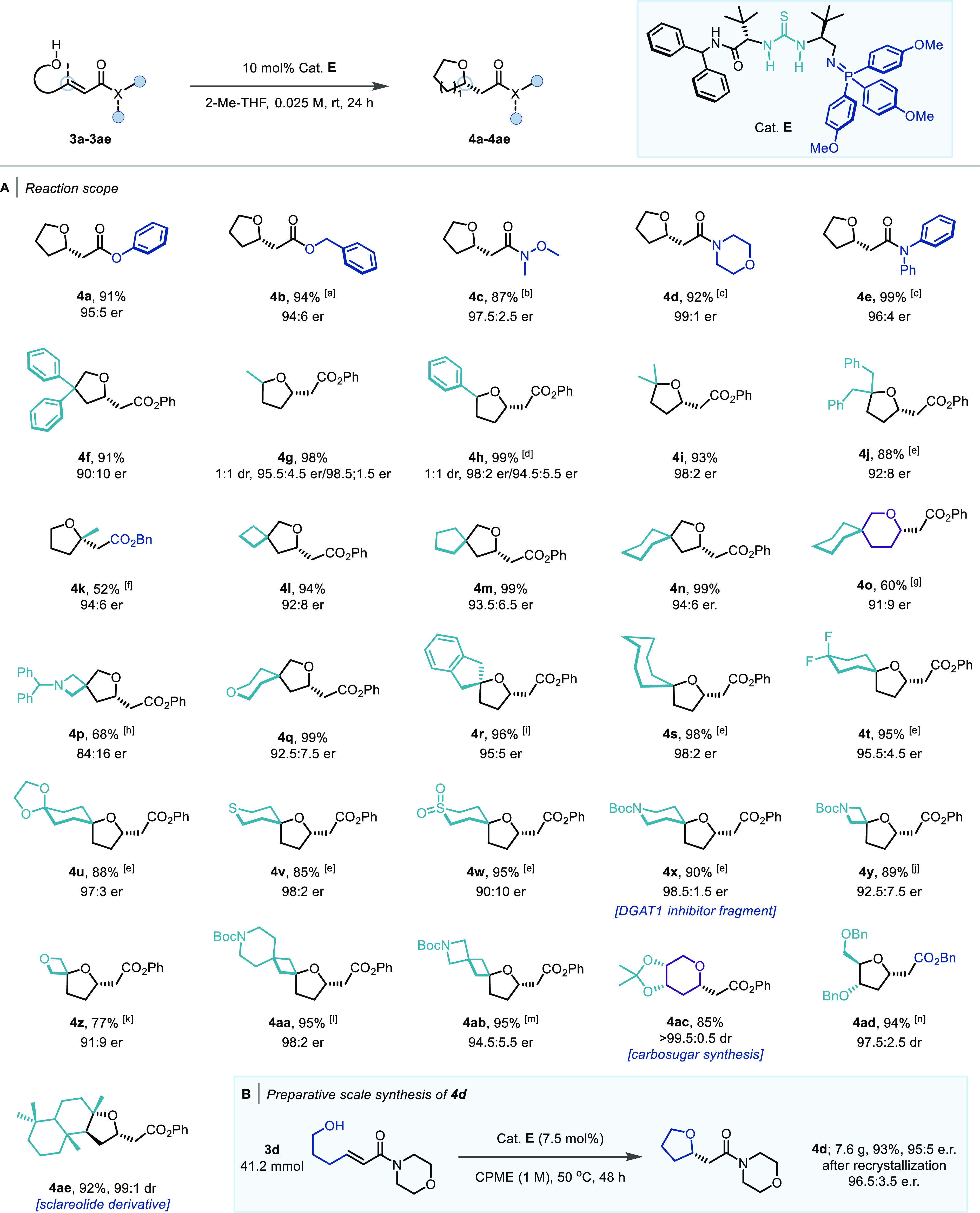
[A] Scope of the BIMP-Catalyzed Intramolecular Oxa-Michael Reaction to α,β-Unsaturated Esters and Amides; Reactions Were Carried Out on 0.2 mmol Scale; All Yields Are Isolated Yields; er Determined by HPLC Analysis on a Chiral Stationary Phase; dr Determined by ^1^H NMR Analysis; Variations from Standard Conditions: [a] 0.2 M; [b] CPME (0.1 M), 48 h; [c] 15 mol % cat. **E** in CPME (0.1 M), 48 h; [d] 8 h; [e] 0.2 M, 48 h; [f] 15 mol % cat. **E**, 0.2 M, 50 °C, 72 h; [g] 8 mol % cat. **E** for 72 h; [h] 48 h; [i] 0.1 M, 48 h; [j] −22 °C; [k] 0.2 M, −22 °C, 10 h; [l] 0.05 M, 48 h; [m] 0.05 M; [n] 0.1 M, 10 mol % Enantiomer of cat. **E**; [B] Preparative Scale Synthesis of **4d**. Stereochemical Configuration of **4a**–**4j** and **4l**–**4ae** Was Assigned by Analogy with **(*S*)-4b** (Determined by Comparison of Its Specific Rotation with That Reported in ref (6j)); Stereochemical Configuration of **4k** Was Determined by Comparison of Its Specific Rotation with that Reported in ref (6c), See the Supporting Information for More Details

With the optimized conditions in hand, the scope of the reaction was explored (Scheme 2A). Variations in the nature of the Michael acceptor were well tolerated, providing access to varied ester and amide derivatives (**4a–4e**) in high yield and enantioselectivity. In the case of **3d** and **3e**, 15 mol % catalyst was required to obtain >90% yield of **4d** and **4e**.

Having investigated modifications to the Michael acceptor, the effect of substituents on the alcohol tether was examined. Pleasingly, β-disubstituted alcohol **3f** underwent smooth cyclization under the optimized conditions, affording **4f** in 91% yield and 90:10 er. Similarly, even more hindered α-methyl (**3g**) and α-phenyl secondary (**3h**) alcohol substrates afforded the corresponding enantioenriched cyclic ethers **4g** and **4h** in quantitative yields and high enantioselectivity as a 1:1 mixture of diastereomers, respectively. Unfortunately, as there was little difference in the reaction rates between the two enantiomeric starting materials with the selected catalyst, an effective kinetic resolution for this class of substrates could not be developed at this time. Next, α-disubstituted tertiary alcohols (**3i**, **3j**) were investigated as substrates and, to our delight, afforded the corresponding *gem*-dimethyl (**4i**) and dibenzyl (**4j**) products in excellent yield and 98:2 and 92:8 er, respectively, although an increase in reaction concentration and 48 h reaction time was required for **3j**. Finally, the effect of β-disubstitution on the Michael acceptor was investigated. While low conversion of **3k** was observed under the optimized conditions, **4k** could be obtained in 52% yield and 94:6 er when the reaction was carried out at higher concentration with 15 mol % cat. **E** at 50 °C for 72 h.

With general reactivity trends established, we envisioned that the newly developed methodology could be applied to the synthesis of medicinally relevant, yet underexplored, enantioenriched oxa-spirocycles.^15^ This family of spirocyclic compounds has been demonstrated to be much more soluble than their all-carbon counterparts and incorporates a further H-bond acceptor to modulate pharmacological properties.^15c^ To date, very few broad scope catalytic enantioselective methods toward these compounds have been developed.^15m−15o^ β-Spirocyclobutane (**4l**), pentane (**4m**), and hexane (**4n**) products were all obtained in excellent yields and enantioselectivities while spirocyclic enantioenriched THP **4o** was obtained in 60% yield and 91:9 er after 72 h. β-Spiroaziridine product **4p** was obtained in good yield and 84:16 er after 48 h, while β-spiro THP **4q** was obtained in quantitative yield and 92.5:7.5 er. Next, substrates bearing spirocyclic tertiary alcohols (**3r–3u**) were assessed in the enantioselective oxa-Michael addition. Products **4r–4u** bearing carbocyclic spirocycles were all obtained in excellent yield and enantioselectivity with both *gem*-difluoro (**4t**) and acetal (**4u**) functionalities being tolerated. Products bearing spiro-heterocycles (**4v**–**4z**) were also obtained in good to excellent yields with >90:10 er with thioethers (**4v**), sulfones (**4w**), *N*-Boc piperidine (**4x**—a DGAT1 inhibitor fragment), and azetidine (**4y**) moieties all being tolerated with minor variations in reaction conditions. Of note, for substrate **3z** bearing an α-oxetane ring, we observed significant retro-Michael reaction and subsequent racemization under the optimized conditions. This challenge could be solved by carefully controlling the reaction temperature; a 77% yield of **4z** in 91:9 er could be obtained by modifying the reaction conditions to 0.2 M 2-MeTHF at −22 °C after 10 h. This group of substrates was also extended to alcohols bearing bis-spirocyclic moieties (**3aa** and **3ab**), affording both *N*-Boc 7-azaspiro[3.5]nonane **4aa** and 2-azaspiro[3.3]heptane products **4ab** in 95% yield and 98:2 and 94.5:5.5 er, respectively.

Following the investigation of the scope of oxa-spirocycles, highly diastereoselective reactions were carried out on substrates derived from sugars (**3ac** and **3ad**) and the natural product sclareolide (**3ae**), employing the same BIMP catalyst. Gratifyingly, both pyranose (**4ac**) and furanose (**4ad**) carbosugar products^16^ were obtained in excellent yield and >95:5 dr. Importantly, when the oxa-Michael reactions were carried out using BEMP (2-*tert*-Butylimino-2-diethylamino-1,3-dimethylperhydro-1,3,2-diazaphosphorine, an achiral superbase) or 1,8-diazabicyclo(5.4.0)undec-7-ene (DBU), a <56:44 ratio of anomers was observed. Finally, sclareolide derivative **4ae** was obtained in 92% yield and 99:1 dr.

To demonstrate the scalability of the newly developed protocol, a multigram quantity of substrate **3d** (8.20 g, 41.2 mmol) was converted into the corresponding enantioenriched product **4d** in 93% yield and 95:5 er using 7.5 mol % catalyst in cyclopentyl methyl ether (CPME) at 50 °C for 48 h. The er of the product was increased to 96.5:3.5 er following recrystallization from hot hexane (Scheme 2B).

**Scheme 3:**
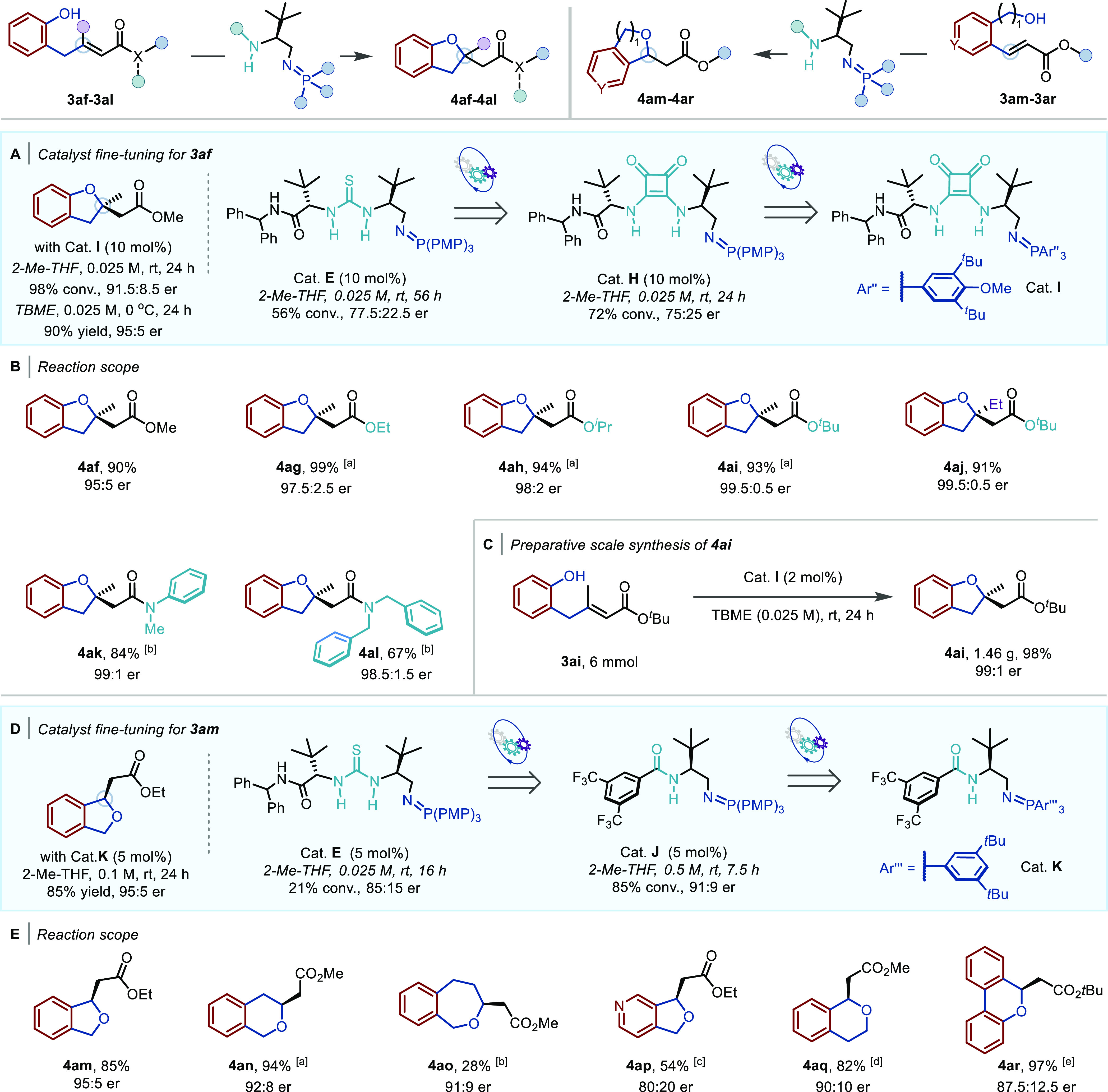
[A] Optimization of Reaction Conditions for **3af**; [B] Scope of the BIMP-Catalyzed Intramolecular Oxa-Michael Reaction to α,β-Unsaturated Ester and Amide; Reactions Were Carried on 0.2 mmol Scale; All Yields Are Isolated Yields; er Determined by HPLC Analysis on a Chiral Stationary Phase; Variations from Standard Conditions: [a] 17 h Reaction Time; [b] 40 °C, 96 h; [C] Preparative Scale Synthesis of **4ai**; the Stereochemical Configurations of **4af**–**4al** Were Assigned by Analogy with **(*R*)-5i** (Determined by Comparison of Its Specific Rotation with That Reported in ref (18)); [D] Optimization of Reaction Conditions for **3am**; [E] Scope of the BIMP-Catalyzed Intramolecular Oxa-Michael Reaction to α,β-Unsaturated Esters; Reactions Were Carried Out on 0.2 mmol Scale; All Yields Are Isolated Yields; er Determined by HPLC Analysis on a Chiral Stationary Phase; Variations from Standard Conditions: [a] (0.5 M), 10 mol % cat. **K**, 72 h; [b] (0.5 M), 10 mol % cat. **K**, 60 °C, 72 h; [c] 50 °C, 72 h; [d] (0.5 M), 10 mol % cat. **K**, 50 °C, 72 h; [**e**] (0.025 M), 10 mol % cat. **K**, −22 °C; Stereochemical Configuration of **4an**–**4ap**, **4ar** Were Assigned by Analogy with **(*R*)-4am** (Determined by Comparison of Its Specific Rotation with That Reported in ref (11f)); Stereochemical Configuration of **4aq** Was Determined by Comparison of Its Specific Rotation with That Reported in ref (6j)

Having established a broad scope for aliphatic primary, secondary, and tertiary alcohols, we turned our attention to substrates bearing a phenol nucleophile tethered to β-disubstituted, α,β-unsaturated ester or amide (**3af–3al**) (Scheme 3A). When previously optimal catalyst **E** was used on a model substrate **3af** however, only a modest yield of **4af** was obtained in a moderate 77.5:22.5 er. The issue of reactivity was easily overcome by employing catalyst **H**, bearing a squaramide H-bond donor motif in place of a thiourea.^14f,14g,17^ This catalyst exhibited excellent performance; the desired product was obtained in 72% conversion and 75:25 er after 24 h (vs 168 h with Takemoto’s optimal system). The enantioselectivity was further improved to 91.5:8.5 er using catalyst **I** which possesses bulkier aryl substituents on the iminophosphorane moiety. When this catalyst was paired with cooling the reaction to 0 °C and the use of *tert*-butyl methyl ether (TBME) as the solvent, **4af** was obtained in 90% yield and 95:5 er (see the Supporting Information for full optimization). Under the optimized reaction conditions, products decorated with increasingly more sterically encumbered esters (**4ag–4ai**) were obtained in excellent yield and enantioselectivity, and in only 17 h. Additionally, even less reactive substrates bearing a β-ethyl (**3aj**) or amide Michael acceptors (**3ak** and **3al**) underwent the desired 1,4-addition to afford products **4aj–4al** in good yield and excellent enantioselectivity (Scheme 3B). When using substrates bearing amide acceptors however, 40 °C and 96 h were required to obtain satisfactory amounts of the desired products. When the reaction was carried out on gram scale, as little as 2 mol % of catalyst **I** could be employed to obtain **4ai** in 98% yield and 99:1 er (Scheme 3C).

Subsequently, substrates bearing tethered benzylic alcohols as nucleophiles were investigated. Once again, catalyst **E** bearing a thiourea H-bond donor proved to be ineffective with this class of substrates, leading to only 21% conversion to product **4am** in and 85:15 er after 24 h. Once again, thanks to the modular nature of the BIMP catalyst family, a superior amide-derived catalyst (**J**) was quickly identified (see the Supporting Information for optimization). Using 5 mol % **J**, 85% conversion to product **4am** in 91:9 er was achieved in 7.5 h at rt (vs 96 h at 50 °C employing Kim’s protocol). Utilizing 3,5-di-*tert*-butyl-triphenyl phosphine to generate the iminophosphorane (catalyst **K**) paired with diluting the reaction to 0.1 M resulted in 85% yield of **4am** in an improved 95:5 er (Scheme 3D). Using catalyst **K**, 6- (**4am**) and 7-membered (**4an**) ring products were obtained in 94 and 28% yield and 92:8 and 91:9 er, respectively. In both cases however, an increase in temperature and reaction time was required. When substrate **3ap** bearing a pyridine moiety was used, product **4ap** was obtained in 54% yield and 80:20 er. Catalyst **K** was also found to be effective in promoting the cyclization of substrates having different linkages (**3aq–3ar**). Alternative isochroman isomer **4aq** was attained in 82% yield and 90:10 er using 10 mol % catalyst, while biaryl product **4ar** was obtained in 97% yield and moderate er when the reaction was cooled to −22 °C (Scheme 3E).

**Scheme 4:**
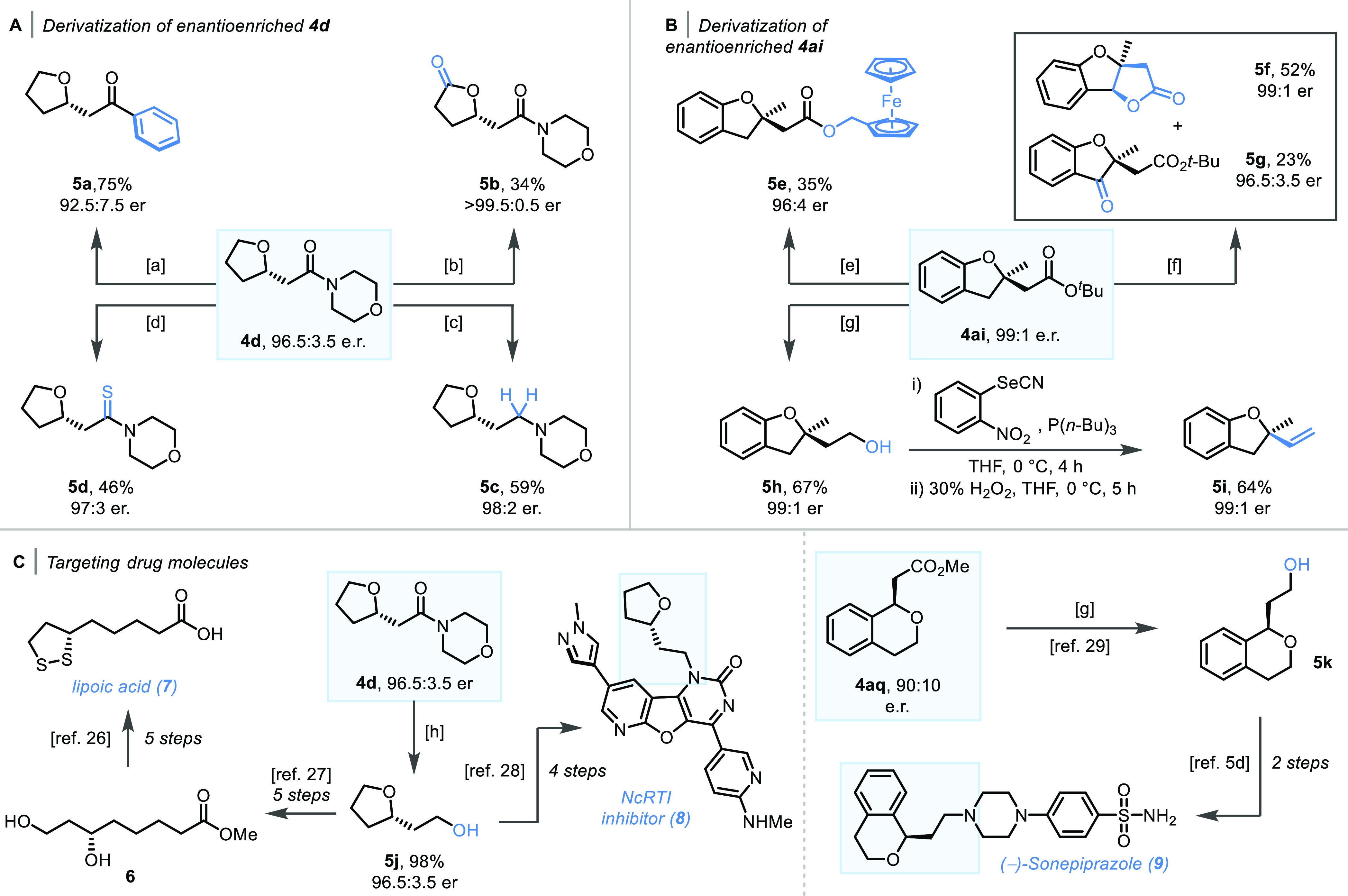
[A] Derivatization of Enantioenriched**4d**, [a] PhCeCl_2_ (2.5 equiv), THF (1 M), −78 °C, 2 h; [b] RuCl_3_·3H_2_O (2 mol %), NaIO_4_ (8.0 equiv), MeCN/H_2_O/CCl_4_ = 1/1.5/1 (0.017 M), rt, 16 h; [c] Hantzsch Ester (2.5 equiv), Tf_2_O (1.1 equiv), CH_2_Cl_2_ (0.25 M), 0 °C to rt, 16 h; [d] Lawesson’s Reagent (0.55 equiv), THF (0.43 M), 60 °C, 72 h; [B] Derivatization of Enantioenriched**4ai**: [e] (i) TFA (0.14 M), CH_2_Cl_2_ (0.14 M), 0 °C to rt, 2 h, (ii) SOCl_2_ (3.0 equiv), DMF (cat.), CH_2_Cl_2_ (0.4 M), 0 °C to rt, 2 h, (iii) Ferrocenemethanol (1.2 equiv), Et_3_N (1.2 equiv), CH_2_Cl_2_ (0.24 M), 0 °C to rt, 2 h; [f] K_2_S_2_O_8_ (3.0 equiv), CuSO_4_ (1.0 equiv), MeCN/H_2_O = 1/1 (0.014 M), 80 °C, 1 h; [g] LiAlH_4_ (1.5 equiv), THF (1.0 M), 0 °C to rt, 4 h; and [C] Targeting Drug Molecules, [h] SmI_2_ (8.0 equiv), Et_3_N (71.8 equiv), H_2_O (0.77 M), rt, 16 h

With the scope of the reaction established over three distinct substrate classes, the enantioenriched cyclic ether products were then derivatized to a wide range of attractive enantioenriched building blocks and drug precursors (Scheme 4). For example, compound **4d** was converted to the corresponding ketone using PhCeCl_2_ in 75% yield with only a slight erosion in optical purity being detected. Treating the same substrate with 2 mol % RuCl_3_·3H_2_O and sodium periodate furnished lactone product **5b** in moderate yield and >99.5:0.5 er.^18^ Alternatively, the amide moiety could be fully reduced to corresponding amine **5c** with Hantzsch ester and Tf_2_O or to thioamide **5d** using Lawesson’s reagent.^19,20^ Both products were obtained in moderate yield with no racemization being observed (Scheme 4A).

Further transformations of compound **4ai** were then explored. For example, compound **4ai** was smoothly converted to ferrocenyl ester **5e** via ester hydrolysis followed by conversion to the corresponding acyl chloride and coupling with ferrocenemethanol. Alternatively, when **4ai** was treated with potassium persulfate and CuSO_4_, an easily separable mixture of lactone product **5f** and ketone **5g** was obtained in 52 and 23% yield, respectively, with no erosion of optical purity.^21,22^**4ai** was then reduced to alcohol **5h** in 67% yield using LiAlH_4_ and subsequently converted to corresponding terminal olefin **5i** by Grieco elimination.^23^ The specific rotation of **5i** was then compared to literature data^24^ and used to determine the absolute configuration of compounds **4af**–**4al** (Scheme 4B).

Next, selected oxa-Michael products were converted into valuable drug and natural product intermediates (Scheme 4C). **4d** was directly converted to alcohol **5j**(25) in 98% yield using SmI_2_ and Et_3_N, providing access to lipoic acid (**7**)^26^ precursor **6**(27) and NcRTI inhibitor **8**.^28^ Isochroman product **4aq** could also be reduced according to literature precedent to corresponding alcohol **5k**,^29^ a key intermediate in the synthesis of highly selective D_4_ receptor antagonist Sonepiprazole (**9**).^5d^

**Figure 1:**
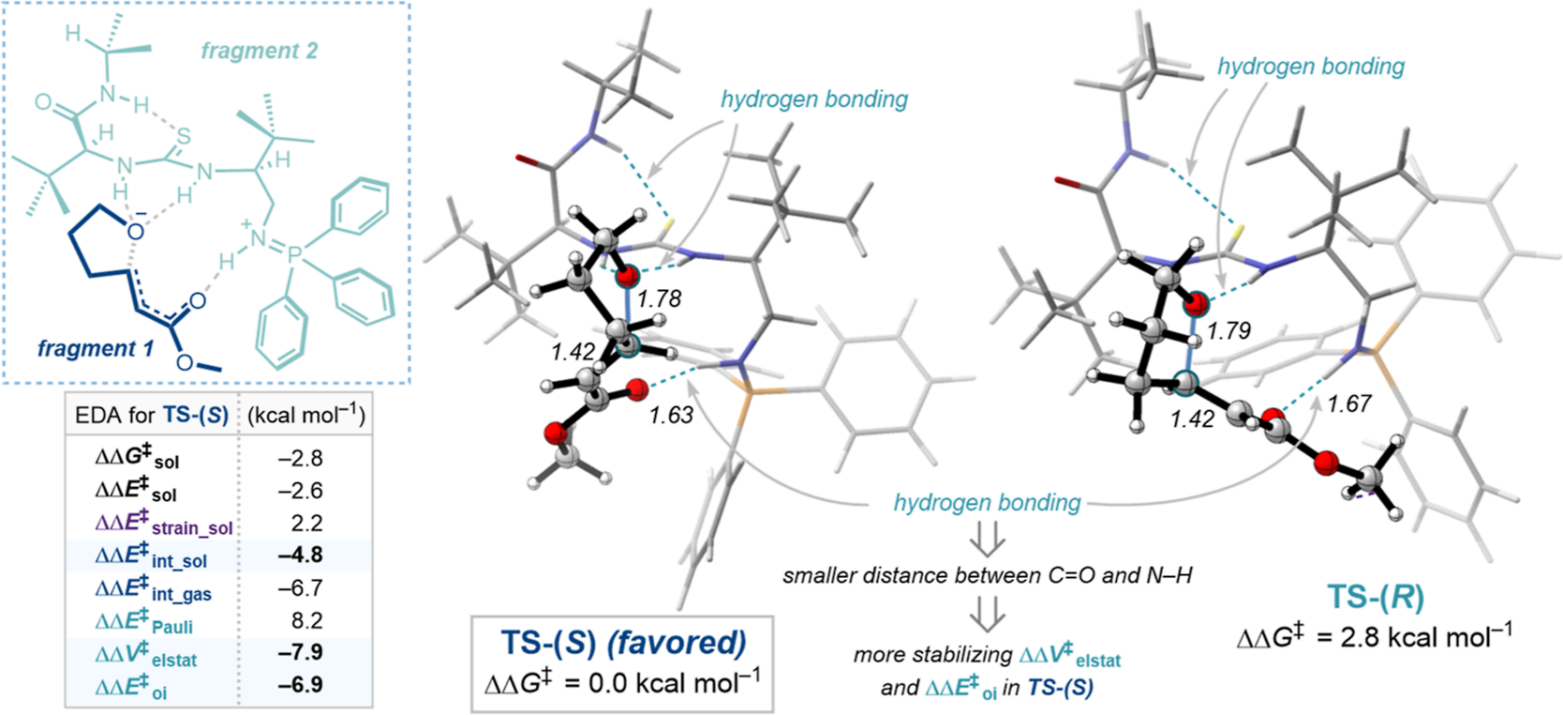
TS structures (relative energies [kcal mol^–1^]) of the BIMP-catalyzed intramolecular oxa-Michael addition computed at COSMO(THF)-ZORA-M06-2X/TZ2P//COSMO(THF)-ZORA-BLYP-D3(BJ)/DZP. Activation strain, EDA values (kcal mol^–1^), and bond lengths (Å) of the TSs are provided in the insert.

To elucidate the origin of stereocontrol in the BIMP-catalyzed intramolecular oxa-Michael reaction to α,β-unsaturated esters, density functional theory (DFT) calculations at COSMO(THF)-ZORA-M06-2X/TZ2P//COSMO(THF)-ZORA-BLYP-D3(BJ)/DZP were performed using Amsterdam Density Functional (ADF) software package (Figure 1).^30^ The proposed enantiodetermining step is the C–O bond-forming intramolecular conjugate addition. An extensive transition-state (TS) search was performed by considering the conformational freedom of the catalyst structure and two potential activation modes using the model substrate **A** and catalyst **B** (see Figures S1–S4 in the Supporting Information for more details).^14f,14g^ Among the combination of possible conformations of the “left arm (LA)” (catalyst side chain with the amide and ^*t*^Bu groups), “right arm (RA)” (catalyst side chain with the imino-phosphorane and ^*t*^Bu groups), and the activation modes, the most favorable transition structures for the formation of both enantiomers are found to be **TS-(*S*)** and **TS-(*R*)**. Common stabilizing intermolecular interactions can be observed in these TSs. The preferred transition structure is **TS-(*S*)** that forms the (*S*)-product (ΔΔ*G*^‡^ = 2.8 kcal mol^–1^), and this is in agreement with the experimentally confirmed absolute stereochemical outcome of the reaction.

Next, to quantitatively reveal the origin of the enantioselectivity of the conjugate addition step, we employed the activation strain model^31^ in conjunction with an energy decomposition analysis (EDA).^32^ The bond energies (Δ*E*_^‡^sol_) of **TS-(*S*)** and **TS-(*R*)** in solution were decomposed into the strain energy (Δ*E*_^‡^strain_sol_) and the interaction energy (Δ*E*_^‡^int_sol_) by fragmentation of the TS geometries into the deprotonated substrate (fragment 1 in Figure 1) and the protonated catalyst (fragment 2 in Figure 1). These analyses identified that the kinetic preference for the formation of the (*S*)-product via **TS-(*S*)** arises due to a more stabilizing relative interaction energy (ΔΔ*E*_^‡^int_sol_ = −4.8 kcal mol^–1^). The decisive role of the interaction energy on the reactivity trends prompted the use of our canonical EDA in the gas phase, which decomposes the Δ*E*_^‡^int_ into three physically meaningful terms: Δ*E*^‡^_Pauli_ = Pauli repulsion; Δ*V*^‡^_elstat_ = electrostatic interactions; and Δ*E*^‡^_oi_ = orbital interactions (see the Supporting Information for details). Inspection of the relative EDA terms shows that the combination of more stabilizing electrostatic and orbital interactions for **TS-(*S*)** is decisive for setting the trend in the relative interaction energies and thus the bonding energies (Figure 1). Further analysis identified that the more stabilizing ΔΔ*V*^‡^_elstat_ of **TS-(*S*)** originates from a smaller distance between the positively charged protonated iminophosphorane and the partially negatively charged carbonyl oxygen. These tightly interacting moieties also enter into stronger hydrogen bonding and thus more stabilizing orbital interactions ΔΔ*E*^‡^_oi_, which also contribute to lower the energy of **TS-(*S*)**. Therefore, the most favorable TS conformation **TS-(*S*)** creates an ideal-fit pocket where the substrate can coordinate with maximum stabilizing interactions during the C–O bond-forming step. Overall, the DFT calculations and subsequent analysis of the stereoselectivity-determining intramolecular conjugate addition provide detailed insights and understanding into the origin of the enantioselectivity for this transformation using a tuneable BIMP catalyst.^33^

## Conclusions

We have developed a unified, enantioselective, metal-free approach to substituted cyclic ethers. Enabled by the superbasic and highly modular BIMP catalyst family, the newly established oxa-Michael addition is highly efficient over a multitude of substrate classes bearing diverse alcohol pronucleophiles and low electrophilicity Michael acceptors. It was demonstrated that BIMP catalysts could efficiently promote known but highly challenging oxa-Michael reactions where previous best-in-class catalysts were found to be recalcitrant, as well smoothly promoting intramolecular oxa-Michael additions of even more demanding substrates. The enantioenriched products obtained were then converted to further attractive enantioenriched building blocks and intermediates en-route to bioactive compounds. Furthermore, DFT calculations were employed to elucidate the origin of stereocontrol by the BIMP catalyst. Activation strain and EDA revealed that the preferred TS structures benefit from a tighter binding between the catalyst and the substrate through an intermolecular hydrogen bonding network that induces stabilizing electrostatic and orbital interactions.

## Supporting Information Available

Further optimization and DFT studies, experimental procedures, characterization data, NMR spectra, HPLC and GC traces, and xyz coordinates (PDF)
