# Supplementary material for: Catalytic Enantioselective Intramolecular Oxa-Michael Reaction to α,β-Unsaturated Esters and Amides
Source: J Am Chem Soc. 2023 May 30;145(23):12771–82. doi: 10.1021/jacs.3c03182 (PMC10273320; doi:10.1021/jacs.3c03182)
Supplement: Supplementary file 1 — ja3c03182_si_001.pdf [file ja3c03182_si_001.pdf]

Supporting Information

Catalytic Enantioselective Intramolecular Oxa-Michael Reaction to α,β-Unsaturated Esters and Amides

Guanglong Su,[1] Michele Formica,[‡1] Ken Yamazaki,[‡1,2] Trevor A. Hamlin[2*] and Darren J. Dixon[1*]

[1] Department of Chemistry, Chemistry Research Laboratory, University of Oxford, Mansfield Road, Oxford OX1 3TA (UK). E-mail: darren.dixon@chem.ox.ac.uk

[2] Department of Theoretical Chemistry, Amsterdam Institute of Molecular and Life Sciences (AIMMS), Amsterdam Center for Multiscale Modeling (ACMM), Vrije Universiteit Amsterdam, De Boelelaan 1083, 1081 HV Amsterdam, The Netherlands. E-mail: t.a.hamlin@vu.nl

Supporting Information

Table of Contents

1. General Experimental Data 1

2. Optimization Results 3

3. Preparation of Azide Catalyst Precursors, phosphines and Catalysts 17

4. Preparation of Starting Materials 20

5. Preparation of Racemic Compounds 109

6. Preparation of Enantioenriched Products 109

7. Determination of Absolute Stereochemical Configuration 157

8. Scale-up and Derivatization 129

9. Computational Studies 173

10. NMR Spectra 214

11. HPLC and GC Traces 310

12. References 361

1. General Experimental Details

Reagents and solvents were purchased at reagent-grade from Acros Organics, Sigma-Aldrich, Alfa Aesar, and Fluorochem and used without further purification unless stated. Solvents for extraction or column chromatography were of technical quality. All water used was purified via a Merck Millipore reverse osmosis purification system prior to use. All reactions were performed under N_2 atmosphere if not stated otherwise. Anhydrous solvents (tetrahydrofuran, toluene, dichloromethane, and diethyl ether) were dried by filtration through activated alumina (Sigma-Aldrich, 58 Å pore size, powder 150 mesh, basic) columns and stored under N_2 atmosphere prior to use. Solvents were removed under reduced pressure using Büchi Rotavapor apparatus.

Thin-layer chromatography was performed on SiO_2-60 UV_{254} coated aluminium sheets from Merck (silica gel 60 F254). Visualization was achieved with a UV lamp at a wavelength of 254 nm, or with a $KMnO_4$ solution.

Flash column chromatography was carried out on silica gel 60 (VWR, 40-63 μm). Solvents for extraction and chromatography were of technical quality. Solvent mixtures are individually reported in parenthesis.

Proton, Carbon, and Fluorine nuclear magnetic resonance (^{1}H, ^{13}C, and ^{19}F NMR) spectra were recorded on Bruker DPX200 (200 MHz) Bruker AVG400 (400/101 MHz), Bruker AVH400 (400/101 MHz), Bruker AVC500 (500/126 MHz) and Bruker AVB500 (500/126 MHz) NMR spectrometers at 25 °C. Chemical shifts (δ) are given in ppm, coupling constants (*J*) in Hz. Peak multiplicities are described as singlet (s), doublet (d), triplet (t), pentet (p), or a combination e.g. doublet of doublets, or as a multiplet over a peak range. Some peaks are described as broad (b). The residual deuterated solvent was used as

internal standard ($CDCl_3$: δ_H = 7.26 ppm; CD_3OD: δ_H = 3.31 ppm; $(CD_3)_2SO$: δ_H = 2.50 ppm). HSQC, COSY and HMBC experiments were used for ^1H and ^{13}C NMR signals assignment where required.

Melting points (m.p.) were determined with a Leica Galen III Hot-stage melting point apparatus and microscope and on a Kofler hot block and are reported uncorrected.

Infrared (IR) spectra were recorded on a Bruker Tensor 27 FT-IR spectrometer as a thin film.

High-resolution mass spectrometry (HR-MS-ESI) was performed on a Bruker µTOF mass spectrometer. The molecular ion (M^+) is reported in *m/z* units.

Chiral HPLC / GC The enantiomeric excesses were determined by HPLC analysis on an Agilent 1200 Series instrument or by GC analysis on an Agilent 7820A instrument employing a chiral stationary phase column specified in the individual experiment and by comparing the samples with the appropriate racemic mixtures.

$[\alpha]_D^T$ Optical rotations were recorded using a Perkin Elmer 341 polarimeter; **$[\alpha]_D^T$** values are reported in 10^{-1} deg·cm^2 g^{-1} ; concentrations (c) are quoted in g/100 mL; D refers to the D-line of sodium (589 nm); temperatures (T) are given in degrees Celsius (°C). (+) and (–) compound number prefixes indicate the sign of the optical rotation.

2 Optimization Results

General procedure for optimization of the intramolecular oxy-Michael reaction of alcohol 3a:

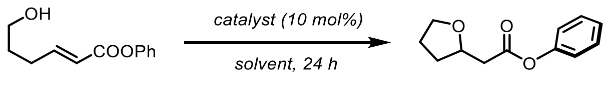

3a **4a**

Alcohol **3a** (10.3 mg, 0.05 mmol, 1.0 eq.) was added to a solution of catalyst (0.005 mmol, 0.1 eq.) in the appropriate solvent under N_2 at room temperature. The reaction mixture was stirred at the specified temperature for 24 hours unless otherwise indicated. The reaction was quenched by the addition of 1M AcOH in CH_2Cl_2 (1 mL). Conversion was determined by crude ^{1}H NMR. Volatiles were removed by a stream of N_2 gas, followed by filtration through silica gel with 30% Et_2O in pentane. The crude product was analysed by chiral HPLC (Chiralcel IB, hexane/isopropanol = 95/5, 1.0 ml/min, λ = 210 nm, t_R (major) = 15.5 min, t_R (minor) = 11.0 min)

Table S1: Catalyst screen[a]

entry	cat. X	Conv. (%)[b]	er[c]	entry	cat. X	Conv. (%)[b]	er[c]
1	**A**	>95	86.5 : 13.5	18	**R**	>95	90.5 : 9.5
2	**B**	>95	84.4 : 15.6	19	**S**	>95	74.5 : 25.5
3	**C**	93%	80 : 20	20	**T**	95	71.5 : 28.5
4	**D**	>95	79 : 21	21	**U**	95	87 : 13
5	**E**	95	81.5 : 18.5	22	**V**	>95	90.5 : 9.5
6	**F**	93	83 : 17	23	**W**	53	76.5 : 23.5
7	**G**	91	84 : 16	24	**X**	>95	84.5 : 15.5
8	**H**	88	80 : 20	25	**Y**	>95	90.5 : 9.5
9	**I**	>95	91 : 9	26	**Z**	>95	79 : 21
10	**J**	95	90 : 10	27			
11	**K**	>95	90 : 10	28			
**12**	**L**	**>95**	**92 : 8**				
13	**M**	>95	88 : 12				
14	**N**	>95	85.5 : 14.5				
15	**O**	>95	83.5 : 16.5				
16	**P**	>95	84 : 16				
17	**Q**	>95	82.5 : 17.5				

Table S1. Detailed catalyst screen results for the intramolecular oxy-Michael reaction of alcohol **3a**. [a] reaction was carried out at room temperature in 0.1 M CPME. [b] determined by ^{1}H NMR analysis of crude reaction mixture. [c] determined by HPLC analysis on chiral stationary phase. CPME = cyclopentyl methyl ether.

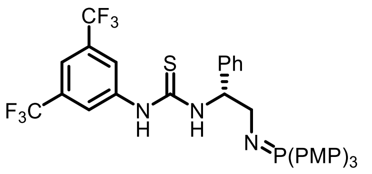

A

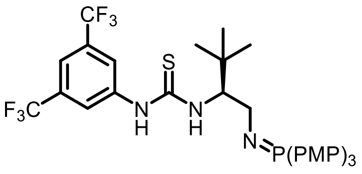

B

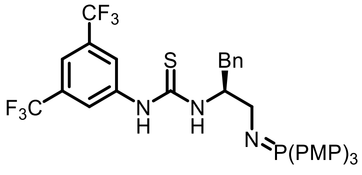

C

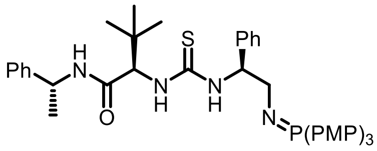

D

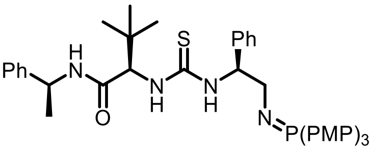

E

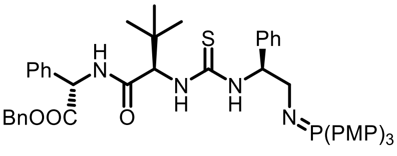

F

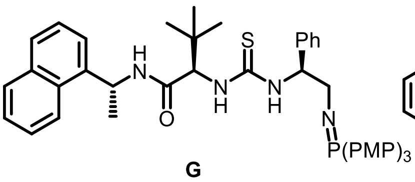

G

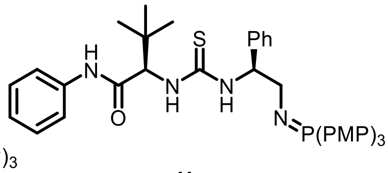

H

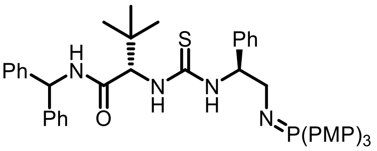

I

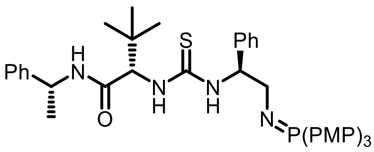

J

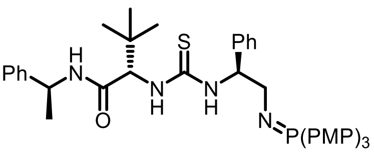

K

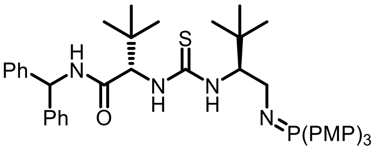

L

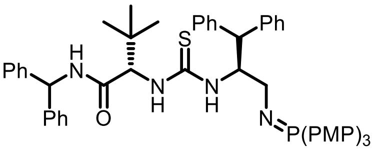

M

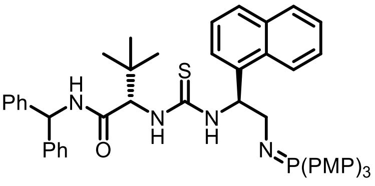

N

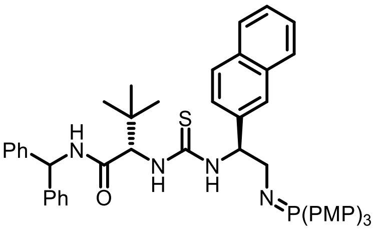

O

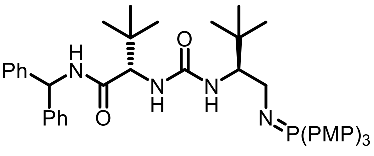

P

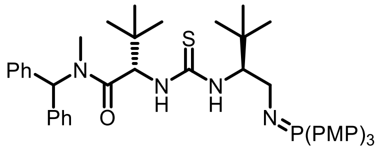

Q

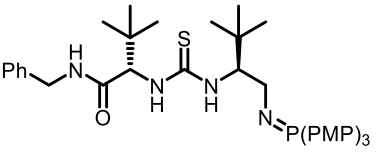

R

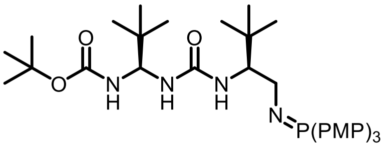

S

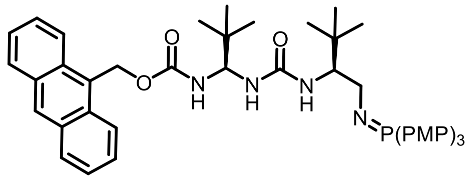

T

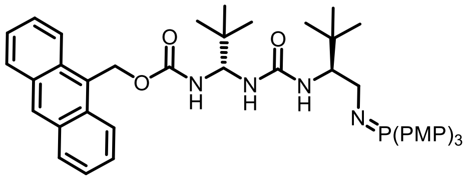

U

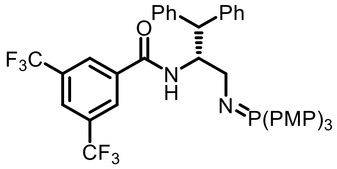

V

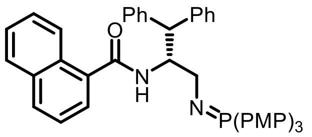

W

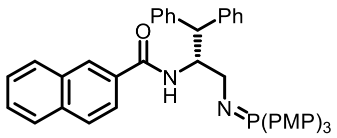

X

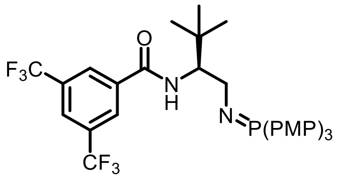

Y

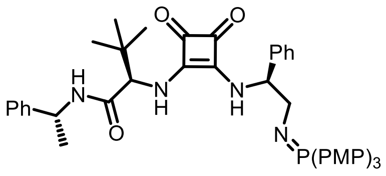

Z

Figure S1. Selected catalysts assessed in the optimization of the intramolecular oxy-Michael reaction of alcohol **3a**.

Table S2: Solvent and conditions optimization

entry	cat. X	Conc.	temp/°C	solvent	Conv. (%)[a]	er[b]
1	**L**	0.1M	rt	CPME	>95	92 : 8
2	**L**	0.1M	rt	Et_2O	>95	92 : 8
3	**L**	0.1M	rt	THF	>95	93 : 7
4	**L**	0.1M	rt	TBME	>95	92.5 : 7.5
**5**	**L**	**0.1M**	**rt**	**2-methyl THF**	**>95**	**93.4 : 6.6**
6	**L**	0.1M	rt	dioxane	>95	89.5 : 10.5
7	**L**	0.1M	rt	CH_2Cl_2	>95	87.5 : 12.5
8	**L**	0.1M	rt	MeCN	>95	89 : 11
9	**L**	0.1M	rt	EtOAc	>95	92 : 8
10	**L**	0.1M	rt	PhMe	>95	85 : 15
11	**L**	0.1M	3	CPME	>95	91.5 : 8.5
12	**L**	0.1M	–22	CPME	>95	91 : 9
13[c]	**L**	0.1M	40	CPME	>95	91 : 9
14	**L**	0.1M	40	CPME	>95	89.5 : 10.5
**15**	**L**	**0.025M**	**rt**	**CPME**	**>95**	**94.5 : 5.5**
16	**L**	0.4M	rt	CPME	>95	85 : 15

Table S2. Detailed solvent and conditions optimization results for the intramolecular oxy-Michael reaction of alcohol **3a**. [a] determined by ^1H NMR analysis of crude reaction mixture. [b] determined by HPLC analysis on chiral stationary phase. [c] reaction was carried out for 8 hours. rt = room temperature. CPME = cyclopentyl methyl ether. TBME = *tert*-butyl methyl ether.

Table S3: Phosphine screen[a]

entry	cat. X	Conc.	PR_3	solvent	Conv. (%)[b]	er[c]
1	**L**	0.1M	$P(PMP)_3$	CPME	>95	92 : 8
4	**L**	0.1M	$P(^nBu)_3$	CPME	>95	88.5 : 11.5
5	**L**	0.1M	I	CPME	>95	91.2 : 8.8
6	**L**	0.1M	II	CPME	>95	90 : 10
7	**L**	0.1M	III	CPME	>95	87 : 13
8	**L**	0.025M	IV	2-methyl THF	40	85 : 15
9	**L**	0.025M	$P(p\text{-Cl-}C_6H_4)_3$	2-methyl THF	6	80 : 20
10	**L**	0.025M	$P(p\text{-tol})_3$	2-methyl THF	59	95.5 : 4.5
11	**L**	0.025M	PPh_3	2-methyl THF	87	96 : 4
**12**	**L**	**0.025M**	**$P(PMP)_3$**	**2-methyl THF**	**>95 (91)[d]**	**95 : 5**
13	**Y**	0.025M	PPh_3	2-methyl THF	18	87 : 13
14	**Y**	0.025M	$P(p\text{-tol})_3$	2-methyl THF	52	92.7 : 7.3
15	**Y**	0.025M	I	2-methyl THF	95	89.6 : 10.4
16	**Y**	0.025M	$P(m\text{-tol})_3$	2-methyl THF	58	90.8 : 9.2

Table S3. Detailed phosphine screen results for the intramolecular oxa-Michael reactions of alcohol **3a**. [a] reaction was carried out at room temperature for 24 hours unless otherwise indicated. [b] determined by ^1H NMR analysis of crude reaction mixture. [c] determined by HPLC analysis on chiral stationary phase. [d] yield of isolated product. PMP = *para*-methoxy phenyl.

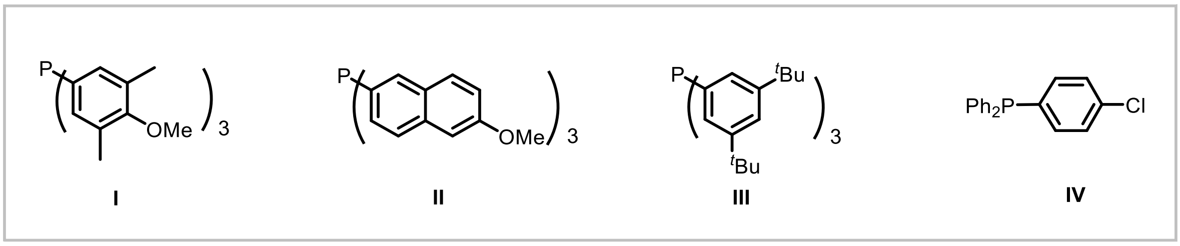

Figure S2. Selected phosphines assessed in the optimization of the intramolecular oxy-Michael reaction of **3a**.

General procedure for optimization of the intramolecular oxy-Michael reaction of alcohol 3af:

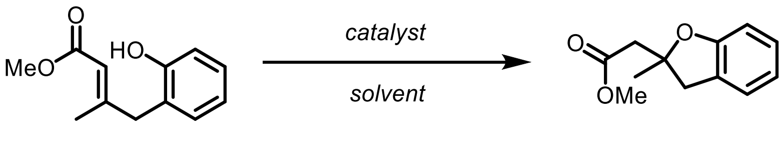

Alcohol **3af** (20.6 mg, 0.1 mmol, 1.0 eq.) was added to a solution of catalyst (0.01 mmol, 0.1 eq. (unless otherwise indicated)) in the appropriate solvent under N_2 at room temperature. The reaction mixture was stirred at the specified temperature for specified time. The reaction was quenched by the addition of 1M AcOH in CH_2Cl_2 (1 mL). Conversion was determined by crude ^1H NMR. Volatiles were removed by a stream of N_2 gas, followed by filtration through silica gel with 30% Et_2O in pentane. The crude product was analysed by chiral HPLC (Chiralcel OD, hexane/isopropanol = 97/3, 1.0 ml/min, λ = 220 nm, t_R (major) = 6.8 min, t_R (minor) = 11.0 min)

Table S4: Optimization result

entry	cat. X	Conc.	temp/°C	time/h	solvent	Conv. (%)[a]	er[b]
1	**L**	0.025M	rt	56	2-methyl THF	56	77.5 : 22.5
2	**L**[c]	0.025M	rt	46	2-methyl THF	51	85 : 15
3	**L**	0.025M	60	46	2-methyl THF	97	66 : 34
4	**L**	0.1M	rt	46	2-methyl THF	98	66.5 : 33.5
**5**	**L**[d]	0.025M	**rt**	46	2-methyl THF	81	76:24
6	**L**[c]	0.05M	rt	24	2-methyl THF	79	79.5 : 20.5
7	**L**[e]	0.025M	rt	24	2-methyl THF	31	N.D.
8	**AC**[e]	0.025M	rt	24	2-methyl THF	18	N.D.
9	**Y**[e]	0.025M	rt	24	2-methyl THF	59	83.5 : 16.5
10	**L**[f]	0.025M	rt	24	2-methyl THF	N.R.	N.D.
11	**L**[g]	0.025M	rt	24	2-methyl THF	N.R.	N.D.
12	**L**[c]	0.025M	rt	24	Et_2O	95	81 : 19
13	**L**[c]	0.025M	rt	24	CPME	91	82 : 18
14	**L**[c]	0.025M	rt	24	dioxane	<5	67 : 33
15	**L**[c]	0.025M	rt	24	toluene	>95	62 : 38
16	**L**[c]	0.025M	rt	24	EtOAc	89	77 : 23
17	**L**[c]	0.025M	rt	24	CH_2Cl_2	>95	60 : 40
18	**M**[c]	0.025M	rt	24	CPME	15	52 : 48
19	**N**[c]	0.025M	rt	24	CPME	77	58 : 42
20	**O**[c]	0.025M	rt	24	CPME	83	66 : 34
21	**I**[c]	0.025M	rt	24	CPME	44	55 : 45
22	**AD**[c]	0.025M	rt	24	CPME	91	77 : 23
23	**L**[h]	0.025M	rt	24	CPME	85	67 : 33
24	**L**[i]	0.025M	rt	24	CPME	11	77 : 23
25	**B**[c]	0.025M	rt	24	CPME	79	62 : 38
26	**AE**	0.025M	rt	24	CPME	45	68 : 32

entry	cat. X	Conc.	temp/°C	time/h	solvent	Conv. (%)[a]	er[b]
27	**C**[c]	0.025M	rt	24	CPME	95	66 : 34
28	**A**[c]	0.025M	rt	24	CPME	46	66 : 34
29	**AF**	0.025M	rt	24	CPME	82	56 : 44
30	**V**[c]	0.025M	rt	24	CPME	55	63 : 37
31	**W**[c]	0.025M	rt	24	CPME	N.R.	N.D.
32	**X**[c]	0.025M	rt	24	CPME	23	63 : 37
33	**Y**[c]	0.025M	rt	24	CPME	>95	64 : 36
34	**AG**	0.025M	rt	24	CPME	>95	63 : 37
35	**AC**	0.025M	rt	24	CPME	62	82.5 : 17.5
36	**AH**	0.025M	rt	24	CPME	>95	81 : 19
37	**AH**[h]	0.025M	rt	24	CPME	>95	81.5 : 18.5
38	**AI**	0.025M	rt	24	CPME	>95	79 : 21
39	**AJ**	0.025M	rt	24	CPME	>95	77 : 23
40	**AJ**[h]	0.025M	rt	24	CPME	>95	83 : 17
41	**AH**[h]	0.025M	rt	24	THF	50	81.5 : 18.5
42	**AH**[h]	0.025M	rt	24	2-methyl THF	83	81 : 19
43	**AH**[h]	0.025M	rt	24	Et_2O	>95	82.5 : 17.5
44	**AH**[h]	0.025M	rt	24	TBME	>95	83.5 : 16.5
45	**AH**[h]	0.025M	rt	24	EtOAc	>95	80 : 20
46	**AC**[h]	0.025M	rt	24	TBME	>95	89 : 11
47	**AC**[j]	0.025M	rt	24	TBME	>95	93 : 7
48	**AC**[j]	**0.025M**	**0**	**24**	**TBME**	**>95 (90)[m]**	**95 : 5**
49	**AC**[j]	0.025M	–20	24	TBME	25	96 : 4
50	**AC**[j] [k]	0.025M	rt	24	TBME	>95	93 : 7
51	**AC**[j] [l]	0.025M	rt	24	TBME	>95	94 : 6
52	**AC**[j] [k]	0.025M	0	24	TBME	52	95.5 : 4.5

Table S4. Detailed optimization results for the intramolecular oxy-Michael reaction of alcohol **3af**. [a] determined by ^{1}H NMR analysis of crude reaction mixture. [b] determined by HPLC analysis on chiral stationary phase. [c] phosphine **I** was used instead of $P(PMP)_3$ to generate the iminophosphorane base. PMP = *para*-methoxy phenyl. [d] 20% cat. used. [e] phosphine **V** was used to generate the iminophosphorane base. [f] phosphine **VI** was used to generate the iminophosphorane base. [g] phosphine **VII** was used to generate the iminophosphorane base. [h] phosphine **III** was used to generate the iminophosphorane base. [i] triphenylphosphine was used to generate the iminophosphorane base. [j] phosphine **VIII** was used to generate the iminophosphorane base. [k] 5% cat. used. [l] 2% cat. used. CPME = cyclopentyl methyl ether. [m] yield of isolated product. TBME = *tert*-butyl methyl ether. N.R. = No Reaction. N.D. = Not Determined.

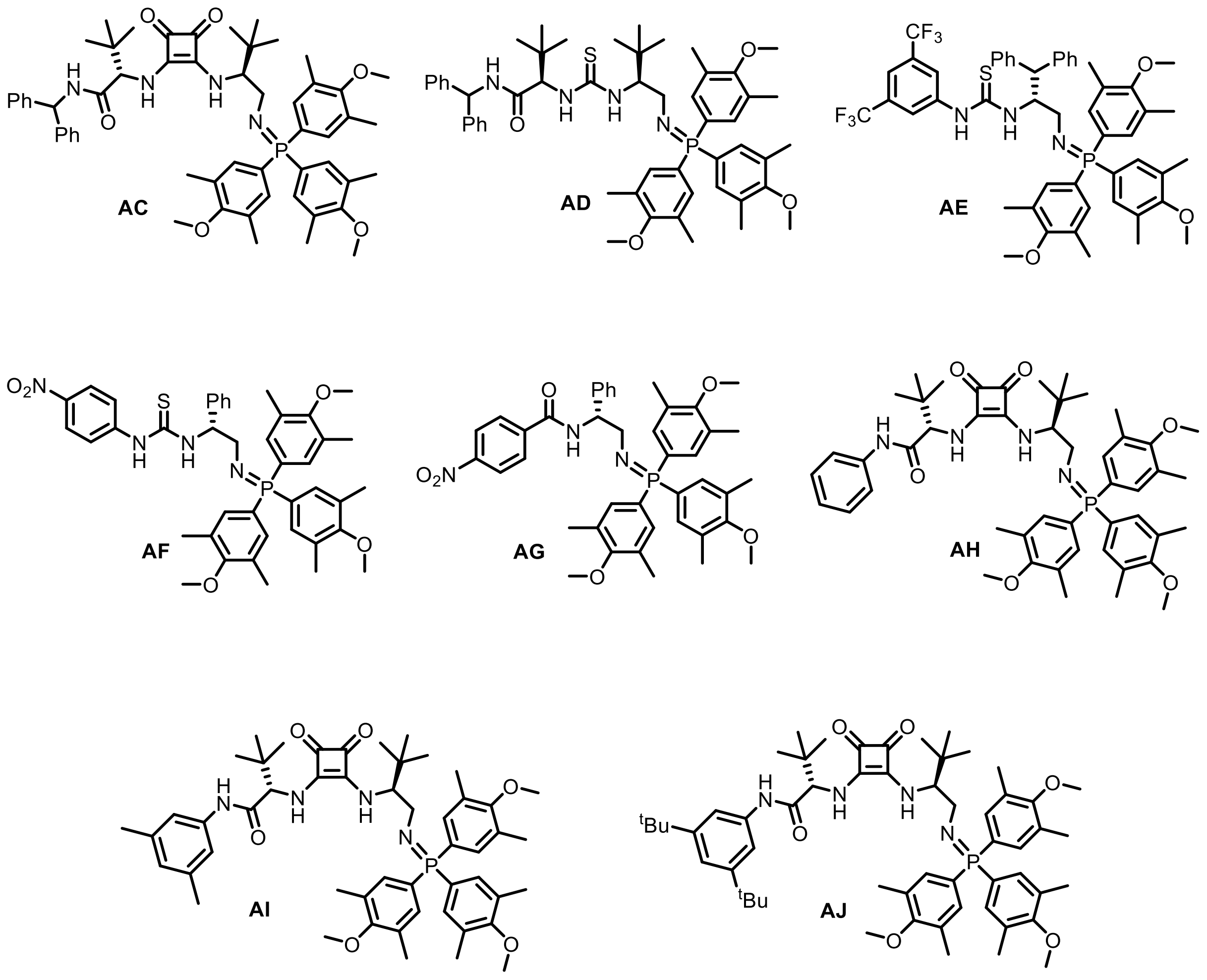

Figure S3. Selected catalysts assessed in the optimization of the intramolecular oxy-Michael reaction of alcohol **3af**.

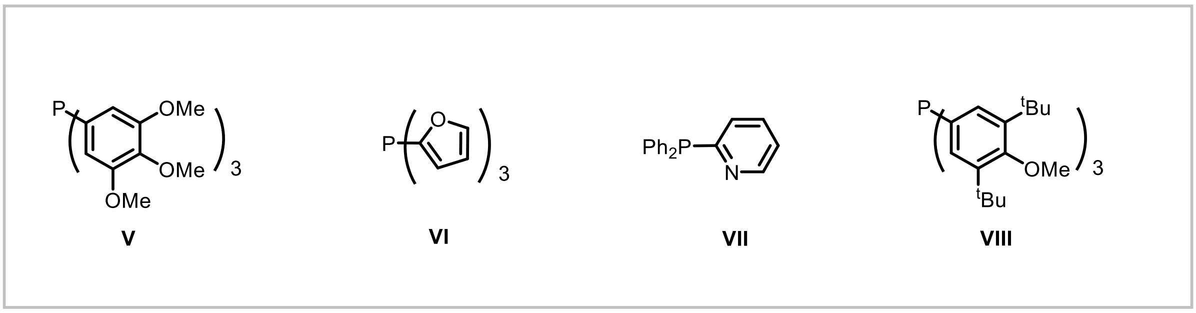

Figure S4. Selected phosphines assessed in the optimization of the intramolecular oxy-Michael reaction of alcohol **3af**.

General Procedure for Optimization of the intramolecular oxy-Michael reaction of alcohol 3am:

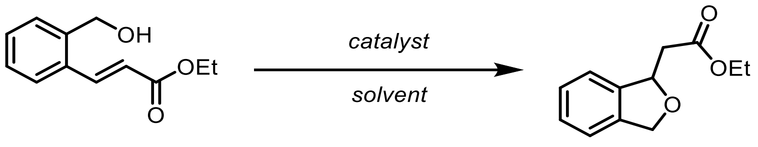

3am **4am**

Alcohol **3am** (20.6 mg, 0.1 mmol, 1.0 eq.) was added to a solution of catalyst (0.005 mmol, 0.05 eq. (unless otherwise indicated)) in the appropriate solvent under N_2 at room temperature. The reaction mixture was stirred at the specified temperature for specified time. The reaction was quenched by the addition of 1M AcOH in CH_2Cl_2 (1 mL). Conversion was determined by crude ^{1}H NMR. Volatiles were removed by a stream of N_2 gas, followed by filtration through silica gel with 30% Et_2O in pentane. The crude product was analysed by chiral HPLC (Chiralcel IA, hexane/isopropanol = 98/2, 1.0 ml/min, λ = 210 nm, t_R (major) = 9.5 min, t_R (minor) = 10.7 min)

Table S5: Optimization result

entry	cat. X	Conc.	temp/°C	time/h	solvent	Conv. (%)[a]	er[b]
1	**L**	0.5M	50	7.5	2-methyl THF	>95	73.5:26.5
2	**L**	0.1M	50	7.5	2-methyl THF	94	81.5:18.5
3	**L**	0.5M	rt	7.5	2-methyl THF	92	82:18
4	**AC**[c]	0.5M	50	7.5	TBME	>95	54 : 46
**5**	**L**	0.025M	rt	16	2-methyl THF	21	85 : 15
6	**L**	0.025M	50	16	2-methyl THF	80	82.5 : 17.5
7	**L**	0.5M	−22	16	2-methyl THF	34	86 : 14
8	**AD**[d]	0.5M	rt	7.5	2-methyl THF	80	75 : 25
9	**L**	0.5M	rt	7.5	toluene	>95	77 : 23
10	**L**	0.5M	rt	7.5	$CF_3C_6H_5$	>95	73.5 : 26.5
11	**L**	0.5M	rt	7.5	CH_2Cl_2	>95	76.5 :23.5
12	**L**	0.5M	rt	7.5	EtOAc	>95	78.5 : 21.5

entry	cat. X	Conc.	temp/°C	time/h	solvent	Conv. (%)[a]	er[b]
13	**Y**	0.5M	rt	7.5	2-methyl THF	85	91 : 9
14	**Y**	0.5M	rt	7.5	THF	74%	91 : 9
15	**Y**	0.5M	rt	7.5	TBME	>95	88.5 : 11.5
16	**Y**	0.5M	rt	7.5	CPME	>95	89 : 11
17	**Y**	0.5M	rt	7.5	Et_2O	>95	88.8 : 11.2
18	**Y**	0.5M	rt	7.5	1,4-dioxane	43%	90 : 10
19	**V**	0.5M	rt	7.5	2-methyl THF	75	86.5 : 13.5
20	**W**	0.5M	rt	7.5	2-methyl THF	N.R.	N.D.
21	**AK**	0.5M	rt	7.5	2-methyl THF	48	77 : 23
22	**AL**	0.5M	rt	7.5	2-methyl THF	67	80.5 : 19.5
23	**AM**	0.5M	rt	7.5	2-methyl THF	>95	84.5 : 15.5
24	**AN**	0.5M	rt	7.5	2-methyl THF	36	64 : 36
25	**Y**[e]	0.5M	rt	7.5	2-methyl THF	>95	93.5 : 6.5
26	**Y**[f]	0.5M	rt	7.5	2-methyl THF	>95	93 : 7
27	**Y**[c]	0.5M	rt	7.5	2-methyl THF	75	91.5 : 8.5
28	**Y**[e]	0.5M	0	24	2-methyl THF	59	96 : 4
29	**Y**[e]	1M	0	7.5	2-methyl THF	26	95.5 : 4.5
30	**Y**[e]	0.1M	rt	24	2-methyl THF	87	95.8 : 4.2
31	**Y**[e]	0.1M	50	7.5	2-methyl THF	88	94 : 6
32	**Y**[e]	0.025M	50	24	2-methyl THF	79	94.5 : 5.5
**33**	**Y**[e]	**0.1M**	**rt**	**24**	**2-methyl THF**	**82**[g]	**95 : 5**

Table S5. Detailed optimization results for intramolecular oxa-Michael reactions of alcohol **3am**. [a] determined by ^{1}H NMR analysis of crude reaction mixture. [b] determined by HPLC analysis on chiral stationary phase. [c] phosphine **VIII** was used to generate the iminophosphorane base. [d] $P(PMP)_3$ was used to generate the iminophosphorane base. PMP = *para*-methoxy phenyl. [e] phosphine **III** was used to generate the iminophosphorane base. [f] phosphine **I** was used to generate the iminophosphorane base. [g] yield of isolated product.

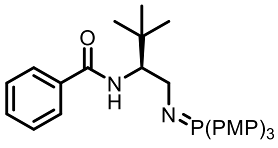

AK

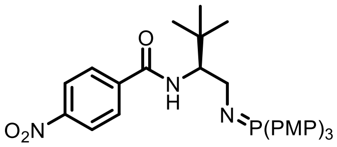

AL

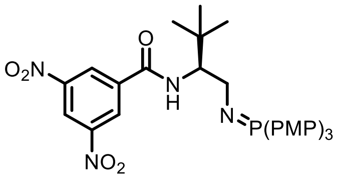

AM

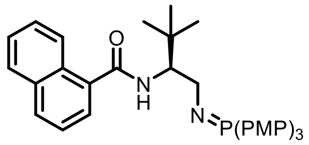

AN

Figure S5. Selected catalysts assessed in the optimization of the intramolecular oxy-Michael reaction of alcohol **3am**.

3 Preparation of Azide Catalyst Precursor, Phosphine and Catalyst

Synthesis of azide catalyst precursor

Catalysts **E** [1] and **K** [2] were prepared according to literature procedures.

Catalyst **I** was prepared according to the following procedure:

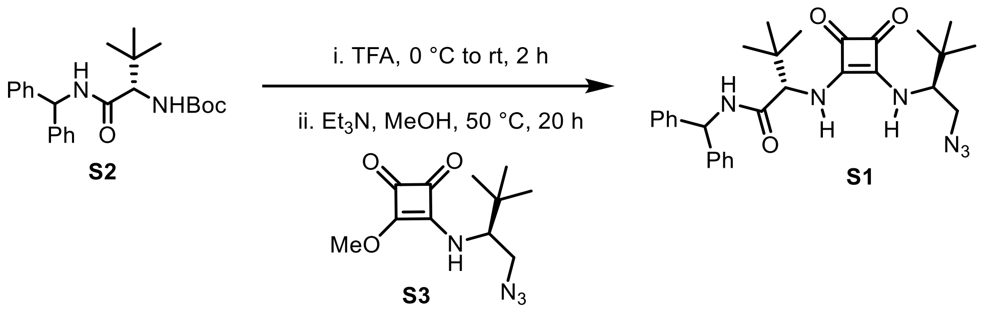

Catalyst Precursor (*S*)-2-((2-(((*S*)-1-azido-3,3-dimethylbutan-2-yl)amino)-3,4-dioxocyclobut-1-en-1-yl)amino)-*N*-benzhydryl-3,3-dimethylbutanamide (S1) was prepared according to the following procedure. According to the modified literature procedure reported by D. M. Rotstein *et. al.,* [3] trifluoroacetic acid (6.0 mL) was added to the *tert*-butyl (*S*)-(1-(benzhydrylamino)-3,3-dimethyl-1-oxobutan-2-yl)carbamate **(S2)** [1] (2.03 g, 5.12 mmol, 1.0 eq.) under N_2 at 0 °C. The reaction mixture was stirred for 2 hours at 0 °C, before warming to room temperature. Volatiles were removed under a stream of nitrogen gas behind a blast shield to afford TFA salt of (*S*)-2-amino-*N*-benzhydryl-3,3-dimethylbutanamide which was used as crude for next step without any purification.

A solution of the TFA salt of (*S*)-2-amino-*N*-benzhydryl-3,3-dimethylbutanamide in anhydrous MeOH (17.0 mL) was added to a solution of (*S*)-3-((1-azido-3,3-dimethylbutan-2-yl)amino)-4-methoxycyclobut-3-ene-1,2-dione [4] **(S3)** (1.29 g, 5.12 mmol, 1.0 eq.) and triethylamine (3.62 g, 35.84 mmol, 7.0 eq.) in anhydrous

MeOH (3.2 mL) under N_2 at room temperature. The reaction mixture was heated to 50 °C and stirred for 20 hours. The reaction mixture was cooled to room temperature and evaporated to dryness under reduced pressure. Purification by silica gel chromatography (CH_2Cl_2/MeCN = 4/1) afforded the title compound as a white solid (2.12 g, 81%).

^{1}H NMR (400 MHz, $(CD_3)_2SO$): δ 9.42 (d, *J* = 8.8 Hz, 1H, NHCO), 7.84 (d, *J* = 10.1 Hz, 1H, NHC), 7.77 (d, *J* = 10.3 Hz, 1H, NHC), 7.42 – 7.20 (m, 10H, Ar-H), 6.21 (d, *J* = 8.8 Hz, 1H, CHNHCO), 4.68 (d, *J* = 10.1 Hz, 1H, CHNHC), 4.01 (td, *J* = 10.1, 3.1 Hz, 1H, CHNHC), 3.50 (dd, *J* = 13.1, 3.2 Hz, 1H, CH_2), 3.38 (dd, *J* = 13.1, 10.1 Hz, 1H, CH_2), 0.91 (s, 9H, $CHC(CH_3)_3$), 0.89 (s, 9H, $CHC(CH_3)_3$) ppm; **^{13}C NMR (101 MHz, $(CD_3)_2SO$)** δ 182.2 (squaramide CO), 182.1 (squaramide CO), 168.6 (amide CO), 167.9 (squaramide CNH), 167.3 (squaramide CNH), 142.2 (ArC), 142.1 (ArC), 128.4 (ArCH), 128.3 (ArCH), 127.4 (ArCH), 127.2 (ArCH), 127.0 (ArCH), 63.4 (CHNHC), 62.6 (CHNHC), 56.0 (CHNHCO), 51.3 (CH_2), 35.8 ($CHC(CH_3)_3$), 34.0 ($CHC(CH_3)_3$), 26.0 (2 x $CHC(CH_3)_3$) ppm; **HRMS** (ESI) m/z calcd. for $C_{29}H_{37}N_6O_3$ ($[M+H]^+$) 517.2922, found 517.2917; **FT-IR (thin film)** ν_{max} 3710, 3628, 3291, 2963, 2359, 2099, 1802, 1651, 1584, 1496, 1459, 1402, 1308, 1274, 1103, 1086, 1002, 943, 908, 890, 823, 746, 703, 694, 686, 677 cm^{-1}; **m.p.**: >230 °C; **$[\alpha]_D^{25}$** = +29.7 (c=0.46, $(CH_3)_2SO$).

Synthesis of phosphine

Phosphines **S4** [4] and **S5** [5] were prepared according to literature procedures.

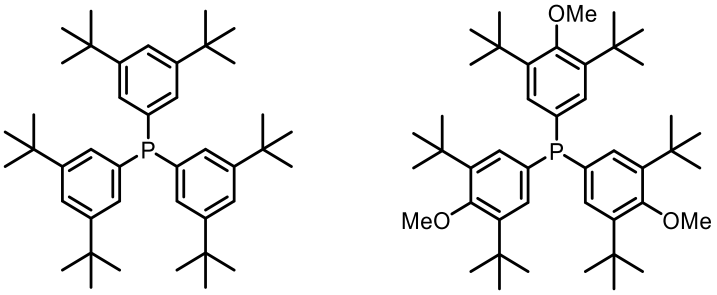

S4 **S5**

Synthesis of catalyst

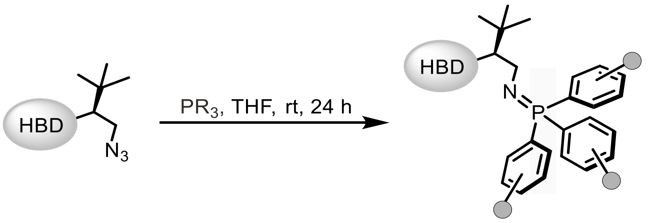

Staudinger reaction for *in-situ* generation of the active BIMP catalysts: THF (0.025 M) was added to the BIMP catalysts precursors (1.0 eq.) and the appropriate phosphine (1.0 eq.) in a mass spectrometry vial under N_2 at room temperature. The reaction mixture was stirred for 24 hours before evaporating to dryness under a stream of nitrogen gas. The iminophosphorane product was confirmed by LCMS and TLC. The resulting mixture was dried under vacuum then used as crude for enantioselective reactions without any purification.

4 Preparation of Starting Materials

Preparation of Wittig reagents S6, S7 and S8

Methyl-, Ethyl-, *iso*-Propyl-, *tert*-Butyl- and Benzyl-(triphenylphosphaneylidene) acetate are commercially available and were used as purchased. Wittig reagents **S6, S7 and S8** were prepared as followed.

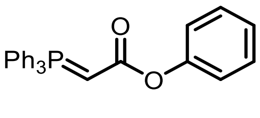

Phenyl-(triphenylphosphaneylidene) acetate (S6) was prepared according to the following procedure. According to the modified literature procedure reported by B. J. Cowen, *et. al.,* [6] a solution of phenyl bromoacetate (10.0 g, 46.5 mmol) in anhydrous $CHCl_3$ (23.3 mL) was slowly added to a solution of triphenylphosphine (12.2 g, 46.5 mmol, 1.0 eq.) in anhydrous $CHCl_3$ (23.3 mL) under N_2 at room temperature (exothermic reaction!). The reaction mixture was stirred for 2 hours at room temperature before evaporating to dryness under reduced pressure. The resulting gum-like mixture was triturated with Et_2O (200 mL) and stood for 15 min. The resulting precipitated salt was filtered, washed with Et_2O (3 x 100 mL) and dried under vacuum.

2M NaOH (aq) (270 mL) was added to a solution of the salt in CH_2Cl_2 (250 mL) under air at room temperature. The reaction mixture was stirred for 10 mins at room temperature. The organic layer was then separated, dried over Na_2SO_4, filtered and evaporated to dryness under reduced pressure, affording the title compound as a white foam which was used as crude for next step without any further purification (17.5 g, 95% yield) (1:0.5 (**A**:**B**) mixture of rotamers). Data is consistent with the published literature.

^{1}H NMR (400 MHz, $CDCl_3$) δ 7.79 – 7.66 (m, 10H, Ar-H (**A**)), 7.58 (tq, J = 6.6, 1.6 Hz, 5H, Ar-H (**A**)), 7.53 – 7.44 (m, 7.5H, Ar-H (**B**)), 7.33 – 7.25 (m, 2H, Ar-H (**A**)), 7.19 – 7.12 (m, 3H, Ar-H (**A**) and Ar-H (**B**)), 7.10 – 7.04 (m, 1H, Ar-H (**B**)), 7.02 – 6.96 (m, 0.5H Ar-H (**B**)), 6.70 – 6.57 (m, 1H, Ar-H (**A**)), 3.24 – 3.15 (m, 1H, CH (**A**)), 3.03 – 2.90 (m, 0.5H CH (**B**)) ppm; **^{31}P-NMR (162 MHz, $CDCl_3$)** δ 18.7 (**A**), 16.9 (**B**) ppm.

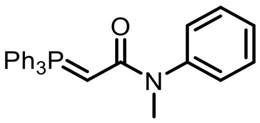

***N*-Methyl-*N*-phenyl-2-(triphenylphosphaneylidene)acetamide (S7)** was prepared according to the following procedure. According to the modified literature procedure reported by D. Lee, *et. al.,* [7] 2-chloroacetyl chloride (3.4 g, 30.0 mmol) was slowly added to a solution of *N*-methylaniline (3.6 g, 33.0 mmol, 1.1 eq.) and triethylamine (3.7 g, 36.6 mmol, 1.22 eq.) in anhydrous $CHCl_3$ (52 mL) under N_2 at 0 °C. The reaction mixture was warmed to room temperature and stirred for 22 hours before quenching with a saturated aqueous solution of $NaHCO_3$ (50 mL). The aqueous layer was extracted with CH_2Cl_2 (3 x 50 mL). The combined organic layers were washed with brine (100 mL), dried over Na_2SO_4, filtered and evaporated to dryness under reduced pressure, affording 2-chloro-*N*-methyl-*N*-phenylacetamide as a light-yellow liquid which was used as crude for next step without any purification (5.6 g, 99% yield).

According to the modified literature procedure reported by K. Diehl, *et. al.,* [8] triphenylphosphine (7.6 g, 29.0 mmol, 1.0 eq.) was added to a solution of 2-chloro-*N*-methyl-*N*-phenylacetamide (5.3 g, 29.0 mmol) in toluene (29 mL) under N_2 at room temperature. The reaction mixture was heated to reflux and stirred

for 24 hours before cooling to room temperature. The resulting precipitated salt was filtered, washed with toluene (2 x 50 mL) and dried under vacuum. Triethylamine (4.4 g, 43.5 mmol, 1.5 eq.) was added to a solution of the salt in $CHCl_3$ (150 mL) under N_2 at room temperature. The reaction mixture was stirred for 3 hours at room temperature before quenching with a saturated aqueous solution of $NaHCO_3$ (100 mL). The aqueous layer was extracted with CH_2Cl_2 (3 x 100 mL). The combined organic layers were washed with brine (100 mL), dried over Na_2SO_4, filtered and evaporated to dryness under reduced pressure, affording the title compound as a brown liquid which was used as crude for next step without any purification (10.6 g, 89% yield). Data is consistent with the published literature.

^{1}H NMR (400 MHz, $CDCl_3$) δ 7.67 – 7.59 (m, 5H, Ar-H), 7.52 – 7.38 (m, 10H, Ar-H), 7.32 (t, *J* = 7.8 Hz, 2H, Ar-H), 7.21 – 7.15 (m, 2H, Ar-H), 7.14 – 7.08 (m, 1H, Ar-H), 3.29 (s, 3H, CH_3), 2.81 – 2.69 (m, 1H, CH) ppm; **^{31}P-NMR (162 MHz, $CDCl_3$)** δ 17.1 ppm.

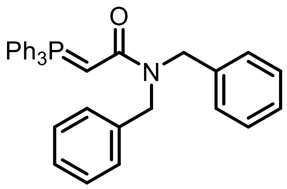

***N, N*-Dibenzyl-2-(triphenylphosphaneylidene)acetamide (S8)** was prepared according to the following procedure. Triphenylphosphine (3.3 g, 12.5 mmol, 1.0 eq.) was added to a solution of *N*, *N*-dibenzyl-2-chloroacetamide (3.4 g, 12.5 mmol) in toluene (21 mL) under N_2 at room temperature. The reaction mixture was heated to 80 °C and stirred for 22 hours before cooling to room temperature. The resulting precipitated salt was filtered, washed with toluene (2 x 50 mL) and dried under vacuum.

1M NaOH (aq) (16 mL) was added to a solution of the salt in CH_2Cl_2 (16 mL) under air at room temperature. The reaction mixture was stirred for 1 hour at room temperature. The aqueous layer was extracted with CH_2Cl_2 (3 x 100 mL). The combined organic layers were washed with brine (100 mL), dried over Na_2SO_4, filtered and evaporated to dryness under reduced pressure, affording the title compound as a pale-yellow solid which was used as crude for next step without any further purification (3.6 g, 58% yield)

^{1}H NMR (400 MHz, $CDCl_3$) δ 7.79 – 7.67 (m, 5H, Ar-H), 7.59 – 7.51 (m, 3H, Ar-H), 7.50 – 7.44 (m, 7H, Ar-H), 7.34 (d, *J* = 4.4 Hz, 8H, Ar-H), 7.29 – 7.23 (m, 2H, Ar-H), 4.58 (s, 4H, CH_2), 3.24 – 2.76 (m, 1H, CH) ppm; **^{31}P-NMR (162 MHz, $CDCl_3$)** δ 18.1 ppm; **HRMS** (ESI) m/z calcd. for $C_{35}H_{30}NOP$ ($[M+H]^+$) 500.2126, found 500.2138; **m.p.**: 124-126 °C.

General information of starting materials for the intramolecular oxy-Michael reaction

Starting materials for the intramolecular oxy-Michael reaction typically contain <10% inseparable Z-isomer.

The Z-isomer exhibited a much slower reaction rate compared to E-isomer.

Starting materials	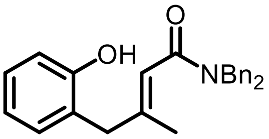 (*E*)- (**3al**)	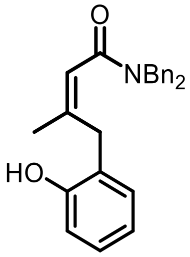 (*Z*)- (**S9**)
Reaction condition	10% cat. **I**, TBME (0.025M), 40 °C, 96 h	
Intramolecular oxy-Michael reaction outcome	67% yield, 98.5:1.5 er	No reaction

General procedure A for preparation of starting materials 3a and 3b

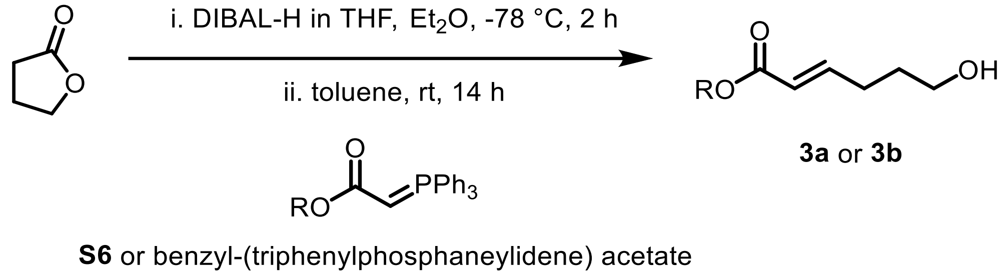

According to the modified literature procedure reported by Y. Kobayashi *et. al.,* [9] DIBAL (4.31 g, 75 mmol, 1.0 M solution in toluene) was slowly added to a solution of dihydrofuran-2-one (3.62 mL, 50 mmol) in Et_2O (50 mL) under N_2 at −78 °C. The reaction mixture was stirred at the same temperature for 2 hours before quenching with MeOH (50 mL) at −78 °C. The resulting mixture was gradually warmed up to room temperature and filtered through a pad of Celite. The filter cake was washed with MeOH (3 x 20 mL) and the combined filtrate was carefully evaporated to dryness under reduced pressure, affording crude tetrahydrofuran-2-ol which was used as crude for next step without any purification. Notably, the yield of this reduction reaction depended on the quality of the reagents used.

Tetrahydrofuran-2-ol (1.0 eq.) in toluene (5 mL) was added to a solution of Phenyl-(triphenylphosphaneylidene) acetate **(S6)** or benzyl-(triphenylphosphaneylidene) acetate (2.5 eq.) in toluene (0.12M) under N_2 at room temperature. The reaction mixture was stirred at room temperature for 14 hours before evaporating to dryness under reduced pressure. Purification by silica gel chromatography afforded starting materials **3a or 3b**.

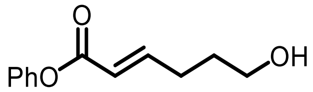

Phenyl (*E*)-6-hydroxyhex-2-enoate (3a) was prepared following **General Procedure A**, using tetrahydrofuran-2-ol (0.93 g, mmol, 10.5 mmol, 1.0 eq.), phenyl-(triphenylphosphoranylidene)acetate **S6** (10.4 g, 26.2 mmol, 2.5 eq.) and toluene (87 mL) to afford the title compound as an colorless oil (2.8 g, 52%). Silica gel chromatography condition: pentane/EtOAc = 3:2. Data is consistent with the published literature.[29]

^{1}H NMR (400 MHz, CDCl$_3$) δ 7.43 – 7.34 (m, 2H, Ar-H), 7.25 – 7.15 (m, 2H, Ar-H), 7.13 – 7.09 (m, 2H, Ar-H and CH), 6.06 (dt, *J* = 15.6, 1.6 Hz, 1H, CH), 3.72 (t, *J* = 6.3 Hz, 2H, CH$_2$), 2.40 (dtd, *J* = 8.3, 7.0, 1.6 Hz, 2H, CH$_2$), 1.79 (ddt, *J* = 8.4, 7.4, 6.4 Hz, 2H, CH$_2$), 1.48 (s, 1H, OH) ppm; **^{13}C NMR (101 MHz, CDCl$_3$)** δ 165.0 (CO), 150.95 (CH), 150.89 (ArC), 129.5 (ArCH), 125.8 (ArCH), 121.8 (ArCH), 121.2 (CH), 62.1 (CH$_2$), 31.0 (CH$_2$), 28.9 (CH$_2$) ppm; **HRMS** (ESI) m/z calcd. for $C_{12}H_{14}O_3Na$ ([M+Na]$^+$) 229.0837, found 229.0835.

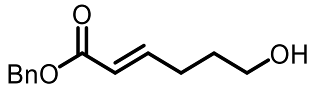

Benzyl (*E*)-6-hydroxyhex-2-enoate (3b) was prepared following **General Procedure A**, using tetrahydrofuran-2-ol (0.45 g, 5 mmol, 2.5 eq.), benzyl-(triphenylphosphoranylidene)acetate (0.82 g, 2.0 mmol, 1.0 eq.) and toluene (6.7 mL) to afford the title compound as an colorless oil (286.4 mg, 65%). Silica gel chromatography condition: pentane/Et_2O = 3:7.

^{1}H NMR (400 MHz, $CDCl_3$) δ 7.42 – 7.26 (m, 5H, Ar-H), 7.03 (dt, *J* = 15.6, 6.9 Hz, 1H, CH), 5.90 (dt, *J* = 15.6, 1.6 Hz, 1H, CH), 5.17 (s, 2H, $COOCH_2$), 3.64 (t, *J* = 6.4 Hz, 2H, CH_2), 2.30 (dtd, *J* = 8.3, 7.0, 1.6 Hz, 2H, CH_2), 1.99 (s, 1H, OH), 1.77 – 1.65 (m, 2H, CH_2) ppm; **^{13}C NMR (101 MHz, $CDCl_3$)** δ 166.5 (CO), 149.4 (CH), 136.2 (ArC), 128.6 (2 x ArCH), 128.3 (ArCH), 121.5 (CH), 66.2 ($COOCH_2$), 61.9 (CH_2), 30.9 (CH_2), 28.6 (CH_2) ppm; **HRMS** (ESI) m/z calcd. for $C_{13}H_{16}O_3Na$ ($[M+Na]^+$) 243.0993, found 243.0992; **FT-IR (thin film)** ν_{max} 3392, 2939, 2360, 2341, 1717, 1653, 1498, 1378, 1319, 1267, 1191, 1097, 1027, 980, 917, 840, 797, 789, 697, 677, 668 cm^{-1}.

Preparation of starting materials 3c and 3d

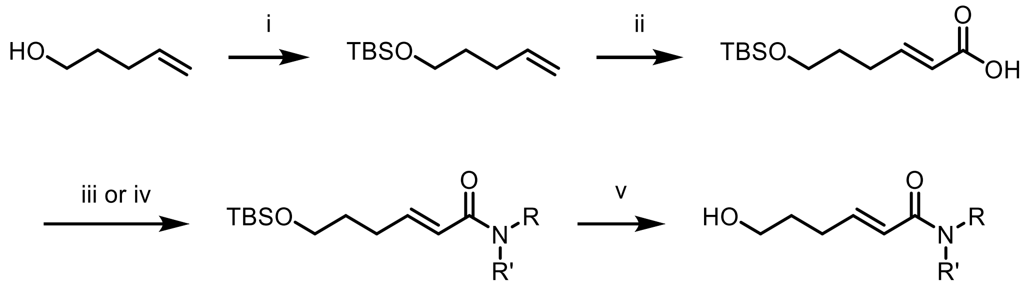

Scheme S1. Synthesis of starting materials **3c** and **3d**. i. TBSCl, imidazole, DMAP, CH_2Cl_2, rt, 1 h; ii. Hoveyda-Grubbs 2nd generation catalyst, acrylic acid, CH_2Cl_2, 45 °C, 16 h; iii. ethyl chloroformate, *N*,*O*-dimethylhydroxylamine hydrochloride, Et_3N, CH_2Cl_2, 0 °C to rt, 1 h; iv. HATU, morpholine, DIPEA, DMF, 0 °C to rt, 16 h; iv. CSA, CH_2Cl_2, MeOH, 4 h.

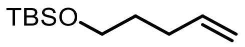

***tert*-Butyldimethyl(pent-4-en-1-yloxy)silane (S10)** was prepared according to the following procedure. According to the modified literature procedure reported by E. M. Stang *et. al.,* [10] *tert*-butyldimethylsilyl chloride (37.3 g, 247.5 mmol, 1.1 eq.), imidazole (23.0 g, 337.5 mmol, 1.5 eq.) and DMAP (1.4 g, 11.25 mmol, 0.05 eq.) were added to a solution of pent-4-en-1-ol (19.4 g, 225 mmol, 1.0 eq.) in CH_2Cl_2 (225 mL) under N_2 at room temperature. The reaction mixture was stirred at room temperature for 1 hour before filtering through a silica plug and eluting with 1% EtOAc/hexanes, affording the title compound as a colorless oil (44.7 g, 99%). Data is consistent with the published literature.

^{1}H NMR (400 MHz, $CDCl_3$) δ 5.82 (ddt, *J* = 16.9, 10.2, 6.6 Hz, 1H, $C\underline{H}=CH_2$), 5.07 – 4.91 (m, 2H, $CH=C\underline{H}_2$), 3.62 (t, *J* = 6.5 Hz, 2H, $C\underline{H}_2CH_2CH_2CH=CH_2$), 2.11 (dtt, *J* = 8.0, 6.6, 1.5 Hz, 2H, $CH_2CH_2C\underline{H}_2CH=CH_2$), 1.61 (ddt, *J* = 8.5, 7.4, 6.5 Hz, 2H, $CH_2C\underline{H}_2CH_2CH=CH_2$), 0.90 (s, 9H, $C(C\underline{H}_3)_3$), 0.05 (s, 6H, $Si(C\underline{H}_3)_2$) ppm; **^{13}C NMR (101 MHz, $CDCl_3$)** δ 138.7 ($\underline{C}H=CH_2$), 114.6 ($CH=\underline{C}H_2$), 62.7 ($\underline{C}H_2CH_2CH_2CH=CH_2$), 32.2 ($CH_2\underline{C}H_2CH_2CH=CH_2$), 30.2 ($CH_2CH_2\underline{C}H_2CH=CH_2$), 26.1 ($C(\underline{C}H_3)_3$), 18.5 ($\underline{C}(CH_3)_3$), -5.1 ($Si(\underline{C}H_3)_2$) ppm.

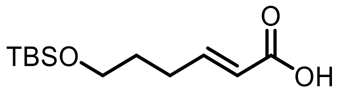

(*E*)-6-((*tert*-Butyldimethylsilyl)oxy)hex-2-enoic acid (S11) was prepared according to the following procedure. A solution of Hoveyda-Grubbs 2nd generation catalyst (31.4 mg, 0.05 mmol, 0.5 mol%) in degassed CH_2Cl_2 (5 mL) was added dropwise (0.5 mL/h) by using a syringe pump to a mixture of ***tert*-Butyldimethyl(pent-4-en-1-yloxy)silane (S10)** (2.0 g, 10 mmol, 1.0 eq.) and acrylic acid (2.2 g, 30 mmol, 3.0 eq.) under N_2 at 45 °C. The reaction mixture was stirred at 45 °C for 16 hours before filtering through a filter paper. The filter cake was washed with CH_2Cl_2 (2 x 10 mL). The combined filtrate was evaporated to dryness under reduced pressure. Purification by silica gel chromatography (20% Et_2O in pentane and 1% AcOH) afforded the title compound as a white solid (1.9 g, 79%).

^{1}H NMR (400 MHz, $CDCl_3$) δ 7.11 (dt, *J* = 15.6, 7.0 Hz, 1H, $CH_2CH_2CH_2C\underline{H}{=}CH$), 5.84 (dt, *J* = 15.6, 1.6 Hz, 1H, $CH_2CH_2CH_2CH{=}C\underline{H}$), 3.64 (t, *J* = 6.2 Hz, 2H, $C\underline{H_2}CH_2CH_2CH{=}CH$), 2.38 – 2.26 (m, 2H, $CH_2CH_2C\underline{H_2}CH{=}CH$), 1.69 (tt, *J* = 7.3, 6.2 Hz, 2H, $CH_2C\underline{H_2}CH_2CH{=}CH$), 0.89 (s, 9H, $C(C\underline{H_3})_3$), 0.05 (s, 6H, $Si(C\underline{H_3})_2$) ppm. (COO$\underline{H}$ could not be observed); **^{13}C NMR (101 MHz, $CDCl_3$)** δ 172.0 ($\underline{C}$(O)O), 152.1 ($CH_2CH_2CH_2\underline{C}H{=}CH$), 120.9 ($CH_2CH_2CH_2CH{=}\underline{C}H$), 62.3 ($\underline{C}H_2CH_2CH_2CH{=}CH$), 31.1 ($CH_2\underline{C}H_2CH_2CH{=}CH$), 29.0 ($CH_2CH_2\underline{C}H_2CH{=}CH$), 26.1 ($C(\underline{C}H_3)_3$), 18.4 ($\underline{C}(CH_3)_3$), -5.2 ($Si(\underline{C}H_3)_2$) ppm; **HRMS** (ESI) m/z calcd. for $C_{12}H_{25}O_3Si$ ($[M+H]^+$) 245.1567, found 245.1568; **FT-IR (thin film)** ν_{max} 3026, 2928, 2856, 1692, 1645, 1470, 1424, 1321, 1289, 1256, 1097, 985, 962, 835, 774, 739, 611 cm^{-1}; **m.p.**: 53-54 °C.

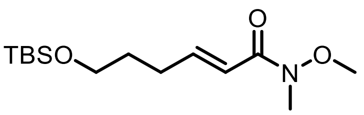

(*E*)-6-((*tert*-Butyldimethylsilyl)oxy)-*N*-methoxy-*N*-methylhex-2-enamide (S12) was prepared according to the following procedure. According to the modified literature procedure reported by K. R. Prasad *et. al.,* [11] Et_3N (333.3 mg, 3.3 mmol, 1.3 eq.) and ethyl chloroformate (325.6 mg, 3.0 mmol, 1.2 eq.) were added to a solution of **(*E*)-6-((*tert*-Butyldimethylsilyl)oxy)hex-2-enoic acid (S11)** (611.1 mg, 2.5 mmol, 1.0 eq.) in CH_2Cl_2 (15 mL) under N_2 at 0 °C. The resulting mixture was stirred at 0 °C for 30 min before adding *N*,*O*-dimethylhydroxylamine hydrochloride (273.2 mg, 2.8 mmol, 1.1 eq.) in one lot, followed by dropwise addition of Et_3N (506.0 mg, 5.0 mmol, 2.0 eq.). The mixture was warmed to room temperature and stirred for 1 h before diluting with EtOAc (10 mL) and H_2O (10 mL). The aqueous layer was extracted with EtOAc (3 × 50 mL). The combined organic layers were washed with 1M HCl (30 mL), brine (30 mL), dried over Na_2SO_4, filtered and evaporated to dryness under reduced pressure. Purification by silica gel chromatography (pentane/Et_2O = 3/2) afforded the title compound as a colorless oil (426.0 mg, 60%). Data is consistent with the published literature.[12]

^{1}H NMR (400 MHz, $CDCl_3$) δ 6.98 (dt, *J* = 15.5, 7.0 Hz, 1H, $CH_2CH_2CH_2C\underline{H}{=}CH$), 6.41 (dt, *J* = 15.4, 1.6 Hz, 1H, $CH_2CH_2CH_2CH{=}C\underline{H}$), 3.69 (s, 3H, $C\underline{H}_3$), 3.63 (t, *J* = 6.2 Hz, 2H, $C\underline{H}_2CH_2CH_2CH{=}CH$), 3.23 (s, 3H, $C\underline{H}_3$), 2.31 (dtd, *J* = 8.3, 7.0, 1.6 Hz, 2H, $CH_2CH_2C\underline{H}_2CH{=}CH$), 1.68 (ddt, *J* = 8.5, 7.4, 6.3 Hz, 2H, $CH_2C\underline{H}_2CH_2CH{=}CH$), 0.89 (s, 9H, $C(C\underline{H}_3)_3$), 0.05 (s, 6H, $Si(C\underline{H}_3)_2$) ppm; **^{13}C NMR (101 MHz, $CDCl_3$)** δ 167.2 ($\underline{C}(O)N$), 147.5 ($CH_2CH_2CH_2\underline{C}H{=}CH$), 119.1 ($CH_2CH_2CH_2CH{=}\underline{C}H$), 62.4 ($\underline{C}H_2CH_2CH_2CH{=}CH$), 61.8 ($\underline{C}H_3$), 32.5 ($\underline{C}H_3$), 31.5 ($CH_2\underline{C}H_2CH_2CH{=}CH$), 29.0 ($CH_2CH_2\underline{C}H_2CH{=}CH$), 26.1 ($C(\underline{C}H_3)_3$), 18.5 ($\underline{C}(CH_3)_3$), -5.2 ($Si(\underline{C}H_3)_2$) ppm.

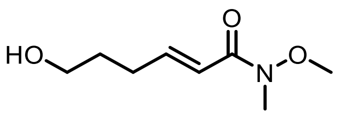

(*E*)-6-Hydroxy-*N*-methoxy-*N*-methylhex-2-enamide (3c) was prepared according to the following procedure. Camphorsulfonic acid (295.0 mg, 1.27 mmol, 1.0 eq.) was added to a solution of **(*E*)-6-((*tert*-Butyldimethylsilyl)oxy)-*N*-methoxy-*N*-methylhex-2-enamide (S12)** (363.9 mg, 1.27 mmol, 1.0 eq.) in CH_2Cl_2 (6 mL) and CH_3OH (6 mL) under N_2 at room temperature. The reaction mixture was stirred at room temperature for 4 hours before quenching with a saturated aqueous solution of $NaHCO_3$ (10 mL). The aqueous layer was extracted with CH_2Cl_2 (3 x 50 mL). The combined organic layers were washed with brine (100 mL),dried over Na_2SO_4, filtered and evaporated to dryness under reduced pressure. Purification by silica gel chromatography (CH_2Cl_2/CH_3OH = 95/5) afforded the title compound as a pale-yellow oil (196.2 mg, 90%).

^{1}H NMR (400 MHz, CDCl$_3$) δ 6.98 (dt, *J* = 15.4, 7.0 Hz, 1H, $CH_2CH_2CH_2C\underline{H}$=CH), 6.43 (dt, *J* = 15.4, 1.6 Hz, 1H, $CH_2CH_2CH_2CH=C\underline{H}$), 3.74 – 3.63 (m, 5H, $C\underline{H_2}CH_2CH_2CH$=CH and $C\underline{H_3}$), 3.23 (s, 3H, $C\underline{H_3}$), 2.34 (dtd, *J* = 8.3, 7.1, 1.6 Hz, 2H, $CH_2CH_2C\underline{H_2}CH$=CH), 1.79 – 1.70 (m, 3H, $CH_2C\underline{H_2}CH_2CH$=CH and O$\underline{H}$); **^{13}C NMR (101 MHz, CDCl$_3$)** δ 167.1 ($\underline{C}$(O)N), 147.1 ($CH_2CH_2CH_2\underline{C}$H=CH), 119.3 ($CH_2CH_2CH_2CH=\underline{C}$H), 62.2 ($\underline{C}H_2CH_2CH_2CH$=CH), 61.8 ($\underline{C}H_3$), 32.5 ($\underline{C}H_3$), 31.4 ($CH_2\underline{C}H_2CH_2CH$=CH), 28.9 ($CH_2CH_2\underline{C}H_2CH$=CH) ppm; **HRMS** (ESI) m/z calcd. for $C_8H_{16}NO_3$ ([M+H]$^+$) 174.1125, found 174.1126; **FT-IR (thin film)** ν_{max} 3409, 2938, 2359, 2341, 1659, 1616, 1427, 1386, 1181, 1113, 1060, 993, 918, 874, 797, 788, 711, 702, 694, 686, 677, 668 cm^{-1}.

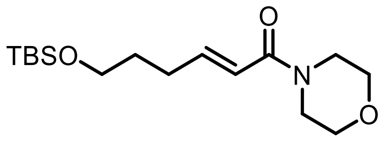

(*E*)-6-((*tert*-Butyldimethylsilyl)oxy)-1-morpholinohex-2-en-1-one (S13) was prepared according to the following procedure. According to the modified literature procedure reported by R. Venkateshwarlu *et. al.,* [13] HATU (24.7 g, 64.9 mmol, 1.1 eq.) and DIPEA (11.5 g, 88.5 mmol, 1.5 eq.) was added to a solution of **(*E*)-6-((*tert*-Butyldimethylsilyl)oxy)hex-2-enoic acid (S11)** (14.4 g, 59.0 mmol, 1.0 eq.) in DMF (295 mL) under N_2 at 0 °C. The resulting mixture was stirred at 0 °C for 5 min before warming to room temperature. Morpholine (5.7 g, 64.9 mmol, 1.1 eq.) was added. The mixture was stirred at room temperature for 16 hours before quenching with a saturated aqueous solution of $NaHCO_3$ (100 mL). The aqueous layer was extracted with CH_2Cl_2 (3 x 200 mL). The combined organic layers were washed with brine (200 mL),dried over Na_2SO_4, filtered and evaporated to dryness under reduced pressure. Purification by silica gel chromatography (pentane/Et_2O= 1/1) afforded the title compound as a pale-orange oil (14.7 g, 80%).

^{1}H NMR (400 MHz, $CDCl_3$) δ 6.89 (dt, *J* = 15.0, 7.0 Hz, 1H, $CH_2CH_2CH_2C\underline{H}$=CH), 6.20 (dt, *J* = 15.1, 1.6 Hz, 1H, $CH_2CH_2CH_2CH$=$C\underline{H}$), 3.80 – 3.37 (m, 10H, $C\underline{H_2}CH_2CH_2CH$=CH and $NC\underline{H_2}C\underline{H_2}O$), 2.26 (dtd, *J* = 8.3, 6.9, 1.6 Hz, 2H, $CH_2CH_2C\underline{H_2}CH$=CH), 1.71 – 1.58 (m, 2H, $CH_2C\underline{H_2}CH_2CH$=CH), 0.87 (s, 9H, $C(C\underline{H_3})_3$), 0.03 (s, 6H, $Si(C\underline{H_3})_2$) ppm; **^{13}C NMR (101 MHz, $CDCl_3$)** δ 165.8 ($\underline{C}$(O)N), 146.8 ($CH_2CH_2CH_2\underline{C}H$=CH), 119.8 ($CH_2CH_2CH_2CH$=$\underline{C}H$), 66.9 (2C, $NCH_2\underline{C}H_2O$), 62.3 ($\underline{C}H_2CH_2CH_2CH$=CH), 46.2 ($N\underline{C}H_2CH_2O$), 42.4 ($N\underline{C}H_2CH_2O$), 31.5 ($CH_2\underline{C}H_2CH_2CH$=CH), 29.0 ($CH_2CH_2\underline{C}H_2CH$=CH), 26.0 ($C(\underline{C}H_3)_3$), 18.4 ($\underline{C}(CH_3)_3$), -5.2 ($Si(\underline{C}H_3)_2$) ppm; **HRMS** (ESI) m/z calcd. for

$C_{16}H_{32}NO_3Si$ ([M+H]$^+$) 314.2146, found 314.2146; **FT-IR (thin film)** ν_{max} 2928, 2856,1660, 1621, 1431, 1361, 1255, 1114, 1025, 977, 836, 776, 715 cm^{-1}.

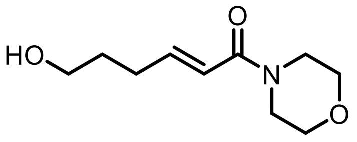

(*E*)-6-Hydroxy-1-morpholinohex-2-en-1-one (3d) was prepared following **the procedure for the synthesis of (*E*)-6-Hydroxy-*N*-methoxy-*N*-methylhex-2-enamide (3c)**, using **(*E*)-6-((*tert*-Butyldimethylsilyl)oxy)-1-morpholinohex-2-en-1-one (S13)** (1.3 g, 4.2 mmol, 1.0 eq.), Camphorsulfonic acid (2.0 g, 8.4 mmol, 2.0 eq.), CH_2Cl_2 (19 mL) and CH_3OH (19 mL) to afford the title compound as an pale-yellow oil (537.0 mg, 64%). Silica gel chromatography condition: CH_2Cl_2/ CH_3OH = 95:5.

^{1}H NMR (400 MHz, CDCl$_3$) δ 6.89 (dt, *J* = 14.5, 7.0 Hz, 1H, $CH_2CH_2CH_2C\underline{H}{=}CH$), 6.23 (dt, *J* = 15.0, 1.6 Hz, 1H, $CH_2CH_2CH_2CH{=}C\underline{H}$), 3.90 – 3.36 (m, 10H, $C\underline{H}_2CH_2CH_2CH{=}CH$ and $NC\underline{H}_2C\underline{H}_2O$), 2.30 (qd, *J* = 7.1, 1.6 Hz, 2H, $CH_2CH_2C\underline{H}_2CH{=}CH$), 1.88 (s, 1H, $O\underline{H}$), 1.77 – 1.66 (m, 2H, $CH_2C\underline{H}_2CH_2CH{=}CH$) ppm; **^{13}C NMR (101 MHz, CDCl$_3$)** δ 165.8 ($\underline{C}(O)N$), 146.5 ($CH_2CH_2CH_2\underline{C}H{=}CH$), 120.0 ($CH_2CH_2CH_2CH{=}\underline{C}H$), 66.9 (2C, $NCH_2\underline{C}H_2O$), 62.0 ($\underline{C}H_2CH_2CH_2CH{=}CH$), 46.2 ($N\underline{C}H_2CH_2O$), 42.4 ($N\underline{C}H_2CH_2O$), 31.4 ($CH_2\underline{C}H_2CH_2CH{=}CH$), 29.0 ($CH_2CH_2\underline{C}H_2CH{=}CH$) ppm; **HRMS** (ESI) m/z calcd. for $C_{10}H_{18}O_3N$ ([M+H]$^+$) 200.1281, found 200.1280; **FT-IR (thin film)** ν_{max} 3394, 2922, 2855, 1656, 1601, 1437, 1301, 1269, 1238, 1114, 1049, 1020, 979, 854, 701 cm^{-1}.

Preparation of starting materials 3e

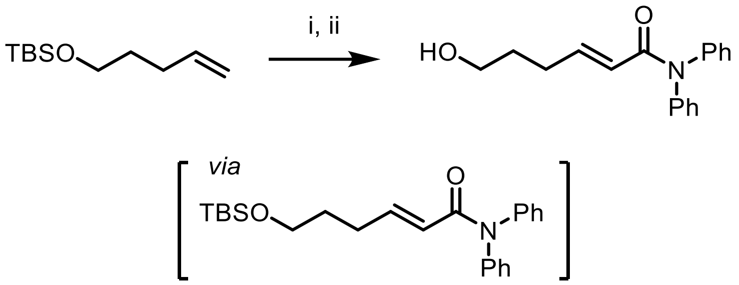

Scheme S2. Synthesis of starting materials **3e**. i. Hoveyda-Grubbs 2nd generation catalyst, acryloyl chloride, CH_2Cl_2, 45 °C, 24 h then diphenylamine, K_3PO_4, 16 h; ii. CSA, CH_2Cl_2, MeOH, 4 h.

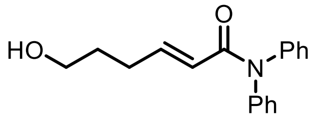

(*E*)-6-Hydroxy-*N*,*N*-diphenylhex-2-enamide (3e) was prepared according to the following procedure. A solution of Hoveyda-Grubbs 2nd generation catalyst (3.2 mg, 0.005 mmol, 0.25 mol%) in degassed CH_2Cl_2 (0.5 mL) was added dropwise (0.5 mL/h) by using a syringe pump to a mixture of ***tert*-Butyldimethyl(pent-4-en-1-yloxy)silane (S10)** (400.4 mg, 2.0 mmol, 1.0 eq.) and acryloyl chloride (290.0 mg, 3.2 mmol, 1.6 eq.) under N_2 at 45 °C. The resulting mixture was stirred at 45 °C for 1 hour. Another 0.5 mL solution of Hoveyda-Grubbs 2nd generation catalyst (3.2 mg, 0.005 mmol, 0.25 mol%) in degassed CH_2Cl_2 was added dropwise (0.5 mL/h) by using a syringe pump to the mixture. The reaction was further stirred for 21 hours at 45 °C before cooling down to room temperature and quenching with diphenylamine (541.5 mg, 3.2 mmol, 1.6 eq.) and K_3PO_4 (1.62 g, 7.6 mmol, 3.8 eq.). The resulting slurry was stirred at room temperature for 16 hours before directly loading onto the silica gel. Purification by silica gel

chromatography (pentane/EtOAc = 4:1) afforded the TBS-protected acrylamide as a pale-yellow oil (192.2 mg, 25%).

^{1}H NMR (400 MHz, $CDCl_3$) δ 7.47 – 7.17 (m, 10H, Ar-H), 7.04 (dt, *J* = 15.1, 7.0 Hz, 1H, $CH_2CH_2CH_2C\underline{H}$=CH), 5.88 (dt, *J* = 15.1, 1.5 Hz, 1H, $CH_2CH_2CH_2CH=C\underline{H}$), 3.58 (t, *J* = 6.2 Hz, 2H, $C\underline{H}_2CH_2CH_2CH$=CH), 2.30 – 2.13 (m, 2H, $CH_2CH_2C\underline{H}_2CH$=CH), 1.71 – 1.52 (m, 2H, $CH_2C\underline{H}_2CH_2CH$=CH), 0.88 (s, 9H, $C(C\underline{H}_3)_3$), 0.03 (s, 6H, $Si(C\underline{H}_3)_2$) ppm; **HRMS** (APCI) m/z calcd. for $C_{24}H_{34}O_2NSi$ ($[M+H]^+$) 396.2353, found 396.2353

TBS removal followed **the procedure for the synthesis of (*E*)-6-Hydroxy-*N*-methoxy-*N*-methylhex-2-enamide (3c)**, using TBS-protected acrylamide (173.1 mg, 0.44 mmol, 1.0 eq.), Camphorsulfonic acid (102.2 mg, 0.44 mmol, 1.0 eq.), CH_2Cl_2 (2 mL) and CH_3OH (2 mL) to afford the title compound as a white solid (110.7 mg, 91%). Silica gel chromatography condition: pentane/ acetone = 7:3.

^{1}H NMR (400 MHz, $CDCl_3$) δ 7.36 (t, *J* = 7.6 Hz, 4H, Ar-H), 7.29 – 7.19 (m, 6H, Ar-H), 7.01 (dt, *J* = 14.6, 7.1 Hz, 1H, $CH_2CH_2CH_2C\underline{H}$=CH), 5.88 (dt, *J* = 15.1, 1.6 Hz, 1H, $CH_2CH_2CH_2CH=C\underline{H}$), 3.58 (t, *J* = 6.4 Hz, 2H, $C\underline{H}_2CH_2CH_2CH$=CH), 2.19 (ddd, *J* = 15.4, 7.4, 1.7 Hz, 2H, $CH_2CH_2C\underline{H}_2CH$=CH), 1.64 (dt, *J* = 8.2, 6.6 Hz, 3H, $CH_2C\underline{H}_2CH_2CH$=CH and O$\underline{H}$) ppm; **^{13}C NMR (101 MHz, $CDCl_3$)** δ 166.2 ($\underline{C}$(O)N), 146.4 ($CH_2CH_2CH_2\underline{C}H$=CH), 142.9 (Ar$\underline{C}$), 129.3 (Ar$\underline{C}$H), 127.6 (Ar$\underline{C}$H), 126.9 (Ar$\underline{C}$H), 123.0 ($CH_2CH_2CH_2CH=\underline{C}H$), 62.1 ($\underline{C}H_2CH_2CH_2CH$=CH), 31.2 ($CH_2\underline{C}H_2CH_2CH$=CH), 28.8 ($CH_2CH_2\underline{C}H_2CH$=CH) ppm; **HRMS** (ESI) m/z calcd. for $C_{18}H_{20}O_2N$ ($[M+H]^+$) 282.1489, found 282.1489; **FT-IR (thin film)** ν_{max} 3659, 2981, 2888, 1664, 1630, 1592, 1491, 1381, 1252, 1154, 1073, 955, 830, 757, 700, 668, 625 cm^{-1}; **m.p.**: 72-73 °C.

Preparation of Starting Materials 3g

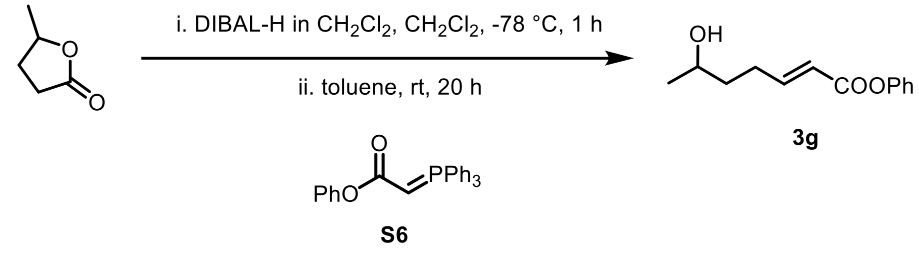

Phenyl (*E*)-6-hydroxyhept-2-enoate (3g) was prepared according to the following procedure. DIBAL (1M solution in CH_2Cl_2) (50 mL, 50 mmol, 2.0 eq.) was added dropwise to a solution of 5-methyldihydrofuran-2(3H)-one (2.5 g, 25 mmol, 1.0 eq.) in CH_2Cl_2 (67 mL) under N_2 at −78 ºC. The reaction mixture was stirred at the same temperature for 1 hour before warming up to room temperature and slowly adding H_2O (2 mL), NaOH (15% aqueous solution, 2 mL) and H_2O (5 mL). The resulting mixture was stirred for 15 mins before drying over $MgSO_4$, filtering and carefully evaporating to dryness under reduced pressure to afford the hemiacetal as a colorless oil which was used as crude for next step without any further purification.

hemiacetal (408.5 mg, 4.0 mmol, 1.0 eq.) was added to a solution of **phenyl-(triphenylphosphoranylidene)acetate S6** (4.76 g, 12 mmol, 3.0 eq.) in toluene (40.3 mL) under N_2 at room temperature. The reaction mixture was stirred at room temperature for 20 hours before evaporating to dryness under reduced pressure. Purification by silica gel chromatography (CH_2Cl_2/EtOAc = 9/1) afforded the title compound as a colorless oil (162.3 mg, 19%).

^{1}H NMR (400 MHz, $CDCl_3$) δ 7.43 – 7.34 (m, 2H, Ar-H), 7.25 – 7.15 (m, 2H, $CH_2C\underline{H}$=CH and Ar-H), 7.14 – 7.09 (m, 2H, Ar-H), 6.05 (dt, *J* = 15.6, 1.6 Hz, 1H, $CH_2CH=C\underline{H}$), 3.87 (qd, *J* = 6.3, 4.6 Hz, 1H, $OHC\underline{H}$), 2.52 – 2.28 (m, 2H, $C\underline{H}_2CH=CH$), 1.66 (td, *J* = 7.7, 5.9 Hz, 2H, $OHCHC\underline{H}_2$), 1.39 (d, *J* = 4.4 Hz,

1H, OH), 1.25 (d, *J* = 6.2 Hz, 3H, CH_3) ppm; **^{13}C NMR (101 MHz, $CDCl_3$)** δ 165.1 (C(O)O), 151.3 (CH_2CH=CH), 150.9 (ArC), 129.5 (ArCH), 125.8 (ArCH), 121.8 (ArCH), 121.0 (CH_2CH=CH), 67.4 (OHCH), 37.3 (OHCHCH_2), 28.9 (CH_2CH=CH), 23.9 (CH_3) ppm; **HRMS** (ESI) m/z calcd. for $C_{13}H_{16}O_3Na$ ($[M+Na]^+$) 243.0992, found 243.0993; **FT-IR (thin film)** ν_{max} 2981, 2359, 2341, 1735, 1651, 1494, 1376, 1257, 1197, 1153, 959, 900, 849, 840, 832, 823, 814, 806, 797, 754, 694, 669 cm^{-1}.

Preparation of Starting Materials 3h

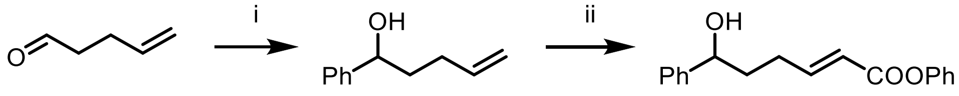

Scheme S3. Synthesis of starting materials **3h**. i. PhMgBr (3M in Et_2O), Et_2O, −78 °C, 1 h; ii. Hoveyda-Grubbs 2nd generation catalyst, phenyl acrylate, CH_2Cl_2, 45 °C, 2 h.

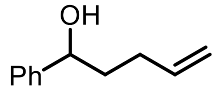

1-Phenylpent-4-en-1-ol (S14) was prepared according to the following procedure. PhMgBr (3M in Et_2O, 1.1 mL, 3.3 mmol, 1.1 eq.) was added dropwise to a solution of pent-4-enal (252.4 mg, 3.0 mmol, 1.0 eq.) in Et_2O (3 mL) under N_2 at −78 °C. The reaction mixture was stirred at −78 °C for 1 hour before warming to room temperature and stirring for 24 hours. The mixture was then quenched with a saturated aqueous solution of NH_4Cl (5 mL). The aqueous layer was extracted with Et_2O (3 x 20 mL). The combined organic layers were washed with brine (20 mL), dried over Na_2SO_4, filtered and carefully evaporated to dryness under reduced pressure. Purification by silica gel chromatography (pentane/Et_2O = 17:3) afforded the title compound as a yellow oil (201.8 mg, 42%). Data is consistent with the published literature.[14]

^{1}H NMR (400 MHz, CDCl$_3$) δ 7.40 – 7.26 (m, 5H, Ar-H), 5.85 (ddt, *J* = 16.9, 10.2, 6.6 Hz, 1H, CH=CH$_2$), 5.05 (dq, *J* = 17.1, 1.7 Hz, 1H, CH=CH$_2$), 4.99 (ddt, *J* = 10.2, 2.2, 1.3 Hz, 1H, CH=CH$_2$), 4.70 (dd, *J* = 7.7, 5.6 Hz, 1H, OHCH), 2.25 – 2.04 (m, 2H, CH$_2$CH=CH$_2$), 2.03 – 1.73 (m, 3H, OHCHCH$_2$) ppm; **^{13}C NMR (101 MHz, CDCl$_3$)** δ 144.8 (ArC), 138.3 (CH=CH$_2$), 128.6 (ArCH), 127.7 (ArCH), 126.0 (ArCH), 115.1 (CH=CH$_2$), 74.2 (OHCH), 38.2 (OHCHCH$_2$), 30.2 (CH$_2$CH=CH$_2$) ppm.

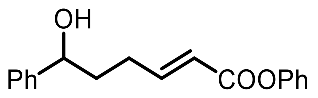

Phenyl (*E*)-6-hydroxy-6-phenylhex-2-enoate (3h) was prepared according to the following procedure. A solution of Hoveyda-Grubbs 2nd generation catalyst (3.1 mg, 0.005 mmol, 0.5 mol%) in degassed CH_2Cl_2 (0.5 mL) was added dropwise (0.5 mL/h) by using a syringe pump to a mixture of **1-Phenylpent-4-en-1-ol (S14)** (162.1 g, 1.0 mmol, 1.0 eq.) and phenyl acrylate (444.2 mg, 3 mmol, 3.0 eq.) under N_2 at 45 °C. The reaction mixture was stirred at 45 °C for 2 hours before directly loading onto the silica gel. Purification by silica gel chromatography (pentane/EtOAc = 4:1) afforded the title compound as an off-white solid (231.4 mg, 82%).

^{1}H NMR (400 MHz, $CDCl_3$) δ 7.46 – 7.34 (m, 6H, Ar-H), 7.33 – 7.28 (m, 1H, Ar-H), 7.25 – 7.14 (m, 2H, Ar-H and $CH_2C\underline{H}$=CH), 7.13 – 7.07 (m, 2H, Ar-H), 6.04 (dt, *J* = 15.6, 1.6 Hz, 1H, $CH_2CH=C\underline{H}$), 4.74 (dd, *J* = 7.8, 5.4 Hz, 1H, $OHC\underline{H}$), 2.53 – 2.27 (m, 2H, $C\underline{H}_2CH=CH$), 2.09 – 1.87 (m, 2H, $OHCHC\underline{H}_2$), 1.85 (s, 1H, $O\underline{H}$) ppm; **^{13}C NMR (101 MHz, $CDCl_3$)** δ 165.0 ($\underline{C}$(O)O), 150.94 ($CH_2\underline{C}H=CH$), 150.89 (Ar$\underline{C}$), 144.3 (Ar$\underline{C}$), 129.5 (Ar$\underline{C}$H), 128.8 (Ar$\underline{C}$H), 128.0 (Ar$\underline{C}$H), 126.0 (Ar$\underline{C}$H), 125.8 (Ar$\underline{C}$H), 121.8 (Ar$\underline{C}$H), 121.1 ($CH_2CH=\underline{C}H$), 73.9 ($OH\underline{C}H$), 37.1 ($OHCH\underline{C}H_2$), 28.8 ($\underline{C}H_2CH=CH_2$) ppm; **HRMS** (ESI) m/z calcd. for $C_{18}H_{18}O_3Na$ ($[M+Na]^+$) 305.1148, found 305.1149; **FT-IR (thin film)** ν_{max} 3654, 2981, 2888, 1737, 1650, 1461, 1383, 1252, 1196, 1148, 1072, 955, 701, 659 cm^{-1}; **m.p.**: 43-44 °C.

Preparation of Starting Materials 3i

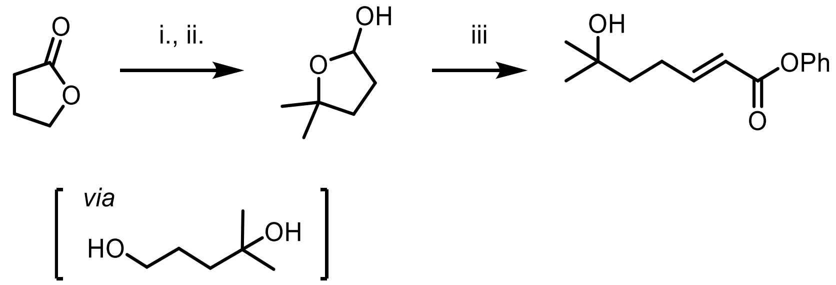

Scheme S4. Synthesis of starting materials **3i**. i. MeMgBr (1M in THF), THF, 0 °C, 2 h; ii. IBX, DMSO, acetone, rt, 2 h; iii. Phenyl-(triphenylphosphoranylidene)acetate **S6**, toluene, rt, 20 h.

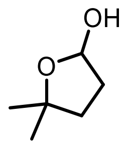

5,5-Dimethyltetrahydrofuran-2-ol (S15) was prepared according to the following procedure. MeMgBr (1M in THF, 50 mL, 50 mmol, 2.5 eq.) was added dropwise to a solution of dihydrofuran-2(3H)-one (1.73 g, 20 mmol, 1.0 eq.) in THF (30 mL) under N_2 at 0 °C. The reaction mixture was stirred at 0 °C for 2 hours before quenching with a saturated aqueous solution of NH_4Cl (10 mL). The aqueous layer was extracted with $CHCl_3$/IPA (3/1) (3 x 100 mL). The combined organic layers were washed with brine (100 mL), dried over Na_2SO_4, filtered and carefully evaporated to dryness under reduced pressure. Purification by silica gel chromatography (acetone) afforded the diol (1.10 g, 47%)

IBX (2.86 g, 10.2 mmol, 1.2 eq.) was added to a mixture of DMSO (7.2 mL) and acetone (27 mL) under N_2 at room temperature. The reaction mixture was stirred for 1 hour before adding the diol. The resulting

mixture was stirred for 1 hour before quenching with H_2O (10 mL). The aqueous layer was extracted with $CHCl_3$/IPA (3/1) (3 x 50 mL). The combined organic layers were washed with brine (50 mL), dried over Na_2SO_4, filtered and carefully evaporated to dryness under reduced pressure. Purification by silica gel chromatography (pentane/EtOAc = 3/1) afforded the title compound as a yellow oil (397.0 mg, 40%). Data is consistent with the published literature.[15]

^{1}H NMR (400 MHz, $CDCl_3$) δ 5.48 (ddd, *J* = 4.3, 2.7, 1.3 Hz, 1H, $C\underline{H}$), 3.40 (dd, *J* = 2.9, 1.3 Hz, 1H, $O\underline{H}$), 2.10 – 1.89 (m, 3H, $CHC\underline{H}_2C\underline{H}_2$), 1.78 – 1.65 (m, 1H, $CHCH_2C\underline{H}_2$), 1.40 (s, 3H, $C\underline{H}_3$), 1.19 (s, 3H, $C\underline{H}_3$) ppm; **^{13}C NMR (101 MHz, $CDCl_3$)** δ 98.8 ($C\underline{H}$), 82.8($O\underline{C}$), 36.3 ($CHCH_2\underline{C}H_2$), 33.8 ($CH\underline{C}H_2CH_2$), 30.4 ($\underline{C}H_3$), 28.7 ($\underline{C}H_3$) ppm.

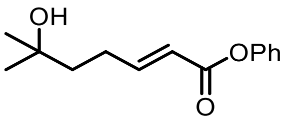

Phenyl (*E*)-6-hydroxy-6-methylhept-2-enoate (3i) was prepared according to the following procedure. **5,5-Dimethyltetrahydrofuran-2-ol (S15)** (376.4 mg, 3.24 mmol, 1.0 eq.) was added to a solution of **phenyl-(triphenylphosphoranylidene)acetate (S6)** (1.29 g, 3.24 mmol, 1.0 eq.) in toluene (10.8 mL) under N_2 at room temperature. The reaction mixture was stirred at room temperature for 20 hours before evaporating to dryness under reduced pressure. Purification by silica gel chromatography (CH_2Cl_2/EtOAc= 9/1) afforded the title compound as a colorless oil (463.0 mg, 61%).

^{1}H NMR (400 MHz, $CDCl_3$) δ 7.43 – 7.33 (m, 2H, Ar-$\underline{H}$), 7.26 – 7.17 (m, 2H, Ar-$\underline{H}$ and $CH_2C\underline{H}$=CH), 7.15 – 7.07 (m, 2H, Ar-$\underline{H}$), 6.05 (dt, *J* = 15.6, 1.6 Hz, 1H, $CH_2CH=C\underline{H}$), 2.45 – 2.35 (m, 2H, $C\underline{H}_2CH=CH$), 1.72 – 1.64 (m, 2H, $C\underline{H}_2CH_2CH=CH$), 1.27 (s, 7H, $C\underline{H}_3$ and $O\underline{H}$) ppm; **^{13}C NMR (101 MHz, $CDCl_3$)** δ

165.1 ($\underline{C}$(O)O), 151.8 ($CH_2\underline{C}H$=CH), 150.9 (Ar$\underline{C}$), 129.5 (Ar$\underline{C}$H), 125.8 (Ar$\underline{C}$H), 121.8 (Ar$\underline{C}$H), 120.7 (CH_2CH=$\underline{C}H$), 70.7 (OH$\underline{C}$), 41.8 ($\underline{C}H_2CH_2CH$=CH), 29.5 ($C\underline{H}_3$), 27.6 ($\underline{C}H_2CH$=CH) ppm; **HRMS** (ESI) m/z calcd. for $C_{14}H_{18}O_3Na$ ($[M+Na]^+$) 257.1148, found 257.1148; **FT-IR (thin film)** ν_{max} 3392, 2980, 2360, 1735, 1651, 1593, 1493, 1378, 1323, 1250, 1196, 1163, 1128, 1071, 968, 927, 815, 754, 721, 688, 668 cm^{-1}.

Preparation of Starting Materials 3k

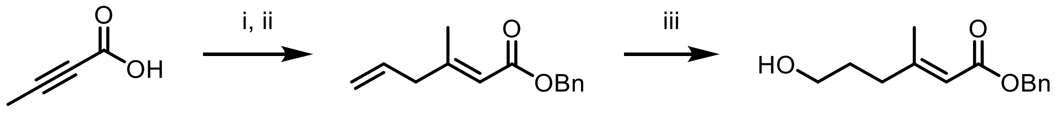

Scheme S5. Synthesis of starting materials **3k**. i. BnBr, K_2CO_3, DMF, rt, 20 h; ii. $Cu(OAc)_2$, allylboronic acid pinacol ester, MeOH, −78 °C for 20 mins then rt 3 h. iii. 9-BBN, −78 °C to rt, 3h then H_2O and $NaBO_3{\cdot}4H_2O$, rt, 20 h.

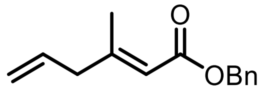

Benzyl (*E*)-3-methylhexa-2,5-dienoate (S16) was prepared according to the following procedure. According to the modified literature procedure reported by Y. Yamamoto *et. al.,* [16] a solution of but-2-ynoic acid (3.00 g, 35.73 mmol, 1.1 eq.) in DMF (21 mL) and BnBr (5.51 g, 32.20 mmol, 1.0 eq.) were added to a suspension of K_2CO_3 (5.78 g, 41.90 mmol, 1.3 eq.) in DMF (32 mL) under N_2 at room temperature. The resulting mixture was stirred at room temperature for 20 hours before diluting with EtOAc (100 mL) and H_2O (300 mL). The aqueous layer was extracted with EtOAc (3 x 100 mL). The combined organic layers were washed with brine (100 mL), dried over Na_2SO_4, filtered and evaporated to dryness under reduced pressure. Purification by silica gel chromatography (pentane/Et_2O = 95/5) afforded the benzyl but-2-ynoate as a colorless oil (5.26 g, 94%).

A solution of benzyl but-2-ynoate (1.43 g, 8.22 mmol, 1.0 eq.), $Cu(OAc)_2$ (45.4 mg, 0.25 mmol, 0.03 eq.) and Allylboronic acid pinacol ester (2.07 g, 12.33 mmol, 1.5 eq.) in anhydrous MeOH (16.5 mL) was degassed at −78 °C for 20 mins. The resulting mixture was warmed to room temperature and stirred for 3

hours under N_2 before evaporating to dryness under reduced pressure. Purification by silica gel chromatography (pentane/Et_2O = 9/1) afforded the title compound as a colorless oil (1.72 g, 97%).

^{1}H NMR (400 MHz, $CDCl_3$) δ 7.44 – 7.28 (m, 5H, Ar-H), 5.85 – 5.71 (m, 2H, CHCH$_2$C and CHC(O)), 5.18 – 5.06 (m, 4H, OCH$_2$ and CH=CH$_2$), 2.88 (dq, *J* = 6.9, 1.3 Hz, 2H, CHCH$_2$C), 2.18 (d, *J* = 1.3 Hz, 3H, CH$_3$) ppm; **^{13}C NMR (101 MHz, $CDCl_3$)** δ 166.6 (C(O)O), 158.8 (CCH$_3$), 136.5 (ArC), 134.3 (CHCH$_2$C or CHC(O)), 128.7 (ArCH), 128.3 (ArCH), 128.2 (ArCH), 118.1 (CH=CH$_2$), 116.2 (CHCH$_2$C or CHC(O)), 65.6 (OCH$_2$), 45.1 (CHCH$_2$C), 19.1 (CCH$_3$) ppm; **HRMS** (ESI) m/z calcd. for $C_{14}H_{17}O_2$ ([M+H]$^+$) 217.1223, found 217.1224; **FT-IR (thin film)** ν_{max} 2981, 1715, 1650, 1455, 1389, 1353, 1215, 1140, 1027, 920, 834, 746, 697 cm^{-1}.

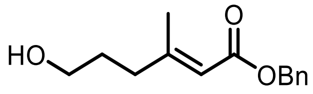

Benzyl (*E*)-6-hydroxy-3-methylhex-2-enoate (3k) was prepared according to the following procedure. According to the modified literature procedure reported by Y. Yamamoto *et. al.,* 9-BBN (0.5M in THF) (15.2 mL, 7.6 mmol, 1.0 eq.) was added to **benzyl (*E*)-3-methylhexa-2,5-dienoate (S16)** (1.64 g, 7.6 mmol, 1.0 eq.) under argon at −78 °C. The mixture was degassed for 5 mins and back filled with argon before warming to room temperature and stirred for 3 hours. H_2O (44.7 mL) and $NaBO_3{\cdot}4H_2O$ (6.00 g, 38.8 mmol, 5.1 eq.) was added under argon at room temperature. The reaction mixture was stirred for 20 hours before extracting with E_2O (3 x 100 mL). The combined organic layers were washed with brine (100 mL), dried over Na_2SO_4, filtered and evaporated to dryness under reduced pressure. Purification by silica gel chromatography (pentane/Et_2O = 3/7) afforded the title compound as a colorless oil (1.03 g, 58%).

^{1}H NMR (400 MHz, $CDCl_3$) δ 7.41 – 7.27 (m, 5H, Ar-H), 5.76 (h, *J* = 1.3 Hz, 1H, CCH), 5.14 (s, 2H, OCH_2), 3.65 (t, *J* = 6.4 Hz, 2H, $OHCH_2CH_2CH_2$), 2.28 – 2.21 (m, 2H, $OHCH_2CH_2CH_2$), 2.21 – 2.17 (m, 3H, CH_3), 1.80 – 1.68 (m, 2H, $OHCH_2CH_2CH_2$), 1.53 (s, 1H, OH) ppm; **^{13}C NMR (101 MHz, $CDCl_3$)** δ 166.6 (C(O)O), 160.3 (CCH_3), 136.5 (ArC), 128.7 (ArCH), 128.3 (ArCH), 128.2 (ArCH), 115.7 (CCH), 65.6 (OCH_2), 62.2 ($OHCH_2CH_2CH_2$), 37.3 ($OHCH_2CH_2CH_2$), 30.4 ($OHCH_2CH_2CH_2$), 19.0 (CCH_3) ppm; **HRMS** (ESI) m/z calcd. for $C_{14}H_{18}O_3Na$ ($[M+Na]^+$) 257.1148, found 257.1148; **FT-IR (thin film)** ν_{max} 2981, 1713, 1645, 1455, 1391, 1219, 1142, 1067, 1015, 745, 697 cm^{-1}.

General procedure B for preparation of starting materials 3f, 3l to 3o and 3q

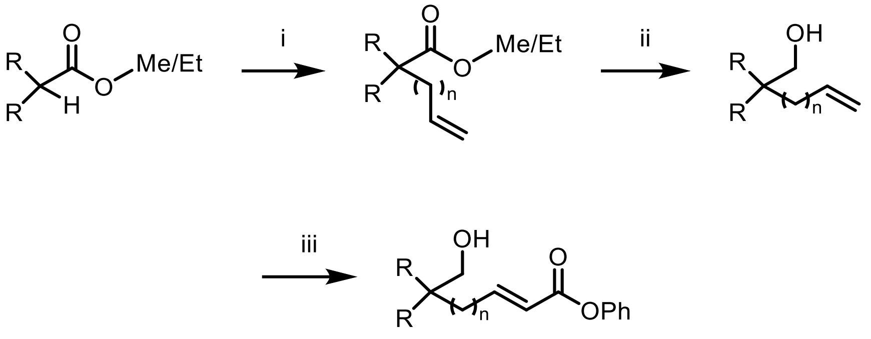

Scheme S6. General Procedure **B** for preparation of starting materials **3f**, **3l** to **3o** and **3q**. i. iPrNH$_2$, n-BuLi, THF, alkylating agent, −78 °C to rt, 16 h; ii. LiAlH$_4$ in THF, Et$_2$O, 0 °C to rt, 1 h; iii. Hoveyda-Grubbs 2nd generation catalyst, phenyl acrylate, CH$_2$Cl$_2$, 45 °C, 3 h.

i. According to the modified literature procedure reported by J. Escudero *et. al.,* [17] ^{n}BuLi (2.5M in hexanes, 1.08 eq.) was added dropwise to a solution of ^{i}PrNH$_2$ (1.1 eq.) in THF (0.4M) under N$_2$ at −78 °C. The mixture was stirred at −78 °C for 15 mins before adding corresponding ester (1 eq.) slowly. The mixture was then stirred at −78 °C for 10 mins before adding corresponding alkylating reagent (1.2 eq.) slowly. The resulting mixture was then warmed to room temperature and stirred for 16 hours before quenching with a saturated aqueous solution of NH$_4$Cl (10 mL). The aqueous layer was extracted with CH$_2$Cl$_2$ (3 x 50 mL). The combined organic layers were washed with brine (100 mL), dried over Na$_2$SO$_4$, filtered and carefully evaporated to dryness under reduced pressure. Purification by silica gel chromatography (pentane/Et$_2$O) afforded the alkyl esters.

ii. A solution of the alkyl esters (1.0 eq.) in Et$_2$O (1M) was added slowly to a solution of LiAlH$_4$ (1.5 eq.) in THF (1M) under N$_2$ at 0 °C. The reaction mixture was stirred at 0 °C for 1 hour before quenching with H$_2$O (5 mL) carefully at 0 °C. The mixture was then warmed to room temperature and more H$_2$O was

added until it became white. The resulting suspension was filtered through a pad of Celite® and carefully evaporated to dryness under reduced pressure to afford the alcohols which were used as crude for next step without any purification.

iii. A solution of Hoveyda-Grubbs 2nd generation catalyst (0.5 mol%) in degassed CH_2Cl_2 (0.01M) was added dropwise (0.5 mL/h) by using a syringe pump to a mixture of the alcohols (1.0 eq.) and phenyl acrylate (3.0 eq.) under N_2 at 45 °C. The reaction mixture was stirred at 45 °C for 3 hours before loading directly onto silica gel. Purification by silica gel chromatography (pentane/EtOAc) afforded starting materials **3f**, **3l** to **3o** and **3q**.

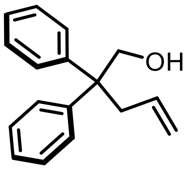

2,2-Diphenylpent-4-en-1-ol (S17) was prepared following **General Procedure B**. Data is consistent with the published literature.[18]

Step i.

Starting Materials: ethyl 2,2-diphenylacetate (2.40 g, 10.0 mmol, 1.0 eq.) and 3-bromoprop-1-ene (1.45 g, 12.0 mmol, 1.2 eq.).

Silica gel chromatography condition: pentane/Et_2O = 95/5.

Product: ethyl 2,2-diphenylpent-4-enoate: colorless oil (2.68 g, 96%).

Step ii.

Starting Materials: ethyl 2,2-diphenylpent-4-enoate (2.36 g, 8.4 mmol, 1.0 eq.)

Product: **2,2-Diphenylpent-4-en-1-ol (S17)**: colorless oil (1.97 g, 99%).

^{1}H NMR (400 MHz, $CDCl_3$) δ 7.38 – 7.16 (m, 10H, Ar-H), 5.45 (ddt, *J* = 17.2, 10.1, 7.1 Hz, 1H, CH=CH_2), 5.15 – 5.05 (m, 1H, CH=CH_2), 5.00 (ddt, *J* = 10.1, 2.3, 1.1 Hz, 1H, CH=CH_2), 4.16 (d, *J* = 6.8 Hz, 2H, CH_2OH), 2.98 (dt, *J* = 7.1, 1.2 Hz, 2H, CH_2CH), 1.21 (t, *J* = 6.9, 1H, OH) ppm; **^{13}C NMR (101 MHz, $CDCl_3$)** δ 145.4 (ArC), 134.7 (CH=CH_2), 128.4 (ArCH), 128.3 (ArCH), 126.5 (ArCH), 118.2 (CH=CH_2), 68.1 (CH_2OH), 51.7 (C), 41.1 (CH_2CH) ppm; **HRMS** (ESI) m/z calcd. for $C_{17}H_{17}O$ ($[M-H]^-$) 237.1279, found 237.0794.

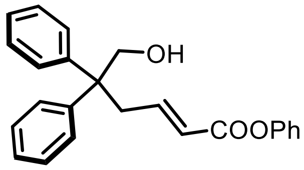

Phenyl (*E*)-6-hydroxy-5,5-diphenylhex-2-enoate (3f) was prepared following **General Procedure B step iii**, using **2,2-Diphenylpent-4-en-1-ol (S17)** (480.8 mg, 2.02 mmol, 1.0 eq.) to afford the title compound as a white solid (514.3 mg, 71%). Silica gel chromatography condition: pentane/Et_2O = 7/3.

^{1}H NMR (400 MHz, $CDCl_3$) δ 7.41 – 7.30 (m, 6H, Ar-H), 7.29 – 7.18 (m, 7H, Ar-H), 7.11 – 7.03 (m, 2H, Ar-H), 6.85 (dt, *J* = 15.3, 7.5 Hz, 1H, $CH_2C\underline{H}$=CH), 6.06 (dt, *J* = 15.6, 1.4 Hz, 1H, $CH_2CH=C\underline{H}$), 4.19 (d, *J* = 6.6 Hz, 2H, $C\underline{H}_2OH$), 3.21 (dd, *J* = 7.5, 1.5 Hz, 2H, $C\underline{H}_2CH=CH$), 1.27 (t, *J* = 6.6 Hz, 1H, O$\underline{H}$) ppm; **^{13}C NMR (101 MHz, $CDCl_3$)** δ 164.5 ($\underline{C}$(O)O), 150.8 (Ar$\underline{C}$), 147.6 ($CH_2\underline{C}H=CH$), 144.5 (2C, Ar$\underline{C}$), 129.4 (2C, Ar$\underline{C}$H), 128.6 (4C, Ar$\underline{C}$H), 128.1 (4C, Ar$\underline{C}$H), 127.0 (2C, Ar$\underline{C}$H), 125.8 (Ar$\underline{C}$H), 123.7 ($CH_2CH=\underline{C}H$), 121.7 (2C, Ar$\underline{C}$H), 68.1 ($\underline{C}H_2OH$), 52.0 ($\underline{C}$), 39.5 ($\underline{C}H_2CH=CH$) ppm; **HRMS** (ESI) m/z calcd. for $C_{24}H_{23}O_3$ ([M+H]$^+$) 359.1642, found 359.1640; **FT-IR (thin film)** ν_{max} 3418, 2980, 2360, 1731, 1650, 1594, 1493, 1445, 1334, 1253, 1195, 1163, 1144, 1068, 1024, 980, 855, 756, 729, 700, 669 cm^{-1}; **m.p.**: 93-94 °C.

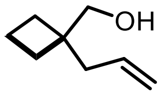

(1-Allylcyclobutyl)methanol (S18) was prepared following **General Procedure B**. Data is consistent with the published literature.[19]

Step i.

Starting Materials: ethyl cyclobutanecarboxylate (1.28 g, 10.0 mmol, 1.0 eq.) and 3-bromoprop-1-ene (1.45 g, 12.0 mmol, 1.2 eq.).

Silica gel chromatography condition: pentane/Et_2O = 95/5.

Product: ethyl 1-allylcyclobutane-1-carboxylate: pale-yellow oil (1.49 g, 89%).

Step ii.

Starting Materials: ethyl 1-allylcyclobutane-1-carboxylate (1.32 g, 7.9 mmol, 1.0 eq.)

Product: **(1-Allylcyclobutyl)methanol (S18)**: pale-orange oil (547.6 mg, 55%).

^{1}H NMR (400 MHz, $CDCl_3$) δ 5.83 (ddt, *J* = 17.4, 10.1, 7.3 Hz, 1H, C<u>H</u>=CH_2), 5.19 – 4.99 (m, 2H, CH=C<u>H</u>$_2$), 3.54 (d, *J* = 5.1 Hz, 2H, C<u>H</u>$_2$OH), 2.26 (dt, *J* = 7.3, 1.3 Hz, 2H, C<u>H</u>$_2$CH), 1.95 – 1.72 (m, 6H, C<u>H</u>$_2$C<u>H</u>$_2$C<u>H</u>$_2$), 1.41 (t, *J* = 5.4 Hz, 1H, O<u>H</u>) ppm; **^{13}C NMR (101 MHz, $CDCl_3$)** δ 135.3 (<u>C</u>H=CH_2), 117.1 (CH=<u>C</u>H$_2$), 68.8 (<u>C</u>H$_2$OH), 42.9 (<u>C</u>), 41.6 (<u>C</u>H$_2$CH), 27.9 (2C, <u>C</u>H$_2$CH$_2$<u>C</u>H$_2$), 15.2 (CH$_2$<u>C</u>H$_2$CH$_2$) ppm; **HRMS** (ESI) m/z calcd. for $C_8H_{14}ONa$ ([M+Na]$^+$) 149.0937, found 149.0122.

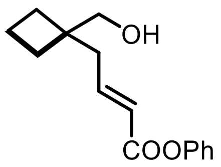

Phenyl (*E*)-4-(1-(hydroxymethyl)cyclobutyl)but-2-enoate (3l) was prepared following **General Procedure B step iii**, using **(1-Allylcyclobutyl)methanol (S18)** (252.2 mg, 2.0 mmol, 1.0 eq.) to afford the title compound as a pale-brown oil (348.7 mg, 71%). Silica gel chromatography condition: pentane/EtOAc = 4/1.

^{1}H NMR (400 MHz, $CDCl_3$) δ 7.43 – 7.34 (m, 2H, Ar-H), 7.25 – 7.20 (m, 1H, Ar-H), 7.20 – 7.09 (m, 3H, Ar-H and $CH_2C\underline{H}$=CH), 6.10 (dt, *J* = 15.6, 1.5 Hz, 1H, $CH_2CH=C\underline{H}$), 3.59 (d, *J* = 4.9 Hz, 2H, $C\underline{H_2}OH$), 2.50 (dd, *J* = 7.7, 1.4 Hz, 2H, $C\underline{H_2}CH$), 1.99 – 1.80 (m, 6H, $C\underline{H_2}C\underline{H_2}C\underline{H_2}$), 1.51 – 1.40 (m, 1H, O$\underline{H}$) ppm; **^{13}C NMR (101 MHz, $CDCl_3$)** δ 164.9 ($\underline{C}$(O)O), 150.9 (Ar$\underline{C}$), 148.3 ($CH_2\underline{C}H$=CH), 129.5 (Ar$\underline{C}$H), 125.8 (Ar$\underline{C}$H), 122.9 ($CH_2CH=\underline{C}H$), 121.8 (Ar$\underline{C}$H), 68.4 ($\underline{C}H_2OH$), 43.1 ($\underline{C}$), 39.8 ($\underline{C}H_2CH$), 28.1 (2C, $\underline{C}H_2CH_2\underline{C}H_2$), 15.3 ($CH_2\underline{C}H_2CH_2$) ppm; **HRMS** (ESI) m/z calcd. for $C_{15}H_{19}O_3$ ($[M+H]^+$) 247.1329, found 247.1330; **FT-IR (thin film)** ν_{max} 3418, 2980, 2360, 2341, 1733, 1650, 1592, 1493, 1384, 1336, 1251, 1196, 1163, 1025, 984, 814, 779, 754, 720, 703, 687, 677, 668 cm^{-1}.

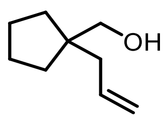

(1-Allylcyclopentyl)methanol (S19) was prepared following **General Procedure B**. Data is consistent with the published literature.[17]

Step i.

Starting Materials: methyl cyclopentanecarboxylate (1.28 g, 10.0 mmol, 1.0 eq.) and 3-bromoprop-1-ene (1.45 g, 12.0 mmol, 1.2 eq.).

Silica gel chromatography condition: pentane/Et_2O = 98/2.

Product: methyl 1-allylcyclopentane-1-carboxylate: pale-yellow oil (1.46 g, 95%).

Step ii.

Starting Materials: methyl 1-allylcyclopentane-1-carboxylate (1.24 g, 8.0 mmol, 1.0 eq.)

Product: **(1-Allylcyclopentyl)methanol (S19)**: colorless oil (0.88 g, 78%).

^{1}H NMR (400 MHz, $CDCl_3$) δ 5.86 (ddt, *J* = 17.4, 10.1, 7.4 Hz, 1H, C<u>H</u>=CH_2), 5.13 – 5.01 (m, 2H, CH=C<u>H_2</u>), 3.42 – 3.37 (m, 2H, C<u>H_2</u>OH), 2.16 (dt, *J* = 7.4, 1.3 Hz, 2H, C<u>H_2</u>CH), 1.68 – 1.51 (m, 4H, C<u>H_2</u> (cyclopentane)), 1.51 – 1.34 (m, 5H, O<u>H</u> and C<u>H_2</u> (cyclopentane)) ppm; **^{13}C NMR (101 MHz, $CDCl_3$)** δ 136.4 (<u>C</u>H=CH_2), 117.1 (CH=<u>C</u>H_2), 69.2 (<u>C</u>H_2OH), 47.4 (<u>C</u>), 42.2 (<u>C</u>H_2CH), 34.3 (2C, <u>C</u>H_2 (cyclopentane)), 25.3 (2C, <u>C</u>H_2 (cyclopentane)) ppm.

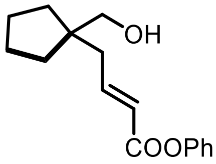

Phenyl (*E*)-4-(1-(hydroxymethyl)cyclopentyl)but-2-enoate (3m) was prepared following **General Procedure B step iii**, using **(1-Allylcyclopentyl)methanol (S19)** (160.2 mg, 1.2 mmol, 1.0 eq.) to afford the title compound as a pale-yellow oil (183.4 mg, 62%). Silica gel chromatography condition: pentane/EtOAc = 17/3.

^{1}H NMR (400 MHz, CDCl$_3$) δ 7.43 – 7.34 (m, 2H, Ar-H), 7.25 – 7.14 (m, 2H, Ar-H and CH$_2$CH=CH), 7.14 – 7.09 (m, 2H, Ar-H), 6.08 (dt, *J* = 15.5, 1.4 Hz, 1H, CH$_2$CH=CH), 3.44 (s, 2H, CH$_2$OH), 2.40 (dd, *J* = 7.8, 1.4 Hz, 2H, CH$_2$CH), 1.72 – 1.58 (m, 4H, CH$_2$ (cyclopentane)), 1.57 – 1.41 (m, 5H, OH and CH$_2$ (cyclopentane)) ppm; **^{13}C NMR (101 MHz, CDCl$_3$)** δ 164.9 (C(O)O), 150.9 (ArC), 149.3 (CH$_2$CH=CH), 129.5 (ArCH), 125.8 (ArCH), 122.8 (CH$_2$CH=CH), 121.8 (ArCH), 68.8 (CH$_2$OH), 47.8 (C), 40.2 (CH$_2$CH), 34.4 (2C, CH$_2$ (cyclopentane)), 25.3 (2C, CH$_2$ (cyclopentane)) ppm; **HRMS** (ESI) m/z calcd. for $C_{16}H_{21}O_3$ ([M+H]$^+$) 261.1485, found 261.1485; **FT-IR (thin film)** ν_{max} 3434, 2950, 2868, 1732, 1650, 1592, 1492, 1316, 1249, 1195, 1163, 1149, 1118, 1038, 981, 815, 778, 750, 688 cm^{-1}.

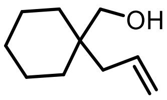

(1-Allylcyclohexyl)methanol (S20) was prepared following **General Procedure B**. Data is consistent with the published literature.[17]

Step i.

Starting Materials: methyl cyclohexanecarboxylate (1.43 g, 10.0 mmol, 1.0 eq.) and 3-bromoprop-1-ene (1.45 g, 12.0 mmol, 1.2 eq.).

Silica gel chromatography condition: pentane/Et_2O = 95/5.

Product: methyl 1-allylcyclohexane-1-carboxylate: colorless oil (1.67 g, 92%).

Step ii.

Starting Materials: methyl 1-allylcyclohexane-1-carboxylate (1.45 g, 8.0 mmol, 1.0 eq.)

Product: **(1-Allylcyclohexyl)methanol (S20)**: colorless oil (1.05 g, 85%).

^{1}H NMR (400 MHz, $CDCl_3$) δ 5.93 – 5.80 (m, 1H, $C\underline{H}=CH_2$), 5.11 – 5.02 (m, 2H, $CH=C\underline{H}_2$), 3.42 (d, *J* = 5.9 Hz, 2H, $C\underline{H}_2OH$), 2.12 (dt, *J* = 7.6, 1.2 Hz, 2H, $C\underline{H}_2CH$), 1.52 – 1.40 (m, 5H, $C\underline{H}_2$ (cyclohexane)), 1.39 – 1.28 (m, 6H, $O\underline{H}$ and $C\underline{H}_2$ (cyclohexane)) ppm; **^{13}C NMR (101 MHz, $CDCl_3$)** δ 135.5 ($\underline{C}H=CH_2$), 117.2 ($CH=\underline{C}H_2$), 69.0 ($\underline{C}H_2OH$), 40.2 ($\underline{C}H_2CH$), 37.9 ($\underline{C}$), 32.5 (2C, $\underline{C}H_2$ (cyclohexane)), 26.5 ($\underline{C}H_2$ (cyclohexane)), 21.6 (2C, $\underline{C}H_2$ (cyclohexane)) ppm; **HRMS** (ESI) m/z calcd. for $C_{10}H_{19}O$ ($[M+H]^+$) 155.1430, found 155.1432.

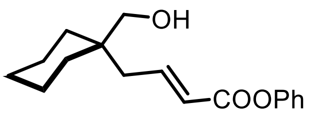

Phenyl (*E*)-4-(1-(hydroxymethyl)cyclohexyl)but-2-enoate (3n) was prepared following **General Procedure B step iii**, using **(1-Allylcyclohexyl)methanol (S20)** (308.3 mg, 2.0 mmol, 1.0 eq.) to afford the title compound as a white solid (413.4 mg, 76%). Silica gel chromatography condition: pentane/EtOAc = 17/3.

^{1}H NMR (400 MHz, $CDCl_3$) δ 7.44 – 7.33 (m, 2H, Ar-H), 7.26 – 7.16 (m, 2H, Ar-H and $CH_2C\underline{H}$=CH), 7.16 – 7.09 (m, 2H, Ar-H), 6.07 (dt, *J* = 15.5, 1.4 Hz, 1H, CH_2CH=C$\underline{H}$), 3.47 (s, 2H, $C\underline{H}_2OH$), 2.37 (dd, *J* = 8.0, 1.4 Hz, 2H, $C\underline{H}_2CH$), 1.49 (tt, *J* = 6.3, 4.2 Hz, 5H, $C\underline{H}_2$ (cyclohexane)), 1.44 – 1.32 (m, 6H, O$\underline{H}$ and $C\underline{H}_2$ (cyclohexane)) ppm; **^{13}C NMR (101 MHz, $CDCl_3$)** δ 164.8 ($\underline{C}$(O)O), 150.9 (Ar$\underline{C}$), 148.8 ($CH_2\underline{C}$H=CH), 129.5 (Ar$\underline{C}$H), 125.8 (Ar$\underline{C}$H), 122.9 (CH_2CH=$\underline{C}$H), 121.8 (Ar$\underline{C}$H), 68.4 ($\underline{C}H_2OH$), 38.8 ($\underline{C}$), 38.2 ($\underline{C}H_2CH$), 32.5 (2C, $\underline{C}H_2$ (cyclohexane)), 26.3 ($\underline{C}H_2$ (cyclohexane)), 21.6 (2C, $\underline{C}H_2$ (cyclohexane)) ppm; **HRMS** (ESI) m/z calcd. for $C_{17}H_{23}O_3$ ([M+H]$^+$) 275.1642, found 275.1642; **FT-IR (thin film)** ν_{max} 3411, 2927, 2858, 1731, 1649, 1592, 1492, 1454, 1313, 1250, 1196, 1163, 1134, 1117, 1027, 985, 927, 815, 777, 737, 688, 628 cm^{-1}; **m.p.**: 48-49 °C.

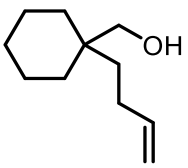

(1-(But-3-en-1-yl)cyclohexyl)methanol (S21) was prepared following **General Procedure B**. Data is consistent with the published literature.[20]

Step i.

Starting Materials: methyl cyclohexanecarboxylate (1.43 g, 10.0 mmol, 1.0 eq.) and 4-bromobut-1-ene (1.61 g, 12.0 mmol, 1.2 eq.).

Silica gel chromatography condition: pentane/Et_2O = 96/4.

Product: methyl 1-(but-3-en-1-yl)cyclohexane-1-carboxylate: colorless oil (1.90 g, 97%).

Step ii.

Starting Materials: methyl 1-(but-3-en-1-yl)cyclohexane-1-carboxylate (1.54 g, 7.8 mmol, 1.0 eq.)

Product: **(1-(But-3-en-1-yl)cyclohexyl)methanol (S21):** colorless oil (1.24 g, 94%).

^{1}H NMR (400 MHz, $CDCl_3$) δ 5.84 (ddt, *J* = 16.8, 10.1, 6.6 Hz, 1H, $C\underline{H}=CH_2$), 5.02 (dq, *J* = 17.1, 1.7 Hz, 1H, $CH=C\underline{H_2}$), 4.93 (ddt, *J* = 10.2, 2.3, 1.2 Hz, 1H, $CH=C\underline{H_2}$), 3.43 (d, *J* = 5.8 Hz, 2H, $C\underline{H_2}OH$), 1.99 (dddd, *J* = 12.9, 6.4, 2.9, 1.4 Hz, 2H, $CH_2C\underline{H_2}CH$), 1.52 – 1.23 (m, 13H, $O\underline{H}$, $C\underline{H_2}CH_2CH$ and $C\underline{H_2}$ (cyclohexane)) ppm; **^{13}C NMR (101 MHz, $CDCl_3$)** δ 139.8 ($\underline{C}H=CH_2$), 114.1 ($CH=\underline{C}H_2$), 68.5 ($\underline{C}H_2OH$), 37.1 ($\underline{C}$), 34.1 ($\underline{C}H_2CH_2CH$), 32.6 (2C, $\underline{C}H_2$ (cyclohexane)), 27.7 ($CH_2\underline{C}H_2CH$), 26.5 ($\underline{C}H_2$ (cyclohexane)), 21.6 (2C, $\underline{C}H_2$ (cyclohexane)) ppm; **HRMS** (ESI) m/z calcd. for $C_{11}H_{21}O$ ($[M+H]^+$) 169.1587, found 169.1588.

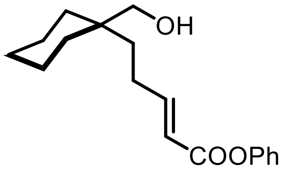

Phenyl (*E*)-5-(1-(hydroxymethyl)cyclohexyl)pent-2-enoate (3o) was prepared following **General Procedure B step iii**, using **(1-(But-3-en-1-yl)cyclohexyl)methanol (S21)** (335.5 mg, 2.0 mmol, 1.0 eq.) to afford the title compound as a colorless oil (492.2 mg, 86%). Silica gel chromatography condition: pentane/EtOAc = 4/1.

^{1}H NMR (400 MHz, $CDCl_3$) δ 7.43 – 7.33 (m, 2H, Ar-$\underline{H}$), 7.25 – 7.17 (m, 2H, Ar-$\underline{H}$ and $CH_2C\underline{H}=CH$), 7.14 – 7.06 (m, 2H, Ar-$\underline{H}$), 6.04 (dt, *J* = 15.7, 1.6 Hz, 1H, $CH_2CH=C\underline{H}$), 3.46 (s, 2H, $C\underline{H}_2OH$), 2.30 – 2.19 (m, 2H, $CH_2C\underline{H}_2CH$), 1.63 – 1.52 (m, 2H, $C\underline{H}_2CH_2CH$), 1.52 – 1.28 (m, 10H, $C\underline{H}_2$ (cyclohexane)) ppm; **^{13}C NMR (101 MHz, $CDCl_3$)** δ 165.2 ($\underline{C}$(O)O), 152.5 ($CH_2\underline{C}H=CH$), 150.9 (Ar$\underline{C}$), 129.5 (Ar$\underline{C}$H), 125.8 (Ar$\underline{C}$H), 121.8 (Ar$\underline{C}$H), 120.4 ($CH_2CH=\underline{C}H$), 68.3 ($\underline{C}H_2OH$), 37.2 (2C, $\underline{C}H_2$ (cyclohexane)), 33.2 ($\underline{C}H_2CH_2CH$), 32.6, 26.6 ($CH_2\underline{C}H_2CH$), 26.5 ($\underline{C}H_2$ (cyclohexane)), 21.6 (2C, $\underline{C}H_2$ (cyclohexane)) ppm; **HRMS** (ESI) m/z calcd. for $C_{18}H_{25}O_3$ ($[M+H]^+$) 289.1836, found 289.1834; **FT-IR (thin film)** ν_{max} 3399, 2926, 2853, 1734, 1650, 1593, 1492, 1454, 1247, 1197, 1163, 1148, 1024, 976, 928, 846, 751, 722, 688, 650, 627 cm^{-1}.

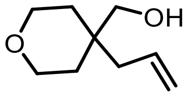

(4-Allyltetrahydro-2H-pyran-4-yl)methanol (S22) was prepared following **General Procedure B**. Data is consistent with the published literature.[21]

Step i.

Starting Materials: methyl tetrahydro-2H-pyran-4-carboxylate (1.45 g, 10.0 mmol, 1.0 eq.) and 3-bromoprop-1-ene (1.45 g, 12.0 mmol, 1.2 eq.).

Silica gel chromatography condition: pentane/Et_2O = 4/1.

Product: methyl 4-allyltetrahydro-2H-pyran-4-carboxylate: yellow oil (1.67 g, 98%).

Step ii.

Starting Materials: methyl 4-allyltetrahydro-2H-pyran-4-carboxylate (1.37 g, 8.1 mmol, 1.0 eq.)

Product: **(4-Allyltetrahydro-2H-pyran-4-yl)methanol (S22)**: pale-yellow oil (1.17 g, 94%).

^{1}H NMR (400 MHz, $CDCl_3$) δ 5.84 (ddt, *J* = 16.8, 10.3, 7.5 Hz, 1H, C<u>H</u>=CH_2), 5.36 – 4.89 (m, 2H, CH=C<u>H</u>$_2$), 3.67 (t, *J* = 5.5 Hz, 4H, OC<u>H</u>$_2$$CH_2$), 3.50 (s, 2H, C<u>H</u>$_2$OH), 2.22 (dt, *J* = 7.5, 1.2 Hz, 2H, C<u>H</u>$_2$CH), 1.60 (s, 1H, O<u>H</u>), 1.56 – 1.40 (m, 4H, OCH_2C<u>H</u>$_2$) ppm; **^{13}C NMR (101 MHz, $CDCl_3$)** δ 134.4 (<u>C</u>H=CH_2), 118.0 (CH=<u>C</u>H$_2$), 67.8 (<u>C</u>H$_2$OH), 63.7 (O<u>C</u>H$_2$$CH_2$), 39.7 (<u>C</u>H$_2$CH), 35.8 (<u>C</u>), 32.4 (OCH_2<u>C</u>H$_2$) ppm; **HRMS** (ESI) m/z calcd. for $C_9H_{17}O_2$ ($[M+H]^+$) 157.1223, found 157.1224.

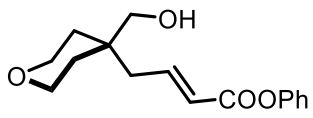

Phenyl (*E*)-4-(4-(hydroxymethyl)tetrahydro-2H-pyran-4-yl)but-2-enoate (3q) was prepared following **General Procedure B step iii**, using **(4-Allyltetrahydro-2H-pyran-4-yl)methanol (S22)** (320.8 mg, 2.05 mmol, 1.0 eq.) to afford the title compound as an off-white solid (214.6 mg, 38%). Silica gel chromatography condition: pentane/EtOAc = 3/7.

^{1}H NMR (400 MHz, $CDCl_3$) δ 7.43 – 7.34 (m, 2H, Ar-H), 7.26 – 7.14 (m, 2H, Ar-H and $CH_2C\underline{H}{=}CH$), 7.14 – 7.09 (m, 2H, Ar-H), 6.10 (dt, *J* = 15.5, 1.4 Hz, 1H, $CH_2CH{=}C\underline{H}$), 3.71 (t, *J* = 5.5 Hz, 4H, $OC\underline{H}_2CH_2$), 3.57 (d, *J* = 4.1 Hz, 2H, $C\underline{H}_2OH$), 2.47 (dd, *J* = 7.9, 1.4 Hz, 2H, $C\underline{H}_2CH$), 1.61 (t, *J* = 2.9 Hz, 1H, $O\underline{H}$), 1.59 – 1.47 (m, 4H, $OCH_2C\underline{H}_2$) ppm; **^{13}C NMR (101 MHz, $CDCl_3$)** δ 164.6 ($\underline{C}(O)O$), 150.8 (Ar$\underline{C}$), 147.4 ($CH_2\underline{C}H{=}CH$), 129.5 (Ar$\underline{C}$H), 125.9 (Ar$\underline{C}$H), 123.6 ($CH_2CH{=}\underline{C}H$), 121.7 (Ar$\underline{C}$H), 67.3 ($\underline{C}H_2OH$), 63.6 ($O\underline{C}H_2CH_2$), 38.0 ($\underline{C}H_2CH$)), 36.6 ($\underline{C}$), 32.5 ($OCH_2\underline{C}H_2$) ppm; **HRMS** (ESI) m/z calcd. for $C_{16}H_{20}O_4Na$ ($[M+Na]^+$) 299.1254, found 299.1253; **FT-IR (thin film)** ν_{max} 3434, 2980, 1732, 1650, 1592, 1492, 1391, 1315, 1240, 1196, 1163, 1135, 1106, 1043, 1025, 994, 897, 837, 779, 749, 689, 648, 628 cm^{-1}; **m.p.**: 57-58 °C.

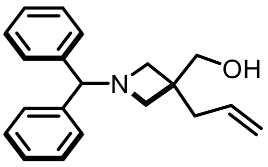

(3-Allyl-1-benzhydrylazetidin-3-yl)methanol (S23) was prepared following **General Procedure B**.

Step i.

Starting Materials: methyl 1-benzhydrylazetidine-3-carboxylate (2.82 g, 10.0 mmol, 1.0 eq.) and 3-bromoprop-1-ene (1.45 g, 12.0 mmol, 1.2 eq.).

Silica gel chromatography condition: pentane/Et_2O = 90/10.

Product: methyl 3-allyl-1-benzhydrylazetidine-3-carboxylate: colorless oil (2.14 g, 67%).

Step ii.

Starting Materials: methyl 3-allyl-1-benzhydrylazetidine-3-carboxylate (2.03 g, 6.3 mmol, 1.0 eq.)

Product: **(3-Allyl-1-benzhydrylazetidin-3-yl)methanol (S23)**: white solid (1.77 g, 96%).

^{1}H NMR (400 MHz, $CDCl_3$) δ 7.42 – 7.34 (m, 4H, Ar-H), 7.31 – 7.22 (m, 4H, Ar-H), 7.20 – 7.14 (m, 2H, Ar-H), 5.77 – 5.62 (m, 1H, CH=CH_2), 5.09 – 4.99 (m, 2H, CH=CH_2), 4.34 (s, 1H, NCH), 3.70 (s, 2H, CH_2OH), 3.60 – 3.36 (m, 1H, OH), 3.21 – 3.14 (m, 2H, NCH_2), 2.94 – 2.87 (m, 2H, NCH_2), 2.24 (dt, *J* = 7.3, 1.2 Hz, 2H, CH_2CH=CH_2) ppm; **^{13}C NMR (101 MHz, $CDCl_3$)** δ 142.0 (ArC), 134.0 (CH=CH_2), 128.6 (4C, ArCH), 127.5 (4C, ArCH), 127.3 (2C, ArCH), 117.7 (CH=CH_2), 77.6 (NCH), 67.8 (CH_2OH), 60.4 (2C, NCH_2), 40.1 (CH_2CH=CH_2), 39.2 (C); **HRMS** (ESI) m/z calcd. for $C_{20}H_{24}NO$ ([M+H]$^+$) 294.1850, found 294.1850; **FT-IR (thin film)** ν_{max} 3345, 3027, 2930, 2838, 1639, 1599, 1492, 1452, 1346, 1253, 1208, 1029, 996, 917, 800, 744, 703, 643, 623, 612 cm^{-1}; **m.p.:** 57-58 °C.

Preparation of starting materials 3p

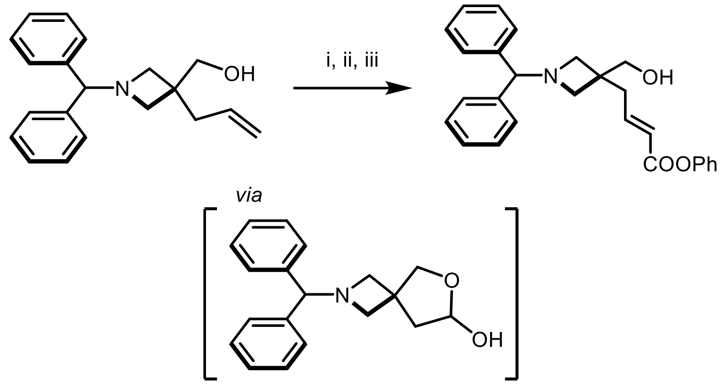

Scheme S7. Synthesis of starting materials **3p**. i. *N*-Methylmorpholine *N*-oxide (50% (w/w) in H_2O), OsO_4 (4% aqueous solution), ^{t}BuOH, THF, rt, 24 h; ii. $NaIO_4$, THF, H_2O, rt, 2 h. iii. Phenyl-(triphenylphosphoranylidene)acetate **S6**, toluene, rt, 48 h.

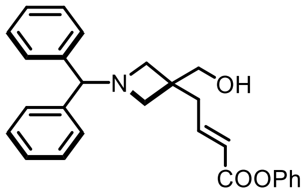

Phenyl (*E*)-4-(1-benzhydryl-3-(hydroxymethyl)azetidin-3-yl)but-2-enoate (3p) was prepared according to the following procedure.

i. *N*-Methylmorpholine *N*-oxide (50% (w/w) in H_2O, 5.6 mL, 1.2 eq.) and OsO_4 (4% aqueous solution, 0.53 mL, 0.002 eq.) were added to a solution of **(3-Allyl-1-benzhydrylazetidin-3-yl)methanol (S23)** (1.22 g, 4.15 mmol, 1.0 eq.) in ^{t}BuOH (28 mL) and THF (11 mL) under N_2 at room temperature. The reaction mixture was stirred at room temperature for 24 hours before cooling to 0 °C and quenching with an aqueous solution of $NaHSO_3$ (12 mL, 3M). The resulting mixture was warmed to room temperature and stirred for

45 mins. The aqueous layer was extracted with $CHCl_3$/IPA (3/1) (3 x 100 mL). The combined organic layers were washed with brine (100 mL), dried over Na_2SO_4, filtered and evaporated to dryness under reduced pressure to afford the diol which was used as crude for next step without any purification.

ii. $NaIO_4$ (2.31 g, 10.80 mmol, 2.6 eq.) was added to a solution of the diol in THF (55 mL) and H_2O (28 mL) under N_2 at room temperature. The reaction mixture was stirred for 2 hours before extracting with $CHCl_3$/IPA (3/1) (3 x 100 mL). The combined organic layers were washed with brine (100 mL), dried over Na_2SO_4, filtered and evaporated to dryness under reduced pressure. Purification by silica gel chromatography (pentane/EtOAc= 1/1) afforded the hemiacetal as a pale-yellow sticky oil (427.0 mg, 35%).

^{1}H NMR (400 MHz, $CDCl_3$) δ 7.43 – 7.37 (m, 4H, Ar-H), 7.30 – 7.22 (m, 4H, Ar-H), 7.22 – 7.13 (m, 2H, Ar-H), 5.48 (dd, *J* = 4.0, 2.6 Hz, 1H, OCH), 4.31 (s, 1H, NCH), 4.20 (d, *J* = 8.7 Hz, 1H, OCH_2), 3.98 (d, *J* = 8.7 Hz, 1H, OCH_2), 3.35 – 3.06 (m, 4H, CH_2NCH_2), 2.54 (s, 1H, OH), 2.18 – 2.12 (m, 2H, CH_2CH) ppm; **HRMS** (ESI) m/z calcd. for $C_{19}H_{22}NO_2$ ($[M+H]^+$) 296.1645, found 296.1643.

iii. hemiacetal (378.0 mg, 1.28 mmol, 1.0 eq.) was added to a solution of phenyl-(triphenylphosphoranylidene)acetate **S6** (760.6 mg, 1.92 mmol, 1.5 eq.) in toluene (6.4 mL) under N_2 at room temperature. The reaction mixture was stirred at room temperature for 48 hours before evaporating to dryness under reduced pressure. Purification by silica gel chromatography (pentane/EtOAc= 3/2) afforded the title compound as a sticky white foam (1:0.2 = ***E*** isomer : ***Z*** isomer) (344.5 mg, 65%).

^{1}H NMR (400 MHz, $CDCl_3$) δ 7.38 (ddt, *J* = 8.4, 7.2, 2.0 Hz, 7H, Ar-H (***E*** and ***Z***)), 7.31 – 7.21 (m, 6H, Ar-H (***E*** and ***Z***)), 7.21 – 7.14 (m, 3H, Ar-H (***E*** and ***Z***)), 7.14 – 7.06 (m, 3H, $CH_2CH{=}CH$ (***E***) and Ar-H (***E***

and ***Z***)), 6.37 (dt, *J* = 11.5, 8.2 Hz, 0.2H, $CH_2C\underline{H}$=CH (***Z***)), 6.13 (dt, *J* = 11.5, 1.5 Hz, 0.2H, $CH_2C\underline{H}$=CH (***Z***)), 6.06 (dt, *J* = 15.5, 1.5 Hz, 1H, $CH_2CH=C\underline{H}$ (***E***)), 4.44 – 4.25 (m, 1.2H, $NC\underline{H}$, (***E*** and ***Z***)), 3.78 – 3.62 (m, *J* = 4.1 Hz, 2.4H, $OC\underline{H}_2$ (***E*** and ***Z***)), 3.17 (d, *J* = 7.3 Hz, 2.6H, $C\underline{H}_2NCH_2$ (***E*** and ***Z***) and $O\underline{H}$ (***Z***)), 2.98 (t, *J* = 8.5 Hz, 3.4H, $CH_2NC\underline{H}_2$ (***E*** and ***Z***) and $O\underline{H}$ (***E***)), 2.56 – 2.49 (m, 2.4H, $C\underline{H}_2CH$ (***E*** and ***Z***)) ppm; **^{13}C NMR (101 MHz, $CDCl_3$)** δ 164.6 ($\underline{C}$(O)O), 150.8 (Ar$\underline{C}$), 146.9 ($CH_2\underline{C}H$=CH), 141.9 (Ar$\underline{C}$), 129.57 (Ar$\underline{C}$H), 129.55 (Ar$\underline{C}$H), 128.7 (Ar$\underline{C}$H), 128.6 (Ar$\underline{C}$H), 127.52 (Ar$\underline{C}$H), 127.46 (Ar$\underline{C}$H and Ar$\underline{C}$), 127.4 (Ar$\underline{C}$H), 125.9 (Ar$\underline{C}$H), 123.1 ($CH_2CH=\underline{C}H$), 121.7 (Ar$\underline{C}$H), 77.5 (N$\underline{C}$H), 67.5 ($O\underline{C}H_2$), 60.0 ($\underline{C}H_2N\underline{C}H_2$), 39.2 ($OHCH_2\underline{C}$), 38.2 ($\underline{C}H_2CH$) ppm. Only ^{13}C NMR for ***E*** isomer was observed; **HRMS** (ESI) m/z calcd. for $C_{27}H_{28}NO_3$ ($[M+H]^+$) 414.2064, found 414.2054; **FT-IR (thin film)** ν_{max} 3659, 2981, 2888, 1729, 1462, 1383, 1252, 1152, 1073, 955, 819, 704 cm^{-1}.

General procedure C for preparation of starting materials 3j, 3r to 3v and 3y to 3ab

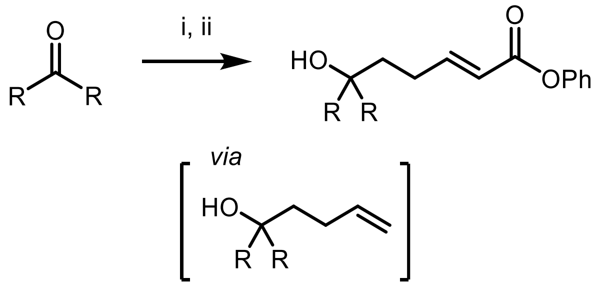

Scheme S8. Synthesis of starting materials **3j**, **3r** to **3v** and **3y** to **3ab**. i. but-3-en-1-ylmagnesium bromide or but-3-en-1-ylcerium(III) chloride, THF, 0 °C to rt, 16 h; ii. Hoveyda-Grubbs 2nd generation catalyst, phenyl acrylate, CH_2Cl_2, 45 °C, 3 h.

But-3-en-1-ylmagnesium bromide was prepared according to literature procedure and titrated before use.[22]

But-3-en-1-ylcerium(III) chloride was prepared according to the following procedure. $CeCl_3$ (1.0 eq.) (anhydrous grade, stored and weighed in a glove box) was stirred at 90 °C for 30 mins and 135 °C for 2 hours under reduced pressure before cooling down to 0 °C. Anhydrous THF (0.5M) was added to the reaction vessel under N_2 at 0 °C. The resulting slurry was warmed to room temperature and stirred for 24 hours under N_2 before cooling down to −78 °C. But-3-en-1-ylmagnesium bromide (1.0 eq.) was added dropwise under N_2 at −78 °C. The resulting mixture was stirred at −78 °C for 1 h, affording a THF solution of but-3-en-1-ylcerium(III) chloride which was used as crude for the addition without any purification.

i. Starting materials **3j**, **3r** to **3v** and **3y** to **3ab** was prepared according to the following procedure. A solution of ketone (1.0 eq.) in THF (0.1M) was added to but-3-en-1-ylmagnesium bromide or But-3-en-1-ylcerium(III) chloride (THF solution, 1.2 eq.) under N_2 at 0 °C. The reaction mixture was stirred at 0 °C for 1 hour before warming to room temperature and stirring for 15 hours. The mixture was then quenched

with a saturated aqueous solution of NH_4Cl (5 mL). The aqueous layer was extracted with Et_2O (3 x 20 mL). The combined organic layers were washed with brine (20 mL), dried over Na_2SO_4, filtered and carefully evaporated to dryness under reduced pressure. Purification by silica gel chromatography (pentane/EtOAc) afforded the terminal alkene.

ii. A solution of Hoveyda-Grubbs 2nd generation catalyst (0.5 mol%) in degassed CH_2Cl_2 (0.01M to the catalyst) was added dropwise (0.5 mL/h) by using a syringe pump to a mixture of the terminal alkene (1.0 eq.) and phenyl acrylate (3.0 eq.) under N_2 at 45 °C. The reaction mixture was stirred at 45 °C for 3 hours before directly loading onto the silica gel. Purification by silica gel chromatography (pentane/EtOAc) afforded starting materials **xx** to **xx**.

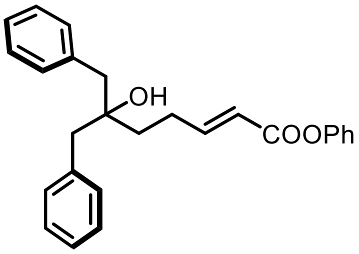

Phenyl (*E*)-6-benzyl-6-hydroxy-7-phenylhept-2-enoate (3j) was prepared following **General Procedure C**.

Step i.

<u>Starting Materials:</u> 1,3-diphenylpropan-2-one (3.06 g, 14.55 mmol, 1.0 eq.) and but-3-en-1-ylmagnesium bromide (17.46 mmol, 1.5 eq.).

<u>Silica gel chromatography condition</u>: pentane/CH_2Cl_2 = 3/2.

Product: **2-Benzyl-1-phenylhex-5-en-2-ol**: colorless oil (1.28 g, 33%).

Step ii.

Starting Materials: **2-Benzyl-1-phenylhex-5-en-2-ol** (567.0 mg, 2.13 mmol, 1.0 eq.)

Silica gel chromatography condition: pentane/EtOAc = 9/1.

Product: **Phenyl (*E*)-6-benzyl-6-hydroxy-7-phenylhept-2-enoate (3j)**: off-white solid (342.7 mg, 42%).

^{1}H NMR (400 MHz, $CDCl_3$) δ 7.41 – 7.17 (m, 13H, Ar-$\underline{H}$), 7.16 – 7.05 (m, 3H, Ar-$\underline{H}$ and $CH_2C\underline{H}=CH$), 5.98 (dt, *J* = 15.7, 1.6 Hz, 1H, $CH_2CH=C\underline{H}$), 2.83 (s, 4H, $ArC\underline{H_2}$), 2.51 – 2.40 (m, 2H, $CH_2C\underline{H_2}CH=CH$), 1.60 – 1.51 (m, 2H, $C\underline{H_2}CH_2CH=CH$), 1.44 (s, 1H, O$\underline{H}$) ppm; **^{13}C NMR (101 MHz, $CDCl_3$)** δ 165.0 ($\underline{C}$(O)O), 151.3 ($CH_2\underline{C}H=CH$), 150.9 (Ar$\underline{C}$), 136.9 (Ar$\underline{C}$), 130.8 (Ar$\underline{C}$H), 129.5 (Ar$\underline{C}$H), 128.6 (Ar$\underline{C}$H), 126.9 (Ar$\underline{C}$H), 125.8 (Ar$\underline{C}$H), 121.7 (Ar$\underline{C}$H), 120.8 ($CH_2CH=\underline{C}H$), 73.9 (OH$\underline{C}$), 45.8 ($Ar\underline{C}H_2$), 36.5 ($\underline{C}H_2CH_2CH=CH$), 27.1 ($CH_2\underline{C}H_2CH=CH$) ppm; **HRMS** (ESI) m/z calcd. for $C_{26}H_{26}ONa$ ($[M+Na]^+$) 409.1774, found 409.1774; **FT-IR (thin film)** ν_{max} 2981, 1731, 1650, 1593, 1493, 1454, 1323, 1250, 1196, 1163, 1146, 1110, 1031, 969, 753, 729, 702, 689 cm^{-1}; **m.p.**: 51-53 °C.

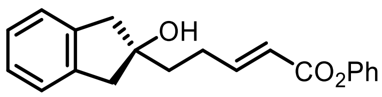

Phenyl (*E*)-5-(2-hydroxy-2,3-dihydro-1H-inden-2-yl)pent-2-enoate (3r) was prepared following **General Procedure C**.

Step i.

Starting Materials: 1,3-dihydro-2H-inden-2-one (330.4 mg, 2.5 mmol, 1.0 eq.) and but-3-en-1-ylcerium(III) chloride (3.0 mmol, 1.2 eq.).

Silica gel chromatography condition: pentane/Et_2O = 4/1.

Product: **2-(But-3-en-1-yl)-2,3-dihydro-1H-inden-2-ol**: orange oil (222.1 mg, 48%).

Step ii.

Starting Materials: **2-(But-3-en-1-yl)-2,3-dihydro-1H-inden-2-ol** (188.5 mg, 1.0 mmol, 1.0 eq.).

Silica gel chromatography condition: pentane/Et_2O = 3/2.

Product: **Phenyl (*E*)-5-(2-hydroxy-2,3-dihydro-1H-inden-2-yl)pent-2-enoate (3r)**: off-white solid (239.5 mg, 78%).

^{1}H NMR (400 MHz, $CDCl_3$) δ 7.52 – 7.05 (m, 10H, Ar-H and CH_2CH=CH), 6.09 (dt, *J* = 15.6, 1.6 Hz, 1H, CH_2CH=CH), 3.19 – 2.90 (m, 4H, $ArCH_2$), 2.66 – 2.44 (m, 2H, CH_2CH_2CH=CH), 2.07 – 1.90 (m, 2H, CH_2CH_2CH=CH), 1.75 (s, 1H, OH) ppm; **^{13}C NMR (101 MHz, $CDCl_3$)** δ 165.1 (C(O)O), 151.5 (CH_2CH=CH), 150.9 (ArC), 141.0 (ArC), 129.5 (ArCH), 127.0 (ArCH), 125.8 (ArCH), 125.4 (ArCH), 121.8 (ArCH), 120.9 (CH_2CH=CH), 82.2 (OC), 47.2 ($ArCH_2$), 38.8 (CH_2CH_2CH=CH), 28.0 (CH_2CH_2CH=CH) ppm; **HRMS** (ESI) m/z calcd. for $C_{20}H_{20}O_3Na$ ($[M+Na]^+$) 331.1305, found 331.1304; **FT-IR (thin film)** ν_{max} 3430, 2921, 1734, 1650, 1593, 1491, 1240, 1196, 1163, 1142, 1024, 984, 896, 741, 689, 625 cm^{-1}; **m.p.**: 58-60 °C.

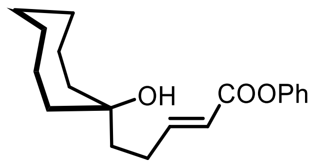

Phenyl (*E*)-5-(1-hydroxycyclooctyl)pent-2-enoate (3s) was prepared following **General Procedure C**.

Step i.

Starting Materials: cyclooctanone (406.8 mg, 3.23 mmol, 1.0 eq.) and but-3-en-1-ylcerium(III) chloride (3.88 mmol, 1.2 eq.).

Silica gel chromatography condition: pentane/Et_2O = 4/1.

Product: **1-(But-3-en-1-yl)cyclooctan-1-ol:** yellow oil (398.3 mg, 68%).

Step ii.

Starting Materials: **1-(But-3-en-1-yl)cyclooctan-1-ol** (273.3 mg, 1.5 mmol, 1.0 eq.).

Silica gel chromatography condition: pentane/Et_2O = 13/7.

Product: **Phenyl (*E*)-5-(1-hydroxycyclooctyl)pent-2-enoate (3s)**: pale-yellow oil (336.8 mg, 75%).

^{1}H NMR (400 MHz, $CDCl_3$) δ 7.43 – 7.33 (m, 2H, Ar-H), 7.28 – 7.16 (m, 2H, Ar-H and CH_2CH=CH), 7.15 – 7.07 (m, 2H, Ar-H), 6.05 (dt, *J* = 15.6, 1.6 Hz, 1H, CH_2CH=CH), 2.47 – 2.34 (m, 2H, CH_2CH_2CH=CH), 1.87 – 1.75 (m, 2H, CH_2 (cyclooctane)), 1.74 – 1.48 (m, 11H, CH_2CH_2CH=CH and CH_2 (cyclooctane)), 1.47 – 1.37 (m, 3H, CH_2 (cyclooctane)), 1.27 (s, 1H, OH) ppm; **^{13}C NMR (101 MHz, $CDCl_3$)** δ 165.1 (C(O)O), 152.3 (CH_2CH=CH), 150.9 (ArC), 129.5 (ArCH), 125.8 (ArCH) , 121.8 (ArCH), 120.5 (CH_2CH=CH), 74.7 (OHC), 39.5 (CH_2CH_2CH=CH), 36.5 (CH_2 (cyclooctane)), 28.3 (CH_2 (cyclooctane)), 26.6 (CH_2CH_2CH=CH), 25.2 (CH_2 (cyclooctane)), 22.5 (CH_2 (cyclooctane)) ppm; **HRMS** (ESI) m/z calcd. for $C_{19}H_{26}O_3Na$ ([M+Na]$^+$) 325.1774, found 325.1775; **FT-IR (thin film)** ν_{max} 3439, 2921,

2853, 1735, 1650, 1593, 1492, 1448, 1321, 1248, 1196, 1162, 1146, 1023, 978, 900, 847, 751, 721, 688, 645, 619 cm^{-1}.

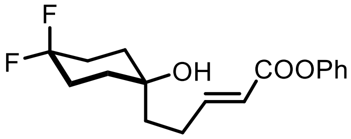

Phenyl (*E*)-5-(4,4-difluoro-1-hydroxycyclohexyl)pent-2-enoate (3t) was prepared following **General Procedure C**.

Step i.

Starting Materials: 4,4-difluorocyclohexan-1-one (335.3 mg, 2.5 mmol, 1.0 eq.) and but-3-en-1-ylcerium(III) chloride (3.0 mmol, 1.2 eq.).

Silica gel chromatography condition: pentane/Et_2O = 17/3.

Product: **1-(But-3-en-1-yl)-4,4-difluorocyclohexan-1-ol**: colorless oil (303.6 mg, 64%).

Step ii.

Starting Materials: **1-(But-3-en-1-yl)-4,4-difluorocyclohexan-1-ol** (247.2 mg, 1.3 mmol, 1.0 eq.).

Silica gel chromatography condition: pentane/Et_2O = 3/2.

Product: **Phenyl (*E*)-5-(4,4-difluoro-1-hydroxycyclohexyl)pent-2-enoate (3t)**: off-white solid (354.8 mg, 88%).

^{1}H NMR (400 MHz, $CDCl_3$) δ 7.44 – 7.34 (m, 2H, Ar-H), 7.26 – 7.14 (m, 2H, Ar-H and $CH_2C\underline{H}$=CH), 7.14 – 7.07 (m, 2H, Ar-H), 6.05 (dt, *J* = 15.6, 1.6 Hz, 1H, $CH_2CH=C\underline{H}$), 2.45 – 2.35 (m, 2H, $CH_2C\underline{H}_2CH=CH$), 2.22 – 2.01 (m, 2H, $CH_2CH_2CF_2C\underline{H}_2CH_2$), 1.94 (ddddd, *J* = 13.2, 10.8, 7.4, 5.3, 2.9 Hz,

2H, $CH_2C\underline{H}_2CF_2CH_2CH_2$), 1.79 – 1.60 (m, 6H, $C\underline{H}_2CH_2CH{=}CH$ and $C\underline{H}_2CH_2CF_2CH_2C\underline{H}_2$), 1.28 – 1.20 (m, 1H, $O\underline{H}$) ppm; **^{13}C NMR (101 MHz, $CDCl_3$)** δ 165.0 ($\underline{C}(O)O$), 151.2 ($CH_2\underline{C}H{=}CH$), 150.8 ($Ar\underline{C}$), 129.5 ($Ar\underline{C}H$), 125.9 ($Ar\underline{C}H$), 125.8 – 121.2 (m, $\underline{C}F_2$), 121.7 ($Ar\underline{C}H$), 121.0 ($CH_2CH{=}\underline{C}H$), 69.9 (d, *J* = 1.6 Hz, $OH\underline{C}$), 40.9 ($\underline{C}H_2CH_2CH{=}CH$), 33.6 (d, *J* = 9.1 Hz, $\underline{C}H_2CH_2CF_2CH_2\underline{C}H_2$), 30.3 – 29.2 (m, $CH_2\underline{C}H_2CF_2\underline{C}H_2CH_2$), 26.5 ($CH_2\underline{C}H_2CH{=}CH$) ppm; **^{19}F NMR (376 MHz, $CDCl_3$)** δ −92.86 (d, *J* = 235.6 Hz), −104.16 (dt, *J* = 237.2, 33.6 Hz) ppm; **HRMS** (ESI) m/z calcd. for $C_{17}H_{20}O_3F_2Na$ ($[M+Na]^+$) 333.1273, found 333.1274; **FT-IR (thin film)** ν_{max} 3371, 2943, 1734, 1718, 1655, 1593, 1494, 1361, 1326, 1287, 1250, 1197, 1168, 1148, 1115, 1023, 969, 853, 820, 767, 688, 645 cm^{-1}; **m.p.**: 112-114 °C.

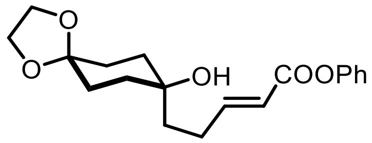

Phenyl (*E*)-5-(8-hydroxy-1,4-dioxaspiro[4.5]decan-8-yl)pent-2-enoate (3u) was prepared following **General Procedure C**.

Step i.

Starting Materials: 1,4-dioxaspiro[4.5]decan-8-one (468.6 mg, 3.0 mmol, 1.0 eq.) and but-3-en-1-ylmagnesium bromide (3.6 mmol, 1.2 eq.).

Silica gel chromatography condition: pentane/EtOAc = 7/3.

Product: **8-(But-3-en-1-yl)-1,4-dioxaspiro[4.5]decan-8-ol**: colorless oil (177.2 mg, 28%).

Step ii.

Starting Materials: **8-(But-3-en-1-yl)-1,4-dioxaspiro[4.5]decan-8-ol** (160.6 mg, 0.76 mmol, 1.0 eq.)

Silica gel chromatography condition: pentane/EtOAc = 1/1.

Product: **Phenyl (*E*)-5-(8-hydroxy-1,4-dioxaspiro[4.5]decan-8-yl)pent-2-enoate (3u)**: off-white solid (197.1 mg, 78%).

^{1}H NMR (400 MHz, CDCl$_3$) δ 7.43 – 7.33 (m, 2H, Ar-H), 7.25 – 7.15 (m, 2H, Ar-H and $CH_2CH=CH$), 7.14 – 7.07 (m, 2H, Ar-H), 6.04 (dt, *J* = 15.6, 1.6 Hz, 1H, $CH_2CH=CH$), 4.02 – 3.87 (m, 4H, OCH_2CH_2O), 2.46 – 2.36 (m, 2H, $CH_2CH_2CH=CH$), 1.89 (ddd, *J* = 12.7, 9.4, 7.0 Hz, 2H, $CCH_2CH_2CCH_2CH_2$), 1.73 – 1.57 (m, 8H, $CH_2CH_2CH=CH$ and $CCH_2CH_2CCH_2CH_2$), 1.36 (s, 1H, OH) ppm; **^{13}C NMR (101 MHz, CDCl$_3$)** δ 165.1 (C(O)O), 151.8 ($CH_2CH=CH$), 150.9 (ArC), 129.5 (ArCH), 125.8 (ArCH), 121.8 (ArCH), 120.7 ($CH_2CH=CH$), 108.7 (OCO), 70.4 (OHC), 64.5 (OCH_2CH_2O), 64.4 (OCH_2CH_2O), 40.7 ($CH_2CH_2CH=CH$), 34.8 ($CCH_2CH_2CCH_2CH_2$), 30.6 ($CCH_2CH_2CCH_2CH_2$), 26.7 ($CH_2CH_2CH=CH$) ppm; **HRMS** (ESI) m/z calcd. for $C_{19}H_{24}O_5Na$ ([M+Na]$^+$) 355.1516, found 355.1514; **FT-IR (thin film)** ν_{max} 3746, 3474, 2940, 2439, 1738, 1650, 1594, 1493, 1439, 1254, 1197, 1165, 1145, 1110, 1036, 931, 825, 745, 718, 690, 676 cm^{-1}; **m.p.**: 36-37 °C.

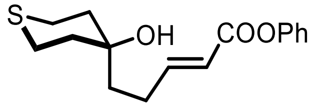

Phenyl (*E*)-5-(4-hydroxytetrahydro-2H-thiopyran-4-yl)pent-2-enoate (3v) was prepared following **General Procedure C**.

Step i.

Starting Materials: tetrahydro-4H-thiopyran-4-one (1.49 g, 12.8 mmol, 1.0 eq.) and but-3-en-1-ylmagnesium bromide (15.4 mmol, 1.2 eq.).

Silica gel chromatography condition: pentane/EtOAc = 4/1.

Product: **4-(But-3-en-1-yl)tetrahydro-2H-thiopyran-4-ol**: orange oil (383.7 mg, 18%).

Step ii.

Starting Materials: **4-(But-3-en-1-yl)tetrahydro-2H-thiopyran-4-ol** (138.4 mg, 0.8 mmol, 1.0 eq.).

Silica gel chromatography condition: pentane/EtOAc = 4/1.

Product: **Phenyl (*E*)-5-(4-hydroxytetrahydro-2H-thiopyran-4-yl)pent-2-enoate (3v)**: off-white solid (220 mg, 95%).

^{1}H NMR (400 MHz, $CDCl_3$) δ 7.44 – 7.33 (m, 2H, Ar-H), 7.25 – 7.14 (m, 2H, Ar-H and CH_2CH=CH), 7.14 – 7.07 (m, 2H, Ar-H), 6.05 (dt, *J* = 15.7, 1.7 Hz, 1H, CH_2CH=CH), 2.96 (brs, 2H, CH_2), 2.51 – 2.34 (m, 4H, CH_2), 1.89 (dt, *J* = 14.0, 3.9 Hz, 2H, CH_2), 1.78 (ddd, *J* = 14.0, 11.3, 3.6 Hz, 2H, CH_2), 1.69 – 1.60 (m, 2H, CH_2), 1.50 – 1.13 (m, 1H, OH) ppm; **^{13}C NMR (101 MHz, $CDCl_3$)** δ 165.0 (C(O)O), 151.4 (CH_2CH=CH), 150.8 (ArC), 129.5 (ArCH), 125.9 (ArCH), 121.7 (ArCH), 120.9 (CH_2CH=CH), 69.8 (OC), 41.6 (CH_2), 38.3 (CH_2), 25.9 (CH_2), 24.3 (CH_2) ppm; **FT-IR (thin film)** ν_{max} 3474, 2931, 2360, 2341, 1728, 1650, 1593, 1492, 1426, 1320, 1246, 1196, 1163, 1145, 1024, 977, 921, 817, 730, 689, 659, 615 cm^{-1}; **m.p.**: 88-89 °C

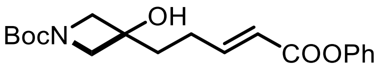

***tert*-Butyl (*E*)-3-hydroxy-3-(5-oxo-5-phenoxypent-3-en-1-yl)azetidine-1-carboxylate (3y)** was prepared following **General Procedure C**.

Step i.

Starting Materials: *tert*-butyl 3-oxoazetidine-1-carboxylate (1.16 g, 6.75 mmol, 1.0 eq.) and but-3-en-1-ylmagnesium bromide (8.10 mmol, 1.2 eq.).

Silica gel chromatography condition: pentane/Et_2O = 2/3.

Product: ***tert*-Butyl 3-(but-3-en-1-yl)-3-hydroxyazetidine-1-carboxylate**: white solid (878.2 mg, 57%).

Step ii.

Starting Materials: ***tert*-Butyl 3-(but-3-en-1-yl)-3-hydroxyazetidine-1-carboxylate** (454.3 mg, 2.0 mmol, 1.0 eq.). 5% Hoveyda-Grubbs 2nd generation catalyst was used.

Silica gel chromatography condition: pentane/Et_2O = 3/7.

Product: ***tert*-Butyl (*E*)-3-hydroxy-3-(5-oxo-5-phenoxypent-3-en-1-yl)azetidine-1-carboxylate (3y)**: off-white solid (663.9 mg, 96%).

^{1}H NMR (400 MHz, $CDCl_3$) δ 7.43 – 7.34 (m, 2H, Ar-H), 7.25 – 7.15 (m, 2H, Ar-H and CH_2CH=CH), 7.14 – 7.08 (m, 2H, Ar-H,), 6.06 (dt, *J* = 15.6, 1.6 Hz, 1H, CH_2CH=CH), 3.92 – 3.75 (m, 4H, CH_2NCH_2), 2.73 – 2.30 (m, 3H, CH_2CH_2CH=CH and OH), 2.00 – 1.90 (m, 2H, CH_2CH_2CH=CH), 1.45 (s, 9H, $C(CH_3)_3$) ppm; **^{13}C NMR (101 MHz, $CDCl_3$)** δ 164.9 (C(O)O), 156.6 (C(O)N), 150.8 (ArC), 150.5 (CH_2CH=CH), 129.5 (ArCH), 125.9 (ArCH) , 121.7 (ArCH), 121.3 (CH_2CH=CH), 80.1 ($C(CH_3)_3$), 70.3 (OHC), 62.3 (CH_2NCH_2), 37.1 (CH_2CH_2CH=CH), 28.5 ($C(CH_3)_3$), 26.7 (CH_2CH_2CH=CH) ppm; **HRMS** (ESI) m/z calcd. for $C_{19}H_{25}O_5Na$ ($[M+Na]^+$) 370.1625, found 370.1627; **FT-IR (thin film)** ν_{max} 3385, 1737, 1676, 1593, 1493, 1426, 1367, 1316, 1247, 1197, 1163, 1104, 984, 929, 855, 773, 689, 643, 610 cm^{-1}; **m.p.**: 88-89 °C.

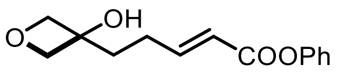

Phenyl (*E*)-5-(3-hydroxyoxetan-3-yl)pent-2-enoate (3z) was prepared following **General Procedure C**.

Step i.

Starting Materials: cyclobutanone (216.2 mg, 3.0 mmol, 1.0 eq.) and but-3-en-1-ylmagnesium bromide (3.6 mmol, 1.2 eq.).

Silica gel chromatography condition: pentane/EtOAc = 3/2.

Product: **3-(But-3-en-1-yl)oxetan-3-ol**: colorless oil (309.7 mg, 81%).

Step ii.

Starting Materials: **3-(But-3-en-1-yl)oxetan-3-ol** (250.6 mg, 1.96 mmol, 1.0 eq.)

Silica gel chromatography condition: pentane/EtOAc = 3/2.

Product: **Phenyl (*E*)-5-(3-hydroxyoxetan-3-yl)pent-2-enoate (3z)**: off-white solid (306.2 mg, 63%).

^{1}H NMR (400 MHz, $CDCl_3$) δ 7.44 – 7.34 (m, 2H, Ar-H), 7.29 – 7.16 (m, 2H, Ar-H and CH_2CH=CH), 7.15 – 7.07 (m, 2H, Ar-H), 6.08 (dt, *J* = 15.6, 1.6 Hz, 1H, CH_2CH=CH), 4.63 – 4.52 (m, 4H, CH_2OCH_2), 2.50 – 2.34 (m, 3H, CH_2CH_2CH=CH and OH), 2.12 – 2.02 (m, 2H, CH_2CH_2CH=CH) ppm; **^{13}C NMR (101 MHz, $CDCl_3$)** δ 164.9 (C(O)O), 150.8 (ArC), 150.5 (CH_2CH=CH), 129.6 (ArCH), 125.9 (ArCH), 121.7 (ArCH), 121.4 (CH_2CH=CH), 84.0 (CH_2OCH_2), 74.5 (OHC), 36.0 (CH_2CH_2CH=CH), 26.6 (CH_2CH_2CH=CH) ppm; **HRMS** (ESI) m/z calcd. for $C_{14}H_{16}O_4Na$ ($[M+Na]^+$) 271.0941, found 271.0940; **FT-IR (thin film)** ν_{max} 3660, 2981, 2888, 1735, 1462, 1382, 1252, 1152, 1073, 956, 818, 652 cm^{-1}; **m.p.**: 36-37 °C

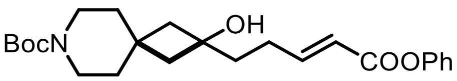

***tert*-Butyl (*E*)-2-hydroxy-2-(5-oxo-5-phenoxypent-3-en-1-yl)-7-azaspiro[3.5]nonane-7-carboxylate (3aa)** was prepared following **General Procedure C**.

Step i.

Starting Materials: *tert*-butyl 2-oxo-7-azaspiro[3.5]nonane-7-carboxylate (486.0 mg, 2.03 mmol, 1.0 eq.) and but-3-en-1-ylmagnesium bromide (2.44 mmol, 1.2 eq.).

Silica gel chromatography condition: pentane/Et_2O = 1/4.

Product: ***tert*-Butyl 2-(but-3-en-1-yl)-2-hydroxy-7-azaspiro[3.5]nonane-7-carboxylate**: orange oil (577.2 mg, 97%).

Step ii.

Starting Materials: ***tert*-Butyl 2-(but-3-en-1-yl)-2-hydroxy-7-azaspiro[3.5]nonane-7-carboxylate** (320.4 mg, 1.09 mmol, 1.0 eq.). 6% Hoveyda-Grubbs 2nd generation catalyst was used.

Silica gel chromatography condition: pentane/EtOAc = 3/2.

Product: ***tert*-Butyl (*E*)-2-hydroxy-2-(5-oxo-5-phenoxypent-3-en-1-yl)-7-azaspiro[3.5]nonane-7-carboxylate (3aa)**: off-white solid (240.6 mg, 53%).

^{1}H NMR (400 MHz, $CDCl_3$) δ 7.42 – 7.34 (m, 2H, Ar-H), 7.25 – 7.17 (m, 2H, Ar-H and $CH_2C\underline{H}=CH$), 7.14 – 7.07 (m, 2H, Ar-H), 6.05 (dt, J = 15.6, 1.6 Hz, 1H, $CH_2CH=C\underline{H}$), 3.37 – 3.24 (m, 4H, $C\underline{H_2}NC\underline{H_2}$), 2.38 (dddd, J = 10.1, 8.4, 6.5, 1.6 Hz, 2H, $CH_2C\underline{H_2}CH=CH$), 2.01 – 1.93 (m, 2H, $CC\underline{H_2}CCH_2$), 1.91 – 1.84 (m, 2H, $CCH_2CC\underline{H_2}$), 1.84 – 1.74 (m, 2H, $C\underline{H_2}CH_2CH=CH$), 1.70 – 1.63 (m, 2H, $C\underline{H_2}CH_2NCH_2CH_2$), 1.60 (brs, 1H, O$\underline{H}$), 1.52 – 1.46 (m, 2H, $CH_2CH_2NCH_2C\underline{H_2}$), 1.45 (s, 9H, $C(C\underline{H_3})_3$) ppm; **^{13}C NMR (101 MHz,**

CDCl$_3$) δ 165.0 (C(O)O), 155.1 (C(O)N), 151.4 (CH$_2$CH=CH), 150.9 (ArC), 129.5 (ArCH), 125.9 (ArCH), 121.7 (ArCH), 120.9 (CH$_2$CH=CH), 79.5 (C(CH$_3$)$_3$), 71.1 (OHC), 45.7 (CCH$_2$CCH$_2$), 41.8 (CH$_2$CH$_2$CH=CH), 40.9 (CH$_2$NCH$_2$), 40.7 (CH$_2$NCH$_2$), 38.9 (CH$_2$CH$_2$NCH$_2$CH$_2$), 38.0 (CH$_2$CH$_2$NCH$_2$CH$_2$), 29.5 (NCH$_2$C), 28.6 (C(CH$_3$)$_3$), 26.7 (CH$_2$CH$_2$CH=CH) ppm; **HRMS** (ESI) m/z calcd. for $C_{24}H_{33}O_5NNa$ ([M+Na]$^+$) 438.2251, found 438.2249; **FT-IR (thin film)** ν_{max} 3438, 2919, 2845, 1737, 1691, 1667, 1594, 1493, 1426, 1366, 1270, 1245, 1197, 1148, 970, 924, 863, 772, 732, 689, 661, 625 cm^{-1}; **m.p.**: 69-70 °C.

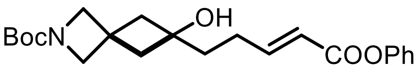

***tert*-Butyl (*E*)-6-hydroxy-6-(5-oxo-5-phenoxypent-3-en-1-yl)-2-azaspiro[3.3]heptane-2-carboxylate (3ab)** was prepared following **General Procedure C**.

Step i.

Starting Materials: *tert*-butyl 6-oxo-2-azaspiro[3.3]heptane-2-carboxylate (508.0 mg, 2.4 mmol, 1.0 eq.) and but-3-en-1-ylmagnesium bromide (2.9 mmol, 1.2 eq.).

Silica gel chromatography condition: pentane/Et$_2$O = 1/4.

Product: ***tert*-Butyl 6-(but-3-en-1-yl)-6-hydroxy-2-azaspiro[3.3]heptane-2-carboxylate**: orange oil (154.0 mg, 24%).

Step ii.

Starting Materials: ***tert*-Butyl 6-(but-3-en-1-yl)-6-hydroxy-2-azaspiro[3.3]heptane-2-carboxylate** (286.6 mg, 1.07 mmol, 1.0 eq.). 3% Hoveyda-Grubbs 2nd generation catalyst was used.

Silica gel chromatography condition: pentane/Et_2O = 1/4.

Product: ***tert*-Butyl (*E*)-6-hydroxy-6-(5-oxo-5-phenoxypent-3-en-1-yl)-2-azaspiro[3.3]heptane-2-carboxylate (3ab)**: off-white solid (255.8 mg, 62%).

^{1}H NMR (400 MHz, $CDCl_3$) δ 7.43 – 7.33 (m, 2H, Ar-H), 7.25 – 7.15 (m, 2H, Ar-H and CH_2CH=CH), 7.14 – 7.07 (m, 2H, Ar-H), 6.05 (dt, *J* = 15.6, 1.6 Hz, 1H, CH_2CH=CH), 3.91 (d, *J* = 13.7 Hz, 4H, CH_2NCH_2), 2.46 – 2.29 (m, 4H, CCH_2CCH_2 and CH_2CH_2CH=CH), 2.27 – 2.17 (m, 2H, CCH_2CCH_2), 1.76 (brs, 1H, OH), 1.73 – 1.66 (m, 2H, CH_2CH_2CH=CH), 1.43 (s, 9H, $C(CH_3)_3$) pp; **^{13}C NMR (101 MHz, $CDCl_3$)** δ 165.0 (C(O)O), 156.2 (C(O)N), 151.1 (CH_2CH=CH), 150.9 (ArC), 129.5 (ArCH) , 125.9 (ArCH), 121.7 (ArCH), 121.0 (CH_2CH=CH), 79.6 ($C(CH_3)_3$), 71.0 (OHC), 61.9 (CH_2NCH_2), 61.2 (CH_2NCH_2), 47.0 (CCH_2CCH_2), 39.0 (CH_2CH_2CH=CH), 29.5 (NCH_2C), 28.5 ($C(CH_3)_3$), 26.9 (CH_2CH_2CH=CH) ppm; **HRMS** (ESI) m/z calcd. for $C_{22}H_{29}O_5NNa$ ($[M+Na]^+$) 410.1938, found 410.1941; **FT-IR (thin film)** ν_{max} 3390, 2923, 2359, 1737, 1700, 1676, 1594, 1493, 1417, 1366, 1317, 1247, 1197, 1164, 1026, 985, 926, 856, 772, 689, 626, 610 cm^{-1}; **m.p.**: 120-121 °C

Preparation of starting materials 3w

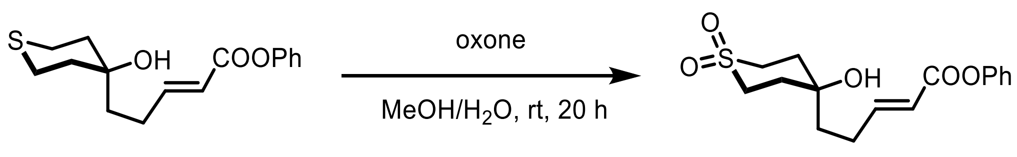

Phenyl (*E*)-5-(4-hydroxy-1,1-dioxidotetrahydro-2H-thiopyran-4-yl)pent-2-enoate (3w) was prepared according to the following procedure. OXONE® (264.4 mg, 0.86 mmol, 2.0 eq.) was added to a solution of **Phenyl (*E*)-5-(4-hydroxytetrahydro-2H-thiopyran-4-yl)pent-2-enoate (3v)** (125.4 mg, 0.43 mmol,

1.0 eq.) in MeOH (1.1 mL) and H_2O (1.1 mL) at room temperature. The resulting mixture was stirred at room temperature for 20 hours before quenching with a solution of Na_2SO_3 (108.4 mg, 0.86 mmol, 2.0 eq.) in H_2O (5 mL). The mixture was stirred at room temperature for 30 mins. The aqueous layer was extracted with CH_2Cl_2 (3 x 50 mL). The combined organic layers were washed with brine (50 mL), dried over Na_2SO_4, filtered and evaporated to dryness under reduced pressure. Purification by silica gel chromatography (pentane/EtOAc = 1/1) afforded the title compound as a white solid (97.3 mg, 70%).

^{1}H NMR (400 MHz, $CDCl_3$) δ 7.44 – 7.34 (m, 2H, Ar-$\underline{H}$), 7.26 – 7.21 (m, 1H, Ar-$\underline{H}$), 7.16 (dd, *J* = 15.6, 6.8 Hz, 1H, $CH_2C\underline{H}=CH$), 7.13 – 7.06 (m, 2H, Ar-$\underline{H}$), 6.07 (dt, *J* = 15.6, 1.6 Hz, 1H, $CH_2CH=C\underline{H}$), 3.40 (td, *J* = 13.8, 3.7 Hz, 2H, $CH_2C\underline{H_2}SC\underline{H_2}CH_2$), 2.92 – 2.81 (m, 2H, $CH_2C\underline{H_2}SC\underline{H_2}CH_2$), 2.46 – 2.35 (m, 2H, $CH_2C\underline{H_2}CH=CH$), 2.21 (td, *J* = 14.2, 3.7 Hz, 2H, $C\underline{H_2}CH_2SCH_2C\underline{H_2}$), 2.07 – 1.96 (m, 2H, $C\underline{H_2}CH_2SCH_2C\underline{H_2}$), 1.79 – 1.70 (m, 2H, $C\underline{H_2}CH_2CH=CH$), 1.54 (s, 1H, O$\underline{H}$) ppm; **^{13}C NMR (101 MHz, $CDCl_3$)** δ 164.9 ($\underline{C}$(O)O), 150.7 (Ar$\underline{C}$), 150.1 ($CH_2\underline{C}H=CH$), 129.6 (Ar$\underline{C}$H), 126.0 (Ar$\underline{C}$H), 121.7 (Ar$\underline{C}$H), 121.5 ($CH_2CH=\underline{C}H$), 68.5 (OH$\underline{C}$), 46.9 ($CH_2\underline{C}H_2S\underline{C}H_2CH_2$), 41.0 ($\underline{C}H_2CH_2CH=CH$), 35.0 ($\underline{C}H_2CH_2SCH_2\underline{C}H_2$), 26.4 ($CH_2\underline{C}H_2CH=CH$) ppm; **HRMS** (ESI) m/z calcd. for $C_{16}H_{20}O_5SNa$ ($[M+Na]^+$) 347.0924, found 347.0922; **FT-IR (thin film)** ν_{max} 3659, 2981, 2888, 1462, 1383, 1252, 1151, 1073, 955, 818 cm^{-1}; **m.p.**: 140-141 °C.

Preparation of starting materials 3x

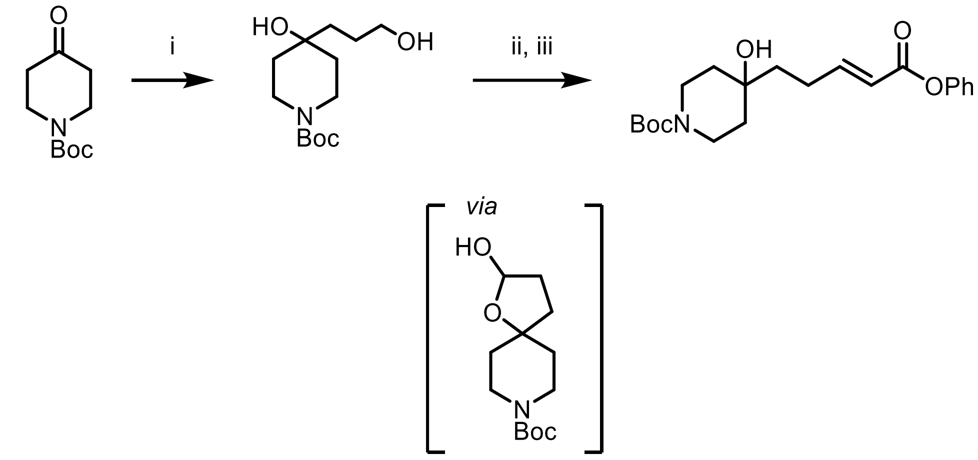

Scheme S9. Synthesis of starting materials **3x**. i. Grignard reagent **S24**, THF, 0 °C to rt, 24 h; ii. DMP, CH_2Cl_2, 0 °C to rt, 4 h; iii. Phenyl-(triphenylphosphoranylidene)acetate **S6**, toluene, rt, 48 h.

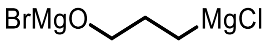

Grignard reagent **S24** was prepared according to literature procedure.[23]

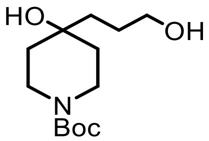

***tert*-Butyl 4-hydroxy-4-(3-hydroxypropyl)piperidine-1-carboxylate (S25)** was prepared according to the following procedure. **Grignard reagent S24** (0.285M in THF) (21 mL, 6.0 mmol, 1.2 eq.) was added dropwise to a solution of *tert*-butyl 4-oxopiperidine-1-carboxylate (995.6 mg, 5.0 mmol, 1.0 eq.) in THF (5 mL) under N_2 at 0 °C. The resulting mixture was warmed to room temperature and stirred for 24 hours

before quenching with a saturated aqueous solution of NH_4Cl (20 mL) and diluting with H_2O (20 mL). The aqueous layer was extracted with $CHCl_3$/IPA (3/1) (3 x 100 mL). The combined organic layers were washed with brine (100 mL), dried over Na_2SO_4, filtered and evaporated to dryness under reduced pressure. Purification by silica gel chromatography (CH_2Cl_2/MeOH = 95/5) afforded the title compound as a pale-yellow oil (746.9 mg, 58%). Data is consistent with the published literature.[24]

^{1}H NMR (400 MHz, $CDCl_3$) δ 3.93 – 3.71 (m, 2H, C<u>H</u>$_2$), 3.66 (dt, *J* = 17.4, 6.1 Hz, 2H, C<u>H</u>$_2$), 3.22 – 3.11 (m, 2H, C<u>H</u>$_2$), 2.39 (brs, 2H, O<u>H</u>), 1.77 – 1.64 (m, 2H, C<u>H</u>$_2$), 1.63 – 1.48 (m, 6H, C<u>H</u>$_2$), 1.45 (s, 9H, C(C<u>H</u>$_3$)$_3$) ppm; **^{13}C NMR (101 MHz, $CDCl_3$)** δ 155.0 (<u>C</u>(O)N), 79.5 (<u>C</u>(CH$_3$)$_3$), 69.2 (OH<u>C</u>), 63.4 (<u>C</u>H$_2$), 63.0 (<u>C</u>H$_2$), 40.2 (<u>C</u>H$_2$), 37.0 (<u>C</u>H$_2$), 32.8 (<u>C</u>H$_2$), 28.6 (C(<u>C</u>H$_3$)$_3$), 26.0 (<u>C</u>H$_2$), 25.7 (<u>C</u>H$_2$) ppm; **HRMS** (ESI) m/z calcd. for $C_{13}H_{25}O_4NNa$ ([M+Na]$^+$) 282.1676, found 282.1674.

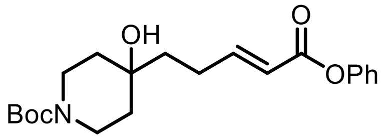

***tert*-Butyl (*E*)-4-hydroxy-4-(5-oxo-5-phenoxypent-3-en-1-yl)piperidine-1-carboxylate (3x)** was prepared according to the following procedure. DMP (1.37 g, 3.22 mmol,1.1 eq.) was added to a solution of ***tert*-Butyl 4-hydroxy-4-(3-hydroxypropyl)piperidine-1-carboxylate (S25)** (756.5 mg, 2.92 mmol, 1.0 eq.) in CH_2Cl_2 (42 mL) under N_2 at 0 °C. The resulting mixture was stirred at 0 °C for 2 hours then room temperature for 2 hours before quenching with an aqueous solution of NaOH (1M, 20 mL). The aqueous layer was extracted with Et_2O (3 x 100 mL). The combined organic layers were washed with brine (100

mL), dried over Na_2SO_4, filtered and evaporated to dryness under reduced pressure. Purification by silica gel chromatography (CH_2Cl_2/MeOH = 95/5) afforded the hemiacetal as a pale-yellow oil (449.4 mg, 60%).

A solution of hemiacetal (407.2 mg, 1.59 mmol, 1.0 eq.) in toluene (2 mL) was added to a solution of **phenyl-(triphenylphosphoranylidene)acetate S6** (757.2 mg, 1.91 mmol, 1.2 eq.) in toluene (6.4 mL) under N_2 at room temperature. The reaction mixture was stirred at room temperature for 48 hours before evaporating to dryness under reduced pressure. Purification by silica gel chromatography (pentane/Et_2O = 3/7) afforded the title compound as a white solid (132.1 mg, 22%).

^{1}H NMR (400 MHz, $CDCl_3$) δ 7.43 – 7.33 (m, 2H, Ar-$\underline{H}$), 7.25 – 7.14 (m, 2H, Ar-$\underline{H}$ and $CH_2C\underline{H}{=}CH$), 7.14 – 7.06 (m, 2H, Ar-$\underline{H}$), 6.05 (dt, *J* = 15.6, 1.6 Hz, 1H, $CH_2CH{=}C\underline{H}$), 3.83 (dt, *J* = 13.4, 4.0 Hz, 2H, $CH_2C\underline{H_2}NCH_2CH_2$), 3.23 – 3.08 (m, 2H, $CH_2CH_2NC\underline{H_2}CH_2$), 2.46 – 2.35 (m, 2H, $CH_2C\underline{H_2}CH{=}CH$), 1.71 – 1.59 (m, 2H, $C\underline{H_2}CH_2CH{=}CH$), 1.55 (dd, *J* = 8.7, 4.0 Hz, 4H, $C\underline{H_2}CH_2NCH_2C\underline{H_2}$), 1.46 (s, 9H, $C(C\underline{H_3})_3$) ppm; **^{13}C NMR (101 MHz, $CDCl_3$)** δ 165.0 ($\underline{C}(O)O$), 154.9 ($\underline{C}(O)N$), 151.4 ($CH_2\underline{C}H{=}CH$), 150.8 (Ar$\underline{C}$), 129.5 (Ar$\underline{C}$H), 125.9 (Ar$\underline{C}$H), 121.7 (Ar$\underline{C}$H), 120.9 ($CH_2CH{=}\underline{C}H$), 79.7 ($\underline{C}(CH_3)_3$), 69.7 (OH$\underline{C}$), 41.2 ($\underline{C}H_2CH_2CH{=}CH$), 39.8 ($CH_2\underline{C}H_2N\underline{C}H_2CH_2$), 36.8 ($\underline{C}H_2CH_2NCH_2\underline{C}H_2$), 28.6 ($C(\underline{C}H_3)_3$), 26.1 ($CH_2\underline{C}H_2CH{=}CH$) ppm; **HRMS** (ESI) m/z calcd. for $C_{21}H_{29}O_5NNa$ ($[M+Na]^+$) 398.1938, found 398.1935; **FT-IR (thin film)** ν_{max} 3659, 2981, 2888, 1665, 1383, 1250, 1152, 1073, 955, 819 cm^{-1}; **m.p.**: 94-96 °C.

Preparation of starting materials 3ac

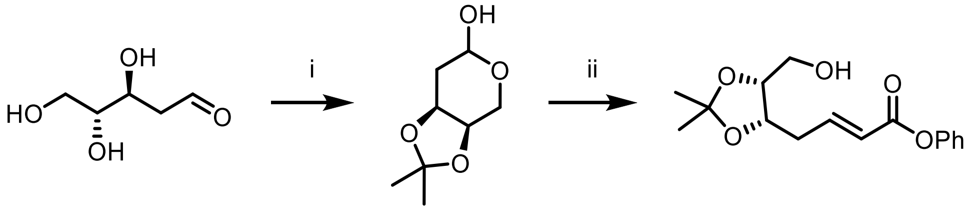

Scheme S10. Synthesis of starting materials **3ac**. i. *p*-TSA, acetone, 0 °C, 3 h. The reaction mixture was stirred at 0 °C for 3 h; ii. Phenyl-(triphenylphosphoranylidene)acetate **S6**, toluene, rt, 24 h.

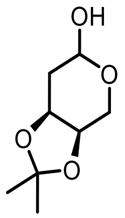

(3a*R*,7a*S*)-2,2-Dimethyltetrahydro-4H-[1,3]dioxolo[4,5-c]pyran-6-ol (S26) was prepared according to the literature procedure reported by R. W. Davis, *et. al.* [25] *p*-TSA (262.5 mg, 1.38 mmol, 0.1 eq.) was added to a solution of *D*-2-deoxyribose (1.85 g, 13.8 mmol, 1.0 eq.) in acetone (27.6 mL) at under N_2 at 0 °C. The reaction mixture was stirred at 0 °C for 3 h before quenching with a saturated aqueous solution of $NaHCO_3$ (20 mL). The slurry was filtered and washed with EtOAc (100 mL). The combined filtrate was evaporated to dryness under reduced pressure. Purification by silica gel chromatography (pentane/EtOAc = 1/1) afforded the title compound as a pale-yellow oil (1:0.5 (**A**:**B**) mixture of anomers) (284.3 mg, 12%). Data is consistent with the published literature.[26]

^{1}H NMR (400 MHz, $CDCl_3$) δ 5.25 (dd, *J* = 7.1, 4.3 Hz, 1H, C$\underline{H}$(**A**)), 5.07 (t, *J* = 3.7 Hz, 0.5H, C$\underline{H}$(**B**)), 4.47 (dt, *J* = 6.6, 4.3 Hz, 1H, C$\underline{H}$(**A**)), 4.41 (dt, *J* = 5.8, 4.5 Hz, 0.5H, C$\underline{H}$(**B**)), 4.22 – 4.13 (m, 1.5H, C$\underline{H}$(**A** and **B**)), 3.97 – 3.95 (m, 0.5H, C$\underline{H}_2$(**B**)), 3.95 – 3.90 (m, 1H, C$\underline{H}_2$(**A**)), 3.74 – 3.69 (m, 1H, C$\underline{H}_2$(**A**)), 3.69 – 3.66 (m, 0.5H, C$\underline{H}_2$(**B**)), 3.16 – 2.56 (brs, 1.5H, O$\underline{H}$(**A** and **B**)), 2.24 (dt, *J* = 14.8, 4.3 Hz, 1H, C$\underline{H}_2$(**A**)), 2.09 (dd, *J* = 4.6, 3.7 Hz, 1H, C$\underline{H}_2$(**B**)), 1.77 (ddd, *J* = 14.8, 7.1, 4.2 Hz, 1H, C$\underline{H}_2$(**A**)), 1.56 (d, *J* = 0.7 Hz, 1.5H, C$\underline{H}_3$(**B**)), 1.49 (d, *J* = 0.7 Hz, 3H, C$\underline{H}_3$(**A**)), 1.36 (d, *J* = 0.8 Hz, 1.5H, C$\underline{H}_3$(**B**)), 1.34 (d, *J* = 0.7 Hz, 3H, C$\underline{H}_3$(**A**)); **^{13}C NMR (101 MHz, $CDCl_3$)** δ 109.5 (O$\underline{C}$O(**B**)), 108.9 (O$\underline{C}$O(**A**)), 91.5 ($\underline{C}$H(**B**)), 91.1 ($\underline{C}$H(**A**)), 71.8 ($\underline{C}$H(**A**)), 71.3 ($\underline{C}$H(**B**)), 70.8 ($\underline{C}$H(**B**)), 70.5 ($\underline{C}$H(**A**)), 62.2 ($\underline{C}H_2$(**A**)), 60.8 ($\underline{C}H_2$(**B**)), 32.4 ($\underline{C}H_2$(**B**)), 32.3 ($\underline{C}H_2$(**A**)), 28.1 ($\underline{C}H_3$(**B**)), 27.4 ($\underline{C}H_3$(**A**)), 25.7 ($\underline{C}H_3$(**B**)), 25.5 ($\underline{C}H_3$(**A**)) ppm.

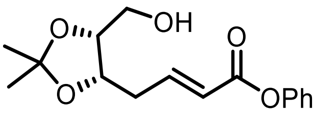

Phenyl (*E*)-4-((4*S*,5*R*)-5-(hydroxymethyl)-2,2-dimethyl-1,3-dioxolan-4-yl)but-2-enoate (3ac) was prepared according to the following procedure. A solution of **(3a*R*,7a*S*)-2,2-Dimethyltetrahydro-4H-[1,3]dioxolo[4,5-c]pyran-6-ol (S26)** (271.2 mg, 1.56 mmol, 1.0 eq.) in toluene (2 mL) was added to a solution of **phenyl-(triphenylphosphoranylidene)acetate (S6)** (927.0 mg, 2.34 mmol, 1.5 eq.) in toluene (7.8 mL) under N_2 at room temperature. The reaction mixture was stirred at room temperature for 24 hours before evaporating to dryness under reduced pressure. Purification by silica gel chromatography (pentane/Et_2O = 1/1) afforded the title compound as a white solid (210.0 mg, 46%).

The title compound decomposed in $CDCl_3$ within a couple of hours, indicated by the better cleanness of ^1H NMR spectrum compared to ^{13}C NMR spectrum.

^1H NMR (400 MHz, $CDCl_3$) δ 7.43 – 7.34 (m, 2H, Ar-H), 7.25 – 7.14 (m, 2H, Ar-H and CH_2CH=CH), 7.14 – 7.08 (m, 2H, Ar-H), 6.14 (dt, *J* = 15.7, 1.6 Hz, 1H, CH_2CH=CH), 4.36 (ddd, *J* = 9.0, 6.3, 4.5 Hz, 1H, OCH), 4.25 (td, *J* = 6.1, 4.9 Hz, 1H, OCH), 3.76 – 3.64 (m, 2H, $OCHCH_2$), 2.69 – 2.47 (m, 2H, $OCHCH_2$), 1.86 (s, 1H, OH), 1.50 (d, *J* = 0.8 Hz, 3H, CH_3), 1.39 (d, *J* = 0.8 Hz, 3H, CH_3) ppm; **^{13}C NMR (101 MHz, $CDCl_3$)** δ 164.7 (C(O)O), 150.8 (ArC), 147.0 (CH_2CH=CH), 129.5 (ArCH), 125.9 (ArCH), 123.0 (CH_2CH=CH), 121.7 (ArCH), 108.8 (OCO), 77.6 (CH), 75.4 (CH), 61.6 (CH_2), 32.8 (CH_2), 28.1 (CH_3), 25.4 (CH_3) ppm; **HRMS** (ESI) m/z calcd. for $C_{16}H_{20}O_5Na$ ($[M+Na]^+$) 315.1203, found 315.1203; **FT-IR (thin film)** ν_{max} 3449, 2935, 1732, 1654, 1592, 1492, 1456, 1371, 1320, 1250, 1196, 1163, 1149, 1112, 1070, 1044, 987, 904, 846, 755, 688, 652, 626, 611 cm^{-1}; **m.p.**: 39-40 °C; **$[\alpha]_D^{25}$** = −30.2 (c=0.95, $CHCl_3$).

Preparation of starting materials 3ad

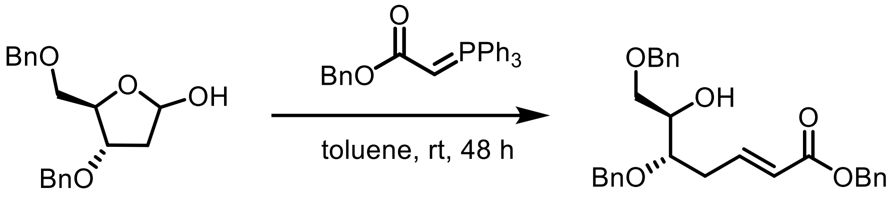

(4*S*,5*R*)-4-(Benzyloxy)-5-((benzyloxy)methyl)tetrahydrofuran-2-ol was prepared according to literature procedure.[27]

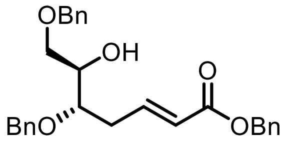

Benzyl (5*S*,6*R*,*E*)-5,7-bis(benzyloxy)-6-hydroxyhept-2-enoate (3ad) was prepared according to the following procedure. A solution of (4*S*,5*R*)-4-(Benzyloxy)-5-((benzyloxy)methyl)tetrahydrofuran-2-ol (512.8 mg, 1.64 mmol, 1.0 eq.) in toluene (2 mL) was added to a solution of benzyl-(triphenylphosphoranylidene)acetate (1.69 g, 4.12 mmol, 2.5 eq.) in toluene (5.5 mL) under N_2 at room temperature. The reaction mixture was stirred at room temperature for 48 hours before evaporating to dryness under reduced pressure. Purification by silica gel chromatography (pentane/Et_2O = 3/2) afforded the title compound as a colorless oil (403.4 mg, 55%).

^{1}H NMR (400 MHz, $CDCl_3$) δ 7.41 – 7.21 (m, 15H, Ar-H), 7.09 (dt, *J* = 15.7, 7.4 Hz, 1H, $CH_2C\underline{H}$=CH), 5.97 (dt, *J* = 15.6, 1.4 Hz, 1H, $CH_2C\underline{H}$=CH), 5.18 (s, 2H, $ArC\underline{H}_2O$), 4.62 – 4.44 (m, 4H, $ArC\underline{H}_2O$), 3.83 (tdd, *J* = 6.2, 4.9, 3.7 Hz, 1H, $BnOCH_2C\underline{H}$), 3.66 – 3.53 (m, 3H, $C\underline{H}CH_2CH$=CH and $BnOC\underline{H}_2$), 2.63 –

2.48 (m, 2H, C<u>H</u>$_2$CH=CH), 2.42 (d, *J* = 5.0 Hz, 1H, O<u>H</u>) ppm; **^{13}C NMR (101 MHz, CDCl$_3$)** δ 166.2 (<u>C</u>(O)O), 146.0 (CH$_2$<u>C</u>H=CH), 137.97 (Ar<u>C</u>), 137.89 (Ar<u>C</u>), 136.3 (Ar<u>C</u>), 128.7 (Ar<u>C</u>H), 128.63 (Ar<u>C</u>H), 128.57 (Ar<u>C</u>H), 128.31 (Ar<u>C</u>H), 128.28 (Ar<u>C</u>H), 128.1 (Ar<u>C</u>H), 128.03 (Ar<u>C</u>H), 128.00 (Ar<u>C</u>H), 127.97 (Ar<u>C</u>H), 123.6 (CH$_2$CH=<u>C</u>H), 78.4 (<u>C</u>HCH$_2$CH=CH), 73.6 (Ar<u>C</u>H$_2$O), 72.5 (Ar<u>C</u>H$_2$O), 71.5 (BnOCH$_2$<u>C</u>H), 70.9 (BnO<u>C</u>H$_2$), 66.2 (Ar<u>C</u>H$_2$O), 33.4 (<u>C</u>H$_2$CH=CH) ppm; **HRMS** (ESI) m/z calcd. for $C_{28}H_{30}O_5Na$ ([M+Na]$^+$) 469.1985, found 469.1982; **FT-IR (thin film)** ν_{max} 3483, 3031, 2866, 1716, 1654, 1496, 1454, 1315, 1265, 1214, 1165, 1073, 1027, 910, 750, 697, 608 cm^{-1}; **$[\alpha]_D^{25}$** = +16.5 (c=0.66, CHCl$_3$).

Preparation of starting materials 3ae

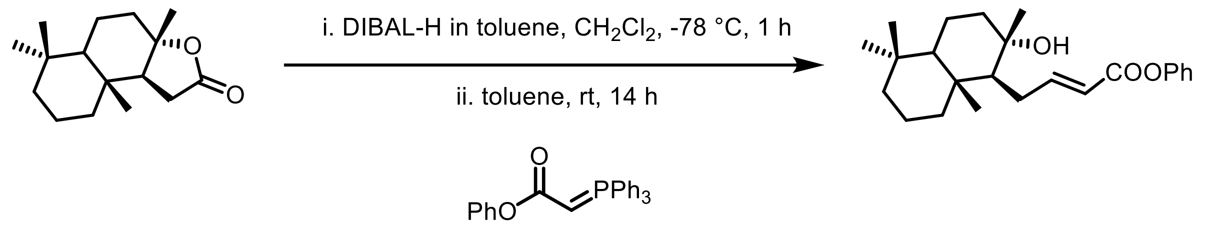

Phenyl (*E*)-4-((1*R*,2*R*,4a*S*,8a*S*)-2-hydroxy-2,5,5,8a-tetramethyldecahydronaphthalen-1-yl)but-2-enoate (3ae) was prepared according to the following procedure. According to the modified literature procedure reported by J. L. Wang *et. al.,* [28] DIBAL (25% wt solution in toluene) (4.72 mL, 7.1 mmol, 1.2 eq.) was added dropwise (0.4 mL/min) to a solution of sclareolide (1.48 g, 5.9 mmol, 1.0 eq.) in CH_2Cl_2 (50 mL) under N_2 at −78 °C. The reaction mixture was stirred at the same temperature for 1 hour before quenching with 2M HCl (61.5 mL) at −78 °C. The resulting mixture was gradually warmed up to room temperature and extracted with CH_2Cl_2 (3 x 100 mL). The combined organic layers were washed with brine (100 mL), dried over Na_2SO_4, filtered and evaporated to dryness under reduced pressure to afford the hemiacetal (1.34 g, 90%) as a white solid which was used as crude for next step without any further purification.

hemiacetal (504.8 mg, 2 mmol, 1.0 eq.) was added to a solution of phenyl-(triphenylphosphoranylidene)acetate **S6** (1.19 g, 3 mmol, 1.5 eq.) in toluene (10 mL) under N_2 at room temperature. The reaction mixture was stirred at room temperature for 14 hours before evaporating to dryness under reduced pressure. Purification by silica gel chromatography (pentane/EtOAc = 9/1) afforded the title compound as a white solid (279.1 mg, 38%).

^{1}H NMR (400 MHz, $CDCl_3$) δ 7.42 – 7.34 (m, 2H, Ar-H), 7.34 – 7.24 (m, 1H, $CH_2C\underline{H}$=CH), 7.24 – 7.18 (m, 1H, Ar-H), 7.15 – 7.07 (m, 2H, Ar-H), 6.00 (dt, *J* = 15.6, 1.6 Hz, 1H, CH_2CH=C$\underline{H}$), 2.52 (dddd, *J* = 15.4, 7.0, 5.0, 1.7 Hz, 1H, $C\underline{H}_2CH$=CH), 2.32 (dddd, *J* = 15.4, 7.4, 5.3, 1.5 Hz, 1H, $C\underline{H}_2CH$=CH), 1.91 (dt, *J* = 12.3, 3.1 Hz, 1H, $C\underline{H}_2$), 1.73 – 1.53 (m, 3H, $C\underline{H}_2$), 1.51 – 1.23 (m, 6H, C$\underline{H}$, $C\underline{H}_2$ and OH), 1.22 – 1.09 (m, 4H, $C\underline{H}_2$ and $C\underline{H}_3$), 1.01 – 0.91 (m, 2H, C$\underline{H}$ and $C\underline{H}_2$), 0.88 (s, 3H, $C\underline{H}_3$), 0.86 (d, *J* = 0.9 Hz, 3H, $C\underline{H}_3$), 0.81 (s, 3H, $C\underline{H}_3$) ppm; **^{13}C NMR (101 MHz, $CDCl_3$)** δ 165.3 ($\underline{C}$O), 155.4 ($CH_2\underline{C}H$=CH), 151.0 (Ar$\underline{C}$), 129.5 (Ar$\underline{C}$H), 125.7 (Ar$\underline{C}$H), 121.8 (Ar$\underline{C}$H), 119.6 (CH_2CH=$\underline{C}$H), 74.0 ($\underline{C}$), 61.7 ($\underline{C}$H), 56.3 ($\underline{C}$H), 44.8 ($\underline{C}H_2$), 41.9 ($\underline{C}H_2$), 40.3 ($\underline{C}H_2$), 39.3 ($\underline{C}$), 33.6 ($\underline{C}$), 33.4 ($\underline{C}H_2$), 28.6 ($\underline{C}H_2CH$=CH), 24.1 ($\underline{C}H_3$), 21.6 ($\underline{C}H_2$), 20.6 ($\underline{C}H_3$), 18.6 ($\underline{C}H_2$), 15.5 ($\underline{C}H_3$) ppm; **HRMS** (ESI) m/z calcd. for $C_{24}H_{34}O_3Na$ ($[M+Na]^+$) 393.2400, found 393.2397; **FT-IR (thin film)** ν_{max} 3501, 3010, 2926, 1723, 1645, 1592, 1492, 1457, 1388, 1312, 1254, 1196, 1162, 1140, 1083, 1025, 972, 937, 909, 754, 688, 666, 642 cm^{-1}; **$[\alpha]D^{25}$** = −3.1 (c=0.50, ($CHCl_3$); **m.p.**: 64-66 °C

General procedure D for preparation of starting materials 3af to 3ai and 3ak to 3al

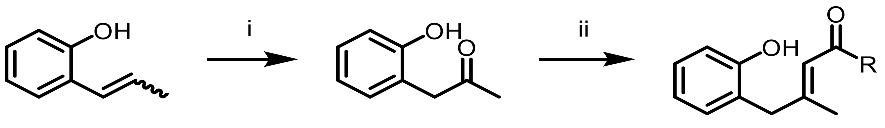

Scheme S11. Synthesis of starting materials **3af** to **3ai** and **3ak** to **3al**. i. $VO(acac)_2$, CH_2Cl_2, 5 mins at rt then *tert*-Butyl hydroperoxide, 40 °C 16 h; ii. Wittig reagents, toluene, rt, 24 h.

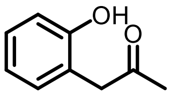

1-(2-Hydroxyphenyl)propan-2-one (S27) was prepared according to the following procedure. According to the modified literature procedure reported by A. Lattanzi, *et. al.,* [29] $VO(acac)_2$ (26.6 mg, 0.1 mmol, 0.02 eq.) was added to a solution of 2-(prop-1-en-1-yl)phenol (6.68 g, 49.8 mmol, 1.0 eq.) in anhydrous CH_2Cl_2 (200 mL) under N_2 at room temperature. The mixture was stirred for 5 mins before adding *tert*-Butyl hydroperoxide (5.5M in nonane) (10.9 mL, 59.8 mmol, 1.2 eq.). The reaction mixture was heated to 40 °C and stirred for 16 hours before evaporating to dryness under reduced pressure. Purification by silica gel chromatography (pentane/Et_2O = 7/3) afforded the title compound as a pale-yellow oil (3.97 g, 53%). Data is consistent with the published literature.

^{1}H NMR (400 MHz, $CDCl_3$) δ 7.24 – 7.12 (m, 1H, Ar-H), 7.08 (dd, *J* = 7.5, 1.7 Hz, 1H, Ar-H), 7.05 – 6.95 (m, 1H, OH), 6.94 – 6.84 (m, 2H, Ar-H), 3.75 (s, 2H, CH_2), 2.29 (d, *J* = 1.5 Hz, 3H, CH_3) ppm; **^{13}C NMR (101 MHz, $CDCl_3$)** δ 210.4 (C(O)), 155.2 (COH), 131.1 (ArCH), 129.2 (ArCH), 121.1 (ArCH), 117.52 (ArCH or ArC), 117.48 (ArCH or ArC), 46.6 (CH_2), 30.3 (CH_3) ppm; **HRMS** (ESI) m/z calcd. for $C_9H_9O_2$ ($[M-H]^-$) 149.0608, found 149.0599.

ii. 1-(2-Hydroxyphenyl)propan-2-one (S27) (1.0 eq.) in toluene (5 mL) was added to a solution of Wittig reagents (1.5 eq.) in toluene (0.1M) under N_2 at room temperature. The reaction mixture was stirred at room temperature for 24 hours before evaporating to dryness under reduced pressure. Purification by silica gel chromatography afforded starting materials **3af** to **3ai** and **3ak** to **3al**.

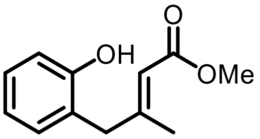

Methyl (*E*)-4-(2-hydroxyphenyl)-3-methylbut-2-enoate (3af) was prepared following **General Procedure D step ii**, using methyl 2-(triphenyl-l5-phosphaneylidene)acetate (5.12 g, 15.3 mmol, 1.5 eq.) to afford the title compound as a colorless oil (946.7 mg, 45%). Silica gel chromatography condition: CH_2Cl_2/EtOAc = 97.5/2.5. Data is consistent with the published literature.[9]

^{1}H NMR (400 MHz, CDCl$_3$) δ 7.17 – 7.11 (m, 1H, Ar-H), 7.10 – 7.04 (m, 1H, Ar-H), 6.89 (td, *J* = 7.5, 1.2 Hz, 1H, Ar-H), 6.78 (dd, *J* = 8.1, 1.2 Hz, 1H, Ar-H), 5.64 (h, *J* = 1.4 Hz, 1H, CH), 5.01 (brs, 1H, OH), 3.67 (s, 3H, OCH$_3$), 3.50 – 3.45 (m, 2H, CH$_2$), 2.19 (d, *J* = 1.3 Hz, 3H, CCH$_3$) ppm.

^{13}C NMR (101 MHz, CDCl$_3$) δ 167.4 (C(O)O), 158.7 (COH or C=CH), 154.0 (COH or C=CH), 131.4 (ArCH), 128.4 (ArCH), 124.1 (ArC), 121.1 (ArCH), 116.3 (CH), 115.8 (ArCH), 51.1 (OCH$_3$), 41.1 (CH$_2$), 19.1 (CCH$_3$) ppm.

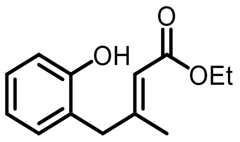

Ethyl (*E*)-4-(2-hydroxyphenyl)-3-methylbut-2-enoate (3ag) was prepared following **General Procedure D step ii**, using ethyl 2-(triphenyl-l5-phosphaneylidene)acetate (1.29 g, 3.7 mmol, 1.5 eq.) to afford the title compound as a colorless oil (391.5 mg, 73%). Silica gel chromatography condition: CH_2Cl_2/EtOAc = 98/2.

^{1}H NMR (400 MHz, $CDCl_3$) δ 7.13 (td, *J* = 7.7, 1.7 Hz, 1H, Ar-H), 7.08 (dd, *J* = 7.5, 1.7 Hz, 1H, Ar-H), 6.89 (td, *J* = 7.4, 1.2 Hz, 1H, Ar-H), 6.78 (dd, *J* = 8.0, 1.2 Hz, 1H, Ar-H), 5.64 (h, *J* = 1.3 Hz, 1H, CH), 5.07 (s, 1H, OH), 4.14 (q, *J* = 7.1 Hz, 2H, CH_2CH_3), 3.49 – 3.44 (m, 2H, CCH_2), 2.19 (d, *J* = 1.3 Hz, 3H, CCH_3), 1.26 (t, *J* = 7.1 Hz, 3H, CH_2CH_3) ppm; **^{13}C NMR (101 MHz, $CDCl_3$)** δ 167.1 (C(O)O), 158.3 (COH or C=CH), 154.1 (COH or C=CH), 131.4 (ArCH), 128.4 (ArCH), 124.1 (ArC), 121.1 (ArCH), 116.8 (CH), 115.8 (ArCH), 59.9 (CH_2CH_3), 41.1 (CCH_2), 19.1 (CCH_3), 14.4 (CH_2CH_3) ppm; **HRMS** (ESI) m/z calcd. for $C_{13}H_{15}O_3$ ([M-H]$^-$) 219.1027, found 219.1021; **FT-IR (thin film)** ν_{max} 3391, 2981, 2360, 1687, 1648, 1595, 1505, 1456, 1352, 1224, 1148, 916, 848, 754, 694, 686, 668 cm^{-1}.

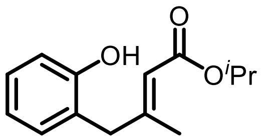

***iso*-Propyl (*E*)-4-(2-hydroxyphenyl)-3-methylbut-2-enoate (3ah)** was prepared following **General Procedure D step ii**, using *iso*-propyl 2-(triphenyl-l5-phosphaneylidene)acetate (1.11 g, 3.06 mmol, 1.5 eq.) to afford the title compound as a colorless oil (356.9 mg, 75%). Silica gel chromatography condition: CH_2Cl_2/EtOAc = 98/2.

^{1}H NMR (400 MHz, CDCl$_3$) δ 7.14 (td, *J* = 7.7, 1.7 Hz, 1H, Ar-H), 7.09 (dd, *J* = 7.5, 1.7 Hz, 1H, Ar-H), 6.89 (td, *J* = 7.4, 1.2 Hz, 1H, Ar-H), 6.79 (dd, *J* = 8.0, 1.1 Hz, 1H, Ar-H), 5.62 (h, *J* = 1.3 Hz, 1H, C=CH), 5.09 – 4.95 (m, 2H, OH and CH(CH_3)$_2$), 3.49 – 3.44 (m, 2H, CCH_2), 2.18 (d, *J* = 1.3 Hz, 3H, CCH_3), 1.24 (s, 3H, CH(CH_3)$_2$), 1.23 (s, 3H, CH(CH_3)$_2$) ppm; **^{13}C NMR (101 MHz, CDCl$_3$)** δ 166.6 (C(O)O), 157.7 (COH or C=CH), 154.1 (COH or C=CH), 131.4 (ArCH), 128.4 (ArCH), 124.1 (ArC), 121.1 (ArCH), 117.4 (C=CH), 115.8 (ArCH), 67.1 (CH(CH_3)$_2$), 41.1 (CCH_2), 22.1 (CH(CH_3)$_2$), 19.1 (CCH_3) ppm; **HRMS** (ESI) m/z calcd. for $C_{14}H_{19}O_3$ ([M+H]$^+$) 235.1329, found 235.1329; **FT-IR (thin film)** ν_{max} 3392, 2981, 2360, 1684, 1645, 1595, 1505, 1456, 1373, 1298, 1229, 1149, 1105, 1043, 916, 841, 754, 729, 694, 686 cm^{-1}.

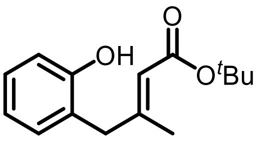

***tert*-Butyl (*E*)-4-(2-hydroxyphenyl)-3-methylbut-2-enoate (3ai)** was prepared following **General Procedure D step ii**, using *tert*-butyl 2-(triphenyl-l5-phosphaneylidene)acetate (702.7 mg, 1.87 mmol, 1.5 eq.) to afford the title compound as an off-white powder (206.3 mg, 67%). Silica gel chromatography condition: CH_2Cl_2/EtOAc = 97.5/2.5.

^{1}H NMR (400 MHz, CDCl$_3$) δ 7.16 – 7.10 (m, 1H, Ar-H), 7.08 (dd, *J* = 7.5, 1.7 Hz, 1H, Ar-H), 6.89 (td, *J* = 7.5, 1.2 Hz, 1H, Ar-H), 6.79 (dd, *J* = 8.0, 1.2 Hz, 1H, Ar-H), 5.59 (h, *J* = 1.3 Hz, 1H, CH), 5.13 (s, 1H, OH), 3.46 – 3.41 (m, 2H, CCH_2), 2.14 (d, *J* = 1.3 Hz, 3H, CCH_3), 1.47 (s, 9H, $C(CH_3)_3$) ppm; **^{13}C NMR (101 MHz, CDCl$_3$)** δ 166.6 (C(O)O), 156.4 (COH or C=CH), 154.2 (COH or C=CH), 131.4 (ArCH), 128.3 (ArCH), 124.2 (ArC), 121.0 (ArCH), 118.7 (CH), 115.9 (ArCH), 80.1 ($C(CH_3)_3$), 41.1 (CCH_2), 28.4 ($C(CH_3)_3$), 18.9 (CCH_3) ppm; **HRMS** (ESI) m/z calcd. for $C_{15}H_{20}O_3Na$ ($[M+Na]^+$) 271.1305, found 271.1305; **FT-IR (thin film)** ν_{max} 3404, 2980, 2360, 1682, 1646, 1595, 1456, 1391, 1366, 1318, 1239, 1141, 1098, 1044, 916, 848, 754, 686, 677 cm^{-1}; **m.p.**: 70-71 °C.

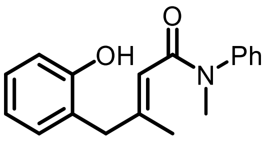

(*E*)-4-(2-Hydroxyphenyl)-*N*,3-dimethyl-*N*-phenylbut-2-enamide (3ak) was prepared following **General Procedure D step ii**, using ***N*-methyl-*N*-phenyl-2-(triphenyl-l5-phosphaneylidene)acetamide (S7)** (1.44 g, 3.52 mmol, 1.5 eq.) to afford the title compound as an white powder (108.8 mg, 17%). Silica gel chromatography condition: pentane/Et_2O = 2/3.

^{1}H NMR (400 MHz, $CDCl_3$) δ 7.46 – 7.16 (m, 3H, Ar-H), 7.14 – 7.07 (m, 2H, Ar-H), 7.06 – 6.96 (m, 1H, Ar-H), 6.74 (dq, *J* = 15.3, 7.0, 6.3 Hz, 3H, Ar-H), 6.29 (s, 1H, OH), 5.45 (s, 1H, CH), 3.30 (s, 3H, NCH_3), 3.27 (d, *J* = 10.7 Hz, 2H, CCH_2), 2.07 (d, *J* = 1.3 Hz, 3H, CCH_3) ppm; **^{13}C NMR (101 MHz, $CDCl_3$)** δ 168.0 (C(O)N), 154.5 (COH or C=CH), 151.4 (COH or C=CH), 144.1 (ArC), 130.8 (ArCH), 129.9 (ArCH), 129.5 (ArCH), 127.8 (ArCH), 127.2 (ArCH), 127.15 (ArCH), 127.06 (ArCH), 124.6 (ArC), 120.3 (ArCH), 119.1 (CH), 115.8 (ArCH), 40.4 (CCH_2), 37.0 (NCH_3), 18.8 (CCH_3); **HRMS** (ESI) m/z calcd. for $C_{18}H_{20}O_2N$ ($[M+H]^+$) 282.1489, found 282.1489; **FT-IR (thin film)** ν_{max} 3649, 2981, 2889, 2360, 2341, 1590, 1496, 1457, 1392, 1265, 1153, 1071, 952, 849, 806, 771, 694, 686 cm^{-1}; **m.p.**: 71-72 °C.

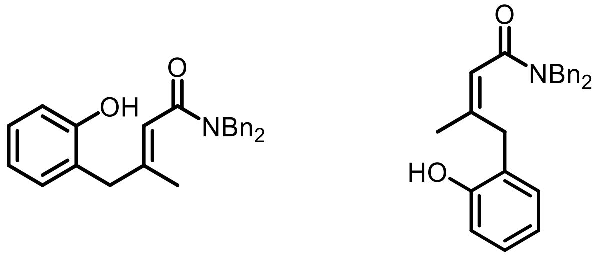

(*E*)-*N*,*N*-Dibenzyl-4-(2-hydroxyphenyl)-3-methylbut-2-enamide (3al) and **(*Z*)-*N*,*N*-Dibenzyl-4-(2-hydroxyphenyl)-3-methylbut-2-enamide (S9)** were prepared following **General Procedure D step ii**, using ***N*,*N*-dibenzyl-2-(triphenyl-l5-phosphaneylidene)acetamide (S8)** (2.21 g, 4.43 mmol, 1.5 eq.) to afford the title compounds as white powder.

E-isomer (**3al**): 384.1 mg, 35%; Silica gel chromatography condition: pentane/Et_2O = 2/3.

Z-isomer (**S9**): 396.0 mg, 36%; Silica gel chromatography condition: pentane/Et_2O = 7/3.

E-isomer (**3al**): **^{1}H NMR (400 MHz, $(CD_3)_2SO$)** δ 9.30 (s, 1H, OH), 7.40 – 7.17 (m, 8H, Ar-H), 7.15 – 7.07 (m, 2H, Ar-H), 7.00 (td, *J* = 7.7, 1.7 Hz, 1H, Ar-H), 6.90 (dd, *J* = 7.5, 1.7 Hz, 1H, Ar-H), 6.77 (dd, *J* = 8.0, 1.2 Hz, 1H, Ar-H), 6.66 (td, *J* = 7.4, 1.2 Hz, 1H, Ar-H), 5.94 (q, *J* = 1.3 Hz, 1H, CH), 4.45 (d, *J* = 22.2 Hz, 4H, NCH_2), 3.30 (s, 2H, CCH_2), 1.87 (d, *J* = 1.2 Hz, 3H, CH_3) ppm ; **^{13}C NMR (101 MHz, $(CD_3)_2SO$)** δ 167.7 (C(O)N), 155.3 (COH or C=CH), 149.0 (COH or C=CH), 137.8 (ArC), 137.3 (ArC), 130.4 (ArCH), 128.6 (ArCH), 128.4 (ArCH), 127.6 (ArCH), 127.4 (ArCH), 127.2 (ArCH), 127.0 (ArCH), 126.9 (ArCH), 124.4 (ArC), 118.9 (ArCH), 118.3 (CH), 115.0 (ArCH), 50.2 (NCH_2), 47.2 (NCH_2), 39.0 (CCH_2), 18.4 (CH_3) ppm ; **HRMS** (ESI) m/z calcd. for $C_{25}H_{26}O_2N$ ($[M+H]^+$) 372.1958, found 372.1955 ; **FT-IR (thin film)** ν_{max} 3660, 2981, 2889, 1593, 1495, 1454, 1382, 1237, 1155, 1082, 955, 822, 753, 700 cm^{-1} ; **m.p.**: 119-120 °C

Z-isomer (**S9**): **^{1}H NMR (400 MHz, $(CD_3)_2SO$)** δ 9.86 (s, 1H, O<u>H</u>), 7.43 – 7.19 (m, 10H, Ar-<u>H</u>), 7.10 (dd, *J* = 7.5, 1.7 Hz, 1H, Ar-<u>H</u>), 7.07 – 7.00 (m, 1H, Ar-<u>H</u>), 6.79 – 6.67 (m, 2H, Ar-<u>H</u>), 6.24 (d, *J* = 1.5 Hz, 1H, C<u>H</u>), 4.57 (d, *J* = 15.5 Hz, 4H, NC<u>H</u>$_2$), 3.68 (s, 2H, CC<u>H</u>$_2$), 1.66 (d, *J* = 1.3 Hz, 3H, C<u>H</u>$_3$) ppm ; **^{13}C NMR (101 MHz, $(CD_3)_2SO$)** δ 168.4 (<u>C</u>(O)N), 155.6 (<u>C</u>OH or <u>C</u>=CH), 150.3 (<u>C</u>OH or <u>C</u>=CH), 137.3 (Ar<u>C</u>), 136.9 (Ar<u>C</u>), 130.8 (Ar<u>C</u>H), 128.7 (Ar<u>C</u>H), 128.5 (Ar<u>C</u>H), 127.6 (Ar<u>C</u>H), 127.5 (Ar<u>C</u>H), 127.4 (Ar<u>C</u>H), 127.1 (Ar<u>C</u>H), 126.8 (Ar<u>C</u>H), 123.8 (Ar<u>C</u>), 118.8 (Ar<u>C</u>H), 118.2 (<u>C</u>H), 115.4 (Ar<u>C</u>H), 50.5 (N<u>C</u>H$_2$), 47.7 (N<u>C</u>H$_2$), 33.7 (C<u>C</u>H$_2$), 23.1 (<u>C</u>H$_3$) ppm ; **HRMS** (ESI) m/z calcd. for $C_{25}H_{26}O_2N$ ($[M+H]^+$) 372.1958, found 372.1960 ; **FT-IR (thin film)** ν_{max} 3659, 2981, 2889, 1603, 1473, 1382, 1250, 1152, 1073, 955, 819, 700 cm^{-1} ; **m.p.**: 49-50 °C

Preparation of starting materials 3aj

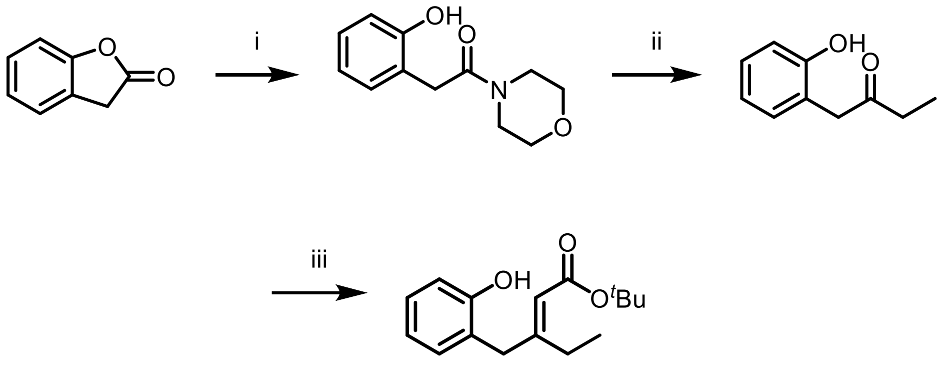

Scheme S12. Synthesis of starting materials **3aj**. i. morpholine, toluene, 110 °C, 20 h; ii. ethylcerium(III) chloride, THF, −78 °C, 2 h; iii. *tert*-butyl 2-(triphenyl-l5-phosphaneylidene)acetate, toluene, rt, 20 h.

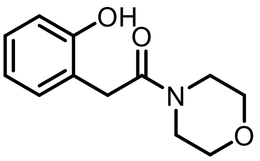

2-(2-Hydroxyphenyl)-1-morpholinoethan-1-one (S28) was prepared according to the following procedure. Morpholine (1.31 g, 15.0 mmol, 1.5 eq.) was added to a solution of benzofuran-2(3H)-one (1.34 g, 10.0 mmol, 1.0 eq.) in toluene (100 mL) under N_2 at room temperature. The reaction mixture was heated to 110 °C and stirred for 20 hours before evaporating to dryness under reduced pressure, affording the title compound as a pale-yellow solid (2.0 g, 90%) which was used as crude for next step without any purification. Data is consistent with the published literature.[30]

^{1}H NMR (400 MHz, CDCl$_3$) δ 9.33 (s, 1H, O<u>H</u>), 7.18 (td, *J* = 7.7, 1.7 Hz, 1H, Ar-<u>H</u>), 6.98 (ddd, *J* = 16.5, 7.8, 1.5 Hz, 2H, Ar-<u>H</u>), 6.83 (td, *J* = 7.4, 1.3 Hz, 1H, Ar-<u>H</u>), 3.73 (s, 2H, ArC<u>H</u>$_2$), 3.71 – 3.59 (m, 8H, NC<u>H</u>$_2$C<u>H</u>$_2$OC<u>H</u>$_2$C<u>H</u>$_2$) ppml; **^{13}C NMR (101 MHz, CDCl$_3$)** δ 171.7 (<u>C</u>(O)N), 156.8 (<u>C</u>OH), 130.2 (Ar<u>C</u>H),

129.2 (Ar<u>C</u>H), 120.7 (Ar<u>C</u>), 120.3 (Ar<u>C</u>H), 118.1 (Ar<u>C</u>H), 66.63 ($NCH_2\underline{C}H_2OCH_2CH_2$), 66.58 ($NCH_2CH_2O\underline{C}H_2CH_2$), 47.2 ($N\underline{C}H_2CH_2OCH_2CH_2$), 42.6 ($NCH_2CH_2OCH_2\underline{C}H_2$), 36.2 ($Ar\underline{C}H_2$) ppm.

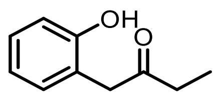

1-(2-Hydroxyphenyl)butan-2-one (S29) was prepared according to the following procedure. $CeCl_3$ (1.85 g, 7.5 mmol, 3.0 eq.) (anhydrous grade, stored and weighed in a glove box) was stirred at 90 °C for 30 mins and 135 °C for 2 hours under reduced pressure before cooling down to 0 °C. Anhydrous THF (15 mL) was added to the reaction vessel under N_2 at 0 °C. The resulting slurry was warmed to room temperature and stirred for 24 hours under N_2 before cooling down to −78 °C. Ethylmagnesium bromide (1M in THF) (7.25 mL, 7.25 mmol, 2.9 eq.) was added dropwise under N_2 at −78 °C. The resulting mixture was stirred at −78 °C for 1 hour, affording a solution of ethylcerium(III) chloride which was used as crude for the addition without any purification.

A solution of **2-(2-Hydroxyphenyl)-1-morpholinoethan-1-one (S28)** (552.8 mg, 2.5 mmol, 1.0 eq.) in anhydrous THF (2.5 mL) was added dropwise to the ethylcerium(III) chloride under N_2 at −78 °C. The resulting mixture was stirred at −78 °C for 2 hours before quenching with a saturated aqueous solution of NH_4Cl (20 mL). The aqueous layer was extracted with Et_2O (3 x 50 mL). The combined organic layers were washed with brine (50 mL), dried over Na_2SO_4, filtered and evaporated to dryness under reduced pressure. Purification by silica gel chromatography (pentane/Et_2O = 7/3) afforded the title compound as a yellow solid (245.9 mg, 60%). Data is consistent with the published literature.[31]

^{1}H NMR (400 MHz, $CDCl_3$) δ 7.53 (s, 1H, O<u>H</u>), 7.17 (ddd, *J* = 8.0, 7.3, 1.7 Hz, 1H, Ar-<u>H</u>), 7.07 (dd, *J* = 7.5, 1.7 Hz, 1H, Ar-<u>H</u>), 6.93 (dd, *J* = 8.1, 1.3 Hz, 1H, Ar-<u>H</u>), 6.87 (td, *J* = 7.4, 1.2 Hz, 1H, Ar-<u>H</u>), 3.73 (s, 2H, ArC<u>H</u>$_2$), 2.65 (q, *J* = 7.2 Hz, 2H, C<u>H</u>$_2$CH$_3$), 1.07 (t, *J* = 7.2 Hz, 3H, CH$_2$C<u>H</u>$_3$) ppm ; **^{13}C NMR (101 MHz, $CDCl_3$)** δ 213.5 (<u>C</u>(O)), 155.5 (<u>C</u>OH), 131.0 (Ar<u>C</u>H), 129.2 (Ar<u>C</u>H), 121.3 (Ar<u>C</u>), 120.9 (Ar<u>C</u>H), 117.7 (Ar<u>C</u>H), 45.7 (Ar<u>C</u>H$_2$), 36.5 (<u>C</u>H$_2$CH$_3$), 7.6 (CH$_2$<u>C</u>H$_3$) ppm ; **HRMS** (ESI) m/z calcd. for $C_{10}H_{12}O_2Na$ ($[M+Na]^+$) 187.0730, found 187.0730.

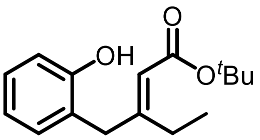

***tert*-Butyl (*E*)-3-(2-hydroxybenzyl)pent-2-enoate (3aj)** was prepared according to the following procedure. **1-(2-Hydroxyphenyl)butan-2-one (S29)** (213.2 mg, 1.30 mmol, 1.0 eq.) was added to a solution of *tert*-butyl 2-(triphenyl-l5-phosphaneylidene)acetate (733.6 mg, 1.95 mmol, 1.5 eq.) in toluene (6.5 mL) under N_2 at room temperature. The reaction mixture was stirred at room temperature for 20 hours before evaporating to dryness under reduced pressure. Purification by silica gel chromatography (pentane/Et_2O = 9/1) afforded the title compound as an off-white solid (229.3 mg, 68%).

^{1}H NMR (400 MHz, $CDCl_3$) δ 7.14 (td, *J* = 7.7, 1.7 Hz, 1H, Ar-H), 7.09 (dd, *J* = 7.5, 1.7 Hz, 1H, Ar-H), 6.90 (td, *J* = 7.4, 1.2 Hz, 1H, Ar-H), 6.79 (dd, *J* = 7.9, 1.2 Hz, 1H, Ar-H), 5.50 – 5.45 (m, 1H, CH), 4.82 (s, 1H, OH), 3.46 (d, *J* = 1.5 Hz, 2H, ArCH_2), 2.59 (q, *J* = 7.5 Hz, 2H, CH_2CH_3), 1.46 (s, 9H, C(CH_3)$_3$), 1.10 (t, *J* = 7.5 Hz, 3H, CH_2CH_3) ppm; **^{13}C NMR (101 MHz, $CDCl_3$)** δ 166.1 (C(O)O), 161.7 (COH or C=CH), 154.1 (COH or C=CH), 131.5 (ArCH), 128.3 (ArCH), 124.3 (ArC), 121.2 (ArCH), 118.2 (CH), 115.9 (ArCH), 80.0 (C(CH_3)$_3$), 38.5 (ArCH_2), 28.4 (C(CH_3)$_3$), 25.4 (CH_2CH_3), 13.3 (CH_2CH_3) ppm; **HRMS** (ESI) m/z calcd. for $C_{16}H_{22}O_3Na$ ([M+Na]$^+$) 285.1461, found 285.1461; **FT-IR (thin film)** ν_{max} 3416, 2935, 2357, 1683, 1642, 1595, 1507, 1456, 1367, 1313, 1229, 1173, 1141, 1100, 989, 847, 753, 663, 641, 620, 611 cm^{-1}; **m.p.**: 101-102 °C.

General procedure E for preparation of starting materials 3am, 3ap to 3aq

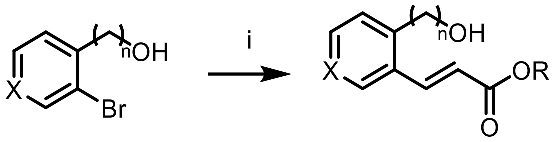

Scheme S13. Synthesis of starting materials **3am**, **3ap** to **3aq**. i. $Pd(OAc)_2$, acrylate, tris(*o*-tolyl)phosphine, triethylamine, 95 °C, 8-18 h.

Acrylate (1.5 eq.) was added to a solution of aryl-bromide (1.0 eq.), $Pd(OAc)_2$ (0.05 eq.) and tris(*o*-tolyl)phosphine (0.1 eq.) in triethylamine (1.44M) in a sealed tube under N_2 at room temperature. The sealed tube was flushed twice with argon gas before sealed with a screw cap. The reaction mixture was heated to 95 °C and stirred for designated time. After cooled back to room temperature, the resulting mixture was diluted with H_2O (50 mL) and extracted with EtOAc (4 x 50 mL). The combined organic layers were washed with brine (100 mL), dried over Na_2SO_4, filtered and evaporated to dryness under reduced pressure. Purification by silica gel chromatography afforded starting materials **3am**, **3ap** to **3aq**.

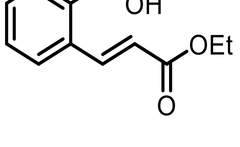

Ethyl (*E*)-3-(2-(hydroxymethyl)phenyl)acrylate (3am) was prepared following **General Procedure E**, using (2-bromophenyl)methanol (2.18 g, 11.63 mmol, 1.0 eq.) and ethyl acrylate (1.75 g, 17.45 mmol, 1.5 eq.) to afford the title compound as a pale-yellow oil (846.5 mg, 36%). Reaction time: 11 hours. Silica gel

chromatography condition: pentane/EtOAc = 7/3. (Longer reaction time led to a greater amount of oxy-Michael cyclization product) Data is consistent with the published literature.[32]

^{1}H NMR (400 MHz, $CDCl_3$) δ 8.02 (d, *J* = 15.9 Hz, 1H, C<u>H</u>=CH), 7.58 (dd, *J* = 7.6, 1.5 Hz, 1H, Ar-<u>H</u>), 7.47 – 7.41 (m, 1H, Ar-<u>H</u>), 7.37 (td, *J* = 7.4, 1.5 Hz, 1H, Ar-<u>H</u>), 7.31 (td, *J* = 7.5, 1.6 Hz, 1H, Ar-<u>H</u>), 6.38 (d, *J* = 15.9 Hz, 1H, CH=C<u>H</u>), 4.81 (s, 2H, C<u>H</u>$_2$OH), 4.25 (q, *J* = 7.1 Hz, 2H, C<u>H</u>$_2$CH$_3$), 2.34 (s, 1H, O<u>H</u>), 1.33 (t, *J* = 7.1 Hz, 3H, CH$_2$C<u>H</u>$_3$) ppm; **^{13}C NMR (101 MHz, $CDCl_3$)** δ 167.1 (<u>C</u>(O)O), 141.5 (<u>C</u>H=CH), 139.7 (Ar<u>C</u>), 133.2 (Ar<u>C</u>), 130.2 (Ar<u>C</u>H), 128.8 (Ar<u>C</u>H), 128.3 (Ar<u>C</u>H), 126.9 (Ar<u>C</u>H), 120.4 (CH=<u>C</u>H), 63.0 (<u>C</u>H$_2$OH), 60.7 (<u>C</u>H$_2$CH$_3$), 14.4 (CH$_2$<u>C</u>H$_3$) ppm; **HRMS** (ESI) m/z calcd. for $C_{12}H_{15}O_3$ ([M+H]$^+$) 207.1016, found 207.1017.

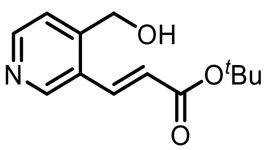

***tert*-Butyl (*E*)-3-(4-(hydroxymethyl)pyridin-3-yl)acrylate (3ap)** was prepared following **General Procedure E**, using (3-bromopyridin-4-yl)methanol (1.0 g, 5.33 mmol, 1.0 eq.) and *tert*-butyl acrylate (1.03 g, 8.00 mmol, 1.5 eq.) to afford the title compound as a white solid (337.8 mg, 27%). Reaction time: 8 hours. Silica gel chromatography condition: CH_2Cl_2/EtOAc = 1/1. (Longer reaction time led to a greater amount of oxy-Michael cyclization product).

^{1}H NMR (400 MHz, $CDCl_3$) δ 8.71 (s, 1H, Ar-<u>H</u>), 8.56 (d, *J* = 5.1 Hz, 1H, Ar-<u>H</u>), 7.71 (dd, *J* = 15.9, 0.6 Hz, 1H, C<u>H</u>=CH), 7.53 (dq, *J* = 5.2, 0.9 Hz, 1H, Ar-<u>H</u>), 6.36 (d, *J* = 16.0 Hz, 1H, CH=C<u>H</u>), 4.87 (d, *J* = 0.9 Hz, 2H, C<u>H</u>$_2$OH), 2.95 (brs, 1H, O<u>H</u>), 1.53 (s, 9H, C(C<u>H</u>$_3$)$_3$) ppm; **^{13}C NMR (101 MHz, $CDCl_3$)** δ

165.7 (C(O)O), 150.3 (ArCH), 148.5 (ArC), 147.5 (ArCH), 136.9 (ArCH), 128.6 (ArC), 124.3 (CH=CH), 121.5 (CH=CH), 81.4 ($C(CH_3)_3$), 61.4 (CH_2OH), 28.3 ($C(CH_3)_3$)ppm; **HRMS** (ESI) m/z calcd. for $C_{13}H_{18}O_3N$ ($[M+H]^+$) 236.1281, found 236.1280; **FT-IR (thin film)** ν_{max} 3660, 2981, 2888, 1710, 1462, 1382, 1323, 1252, 1154, 1073, 955, 831 cm^{-1}; **m.p.**: 84-85 °C.

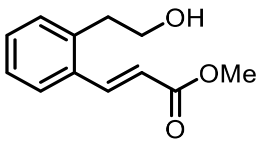

Methyl (*E*)-3-(2-(2-hydroxyethyl)phenyl)acrylate (3aq) was prepared following **General Procedure E**, using 2-(2-bromophenyl)ethan-1-ol (1.0 g, 5.0mmol, 1.0 eq.) and methyl acrylate (645.7 mg, 7.5 mmol, 1.5 eq.) to afford the title compound as a pale-yellow oil (543.0 mg, 53%). Reaction time: 18 hours. Silica gel chromatography condition: pentane/Et_2O = 2/3. Data is consistent with the published literature.[33]

^{1}H NMR (400 MHz, $CDCl_3$) δ 8.02 (d, *J* = 15.8 Hz, 1H, CH=CH), 7.62 – 7.55 (m, 1H, Ar-H), 7.36 – 7.30 (m, 1H, Ar-H), 7.29 – 7.23 (m, 2H, Ar-H), 6.37 (d, *J* = 15.8 Hz, 1H, CH=CH), 3.81 (d, *J* = 5.6 Hz, 5H, CH_2CH_2 and CH_3), 3.03 (t, *J* = 6.8 Hz, 2H, CH_2CH_2), 1.83 (s, 1H, OH) ppm; **^{13}C NMR (101 MHz, $CDCl_3$)** δ 167.5 (C(O)O), 142.3 (CH=CH), 138.2 (ArC), 133.7 (ArC), 131.0 (ArCH), 130.3 (ArCH), 127.3 (ArCH), 126.9 (ArCH), 119.6 (CH=CH), 63.5 (CH_2CH_2), 51.9 (CH_3), 36.6 (CH_2CH_2) ppm; **HRMS** (ESI) m/z calcd. for $C_{12}H_{15}O_3$ ($[M+H]^+$) 207.1016, found 207.1017

Preparation of starting materials 3an

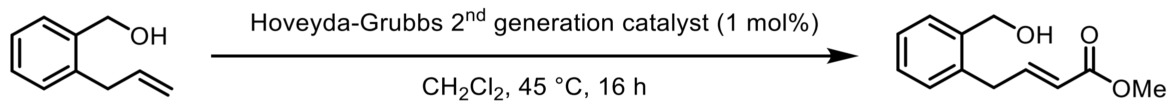

(2-allylphenyl)methanol was prepared according to literature procedure.[34]

Methyl (*E*)-4-(2-(hydroxymethyl)phenyl)but-2-enoate (3an) was prepared according to the following procedure. A solution of Hoveyda-Grubbs 2nd generation catalyst (12.6 mg, 0.02 mmol, 1 mol%) in degassed CH_2Cl_2 (1 mL) was added dropwise (0.5 mL/h) by using a syringe pump to a mixture of (2-allylphenyl)methanol (296.2 mg, 2.0 mmol, 1.0 eq.) and methyl acrylate (516.6 mg, 6.0 mmol, 3.0 eq.) under N_2 at 45 °C. The reaction mixture was stirred at 45 °C for 16 hours before directly loading onto the silica gel. Purification by silica gel chromatography (pentane/EtOAc = 3:1) afforded the title compound as a pale-yellow oil (117.7 mg, 29%).

^{1}H NMR (400 MHz, CDCl$_3$) δ 7.51 – 7.42 (m, 1H, Ar-H), 7.40 – 7.32 (m, 2H, Ar-H), 7.28 – 7.16 (m, 2H, $CH_2C\underline{H}$=CH and Ar-H), 5.82 (dt, *J* = 15.6, 1.8 Hz, 1H, $CH_2CH=C\underline{H}$), 4.76 (d, *J* = 5.2 Hz, 2H, $C\underline{H}_2OH$), 3.79 (s, 3H, $C\underline{H}_3$), 3.71 (dd, *J* = 6.4, 1.8 Hz, 2H, $C\underline{H}_2CH=CH$), 1.78 (t, *J* = 5.6 Hz, 1H, $O\underline{H}$) ppm; **^{13}C NMR (101 MHz, CDCl$_3$)** δ 167.0 ($\underline{C}$(O)O), 147.7 ($CH_2\underline{C}H=CH$), 138.7 (Ar$\underline{C}$), 136.1(Ar$\underline{C}$), 130.3 (Ar$\underline{C}$H), 128.7 (Ar$\underline{C}$H), 128.5 (Ar$\underline{C}$H), 127.4 (Ar$\underline{C}$H), 122.1 ($CH_2CH=\underline{C}H$), 63.4 ($\underline{C}H_2OH$), 51.6 ($\underline{C}H_3$), 35.2 ($\underline{C}H_2CH=CH$) ppm; **HRMS** (ESI) m/z calcd. for $C_{12}H_{14}O_3Na$ ($[M+Na]^+$) 229.0835, found 229.0835; **FT-IR (thin film)** ν_{max} 3428, 1719, 1653, 1492, 1436, 1338, 1275, 1205, 1167, 1106, 1039, 1013, 987, 948, 927, 841, 755, 611 cm^{-1}.

Preparation of starting materials 3ao

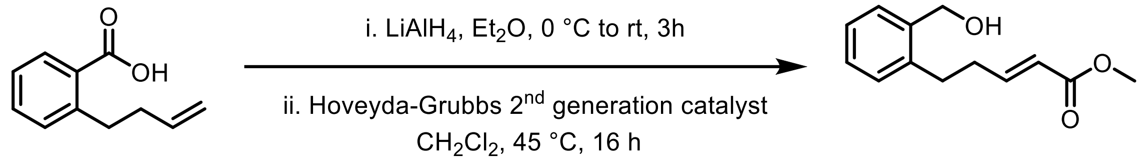

2-(but-3-en-1-yl)benzoic acid was prepared according to literature procedure.[35]

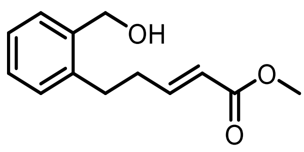

Methyl (*E*)-5-(2-(hydroxymethyl)phenyl)pent-2-enoate (3ao) was prepared according to the following procedure. 2-(but-3-en-1-yl)benzoic acid (768.4 mg, 4.37 mmol, 1.0 eq.) was added slowly to a suspension of $LiAlH_4$ (182.6 mg, 4.81 mmol, 1.1 eq.) in Et_2O (14.6 mL) under N_2 at 0 °C. The resulting mixture was warmed to room temperature and stirred for 3 hours before quenching with 10% HCl aqueous solution (10 mL). The aqueous layer was extracted with Et_2O (3 x 50 mL). The combined organic layers were washed with brine (50 mL), dried over Na_2SO_4, filtered and carefully evaporated to dryness under reduced pressure. Purification by silica gel chromatography (pentane/Et_2O= 7/3) afforded the alcohol as a pale-yellow oil (403.1 mg, 57%).

A solution of Hoveyda-Grubbs 2nd generation catalyst (5.4 mg, 0.0086 mmol, 0.5 mol%) in degassed CH_2Cl_2 (1 mL) was added dropwise (0.5 mL/h) by using a syringe pump to a mixture of the alcohol (278.0 mg, 1.72 mmol, 1.0 eq.) and methyl acrylate (444.3 mg, 5.16 mmol, 3.0 eq.) under N_2 at 45 °C. The reaction mixture was stirred at 45 °C for 16 hours before directly loading onto the silica gel. Purification by silica gel chromatography (pentane/Et_2O = 3:2) afforded the title compound as a white solid (251.7 mg, 67%).

^{1}H NMR (400 MHz, $CDCl_3$) δ 7.37 (dd, *J* = 7.4, 1.6 Hz, 1H, Ar-$\underline{H}$), 7.31 – 7.16 (m, 3H, Ar-$\underline{H}$), 7.02 (dt, *J* = 15.7, 6.9 Hz, 1H, $CH_2CH_2C\underline{H}{=}CH$), 5.86 (dt, *J* = 15.6, 1.6 Hz, 1H, $CH_2CH_2CH{=}C\underline{H}$), 4.71 (s, 2H, $C\underline{H}_2OH$), 3.72 (s, 3H, $C\underline{H}_3$), 2.90 – 2.81 (m, 2H, $C\underline{H}_2CH_2CH{=}CH$), 2.58 – 2.48 (m, 2H, $CH_2C\underline{H}_2CH{=}CH$), 1.77 (s, 1H, $O\underline{H}$) ppm; **^{13}C NMR (101 MHz, $CDCl_3$)** δ 167.2, ($\underline{C}(O)O$), 148.5 ($CH_2CH_2\underline{C}H{=}CH$), 139.2 (Ar$\underline{C}$), 138.4 (Ar$\underline{C}$), 129.4 (Ar$\underline{C}$H), 128.7 (Ar$\underline{C}$H), 128.3 (Ar$\underline{C}$H), 126.8 (Ar$\underline{C}$H), 121.6 ($CH_2CH_2CH{=}\underline{C}H$), 63.4 ($\underline{C}H_2OH$), 51.6 ($\underline{C}H_3$), 33.7 ($CH_2\underline{C}H_2CH{=}CH$), 30.9 ($\underline{C}H_2CH_2CH{=}CH$) ppm; **HRMS** (ESI) m/z calcd. for $C_{13}H_{16}O_3Na$ ($[M+Na]^+$) 243.0992, found 243.0992; **FT-IR (thin film)** ν_{max} 3392, 2921, 2364, 1721, 1656, 1437, 12821204, 1110, 1017, 852, 758, 646, 633, 610 cm^{-1}; **m.p.**: 54-55 °C.

Preparation of starting materials 3ar

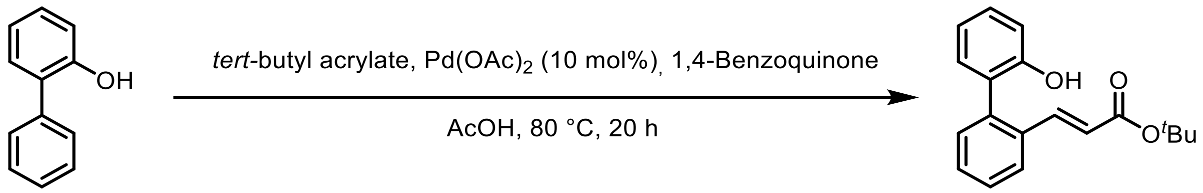

***tert*-Butyl (*E*)-3-(2'-hydroxy-[1,1'-biphenyl]-2-yl)acrylate (3ar)** was prepared according to the literature procedure reported by C. Zhang *et. al.* To an oven-dried screw-cap vial equipped with a magnetic stirrer bar was added [1,1'-biphenyl]-2-ol (851.1 mg, 5.0 mmol, 1.0 eq.), *tert*-butyl acrylate (1.28 g, 10.0 mmol, 2.0 eq.), $Pd(OAc)_2$ (112.3 mg, 0.5 mmol, 0.1 eq.), 1,4-Benzoquinone (540.5 mg, 5.0 mmol, 1.0 eq.) and AcOH (10 mL). The vial was closed under air atmosphere and heated to 80 °C for 20 hours. After cooled to room temperature, the reaction mixture was evaporated to dryness under reduced pressure. Purification by silica gel chromatography (pentane/Et_2O = 17:3) afforded the title compound as an off-white solid (795.7 mg, 54%). Data is consistent with the published literature.[36]

^{1}H NMR (400 MHz, $CDCl_3$) δ 7.81 – 7.74 (m, 1H, Ar-H), 7.52 – 7.39 (m, 3H, CH=CH and Ar-H), 7.38 – 7.33 (m, 1H, Ar-H), 7.29 (ddd, *J* = 8.2, 7.4, 1.8 Hz, 1H, Ar-H), 7.10 (dd, *J* = 7.6, 1.7 Hz, 1H, Ar-H), 7.03 – 6.93 (m, 2H, Ar-H), 6.34 (d, *J* = 16.0 Hz, 1H, CH=CH), 4.89 (s, 1H, OH), 1.46 (s, 9H, $C(CH_3)_3$) ppm; **^{13}C NMR (101 MHz, $CDCl_3$)** δ 166.2 (C(O)O), 152.8 (ArC), 141.4 (CH=CH), 137.6 (ArC), 134.1 (ArC), 131.3 (ArCH), 131.1 (ArCH), 130.3 (ArCH), 129.9 (ArCH), 128.7 (ArCH), 126.9 (ArCH), 126.1 (ArC), 121.6 (CH=CH), 120.8 (ArCH), 116.0 (ArCH), 80.6 ($C(CH_3)_3$), 28.3 ($C(CH_3)_3$) ppm; **HRMS** (ESI) m/z calcd. for $C_{19}H_{20}O_3Na$ ($[M+Na]^+$) 319.1305, found 319.1305; **m.p.**: 102-103 °C.

5 Preparation of Racemic Compounds

DBU (0.2 eq.) or BEMP (0.2 eq.) was added to a solution of starting materials (0.20 mmol, 1.0 eq.) in THF (0.1M) under N_2 at room temperature. The reaction was stirred at room temperature for 16 hours before loading directly onto silica gel. Purification by silica gel chromatography (pentane/Et_2O) afforded the pure samples of racemic compounds for HPLC analysis.

6 Preparation of Enantioenriched Products

General procedure F for the intramolecular oxy-Michael reaction

Corresponding alcohol (0.2 mmol, 1.0 eq.) was added to a solution of the *in situ* generated catalyst **E** (0.02 mmol, 0.1 eq.) in 2-Me-THF (0.025M, 8 mL) unless otherwise indicated under N_2 at room temperature. The reaction mixture was stirred at room temperature for 24 hours unless otherwise indicated before quenching with 1M AcOH in CH_2Cl_2 (4 mL). The resulting mixture was then evaporated to dryness under reduced pressure. Purification by silica gel chromatography (pentane/Et_2O) afforded the pure intramolecular oxy-Michael reaction products, which were taken the isolated yield and analysed by chiral HPLC.

General procedure G for the intramolecular oxy-Michael reaction

Corresponding alcohol (0.2 mmol, 1.0 eq) was added to a solution of the *in situ* generated catalyst **I** (0.02 mmol, 0.1 eq) in TBME (0.025M, 8 mL) unless otherwise indicated under N_2 at 0 °C. The reaction mixture was stirred at 0 °C for 24 hours unless otherwise indicated before quenching with 1M AcOH in CH_2Cl_2 (4 mL). The resulting mixture was then evaporated to dryness under reduced pressure. Purification by silica

gel chromatography (pentane/Et_2O) afforded the pure intramolecular oxy-Michael reaction products, which were taken the isolated yield and analysed by chiral HPLC.

General procedure H for the intramolecular oxy-Michael reaction

Corresponding alcohol (0.2 mmol, 1.0 eq.) was added to a solution of the *in situ* generated catalyst **K** (0.01 mmol, 0.05 eq.) in 2-Me-THF (0.1M, 2.0 mL) unless otherwise indicated under N_2 at room temperature. The reaction mixture was stirred at room temperature for 24 hours unless otherwise indicated before quenching with 1M AcOH in CH_2Cl_2 (0.5 mL). The resulting mixture was then evaporated to dryness under reduced pressure. Purification by silica gel chromatography (pentane/Et_2O) afforded the pure intramolecular oxy-Michael reaction products, which were taken the isolated yield and analysed by chiral HPLC.

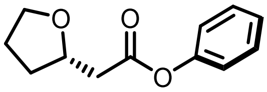

Phenyl (*S*)2-(tetrahydrofuran-2-yl)acetate (4a) was prepared following **General Procedure F**, using **phenyl (*E*)-6-hydroxyhex-2-enoate (3a)** (41.3 mg, 0.2 mmol, 1.0 eq.). Purification by silica gel chromatography (pentane/Et_2O = 4/1) afforded the title compound as a colorless oil (37.6 mg, 91%, 95:5 er). Data is consistent with the published literature.[9]

^{1}H NMR (400 MHz, $CDCl_3$) δ 7.42 – 7.32 (m, 2H, Ar-H), 7.25 – 7.18 (m, 1H, Ar-H), 7.13 – 7.07 (m, 2H, Ar-H), 4.38 (p, *J* = 6.8 Hz, 1H, CH), 3.94 (dt, *J* = 8.3, 6.7 Hz, 1H, CH_2O), 3.80 (td, *J* = 8.0, 6.4 Hz, 1H, CH_2O), 2.85 (dd, *J* = 15.2, 7.1 Hz, 1H, $CH_2C(O)O$), 2.72 (dd, *J* = 15.2, 6.1 Hz, 1H, $CH_2C(O)O$), 2.17 (dddd, *J* = 12.0, 8.2, 6.6, 5.3 Hz, 1H, $CHCH_2CH_2$), 2.01 – 1.89 (m, 2H, $CHCH_2CH_2$), 1.67 (ddt, *J* = 12.2, 8.7, 7.3 Hz, 1H, $CHCH_2CH_2$) ppm; **^{13}C NMR (101 MHz, $CDCl_3$)** δ 169.9 (C(O)O), 150.8 (ArC), 129.5 (ArCH), 125.9 (ArCH), 121.7 (ArCH), 75.3 (CH), 68.2 (CH_2O), 40.8 ($CH_2C(O)O$), 31.4 ($CHCH_2CH_2$), 25.7 ($CHCH_2CH_2$) ppm; **HRMS** (ESI) m/z calcd. for $C_{12}H_{15}O_3$ ($[M+H]^+$) 207.1016, found 207.1017; **$[\alpha]_D^{25}$** = 6.64 (c=0.12, $CHCl_3$); **HPLC:** Chiralcel IB, hexane/isopropanol = 95/5, 1.0 ml/min, λ = 210 nm, t_R (major) = 15.5 min, t_R (minor) = 11.0 min.

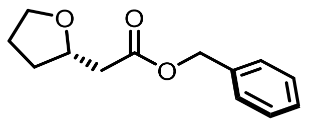

Benzyl (*S*)-2-(tetrahydrofuran-2-yl)acetate (4b) was prepared following **General Procedure F**, (variation from standard conditions: reaction carried out in 0.2M 2-Me-THF) using **benzyl (*E*)-6-hydroxyhex-2-enoate (3b)** (44.2 mg, 0.2 mmol, 1.0 eq.). Purification by silica gel chromatography (pentane/Et_2O = 7/3) afforded the title compound as a pale-yellow oil (41.5 mg, 94%, 94:6 er). Data is consistent with the published literature.[37]

^{1}H NMR (400 MHz, $CDCl_3$) δ 7.41 – 7.28 (m, 5H, Ar-H), 5.15 (s, 2H, CH_2Ar), 4.33 – 4.23 (m, 1H, CH), 3.87 (dt, *J* = 8.4, 6.8 Hz, 1H, CH_2O), 3.75 (ddd, *J* = 8.4, 7.5, 6.4 Hz, 1H, CH_2O), 2.66 (dd, *J* = 15.2, 7.3 Hz, 1H, CH_2C(O)O), 2.52 (dd, *J* = 15.2, 6.0 Hz, 1H, CH_2C(O)O), 2.08 (dddd, *J* = 12.2, 8.2, 6.6, 5.4 Hz, 1H, $CHCH_2CH_2$), 1.89 (ddddd, *J* = 12.2, 11.1, 9.2, 5.8, 3.8 Hz, 2H, $CHCH_2CH_2$), 1.55 (ddt, *J* = 12.1, 8.6, 7.4 Hz, 1H, $CHCH_2CH_2$) ppm; **^{13}C NMR (101 MHz, $CDCl_3$)** δ 171.2 (C(O)O), 136.0 (ArC), 128.6 (2C, ArCH), 128.3 (3C, ArCH), 75.3 (CH), 68.1 (CH_2O), 66.4 (CH_2Ar), 40.8 (CH_2C(O)O), 31.3 ($CHCH_2CH_2$), 25.7 ($CHCH_2CH_2$) ppm; **HRMS** (ESI) m/z calcd. for $C_{13}H_{17}O_3$ ($[M+H]^+$) 221.1172, found 221.1172; **$[\alpha]_D^{25}$** = 6.4 (c=0.40, $CHCl_3$); **HPLC:** Chiralcel OD, hexane/isopropanol = 97/3, 1.0 ml/min, λ = 254 nm, t_R (major) = 11.4 min, t_R (minor) = 9.5 min.

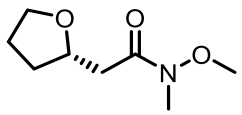

(*S*)-*N*-methoxy-*N*-methyl-2-(tetrahydrofuran-2-yl)acetamide (4c) was prepared following **General Procedure F**, (variation from standard conditions: reaction carried out in 0.1M CPME for 2 days) using **(*E*)-6-hydroxy-*N*-methoxy-*N*-methylhex-2-enamide (3c)** (34.8 mg, 0.2 mmol, 1.0 eq.). Purification by silica gel chromatography (pentane/acetone = 17/3) afforded the title compound as a colorless oil (30.0 mg, 87%, 97.5:2.5 er).

^{1}H NMR (400 MHz, CDCl$_3$) δ 4.26 (dq, *J* = 7.6, 6.5 Hz, 1H, C<u>H</u>), 3.85 (ddd, *J* = 8.5, 7.2, 6.3 Hz, 1H, C<u>H</u>$_2$O), 3.72 (ddd, *J* = 8.4, 7.4, 6.5 Hz, 1H, C<u>H</u>$_2$O), 3.68 (s, 3H, OC<u>H</u>$_3$), 3.16 (s, 3H, NC<u>H</u>$_3$), 2.82 (dd, *J* = 15.3, 6.8 Hz, 1H, C<u>H</u>$_2$C(O)N), 2.49 (dd, *J* = 15.4, 6.3 Hz, 1H, C<u>H</u>$_2$C(O)N), 2.10 (dddd, *J* = 12.1, 8.0, 6.4, 5.4 Hz, 1H, CHC<u>H</u>$_2$CH$_2$), 1.88 (dddd, *J* = 15.2, 7.6, 6.5, 3.0 Hz, 2H, CHCH$_2$C<u>H</u>$_2$), 1.54 (ddt, *J* = 12.2, 8.7, 7.5 Hz, 1H, CHC<u>H</u>$_2$CH$_2$) ppm; **^{13}C NMR (101 MHz, CDCl$_3$)** δ 172.2 (<u>C</u>(O)N), 75.6 (<u>C</u>H), 67.9 (<u>C</u>H$_2$O), 61.3 (O<u>C</u>H$_3$), 38.1(<u>C</u>H$_2$C(O)N), 32.1 (N<u>C</u>H$_3$), 31.6 (CH<u>C</u>H$_2$CH$_2$), 25.7 (CHCH$_2$<u>C</u>H$_2$) ppm; **HRMS** (ESI) m/z calcd. for $C_8H_{16}NO_3$ ([M+H]$^+$) 174.1125, found 174.1124; **FT-IR (thin film)** ν_{max} 3734, 3648, 2980, 2360, 2341, 1655, 1461, 1388, 1250, 1178, 1060, 1000, 966, 753, 667, 613 cm^{-1}; **$[\alpha]_D^{25}$** = −1.7 (c=0.49, CHCl$_3$); **HPLC:** Chiralcel OD, hexane/isopropanol = 95/5, 1.0 ml/min, λ = 200 nm, t_R (major) = 15.0 min, t_R (minor) = 13.1 min.

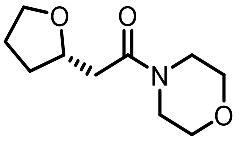

(*S*)-1-morpholino-2-(tetrahydrofuran-2-yl)ethan-1-one (4d) was prepared following **General Procedure F**, (variation from standard conditions: reaction carried out with 15% cat. **E** in 0.1M CPME for 2 days), using **(*E*)-6-hydroxy-1-morpholinohex-2-en-1-one (3d)** (27.7 mg, 0.14 mmol, 1.0 eq.). Purification by silica gel chromatography (pentane/acetone = 3/2) afforded the title compound as a white solid (25.5 mg, 92%, 99:1 er).

^{1}H NMR (400 MHz, $CDCl_3$) δ 4.22 (dq, *J* = 7.7, 6.4 Hz, 1H, $C\underline{H}$), 3.82 (dt, *J* = 8.4, 6.7 Hz, 1H, $C\underline{H}_2O$), 3.70 (dt, *J* = 8.3, 7.0 Hz, 1H, $C\underline{H}_2O$), 3.66 – 3.41 (m, 8H, $NC\underline{H}_2C\underline{H}_2OC\underline{H}_2C\underline{H}_2$), 2.66 (dd, *J* = 14.8, 6.5 Hz, 1H, $C\underline{H}_2C(O)N$), 2.43 (dd, *J* = 14.8, 5.9 Hz, 1H, $C\underline{H}_2C(O)N$), 2.18 – 2.05 (m, 1H, $CHC\underline{H}_2CH_2$), 1.95 – 1.81 (m, 2H, $CHCH_2C\underline{H}_2$), 1.61 – 1.48 (m, 1H, $CHC\underline{H}_2CH_2$) ppm; **^{13}C NMR (101 MHz, $CDCl_3$)** δ 169.6 ($\underline{C}(O)O$), 76.2 ($\underline{C}H$), 68.0 ($\underline{C}H_2O$), 66.9 ($NCH_2\underline{C}H_2O\underline{C}H_2CH_2$), 66.8 ($NCH_2\underline{C}H_2O\underline{C}H_2CH_2$), 46.4 ($N\underline{C}H_2CH_2OCH_2\underline{C}H_2$), 42.0 ($N\underline{C}H_2CH_2OCH_2\underline{C}H_2$), 39.2 ($\underline{C}H_2C(O)N$), 31.6 ($CH\underline{C}H_2CH_2$), 25.7 ($CHCH_2\underline{C}H_2$) ppm; **HRMS** (ESI) m/z calcd. for $C_{10}H_{18}NO_3$ ($[M+H]^+$) 200.1281, found 200.1280; **FT-IR (thin film)** ν_{max} 3853, 3837, 3734, 3648, 3503, 2980, 2885, 2360, 2341, 1716, 1635, 1558, 1541, 1507, 1458, 1437, 1381, 1233, 1151, 1114, 1067, 1034, 965, 850, 754, 669, 659 cm^{-1}; **m.p.**: 70-71 °C; **$[\alpha]_D^{25}$** = −3.6 (c=0.53, $CHCl_3$); **HPLC:** Chiralcel OD, hexane/isopropanol = 95/5, 1.0 ml/min, λ = 220 nm, t_R (major) = 24.2 min, t_R (minor) = 29.6 min.

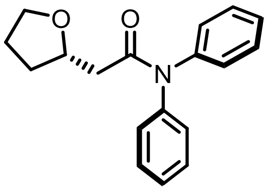

(*S*)-*N*,*N*-diphenyl-2-(tetrahydrofuran-2-yl)acetamide (4e) was prepared following **General Procedure F**, (variation from standard conditions: reaction carried out with 15% cat. **E** in 0.1M CPME for 2 days), using **(*E*)-6-hydroxy-*N*,*N*-diphenylhex-2-enamide (3e)** (58.2 mg, 0.2 mmol, 1.0 eq.). Purification by silica gel chromatography (pentane/acetone = 4/1) afforded the title compound as a white solid (57.6 mg, 99%, 96:4 er).

^{1}H NMR (400 MHz, $CDCl_3$) δ 7.56 – 7.02 (m, 10H, Ar-H), 4.34 (p, *J* = 6.7 Hz, 1H, CH), 3.74 (dtd, *J* = 26.6, 8.3, 6.5 Hz, 2H, CH_2O), 2.66 (dd, *J* = 15.4, 6.4 Hz, 1H, $CH_2C(O)N$), 2.39 (dd, *J* = 15.4, 6.7 Hz, 1H, $CH_2C(O)N$), 2.20 – 2.10 (m, 1H, $CHCH_2CH_2$), 1.92 – 1.76 (m, 2H, $CHCH_2CH_2$), 1.51 (dq, *J* = 12.3, 7.9 Hz, 1H, $CHCH_2CH_2$) ppm; **^{13}C NMR (101 MHz, $CDCl_3$)** δ 170.9 (C(O)N), 142.8 (ArC), 131.3 – 125.3 (ArCH), 76.3 (CH), 67.8 (CH_2O), 41.3 ($CH_2C(O)O$), 31.5 ($CHCH_2CH_2$), 25.7 ($CHCH_2CH_2$) ppm; **HRMS** (ESI) m/z calcd. for $C_{18}H_{20}NO_2$ ($[M+H]^+$) 282.1489, found 282.1488; **FT-IR (thin film)** ν_{max} 3009, 2362, 1668, 1594, 1491, 1452, 1347, 1254, 1216, 1160, 1061, 754, 701, 666 cm^{-1}; **m.p.**: 101-102 °C; **$[\alpha]_D^{25}$** = +7.1 (c=1.04, $CHCl_3$); **HPLC:** Chiralcel AD-H, hexane/isopropanol = 85/15, 1.0 ml/min, λ = 254 nm, t_R (major) = 14.6 min, t_R (minor) = 18.9 min.

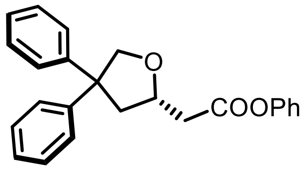

Phenyl (*S*)-2-(4,4-diphenyltetrahydrofuran-2-yl)acetate (4f) was prepared following **General Procedure F**, using **phenyl (*E*)-6-hydroxy-5,5-diphenylhex-2-enoate (3f)** (71.6 mg, 0.2 mmol, 1.0 eq.). Purification by silica gel chromatography (pentane/Et_2O = 4/1) afforded the title compound as a colorless oil (65.2 mg, 91%, 90:10 er).

^{1}H NMR (400 MHz, $CDCl_3$) δ 7.37 – 7.23 (m, 8H, Ar-H), 7.23 – 7.13 (m, 5H, Ar-H), 7.10 – 7.02 (m, 2H, Ar-H), 4.60 (dd, *J* = 8.7, 1.1 Hz, 1H, OCH_2), 4.54 (dq, *J* = 9.1, 6.2 Hz, 1H, CH), 4.22 (d, *J* = 8.8 Hz, 1H, OCH_2), 2.89 (dd, *J* = 15.5, 6.9 Hz, 1H, CH_2CHCH_2), 2.85 – 2.71 (m, 2H, CH_2CHCH_2), 2.49 (dd, *J* = 12.3, 9.2 Hz, 1H, CH_2CHCH_2) ppm; **^{13}C NMR (101 MHz, $CDCl_3$)** δ 169.7 (C(O)O), 150.7 (ArC), 145.9 (ArC), 145.6 (ArC), 129.5 (ArCH), 128.6 (ArCH), 128.5 (ArCH), 127.23 (ArCH), 127.17 (ArCH), 126.7 (ArCH), 126.5 (ArCH), 126.0 (ArCH), 121.7 (ArCH), 77.1 (OCH_2), 75.0 (CH), 56.2 (OCH_2C), 44.5 (CH_2CHCH_2), 40.9 (CH_2CHCH_2) ppm; **HRMS** (ESI) m/z calcd. for $C_{24}H_{23}O_3$ ($[M+H]^+$) 359.1642, found 359.1643; **FT-IR (thin film)** ν_{max} 3734, 3648, 2980, 2886, 2360, 2341, 1757, 1593, 1492, 1457, 1382, 1250, 1193, 1130, 1068, 954, 812, 756, 700, 669, 658, 623, 611 cm^{-1}; **$[\alpha]_D^{25}$** = −52.7 (c=0.33, $CHCl_3$); **HPLC:** Chiralcel AS-H, hexane/isopropanol = 80/20, 1.0 ml/min, λ = 210 nm, t_R (major) = 20.2 min, t_R (minor) = 7.8 min.

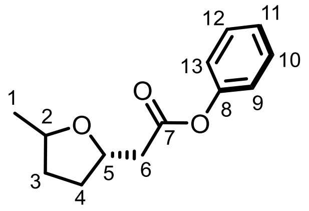

Phenyl 2-((2*S*)-5-methyltetrahydrofuran-2-yl)acetate (4g) was prepared following **General Procedure F**, using **phenyl (*E*)-6-hydroxyhept-2-enoate (3g)** (43.9 mg, 0.2 mmol, 1.0 eq.). Purification by silica gel chromatography (pentane/Et_2O = 17/3) afforded the title compound as a colorless oil (1:1 (**A**:**B**) mixture of diastereomers) (43.0 mg, 98%, 95.5:4.5 er, 98.5:1.5 er).

^{1}H NMR (400 MHz, $CDCl_3$) δ 7.42 – 7.32 (m, 4H, 9-13 (**A** and **B**)), 7.25 – 7.18 (m, 2H, 9-13 (**A** and **B**)), 7.10 (dq, *J* = 6.8, 1.1 Hz, 4H, 9-13 (**A** and **B**)), 4.53 (p, *J* = 6.7 Hz, 1H, 5 (**A** or **B**)), 4.35 (p, *J* = 6.8 Hz, 1H, 5 (**A** or **B**)), 4.19 (dp, *J* = 8.0, 6.0 Hz, 1H, 2 (**B**)), 4.03 (dp, *J* = 7.7, 6.2 Hz, 1H, 2 (**A**)), 2.86 (ddd, *J* = 15.0, 12.0, 6.7 Hz, 2H, 6 (**A** and **B**)), 2.71 (ddd, *J* = 15.1, 14.4, 6.5 Hz, 2H, 6 (**A** and **B**)), 2.30 – 1.98 (m, 4H, 3-4 (**A** and **B**)), 1.81 – 1.66 (m, 2H, 4 (**A** and **B**)), 1.61 – 1.47 (m, 2H, 3 (**A** and **B**)), 1.28 (d, *J* = 6.1 Hz, 3H, 1 (**A**)), 1.25 (d, *J* = 6.1 Hz, 3H, 1 (**B**)) ppm; **^{13}C NMR (101 MHz, $CDCl_3$)** δ 169.9 (7 (**A** or **B**)), 169.8 (7 (**A** or **B**)), 150.8 (8 (**A** and **B**)), 129.5 (9-13 (**A** and **B**)), 125.9 (9-13 (**A** and **B**)), 121.7 (9-13 (**A** and **B**)), 75.9 (2 (**A**)), 75.4 (5 (**A** or **B**)), 75.2 (2 (**B**)), 74.8 (5 (**A** or **B**)), 41.4 (6 (**A** or **B**)), 41.2 (6 (**A** or **B**)), 33.7 (3 (**A** or **B**)), 32.8 (3 (**A** or **B**)), 32.2 (4 (**A** or **B**)), 31.3 (4 (**A** or **B**)), 21.5 (1 (**A** or **B**)), 21.3 (1 (**A** or **B**)) ppm; **HRMS** (ESI) m/z calcd. for $C_{13}H_{17}O_3$ ($[M+H]^+$) 221.1172, found 221.1174; **FT-IR (thin film)** ν_{max} 2970, 2360, 2341, 1758, 1593, 1493, 1457, 1377, 1195, 1162, 1146, 1081, 1024, 934, 894, 814, 768, 690, 636, 618 cm^{-1}; **$[\alpha]_D^{25}$** = +7.9 (c=0.75, $CHCl_3$); **HPLC:** Chiralcel AD-H, hexane/isopropanol = 98/2, 1.0 ml/min, λ = 220 nm, diastereomer **A** t_R (major) = 10.4 min, t_R (minor) = 11.3 min; diastereomer **B** t_R (major) = 13.4 min, t_R (minor) = 15.6 min.

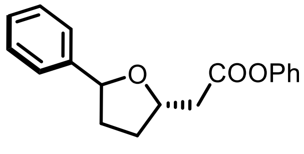

Phenyl 2-((2*S*)-5-phenyltetrahydrofuran-2-yl)acetate (4h) was prepared following **General Procedure F**, (variation from standard conditions: reaction carried out for 8 hours), using **phenyl (*E*)-6-hydroxy-6-phenylhex-2-enoate (3h)** (109.0 mg, 0.39 mmol, 1.0 eq.). Purification by silica gel chromatography (pentane/Et_2O = 17/3) afforded the title compound as a colorless oil (1:1 (**A**:**B**) mixture of diastereomers) (107.9 mg, 99%, 98:2 er, 94.5:5.5 er).

^{1}H NMR (400 MHz, $CDCl_3$) δ 7.31 (dddd, *J* = 12.8, 6.5, 4.1, 2.0 Hz, 12H, Ar-H (**A** and **B**)), 7.23 – 7.13 (m, 4H, Ar-H (**A** and **B**)), 7.10 – 7.01 (m, 4H, Ar-H (**A** and **B**)), 5.04 (dd, *J* = 7.8, 6.3 Hz, 1H, CCH (**A**)), 4.89 (t, *J* = 7.0 Hz, 1H, CCH (**B**)), 4.73 – 4.62 (m, 1H, C<u>H</u>CH_2C(O)O (**A** or **B**)), 4.52 (p, *J* = 6.6 Hz, 1H, C<u>H</u>CH_2C(O)O (**A** or **B**)), 2.93 (ddd, *J* = 16.7, 15.2, 6.9 Hz, 2H, CH_2C(O)O (**A** or **B**)), 2.77 (td, *J* = 15.6, 6.3 Hz, 2H, CH_2C(O)O (**A** or **B**)), 2.37 (dddd, *J* = 14.3, 7.7, 4.7, 2.9 Hz, 1H, CCHCH_2 (**A**)), 2.32 – 2.15 (m, 3H, CCHCH_2 (**A** and **B**) and CCHCH_2CH_2 (**A** and **B**)), 1.93 – 1.71 (m, 4H, CCHCH_2 (**A** and **B**) and CCHCH_2CH_2 (**A** and **B**)) ppm; **^{13}C NMR (101 MHz, $CDCl_3$)** δ 169.73 (C(O)O (**A** or **B**)), 169.71 (C(O)O (**A** or **B**)), 150.7 (2C, ArC (**A** and **B**)), 143.4 (ArC (**A** or **B**)), 142.9 (ArC (**A** or **B**)), 129.5 (4C, ArCH (**A** and **B**)), 128.40 (2C, ArCH (**A** and **B**)), 128.37 (2C, ArCH (**A** and **B**)), 127.3 (ArCH (**A** or **B**)), 127.2 (ArCH (**A** or **B**)), 125.9 (2C, ArCH (**A** and **B**)), 125.8 (2C, ArCH (**A** and **B**)), 125.5 (2C, ArCH (**A** and **B**)), 121.7 (4C, ArCH (**A** and **B**)), 81.3 (CCH (**B**)), 80.6 (CCH (**A**)), 75.9 (CHCH_2C(O)O (**A** or **B**)), 75.8 (CHCH_2C(O)O (**A** or **B**)), 41.1 (CHCH_2C(O)O (**A** or **B**)), 41.0 (CHCH_2C(O)O (**A** or **B**)), 35.2 (CCHCH_2 (**A**)), 34.3 (CCHCH_2 (**B**)), 32.2 (CCHCH_2CH_2 (**A** or **B**)), 31.3 (CCHCH_2CH_2 (**A** or **B**)) ppm; **HRMS** (ESI) m/z calcd.

for $C_{18}H_{19}O_3$ ($[M+H]^+$) 283.1329, found 283.1330; **FT-IR (thin film)** ν_{max} 2980, 2365, 1757, 1593, 1493, 1453, 1194, 1163, 1134, 1055, 1026, 931, 753, 699, 690, 652, 628, 620, 607 cm^{-1}; **$[\alpha]_D^{25}$** = +12.9 (c=0.34, $CHCl_3$); **HPLC:** Chiralcel AS-H, hexane/isopropanol = 99/1, 1.0 ml/min, λ = 220 nm, diastereomer **A** t_R (major) = 23.9 min, t_R (minor) = 26.5 min; diastereomer **B** t_R (major) = 41.3 min, t_R (minor) = 33.3 min.

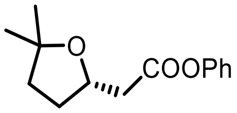

Phenyl (*S*)-2-(5,5-dimethyltetrahydrofuran-2-yl)acetate (4i) was prepared following **General Procedure F**, using **phenyl (*E*)-6-hydroxy-6-methylhept-2-enoate (3i)** (46.9 mg, 0.2 mmol, 1.0 eq.). Purification by silica gel chromatography (pentane/Et_2O = 4/1) afforded the title compound as a colorless oil (43.6 mg, 93%, 98:2 er).

^{1}H NMR (400 MHz, $CDCl_3$) δ 7.42 – 7.32 (m, 2H, Ar-H), 7.25 – 7.18 (m, 1H, Ar-H), 7.13 – 7.05 (m, 2H, Ar-H), 4.52 – 4.41 (m, 1H, CH), 2.88 (dd, *J* = 15.1, 6.2 Hz, 1H, CH_2C(O)O), 2.70 (dd, *J* = 15.1, 7.0 Hz, 1H, CH_2C(O)O), 2.30 – 2.15 (m, 1H, CH_2CH_2), 1.89 – 1.72 (m, 3H, CH_2CH_2), 1.30 (s, 3H, CH_3), 1.26 (s, 3H, CH_3) ppm; **^{13}C NMR (101 MHz, $CDCl_3$)** δ 169.9 (C(O)O), 150.8 (ArC), 129.5 (ArCH), 125.9 (ArCH), 121.7 (ArCH), 81.4(C), 74.7 (CH), 41.6 (CH_2C(O)O), 38.4 (CH_2CH_2), 31.9 (CH_2CH_2), 29.3 (CH_3), 28.2 (CH_3) ppm; **HRMS** (ESI) m/z calcd. for $C_{14}H_{19}O_3$ ($[M+H]^+$) 235.1329, found 235.1330; **FT-IR (thin film)** ν_{max} 2970, 2360, 1757, 1594, 1493, 1458, 1366, 1299, 1196, 1163, 1137, 1051, 936, 814, 754, 689 cm^{-1}; **$[\alpha]_D^{25}$** = −2.3 (c=0.77, $CHCl_3$); **HPLC:** Chiralcel AS-H, hexane/isopropanol = 98/2, 1.0 ml/min, λ = 210 nm, t_R (major) = 9.1 min, t_R (minor) = 7.7 min.

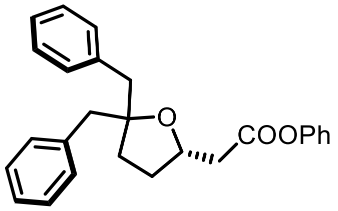

Phenyl (*S*)-2-(5,5-dibenzyltetrahydrofuran-2-yl)acetate (4j) was prepared following **General Procedure F**, (variation from standard conditions: reaction carried out in 0.2M 2-Me-THF for 2 days), using **phenyl (*E*)-6-benzyl-6-hydroxy-7-phenylhept-2-enoate (3j)** (77.1 mg, 0.2 mmol, 1.0 eq.). Purification by silica gel chromatography (pentane/Et_2O = 9/1) afforded the title compound as a white solid (67.8 mg, 88%, 92:8 er).

^{1}H NMR (400 MHz, $CDCl_3$) δ 7.41 – 7.32 (m, 2H, Ar-H), 7.32 – 7.15 (m, 11H, Ar-H), 7.13 – 7.05 (m, 2H, Ar-H), 4.13 (tt, *J* = 8.1, 5.7 Hz, 1H, CH), 2.90 (dd, *J* = 13.6, 11.2 Hz, 2H, CCH_2C), 2.76 (dd, *J* = 28.9, 13.5 Hz, 2H, CCH_2C), 2.60 (dd, *J* = 15.0, 7.7 Hz, 1H, CH_2C(O)O), 2.42 (dd, *J* = 15.0, 5.6 Hz, 1H, CH_2C(O)O), 1.92 – 1.76 (m, 2H, CH_2CH_2), 1.64 (ddt, *J* = 12.1, 6.9, 5.3 Hz, 1H, CH_2CH_2), 0.90 (dq, *J* = 12.0, 8.8 Hz, 1H, CH_2CH_2) ppm; **^{13}C NMR (101 MHz, $CDCl_3$)** δ 170.0 (C(O)O), 150.9 (ArCO), 138.10 (ArC), 138.03 (ArC), 131.1 (ArCH), 131.0 (ArCH), 129.5 (ArCH), 128.1 (ArCH), 127.9 (ArCH), 126.4 (ArCH), 126.3 (ArCH), 125.9 (ArCH), 121.7 (ArCH), 86.0 (C), 75.6 (CH), 46.8 (CCH_2C), 46.6 (CCH_2C), 41.0 (CH_2C(O)O), 33.0 (CH_2CH_2), 32.0 (CH_2CH_2) ppm; **HRMS** (ESI) m/z calcd. for $C_{26}H_{27}O_3$ ($[M+H]^+$) 387.1955, found 387.1955; **FT-IR (thin film)** ν_{max} 3027, 1757, 1594, 1493, 1454, 1278, 1194, 1162, 1143, 1084, 1051, 1029, 922, 754, 702, 668 cm^{-1}; **m.p.**: 38-39 °C; **$[\alpha]_D^{25}$** = +16.2 (c=1.21, $CHCl_3$); **HPLC:** Chiralcel AD-H, hexane/isopropanol = 95/5, 1.0 ml/min, λ = 240 nm, t_R (major) = 10.9 min, t_R (minor) = 9.0 min.

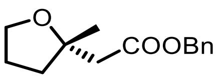

Benzyl (*S*)-2-(2-methyltetrahydrofuran-2-yl)acetate (4k) was prepared following **General Procedure F**, (variation from standard conditions: reaction carried out with 15% cat. **E** in 0.2M 2-Me-THF at 50 °C for 3 days), using **benzyl (*E*)-6-hydroxy-3-methylhex-2-enoate (3k)** (46.2 mg, 0.2 mmol, 1.0 eq.). Purification by silica gel chromatography (pentane/Et_2O = 4/1) afforded the title compound as a colorless oil (24 mg, 52%, 94:6 er). Data is consistent with the published literature.[38]

^{1}H NMR (400 MHz, $CDCl_3$) δ 7.41 – 7.28 (m, 5H, Ar-H), 5.13 (s, 2H, CH_2Ar), 3.90 – 3.75 (m, 2H, CH_2CH_2O), 2.59 (d, *J* = 2.7 Hz, 2H, $CH_2C(O)O$), 2.03 (ddd, *J* = 11.8, 8.6, 6.3 Hz, 1H, CH_2CH_2), 2.00 – 1.85 (m, 2H, CH_2CH_2), 1.73 (ddd, *J* = 11.8, 8.1, 6.0 Hz, 1H, CH_2CH_2), 1.32 (s, 3H, CH_3) ppm; **^{13}C NMR (101 MHz, $CDCl_3$)** δ 171.0 (C(O)O), 136.1 (ArC), 128.7 (ArCH), 128.4 (ArCH), 128.3 (ArCH), 81.0 (C), 67.5 (CH_2CH_2O), 66.3 (CH_2Ar), 45.6 ($CH_2C(O)O$), 36.9 (CH_2CH_2), 26.5 (CH_2CH_2), 25.9 (CH_3) ppm; **HRMS** (ESI) m/z calcd. for $C_{14}H_{19}O_3$ ($[M+H]^+$) 235.1329, found 235.1330; **FT-IR (thin film)** ν_{max} 2972, 2360, 1733, 1498, 1455, 1377, 1216, 1096, 1044, 754, 697, 668, 608 cm^{-1}; **$[\alpha]_D^{25}$** = +6.8 (c=0.64, $CHCl_3$); **HPLC:** Chiralcel OD, hexane/isopropanol = 95/5, 1.0 ml/min, λ = 210 nm, t_R (major) = 30.0 min, t_R (minor) = 31.6 min.

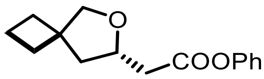

Phenyl (*S*)-2-(6-oxaspiro[3.4]octan-7-yl)acetate (4l) was prepared following **General Procedure F**, using **phenyl (*E*)-4-(1-(hydroxymethyl)cyclobutyl)but-2-enoate (3l)** (49.4 mg, 0.2 mmol, 1.0 eq.). Purification by silica gel chromatography (pentane/EtOAc = 9/1) afforded the title compound as a colorless oil (46.4 mg, 94%, 92:8 er).

^{1}H NMR (400 MHz, $CDCl_3$) δ 7.42 – 7.32 (m, 2H, Ar-H), 7.27 – 7.17 (m, 1H, Ar-H), 7.14 – 7.06 (m, 2H, Ar-H), 4.42 (ddt, *J* = 8.2, 7.2, 6.1 Hz, 1H, CH), 3.84 (d, *J* = 8.4 Hz, 1H, CH_2O), 3.77 (d, *J* = 8.4 Hz, 1H, CH_2O), 2.85 (dd, *J* = 15.3, 7.2 Hz, 1H, $CH_2C(O)O$), 2.71 (dd, *J* = 15.3, 6.0 Hz, 1H, $CH_2C(O)O$), 2.26 (dd, *J* = 12.3, 6.4 Hz, 1H, $CHCH_2C$), 2.15 – 2.00 (m, 4H, CH_2 (cyclobutane)), 1.98 – 1.81 (m, 2H, CH_2 (cyclobutane)), 1.74 (dd, *J* = 12.4, 8.2 Hz, 1H, $CHCH_2C$) ppm; **^{13}C NMR (101 MHz, $CDCl_3$)** δ 169.8 (C(O)O), 150.7 (ArC), 129.5 (ArCH), 125.9 (ArCH), 121.7 (ArCH), 79.2 (CH_2O), 74.9 (CH), 46.2 ($CHCH_2C$), 45.1 ($CHCH_2C$), 41.1 ($CH_2C(O)O$), 33.1 (CH_2 (cyclobutane)), 31.4 (CH_2 (cyclobutane)), 16.5 (CH_2 (cyclobutane)) ppm; **HRMS** (ESI) m/z calcd. for $C_{15}H_{19}O_3$ ([M+H]$^+$) 247.1329, found 247.1328; **FT-IR (thin film)** ν_{max} 2929, 2852, 2360, 1758, 1593, 1493, 1386, 1195, 1162, 1135, 1106, 1045, 929, 897, 815, 768, 690, 607 cm^{-1}; **$[\alpha]_D^{25}$** = −2.7 (c=0.82, $CHCl_3$); **HPLC:** Chiralcel AS-H, hexane/isopropanol = 70/30, 1.0 ml/min, λ = 210 nm, t_R (major) = 15.8 min, t_R (minor) = 6.0 min.

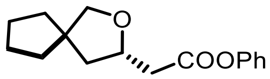

Phenyl (*S*)-2-(2-oxaspiro[4.4]nonan-3-yl)acetate (4m) was prepared following **General Procedure F**, using **phenyl (*E*)-4-(1-(hydroxymethyl)cyclopentyl)but-2-enoate (3m)** (52.3 mg, 0.2 mmol, 1.0 eq.). Purification by silica gel chromatography (pentane/Et_2O = 4/1) afforded the title compound as a colorless oil (51.7 mg, 99%, 93.5:6.5 er).

^{1}H NMR (400 MHz, $CDCl_3$) δ 7.45 – 7.32 (m, 2H, Ar-H), 7.25 – 7.19 (m, 1H, Ar-H), 7.14 – 7.06 (m, 2H, Ar-H), 4.48 (ddt, *J* = 8.7, 7.2, 6.2 Hz, 1H, CH), 3.70 (d, *J* = 8.1 Hz, 1H, CH_2O), 3.60 (d, *J* = 8.1 Hz, 1H, CH_2O), 2.89 (dd, *J* = 15.3, 7.2 Hz, 1H, $CH_2C(O)O$), 2.74 (dd, *J* = 15.3, 6.0 Hz, 1H, $CH_2C(O)O$), 2.09 (dd, *J* = 12.2, 6.5 Hz, 1H, $CHCH_2C$), 1.75 – 1.53 (m, 9H, $CHCH_2C$ and CH_2 (cyclopentane)) ppm; **^{13}C NMR (101 MHz, $CDCl_3$)** δ 169.9 (C(O)O), 150.7 (ArC), 129.5 (ArCH), 125.9 (ArCH), 121.7 (ArCH), 78.8 (CH_2O), 75.4 (OCH), 51.1 ($CHCH_2C$), 45.1 ($CHCH_2C$), 41.3 ($CH_2C(O)O$), 37.6 (CH_2 (cyclopentane)), 36.8 (CH_2 (cyclopentane)), 24.88 (CH_2 (cyclopentane)), 24.86 (CH_2 (cyclopentane)) ppm; **HRMS** (ESI) m/z calcd. for $C_{16}H_{21}O_3$ ($[M+H]^+$) 261.1485, found 261.1483; **FT-IR (thin film)** ν_{max} 2952, 2862, 1758, 1593, 1493, 1453, 1194, 1162, 1112, 1055, 929, 897, 754, 690, 667, 643 cm^{-1}; **$[\alpha]_D^{25}$** = −1.1 (c=0.86, $CHCl_3$); **HPLC:** Chiralcel AS-H, hexane/isopropanol = 95/5, 1.0 ml/min, λ = 230 nm, t_R (major) = 36.9 min, t_R (minor) = 11.2 min.

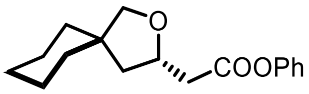

Phenyl (*S*)-2-(2-oxaspiro[4.5]decan-3-yl)acetate (4n) was prepared following **General Procedure F**, using **phenyl (*E*)-4-(1-(hydroxymethyl)cyclohexyl)but-2-enoate (3n)** (54.7 mg, 0.2 mmol, 1.0 eq.). Purification by silica gel chromatography (pentane/Et_2O = 17/3) afforded the title compound as a colorless oil (54.2 mg, 99%, 94:6 er).

^{1}H NMR (400 MHz, $CDCl_3$) δ 7.42 – 7.32 (m, 2H, Ar-H), 7.25 – 7.17 (m, 1H, Ar-H), 7.14 – 7.06 (m, 2H, Ar-H), 4.45 (dq, *J* = 8.9, 6.6 Hz, 1H, CH), 3.67 (d, *J* = 8.5 Hz, 1H, CH_2O), 3.59 (d, *J* = 8.5 Hz, 1H, CH_2O), 2.88 (dd, *J* = 15.3, 7.2 Hz, 1H, $CH_2C(O)O$), 2.73 (dd, *J* = 15.3, 6.0 Hz, 1H, $CH_2C(O)O$), 2.06 (dd, *J* = 12.4, 6.6 Hz, 1H, $CHCH_2C$), 1.63 – 1.32 (m, 11H, $CHCH_2C$ and CH_2 (cyclohexane)) ppm; **^{13}C NMR (101 MHz, $CDCl_3$)** δ 169.8 (C(O)O), 150.7 (ArC), 129.5 (ArCH), 125.9 (ArCH), 121.7 (ArCH), 78.6 (CH_2O), 74.9 (OCH), 51.1 ($CHCH_2C$), 44.1 ($CHCH_2C$), 41.3 ($CH_2C(O)O$), 37.0 (CH_2 (cyclohexane)), 35.6 (CH_2 (cyclohexane)), 30.4 (CH_2 (cyclohexane)), 26.1 (CH_2 (cyclohexane)), 24.1 (CH_2 (cyclohexane)), 23.6 (CH_2 (cyclohexane)) ppm; **HRMS** (ESI) m/z calcd. for $C_{17}H_{23}O_3$ ($[M+H]^+$) 275.1642, found 275.1641; **FT-IR (thin film)** ν_{max} 2923, 2851, 2360, 1758, 1593, 1492, 1449, 1389, 1297, 1194, 1163, 1148, 1115, 1056, 1023, 928, 896, 815, 768, 690 cm^{-1}; **$[\alpha]_D^{25}$** = −6.5 (c=0.97, $CHCl_3$); **HPLC:** Chiralcel AS-H, hexane/isopropanol = 95/5, 1.0 ml/min, λ = 240 nm, t_R (major) = 33.1 min, t_R (minor) = 10.7 min.

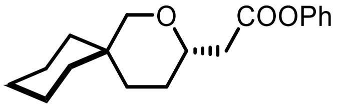

Phenyl (*S*)-2-(2-oxaspiro[5.5]undecan-3-yl)acetate (4o) was prepared following **General Procedure F**, (variation from standard conditions: reaction carried out with 8% cat. **E** for 3 days), using **phenyl (*E*)-5-(1-(hydroxymethyl)cyclohexyl)pent-2-enoate (3o)** (72.0 mg, 0.25 mmol, 1.0 eq.). Purification by silica gel chromatography (pentane/Et_2O = 9/1) afforded the title compound as a colorless oil (43.2 mg, 60%, 91:9 er).

^{1}H NMR (400 MHz, $CDCl_3$) δ 7.42 – 7.33 (m, 2H, Ar-H), 7.25 – 7.18 (m, 1H, Ar-H), 7.12 – 7.06 (m, 2H, Ar-H), 3.87 – 3.71 (m, 2H, CH_2O and CH), 3.16 (d, *J* = 11.4 Hz, 1H, CH_2O), 2.79 (dd, *J* = 15.2, 7.7 Hz, 1H, $CH_2C(O)O$), 2.66 (dd, *J* = 15.2, 5.3 Hz, 1H, $CH_2C(O)O$), 1.80 (dq, *J* = 13.4, 3.2 Hz, 1H, CH_2), 1.69 – 1.34 (m, 10H, CH_2), 1.27 (td, *J* = 13.0, 5.3 Hz, 1H, CH_2), 1.20 – 1.12 (m, 2H, CH_2) ppm; **^{13}C NMR (101 MHz, $CDCl_3$)** δ 170.0 (C(O)O), 150.8 (ArC), 129.5 (ArCH), 125.9 (ArCH), 121.7 (ArCH), 76.9 (CH_2O), 75.0 (CH), 41.5 ($CH_2C(O)O$), 36.6 (CH_2), 34.1 (CH_2), 32.1 (C), 31.3 (CH_2), 27.3 (CH_2), 26.9 (CH_2), 21.7 (CH_2), 21.6 (CH_2) ppm; **HRMS** (ESI) m/z calcd. for $C_{18}H_{25}O_3$ ($[M+H]^+$) 289.1798, found 289.1799; **FT-IR (thin film)** ν_{max} 2980, 2926, 2850, 2360, 2341, 1760, 1593, 1492, 1454, 1381, 1241, 1193, 1162, 1149, 1120, 1093, 1023, 938, 891, 817, 762, 688, 652, 622, 609 cm^{-1}; **$[\alpha]_D^{25}$** = −23.8 (c=0.74, $CHCl_3$); **HPLC:** Chiralcel AD-H, hexane/isopropanol = 95/5, 1.0 ml/min, λ = 210 nm, t_R (major) = 10.0 min, t_R (minor) = 8.7 min.

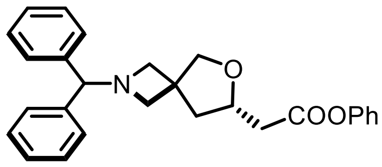

Phenyl (*S*)-2-(2-benzhydryl-6-oxa-2-azaspiro[3.4]octan-7-yl)acetate (4p) was prepared following **General Procedure F**, (variation from standard conditions: reaction carried out for 2 days), using **phenyl (*E*)-4-(1-benzhydryl-3-(hydroxymethyl)azetidin-3-yl)but-2-enoate (3p)** (82.9 mg, 0.2 mmol, 1.0 eq.). Purification by silica gel chromatography (pentane/Et_2O = 1/1) afforded the title compound as a white solid (56.4 mg, 68%, 84:16 er).

^{1}H NMR (400 MHz, $CDCl_3$) δ 7.44 – 7.38 (m, 4H, Ar-H), 7.38 – 7.33 (m, 2H, Ar-H), 7.26 (dd, *J* = 8.4, 6.8 Hz, 4H, Ar-H), 7.23 – 7.13 (m, 3H, Ar-H), 7.12 – 7.04 (m, 2H, Ar-H), 4.40 – 4.32 (m, 1H, OCH), 4.32 (s, 1H, NCH), 4.05 (d, *J* = 8.9 Hz, 1H, CH_2O), 3.90 (d, *J* = 8.9 Hz, 1H, CH_2O), 3.26 – 3.10 (m, 4H, CH_2 (azetidine)), 2.81 (dd, *J* = 15.4, 7.2 Hz, 1H, $CH_2C(O)O$), 2.68 (dd, *J* = 15.4, 6.0 Hz, 1H, $CH_2C(O)O$), 2.40 (dd, *J* = 12.7, 6.5 Hz, 1H, $CHCH_2C$), 1.88 (dd, *J* = 12.8, 7.9 Hz, 1H, $CHCH_2C$) ppm; **^{13}C NMR (101 MHz, $CDCl_3$)** δ 169.6 (C(O)O), 150.7 (ArCO), 142.3 (ArCCH), 129.5 (ArCH), 128.5 (ArCH), 127.6 (ArCH), 127.2 (ArCH), 126.0 (ArCH), 121.6 (ArCH), 78.1 (NCH), 77.6 (CH_2O), 75.0 (OCH), 64.1 (CH_2 (azetidine)), 63.5 (CH_2 (azetidine)), 43.4 (CH_2O), 41.2 ($CHCH_2C$), 40.8 ($CH_2C(O)O$) ppm; **HRMS** (ESI) m/z calcd. for $C_{27}H_{28}NO_3$ ($[M+H]^+$) 414.2064, found 414.2055; **FT-IR (thin film)** ν_{max} 3025, 2935, 2826, 2361, 1756, 1594, 1492, 1452, 1194, 1162, 1112, 1073, 1050, 1028, 928, 896, 753, 704, 667, 645, 609 cm^{-1}; **m.p.**: 80-81 °C; **$[\alpha]_D^{25}$** = −2.2 (c=1.05, $CHCl_3$); **HPLC:** Chiralcel AD-H, hexane/isopropanol = 93/7, 1.0 ml/min, λ = 220 nm, t_R (major) = 15.9 min, t_R (minor) = 15.0 min.

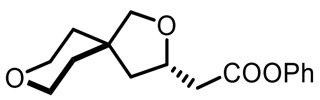

Phenyl (*S*)-2-(2,8-dioxaspiro[4.5]decan-3-yl)acetate (4q) was prepared following **General Procedure F**, using **phenyl (*E*)-4-(4-(hydroxymethyl)tetrahydro-2H-pyran-4-yl)but-2-enoate (3q)** (56.9 mg, 0.2 mmol, 1.0 eq.). Purification by silica gel chromatography (pentane/Et_2O = 3/7) afforded the title compound as a white solid (56.3 mg, 99%, 92.5:7.5 er).

^{1}H NMR (400 MHz, $CDCl_3$) δ 7.41 – 7.32 (m, 2H, Ar-H), 7.28 – 7.17 (m, 1H, Ar-H), 7.13 – 7.05 (m, 2H, Ar-H), 4.46 (dtd, *J* = 8.8, 6.9, 6.0 Hz, 1H, CH), 3.73 (d, *J* = 8.7 Hz, 1H, CCH_2O), 3.70 – 3.61 (m, 5H, CCH_2O and CCH_2CH_2O), 2.89 (dd, *J* = 15.5, 7.1 Hz, 1H, $CH_2C(O)O$), 2.75 (dd, *J* = 15.4, 6.0 Hz, 1H, $CH_2C(O)O$), 2.15 (dd, *J* = 12.5, 6.6 Hz, 1H, $CHCH_2C$), 1.70 – 1.57 (m, 4H, CCH_2CH_2O), 1.53 (dd, *J* = 12.6, 8.9 Hz, 1H, $CHCH_2C$) ppm; **^{13}C NMR (101 MHz, $CDCl_3$)** δ 169.6 (C(O)O), 150.6 (ArC), 129.5 (ArCH), 125.9 (ArCH), 121.6 (ArCH), 78.0 (CCH_2O), 74.8 (OCH), 65.8 (CCH_2CH_2O), 65.4 (CCH_2CH_2O), 43.8 ($CHCH_2C$), 41.8 ($CHCH_2C$), 41.0 ($CH_2C(O)O$), 36.7 (CCH_2CH_2O), 35.6 (CCH_2CH_2O) ppm; **HRMS** (ESI) m/z calcd. for $C_{16}H_{21}O_4$ ($[M+H]^+$) 277.1434, found 277.1435; **FT-IR (thin film)** ν_{max} 3852, 3734, 3648, 2980, 2849, 2360, 2341, 1758, 1593, 1492, 1457, 1388, 1303, 1232, 1194, 1163, 1107, 1055, 1016, 944, 898, 841, 754, 691, 668, 645, 625 cm^{-1}; **m.p.**: 28-30 °C; **$[\alpha]_D^{25}$** = −6.0 (c=1.05, $CHCl_3$); **HPLC:** Chiralcel AD-H, hexane/isopropanol = 95/5, 1.0 ml/min, λ = 220 nm, t_R (major) = 18.3 min, t_R (minor) = 19.4 min.

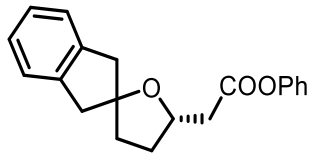

Phenyl (*S*)-2-(1',3',4,5-tetrahydro-3H-spiro[furan-2,2'-inden]-5-yl)acetate (4r) was prepared following **General Procedure F**, (variation from standard conditions: reaction carried out in 0.1M 2-Me-THF for 2 days), using **phenyl (*E*)-5-(2-hydroxy-2,3-dihydro-1H-inden-2-yl)pent-2-enoate (3r)** (61.5 mg, 0.2 mmol, 1.0 eq.). Purification by silica gel chromatography (pentane/Et_2O = 17/3) afforded the title compound as a white solid (59 mg, 96%, 95:5 er).

^{1}H NMR (400 MHz, $CDCl_3$) δ 7.43 – 7.34 (m, 2H, Ar-H), 7.29 – 7.14 (m, 5H, Ar-H), 7.14 – 7.06 (m, 2H, Ar-H), 4.57 (p, *J* = 6.8 Hz, 1H, CH), 3.24 (dd, *J* = 15.9, 4.2 Hz, 2H, CCH_2C), 3.10 – 2.89 (m, 3H, $CH_2C(O)O$ and CCH_2C), 2.77 (dd, *J* = 15.1, 7.1 Hz, 1H, $CH_2C(O)O$), 2.39 – 2.27 (m, 1H, CH_2CH_2), 2.17 – 2.00 (m, 2H, CH_2CH_2), 1.92 (ddt, *J* = 12.3, 8.4, 7.0 Hz, 1H, CH_2CH_2) ppm; **^{13}C NMR (101 MHz, $CDCl_3$)** δ 169.8 (C(O)O), 150.7 (ArC), 141.5 (ArC), 141.4 (ArC), 129.5 (2C, ArCH), 126.6 (2C, ArCH), 125.9 (ArCH), 124.7 (ArCH), 124.6 (ArCH), 121.7 (2C, ArCH), 91.6 (C), 75.0 (CH), 46.3 (CCH_2C), 45.5 (CCH_2C), 41.5 ($CH_2C(O)O$), 36.9 (CH_2CH_2), 31.6 (CH_2CH_2) ppm; **HRMS** (ESI) m/z calcd. for $C_{20}H_{21}O_3$ ($[M+H]^+$) 309.1308, found 309.1485; **FT-IR (thin film)** ν_{max} 2942, 1756, 1593, 1492, 1459, 1417, 1302, 1195, 1162, 1073, 1054, 1023, 932, 898, 814, 744, 689, 625 cm^{-1}; **m.p.**: 52-53 °C; **$[\alpha]_D^{25}$** = +15.3 (c=1.14, $CHCl_3$); **HPLC:** Chiralcel IB, hexane/isopropanol = 98/2, 1.0 ml/min, λ = 220 nm, t_R (major) = 17.3 min, t_R (minor) = 13.8 min.

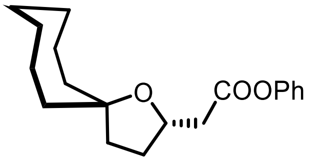

Phenyl (*S*)-2-(1-oxaspiro[4.7]dodecan-2-yl)acetate (4s) was prepared following **General Procedure F**, (variation from standard conditions: reaction carried out in 0.2M 2-Me-THF for 2 days), using **phenyl (*E*)-5-(1-hydroxycyclooctyl)pent-2-enoate (3s)** (60.3 mg, 0.2 mmol, 1.0 eq.). Purification by silica gel chromatography (pentane/Et_2O = 9/1) afforded the title compound as a colorless oil (59.1 mg, 98%, 98:2 er).

^{1}H NMR (400 MHz, $CDCl_3$) δ 7.42 – 7.32 (m, 2H, Ar-H), 7.25 – 7.19 (m, 1H, Ar-H), 7.13 – 7.05 (m, 2H, Ar-H), 4.41 (p, *J* = 6.8 Hz, 1H, CH), 2.87 (dd, *J* = 14.8, 6.3 Hz, 1H, $CH_2C(O)O$), 2.68 (dd, *J* = 14.9, 7.0 Hz, 1H, $CH_2C(O)O$), 2.23 – 2.09 (m, 1H, CH_2CH_2), 1.91 – 1.40 (m, 17H, CH_2CH_2) ppm; **^{13}C NMR (101 MHz, $CDCl_3$)** δ 170.0 (C(O)O), 150.8 (ArC), 129.5 (ArCH), 125.8 (ArCH), 121.7 (ArCH), 86.7 (C), 74.2 (CH), 41.6 ($CH_2C(O)O$), 37.1 (CH_2CH_2), 37.0 (CH_2CH_2), 35.7 (CH_2CH_2), 31.5 (CH_2CH_2), 28.5 (CH_2CH_2), 28.3 (CH_2CH_2), 24.8 (CH_2CH_2) , 23.0 (CH_2CH_2), 22.7 (CH_2CH_2) ppm; **HRMS** (ESI) m/z calcd. for $C_{19}H_{27}O_3$ ($[M+H]^+$) 303.1955, found 303.1956; **FT-IR (thin film)** ν_{max} 2920, 1758, 1594, 1492, 1446, 1298, 1194, 1162, 1144, 1116, 1072, 1051, 935, 898, 814, 753, 689 cm^{-1}; **$[\alpha]_D^{25}$** = +9.6 (c=1.07, $CHCl_3$); **HPLC:** Chiralcel AS-H, hexane/isopropanol = 98/2, 1.0 ml/min, λ = 220 nm, t_R (major) = 6.8 min, t_R (minor) = 7.9 min.

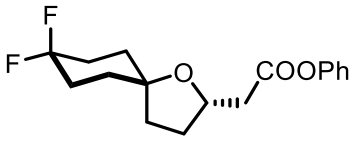

Phenyl (*S*)-2-(8,8-difluoro-1-oxaspiro[4.5]decan-2-yl)acetate (4t) was prepared following **General Procedure F**, (variation from standard conditions: reaction carried out in 0.2M 2-Me-THF for 2 days), using **phenyl (*E*)-5-(4,4-difluoro-1-hydroxycyclohexyl)pent-2-enoate (3t)** (61.9 mg, 0.2 mmol, 1.0 eq.). Purification by silica gel chromatography (pentane/Et_2O = 4/1) afforded the title compound as a white solid (58.8 mg, 95%, 95.5:4.5 er).

^{1}H NMR (400 MHz, $CDCl_3$) δ 7.44 – 7.35 (m, 2H, Ar-H), 7.29 – 7.20 (m, 1H, Ar-H), 7.13 – 7.05 (m, 2H, Ar-H), 4.57 – 4.36 (m, 1H, CH), 2.83 (dd, *J* = 14.8, 7.0 Hz, 1H, $CH_2C(O)O$), 2.73 (dd, *J* = 14.9, 6.2 Hz, 1H, $CH_2C(O)O$), 2.32 – 2.04 (m, 3H, CH_2), 1.94 (dtdd, *J* = 13.6, 7.7, 3.8, 2.0 Hz, 2H, CH_2), 1.88 – 1.72 (m, 6H, CH_2), 1.72 – 1.61 (m, 1H, CH_2) ppm; **^{13}C NMR (101 MHz, $CDCl_3$)** δ 169.8 (C(O)O), 150.8 (ArC), 129.5 (ArCH), 126.0 (ArCH), 123.7 (dd, *J* = 243.1, 240.1 Hz, CF), 121.6 (ArCH), 80.6 (d, *J* = 1.6 Hz, CH_2C), 74.9 (CH), 41.5 ($CH_2C(O)O$), 36.5 (d, *J* = 2.3 Hz, CH_2CH_2CH), 34.6 (dd, *J* = 8.5, 1.8 Hz, CH_2CH_2C), 33.1 (dd, *J* = 8.6, 1.7 Hz, CH_2CH_2C), 31.2 (CH_2CH_2CH), 30.9 (ddd, *J* = 25.5, 23.7, 2.3 Hz, 2C, CH_2CH_2C) ppm; **^{19}F NMR (377 MHz, $CDCl_3$)** δ −93.54 (d, *J* = 234.5 Hz), −102.91 (dt, *J* = 235.9, 32.8 Hz) ppm; **HRMS** (ESI) m/z calcd. for $C_{17}H_{20}O_3F_2Na$ ($[M+Na]^+$) 333.1273, found 333.1270; **FT-IR (thin film)** ν_{max} 3728, 3608, 2943, 2361, 2342, 1758, 1594, 1493, 1441, 1377, 1358, 1261, 1195, 1162, 1139, 1111, 1052, 982, 935, 895, 815, 770, 753, 720, 690, 660, 626 cm^{-1}; **m.p.**: 39-40 °C; **$[\alpha]_D^{25}$** = +17.2 (c=0.29, $CHCl_3$); **HPLC:** Chiralcel AD-H, hexane/isopropanol = 99/1, 1.0 ml/min, λ = 210 nm, t_R (major) = 13.5 min, t_R (minor) = 15.0 min.

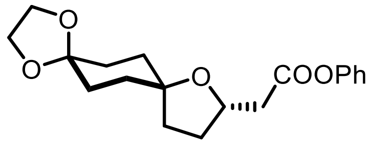

Phenyl (*S*)-2-(1,4,9-trioxadispiro[4.2.48.25]tetradecan-10-yl)acetate (4u) was prepared following **General Procedure F**, (variation from standard conditions: reaction carried out in 0.2M 2-Me-THF for 2 days), using **phenyl (*E*)-5-(8-hydroxy-1,4-dioxaspiro[4.5]decan-8-yl)pent-2-enoate (3u)** (66.2 mg, 0.2 mmol, 1.0 eq.). Purification by silica gel chromatography (pentane/Et_2O = 11/9) afforded the title compound as a colorless oil (58.2 mg, 88%, 97:3 er).

^{1}H NMR (400 MHz, $CDCl_3$) δ 7.41 – 7.32 (m, 2H, Ar-H), 7.25 – 7.16 (m, 1H, Ar-H), 7.11 – 7.03 (m, 2H, Ar-H), 4.50 – 4.39 (m, 1H, CH), 3.99 – 3.87 (m, 4H, $C\underline{H}_2C\underline{H}_2O$), 2.83 (dd, *J* = 14.8, 6.8 Hz, 1H, $C\underline{H}_2C(O)O$), 2.69 (dd, *J* = 14.8, 6.5 Hz, 1H, $C\underline{H}_2C(O)O$), 2.26 – 2.11 (m, 1H, $C\underline{H}_2CH$), 1.96 – 1.82 (m, 2H, $CC\underline{H}_2CH_2C$), 1.82 – 1.72 (m, 5H, $C\underline{H}_2CH$, $C\underline{H}_2CH_2CH$, $CC\underline{H}_2CH_2C$), 1.72 – 1.54 (m, 4H, $CC\underline{H}_2C\underline{H}_2C$) ppm; **^{13}C NMR (101 MHz, $CDCl_3$)** δ 169.9 ($\underline{C}(O)O$), 150.8 (Ar$\underline{C}$), 129.5 (Ar$\underline{C}$H), 125.9 (Ar$\underline{C}$H), 121.7 (Ar$\underline{C}$H), 108.8 (O$\underline{C}$O), 81.6 (O$\underline{C}$), 74.6 ($\underline{C}$H), 64.35 ($\underline{C}H_2CH_2O$), 64.26 ($CH_2\underline{C}H_2O$), 41.7 ($\underline{C}H_2C(O)O$), 36.4 ($C\underline{C}H_2CH_2C$), 35.7 ($C\underline{C}H_2CH_2C$), 34.3 ($\underline{C}H_2CH_2CH$), 31.9 ($C\underline{C}H_2CH_2C$), 31.8 ($C\underline{C}H_2CH_2C$), 31.3 ($\underline{C}H_2CH$) ppm; **HRMS** (ESI) m/z calcd. for $C_{19}H_{25}O_5$ ($[M+H]^+$) 333.1697, found 333.1695; **FT-IR (thin film)** ν_{max} 2935, 2878, 2360, 1756, 1593, 1493, 1445, 1373, 1195, 1163, 1143, 1100, 1049, 964, 931, 815, 769, 690, 659, 645, 628 cm^{-1}; **$[\alpha]_D^{25}$** = +15.0 (c=0.88, $CHCl_3$); **HPLC:** Chiralcel AS-H, hexane/isopropanol = 95/5, 1.0 ml/min, λ = 220 nm, t_R (major) = 18.9 min, t_R (minor) = 29.8 min.

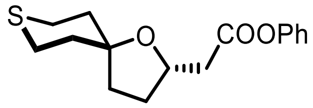

Phenyl (*S*)-2-(1-oxa-8-thiaspiro[4.5]decan-2-yl)acetate (4v) was prepared following **General Procedure F**, (variation from standard conditions: reaction carried out in 0.2M 2-Me-THF for 2 days), using **phenyl (*E*)-5-(4-hydroxytetrahydro-2H-thiopyran-4-yl)pent-2-enoate (3v)** (58.2 mg, 0.2 mmol, 1.0 eq.). Purification by silica gel chromatography (pentane/Et_2O = 4/1) afforded the title compound as a colorless oil (49.4 mg, 85%, 98:2 er).

^{1}H NMR (400 MHz, $CDCl_3$) δ 7.43 – 7.34 (m, 2H, Ar-H), 7.25 – 7.19 (m, 1H, Ar-H), 7.12 – 7.04 (m, 2H, Ar-H), 4.45 (qd, *J* = 7.0, 5.9 Hz, 1H, CH), 3.15 – 2.87 (m, 2H, SCH_2CH_2), 2.81 (dd, *J* = 14.9, 7.0 Hz, 1H, $CH_2C(O)O$), 2.70 (dd, *J* = 14.9, 6.3 Hz, 1H, $CH_2C(O)O$), 2.46 (p, *J* = 7.0, 6.2 Hz, 2H, SCH_2CH_2), 2.24 – 2.10 (m, 1H, CH_2CH_2CH), 1.92 (dq, *J* = 13.7, 5.3, 4.5 Hz, 2H, SCH_2CH_2), 1.86 – 1.68 (m, 5H, CH_2CH_2CH, CH_2CH_2CH and SCH_2CH_2) ppm; **^{13}C NMR (101 MHz, $CDCl_3$)** δ 169.8 (C(O)O), 150.7 (ArC), 129.5 (ArCH), 125.9 (ArCH), 121.6 (ArCH), 81.0 (C), 74.7 (CH), 41.6 ($CH_2C(O)O$), 39.7 (SCH_2CH_2), 38.1 (SCH_2CH_2), 37.7 (CH_2CH_2CH), 31.1 (CH_2CH_2CH), 25.8 (SCH_2CH_2), 25.7 (SCH_2CH_2) ppm; **HRMS** (ESI) m/z calcd. for $C_{16}H_{21}O_3S$ ($[M+H]^+$) 293.1206, found 293.1208; **FT-IR (thin film)** ν_{max} 2931, 1756, 1593, 1493, 1426, 1242, 1194, 1163, 1146, 1064, 937, 814, 753, 689, 654, 628 cm^{-1}; **$[\alpha]_D^{25}$** = +18.8 (c=0.90, $CHCl_3$); **HPLC:** Chiralcel AS, hexane/isopropanol = 98/2, 1.0 ml/min, λ = 230 nm, t_R (major) = 28.1 min, t_R (minor) = 15.4 min.

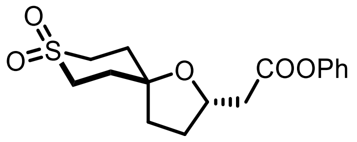

Phenyl (*S*)-2-(8,8-dioxido-1-oxa-8-thiaspiro[4.5]decan-2-yl)acetate (4w) was prepared following **General Procedure F**, (variation from standard conditions: reaction carried out in 0.2M 2-Me-THF for 2 days), using **phenyl (*E*)-5-(4-hydroxy-1,1-dioxidotetrahydro-2H-thiopyran-4-yl)pent-2-enoate (3w)** (64.8 mg, 0.2 mmol, 1.0 eq.). Purification by silica gel chromatography (Et_2O) afforded the title compound as a white solid (61.5 mg, 95%, 90:10 er).

^{1}H NMR (400 MHz, $CDCl_3$) δ 7.43 – 7.33 (m, 2H, Ar-H), 7.25 – 7.20 (m, 1H, Ar-H), 7.11 – 7.03 (m, 2H, Ar-H), 4.48 (tt, *J* = 7.3, 6.1 Hz, 1H, CH), 3.44 – 3.30 (m, 2H, $SC\underline{H_2}CH_2$), 2.87 (dp, *J* = 14.7, 3.9 Hz, 2H, $C\underline{H_2}C(O)O$), 2.82 – 2.70 (m, 2H, $SC\underline{H_2}CH_2$), 2.35 – 2.14 (m, 3H, $CH_2C\underline{H_2}CH$ and $SCH_2C\underline{H_2}$), 2.10 – 1.96 (m, 2H, $SCH_2C\underline{H_2}$), 1.95 – 1.74 (m, 3H, $CH_2C\underline{H_2}CH$ and $C\underline{H_2}CH_2CH$) ppm; **^{13}C NMR (101 MHz, $CDCl_3$)** δ 169.3 (C(O)O), 150.5 (ArC), 129.6 (ArCH), 126.0 (ArCH), 121.5 (ArCH), 78.7 (C), 75.4 (CH), 48.2 ($SC\underline{H_2}CH_2$), 48.1 ($SC\underline{H_2}CH_2$), 41.1 ($\underline{C}H_2C(O)O$), 37.0 ($CH_2C\underline{H_2}CH$ or $C\underline{H_2}CH_2CH$), 36.1 ($SCH_2\underline{C}H_2$), 34.5 ($SCH_2\underline{C}H_2$), 31.0 ($CH_2C\underline{H_2}CH$ or $C\underline{H_2}CH_2CH$) ppm; **HRMS** (ESI) m/z calcd. for $C_{16}H_{21}O_5S$ ($[M+H]^+$) 325.1104, found 325.1100; **FT-IR (thin film)** ν_{max} 3022, 2362, 1754, 1593, 1493, 1324, 1291, 1194, 1163, 1132, 1066, 927, 847, 752, 690, 667 cm^{-1}; **m.p.**: 89-90 °C; **$[\alpha]_D^{25}$** = +16.7 (c=1.11, $CHCl_3$); **HPLC:** Chiralcel AD-H, hexane/isopropanol = 85/15, 1.0 ml/min, λ = 254 nm, t_R (major) = 23.1 min, t_R (minor) = 27.2 min.

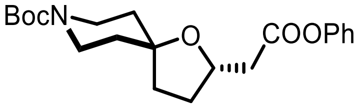

***tert*-Butyl (*S*)-2-(2-oxo-2-phenoxyethyl)-1-oxa-8-azaspiro[4.5]decane-8-carboxylate (4x)** was prepared following **General Procedure F**, (variation from standard conditions: reaction carried out in 0.2M 2-Me-THF for 2 days), using ***tert*-butyl (*E*)-4-hydroxy-4-(5-oxo-5-phenoxypent-3-en-1-yl)piperidine-1-carboxylate (3x)** (74.7 mg, 0.2 mmol, 1.0 eq.). Purification by silica gel chromatography (pentane/Et_2O = 1/1) afforded the title compound as a colorless oil (67.2 mg, 90%, 98.5:1.5 er).

^{1}H NMR (400 MHz, $CDCl_3$) δ 7.41 – 7.32 (m, 2H, Ar-H), 7.24 – 7.18 (m, 1H, Ar-H), 7.11 – 7.03 (m, 2H, Ar-H), 4.52 – 4.36 (m, 1H, CH), 3.61 (d, *J* = 13.5 Hz, 2H, SCH_2CH_2), 3.33 (ddt, *J* = 13.4, 9.9, 3.8 Hz, 2H, SCH_2CH_2), 2.81 (dd, *J* = 14.8, 7.0 Hz, 1H, CH_2C(O)O), 2.70 (dd, *J* = 14.8, 6.3 Hz, 1H, CH_2C(O)O), 2.28 – 2.13 (m, 1H, CH_2CH_2CH), 1.89 – 1.69 (m, 3H, CH_2CH_2CH and CH_2CH_2CH), 1.68 – 1.48 (m, 4H, SCH_2CH_2), 1.45 (s, 9H, C$(CH_3)_3$) ppm; **^{13}C NMR (101 MHz, $CDCl_3$)** δ 169.8 (C(O)O), 154.9 (C(O)O), 150.7 (ArC), 129.5 (ArCH), 125.9 (ArCH), 121.6 (ArCH), 80.7 (C), 79.4 (C), 74.7 (CH), 41.6 (CH_2C(O)O), 41.3 (brs, 2C, SCH_2CH_2), 37.8 (SCH_2CH_2), 36.6 (CH_2CH_2CH), 36.4 (SCH_2CH_2), 31.1 (CH_2CH_2CH), 28.5 (C$(CH_3)_3$) ppm; **HRMS** (ESI) m/z calcd. for $C_{21}H_{29}O_5NNa$ ($[M+Na]^+$) 398.1938, found 398.1934; **FT-IR (thin film)** ν_{max} 2937, 2872, 2360, 2341, 1758, 1692, 1594, 1422, 1365, 1278, 1194, 1150, 1070, 895, 822, 690, 658, 630 cm^{-1}; **$[\alpha]_D^{25}$** = 18.5 (c=0.40, $CHCl_3$); **HPLC:** Chiralcel AS-H, hexane/isopropanol = 98/2, 1.0 ml/min, λ = 210 nm, t_R (major) = 19.0 min, t_R (minor) = 44.9 min.

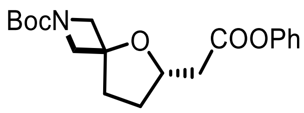

***tert*-butyl (*S*)-6-(2-oxo-2-phenoxyethyl)-5-oxa-2-azaspiro[3.4]octane-2-carboxylate (4y)** was prepared following **General Procedure F**, (variation from standard conditions: reaction carried out at –22 °C), using ***tert*-butyl (*E*)-3-hydroxy-3-(5-oxo-5-phenoxypent-3-en-1-yl)azetidine-1-carboxylate (3y)** (69.6 mg, 0.2 mmol, 1.0 eq.). Purification by silica gel chromatography (pentane/Et_2O = 1/1) afforded the title compound as a off-white solid (61.9 mg, 89%, 92.5:7.5 er).

^{1}H NMR (400 MHz, $CDCl_3$) δ 7.41 – 7.32 (m, 2H, Ar-H), 7.22 (ddt, J = 7.9, 6.9, 1.2 Hz, 1H, Ar-H), 7.12 – 7.04 (m, 2H, Ar-H), 4.47 (tt, *J* = 7.0, 6.0 Hz, 1H, CH), 4.04 (ddd, *J* = 8.9, 5.6, 1.1 Hz, 2H, NCH_2), 3.86 (td, *J* = 9.0, 1.1 Hz, 2H, NCH_2), 2.81 (dd, *J* = 15.4, 7.0 Hz, 1H, $CH_2C(O)O$), 2.71 (dd, *J* = 15.4, 6.2 Hz, 1H, $CH_2C(O)O$), 2.25 – 2.07 (m, 3H, CH_2CH_2), 1.80 – 1.69 (m, 1H, CH_2CH_2), 1.43 (s, 9H, $C(CH_3)_3$) ppm; **^{13}C NMR (101 MHz, $CDCl_3$)** δ 169.4 (C(O)O), 156.5 (C(O)O), 150.6 (ArC), 129.5 (ArCH), 126.0 (ArCH), 121.6 (ArCH), 79.6 (C), 78.4 (C), 75.9 (CH), 62.3 (NCH_2), 41.0 ($CH_2C(O)O$), 35.9 2 (CH_2CH_2), 30.8 2 (CH_2CH_2), 28.5 ($C(CH_3)_3$) ppm; **HRMS** (ESI) m/z calcd. for $C_{19}H_{26}O_5N$ ($[M+H]^+$) 348.1805, found 348.1799; **FT-IR (thin film)** ν_{max} 2976, 2877, 2359, 1759, 1699, 1594, 1493, 1403, 1367, 1194, 1163, 1085, 1024, 933, 902, 813, 753, 690, 667, 620 cm^{-1}; **m.p.**: 42-43 °C; **$[\alpha]_D^{25}$** = +13.5 (c=1.20, $CHCl_3$); **HPLC:** Chiralcel IB, hexane/isopropanol = 97/3, 1.0 ml/min, λ = 220 nm, t_R (major) = 24.3 min, t_R (minor) = 21.8 min.

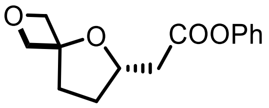

Phenyl (*S*)-2-(2,5-dioxaspiro[3.4]octan-6-yl)acetate (4z) was prepared following **General Procedure F**, (variation from standard conditions: reaction carried out in 0.2M 2-Me-THF at –22 °C for 10 hours), using **phenyl (*E*)-5-(3-hydroxyoxetan-3-yl)pent-2-enoate (3z)** (49.4 mg, 0.2 mmol, 1.0 eq.). Purification by silica gel chromatography (pentane/Et_2O = 3/7) afforded the title compound as a colorless oil (38.0 mg, 77%, 91:9 er).

^{1}H NMR (400 MHz, $CDCl_3$) δ 7.43 – 7.33 (m, 2H, Ar-H), 7.27 – 7.19 (m, 1H, Ar-H), 7.13 – 7.05 (m, 2H, Ar-H), 4.86 – 4.76 (m, 2H, OCH_2), 4.58 – 4.51 (m, 2H, OCH_2), 4.51 – 4.41 (m, 1H, CH), 2.80 (dd, *J* = 15.4, 6.9 Hz, 1H, CH_2C(O)O), 2.70 (dd, *J* = 15.4, 6.2 Hz, 1H, CH_2C(O)O), 2.40 – 2.29 (m, 1H, CH_2CH_2), 2.29 – 2.11 (m, 2H, CH_2CH_2), 1.80 – 1.65 (m, 1H, CH_2CH_2) ppm; **^{13}C NMR (101 MHz, $CDCl_3$)** δ 169.4 (C(O)O), 150.6 (ArC), 129.5 (ArCH), 126.0 (ArCH), 121.6 (ArCH), 84.4 (OCH_2), 83.9 (OCH_2), 82.6 (C), 75.9 (CH), 41.0 (CH_2C(O)O), 35.2 (CH_2CH_2), 30.9 (CH_2CH_2) ppm; **HRMS** (ESI) m/z calcd. for $C_{14}H_{17}O_4$ ($[M+H]^+$) 249.1121, found 249.1122; **FT-IR (thin film)** ν_{max} 2947, 2872, 1756, 1593, 1493, 1350, 1216, 1194, 1163, 1145, 1128, 1025, 971, 753, 689, 667 cm^{-1}; **$[\alpha]_D^{25}$** = +13.4 (c=0.69, $CHCl_3$); **HPLC:** Chiralcel AD, hexane/isopropanol = 98/2, 1.0 ml/min, λ = 210 nm, t_R (major) = 22.8 min, t_R (minor) = 24.8 min.

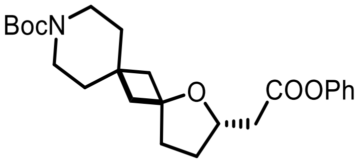

***tert*-Butyl (*S*)-2-(2-oxo-2-phenoxyethyl)-1-oxa-10-azadispiro[4.1.57.15]tridecane-10-carboxylate (4aa)** was prepared following **General Procedure F**, (variation from standard conditions: reaction carried out in 0.05M 2-Me-THF for 2 days), using ***tert*-butyl (*E*)-2-hydroxy-2-(5-oxo-5-phenoxypent-3-en-1-yl)-7-azaspiro[3.5]nonane-7-carboxylate (3aa)** (82.9 mg, 0.2 mmol, 1.0 eq.). Purification by silica gel (pentane/Et_2O = 1/1) afforded the title compound as a white solid (78.8 mg, 95%, 98:2 er).

^{1}H NMR (400 MHz, $CDCl_3$) δ 7.40 – 7.31 (m, 2H, Ar-H), 7.21 (ddt, *J* = 7.9, 6.9, 1.1 Hz, 1H, Ar-H), 7.11 – 7.04 (m, 2H, Ar-H), 4.40 (p, *J* = 6.8 Hz, 1H, CH), 3.35 – 3.24 (m, 4H, NCH_2CH_2), 2.82 (dd, *J* = 15.1, 6.7 Hz, 1H, $CH_2C(O)O$), 2.67 (dd, *J* = 15.1, 6.7 Hz, 1H, $CH_2C(O)O$), 2.15 (dddd, *J* = 12.2, 7.8, 6.7, 5.5 Hz, 1H, CH_2CH), 2.10 – 2.03 (m, 2H, CCH_2C), 2.02 – 1.87 (m, 4H, CH_2CH_2CH and CCH_2C), 1.70 (ddt, *J* = 12.3, 8.2, 7.3 Hz, 1H, CH_2CH), 1.61 – 1.54 (m, 2H, NCH_2CH_2), 1.53 – 1.47 (m, 2H, NCH_2CH_2), 1.44 (s, 9H, $C(CH_3)_3$) ppm; **^{13}C NMR (101 MHz, $CDCl_3$)** δ 169.7 (C(O)O), 155.1 (C(O)O), 150.7 (ArC), 129.5 (ArCH), 125.9 (ArCH), 121.6 (ArCH), 79.6 (C), 79.5 (C), 74.4 (CH), 45.7 (CCH_2C), 45.4 (CCH_2C), 41.5 ($CH_2C(O)O$), 41.1 (NCH_2CH_2), 40.9 (NCH_2CH_2), 39.2 (CH_2CH_2CH), 38.7 (NCH_2CH_2), 36.8 (NCH_2CH_2), 31.0 (CH_2CH), 29.5 (C), 28.5 ($C(CH_3)_3$) ppm; **HRMS** (ESI) m/z calcd. for $C_{24}H_{33}O_5NNa$ ($[M+Na]^+$) 438.2251, found 438.2249; **FT-IR (thin film)** ν_{max} 3015, 2916, 2843, 1759, 1691, 1594, 1493, 1422, 1365, 1271, 1243, 1194, 1163, 1147, 1063, 1026,896, 864, 754, 689, 671, 661, 625 cm^{-1}; **m.p.**: 71-72 °C; **$[\alpha]_D^{25}$** = +13.7 (c=0.23, $CHCl_3$); **HPLC:** Chiralcel AD-H, hexane/isopropanol = 90/10, 1.0 ml/min, λ = 210 nm, t_R (major) = 13.7 min, t_R (minor) = 12.0 min.

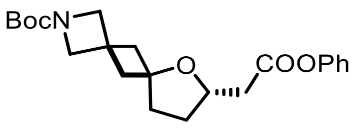

***tert*-Butyl (*S*)-8-(2-oxo-2-phenoxyethyl)-7-oxa-2-azadispiro[3.1.46.14]undecane-2-carboxylate (4ab)** was prepared following **General Procedure F**, (variation from standard conditions: reaction carried out in 0.05M 2-Me-THF), using ***tert*-butyl (*E*)-6-hydroxy-6-(5-oxo-5-phenoxypent-3-en-1-yl)-2-azaspiro[3.3]heptane-2-carboxylate (3ab)** (77.9 mg, 0.2 mmol, 1.0 eq.). Purification by silica gel chromatography (pentane/Et_2O = 1/1) afforded the title compound as a colorless oil (74.0 mg, 95%, 94.5:5.5 er).

^{1}H NMR (400 MHz, $CDCl_3$) δ 7.42 – 7.32 (m, 2H, Ar-H), 7.24 – 7.17 (m, 1H, Ar-H), 7.11 – 7.03 (m, 2H, Ar-H), 4.41 (p, *J* = 6.8 Hz, 1H, CH), 3.89 (d, *J* = 11.9 Hz, 4H, NCH_2 or CCH_2C), 2.78 (dd, *J* = 15.2, 6.9 Hz, 1H, $CH_2C(O)O$), 2.66 (dd, *J* = 15.2, 6.3 -Hz, 1H, $CH_2C(O)O$), 2.46 – 2.36 (m, 2H, NCH_2 or CCH_2C), 2.27 – 2.19 (m, 2H, NCH_2 or CCH_2C), 2.19 – 2.11 (m, 1H, CH_2CH), 1.96 – 1.81 (m, 2H, CH_2CH_2CH), 1.78 – 1.65 (m, 1H, CH_2CH), 1.42 (s, 9H, $C(CH_3)_3$) ppm; **^{13}C NMR (101 MHz, $CDCl_3$)** δ 169.6 (C(O)O), 156.2 (C(O)O), 150.7 (ArC), 129.5 (ArCH), 125.9 (ArCH), 121.6 (ArCH), 79.4 (C), 79.1 (C), 74.9 (CH), 61.8 (NCH_2 or CCH_2C), 60.4 (NCH_2 or CCH_2C), 47.0 (NCH_2 or CCH_2C), 46.4 (NCH_2 or CCH_2C), 41.5 ($CH_2C(O)O$), 36.8 (CH_2CH_2CH), 30.9 (CH_2CH), 29.5 (C), 28.5 ($C(CH_3)_3$) ppm; **HRMS** (ESI) m/z calcd. for $C_{22}H_{30}O_5N$ ($[M+H]^+$) 388.2118, found 388.2114; **FT-IR (thin film)** ν_{max} 2925, 2360, 1758, 1698, 1594, 1493, 1403, 1366, 1318, 1244, 1216, 1194, 1162, 1063, 931, 753, 690, 667 cm^{-1}; **$[\alpha]_D^{25}$** = +12.3 (c=1.34, $CHCl_3$); **HPLC:** Chiralcel AD-H, hexane/isopropanol = 90/10, 1.0 ml/min, λ = 220 nm, t_R (major) = 11.7 min, t_R (minor) = 13.3 min.

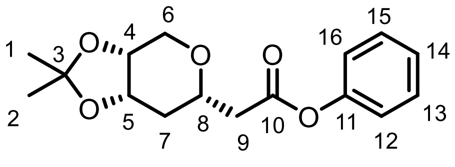

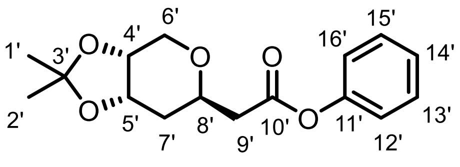

Phenyl 2-((3a*R*,6*S*,7a*S*)-2,2-dimethyltetrahydro-4H-[1,3]dioxolo[4,5-c]pyran-6-yl)acetate (A) (4ac) was prepared following **General Procedure F**, using **phenyl (*E*)-4-((4S,5R)-5-(hydroxymethyl)-2,2-dimethyl-1,3-dioxolan-4-yl)but-2-enoate (3ac)** (61.2 mg, 0.21 mmol, 1.0 eq). After 24 hours, the rest reaction mixture was filtered through a short silica pad to quench catalyst and analysed by NMR (Crude: 93%, **A**:**B** = 94:6 dr), which was then purified by silica gel chromatography (pentane/Et_2O = 13/7) affording the compound as a colorless oil (52 mg, 85%, **A**:**B** = >99.5:0.5 dr) and 8% of mixed diastereomers product (**A**:**B** = 35:65 dr).

Phenyl 2-((3a*R*,6*R*,7a*S*)-2,2-dimethyltetrahydro-4H-[1,3]dioxolo[4,5-c]pyran-6-yl)acetate (B) (S30) was prepared following **General Procedure F**, (variation from standard conditions: reaction carried out with 10% enantiomer of cat. **E**) using **phenyl (*E*)-4-((4S,5R)-5-(hydroxymethyl)-2,2-dimethyl-1,3-dioxolan-4-yl)but-2-enoate (3ac)** (61.2 mg, 0.21 mmol, 1.0 eq). After 24 hours, the rest reaction mixture was filtered through a short silica pad to quench catalyst and analysed by NMR (Crude: 99%, **A**:**B** = 30:70 dr), which was then purified by silica gel chromatography (pentane/Et_2O = 13/7) affording the compound as a colorless oil (45.9 mg, 75%, **A**:**B** = >0.5:99.5 dr) and 16% of mixed diastereomers product (**A**:**B** = 97:3 dr).

Reaction carried out with 10% BEMP under identical conditions afforded the compound (54% yield, **A**:**B** = 44:56 dr);

A (4ac): ^{1}H NMR (400 MHz, $CDCl_3$) δ 7.42 – 7.32 (m, 2H, 12-16), 7.25 – 7.18 (m, 1H, 12-16), 7.11 – 7.03 (m, 2H, 12-16), 4.36 – 4.23 (m, 2H, 5 and 6), 4.03 (ddd, *J* = 5.3, 2.6, 1.2 Hz, 1H, 4), 3.85 – 3.74 (m, 2H, 6 and 8), 2.86 (dd, *J* = 15.7, 8.0 Hz, 1H, 9), 2.66 (dd, *J* = 15.7, 5.1 Hz, 1H, 9), 2.03 (ddd, *J* = 13.1, 6.9, 2.1 Hz, 1H, 7), 1.63 (ddd, *J* = 13.2, 11.3, 9.6 Hz, 1H, 7), 1.55 (s, 3H, 1 or 2), 1.36 (s, 3H, 1 or 2) ppm; **^{13}C NMR (101 MHz, $CDCl_3$)** δ 169.5 (10), 150.7 (11), 129.5 (12-16), 126.0 (12-16), 121.6 (12-16), 109.2 (3), 71.52 (4), 71.48 (5), 71.1 (8), 66.7 (6), 41.0 (9), 35.3 (7), 28.5 (1 or 2), 26.5 (1 or 2) ppm; **HRMS** (ESI) m/z calcd. for $C_{16}H_{21}O_5$ ($[M+H]^+$) 293.1384, found 293.1384; **FT-IR (thin film)** ν_{max} 3409, 3016, 2923, 1756, 1593, 1492, 1397, 1320, 1243, 1194, 1162, 1139, 1089, 1066, 1007, 983, 937, 882, 816, 754, 714, 689, 661, 620 cm^{-1}; **$[\alpha]_D^{25}$** = −34.9 (c=0.87, $CHCl_3$).

B (S30): ^{1}H NMR (400 MHz, $CDCl_3$) δ 7.37 (t, *J* = 7.9 Hz, 2H, 12'-16'), 7.22 (t, *J* = 7.4 Hz, 1H, 12'-16'), 7.12 – 7.06 (m, 2H, 12'-16'), 4.44 – 4.36 (m, 1H, 5'), 4.15 (ddt, *J* = 11.5, 8.4, 3.9 Hz, 2H, 4' and 8'), 3.94 (dd, *J* = 11.6, 6.5 Hz, 1H, 6'), 3.42 (dd, *J* = 11.6, 9.4 Hz, 1H, 6'), 2.78 – 2.62 (m, 2H, 9'), 2.21 (dt, *J* = 14.9, 2.5 Hz, 1H, 7'), 1.85 (ddd, *J* = 15.2, 11.7, 3.9 Hz, 1H, 7'), 1.52 (s, 3H, 1' or 2'), 1.37 (s, 3H, 1' or 2') ppm; **^{13}C NMR (101 MHz, $CDCl_3$)** δ 169.4 (10'), 150.7 (11'), 129.5 (12'-16'), 126.0 (12'-16'), 121.7 (12'-16'), 109.2 (3'), 71.5 (5'), 70.0 (4' or 8'), 69.2 (4' or 8'), 67.9 (6'), 41.0 (9'), 32.5 (7'), 28.3 (1' or 2'), 26.3 (1' or 2') ppm; **HRMS** (ESI) m/z calcd. for $C_{16}H_{21}O_5$ ($[M+H]^+$) 293.1384, found 293.1384; **FT-IR (thin film)** ν_{max} 2988, 2937, 2360, 1758, 1593, 1493, 1457, 1427, 1380, 1217, 1195, 1164, 1143, 1061, 1022, 928, 896, 859, 820, 754, 716, 688, 668, 608 cm^{-1}; **$[\alpha]_D^{25}$** = +45.8 (c=0.92, $CHCl_3$).

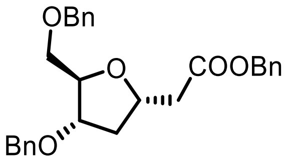

Benzyl 2-((2*S*,4*S*,5*R*)-4-(benzyloxy)-5-((benzyloxy)methyl)tetrahydrofuran-2-yl)acetate (4ad) was prepared following **General Procedure F**, (variation from standard conditions: reaction carried out with 10% enantiomer of cat. **E** in 0.1M 2-Me-THF), using **benzyl (5*S*,6*R*,*E*)-5,7-bis(benzyloxy)-6-hydroxyhept-2-enoate (3ad)** (87.9 mg, 0.2 mmol, 1.0 eq.). Purification by silica gel chromatography (pentane/Et_2O = 7/3) afforded the title compound as a colorless oil (82.6 mg, 94%, 97.5:2.5 dr).

Reaction carried out with 10% cat. **E** under identical conditions afforded the compound in 98% conversion, 64:36 dr.

Reaction carried out with 10% DBU under identical conditions afforded the compound in 99% conversion, 53.5:46.5 dr.

^{1}H NMR (400 MHz, $CDCl_3$) δ 7.43 – 7.28 (m, 15H, Ar-H), 5.18 (s, 2H, OCH_2Ar), 4.67 – 4.47 (m, 5H, $CHCH_2C(O)O$ and OCH_2Ar), 4.19 (td, *J* = 4.9, 2.6 Hz, 1H, BnOCHCH), 4.12 – 4.05 (m, 1H, BnOCH), 3.57 (dd, *J* = 10.2, 4.6 Hz, 1H, CH_2OBn), 3.47 (dd, *J* = 10.2, 5.4 Hz, 1H, CH_2OBn), 2.78 (dd, *J* = 15.4, 6.8 Hz, 1H, $CH_2C(O)O$), 2.60 (dd, *J* = 15.4, 6.2 Hz, 1H, $CH_2C(O)O$), 2.23 (ddd, *J* = 13.1, 5.3, 1.6 Hz, 1H, $CH_2CHCH_2C(O)O$), 1.81 – 1.69 (m, 1H, $CH_2CHCH_2C(O)O$) ppm; **^{13}C NMR (101 MHz, $CDCl_3$)** δ 170.8 (C(O)O), 138.3 (ArC), 138.2 (ArC), 136.0 (ArC), 128.6 (2C, ArCH), 128.45 (2C, ArCH), 128.42 (2C, ArCH), 128.3 (3C, ArCH), 127.69 (3C, ArCH), 127.65 (3C, ArCH), 83.7 (BnOCHCH), 81.2 (BnOCH), 75.0 ($CHCH_2C(O)O$), 73.5 (OCH_2Ar), 71.1 (OCH_2Ar), 71.0 (CH_2OBn), 66.4 (OCH_2Ar), 40.4 ($CH_2C(O)O$), 38.0 ($CH_2CHCH_2C(O)O$) ppm; **HRMS** (ESI) m/z calcd. for $C_{28}H_{31}O_5$ ($[M+H]^+$) 447.2166, found

447.2167; **FT-IR (thin film)** ν_{max} 3031, 2917, 1736, 1497, 1454, 1363, 1215, 1157, 1094, 1028, 995, 750, 697, 612 cm^{-1}; **$[\alpha]_D^{25}$** = +20.2 (c=1.57, $CHCl_3$).

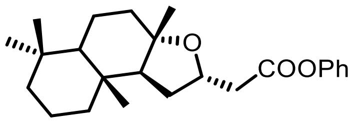

Phenyl 2-((2*S*,3a*S*,9a*R*,9b*S*)-3a,6,6,9a-tetramethyldodecahydronaphtho[2,1-b]furan-2-yl)acetate (4ae) was prepared following **General Procedure F**, using **phenyl (*E*)-4-((1*S*,2*S*,8a*R*)-2-hydroxy-2,5,5,8a-tetramethyldecahydronaphthalen-1-yl)but-2-enoate (3ae)** (73.6 mg, 0.2 mmol, 1.0 eq.). Purification by silica gel chromatography (pentane/Et_2O = 1/1) afforded the title compound as a white solid (67.7 mg, 92%, 99:1 dr).

Reaction carried out with 10% enantiomer of cat. **E** under identical conditions afforded the compound in 61% conversion, 60:40 dr.

Reaction carried out with 10% BEMP under identical conditions afforded the compound in 60% conversion, 1:1 dr.

^{1}H NMR (400 MHz, $CDCl_3$) δ 7.41 – 7.31 (m, 2H, Ar-H), 7.25 – 7.17 (m, 1H, Ar-H), 7.14 – 7.06 (m, 2H, Ar-H), 4.59 – 4.36 (m, 1H, CHCH$_2$C(O)O), 2.96 (dd, *J* = 15.2, 7.1 Hz, 1H, CH$_2$C(O)O), 2.74 (dd, *J* = 15.2, 7.1 Hz, 1H, CH$_2$C(O)O), 2.10 – 2.01 (m, 1H, OCHCH$_2$CH), 1.96 (dt, *J* = 11.6, 3.2 Hz, 1H, CH$_2$CH$_2$CHC(CH$_3$)$_2$), 1.87 – 1.71 (m, 1H, CH$_2$CH$_2$CHC(CH$_3$)$_2$), 1.70 – 1.56 (m, 3H, OCHCH$_2$CH, CH$_2$CH$_2$CH$_2$C(CH$_3$)$_2$ and OCHCH$_2$CH), 1.52 – 1.39 (m, 4H, 1H for CH$_2$CH$_2$CHC(CH$_3$)$_2$ and 3H for CH$_2$CH$_2$CH$_2$C(CH$_3$)$_2$), 1.37 – 1.26 (m, 1H, CH$_2$CH$_2$CHC(CH$_3$)$_2$), 1.19 (d, *J* = 0.9 Hz, 4H, CH$_3$ and

$C\underline{H}_2C\underline{H}_2C\underline{H}_2C(CH_3)_2$), 1.11 – 1.03 (m, 1H, $C\underline{H}_2C\underline{H}_2C\underline{H}_2C(CH_3)_2$), 0.98 (dd, *J* = 12.4, 2.7 Hz, 1H, $C\underline{H}C(CH_3)_2$), 0.88 (s, 3H, $C\underline{H}_3$), 0.87 (s, 3H, $C\underline{H}_3$), 0.84 (s, 3H, $C\underline{H}_3$) ppm; **^{13}C NMR (101 MHz, $CDCl_3$)** δ 169.8 ($\underline{C}$(O)O), 150.8 (Ar$\underline{C}$), 129.4 (Ar$\underline{C}$H), 125.8 (Ar$\underline{C}$H), 121.7 (Ar$\underline{C}$H), 81.2 ($\underline{C}$O), 74.4 ($\underline{C}HCH_2C(O)O$), 61.0 ($OCHCH_2\underline{C}H$), 57.2 ($\underline{C}HC(CH_3)_2$), 43.3 ($\underline{C}H_2C(O)O$), 42.5 ($\underline{C}H_2\underline{C}H_2\underline{C}H_2C(CH_3)_2$), 40.3 ($\underline{C}H_2CH_2CHC(CH_3)_2$), 40.1 ($\underline{C}H_2\underline{C}H_2\underline{C}H_2C(CH_3)_2$), 36.4 ($\underline{C}$), 33.6 ($\underline{C}H_3$), 33.2 ($\underline{C}$), 29.5 ($OCH\underline{C}H_2CH$), 24.8 ($\underline{C}H_3$), 21.2 ($\underline{C}H_3$), 20.9 ($CH_2\underline{C}H_2CHC(CH_3)_2$), 18.5 ($\underline{C}H_2\underline{C}H_2\underline{C}H_2C(CH_3)_2$), 15.6 ($\underline{C}H_3$) ppm; **HRMS** (ESI) m/z calcd. for $C_{24}H_{35}O_3$ ($[M+H]^+$) 371.2581, found 371.2581; **FT-IR (thin film)** ν_{max} 2999, 2924, 2867, 2360, 2342, 1759, 1594, 1493, 1458, 1379, 1334, 1299, 1274, 1220, 1195, 1163, 1119, 1080, 1068, 1042, 1026, 1003, 985, 962, 937, 900, 814, 752, 711, 689, 668, 628 cm^{-1}; **m.p.**: 55-56 °C; **$[\alpha]_D^{25}$** = −7.5 (c=1.24, $CHCl_3$).

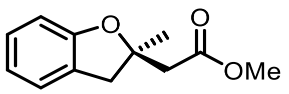

Methyl (*R*)-2-(2-methyl-2,3-dihydrobenzofuran-2-yl)acetate (4af) was prepared following **General Procedure G**, using **methyl (*E*)-4-(2-hydroxyphenyl)-3-methylbut-2-enoate (3af)** (38.1 mg, 0.19 mmol, 1.0 eq.). Purification by silica gel chromatography (pentane/Et_2O = 95/5) afforded the title compound as a colorless oil (34.3 mg, 90%, 95:5 er). Data is consistent with the published literature.[9]

^{1}H NMR (400 MHz, $CDCl_3$) δ 7.18 – 7.06 (m, 2H, Ar-H), 6.84 (td, *J* = 7.4, 1.0 Hz, 1H, Ar-H), 6.75 (d, *J* = 8.0 Hz, 1H, Ar-H), 3.66 (s, 3H, OCH_3), 3.37 (d, *J* = 15.8 Hz, 1H, $CH_2C(O)O$), 3.05 (dd, *J* = 15.9, 1.2 Hz, 1H, $CH_2C(O)O$), 2.78 (d, *J* = 2.5 Hz, 2H, $ArCH_2$), 1.58 (s, 3H, CCH_3) ppm; **^{13}C NMR (101 MHz, $CDCl_3$)** δ 170.8 (C(O)O), 158.4 (ArCO), 128.2 (ArCH), 126.7 (ArCC), 125.3 (ArCH), 120.5 (ArCH), 109.7 (ArCH), 86.1(CH_2C), 51.8 (OCH_3), 45.0 ($ArCH_2$), 41.4 ($CH_2C(O)O$), 26.5 (CCH_3) ppm; **HRMS** (ESI) m/z calcd. for $C_{12}H_{15}O_3$ ($[M+H]^+$) 207.1016, found 207.1017; **HPLC:** Chiralcel OD, hexane/isopropanol = 97/3, 1.0 ml/min, λ = 220 nm, t_R (major) = 6.8 min, t_R (minor) = 11.0 min.

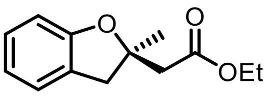

Ethyl (*R*)-2-(2-methyl-2,3-dihydrobenzofuran-2-yl)acetate (4ag) was prepared following **General Procedure F**, (variation from standard conditions: reaction carried out for 17h), using **ethyl (*E*)-4-(2-hydroxyphenyl)-3-methylbut-2-enoate (3ag)** (44.2 mg, 0.2 mmol, 1.0 eq.). Purification by silica gel chromatography (pentane/Et_2O = 9/1) afforded the title compound as a colorless oil (43.9 mg, 99%, 97.5:2.5 er).

^{1}H NMR (400 MHz, $CDCl_3$) δ 7.18 – 7.06 (m, 2H, Ar-H), 6.83 (td, *J* = 7.4, 0.9 Hz, 1H, Ar-H), 6.75 (d, *J* = 8.0 Hz, 1H, Ar-H), 4.17 – 4.05 (m, 2H, OCH_2CH_3), 3.39 (d, *J* = 15.8 Hz, 1H, $CH_2C(O)O$), 3.04 (d, *J* = 15.8 Hz, 1H, $CH_2C(O)O$), 2.83 – 2.70 (m, 2H, $ArCH_2$), 1.58 (s, 3H, CCH_3), 1.21 (t, *J* = 7.1 Hz, 3H, OCH_2CH_3) ppm; **^{13}C NMR (101 MHz, $CDCl_3$)** δ 170.3 (C(O)O), 158.5 (ArCO), 128.2 (ArCH), 126.7 (ArCC), 125.2 (ArCH), 120.5 (ArCH), 109.7 (ArCH), 86.2(CH_2C), 60.6 (OCH_2CH_3), 45.4 ($ArCH_2$), 41.3 ($CH_2C(O)O$), 26.7 (CCH_3), 14.2 ((OCH_2CH_3)) ppm; **HRMS** (ESI) m/z calcd. for $C_{13}H_{17}O_3$ ($[M+H]^+$) 221.1172, found 221.1174; **FT-IR (thin film)** ν_{max} 2980, 2360, 1732, 1598, 1480, 1460, 1372, 1242, 1065, 1031, 888, 752, 668 cm^{-1}; **$[\alpha]_D^{25}$** = +4.6 (c=0.68, $CHCl_3$); **HPLC:** Chiralcel OD, hexane/isopropanol = 98/2, 1.0 ml/min, λ = 230 nm, t_R (major) = 6.9 min, t_R (minor) = 10.2 min.

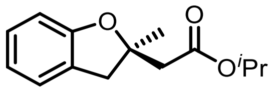

***iso*-Propyl (*R*)-2-(2-methyl-2,3-dihydrobenzofuran-2-yl)acetate (4ah)** was prepared following **General Procedure G**, (variation from standard conditions: reaction carried out for 17h), using ***iso*-propyl (*E*)-4-(2-hydroxyphenyl)-3-methylbut-2-enoate (3ah)** (46.7 mg, 0.2 mmol, 1.0 eq.). Purification by silica gel chromatography (pentane/Et_2O = 9/1) afforded the title compound as a colorless oil (43.9 mg, 94%, 98:2 er).

^{1}H NMR (400 MHz, $CDCl_3$) δ 7.17 – 7.06 (m, 2H, Ar-H), 6.83 (td, *J* = 7.4, 1.0 Hz, 1H, Ar-H), 6.74 (d, *J* = 8.0 Hz, 1H, Ar-H), 4.99 (hept, *J* = 6.3 Hz, 1H, OCH), 3.41 (d, *J* = 15.8 Hz, 1H, CH_2C(O)O), 3.03 (d, *J* = 15.8 Hz, 1H, CH_2C(O)O), 2.81 – 2.66 (m, 2H, ArCH_2), 1.57 (s, 3H, CCH_3), 1.19 (dd, *J* = 8.4, 6.3 Hz, 6H, OCH(CH_3)$_2$) ppm; **^{13}C NMR (101 MHz, $CDCl_3$)** δ 169.8 (C(O)O), 158.6 (ArCO), 128.1 (ArCH), 126.8 (ArCC), 125.2 (ArCH), 120.4 (ArCH), 109.7 (ArCH), 86.2 (CH_2C), 68.1 (OCH), 45.8 (ArCH_2), 41.3 (CH_2C(O)O), 26.9 (CCH_3), 21.83 (OCH(CH_3)$_2$), 21.81 (OCH(CH_3)$_2$) ppm; **HRMS** (ESI) m/z calcd. for $C_{14}H_{19}O_3$ ($[M+H]^+$) 235.1329, found 235.1330; **FT-IR (thin film)** ν_{max} 3648, 2980, 2360, 2341, 1728, 1598, 1481, 1460, 1375, 1327, 1243, 1146, 1106, 1065, 1016, 966, 885, 752, 668 cm^{-1}; **$[\alpha]_D^{25}$** = +3.0 (c=0.80, $CHCl_3$); **HPLC:** Chiralcel IA, hexane/isopropanol = 99.5/0.5, 1.0 ml/min, λ = 220 nm, t_R (major) = 6.4 min, t_R (minor) = 5.8 min.

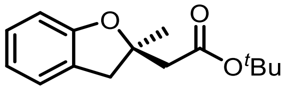

***tert*-Butyl (*R*)-2-(2-methyl-2,3-dihydrobenzofuran-2-yl)acetate (4ai)** was prepared following **General Procedure G**, (variation from standard conditions: reaction carried out for 17h), using ***tert*-butyl (*E*)-4-(2-hydroxyphenyl)-3-methylbut-2-enoate (3ai)** (49.4 mg, 0.2 mmol, 1.0 eq.). Purification by silica gel chromatography (pentane/Et_2O = 95/5) afforded the title compound as a colorless oil (45.9 mg, 93%, 99.5:0.5 er).

^{1}H NMR (400 MHz, $CDCl_3$) δ 7.17 – 7.05 (m, 2H, Ar-H), 6.83 (td, *J* = 7.5, 1.1 Hz, 1H, Ar-H), 6.74 (d, *J* = 7.9 Hz, 1H, Ar-H), 3.42 (dd, *J* = 15.8, 1.0 Hz, 1H, CH_2C(O)O), 3.02 (dd, *J* = 15.8, 1.1 Hz, 1H, CH_2C(O)O), 2.72 (d, *J* = 14.3 Hz, 1H, ArCH_2), 2.64 (d, *J* = 14.3 Hz, 1H, ArCH_2), 1.56 (s, 3H, CCH_3), 1.38 (s, 9H, OC(CH_3)$_3$) ppm; **^{13}C NMR (101 MHz, $CDCl_3$)** δ 169.6 (C(O)O), 158.7 (ArCO), 128.1 (ArCH), 126.9 (ArCC), 125.2 (ArCH), 120.4 (ArCH), 109.6 (ArCH), 86.3 (CH_2C), 81.0 (OC(CH_3)$_3$), 46.8 (ArCH_2), 41.2 (CH_2C(O)O), 28.1 (OC(CH_3)$_3$), 27.0 (CCH_3) ppm; **HRMS** (ESI) m/z calcd. for $C_{15}H_{20}O_3Na$ ($[M+Na]^+$) 271.1305, found 271.1306; **FT-IR (thin film)** ν_{max} 3649, 2980, 2360, 2341, 1727, 1598, 1481, 1460, 1368, 1244, 1160, 1112, 1065, 1016, 956, 888, 866, 842, 751, 708, 668 cm^{-1}; **$[\alpha]_D^{25}$** = +4.3 (c=0.73, $CHCl_3$); **HPLC:** Chiralcel IA, hexane/isopropanol = 99.5/0.5, 1.0 ml/min, λ = 210 nm, t_R (major) = 10.1 min, t_R (minor) = 8.6 min.

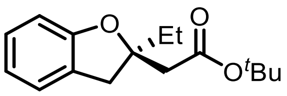

***tert*-Butyl (*R*)-2-(2-ethyl-2,3-dihydrobenzofuran-2-yl)acetate (4aj)** was prepared following **General Procedure G**, using ***tert*-butyl (*E*)-3-(2-hydroxybenzyl)pent-2-enoate (3aj)** (52.3 mg, 0.2 mmol, 1.0 eq.). Purification by silica gel chromatography (pentane/Et_2O = 95/5) afforded the title compound as a colorless oil (47.6 mg, 91%, 99.5:0.5 er).

^{1}H NMR (400 MHz, $CDCl_3$) δ 7.16 – 7.04 (m, 2H, Ar-H), 6.81 (td, *J* = 7.4, 1.0 Hz, 1H, Ar-H), 6.77 – 6.70 (m, 1H, Ar-H), 3.38 (dd, *J* = 15.9, 1.1 Hz, 1H, CH_2C(O)O), 3.06 (dt, *J* = 16.1, 1.1 Hz, 1H, CH_2C(O)O), 2.73 – 2.59 (m, 2H, ArCH_2), 1.86 (q, *J* = 7.1 Hz, 2H, CCH_2CH$_3$), 1.34 (s, 9H, OC(CH_3)$_3$), 0.98 (t, *J* = 7.4 Hz, 3H, CCH$_2$CH_3) ppm; **^{13}C NMR (101 MHz, $CDCl_3$)** δ 169.7 (C(O)O), 159.1 (ArCO), 128.0 (ArCH), 127.0 (ArCC), 125.1 (ArCH), 120.2 (ArCH), 109.4 (ArCH), 88.7 (CH$_2$C), 80.9 (OC(CH$_3$)$_3$), 44.8 (ArCH$_2$), 38.9 (CH$_2$C(O)O), 32.8 (CCH$_2$CH$_3$), 28.0 (OC(CH$_3$)$_3$), 8.0 (CCH$_2$CH$_3$) ppm; **HRMS** (ESI) m/z calcd. for $C_{16}H_{22}O_3Na$ ([M+Na]$^+$) 285.1461, found 285.1461; **FT-IR (thin film)** ν_{max} 3013, 2975, 2930, 1727, 1598, 1482, 1461, 1392, 1368, 1326, 1295, 1246, 1160, 1086, 1016, 1003, 950, 889, 869, 843, 749, 708, 652, 633, 610 cm^{-1}; **$[\alpha]_D^{25}$** = +17.3 (c=0.81, $CHCl_3$); **HPLC:** Chiralcel AD, hexane/isopropanol = 99.5/0.5, 1.0 ml/min, λ = 230 nm, t_R (major) = 5.3 min, t_R (minor) = 4.7 min.

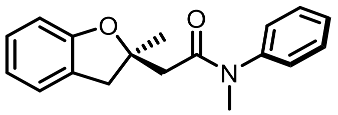

(*R*)-*N*-methyl-2-(2-methyl-2,3-dihydrobenzofuran-2-yl)-*N*-phenylacetamide (4ak) was prepared following **General Procedure G**, (variation from standard conditions: reaction carried out at 40 °C for 4 days), using **(*E*)-4-(2-hydroxyphenyl)-*N*,3-dimethyl-*N*-phenylbut-2-enamide (3ak)** (56.3 mg, 0.2 mmol, 1.0 eq.). Purification by silica gel chromatography (pentane/Et_2O = 7/3) afforded the title compound as a pale-yellow oil (47.3 mg, 84%, 99:1 er).

^{1}H NMR (400 MHz, $CDCl_3$) δ 7.39 (dd, *J* = 8.3, 6.7 Hz, 2H, Ar-H), 7.32 (t, *J* = 7.4 Hz, 1H, Ar-H), 7.21 – 7.10 (m, 3H, Ar-H), 7.06 (t, *J* = 7.7 Hz, 1H, Ar-H), 6.80 (t, *J* = 7.4 Hz, 1H, Ar-H), 6.65 (d, *J* = 7.9 Hz, 1H, Ar-H), 3.47 (d, *J* = 16.1 Hz, 1H, $C\underline{H}_2C(O)N$), 3.27 (s, 3H, $NC\underline{H}_3$), 3.09 (d, *J* = 16.1 Hz, 1H, $C\underline{H}_2C(O)N$), 2.68 – 2.53 (m, 2H, $ArC\underline{H}_2$), 1.53 (s, 3H, $CC\underline{H}_3$) ppm; **^{13}C NMR (101 MHz, $CDCl_3$)** δ 169.8 ($\underline{C}(O)O$), 158.1 (Ar$\underline{C}$O), 143.9 (Ar$\underline{C}$N), 130.0 (Ar$\underline{C}$H), 128.0 (Ar$\underline{C}$H), 127.9 (Ar$\underline{C}$H), 127.6 (Ar$\underline{C}$H), 127.3 (Ar$\underline{C}$C), 125.3 (Ar$\underline{C}$H), 120.3 (Ar$\underline{C}$H), 109.4 (Ar$\underline{C}$H), 87.8 ($CH_2\underline{C}$), 44.0 ($Ar\underline{C}H_2$), 41.7 ($\underline{C}H_2C(O)N$), 37.4 ($N\underline{C}H_3$), 26.5 ($C\underline{C}H_3$) ppm; **HRMS** (ESI) m/z calcd. for $C_{18}H_{20}O_2N$ ($[M+H]^+$) 282.1489, found 282.1488; **FT-IR (thin film)** ν_{max} 2980, 2360, 1651, 1596, 1496, 1480, 1459, 1387, 1239, 1217, 1113, 1069, 884, 751, 700, 667 cm^{-1}; **$[\alpha]_D^{25}$** = +81.9 (c=0.87, $CHCl_3$); **HPLC:** Chiralcel AD-H, hexane/isopropanol = 93/7, 1.0 ml/min, λ = 230 nm, t_R (major) = 10.4 min, t_R (minor) = 8.6 min.

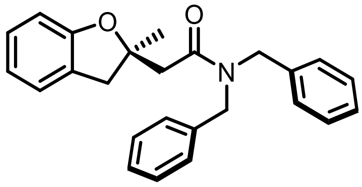

(*R*)-*N*,*N*-dibenzyl-2-(2-methyl-2,3-dihydrobenzofuran-2-yl)acetamide (4al) was prepared following **General Procedure G**, (variation from standard conditions: reaction carried out at 40 °C for 4 days), using **(*E*)-*N*,*N*-dibenzyl-4-(2-hydroxyphenyl)-3-methylbut-2-enamide (3al)** (74.2 mg, 0.2 mmol, 1.0 eq.). Purification by silica gel chromatography (pentane/Et_2O = 7/3) afforded the title compound as a colorless oil (49.7 mg, 67%, 98.5:1.5 er).

^{1}H NMR (400 MHz, $CDCl_3$) δ 7.49 – 6.97 (m, 12H, Ar-H), 6.91 (t, *J* = 7.4 Hz, 1H, Ar-H), 6.72 (d, *J* = 7.9 Hz, 1H, Ar-H), 4.85 (d, *J* = 15.0 Hz, 1H, NCH_2), 4.73 (t, *J* = 16.1 Hz, 1H, NCH_2), 4.42 (dd, *J* = 15.9, 3.7 Hz, 2H, NCH_2), 3.68 (d, *J* = 16.1 Hz, 1H, $CH_2C(O)N$), 3.22 – 3.13 (m, 1H, $CH_2C(O)N$), 3.02 (d, *J* = 14.7 Hz, 1H, $ArCH_2$), 2.93 (d, *J* = 14.7 Hz, 1H, $ArCH_2$), 1.66 (s, 3H, CCH_3) ppm; **^{13}C NMR (101 MHz, $CDCl_3$)** δ 170.6 (C(O)N), 158.2 (ArCO), 137.1 ($ArCCH_2N$), 136.6 ($ArCCH_2N$), 129.1 (ArCH), 128.7 (ArCH), 128.1 (ArCH), 127.9 (ArCH), 127.7 (ArCH), 127.3 (ArCH), 127.1 ($ArCCH_2C$), 126.5 (ArCH), 125.5 (ArCH), 120.6 (ArCH), 109.5 (ArCH), 87.7 (CH_2C), 50.4 (NCH_2), 48.2 (NCH_2), 43.7 ($ArCH_2$), 41.2 ($CH_2C(O)N$), 27.5 (CCH_3) ppm; **HRMS** (ESI) m/z calcd. for $C_{25}H_{26}O_2N$ ($[M+H]^+$) 372.1958, found 372.1953; **FT-IR (thin film)** ν_{max} 3029, 1639, 1480, 1451, 1361, 1328, 1242, 1080, 1029, 958, 884, 751, 699, 655, 619 cm^{-1}; **$[\alpha]_D^{25}$** = +57.1 (c=0.83, $CHCl_3$); **HPLC:** Chiralcel AS-H, hexane/isopropanol = 97/3, 1.0 ml/min, λ = 230 nm, t_R (major) = 20.8 min, t_R (minor) = 17.9 min.

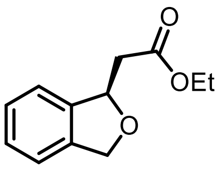

Ethyl (*R*)-2-(1,3-dihydroisobenzofuran-1-yl)acetate (4am) was prepared following **General Procedure H**, using **ethyl (*E*)-3-(2-(hydroxymethyl)phenyl)acrylate (3am)** (41.3 mg, 0.2 mmol, 1.0 eq.). Purification by silica gel chromatography (pentane/Et_2O = 4/1) afforded the title compound as a colorless oil (35.1 mg, 85%, 95:5 er). Data is consistent with the published literature.[32]

^{1}H NMR (400 MHz, $CDCl_3$) δ 7.33 – 7.12 (m, 4H, Ar-H), 5.72 – 5.55 (m, 1H, OCH), 5.13 (ddd, J = 12.2, 2.6, 0.8 Hz, 1H, $CH_2C(O)O$), 5.09 – 5.00 (m, 1H, $CH_2C(O)O$), 4.18 (q, *J* = 7.1 Hz, 2H, OCH_2CH_3), 2.83 – 2.66 (m, 2H, $CHOCH_2$), 1.25 (t, *J* = 7.1 Hz, 3H, OCH_2CH_3) ppm; **^{13}C NMR (101 MHz, $CDCl_3$)** δ 171.0 (C(O)O), 140.8 (ArC), 139.3 (ArC), 128.0 (ArCH), 127.5 (ArCH), 121.3 (ArCH), 121.2 (ArCH), 80.5 (OCH), 72.8 ($CH_2C(O)O$), 60.8 (OCH_2CH_3), 41.8 ($CHOCH_2$), 14.3 (OCH_2CH_3) ppm; **$[\alpha]_D^{25}$** = +32.7 (c=0.24, $CHCl_3$); **HPLC:** Chiralcel IA, hexane/isopropanol = 98/2, 1.0 ml/min, λ = 210 nm, t_R (major) = 9.5 min, t_R (minor) = 10.7 min.

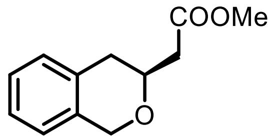

Methyl (*S*)-2-(isochroman-3-yl)acetate (4an) was prepared following **General Procedure H**, (variation from standard conditions: reaction carried out in 2-Me THF (0.5M) with 10% cat. **K** for 3 days), using **methyl (*E*)-4-(2-(hydroxymethyl)phenyl)but-2-enoate (3an)** (41.1 mg, 0.2 mmol, 1.0 eq.). Purification by silica gel chromatography (pentane/Et_2O = 17/3) afforded the title compound as a pale-yellow oil (38.6 mg, 94%, 92:8 er).

^{1}H NMR (400 MHz, $CDCl_3$) δ 7.21 – 7.13 (m, 2H, Ar-H), 7.10 (dd, *J* = 5.4, 3.6 Hz, 1H, Ar-H), 7.00 (dd, *J* = 5.4, 3.6 Hz, 1H, Ar-H), 4.84 (s, 2H, ArCH_2O), 4.17 (tdd, *J* = 7.7, 6.5, 5.2 Hz, 1H, CH), 3.74 (s, 3H, CH_3), 2.80 (d, *J* = 7.0 Hz, 2H, ArCH_2CH), 2.72 (dd, *J* = 15.4, 7.8 Hz, 1H, CH_2C(O)O), 2.60 (dd, *J* = 15.4, 5.1 Hz, 1H, CH_2C(O)O) ppm; **^{13}C NMR (101 MHz, $CDCl_3$)** δ 171.6 (C(O)O), 134.4 (ArC), 132.7 (ArC), 128.9 (ArCH), 126.6 (ArCH), 126.3 (ArCH), 124.3 (ArCH), 71.6 (CH), 68.4 (ArCH_2O), 51.9 (CH_3), 41.0 (CH_2C(O)O), 33.7 (ArCH_2CH) ppm; **HRMS** (ESI) m/z calcd. for $C_{12}H_{15}O_3$ ($[M+H]^+$) 207.1016, found 207.1017; **FT-IR (thin film)** ν_{max} 3023, 2952, 1738, 1493, 1437, 1374, 1344, 1300, 1246, 1217, 1160, 1098, 1060, 1037, 994, 863, 753, 672, 643, 632, 611 cm^{-1}; **$[\alpha]_D^{25}$** = −92.5 (c=0.71, $CHCl_3$); **HPLC:** Chiralcel IB, hexane/isopropanol = 97/3, 1.0 ml/min, λ = 210 nm, t_R (major) = 18.6 min, t_R (minor) = 7.9 min.

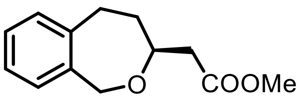

Methyl (*S*)-2-(1,3,4,5-tetrahydrobenzo[c]oxepin-3-yl)acetate (4ao) was prepared following **General Procedure H**, (variation from standard conditions: reaction carried out in 2-Me THF (0.5M) with 10% cat. **K** at 60 °C for 3 days), using **methyl (*E*)-5-(2-(hydroxymethyl)phenyl)pent-2-enoate (3ao)** (43.2 mg, 0.2 mmol, 1.0 eq.). Purification by silica gel chromatography (pentane/Et_2O = 17/3) afforded the title compound as a colorless oil (12.1 mg, 28%, 91:9 er).

^{1}H NMR (400 MHz, $CDCl_3$) δ 7.24 – 7.10 (m, 4H, Ar-$\underline{H}$), 4.69 (d, *J* = 3.5 Hz, 2H, $ArC\underline{H}_2O$), 4.23 (tdd, *J* = 10.3, 5.0, 2.0 Hz, 1H, $C\underline{H}$), 3.71 (s, 3H, $C\underline{H}_3$), 3.12 (ddd, *J* = 14.4, 12.1, 2.0 Hz, 1H, $CHCH_2C\underline{H}_2$), 2.88 (ddd, *J* = 15.0, 6.8, 1.9 Hz, 1H, $CHCH_2C\underline{H}_2$), 2.60 (dd, *J* = 15.3, 8.1 Hz, 1H, $C\underline{H}_2C(O)O$), 2.44 (dd, *J* = 15.3, 5.0 Hz, 1H, $C\underline{H}_2C(O)O$), 1.95 (ddt, *J* = 14.0, 6.8, 2.0 Hz, 1H, $CHC\underline{H}_2CH_2$), 1.60 (dddd, *J* = 14.1, 12.3, 10.6, 1.9 Hz, 1H, $CHC\underline{H}_2CH_2$) ppm; **^{13}C NMR (101 MHz, $CDCl_3$)** δ 171.9 ($\underline{C}(O)O$), 142.0 ($Ar\underline{C}$), 139.9 ($Ar\underline{C}$), 129.2 ($Ar\underline{C}H$), 128.6 ($Ar\underline{C}H$), 128.1 ($Ar\underline{C}H$), 126.4 ($Ar\underline{C}H$), 81.7 ($\underline{C}H$), 74.0 ($Ar\underline{C}H_2O$), 51.8 ($\underline{C}H_3$), 42.0 ($C\underline{H}_2C(O)O$), 34.8 ($CH\underline{C}H_2CH_2$), 34.3 ($CHCH_2\underline{C}H_2$) ppm; **HRMS** (ESI) m/z calcd. for $C_{13}H_{17}O_3$ ($[M+H]^+$) 221.1172, found 221.1174; **FT-IR (thin film)** ν_{max} 2951, 2848, 1738, 1437, 1361, 1282, 1203, 1150, 1121, 1086, 1063, 1028, 755, 671, 625 cm^{-1}; **$[\alpha]_D^{25}$** = +1.6 (c=0.21, $CHCl_3$); **HPLC:** Chiralcel IB, hexane/isopropanol = 98/2, 1.0 ml/min, λ = 220 nm, t_R (major) = 14.2 min, t_R (minor) = 9.4 min.

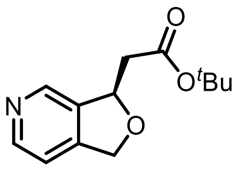

***tert*-Butyl (*R*)-2-(1,3-dihydrofuro[3,4-c]pyridin-3-yl)acetate (4ap)** was prepared following **General Procedure H**, (variation from standard conditions: reaction carried out at 50 °C for 3 days), using ***tert*-butyl (*E*)-3-(4-(hydroxymethyl)pyridin-3-yl)acrylate (3ap)** (47.3 mg, 0.2 mmol, 1.0 eq.). Purification by silica gel chromatography (CH_2Cl_2/CH_3OH = 97/3) afforded the title compound as a colorless oil (25.5 mg, 54%, 80:20 er).

^{1}H NMR (400 MHz, $CDCl_3$) δ 8.51 – 8.45 (m, 2H, Ar-H), 7.16 (dd, *J* = 5.0, 1.1 Hz, 1H, Ar-H), 5.62 (tt, *J* = 6.3, 2.1 Hz, 1H, CH), 5.09 (ddd, *J* = 13.7, 2.6, 1.0 Hz, 1H, CH_2), 5.00 (ddd, *J* = 13.7, 1.6, 0.9 Hz, 1H, CH_2), 2.70 (d, *J* = 6.2 Hz, 2H, CH_2), 1.40 (s, 9H, $C(CH_3)_3$) ppm; **^{13}C NMR (101 MHz, $CDCl_3$)** δ 169.5 (C(O)O), 148.9 (ArC), 148.7 (ArCH), 143.4 (ArCH), 137.2 (ArC), 116.5 (ArCH), 81.2 ($C(CH_3)_3$), 79.2 (CH), 72.1 (CH_2), 42.4 (CH_2), 28.1 ($C(CH_3)_3$) ppm; **HRMS** (ESI) m/z calcd. for $C_{13}H_{18}O_3N$ ($[M+H]^+$) 236.1281, found 236.1282; **FT-IR (thin film)** ν_{max} 2966, 2360, 1780, 1727, 1592, 1457, 1422, 1394, 1368, 1307, 1279, 1250, 1219, 1151, 1071, 1029, 1003, 844, 756, 695, 660, 609 cm^{-1}; **$[\alpha]_D^{25}$** = +5.3 (c=0.49, $CHCl_3$); **HPLC:** Chiralcel OD, hexane/isopropanol = 97/3, 1.0 ml/min, λ = 210 nm, t_R (major) = 31.4 min, t_R (minor) = 23.2 min.

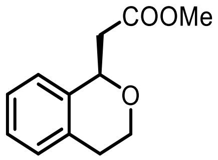

Methyl (*R*)-2-(isochroman-1-yl)acetate (4aq) was prepared following **General Procedure H**, (variation from standard conditions: reaction carried out in 2-Me THF (0.5M) with 10% cat. **K** at 50 °C for 3 days), using **methyl (*E*)-3-(2-(2-hydroxyethyl)phenyl)acrylate (3aq)** (41.2 mg, 0.2 mmol, 1.0 eq.). Purification by silica gel chromatography (pentane/Et_2O = 4/1) afforded the title compound as a colorless oil (33.8 mg, 82%, 90:10 er). Data is consistent with the published literature.[37]

^{1}H NMR (400 MHz, $CDCl_3$) δ 7.23 – 7.15 (m, 2H, Ar-$\underline{H}$), 7.15 – 7.09 (m, 1H, Ar-$\underline{H}$), 7.08 – 7.01 (m, 1H, Ar-$\underline{H}$), 5.25 (dd, *J* = 9.6, 3.5 Hz, 1H, C$\underline{H}$), 4.13 (ddd, *J* = 11.5, 5.3, 4.2 Hz, 1H, $OC\underline{H}_2CH_2$), 3.82 (ddd, *J* = 11.4, 9.1, 4.0 Hz, 1H, $OC\underline{H}_2CH_2$), 3.77 (s, 3H, $C\underline{H}_3$), 3.05 – 2.93 (m, 1H, $OCH_2C\underline{H}_2$), 2.90 (dd, *J* = 15.2, 3.5 Hz, 1H, $C\underline{H}_2C(O)O$), 2.82 – 2.68 (m, 2H, $C\underline{H}_2C(O)O$ and $OCH_2C\underline{H}_2$) ppm; **^{13}C NMR (101 MHz, $CDCl_3$)** δ 171.8, ($\underline{C}$(O)O), 136.8 (Ar$\underline{C}$), 134.0 (Ar$\underline{C}$), 129.2 (Ar$\underline{C}$H), 126.8 (Ar$\underline{C}$H), 126.4 (Ar$\underline{C}$H), 124.6 (Ar$\underline{C}$H), 73.0 ($\underline{C}$H), 63.2 ($O\underline{C}H_2CH_2$), 52.0 ($\underline{C}H_3$), 41.7 ($\underline{C}H_2C(O)O$), 28.9 ($OCH_2\underline{C}H_2$) ppm; **HRMS** (ESI) m/z calcd. for $C_{12}H_{15}O_3$ ($[M+H]^+$) 207.1016, found 207.1018; **$[\alpha]_D^{25}$** = +93.8 (c=0.36, $CHCl_3$); **HPLC:** Chiralcel OD, hexane/isopropanol = 98/2, 1.0 ml/min, λ = 210 nm, t_R (major) = 23.0 min, t_R (minor) = 19.4 min.

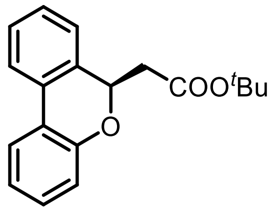

***tert*-Butyl (*R*)-2-(6H-benzo[c]chromen-6-yl)acetate (4ar)** was prepared following **General Procedure H**, (variation from standard conditions: reaction carried out with 10% cat. **K** in 0.025M 2-Me-THF at –22 °C), using ***tert*-butyl (*E*)-3-(2'-hydroxy-[1,1'-biphenyl]-2-yl)acrylate (3ar)** (59.2 mg, 0.2 mmol, 1.0 eq.). Purification by silica gel chromatography (pentane/Et_2O = 9/1) afforded the title compound as a white solid (57.4 mg, 97%, 87.5:12.5 er).

^{1}H NMR (400 MHz, $CDCl_3$) δ 7.74 (ddd, *J* = 8.1, 5.0, 1.3 Hz, 2H, Ar-H), 7.38 (td, *J* = 7.6, 1.4 Hz, 1H, Ar-H), 7.29 (td, *J* = 7.5, 1.2 Hz, 1H, Ar-H), 7.27 – 7.22 (m, 1H, Ar-H), 7.18 (ddt, *J* = 7.6, 1.4, 0.6 Hz, 1H, Ar-H), 7.06 (td, *J* = 7.5, 1.3 Hz, 1H, Ar-H), 6.98 (dd, *J* = 8.1, 1.2 Hz, 1H, Ar-H), 5.67 (dd, *J* = 9.0, 5.1 Hz, 1H, CH), 2.85 (dd, *J* = 14.9, 9.0 Hz, 1H, $CH_2C(O)O$), 2.62 (dd, *J* = 14.9, 5.1 Hz, 1H, $CH_2C(O)O$), 1.49 (s, 9H, $C(CH_3)_3$) ppm; **^{13}C NMR (101 MHz, $CDCl_3$)** δ 169.6 (C(O)O), 152.5 (ArC), 133.4 (ArC), 129.8 (ArCH), 129.2 (ArC), 128.6 (ArCH), 127.9 (ArCH), 124.9 (ArCH), 123.1 (ArCH), 122.4 (ArCH), 122.24 (ArC), 122.23 (ArCH), 118.3 (ArCH), 81.1 ($C(CH_3)_3$), 74.5 (CH), 41.5 ($CH_2C(O)O$), 28.2 ($C(CH_3)_3$) ppm; **HRMS** (ESI) m/z calcd. for $C_{19}H_{20}O_3Na$ ($[M+Na]^+$) 319.1305, found 319.1305; **FT-IR (thin film)** ν_{max} 2977, 1727, 1607, 1593, 1485, 1456, 1440, 1392, 1367, 1301, 1247, 1199, 1150, 1123, 1098, 1041, 1020, 959, 842, 807, 755, 725, 645, 617 cm^{-1}; **m.p.**: 58-60 °C; **$[\alpha]_D^{25}$** = +33.1 (c=1.08, $CHCl_3$); **HPLC:** Chiralcel OD, hexane/isopropanol = 90/10, 1.0 ml/min, λ = 240 nm, t_R (major) = 9.9 min, t_R (minor) = 28.6 min.

7 Determination of Absolute Stereochemical Configuration

Compound	This work	Literature
**4b**	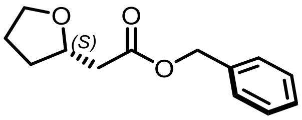	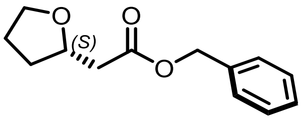
	$[\alpha]_D^{25}$ = +6.4 (c=0.40, $CHCl_3$)<br>er = 94:6	$[\alpha]_D^{25}$ = +3.1 (c = 0.40, $CHCl_3$)<br>er = 97:3 [37]
**5i**	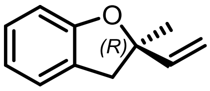	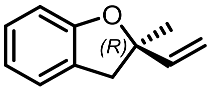
	$[\alpha]_D^{25}$ = +6.0 (c=0.32, $CHCl_3$)<br>er = 99:1	$[\alpha]_D^{23}$ = +9.4 (c=1.0, $CHCl_3$)<br>er = 90.5:9.5 [39]
**4am**	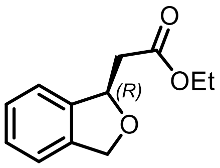	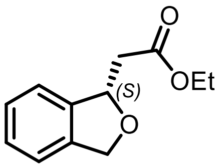
	$[\alpha]_D^{25}$ = +32.7 (c=0.24, $CHCl_3$)<br>er = 95:5	$[\alpha]_D^{26}$ = −35.8 (c=0.40, $CHCl_3$)<br>er = 90.5:9.5 [32]
**4aq**	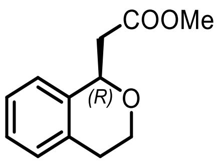	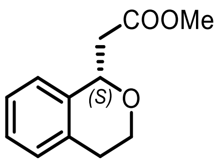
	$[\alpha]_D^{25}$ = +93.8 (c=0.36, $CHCl_3$)<br>er = 90:10	$[\alpha]_D^{25}$ = −51.5 (c = 0.50, $CHCl_3$)<br>er = 67:33 [37]

Compound	This work	Literature
**4k**	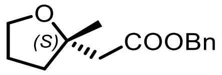	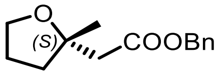
	$[\alpha]_D^{25}$ = +6.8 (c=0.64, $CHCl_3$)<br>er = 94:6	$[\alpha]_D^{25}$ = +4.5 (c = 0.30, $CHCl_3$)<br>er = 95:5 [38]
**5a**	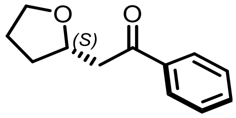	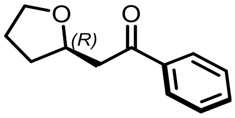
	$[\alpha]_D^{23}$ = +2.3 (c=4.98, CH_2Cl_2)<br>er = 92.5:7.5	$[\alpha]_D^{23}$ = −3.5 (c=4.98, CH_2Cl_2)<br>er = 97.5:2.5 [40]
**5j**	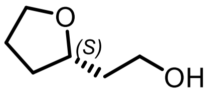	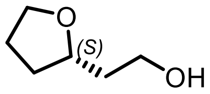
	$[\alpha]_D^{25}$ = +11.0 (c=0.23, $CHCl_3$)<br>er = 96.5:3.5	$[\alpha]_D^{26}$ = −5.9 (c=2.96, EtOH)<br>er = 97:3 [40]

8 Scale up and Derivatization

Preparative scale synthesis of 4d

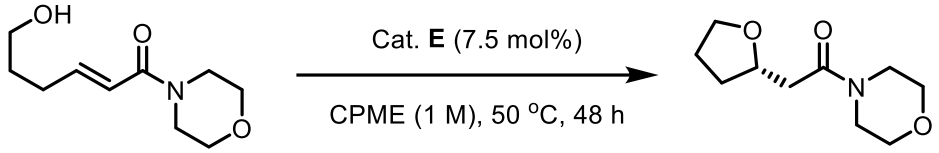

3d; 41.2 mmol

4d
7.6 g, 93%, 95:5 e.r.
after recrystallisation
96.5:3.5 e.r.

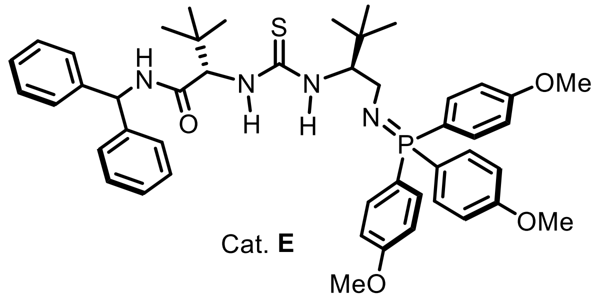

Cat. **E**

CPME (0.025 M, 124 mL) was added to the BIMP catalysts precursors **S1** (1.484 g, 3.09 mmol, 0.075 eq.) and tris(4-methoxyphenyl)phosphine (1.088 g, 3.09 mmol, 0.075 eq.) in a 250 mL RBF under N_2 at room temperature. The reaction mixture was stirred for 24 hours before evaporating to dryness under reduced pressure. The iminophosphorane product was confirmed by HRMS and TLC and used as crude for enantioselective reactions without any purification.

Alcohol **3d** (8.21 g, 41.2 mmol, 1.0 eq.) was added to a solution of the *in situ* generated catalyst (3.09 mmol, 0.075 eq.) in CPME (1M, 41.2 mL) under N_2 at 50 °C. The reaction mixture was stirred at room temperature for 48 hours before quenching with 1M AcOH in CH_2Cl_2 (20 mL). The resulting mixture was then evaporated to dryness under reduced pressure. Purification by silica gel chromatography (pentane/acetone = 3/2) afforded **4d** as an off-white solid (7.60 g, 93%, 95:5 er).

Material was refluxed in the minimum volume of hexane (approximately 100 mL) before carefully cooling down to room temperature. The crushed out solid was filtered, washed with minimum volume of cold hexane (approximately 10 mL) and dried under reduced pressure to afford **4d** in 96.5:3.5 er.

Preparative scale synthesis of 4ai

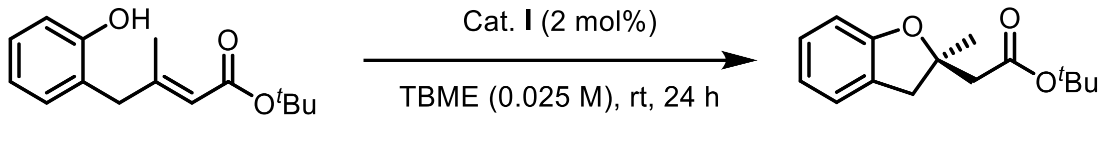

3ai, 6 mmol

4ai, 1.46 g, 98%
99:1 e.r.

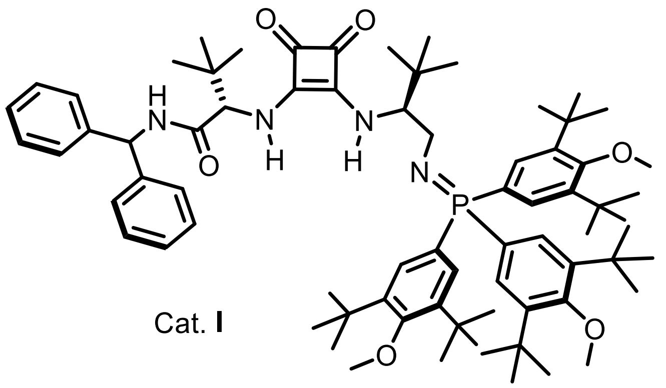

Cat. **I**

THF (0.025 M, 4.8 mL) was added to the BIMP catalysts precursors (62 mg, 0.12 mmol, 0.02 eq.) and tris(4-methoxyphenyl)phosphine (82.7 mg, 0.12 mmol, 0.02 eq.) in a 10 mL RBF under N_2 at room temperature. The reaction mixture was stirred for 24 hours before evaporating to dryness under reduced pressure. The iminophosphorane product was confirmed by HRMS and TLC and used as crude for enantioselective reactions without any purification.

Alcohol **3ai** (1.49 g, 6.0 mmol, 1.0 eq.) was added to a solution of the *in situ* generated catalyst (0.12 mmol, 0.02 eq.) in TBME (0.025M, 240 mL) under N_2 at room temperature. The reaction mixture was stirred at room temperature for 24 hours before quenching with 1M AcOH in CH_2Cl_2 (120 mL). The resulting

mixture was then evaporated to dryness under reduced pressure. Purification by silica gel chromatography (pentane/Et_2O = 95/5) afforded **4ai** as a colorless oil (1.46 g, 98%, 99:1 er).

Derivatization

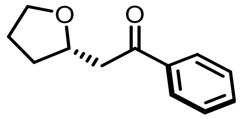

(*S*)-1-phenyl-2-(tetrahydrofuran-2-yl)ethan-1-one (5a) was prepared according to the following procedure. $CeCl_3$ (296.0 mg, 1.2 mmol, 3.0 eq.) (anhydrous grade, stored and weighed in a glove box) was stirred at 90 °C for 30 mins and 135 °C for 2 hours under reduced pressure before cooling down to 0 °C. Anhydrous THF (2.4 mL) was added to the reaction vessel under N_2 at 0 °C. The resulting slurry was warmed to room temperature and stirred for 24 hours under N_2 before cooling down to −78 °C. Phenylmagnesium bromide (1M in THF) (1.0 mL, 1.0 mmol, 2.5 eq.) was added dropwise under N_2 at −78 °C. The resulting mixture was stirred at −78 °C for 1 hour, affording a solution of phenylcerium(III) chloride which was used as crude for the addition without any purification.

A solution of **(*S*)-1-morpholino-2-(tetrahydrofuran-2-yl)ethan-1-one (4d)** (79.7 mg, 0.4 mmol, 1.0 eq., 96.5:3.5 er) in anhydrous THF (0.4 mL) was added dropwise to the phenylcerium(III) chloride under N_2 at −78 °C. The resulting mixture was stirred at −78 °C for 2 hours before quenching with a saturated aqueous solution of NH_4Cl (10 mL). The aqueous layer was extracted with Et_2O (3 x 50 mL). The combined organic layers were washed with brine (50 mL), dried over Na_2SO_4, filtered and evaporated to dryness under reduced pressure. Purification by silica gel chromatography (pentane/Et_2O = 7/3) afforded the title compound as a colorless oil (57.0 mg, 75%, 92.5:7.5 er). Data is consistent with the published literature.[40]

^{1}H NMR (400 MHz, $CDCl_3$) δ 7.99 – 7.92 (m, 2H, Ar-$\underline{H}$), 7.59 – 7.49 (m, 1H, Ar-$\underline{H}$), 7.49 – 7.39 (m, 2H, Ar-$\underline{H}$), 4.39 (dq, *J* = 7.6, 6.4 Hz, 1H, C$\underline{H}$), 3.88 (dt, *J* = 8.3, 6.8 Hz, 1H, $OC\underline{H}_2CH_2CH_2$), 3.79 – 3.68 (m, 1H, $OC\underline{H}_2CH_2CH_2$), 3.38 (dd, *J* = 16.3, 6.1 Hz, 1H, $C\underline{H}_2C(O)$), 3.04 (dd, *J* = 16.2, 6.7 Hz, 1H, $C\underline{H}_2C(O)$), 2.18 (ddt, *J* = 12.3, 7.9, 6.1 Hz, 1H, $OCH_2CH_2C\underline{H}_2$), 1.91 (dqd, *J* = 9.5, 7.2, 2.1 Hz, 2H, $OCH_2C\underline{H}_2CH_2$), 1.55 (ddt, *J* = 12.4, 8.8, 7.7 Hz, 1H, $OCH_2CH_2C\underline{H}_2$) ppm; **^{13}C NMR (101 MHz, $CDCl_3$)** δ 198.5 ($\underline{C}$(O)), 137.2 (Ar$\underline{C}$), 133.2 (Ar$\underline{C}$H), 128.7 (Ar$\underline{C}$H), 128.3 (Ar$\underline{C}$H), 75.5 ($\underline{C}$H), 67.9 ($O\underline{C}H_2CH_2CH_2$), 44.7 ($\underline{C}H_2C(O)$), 31.7 ($OCH_2CH_2\underline{C}H_2$), 25.7 ($OCH_2\underline{C}H_2CH_2$) ppm; **HRMS** (ESI) m/z calcd. for $C_{12}H_{15}O_2$ ($[M+H]^+$) 191.1067, found 191.1067; **FT-IR (thin film)** ν_{max} 2871, 1683, 1598, 1580, 1449, 1280, 1214, 1064, 1002, 926, 752, 690, 668 cm^{-1}; **$[\alpha]_D^{23}$** = +2.3 (c=4.98, CH_2Cl_2); **HPLC:** Chiralcel IA, hexane/isopropanol = 99/1, 1.0 ml/min, λ = 280 nm, t_R (major) = 22.4 min, t_R (minor) = 20.7 min.

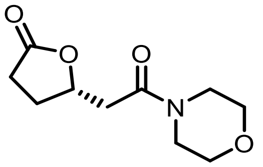

(*S*)-5-(2-morpholino-2-oxoethyl)dihydrofuran-2(3H)-one (5b) was prepared according to the following procedure. According to the modified literature procedure reported by H. Y. Kwon *et. al.*, [41] $NaIO_4$ (0.69 g, 3.2 mmol, 8 eq.) and $RuCl_3{\cdot}3H_2O$ (2.1 mg, 0.008 mmol, 0.02 eq.) were added to a solution of **(*S*)-1-morpholino-2-(tetrahydrofuran-2-yl)ethan-1-one (4d)** (79.7 mg, 0.4 mmol, 1.0 eq., 96.5:3.5 er) in a mixture of CCl_4 (6.7 mL), MeCN (6.7 mL) and H_2O (10 mL) under air at room temperature. The resulting mixture was stirred for 24 hours at room temperature before extracting with CH_2Cl_2 (3 x 30 mL). The combined organic layers were dried over Na_2SO_4, filtered and evaporated to dryness under reduced

pressure. Purification by silica gel chromatography (pentane/acetone = 11:9) afforded the title compound as a white solid (29.0 mg, 34%, >99.5:0.5 er).

^{1}H NMR (400 MHz, CDCl$_3$) δ 5.06 – 4.83 (m, 1H, C<u>H</u>), 3.74 – 3.52 (m, 6H, NC<u>H</u>$_2$C<u>H</u>$_2$O), 3.44 (td, *J* = 4.4, 1.3 Hz, 2H, NC<u>H</u>$_2$C<u>H</u>$_2$O), 2.88 (dd, *J* = 15.8, 5.5 Hz, 1H, C<u>H</u>$_2$C(O)N), 2.63 – 2.47 (m, 4H, C<u>H</u>$_2$C(O)N and C<u>H</u>$_2$C<u>H</u>$_2$C(O)O), 2.04 – 1.88 (m, 1H, C<u>H</u>$_2$CH$_2$C(O)O) ppm; **^{13}C NMR (101 MHz, CDCl$_3$)** δ 176.7 (<u>C</u>(O)), 167.6 (<u>C</u>(O)), 77.5 (<u>C</u>H), 66.8 (N<u>C</u>H$_2$CH$_2$O), 66.6 (N<u>C</u>H$_2$CH$_2$O), 46.2 (N<u>C</u>H$_2$<u>C</u>H$_2$O), 42.0 (N<u>C</u>H$_2$<u>C</u>H$_2$O), 38.5 (<u>C</u>H$_2$C(O)N), 28.6 (<u>C</u>H$_2$<u>C</u>H$_2$C(O)O), 28.3 (<u>C</u>H$_2$<u>C</u>H$_2$C(O)O) ppm; **HRMS** (ESI) m/z calcd. for $C_{10}H_{16}O_4N$ ([M+H]$^+$) 214.1074, found 214.1076; **FT-IR (thin film)** ν_{max} 3500, 3018, 2922, 2857, 2349, 1769, 1637, 1444, 1353, 1302, 1274, 1233, 1180, 1146, 1114, 1069, 1036, 963, 920, 851, 752, 665, 652, 632, 613 cm^{-1}; **m.p.**: 78-79 °C; **$[\alpha]_D^{25}$** = +2.9 (c=0.70, CHCl$_3$); **Chiral GC:** Supelco β-dex™ 325, 30 m, 0.25 mm, 0.25 μm, carrier gas He (flow rate 20 cm/s); column temperature: 30℃ for 10 mins, then ramp 20℃/min to 230℃ for 10 min, then 230℃ for 40 mins; t_R (single enantiomer) = 23.84 min.

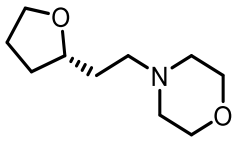

(*S*)-4-(2-(tetrahydrofuran-2-yl)ethyl)morpholine (5c) was prepared according to the following procedure. According to the modified literature procedure reported by G. Barbe *et. al.*, [42] Tf_2O (124.2 mg, 0.44 mmol, 1.1 eq.) was added to a solution of **(*S*)-1-morpholino-2-(tetrahydrofuran-2-yl)ethan-1-one (4d)** (79.7 mg, 0.40 mmol, 1.0 eq., 96.5:3.5 er) in CH_2Cl_2 (1.6 mL) under N_2 at room temperature. The resulting mixture was stirred for 5 mins at room temperature before adding Hantzsch ester (254.0 mg, 1.0

mmol, 2.5 eq.). The mixture was stirred for 16 hours before quenching with MeOH (1.6 mL) and 12M NaOH aqueous solution (0.4 mL). The resulting mixture was further stirred for 2 hours. The aqueous layers was extracting with CH_2Cl_2 (3 x 30 mL). The combined organic layers were dried over Na_2SO_4, filtered and evaporated to dryness under reduced pressure. Purification by silica gel chromatography (CH_2Cl_2/MeOH = 1/0 to 97.5/2.5) afforded the title compound as a colorless oil (43.2 mg, 59%, 98:2 er).

^{1}H NMR (400 MHz, CDCl$_3$) δ 3.92 – 3.77 (m, 2H, C<u>H</u> and C<u>H</u>$_2$), 3.74 – 3.64 (m, 5H, C<u>H</u>$_2$), 2.43 (ttt, *J* = 17.8, 12.2, 5.6 Hz, 6H, C<u>H</u>$_2$), 2.05 – 1.93 (m, 1H, C<u>H</u>$_2$), 1.92 – 1.80 (m, 2H, C<u>H</u>$_2$), 1.80 – 1.60 (m, 2H, C<u>H</u>$_2$), 1.52 – 1.39 (m, 1H, C<u>H</u>$_2$) ppm; **^{13}C NMR (101 MHz, CDCl$_3$)** δ 77.7 (<u>C</u>H), 67.8 (<u>C</u>H$_2$), 67.0 (<u>C</u>H$_2$), 56.3 (<u>C</u>H$_2$), 53.9 (<u>C</u>H$_2$), 32.8 (<u>C</u>H$_2$), 31.7 (<u>C</u>H$_2$), 25.8 (<u>C</u>H$_2$) ppm; **HRMS** (ESI) m/z calcd. for $C_{10}H_{20}O_2N$ ($[M+H]^+$) 186.1489, found 186.1488; **FT-IR (thin film)** ν_{max} 3486, 2950, 2853, 1656, 1448, 1360, 1275, 1208, 1142, 1117, 1071, 1007, 916, 865, 753, 610 cm^{-1}; **$[\alpha]_D^{25}$** = +9.5 (c=0.89, CHCl$_3$); **Chiral GC:** Supelco β-dex™ 325, 30 m, 0.25 mm, 0.25 μm, carrier gas He (flow rate 20 cm/s); column temperature: 30 ℃ for 10 mins, then ramp 20℃/min to 230℃ for 10 min, then 230℃ for 40 mins; t_R (major) = 33.64 min, t_R (minor) = 32.88 min.

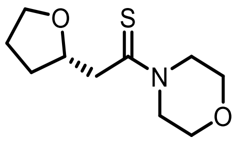

(*S*)-1-morpholino-2-(tetrahydrofuran-2-yl)ethane-1-thione (5d) was prepared according to the following procedure. According to the modified literature procedure reported by L. K. Ransborg *et. al.*, [43] Lawesson's reagent (66.8 mg, 0.17 mmol, 0.55 eq.) was added to a solution of **(*S*)-1-morpholino-2-**

(tetrahydrofuran-2-yl)ethan-1-one (4d) (59.8 mg, 0.3 mmol, 1.0 eq., 96.5:3.5 er) in anhydrous THF (0.7 mL) under N_2 at room temperature. The reaction mixture was stirred at 60 °C for 3 days before evaporating to dryness under reduced pressure. Purification by silica gel chromatography (CH_2Cl_2/EtOAc = 9:1) afforded the title compound as a colorless oil (29.7 mg, 46%, 97:3 er).

^{1}H NMR (400 MHz, $CDCl_3$) δ 4.61 (ddd, *J* = 13.6, 5.7, 3.3 Hz, 1H), 4.29 (qd, *J* = 7.2, 4.7 Hz, 1H, $C\underline{H}$), 4.09 (ddd, *J* = 13.5, 7.6, 3.4 Hz, 1H, $NC\underline{H}_2C\underline{H}_2O$), 4.01 – 3.89 (m, 1H, $NC\underline{H}_2C\underline{H}_2O$), 3.85 – 3.62 (m, 7H, $OC\underline{H}_2CH_2CH_2$ and $NC\underline{H}_2C\underline{H}_2O$), 3.17 – 3.01 (m, 2H, $C\underline{H}_2C(S)$), 2.18 – 2.05 (m, 1H, $OCH_2CH_2C\underline{H}_2$), 1.88 (p, *J* = 7.0 Hz, 2H, $OCH_2C\underline{H}_2CH_2$), 1.76 – 1.62 (m, 1H, $OCH_2CH_2C\underline{H}_2$) ppm; **^{13}C NMR (101 MHz, $CDCl_3$)** δ 200.3 ($\underline{C}(S)$), 79.0 ($\underline{C}H$), 68.1 ($N\underline{C}H_2\underline{C}H_2O$ or $O\underline{C}H_2CH_2CH_2$), 66.7 ($N\underline{C}H_2\underline{C}H_2O$ or $O\underline{C}H_2CH_2CH_2$), 66.6 ($N\underline{C}H_2\underline{C}H_2O$ or $O\underline{C}H_2CH_2CH_2$), 51.1 ($N\underline{C}H_2\underline{C}H_2O$ or $O\underline{C}H_2CH_2CH_2$), 50.3 ($N\underline{C}H_2\underline{C}H_2O$ or $O\underline{C}H_2CH_2CH_2$), 49.0 ($\underline{C}H_2C(S)$), 31.5 ($OCH_2CH_2\underline{C}H_2$), 25.6 ($OCH_2\underline{C}H_2CH_2$) ppm; **HRMS** (ESI) m/z calcd. for $C_{10}H_{18}O_2NS$ ($[M+H]^+$) 216.1053, found 206.1053; **FT-IR (thin film)** ν_{max} 3774, 3706, 3487, 2919, 2855, 2349, 2283, 1711, 1691, 1658, 1641, 1548, 1492, 1433, 1281, 1199, 1116, 1090, 1063, 1024, 871, 771, 718, 664, 652, 639, 621, 612 cm^{-1}; **$[\alpha]_D^{25}$** = +18.6 (c=0.58, $CHCl_3$); **HPLC:** Chiralcel OD, hexane/isopropanol = 97/3, 1.0 ml/min, λ = 280 nm, t_R (major) = 19.7 min, t_R (minor) = 24.0 min.

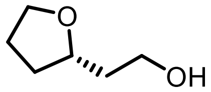

(*S*)-2-(Tetrahydrofuran-2-yl)ethan-1-ol (5j) was prepared according to the following procedure. According to the modified literature procedure reported by M. Szostak *et. al.*, [44] to an oven-dried round

bottom flask equipped with a magnetic stirrer bar was added **(*S*)-1-morpholino-2-(tetrahydrofuran-2-yl)ethan-1-one (4d)** (79.7 mg, 0.4 mmol, 1.0 eq., 96.5:3.5 er), Samarium(II) iodide (0.1M THF solution) (32 mL, 3.2 mmol, 8.0 eq.), degassed Et_3N (4 mL) and degassed H_2O (0.52 mL) under N_2 at room temperature. The resulting mixture was stirred vigorously under N_2 at room temperature. After 18 hours, air was bubbled through the mixture to quench the reaction. The reaction mixture was diluted with CH_2Cl_2 (30 mL) and 1M NaOH (10 mL) before extracting with CH_2Cl_2 (3 x 30 mL). The combined organic layers were dried over Na_2SO_4, filtered and evaporated to dryness under reduced pressure. Purification by silica gel chromatography (pentane/Et_2O = 3:7) afforded the title compound as a colorless oil (21.7 mg, 47%, 96.5:3.5 er). Data is consistent with the published literature.[40]

The reaction was repeated in a 0.05 mmol scale and 1.0 mL reaction mixture was taken after air bubbling for GC analysis. GC yield and er was determined with DMSO as an external standard to be 98% yield and 96.5:3.5 er.

^{1}H NMR (400 MHz, $CDCl_3$) δ 4.00 (tdd, *J* = 8.0, 6.4, 4.3 Hz, 1H, C<u>H</u>), 3.88 (ddd, *J* = 8.3, 7.3, 6.3 Hz, 1H, C<u>H</u>$_2$), 3.80 – 3.68 (m, 3H, C<u>H</u>$_2$), 2.64 (brs, 1H, O<u>H</u>), 2.07 – 1.95 (m, 1H, C<u>H</u>$_2$), 1.95 – 1.82 (m, 2H, C<u>H</u>$_2$), 1.80 – 1.67 (m, 2H, C<u>H</u>$_2$), 1.52 (ddt, *J* = 11.9, 8.7, 7.7 Hz, 1H, C<u>H</u>$_2$) ppm; **^{13}C NMR (101 MHz, $CDCl_3$)** δ 79.3 (<u>C</u>H), 68.0 (<u>C</u>H$_2$), 61.7 (<u>C</u>H$_2$), 37.4 (<u>C</u>H$_2$), 31.8 (<u>C</u>H$_2$), 25.6 (<u>C</u>H$_2$) ppm; **HRMS** (ESI) m/z calcd. for $C_6H_{13}O_2$ ($[M+H]^+$) 117.0910, found 117.0912; **FT-IR (thin film)** ν_{max} 3020, 2922, 2851, 1434, 1312, 1216, 1029, 759, 650, 630, 608 cm^{-1}; **$[\alpha]_D^{25}$** = +11.0 (c=0.23, $CHCl_3$); **Chiral GC:** Supelco β-dex™ 325, 30 m, 0.25 mm, 0.25 μm, carrier gas He (flow rate 20 cm/s); column temperature: 30 ℃ for 10 mins,

then ramp 20 ℃/min to 230 ℃ for 10 min, then 230 ℃ for 40 mins; t_R (major) = 27.56 min, t_R (minor) = 27.65 min.

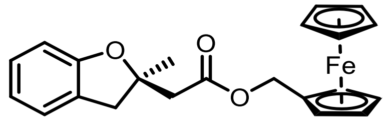

Ferrocene-methyl (*R*)-2-(2-methyl-2,3-dihydrobenzofuran-2-yl)acetate (5e) was prepared according to the following procedure. Trifluoroacetic acid (4.3 mL, 7 mL/mmol) was added dropwise to a solution of the ***tert*-Butyl (*R*)-2-(2-methyl-2,3-dihydrobenzofuran-2-yl)acetate (4ai)** (150.7 mg, 0.61 mmol, 1.0 eq., 99:1 er) in CH_2Cl_2 (4.3 mL, 7 mL/mmol) under N_2 at 0 °C. The reaction mixture was warmed to room temperature and stirred for 2 hours. Volatiles were removed under a stream of nitrogen gas to afford (*R*)-2-(2-methyl-2,3-dihydrobenzofuran-2-yl)acetic acid which was used as crude for next step without any purification.

Thionyl chloride (217.7 mg, 1.83 mmol, 3.0 eq.) and a few drops of DMF was added dropwise to a solution of the (*R*)-2-(2-methyl-2,3-dihydrobenzofuran-2-yl)acetic acid in CH_2Cl_2 (1.6 mL, 0.4M) under N_2 at 0 °C. The reaction mixture was warmed to room temperature and stirred for 2 hours. Volatiles were removed under a stream of nitrogen gas to afford (*R*)-2-(2-methyl-2,3-dihydrobenzofuran-2-yl)acetyl chloride which was used as crude for next step without any purification.

Ferrocenemethanol (158.2 mg, 0.74 mmol, 1.2 eq.) and Et_3N (74.9 mg, 0.74 mmol, 1.2 eq.) was added to a solution of the (*R*)-2-(2-methyl-2,3-dihydrobenzofuran-2-yl)acetyl chloride in CH_2Cl_2 (2.6 mL) under N_2 at 0 °C. The reaction mixture was warmed to room temperature and stirred for 2 hours before quenching

with saturated aqueous solution of NH_4Cl (10 mL). The organic layer was washed with saturated aqueous solution of $NaHCO_3$ (10 mL), dried over Na_2SO_4, filtered and evaporated to dryness under reduced pressure. Purification by silica gel chromatography (pentane/Et2O = 4/1) afforded the title compound as a pale-yellow oil (82.5 mg, 35%, 96:4 er).

^{1}H NMR (400 MHz, CDCl$_3$) δ 7.20 – 7.07 (m, 2H, Ar-H), 6.85 (td, *J* = 7.4, 1.0 Hz, 1H, Ar-H), 6.75 (dd, *J* = 8.0, 1.0 Hz, 1H, Ar-H), 4.90 (s, 2H, OCH_2), 4.29 (s, 1H, Ar-H (ferrocene)), 4.25 (dq, *J* = 2.6, 1.5 Hz, 2H, Ar-H (ferrocene)), 4.20 – 4.15 (m, 5H, Ar-H (ferrocene)), 4.14 (s, 1H, Ar-H (ferrocene)), 3.41 – 3.33 (m, 1H, CH_2C(O)O), 3.06 – 2.97 (m, 1H, CH_2C(O)O), 2.83 – 2.70 (m, 2H, ArCH_2), 1.56 (s, 3H, CH_3) ppm; **^{13}C NMR (101 MHz, CDCl$_3$)** δ 170.1 (C(O)O), 158.4 (ArCO), 128.1 (ArCH), 126.6 (ArCC), 125.2 (ArCH), 120.5 (ArCH), 109.7 (ArCH), 86.1 (ArC (ferrocene) or OC), 81.1 (ArC (ferrocene) or OC), 69.6 (ArCH (ferrocene)), 69.4 (ArCH (ferrocene)), 68.9 (ArCH (ferrocene)), 68.6 (ArCH (ferrocene)), 68.5 (ArCH (ferrocene)), 68.4 (ArCH (ferrocene)), 68.0 (ArCH (ferrocene)), 63.1 (OCH_2), 45.2 (ArCH_2), 41.3 (CH_2C(O)O), 26.6 ppm; **HRMS** (ESI) m/z calcd. for $C_{22}H_{22}O_3Fe$ ($[M+H+Na]^+$) 414.0364, found 414.0364; **FT-IR (thin film)** ν_{max} 2980, 1731, 1598, 1481, 1461, 1378, 1329, 1242, 1190, 1170, 1106, 1064, 1040, 1000, 924, 885, 820, 751, 710, 625 cm^{-1}; **$[\alpha]_D^{25}$** = +5.4 (c=1.51, $CHCl_3$); **HPLC:** Chiralcel IB, hexane/isopropanol = 98/2, 1.0 ml/min, λ = 280nm, t_R (major) = 11.7 min, t_R (minor) = 13.0 min.

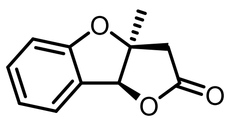

5f

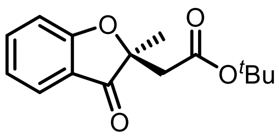

5g

(3a*S*,8b*S*)-3a-methyl-3a,8b-dihydrofuro[3,2-b]benzofuran-2(3H)-one (5f) and ***tert*-Butyl (*S*)-2-(2-methyl-3-oxo-2,3-dihydrobenzofuran-2-yl)acetate (5g)** were prepared according to the following procedure. To an oven-dried screw-cap vial equipped with a magnetic stirrer bar was added $K_2S_2O_8$ (316.3 mg, 1.17 mmol, 3.0 eq.), $CuSO_4$ (62.3 mg, 0.39 mmol, 1.0 eq.), ***tert*-Butyl (*R*)-2-(2-methyl-2,3-dihydrobenzofuran-2-yl)acetate (4ai)** (97.2 mg, 0.39 mmol, 1.0 eq., 99:1 er), MeCN (14 mL) and H_2O (14 mL). The vial was closed and heated to 80 °C. The resulting mixture was stirred at 80 °C for 1 hour. After cooled to room temperature, the mixture was extracted with Et_2O (3 x 50 mL). The combined organic layers were washed with brine (50 mL). The resulting mixture was dried over Na_2SO_4, filtered and evaporated to dryness under reduced pressure. Purification by silica gel chromatography (pentane/Et_2O = 1:1) afforded **5f** as a white solid (38.5 mg, 52%, 99:1 er) and (pentane/Et_2O = 95:5) afforded **5g** as a white solid (22.8 mg, 23%, 96.5:3.5 er)

5f ^{1}H NMR (400 MHz, CDCl$_3$) δ 7.45 (ddd, *J* = 7.6, 1.4, 0.7 Hz, 1H, Ar-H), 7.33 (ddd, *J* = 8.2, 7.5, 1.4 Hz, 1H, Ar-H), 6.98 (td, *J* = 7.5, 0.9 Hz, 1H, Ar-H), 6.85 (dq, *J* = 8.2, 0.7 Hz, 1H, Ar-H), 5.58 (s, 1H, CH), 3.12 (d, *J* = 18.8 Hz, 1H, CH_2), 2.84 (d, *J* = 18.8 Hz, 1H, CH_2), 1.62 (s, 3H, CH_3) ppm; **^{13}C NMR (101 MHz, CDCl$_3$)** δ 174.4 (C(O)O), 160.2 (ArCO), 132.2 (ArCH), 127.3 (ArCH), 122.9 (ArC), 121.7 (ArCH), 111.0 (ArCH), 89.7 (OC), 88.1 (CH), 41.0 (CH_2), 23.0 (CH_3) ppm; **HRMS** (ESI) m/z calcd. for $C_{11}H_9O_3$ ([M-H]$^-$) 189.0546, found 189.0549; **FT-IR (thin film)** ν_{max} 1779, 1614, 1601, 1478, 1468, 1403, 1384, 1329, 1297, 1257, 1192, 1166, 1123, 1075, 996, 940, 896, 877, 840, 822, 753, 702, 624 cm^{-1}; **m.p.**: 79-80 °C; **$[\alpha]_D^{25}$**

= −22.5 (c=0.72, $CHCl_3$); **HPLC:** Chiralcel AD-H, hexane/isopropanol = 90/10, 1.0 ml/min, λ = 280 nm, t_R (major) = 18.6 min, t_R (minor) = 12.9 min.

5g ^{1}H NMR (400 MHz, $CDCl_3$) δ 7.72 – 7.65 (m, 1H, Ar-H), 7.60 (ddd, *J* = 8.6, 7.2, 1.5 Hz, 1H, Ar-H), 7.11 – 7.05 (m, 2H, Ar-H), 3.03 (d, *J* = 15.7 Hz, 1H, CH_2), 2.80 (d, *J* = 15.7 Hz, 1H, CH_2), 1.43 (s, 3H, CH_3), 1.14 (s, 9H, $C(CH_3)_3$) ppm; **^{13}C NMR (101 MHz, $CDCl_3$)** δ 202.7 (C(O)), 171.2 (C(O)O), 167.6 (ArCO), 137.8 (ArCH), 124.8 (ArCH), 121.9 (ArCH), 121.0 (ArCC), 113.5 (ArCH), 86.9 (OC), 81.7 ($C(CH_3)_3$), 43.5 (CH_2), 27.7 ($C(CH_3)_3$), 22.9 (CH_3) ppm; **HRMS** (ESI) m/z calcd. for $C_{15}H_{18}O_4Na$ ($[M+Na]^+$) 285.1097, found 285.1098; **FT-IR (thin film)** ν_{max} 2980, 2358, 1726, 1614, 1478, 1465, 1393, 1369, 1323, 1307, 1271, 1158, 1121, 1074, 963, 873, 845, 757, 688, 628 cm^{-1}; **m.p.**: 51-53 °C; **$[\alpha]_D^{25}$** = +12.3 (c=0.70, $CHCl_3$); **HPLC:** Chiralcel AD-H, hexane/isopropanol = 97/3, 1.0 ml/min, λ = 254 nm, t_R (major) = 7.6 min, t_R (minor) = 9.0 min.

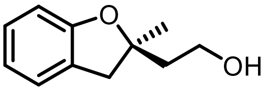

(*R*)-2-(2-methyl-2,3-dihydrobenzofuran-2-yl)ethan-1-ol (5h) was prepared according to the following procedure. $LiAlH_4$ (0.54 mL, 2M in THF, 1.5 eq.) was added dropwise to a solution of the ***tert*-Butyl (*R*)-2-(2-methyl-2,3-dihydrobenzofuran-2-yl)acetate (4ai)** (176.1 mg, 0.71 mmol, 1.0 eq., 99:1 er) in THF (0.71 mL) under N_2 at 0 °C. The reaction mixture was warmed to room temperature and stirred for 4 hours before quenching with saturated aqueous solution of NH_4Cl (5 mL). The organic layer was washed with saturated aqueous solution of $NaHCO_3$ (5 mL), dried over Na_2SO_4, filtered and evaporated to dryness under reduced pressure. Purification by silica gel chromatography (pentane/Et_2O = 3/2) afforded the title compound as a colorless oil (84.8 mg, 67%, 99:1 er).

^{1}H NMR (400 MHz, $CDCl_3$) δ 7.18 – 7.07 (m, 2H, Ar-H), 6.84 (td, *J* = 7.4, 1.0 Hz, 1H, Ar-H), 6.74 (d, *J* = 8.0 Hz, 1H, Ar-H), 3.90 (ddd, *J* = 12.3, 7.4, 5.5 Hz, 1H, CH_2CH_2), 3.82 (q, *J* = 5.6 Hz, 1H, CH_2CH_2), 3.20 – 3.12 (d, *J* = 15.5 Hz, 1H, $ArCH_2$), 2.99 (d, *J* = 15.5 Hz, 1H, $ArCH_2$), 2.17 – 2.05 (m, 2H, CH_2CH_2 and OH), 1.99 (ddd, *J* = 14.5, 6.2, 5.3 Hz, 1H, CH_2CH_2), 1.48 (s, 3H, CH_3) ppm; **^{13}C NMR (101 MHz, $CDCl_3$)** δ 158.5 (ArCO), 128.3 (ArCH), 126.7 (ArCC), 125.3 (ArCH), 120.6 (ArCH), 109.8 (ArCH), 88.7 (OC), 59.4 (CH_2CH_2), 43.0 (CH_2CH_2), 42.3 ($ArCH_2$), 26.4 (CH_3) ppm; **HRMS** (ESI) m/z calcd. for $C_{11}H_{15}O_2$ ($[M+H]^+$) 179.1067, found 179.1067; **FT-IR (thin film)** ν_{max} 3384, 2929, 1597, 1481, 1461, 1327, 1239, 1123, 1053, 1016, 870, 750, 710, 667 cm^{-1}; **$[\alpha]_D^{25}$** = +6.4 (c=0.56, $CHCl_3$); **HPLC:** Chiralcel IB, hexane/isopropanol = 95/5, 1.0 ml/min, λ = 230nm, t_R (major) = 10.0 min, t_R (minor) = 11.5 min.

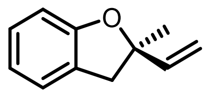

(*R*)-2-methyl-2-vinyl-2,3-dihydrobenzofuran (5i) was prepared according to the following procedure. According to the modified literature procedure reported by L. F. Tietze *et. al.*, [45] 2-nitrophenyl selenocyanate (190.8 mg, 0.84 mmol, 2.0 eq.) and $^{n}Bu_3P$ (161.9 mg, 0.80 mmol, 1.91 eq.) were added to a solution of **(*R*)-2-(2-methyl-2,3-dihydrobenzofuran-2-yl)ethan-1-ol (5h)** (74.8 mg, 0.42 mmol, 1.0 eq., 99:1 er) in THF (5.3 mL) under N_2 at 0 °C. The reaction mixture was stirred at 0 °C for 1.5 h before adding additional 2-nitrophenyl selenocyanate (47.7 mg, 0.21 mmol, 0.50 eq.) and $^{n}Bu_3P$ (40.5 mg, 0.20 mmol, 0.48 eq.). The stirring was continued at 0 °C for 2.5 h before quenching with saturated aqueous solution of $NaHCO_3$ (10 mL) at 0 °C. The aqueous layer was extracted with Et_2O (3×50 mL). The combined organic layers were dried over Na_2SO_4, filtered and evaporated to dryness under reduced pressure affording (*R*)-2-methyl-2-(2-

((2-nitrophenyl)selanyl)ethyl)-2,3-dihydrobenzofuran which was used as crude for next step without any purification.

H_2O_2 (30% (w/w) in H_2O, 0.1 mL) was added to a solution of (*R*)-2-methyl-2-(2-((2-nitrophenyl)selanyl)ethyl)-2,3-dihydrobenzofuran in THF (5 mL) under N_2 at 0 °C. additional H_2O_2 (30% (w/w) in H_2O, 0.1 mL) was added every 1 hour to the reaction mixture. After 3 hours, the reaction was quenched with H_2O (10 mL). The aqueous layer was extracted with CH_2Cl_2 (3×50 mL). The combined organic layers were dried over Na_2SO_4, filtered and evaporated to dryness under reduced pressure. Purification by silica gel chromatography (pentane to pentane/Et_2O = 95/5) afforded the title compound as a pale-yellow oil (42.6 mg, 64%, 99:1 er). Data is consistent with the published literature.[39]

^{1}H NMR (400 MHz, CDCl$_3$) δ 7.14 (tq, *J* = 7.7, 0.8 Hz, 2H, Ar-H), 6.85 (td, *J* = 7.4, 1.0 Hz, 1H, Ar-H), 6.81 (dt, *J* = 7.5, 0.9 Hz, 1H, Ar-H), 6.07 (dd, *J* = 17.3, 10.7 Hz, 1H, CHCH$_2$), 5.33 (dd, *J* = 17.3, 1.1 Hz, 1H, CHCH$_2$), 5.11 (dd, *J* = 10.7, 1.1 Hz, 1H, CHCH$_2$), 3.20 (dt, *J* = 15.4, 1.0 Hz, 1H, ArCH$_2$), 3.07 (dt, *J* = 15.4, 1.0 Hz, 1H, ArCH$_2$), 1.57 (s, 3H, CH$_3$) ppm; **^{13}C NMR (101 MHz, CDCl$_3$)** δ 159.0 (ArCO), 141.8 (CHCH$_2$), 128.2 (ArCH), 126.6 (ArCC), 125.2 (ArCH), 120.4 (ArCH), 112.9 (CHCH$_2$), 109.7 (ArCH), 87.6 (OC), 42.2 (ArCH$_2$), 26.2 (CH$_3$) ppm; **HRMS** (ESI) m/z calcd. for $C_{11}H_{13}O$ ($[M+H]^+$) 161.0961, found 161.0961; **$[\alpha]_D^{25}$** = 6.0 (c=0.32, CHCl3); **HPLC:** Chiralcel AS-H hexane/isopropanol = 99.5/0.5, 1.0 ml/min, λ = 280nm, t_R (major) = 3.9 min, t_R (minor) = 4.1 min.

9 Computational Studies

General computational information

All calculations reported in this paper were performed using the Amsterdam Density Functional (ADF) software.[46] Equilibrium structures and transition state geometries were optimized using the BLYP functional[47, 48] and the DZP basis set.[49] Solvent effects of THF were accounted for using the conducter-like screening model (COSMO) of solvation.[50] Dispersion interactions were included using Grimme's DFT-D3 correction with Becke-Johnson damping.[51] The zeroth-order regular approximation (ZORA) was used to account for scalar relativistic effects.[52] This level is referred to as COSMO(THF)-ZORA-BLYP-D3(BJ)/DZP. All stationary points have been verified, through vibrational analysis, to be minima (zero imaginary frequencies) or transition state structures (one imaginary frequency). The character of the normal mode associated with the imaginary frequency has been analyzed to ensure it resembles the reaction under consideration. Optimized structures were illustrated using CYLview20.[53] Potential energies were refined by means of single point calculations using the M06-2X functional[54] and the TZ2P basis set.[49] This level is denoted COSMO(THF)-ZORA-M06-2X/TZ2P//COSMO(THF)-ZORA-BLYP-D3(BJ)/DZP.

Quantitative analyses of the activation barriers associated with the oxa-Michael addition were obtained by means of the activation strain model (ASM), which involves decomposing the electronic energy of the transition structure $\Delta E^{\ddagger}$ into the strain $\Delta E^{\ddagger}_{strain}$ associated with the structural deformation of the reactants from their equilibrium geometry and the interaction $\Delta E^{\ddagger}_{int}$ between the deformed reactants [Eq. 1].[55] The $\Delta E^{\ddagger}_{strain}$ is determined by the rigidity of the reactants and by the extent to which they must deform to

achieve the geometry of the transition structure. The $\Delta E^{‡}_{int}$ is usually stabilizing and is related to the electronic structure of the reactants and how they are mutually oriented over the course of the reaction.

$$\Delta E^{‡} = \Delta E_{strain}{}^{‡} + \Delta E_{int}{}^{‡} \tag{1}$$

The interaction energy between the deformed reactants can be further analyzed in terms of quantitative Kohn-Sham molecular orbital theory (KS-MO) together with a canonical energy decomposition analysis (EDA).[56] The EDA decomposes the $\Delta E^{‡}_{int}$ into the following three physically meaningful energy terms [Eq. 4]:

$$\Delta E^{‡}_{int} = \Delta V^{‡}_{elstat} + \Delta E^{‡}_{Pauli} + \Delta E^{‡}_{oi} \tag{4}$$

Herein, $\Delta V^{‡}_{elstat}$ is the classical electrostatic interaction between the unperturbed charge distributions of the (deformed) reactants and is usually attractive. The Pauli repulsion, $\Delta E^{‡}_{Pauli}$, comprises the destabilizing interaction between occupied closed-shell orbitals of both fragments due to the Pauli principle. Finally, the orbital interaction energy, $\Delta E^{‡}_{oi}$, accounts for polarization and charge transfer between the fragments, such as HOMO–LUMO interactions. A detailed, step-by-step, guide on how to perform and interpret the ASM and EDA can be found in Ref. 55a.

Computational Details

In general, there are two modes for which the substrate may bind to the substrate in the transition states (Mode A and Mode B) as originally hypothesized by Pápai (Figure S6).[57] In the case of mode A, the hydrogen-bond donor (HBD) coordinates to the electrophile (carbonyl) and the protonated iminophosphorane coordinates to the nucleophile (alkoxide). In the mode B, the HBD coordinates to the nucleophile (alkoxide) and the protonated iminophosphorane coordinates to the electrophile (carbonyl). Both activation modes were considered in the calculations of the TSs described below.

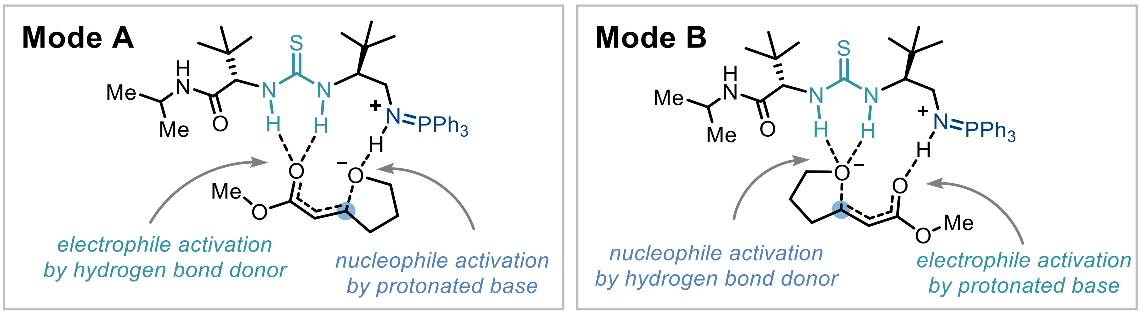

Figure S6. Activation modes of the BIMP catalyst.

In order to differentiate the computed transition states with several conformations, they are named according to the following figure that includes information of the coordination mode, conformations of "*left and right arms*", and the absolute configuration of the product.

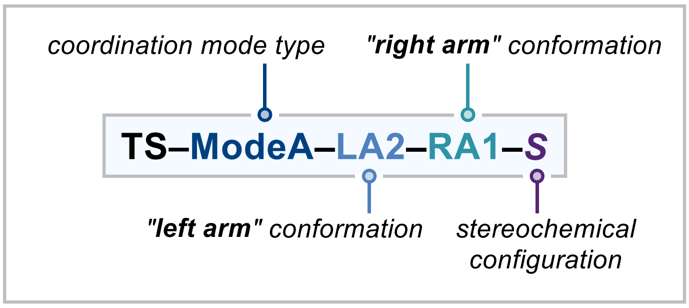

Figure S7. Nomenclature for BIMP-catalyzed oxa-Michael addition transition structures.

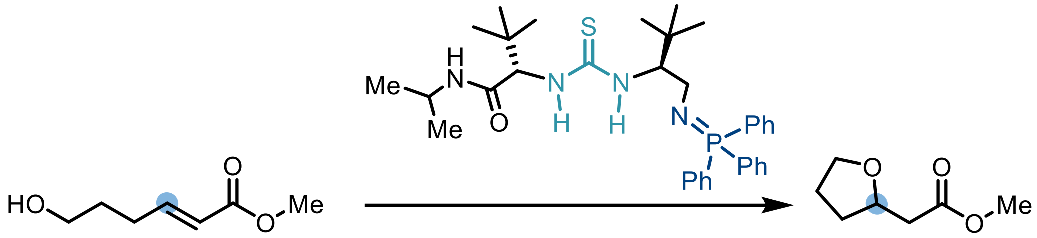

Figure S8. Model reaction for the computational study.

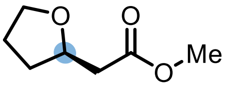

***(R)*-product**

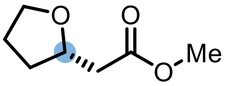

***(S)*-product**

TSs for (R)-product	ΔΔ*G*‡	ΔΔ*E*‡
**TS–ModeA–LA1–RA1–*R***	*n.d.*	*n.d.*
**TS–ModeA–LA1–RA2–*R***	15.1	14.8
**TS–ModeA–LA2–RA1–*R***	13.2	13.5
**TS–ModeA–LA2–RA2–*R***	12.5	10.0
**TS–ModeB–LA1–RA1–*R***	**2.8**	**2.6**
**TS–ModeB–LA1–RA2–*R***	16.2	15.7
**TS–ModeB–LA2–RA1–*R***	9.7	8.8
**TS–ModeB–LA2–RA2–*R***	14.4	13.0

TSs for (S)-product	ΔΔ*G*‡	ΔΔ*E*‡
**TS–ModeA–LA1–RA1–*S***	*n.d.*	*n.d.*
**TS–ModeA–LA1–RA2–*S***	8.6	7.2
**TS–ModeA–LA2–RA1–*S***	10.2	10.9
**TS–ModeA–LA2–RA2–*S***	13.1	12.6
**TS–ModeB–LA1–RA1–*S***	**0.0**	**0.0**
**TS–ModeB–LA1–RA2–*S***	11.2	10.4
**TS–ModeB–LA2–RA1–*S***	*n.d.*	*n.d.*
**TS–ModeB–LA2–RA2–*S***	13.3	12.7

Figure S9. Relative stability of the squaramide BIMP-catalyzed oxa-Michael addition transition structures computed at COSMO(THF)-ZORA-M06-2X/TZ2P//COSMO(THF)-ZORA-BLYP-D3(BJ)/DZP. Energies (kcal mol^{-1}) are provided in the insert.

Table S6. Cartesian coordinates (in Å), energies (in kcal mol^{-1}), and number of imaginary frequencies of all stationary points, computed at COSMO(THF)-ZORA-BLYP-D3(BJ)/DZP. Energies (in kcal mol^{-1}) at COSMO(THF)-ZORA-M06-2X/TZ2P//COSMO(THF)-ZORA-BLYP-D3(BJ)/DZP are also provided.

TS–Mode A–LA1–RA2–*R*
COSMO(THF)-ZORA-M06-2X/TZ2P//COSMO(THF)-ZORA-BLYP-D3(BJ)/DZP
E = -20667.55
G = -20150.26
COSMO(THF)-ZORA-BLYP-D3(BJ)/DZP

E = -14451.20

G = -13933.91

N_{imag} = 1, 290*i* cm^{-1}

C	-4.90471046	-2.02674059	4.36744488
N	-4.30304562	-0.91922131	3.62825553
H	-4.41573382	-0.91438864	2.60102052
C	-1.74426656	-4.18578314	4.68616703
S	-3.05611022	0.06224540	5.80893027
N	-2.73651168	0.62874498	3.12725786
H	-3.11209070	0.40524343	2.19031865
C	-1.81650165	1.77485791	3.12042536
H	-1.36231711	1.72251290	2.11752414
H	-0.94621933	-5.91676054	5.74476439
C	-0.67039560	1.63035972	4.12451595
N	0.20977013	0.48612990	3.74946951
H	-1.08622930	1.48692812	5.12691877
P	1.59866196	0.22354581	4.57426957
C	1.42516515	-0.54475230	6.21190629
C	0.15670618	-0.91296568	6.66975561
C	2.55484573	-0.70845733	7.03520254
C	0.01227601	-1.45799684	7.94878041
H	-0.72059117	-0.75380307	6.04659966
C	2.40590543	-1.27170590	8.30201137
H	3.54012037	-0.39649222	6.69090943
C	1.13407055	-1.64726052	8.75945086
H	-0.97908187	-1.73529413	8.30566853
H	3.27888630	-1.41097439	8.93836889
H	1.02271228	-2.07961323	9.75339340
C	2.35120666	1.83999158	4.96374705
C	1.88668621	2.51435277	6.10894953
C	3.30765885	2.44954395	4.13681427
C	2.35949607	3.79264106	6.40664618
H	1.15930775	2.04337554	6.76654588
C	3.78529079	3.72517720	4.44880230
H	3.70115017	1.93481852	3.26646778
C	3.30809677	4.40006257	5.57676407
H	1.98999303	4.31116423	7.29022808
H	4.53496225	4.18858429	3.80892601
H	3.68144121	5.39550657	5.81406191
C	2.68590912	-0.80620907	3.56857755
C	3.09980458	-0.34751213	2.30488152
C	3.10342070	-2.06489441	4.03323883

C	3.95032619	-1.13213413	1.52867410
H	2.72519691	0.59434656	1.91189684
C	3.93690174	-2.85270180	3.23771306
H	2.76806004	-2.43567195	4.99750359
C	4.36741322	-2.38440006	1.99217300
H	4.24556760	-3.83562217	3.59076702
H	5.02024840	-3.00162889	1.37587205
H	-0.06909911	2.54375269	4.11084002
H	4.26807544	-0.77630547	0.54979757
H	-0.84143067	4.43436355	3.75778461
H	-0.17125145	-0.24785252	3.07014754
H	-1.99811229	-4.74804212	3.77802854
H	-6.71627432	-0.01673200	3.88752382
H	-4.84754392	-1.75583852	5.43047582
H	-4.36054346	2.39610525	2.18384036
C	-2.55332465	3.16228111	3.17995938
C	-3.24876683	3.39514594	4.53545095
C	-1.53056984	4.29213913	2.91455619
C	-3.61034321	3.18533038	2.05081557
H	-3.99812098	2.61989520	4.73472851
H	-2.52998512	3.38792643	5.36595024
H	-3.75228648	4.37383846	4.52630123
C	-3.36920574	-0.05324942	4.11161085
C	-6.41004287	-2.18685770	4.00444420
C	-7.12871641	-0.88227208	4.42312506
C	-6.62760967	-2.42333775	2.49139101
C	-6.99234874	-3.36346734	4.81910133
H	-7.01773443	-0.70194709	5.50298045
H	-8.20175531	-0.95585635	4.19306894
H	-6.08740238	-3.31701196	2.15984439
H	-7.70191357	-2.55878393	2.29387880
H	-6.28439345	-1.56600068	1.89538389
H	-8.07851367	-3.42711590	4.65772639
H	-6.53664470	-4.31424760	4.51836724
H	-6.81747770	-3.21500988	5.89615088
C	-4.00720388	-3.28689703	4.16580974
O	-4.30539521	-4.24891180	3.43802587
N	-2.82840395	-3.19819384	4.83873042
H	-2.65024936	-2.33775543	5.37120749
H	-4.12512082	4.15697136	2.04215908
H	-3.13758879	3.03743431	1.06811849
H	-0.93630592	4.08532417	2.01124465

H	-2.06237679	5.24217310	2.75953043
C	-0.39561683	-3.47762199	4.49571709
H	-2.70024273	-5.66502415	5.97129273
C	-1.73046638	-5.15729538	5.87827388
H	-1.52853958	-4.61222227	6.81270718
H	0.37081393	-4.20725105	4.19724066
H	-0.06691043	-3.01670568	5.43482458
H	-0.46596546	-2.70102102	3.72273272
C	-2.04906560	-2.18631241	1.10316327
C	-2.52678165	-1.51119925	-0.04759559
H	-4.72406768	1.76476333	-0.70125810
H	-5.89334475	0.44478844	-0.97882832
H	-2.65948706	-2.11582191	1.99995303
C	-3.56514359	-0.57152282	-0.03050634
H	-1.22062332	-4.03100474	1.85241683
C	0.05532969	-3.16045969	0.32796794
O	-4.22553077	-0.13589712	0.95864614
O	-3.87261460	-0.06754575	-1.30734445
C	-4.95820044	0.89806924	-1.33031436
H	-5.05294247	1.20009118	-2.37789867
C	-1.33144045	-3.50918769	0.89592145
H	-1.90312867	-4.15696746	0.21510424
H	-2.08389893	-1.74002587	-1.01641156
H	-0.03916351	-2.83671654	-0.71919459
H	0.74293559	-4.01885120	0.35419381
C	0.55590535	-1.99738866	1.22141915
O	-0.56427963	-1.36027515	1.84174072
H	1.13743667	-1.27696842	0.62131495
H	1.22710479	-2.40761447	1.99294090

TS–Mode A–LA2–RA1–*R*

COSMO(THF)-ZORA-M06-2X/TZ2P//COSMO(THF)-ZORA-BLYP-D3(BJ)/DZP

E = -20669.45

G = -20151.58

COSMO(THF)-ZORA-BLYP-D3(BJ)/DZP

E = -14455.97

G = -13938.10

N_{imag} = 1, 54*i* cm^{-1}

C	-4.70531648	-0.59944045	0.77543396
N	-4.44649942	-0.08422575	2.13528564
H	-3.48243240	0.27604182	2.22718302
C	-8.20271248	0.86874818	0.02732413

S	-6.66522615	-0.82361933	3.57363363
N	-4.26592222	0.10196902	4.38341446
H	-3.32321471	0.44601444	4.14938279
C	-4.63884152	0.08665270	5.78811280
H	-5.67722872	-0.26932797	5.81554920
H	-2.66237102	2.39291056	6.17827927
C	-4.70811227	1.53663878	6.35309467
N	-3.68795552	2.48431378	5.84226911
H	-4.66936928	1.50004108	7.44733968
P	-4.12721768	3.99994079	5.40614205
C	-4.81617811	5.05718370	6.72390322
C	-5.36237414	4.49466363	7.88998328
C	-4.80106709	6.45532814	6.55976295
C	-5.89469178	5.32542937	8.88038179
H	-5.37227247	3.41900738	8.03559108
C	-5.33965235	7.27794778	7.55003713
H	-4.36239131	6.89821124	5.66637786
C	-5.88685767	6.71403489	8.71030962
H	-6.31357231	4.88474768	9.78531710
H	-5.32601686	8.35983070	7.41919098
H	-6.30177122	7.35866429	9.48431162
C	-5.36060834	3.91690039	4.08534612
C	-6.50979147	4.72313420	4.08950890
C	-5.08251047	3.08429389	2.98713171
C	-7.36436990	4.70908610	2.98363412
H	-6.73688162	5.35584847	4.94398368
C	-5.93070640	3.09067157	1.88279615
H	-4.20209509	2.45245754	3.00332223
C	-7.06669168	3.90717258	1.87756353
H	-8.25867183	5.33087725	2.98507748
H	-5.70745584	2.46437894	1.02552269
H	-7.72498544	3.91420943	1.01092183
C	-2.68043541	4.90540409	4.80670728
C	-2.56369988	5.27863011	3.46144106
C	-1.71421674	5.31218659	5.74355377
C	-1.46896955	6.04233796	3.05036897
H	-3.31285369	4.97789829	2.73588767
C	-0.63284687	6.08279546	5.32589342
H	-1.81423785	5.03570322	6.78958715
C	-0.50657503	6.44484258	3.97862501
H	0.11554309	6.40135553	6.04981521
H	0.34389185	7.04469227	3.65559142

H	-5.69702547	1.92554673	6.07313292
H	-1.37100477	6.31875449	2.00174834
H	-3.82107677	-0.01970805	8.57410352
C	-9.22221602	1.11717038	1.14539291
H	-7.82686341	1.83166443	-0.35536858
H	-4.06222039	-2.69773753	2.61233950
H	-3.81671422	-0.24833669	0.23867141
H	-3.93336165	-2.43573989	4.95600280
C	-3.80268580	-0.97603165	6.58763589
C	-2.28345934	-0.76839488	6.44399648
C	-4.19602251	-0.92701037	8.08082929
C	-4.17475461	-2.36899175	6.02428210
H	-1.97125295	-0.87711207	5.39557670
H	-1.96160396	0.22350519	6.78492854
H	-1.75076348	-1.53342061	7.03020672
C	-5.05761337	-0.26067595	3.34004127
C	-4.68020062	-2.17100713	0.57640839
C	-3.68273917	-2.77688650	1.58644568
C	-6.05196769	-2.86335481	0.72436342
C	-4.16619971	-2.41761054	-0.86352351
H	-2.70828252	-2.26850769	1.54041074
H	-3.52772331	-3.84160735	1.35812425
H	-6.77327399	-2.47887935	-0.00873648
H	-5.92711793	-3.94258244	0.54711870
H	-6.46597071	-2.71326832	1.72666658
H	-4.15680238	-3.49601377	-1.07857821
H	-4.81184658	-1.92274337	-1.60249884
H	-3.14357704	-2.03120864	-0.98908105
C	-5.83942903	0.13197535	0.01631584
O	-5.58759284	0.67981853	-1.08053303
N	-7.05234505	0.14564584	0.59358814
H	-7.12955110	-0.24047240	1.55337301
H	-3.60899284	-3.14928586	6.55450688
H	-5.24871106	-2.57262024	6.14168359
H	-5.28896881	-0.96750600	8.20861497
H	-3.76080505	-1.79037159	8.60515257
C	-8.81097226	0.08897045	-1.15057916
H	-8.05035923	-0.08820463	-1.92130125
H	-9.63960547	0.65857759	-1.59614657
H	-9.19772199	-0.88013967	-0.80276346
H	-8.76430894	1.65927206	1.98248765
H	-9.61461651	0.16193695	1.52569098

H	-10.06557705	1.70661002	0.76084720
C	-0.05248501	1.79673423	5.03483782
C	-0.40935358	2.52206998	3.88605073
H	-3.26932229	2.21669935	0.96270429
H	-1.80597814	1.37208958	0.38302279
H	-0.35742604	0.75159751	5.03174433
C	-1.30441488	2.01368067	2.92864721
H	1.64047380	1.16621084	6.21615106
C	0.97634067	3.11756641	6.90310617
O	-1.92861614	0.91127900	2.95790129
O	-1.45580110	2.86679370	1.82775043
C	-2.21132550	2.33739590	0.71127167
H	-2.11045260	3.07897255	-0.08725597
C	1.24748838	2.09635958	5.77659200
H	2.00009111	2.48232158	5.07418106
H	-0.00420879	3.51771045	3.71882368
H	0.82253642	4.10990390	6.45873502
H	1.81193744	3.17661822	7.61792168
C	-0.32856229	2.63448262	7.55542031
O	-1.18128993	2.22388674	6.51146402
H	-0.79400480	3.44145766	8.15456315
H	-0.10293494	1.79321755	8.24752808

TS–Mode A–LA2–RA2–*R*

COSMO(THF)-ZORA-M06-2X/TZ2P//COSMO(THF)-ZORA-BLYP-D3(BJ)/DZP

E = -20670.10

G = -20155.13

COSMO(THF)-ZORA-BLYP-D3(BJ)/DZP

E = -14449.61

G = -13934.64

N_{imag} = 1, 323*i* cm^{-1}

C	-5.88343258	0.88355602	2.30534127
N	-4.99405622	1.15950075	3.46054793
H	-4.59663086	2.10606769	3.36293177
C	-8.72954209	-1.00674951	4.10175049
S	-4.51091567	-1.28919438	4.58114719
N	-3.04486946	1.00777815	4.58714913
H	-2.91602443	1.90939361	4.09576296
C	-1.87845119	0.60572016	5.39160544
H	-1.20384316	1.45732878	5.23680878
C	-8.70883811	-1.33720051	5.59916037
C	-1.16527057	-0.60934996	4.77516496

N	-0.69676503	-0.21295001	3.41833580
H	-1.86523686	-1.44397711	4.69708940
P	0.06919184	-1.23638575	2.39259707
C	-1.03519519	-2.40052565	1.55385126
C	-2.20201207	-2.87975414	2.17599489
C	-0.68069557	-2.84247396	0.26165109
C	-3.00202675	-3.80833073	1.50934192
H	-2.52183956	-2.51199649	3.15026829
C	-1.48993785	-3.77055789	-0.39581516
H	0.21154428	-2.45462421	-0.23309211
C	-2.64735337	-4.25776130	0.22972611
H	-3.91529218	-4.16595438	1.98808624
H	-1.21970245	-4.10606477	-1.40029194
H	-3.27955040	-4.97831678	-0.28813381
C	1.36969486	-2.19960609	3.20837684
C	1.51920337	-3.57972615	2.99361778
C	2.21812057	-1.52493642	4.10713754
C	2.52653348	-4.27753407	3.66662220
H	0.85326475	-4.10568412	2.31271345
C	3.22129697	-2.22890013	4.77268077
H	2.09235617	-0.45805296	4.28250633
C	3.37572122	-3.60463319	4.55168792
H	2.64421175	-5.34837369	3.50131902
H	3.87942048	-1.70727613	5.46678323
H	4.15729021	-4.15338845	5.07629680
C	0.79670397	-0.18681857	1.11484593
C	2.16060475	-0.26368426	0.79905029
C	-0.05722193	0.66926536	0.39712276
C	2.67204508	0.53020067	-0.23171051
H	2.81706653	-0.93474425	1.34883780
C	0.46377760	1.45657299	-0.62756009
H	-1.11402799	0.71736383	0.64123050
C	1.82655104	1.38624441	-0.94307022
H	-0.19217671	2.12949870	-1.17810381
H	2.22942064	2.00448500	-1.74445179
H	-0.32152717	-0.91492288	5.41012270
H	3.73169327	0.47702653	-0.47756177
H	0.03731244	0.06581870	7.34566429
H	-0.28959246	0.77229255	3.39624190
H	-9.51308298	-0.26164361	3.89780262
H	-6.64945681	-1.62314201	1.04980065
H	-6.05013649	1.89655078	1.91514946

H	-4.07419100	1.45042125	6.95088237
C	-2.08105730	0.54015348	6.94795782
C	-2.58970679	-0.82253654	7.46437840
C	-0.70518791	0.83692898	7.59664737
C	-3.07303273	1.65179857	7.35361756
H	-3.57444480	-1.05766389	7.04774267
H	-1.90303313	-1.63824913	7.19490398
H	-2.65800379	-0.79270778	8.56288652
C	-4.15060741	0.34167135	4.17836279
C	-5.26027936	0.07071670	1.08783478
C	-5.57087207	-1.44040772	1.15676155
C	-5.88090876	0.63853893	-0.21161701
C	-3.73322165	0.29644077	1.04511409
H	-5.23806334	-1.86887326	2.10590987
H	-5.05318461	-1.95765062	0.33589684
H	-5.60058599	1.69439438	-0.34893967
H	-5.51294992	0.06850660	-1.07830719
H	-6.97565325	0.57245312	-0.18116098
H	-3.33441583	-0.12942834	0.11241568
H	-3.48802018	1.36774957	1.08257232
H	-3.22317068	-0.19357937	1.88051416
C	-7.31192992	0.41003970	2.65233647
O	-8.26579391	0.76514431	1.92557962
N	-7.44917606	-0.37823485	3.73546609
H	-6.58489940	-0.75085339	4.16829287
H	-3.14183093	1.71024499	8.44977159
H	-2.74406627	2.63102328	6.97472492
H	-0.31492208	1.81084135	7.26607206
H	-0.80454387	0.85872750	8.69169364
C	-8.99171627	-2.24933385	3.23207141
H	-7.91077391	-2.06095127	5.82386778
H	-9.66792268	-1.77848573	5.90310001
H	-8.53297182	-0.43082452	6.19514737
H	-8.20452210	-3.00010635	3.39592326
H	-8.99925465	-1.97356622	2.16963238
H	-9.96347790	-2.69795385	3.48586359
C	-0.29314248	3.45973068	2.69073663
C	-1.07846750	4.43785115	3.41409277
H	-4.61752051	4.01873412	5.18600683
H	-5.05205098	5.03261627	3.78048801
H	-0.91839422	2.85884535	2.02153932
C	-2.44815906	4.26813373	3.57957188

H	1.25528550	3.30316239	1.17444581
C	2.10401332	3.69818420	3.12379296
O	-3.18646733	3.37119221	3.05014451
O	-3.05032675	5.21833068	4.43778180
C	-4.45833822	4.99164097	4.70269631
H	-4.75960091	5.79755489	5.37996860
C	1.02371398	3.92258070	2.05103737
H	0.95923928	4.97057738	1.72893427
H	-0.57568176	5.23386323	3.96284509
H	2.03353179	4.46765302	3.90750538
H	3.12343070	3.70975923	2.71204188
C	1.72414921	2.32816687	3.69348400
O	0.28060637	2.28307693	3.69803288
H	2.08892870	2.18357756	4.72294198
H	2.13644570	1.52797767	3.05322445

TS–Mode B–LA1–RA1–*R* (TS-(*R*))

COSMO(THF)-ZORA-M06-2X/TZ2P//COSMO(THF)-ZORA-BLYP-D3(BJ)/DZP

E = -20680.02

G = -20162.25

COSMO(THF)-ZORA-BLYP-D3(BJ)/DZP

E = -14461.58

G = -13943.81

N_{imag} = 1, 312*i* cm^{-1}

C	-6.21301156	-1.66245794	3.77224319
N	-5.02323945	-0.82424391	3.63535172
H	-4.45315277	-0.90110994	2.77097924
C	-4.86811526	-4.30007128	6.20408596
S	-5.17472204	0.11488987	6.17946684
N	-3.36097341	0.61773222	4.20825050
H	-3.02408778	0.36127771	3.25861630
C	-2.40107032	1.22768360	5.11865071
H	-2.90906557	1.29872121	6.08411678
H	-2.44400235	6.18895436	0.97758695
C	-2.05710227	2.64975626	4.63848094
N	-3.22519084	3.51640574	4.39182899
H	-1.52058512	2.61088045	3.68215843
P	-4.13649113	4.26386582	5.52846278
C	-5.81389175	3.61203545	5.68714858
C	-6.41630294	3.00194127	4.57561721
C	-6.52958398	3.79518596	6.88127071
C	-7.74791480	2.59621514	4.66425397

H	-5.85407948	2.83735011	3.65851235
C	-7.85208735	3.35756594	6.96478092
H	-6.05830173	4.26856366	7.74066468
C	-8.46286577	2.76669198	5.85302735
H	-8.22507871	2.13643323	3.80382144
H	-8.40644135	3.48480477	7.89384558
H	-9.49863106	2.43419154	5.91616414
C	-4.29947531	6.00771477	5.04867287
C	-3.14956242	6.69489181	4.61917751
C	-5.54005711	6.66420710	5.08036709
C	-3.24147705	8.03116479	4.23357682
H	-2.19378665	6.17860716	4.56893051
C	-5.62516426	8.00217838	4.68522282
H	-6.43525853	6.13339394	5.39415565
C	-4.47950700	8.68497006	4.26460894
H	-2.35032904	8.55998196	3.89833546
H	-6.58974592	8.50759708	4.69963351
H	-4.55126742	9.72626357	3.95276045
C	-3.30311000	4.13309916	7.13071520
C	-3.50021396	2.97128965	7.89933842
C	-2.43549653	5.14178559	7.58080796
C	-2.81148149	2.81656041	9.10200267
H	-4.17454337	2.18961033	7.55042244
C	-1.74762787	4.97285190	8.78499222
H	-2.30213525	6.05604545	7.00869724
C	-1.93174988	3.81138484	9.54245712
H	-1.07199477	5.75311247	9.13257572
H	-1.39338588	3.68505094	10.48120569
H	-1.39878849	3.13695591	5.36536003
H	-2.96102790	1.91469972	9.69457027
H	0.17293999	1.90425197	6.17509232
C	-3.08075602	1.92564574	0.27070040
H	-5.32810822	-5.18397197	5.73817254
H	-6.72263805	0.52481598	2.22077773
H	-6.80255542	-1.22572267	4.59036781
H	-2.16528336	-1.56912472	4.96935747
C	-1.12965843	0.33175797	5.33340093
C	-0.29147133	0.21155781	4.04020458
C	-0.27223640	0.94343689	6.46672049
C	-1.59333362	-1.07662419	5.76502681
H	-0.90368337	-0.13950048	3.19842451
H	0.16435796	1.17009086	3.75546402

H	0.52280388	-0.51276189	4.19165391
C	-4.48084253	-0.04458377	4.60625833
C	-7.09478699	-1.62406540	2.48831083
C	-7.57130647	-0.16773924	2.29242583
C	-6.32711364	-2.08131749	1.22846876
C	-8.32849212	-2.52647212	2.71604123
H	-8.19677961	0.14424595	3.13910012
H	-8.16885549	-0.08512306	1.37271384
H	-5.96663526	-3.10867873	1.34410609
H	-6.99095755	-2.02488387	0.35258806
H	-5.46882841	-1.43081143	1.03250243
H	-9.01926979	-2.43503292	1.86499466
H	-8.03488581	-3.57858689	2.81503171
H	-8.86942796	-2.22501050	3.62648562
C	-5.76560990	-3.08248451	4.23538344
O	-5.64349997	-4.06151311	3.47754632
N	-5.49321146	-3.12876700	5.56492950
H	-5.44760605	-2.22288314	6.05983636
H	-0.72083215	-1.70456256	5.99731524
H	-2.23690026	-1.02327041	6.65353559
H	-0.87608350	1.10697717	7.37132171
H	0.55133901	0.26061783	6.72313541
C	-3.35360340	-4.33151120	5.93785999
C	-3.58721605	-0.30206679	-0.80532355
H	-3.51704159	0.23028224	-1.76412086
H	-2.22345228	2.01847562	-0.39517329
H	-1.42184090	-0.44660265	-0.63103133
H	-2.18408985	-1.97797378	-1.14304532
C	-2.46045972	-1.47496538	0.98456252
O	-3.11978928	-0.38108180	1.62975439
H	-1.48507771	-1.66597297	1.46278640
H	-3.07001127	-2.39546056	1.06942334
C	-5.19477078	-4.27958701	7.70227624
H	-2.37647392	5.25075119	2.50207909
H	-3.94673481	5.51791590	1.70554290
H	-4.77916025	0.74934134	0.76163831
C	-3.42703083	3.03593276	1.04354571
H	-4.44020122	-0.99372418	-0.85950983
C	-2.30091018	-1.09548405	-0.49664026
O	-4.32695709	3.12777543	1.93357809
O	-2.64413980	4.17254019	0.72425717
C	-2.87738900	5.35114528	1.53413730

C	-3.77261611	0.68469109	0.34454001
H	-6.28162208	-4.28058327	7.86455635
H	-4.77180645	-3.38088595	8.17707567
H	-4.76383970	-5.16082849	8.19689128
H	-3.15623378	-4.30767171	4.85801638
H	-2.90932850	-5.24659995	6.35663477
H	-2.86922034	-3.46238143	6.40142236
H	-3.67828966	3.38298603	3.44658515

TS–Mode B–LA1–RA2–*R*

COSMO(THF)-ZORA-M06-2X/TZ2P//COSMO(THF)-ZORA-BLYP-D3(BJ)/DZP

E = -20666.40

G = -20149.36

COSMO(THF)-ZORA-BLYP-D3(BJ)/DZP

E = -14450.13

G = -13933.09

$\boldsymbol{N}_{\mathrm{imag}}$ = 1, 45*i* cm^{-1}

C	-4.70575947	-2.59626889	4.09966970
N	-4.10071213	-1.41845154	3.48353796
H	-4.18833244	-1.27487658	2.45630972
C	-1.45463796	-4.57255707	4.42803139
S	-2.93041856	-0.62979495	5.80585381
N	-3.04351782	0.57925169	3.34980701
H	-3.46807506	0.53299382	2.39598673
C	-1.99008943	1.59502461	3.48358836
H	-1.68477856	1.76406670	2.44066137
H	-0.41829583	-6.20485921	5.43805955
C	-0.71858069	1.07015090	4.19778936
N	0.45346724	1.25796723	3.31001827
H	-0.83546001	0.00971665	4.43696253
P	1.96135519	1.57110984	3.84701420
C	2.65006060	0.30561659	4.94397739
C	2.12083041	-0.99340237	4.88285942
C	3.72638675	0.59478932	5.80100643
C	2.67344467	-2.00225314	5.67331895
H	1.28272784	-1.20759191	4.22723799
C	4.27633342	-0.42166821	6.58346511
H	4.12957931	1.60439635	5.85659459
C	3.75140109	-1.71796336	6.51863518
H	2.26306057	-3.00852437	5.63280654
H	5.11172221	-0.20181993	7.24654242
H	4.18020669	-2.50811118	7.13368725

C	1.98485112	3.12152548	4.77951766
C	1.58451232	3.12425936	6.12813547
C	2.26064504	4.33123162	4.12103872
C	1.43944064	4.33728269	6.80307838
H	1.38454511	2.18858667	6.64658773
C	2.12072166	5.53806892	4.80741893
H	2.58237400	4.33219127	3.08214613
C	1.70262264	5.54237885	6.14287638
H	1.11899525	4.34051863	7.84390475
H	2.33611312	6.47635392	4.29813457
H	1.58631285	6.48741882	6.67203475
C	2.96805096	1.67425794	2.35217134
C	2.44008879	2.30426491	1.21066890
C	4.24644143	1.09355994	2.31752459
C	3.19611316	2.34884292	0.03956275
H	1.43959491	2.72864512	1.23052518
C	4.99286493	1.14368690	1.13876375
H	4.64626156	0.58567655	3.19175880
C	4.46930808	1.76932426	0.00236303
H	5.97983566	0.68466224	1.10622785
H	5.05250937	1.79976329	-0.91722614
H	-0.54619216	1.58773743	5.14653029
H	2.78539622	2.82791859	-0.84796093
H	-0.58986510	3.83846905	4.58331657
H	0.33620501	0.81686845	2.35130543
H	-1.63442670	-5.14069401	3.50459168
H	-6.57409449	-0.62146098	3.54601610
H	-4.69684760	-2.40590831	5.18244013
H	-4.51957159	2.68282323	3.11035618
C	-2.52689685	2.98079436	3.98036576
C	-3.03647491	2.91973149	5.43230596
C	-1.39391004	4.02503612	3.86411606
C	-3.68876286	3.39834149	3.04930923
H	-3.83537297	2.17501048	5.53380405
H	-2.23577737	2.64369611	6.13178339
H	-3.42606805	3.90525627	5.73038097
C	-3.36906309	-0.47254557	4.13526507
C	-6.19431513	-2.77929290	3.68343912
C	-6.95324265	-1.49492709	4.09322562
C	-6.36423086	-3.00607066	2.16558183
C	-6.77156315	-3.97947423	4.46687236
H	-6.84012194	-1.29796515	5.17006047

H	-8.02484441	-1.60619886	3.87175495
H	-5.85594250	-3.92354531	1.85187739
H	-7.43478464	-3.07943709	1.91990881
H	-5.94989244	-2.16900368	1.59476719
H	-7.84258345	-4.09331252	4.24291649
H	-6.25858657	-4.91004193	4.19346945
H	-6.66525877	-3.82496776	5.55182163
C	-3.76128982	-3.81194657	3.85792545
O	-3.98332510	-4.73245306	3.05135256
N	-2.64288723	-3.72896645	4.62234338
H	-2.53765100	-2.87623163	5.19586456
H	-4.06398307	4.39167886	3.33575518
H	-3.35407829	3.44479872	2.00142403
H	-0.95548316	4.02663260	2.85521729
H	-1.79112823	5.03045327	4.06731428
C	-0.22906590	-3.66487828	4.24854563
H	-2.18563709	-6.18679976	5.69556891
C	-1.29143143	-5.55568120	5.59863934
H	-1.14761065	-5.00770899	6.54239557
H	0.67839707	-4.26793947	4.10192546
H	-0.09095640	-3.04525605	5.14642343
H	-0.35952192	-3.00869578	3.37892204
C	-2.70053348	-0.46954021	-0.29825691
C	-1.74505154	-1.37148911	0.20102738
H	2.07312109	-2.27336124	2.17809511
H	1.88878474	-0.64114984	1.45756355
H	-2.37494102	0.57193061	-0.28885818
C	-0.54547391	-0.92039815	0.78157200
H	-3.61469590	-0.01734274	-2.18976645
C	-5.07838281	-1.04311006	-0.96165909
O	-0.14729995	0.27486573	0.89618613
O	0.26877390	-1.97625088	1.23052214
C	1.69368621	-1.70593892	1.32334578
H	2.18435345	-2.05225432	0.40290898
C	-3.62782358	-0.84111825	-1.45862712
H	-3.25511837	-1.74119494	-1.96721108
H	-1.90465271	-2.44606889	0.12250656
H	-5.18343513	-2.05196648	-0.53446781
H	-5.81239944	-0.92958307	-1.77477538
C	-5.24709493	-0.01464952	0.16159169
O	-4.10529182	-0.15211528	0.98195751
H	-6.16713081	-0.20119398	0.74459618

H	-5.32642594	1.00696415	-0.26809149

TS–Mode B–LA2–RA1–*R*

COSMO(THF)-ZORA-M06-2X/TZ2P//COSMO(THF)-ZORA-BLYP-D3(BJ)/DZP

E = -20672.93

G = -20156.33

COSMO(THF)-ZORA-BLYP-D3(BJ)/DZP

E = -14456.33

G = -13939.73

$\boldsymbol{N}_{\mathrm{imag}}$ = 1, 303*i* cm^{-1}

C	-4.76979380	0.55971416	0.34917117
N	-4.41367563	0.16265531	1.71911962
H	-3.38003560	0.04248142	1.85302527
C	-7.06737513	3.60662784	0.99661421
S	-6.81384938	-0.09548191	3.03692500
N	-4.26148778	-0.30919665	3.91484321
H	-3.25376019	-0.27829503	3.69478045
C	-4.65831667	-0.47225008	5.30125091
H	-5.75307714	-0.42316195	5.31191283
H	-3.80843563	2.37412019	4.82838562
C	-4.12851565	0.72147098	6.12585198
N	-4.53488225	2.00526483	5.49707550
H	-3.03274304	0.70106483	6.20447232
P	-5.31510628	3.14935023	6.38600160
C	-4.32872435	3.92390113	7.70319015
C	-3.75189561	3.11431164	8.70095197
C	-4.04720543	5.29927019	7.66943125
C	-2.89594507	3.67852379	9.64591358
H	-3.96506577	2.04876667	8.74299594
C	-3.18626302	5.85566993	8.61851779
H	-4.48516586	5.93014964	6.90006219
C	-2.60778548	5.04803075	9.60151480
H	-2.44900635	3.04859929	10.41369378
H	-2.96386026	6.92141899	8.58403793
H	-1.93187815	5.48440460	10.33600536
C	-6.73687652	2.34037075	7.14671622
C	-7.09062309	2.56924208	8.48592543
C	-7.50748922	1.48523512	6.33717495
C	-8.21009603	1.92507572	9.01957750
H	-6.50017286	3.23952325	9.10627705
C	-8.61750402	0.84351359	6.88420251
H	-7.23188643	1.30517343	5.29797106

C	-8.96796310	1.06013483	8.22294381
H	-8.48588617	2.09582716	10.05917397
H	-9.20824515	0.17016948	6.26400026
H	-9.83522981	0.55424592	8.64597889
C	-5.86964390	4.47512491	5.29276085
C	-7.07515250	5.12714111	5.60477378
C	-5.07543828	4.91204827	4.21836799
C	-7.48175930	6.22316340	4.84212517
H	-7.69448442	4.77738044	6.42858670
C	-5.50013929	6.00632205	3.46206398
H	-4.14045459	4.41024547	3.97521688
C	-6.69383664	6.66410466	3.77438917
H	-4.89260557	6.34455192	2.62342742
H	-7.01724183	7.51623247	3.17725298
H	-4.53729784	0.65493044	7.13853282
H	-8.41884232	6.72574016	5.07778771
H	-4.17911094	-1.33079814	8.00722254
C	-7.96735296	3.67525934	2.23501746
H	-6.29231679	4.38715433	1.06025111
H	-5.49268855	-2.13139367	0.92442864
H	-3.78007042	0.66069749	-0.11276297
H	-4.62169068	-2.87751540	3.96014248
C	-4.24240275	-1.86855160	5.86794990
C	-2.71173960	-2.07280984	5.80884744
C	-4.73534016	-1.99165579	7.32807800
C	-4.93602208	-2.95032979	5.00952333
H	-2.33352408	-1.97269283	4.78174135
H	-2.17753681	-1.35157908	6.44323962
H	-2.45682824	-3.08213182	6.16460790
C	-5.09530869	-0.06178446	2.86409183
C	-5.51549553	-0.51272668	-0.55185053
C	-5.10275363	-1.92253935	-0.08006450
C	-7.05419124	-0.38812359	-0.54012097
C	-5.02179661	-0.29342207	-2.00259362
H	-4.00824369	-2.02706343	-0.05239313
H	-5.50255716	-2.67558959	-0.77549402
H	-7.37655207	0.58323820	-0.93839693
H	-7.48330585	-1.17655541	-1.17745439
H	-7.44997618	-0.49533349	0.47532971
H	-5.53565111	-0.98757507	-2.68367561
H	-5.22328779	0.73438156	-2.33458470
H	-3.93861376	-0.47265124	-2.08079742

C	-5.33958954	1.99486357	0.21431004
O	-4.83825628	2.77912422	-0.62019366
N	-6.38186483	2.30387692	1.00474186
H	-6.71962235	1.58216958	1.66395070
H	-4.67768773	-3.95167457	5.38385422
H	-6.02864350	-2.83367859	5.04413263
H	-5.80557076	-1.74532278	7.40371571
H	-4.59706415	-3.02249752	7.68545919
C	-7.86498318	3.80508701	-0.30384686
H	-7.20333104	3.72079630	-1.17455509
H	-8.33524181	4.79959923	-0.31067080
H	-8.65497339	3.04349503	-0.38166574
H	-7.38342723	3.53270572	3.15067648
H	-8.73842560	2.89111247	2.18969152
H	-8.46644500	4.65034427	2.28685435
C	-1.22481941	1.46957629	1.94326328
C	-0.57989093	2.10918769	3.03437375
H	-1.91792577	3.50828089	6.58217927
H	-1.88025330	4.93768833	5.51597832
H	-2.20672237	1.88957194	1.71358717
C	-1.32356065	2.84421460	3.96386098
H	-1.05408784	0.95389323	-0.15150993
C	0.11268557	-0.41126379	1.06892370
O	-2.56971390	3.06784763	3.94586867
O	-0.53556702	3.39536868	4.99678076
C	-1.26953517	4.15905067	5.98657384
H	-0.51096981	4.61265656	6.63208414
C	-0.41066472	1.00197814	0.74113293
H	0.40892461	1.70282502	0.52995301
H	0.48393858	1.95736796	3.21311182
H	0.90246779	-0.34715352	1.83366825
H	0.52223046	-0.92480591	0.18650511
C	-1.11999780	-1.12427515	1.64526109
O	-1.82155319	-0.16223621	2.43769678
H	-0.83954214	-1.99014013	2.26807666
H	-1.75686623	-1.49256292	0.81625639

TS–Mode B–LA2–RA2–*R*

COSMO(THF)-ZORA-M06-2X/TZ2P//COSMO(THF)-ZORA-BLYP-D3(BJ)/DZP

E = -20668.22

G = -20152.11

COSMO(THF)-ZORA-BLYP-D3(BJ)/DZP

E = -14448.44

G = -13932.33

N_{imag} = 1, 309*i* cm^{-1}

C	-6.02772541	0.20132743	2.11887739
N	-4.94514258	0.66501508	3.01339924
H	-4.78922157	1.68256902	2.93800207
C	-8.04875503	-1.83898684	4.70710641
S	-3.84379876	-1.65146078	3.97023048
N	-3.11810322	0.94484116	4.29858939
H	-3.32569822	1.92112869	4.00640904
C	-2.18485939	0.82389429	5.42382948
H	-1.73196287	1.82639577	5.45492027
C	-7.55915836	-2.00803726	6.15166554
C	-1.03162479	-0.14001780	5.10962412
N	-0.32215550	0.36847071	3.90573394
H	-1.42864561	-1.14864695	4.94728514
P	0.85277226	-0.55596640	3.20366754
C	0.25016145	-2.09725029	2.45597772
C	-1.00567521	-2.06317219	1.82336815
C	1.00515381	-3.28308793	2.47511936
C	-1.50265494	-3.21686123	1.21800586
H	-1.57194687	-1.13987534	1.79350087
C	0.50018006	-4.43029582	1.85861888
H	1.97614369	-3.31362570	2.96502051
C	-0.75290619	-4.39807951	1.23320273
H	-2.48146558	-3.19151673	0.74224306
H	1.08290810	-5.35043257	1.87056678
H	-1.14510594	-5.29796426	0.76016295
C	2.07327234	-1.00099488	4.46273060
C	1.76486407	-1.98611378	5.41870189
C	3.30050242	-0.32069635	4.51781144
C	2.68153226	-2.27934983	6.42877339
H	0.82126310	-2.52566804	5.37123966
C	4.21389403	-0.62536553	5.53030682
H	3.54232733	0.43414615	3.77259707
C	3.90449566	-1.60003233	6.48459053
H	2.44301492	-3.04080286	7.16998537
H	5.16809814	-0.10207900	5.57089788
H	4.61949227	-1.83467794	7.27224786
C	1.61718593	0.45768198	1.92186498
C	1.49563951	1.85576814	1.95546267
C	2.32515829	-0.17331735	0.88588091

C	2.06642147	2.61737949	0.93652918
H	0.93195990	2.33684894	2.74918985
C	2.91194384	0.60091694	-0.11652665
H	2.39640822	-1.25877412	0.84663956
C	2.77720336	1.99385880	-0.09505147
H	3.45701668	0.11498661	-0.92474847
H	3.21910750	2.59367651	-0.88999028
H	-0.32897665	-0.15052494	5.95150222
H	1.94300444	3.69922727	0.94357074
H	-0.90410421	1.05571946	7.83586717
H	-0.95733667	0.75312275	3.15442090
H	-8.92797340	-1.17838604	4.68837701
H	-6.71131294	-2.47389449	1.30493371
H	-6.41397754	1.15524602	1.73762027
H	-4.92422029	0.62885710	6.21052990
C	-2.82598512	0.61088268	6.85590102
C	-2.88059002	-0.86940119	7.29852359
C	-1.94991391	1.39626535	7.86208514
C	-4.25543455	1.19381444	6.87168210
H	-3.49333322	-1.46075035	6.61033214
H	-1.87807346	-1.31831217	7.34420335
H	-3.31505486	-0.92710837	8.30821652
C	-3.97790147	0.04523125	3.75279455
C	-5.63947464	-0.62057092	0.81679642
C	-5.70289201	-2.15173472	1.00870727
C	-6.65456416	-0.22772286	-0.28441174
C	-4.22740245	-0.20389164	0.37240188
H	-4.99524053	-2.48031614	1.77548548
H	-5.45828682	-2.64396874	0.05485742
H	-6.59282634	0.84848676	-0.50685850
H	-6.43111425	-0.78341570	-1.20724864
H	-7.68208992	-0.45708735	0.02602859
H	-3.97957161	-0.70277068	-0.57602072
H	-4.15679864	0.88234617	0.21839846
H	-3.47491762	-0.47369590	1.11651705
C	-7.25456236	-0.39225295	2.85506889
O	-8.40487351	-0.13878738	2.43708114
N	-6.99980538	-1.15743527	3.93351738
H	-6.02039288	-1.45112795	4.09264151
H	-4.66042230	1.14750429	7.89330069
H	-4.26177624	2.24446014	6.54803205
H	-1.96524621	2.47394768	7.64010327

H	-2.32401667	1.25105566	8.88598345
C	-8.42262604	-3.18023470	4.05235848
H	-6.65487264	-2.63423056	6.18156951
H	-8.33347118	-2.49207269	6.76264082
H	-7.31685656	-1.03255276	6.59524003
H	-7.54859686	-3.84779292	4.02698663
H	-8.76944388	-3.01629757	3.02363200
H	-9.22496758	-3.67501468	4.61923908
C	-2.06636511	3.70187096	2.64808300
C	-1.83806628	3.39007758	1.28455455
H	-1.66750398	-0.10931652	-0.72408391
H	0.02784541	0.40170155	-0.51175857
H	-1.62484808	2.98551744	3.34328013
C	-1.56192715	2.08130786	0.86347726
H	-1.14763267	5.22578791	3.86981770
C	-3.29685282	5.62881558	3.73209925
O	-1.46273620	1.04209229	1.56695418
O	-1.37446342	1.97216891	-0.52475843
C	-0.93132989	0.66255015	-0.96998121
H	-0.82096572	0.74844469	-2.05596747
C	-1.95051392	5.15604507	3.12021571
H	-1.66922421	5.79689300	2.27377599
H	-1.94904404	4.16594171	0.52809482
H	-3.88323636	6.16309367	2.96957579
H	-3.14523206	6.30633682	4.58554959
C	-4.04110119	4.34862063	4.13974758
O	-3.77308594	3.37956085	3.12929511
H	-5.12864981	4.51839372	4.20889388
H	-3.69238720	4.00950708	5.13726315

TS–Mode A–LA1–RA2–*S*

COSMO(THF)-ZORA-M06-2X/TZ2P//COSMO(THF)-ZORA-BLYP-D3(BJ)/DZP

E = -20674.00

G = -20157.93

COSMO(THF)-ZORA-BLYP-D3(BJ)/DZP

E = -14455.58

G = -13939.51

$\boldsymbol{N}_{imag}$ = 1, 323*i* cm^{-1}

C	-1.29737585	-2.82083345	5.28140065
N	-1.26805625	-1.96497415	4.09125302
H	-0.94366319	-2.39866650	3.21242418
C	2.36397668	-2.29057600	6.19157487

S	-1.41098703	0.28250513	5.59537108
N	-0.90639083	-0.05276758	2.92206642
H	-0.69579686	-0.75364866	2.18482458
C	-0.95617927	1.33199986	2.43328979
H	-0.43105042	1.25855916	1.47287280
H	3.95432181	-3.02538947	7.48557886
C	-0.11972502	2.31785998	3.26648028
N	1.27310576	1.78723013	3.34916049
H	-0.52221199	2.42813053	4.27642129
P	2.48494702	2.73671538	3.93786366
C	2.03279087	3.30614351	5.58896047
C	1.28421549	2.45055964	6.41417371
C	2.48471224	4.54710539	6.07009922
C	0.98422352	2.84359439	7.71809916
H	0.90769845	1.50731468	6.02876664
C	2.18811827	4.92478553	7.38150863
H	3.06107105	5.21104944	5.42932361
C	1.44031239	4.07449641	8.20443096
H	0.38980121	2.18840555	8.35393511
H	2.53771106	5.88470999	7.75887237
H	1.20766985	4.37543343	9.22539823
C	2.86623694	4.20950204	2.94612537
C	1.88794455	5.21237878	2.80148213
C	4.07946717	4.30612474	2.24544782
C	2.11896969	6.28790822	1.94374016
H	0.96046256	5.16456979	3.36806089
C	4.30482153	5.38977508	1.39390822
H	4.84335092	3.54164940	2.36619663
C	3.32438234	6.37419585	1.23627530
H	1.36103877	7.06190691	1.83127126
H	5.24739312	5.46386387	0.85321255
H	3.50133613	7.21557604	0.56734107
C	3.98184724	1.72381937	4.02239658
C	4.21185215	0.69085667	3.09654787
C	4.91410635	1.99643934	5.03669003
C	5.38594960	-0.05840332	3.19433191
H	3.47975587	0.45207558	2.32244856
C	6.08479074	1.24004926	5.11943887
H	4.72305591	2.77948037	5.76801667
C	6.32149029	0.21406973	4.19863241
H	6.80469409	1.44589061	5.91059561
H	7.23203610	-0.38028612	4.26992630

H	-0.14480763	3.28650384	2.75099116
H	5.56424767	-0.86727386	2.48615446
H	-1.77560503	3.90468406	1.56293153
H	1.53362651	1.30743533	2.42780488
H	2.69262914	-2.89320991	5.33007864
H	-3.79544802	-2.61428291	4.17808975
H	-1.60802311	-2.16516915	6.10512411
H	-3.31656180	-0.14818771	2.00645168
C	-2.39541144	1.84403082	2.06432311
C	-3.21934372	2.28648480	3.29019622
C	-2.24667818	3.03451018	1.08585038
C	-3.13808386	0.70119494	1.33459236
H	-3.34349012	1.46257806	4.00150120
H	-2.73726889	3.11938866	3.82128151
H	-4.21336285	2.62580282	2.96028589
C	-1.18900940	-0.59732170	4.12666283
C	-2.36740028	-3.94432034	5.17557292
C	-3.74806250	-3.25542348	5.06875673
C	-2.15907380	-4.85516081	3.94774671
C	-2.31538726	-4.78234308	6.47254634
H	-3.94412765	-2.62883000	5.95147364
H	-4.54189074	-4.01325905	4.99694911
H	-1.19105898	-5.36323902	4.00235587
H	-2.95863816	-5.61062533	3.90988220
H	-2.20109161	-4.27796643	3.01375104
H	-3.12802746	-5.52357991	6.47289039
H	-1.35925618	-5.31369469	6.55978880
H	-2.44120175	-4.13971382	7.35778272
C	0.16057819	-3.26061616	5.57016087
O	0.64071389	-4.36463035	5.26176998
N	0.88945678	-2.26316556	6.13904959
H	0.42031883	-1.35554385	6.24861624
H	-4.10880307	1.06284611	0.96595131
H	-2.55815696	0.33682080	0.47302808
H	-1.64391963	2.75295806	0.20899600
H	-3.23838062	3.34913424	0.72941506
C	2.88297257	-0.85867749	6.03616783
H	2.44966877	-3.98443337	7.55380246
C	2.85600463	-2.96696471	7.48004508
H	2.53098515	-2.39174792	8.36010036
H	3.97721705	-0.85032957	5.95931630
H	2.59670921	-0.24063387	6.90015780

H	2.47105231	-0.40311762	5.12898075
C	1.12648751	-0.29867987	-0.25401725
C	1.53336901	-1.63336971	-0.01041671
H	0.70725540	-4.38751528	2.82032320
H	-0.32770939	-4.85340050	1.43793114
H	0.08975876	-0.09886802	0.02176391
C	0.80718803	-2.50351243	0.81542705
H	0.77012302	1.09775354	-1.87444762
C	2.83124569	1.25177265	-1.21181804
O	-0.30568204	-2.29463286	1.39231515
O	1.40706083	-3.76513613	0.92818072
C	0.70554369	-4.71469500	1.77541654
H	1.26271084	-5.65267141	1.67879356
C	1.57054117	0.41951612	-1.53444389
H	1.75799090	-0.31233779	-2.33369879
H	2.47772657	-1.99052640	-0.42057145
H	3.70659275	0.58745674	-1.12478031
H	3.03773229	2.01460526	-1.97726513
C	2.51306622	1.85977740	0.15770480
O	1.91664322	0.81408563	0.92123656
H	3.42773231	2.21809181	0.66047049
H	1.83001854	2.73013996	0.04807059

TS–Mode A–LA2–RA1–*S*

COSMO(THF)-ZORA-M06-2X/TZ2P//COSMO(THF)-ZORA-BLYP-D3(BJ)/DZP

E = -20672.40

G = -20154.22

COSMO(THF)-ZORA-BLYP-D3(BJ)/DZP

E = -14460.59

G = -13942.41

N_{imag} = 1, 44i cm^{-1}

C	-4.68339030	-2.45069467	-3.13212837
N	-4.05119402	-2.14262993	-1.83683294
H	-3.04080884	-1.96742031	-1.93034444
C	-7.55129836	0.14372447	-3.20672240
S	-6.11460380	-2.37152861	-0.05235269
N	-3.53664443	-1.64277508	0.31209938
H	-2.61716222	-1.43964300	-0.09622390
C	-3.64263547	-1.62102176	1.76183054
H	-4.68880208	-1.86790154	1.97877543
H	-1.79621004	0.82571892	1.20441546
C	-3.36290960	-0.17892389	2.33314365

N	-2.89936516	0.80603198	1.34715722
H	-2.58319754	-0.22456149	3.10035264
P	-3.73791181	2.04331632	0.71371846
C	-5.39926462	2.14548192	1.42609923
C	-6.25850494	1.04869660	1.23393826
C	-5.85148769	3.29003063	2.10116906
C	-7.55718415	1.09342239	1.73685726
H	-5.93097184	0.16001772	0.69669112
C	-7.16068991	3.32896973	2.59037918
H	-5.19728388	4.14813158	2.23411711
C	-8.01100715	2.23223302	2.41268202
H	-8.21618205	0.23873130	1.59119786
H	-7.51534601	4.21898793	3.10849266
H	-9.02978352	2.26729482	2.79744535
C	-3.97757927	1.94463658	-1.08039901
C	-4.90895913	2.78952614	-1.70973133
C	-3.21579739	1.04525925	-1.83421694
C	-5.07182962	2.72235589	-3.09339121
H	-5.51016710	3.48275446	-1.12254666
C	-3.39375857	0.97866919	-3.21675250
H	-2.49654977	0.38929898	-1.35943757
C	-4.32036526	1.81211185	-3.84636443
H	-5.80311016	3.36470278	-3.58238625
H	-2.81576721	0.26055903	-3.79604262
H	-4.47460573	1.73730526	-4.92141791
C	-2.82142380	3.57550338	1.03809896
C	-2.33156553	4.37655837	-0.00367564
C	-2.52199399	3.89487796	2.37516120
C	-1.55007195	5.49705214	0.29406331
H	-2.54457018	4.12120592	-1.03900825
C	-1.76189765	5.02638346	2.66472833
H	-2.86698839	3.24914122	3.18138777
C	-1.27228938	5.82596010	1.62392611
H	-1.53499409	5.27605357	3.70045478
H	-0.66449311	6.70101729	1.85174084
H	-4.27483798	0.17916283	2.82514249
H	-1.15726785	6.11078531	-0.51553654
H	-2.60325128	-1.82156170	4.41687173
C	-8.01621975	0.90825404	-1.96299439
H	-7.09737932	0.84261104	-3.92341325
H	-4.75783783	-4.67148089	-1.32373973
H	-3.82995704	-2.37256593	-3.81612852

H	-3.06257793	-4.14939299	0.75832224
C	-2.74981182	-2.73496640	2.40818187
C	-1.25532356	-2.50796536	2.08798612
C	-2.97931052	-2.73529937	3.93752534
C	-3.18896444	-4.10679219	1.84741639
H	-1.04952417	-2.66563745	1.02206365
H	-0.91491053	-1.49249132	2.33155989
H	-0.64002180	-3.22618798	2.65072517
C	-4.49879542	-2.04662230	-0.56054018
C	-5.23818786	-3.92978138	-3.33152917
C	-4.50562365	-4.88673570	-2.36919274
C	-6.76453701	-4.04061011	-3.12517473
C	-4.91537354	-4.33576620	-4.79040117
H	-3.41642503	-4.80964457	-2.48791756
H	-4.79987208	-5.92310576	-2.59271751
H	-7.30673761	-3.42404357	-3.85461607
H	-7.07304955	-5.08685882	-3.27195020
H	-7.05012450	-3.72338981	-2.11700369
H	-5.31590264	-5.33913812	-4.99746941
H	-5.36140352	-3.62520351	-5.49900057
H	-3.82792962	-4.35653806	-4.95977910
C	-5.65728885	-1.38011646	-3.68617780
O	-5.63837220	-1.11780484	-4.90623318
N	-6.49106401	-0.79647122	-2.80617479
H	-6.53414156	-1.19207549	-1.85354901
H	-2.58029658	-4.90692395	2.29392995
H	-4.24685834	-4.30469158	2.07201110
H	-4.05086571	-2.83074391	4.17131083
H	-2.45365387	-3.58771613	4.39211289
C	-8.71086579	-0.59329625	-3.90008475
H	-8.34441879	-1.13671749	-4.78002515
H	-9.47871441	0.12478732	-4.22376749
H	-9.17259370	-1.31163463	-3.20683202
H	-7.17449433	1.41597343	-1.48121151
H	-8.46502063	0.21921611	-1.23253064
H	-8.77112032	1.65826061	-2.23667443
C	0.47977822	0.60015823	-0.60310834
C	0.82099517	-0.73973076	-0.82963709
H	-1.48941586	-3.98895138	-1.15163060
H	-1.01901694	-3.68349260	-2.84791076
H	-0.42262812	0.94622003	-1.10398532
C	-0.14203832	-1.66246978	-1.27982734

H	1.20904844	2.61611338	-0.81596281
C	1.76906331	1.86797807	1.13866332
O	-1.34650183	-1.41385516	-1.56726680
O	0.33806935	-2.96743160	-1.39985653
C	-0.64566694	-3.93865153	-1.84848129
H	-0.11864670	-4.89681827	-1.87301415
C	1.54696309	1.66388031	-0.37844455
H	2.48205961	1.37485504	-0.87896929
H	1.80524280	-1.11459671	-0.55128021
H	2.33777729	1.01849300	1.54844653
H	2.32360342	2.79654216	1.34551691
C	0.35823340	1.87803268	1.73738268
O	-0.32194814	0.77074909	1.16939144
H	0.38765132	1.78288257	2.83885875
H	-0.13256355	2.83988685	1.49523033

TS–Mode A–LA2–RA2–*S*

COSMO(THF)-ZORA-M06-2X/TZ2P//COSMO(THF)-ZORA-BLYP-D3(BJ)/DZP

E = -20669.57

G = -20152.46

COSMO(THF)-ZORA-BLYP-D3(BJ)/DZP

E = -14452.37

G = -13935.26

$\boldsymbol{N}_{imag}$ = 1, 290*i* cm^{-1}

C	-3.61721858	-2.82506002	-1.52088685
N	-2.75070519	-2.78267672	-0.32231234
H	-2.46898360	-1.81946679	-0.09456379
C	-6.37462477	-5.22012349	-0.27140526
S	-2.14464679	-5.40760112	0.09391110
N	-0.94134009	-3.13087752	0.98777932
H	-0.87927985	-2.10332431	0.88620855
C	0.02024688	-3.68470107	1.95338220
H	0.75392230	-2.86967470	2.05107360
C	-6.36398797	-5.84145890	1.13118202
C	0.80667306	-4.87661554	1.37601655
N	1.39794341	-4.43248891	0.09851906
H	0.13413543	-5.71797821	1.19090582
P	1.99679932	-5.43897252	-1.02949172
C	0.78856892	-6.69201303	-1.50767770
C	0.51731562	-7.76337533	-0.63886243
C	0.05326926	-6.53487315	-2.69244350
C	-0.50908628	-8.65544523	-0.94658841

H	1.09634349	-7.89523998	0.27337870
C	-0.95876576	-7.44374145	-3.00006228
H	0.26290113	-5.70360853	-3.36048880
C	-1.24762989	-8.49421266	-2.12451066
H	-0.73375006	-9.47626518	-0.26683407
H	-1.53349398	-7.32193029	-3.91581294
H	-2.05053781	-9.19193336	-2.36028509
C	3.48890841	-6.32555957	-0.48720051
C	3.84424570	-7.58134000	-1.00612849
C	4.31568996	-5.68963971	0.45481442
C	5.02843553	-8.19262197	-0.58678006
H	3.19970680	-8.08051596	-1.72704654
C	5.49561155	-6.30827612	0.87113578
H	4.03433307	-4.71834897	0.85677661
C	5.85223841	-7.55774654	0.34964929
H	5.30554546	-9.16676190	-0.98745926
H	6.13524825	-5.81797166	1.60375802
H	6.77257496	-8.03961395	0.67747581
C	2.43044134	-4.40025727	-2.44468118
C	3.38966446	-4.85696544	-3.36540268
C	1.81615574	-3.14893015	-2.61776375
C	3.72533451	-4.06191209	-4.46197827
H	3.88479857	-5.81500122	-3.21971965
C	2.16881943	-2.35903555	-3.71147298
H	1.10325326	-2.77593025	-1.89356757
C	3.11576548	-2.81245582	-4.63425505
H	1.69981045	-1.38457739	-3.83635927
H	3.38611939	-2.19127238	-5.48777654
H	1.58923807	-5.17712206	2.08713172
H	4.47012770	-4.41237206	-5.17537238
H	1.19477821	-4.96514433	4.21210691
H	1.78722579	-3.43130679	0.11271634
H	-7.19529509	-4.49195419	-0.34867171
H	-4.38360973	-4.91235744	-3.36340650
H	-3.83490792	-1.76321377	-1.67274778
H	-2.24065157	-2.51278203	3.13503678
C	-0.55466185	-3.89345703	3.39816456
C	-1.46960386	-5.12949223	3.51609421
C	0.63216458	-4.03696195	4.38109378
C	-1.35819057	-2.62812786	3.77787082
H	-2.33038366	-5.05144768	2.84306092
H	-0.93197500	-6.05426518	3.26462581

H	-1.83296257	-5.21733049	4.55179353
C	-1.92631721	-3.71536878	0.25931632
C	-2.96611899	-3.30522627	-2.89397109
C	-3.30084645	-4.77277051	-3.23959672
C	-3.54042344	-2.39608538	-4.00783641
C	-1.44006911	-3.11475455	-2.83719257
H	-2.94498886	-5.45213893	-2.46009673
H	-2.81641279	-5.03798028	-4.19137354
H	-3.24298942	-1.34913560	-3.84587303
H	-3.15279061	-2.71571527	-4.98697734
H	-4.63687093	-2.44820552	-4.02723755
H	-1.00718578	-3.32896727	-3.82504657
H	-1.17659074	-2.08380714	-2.56633256
H	-0.98253008	-3.79547697	-2.11314474
C	-5.02708642	-3.42241751	-1.30989546
O	-5.99509327	-2.92683859	-1.92831614
N	-5.12673092	-4.46543892	-0.46558036
H	-4.24004084	-4.90709034	-0.15448438
H	-1.69574135	-2.69833560	4.82203885
H	-0.74119418	-1.72227457	3.67534465
H	1.32746368	-3.18855857	4.28952587
H	0.25699189	-4.05877870	5.41484434
C	-6.54302946	-6.27698632	-1.37785680
H	-5.52280344	-6.54326419	1.23528638
H	-7.29693925	-6.39360841	1.31012026
H	-6.26117263	-5.06252968	1.89924618
H	-5.70815782	-6.99263324	-1.34874369
H	-6.55645438	-5.79578264	-2.36442241
H	-7.48463122	-6.82947144	-1.24333458
C	1.74697998	-0.26583470	0.18899992
C	0.80972733	-0.01023905	-0.84129038
H	-3.25709843	0.05478667	-0.97372028
H	-2.65344725	1.72326589	-0.81193127
H	1.31056179	-0.33754736	1.18681976
C	-0.57351296	-0.03772527	-0.62038947
H	3.57609869	0.43315141	1.11025740
C	3.99621815	-0.49922403	-0.80116779
O	-1.19987778	-0.38149407	0.42801304
O	-1.31003340	0.39781238	-1.74138305
C	-2.66328435	0.83489816	-1.45956141
H	-3.10108130	1.08190220	-2.43110198
C	3.12143574	0.38636460	0.10836693

H	3.03879094	1.41214881	-0.27788606
H	1.15826832	0.31473210	-1.82039072
H	3.63556324	-0.44386813	-1.83995923
H	5.05559485	-0.20385468	-0.78136411
C	3.76877110	-1.91489358	-0.24805527
O	2.40469344	-1.99002712	0.15600675
H	3.98491001	-2.67453382	-1.01925010
H	4.44798394	-2.09719871	0.61024650

TS–Mode B–LA1–RA1–*S* (TS-(*S*))

COSMO(THF)-ZORA-M06-2X/TZ2P//COSMO(THF)-ZORA-BLYP-D3(BJ)/DZP

E = -20682.63

G = -20165.09

COSMO(THF)-ZORA-BLYP-D3(BJ)/DZP

E = -14463.95

G = -13946.41

N_{imag} = 1, 296*i* cm^{-1}

C	-3.68517944	-2.71994208	-1.33345345
N	-2.67455397	-2.55203635	-0.28507877
H	-2.16336393	-1.64524927	-0.28188136
C	-1.22495714	-4.28345939	-3.80442574
S	-2.57068745	-5.19158706	0.36667335
N	-0.80111611	-3.18115673	0.82899489
H	-0.53059276	-2.20208605	0.59800457
C	0.04745024	-3.92742730	1.74692489
H	-0.53286091	-4.80271846	2.07057696
C	-0.92924601	-5.78594875	-3.87186192
C	0.33757807	-3.02369013	2.96676871
N	-0.86254575	-2.47915379	3.62111043
H	0.92951786	-2.15358795	2.65222646
P	-1.79002682	-3.26805190	4.72496805
C	-0.70204997	-4.35578960	5.67448593
C	-0.25898671	-5.56225416	5.10234512
C	-0.24227516	-3.96151046	6.94055337
C	0.64911716	-6.36278785	5.79395880
H	-0.62341407	-5.87448251	4.12628813
C	0.66218205	-4.77414069	7.62921567
H	-0.59193119	-3.03251901	7.38598318
C	1.10941491	-5.96914043	7.05625318
H	0.99649925	-7.29411941	5.34944014
H	1.01645757	-4.47265120	8.61390402
H	1.81690301	-6.59787051	7.59540874

C	-3.17730030	-4.26429041	4.11578493
C	-3.52602801	-5.48223076	4.72622529
C	-3.97315288	-3.72820418	3.08957326
C	-4.67310252	-6.15904026	4.30620084
H	-2.91949459	-5.89335188	5.52979966
C	-5.12056030	-4.40955893	2.68754779
H	-3.69597243	-2.78643629	2.62368794
C	-5.47325004	-5.61906075	3.29258450
H	-4.94469400	-7.10355464	4.77615169
H	-5.73166095	-4.00123867	1.88758520
H	-6.36940419	-6.14809700	2.96908649
C	-2.51919150	-1.98777598	5.77791148
C	-3.74637120	-2.22339864	6.41779338
C	-1.86019316	-0.75842773	5.93535498
C	-4.30762669	-1.22827231	7.22063652
H	-4.27156065	-3.16481898	6.27219959
C	-2.43246091	0.23239247	6.73376487
H	-0.92339819	-0.57704979	5.41424036
C	-3.65330669	-0.00161018	7.37700125
H	-1.93027420	1.19253156	6.84535365
H	-4.10080369	0.77768020	7.99307395
H	0.93052275	-3.56366316	3.71054643
H	-5.26354065	-1.40619000	7.71157602
H	2.67258480	-4.48349979	2.83204804
H	-1.42023954	-1.75416340	3.09080411
H	-1.62019933	-3.94244426	-4.77336819
H	-3.62395817	-0.07330815	-2.35399176
H	-4.18615440	-3.67754550	-1.13385413
H	0.27942305	-4.99860581	-0.76709008
C	1.35904340	-4.44583806	1.05367608
C	2.14320977	-3.27722970	0.41357226
C	2.24756780	-5.17231766	2.08964996
C	0.96882771	-5.45270771	-0.04904176
H	1.52829008	-2.74664269	-0.32521888
H	2.48500740	-2.54961019	1.16293438
H	3.03279932	-3.66648523	-0.10315511
C	-1.98841053	-3.57362586	0.30322669
C	-4.75840915	-1.59490005	-1.26457054
C	-4.14466924	-0.18151124	-1.39681201
C	-5.79384497	-1.83659868	-2.38542789
C	-5.46212259	-1.70511228	0.10628916
H	-3.44002179	0.03100381	-0.58342464

H	-4.94858102	0.56830683	-1.33697372
H	-6.22669054	-2.84628705	-2.30657560
H	-6.61386284	-1.10842534	-2.29886762
H	-5.33486989	-1.72913077	-3.37593178
H	-6.22537619	-0.91960226	0.20408916
H	-5.95564360	-2.68175304	0.21189073
H	-4.74035013	-1.58603933	0.92068914
C	-2.92275614	-2.85607152	-2.68543723
O	-2.83513413	-1.95865311	-3.54120001
N	-2.29029994	-4.05567995	-2.80769033
H	-2.31992194	-4.68605353	-1.99383370
H	1.86858979	-5.78669370	-0.58721364
H	0.46492115	-6.32944681	0.37975422
H	1.67729118	-5.94685897	2.62358603
H	3.08612993	-5.66630616	1.57674614
C	0.02833482	-3.45602707	-3.46402601
H	-0.55688837	-6.14980967	-2.90250511
H	-0.15951111	-5.98601065	-4.62947894
H	-1.83455278	-6.35205797	-4.13269188
H	0.44231057	-3.77282706	-2.49830740
H	-0.22318512	-2.39061538	-3.40261261
H	0.79853960	-3.59313179	-4.23731887
C	-0.79436173	0.69115180	1.15638087
C	-2.12522157	1.09809704	1.46154126
H	-5.32304161	-0.83434568	3.02852712
H	-4.52232283	-0.01791823	4.39579103
H	-0.30416489	0.09672969	1.93118763
C	-2.95824567	0.31999938	2.27121121
H	1.16713039	1.43456726	0.61047141
C	-0.19498393	1.49401879	-1.08258477
O	-2.71318135	-0.80791177	2.78320397
O	-4.21870231	0.89308558	2.51702564
C	-5.04500553	0.14009895	3.44581249
H	-5.94051894	0.75202884	3.59673455
C	0.10981833	1.67554632	0.42040717
H	-0.07447608	2.70628351	0.75358908
H	-2.55198326	1.96792163	0.96410369
H	-1.19363403	1.89779766	-1.30958838
H	0.53974573	1.99966713	-1.72638792
C	-0.19112524	-0.03137749	-1.26920916
O	-0.74126826	-0.59164496	-0.07274106
H	-0.79930049	-0.33934483	-2.13518217

H	0.84332054	-0.39367199	-1.42422826

TS–Mode B–LA1–RA2–*S*

COSMO(THF)-ZORA-M06-2X/TZ2P//COSMO(THF)-ZORA-BLYP-D3(BJ)/DZP

E = -20671.42

G = -20154.74

COSMO(THF)-ZORA-BLYP-D3(BJ)/DZP

E = -14459.08

G = -13942.40

N_{imag} = 1, 36*i* cm^{-1}

C	-2.79066081	-1.49811727	4.28718121
N	-2.27545310	-0.33111411	3.55942902
H	-2.10267152	-0.46801227	2.53754972
C	0.58575315	-3.08795454	3.81802866
S	-1.17389780	0.69977673	5.81474321
N	-0.76285165	1.30271852	3.16738691
H	-0.96966183	0.98710189	2.18508361
C	0.10998135	2.47266948	3.26679695
H	0.56395474	2.51408684	2.27032212
H	2.65186799	-3.49840785	4.34474342
C	1.26776819	2.31425156	4.26712893
N	2.04100658	1.07001186	3.95578055
H	0.89203132	2.23416880	5.29009593
P	3.52991220	0.89457021	4.67862343
C	3.29233062	0.83857487	6.46199960
C	2.11665231	0.25917760	6.96730916
C	4.31443797	1.25813527	7.33164156
C	1.96380335	0.10945563	8.34580259
H	1.31512429	-0.03441459	6.29440825
C	4.15375232	1.09354859	8.70840523
H	5.22640970	1.70270088	6.93865781
C	2.98149493	0.51898256	9.21462061
H	1.04709112	-0.32697395	8.74030846
H	4.94377662	1.41409938	9.38579998
H	2.86058389	0.39335311	10.28988919
C	4.66940253	2.25166979	4.29662169
C	4.43972626	3.52008071	4.86547984
C	5.67247461	2.08661996	3.32695496
C	5.19894000	4.61367549	4.44831188
H	3.68243479	3.65066898	5.63566335
C	6.42956033	3.18673968	2.92036764
H	5.85839786	1.10859282	2.89019575

C	6.18848197	4.44891177	3.47232979
H	5.01887050	5.59413173	4.88688856
H	7.20684312	3.05746875	2.16848214
H	6.77714219	5.30582582	3.14686793
C	4.26150317	-0.66641172	4.12993098
C	4.20318670	-1.06289033	2.78254842
C	4.92251317	-1.46659230	5.07830570
C	4.80313105	-2.26245992	2.39680067
H	3.68920748	-0.45392828	2.04402612
C	5.52104712	-2.66145442	4.67643724
H	4.95468629	-1.17315762	6.12464964
C	5.46151913	-3.06046851	3.33734469
H	6.02361504	-3.28572503	5.41378918
H	5.92309514	-3.99772833	3.02839746
H	1.90210941	3.20494973	4.17659220
H	4.74976184	-2.57538773	1.35462441
H	1.14563247	5.09900113	3.68755701
H	2.13072136	0.94656934	2.91996341
H	0.33990221	-4.10700062	3.48797995
H	-4.67904959	0.45426295	4.25057725
H	-2.63146576	-1.28587356	5.35318345
H	-2.57029172	3.06132613	2.67090819
C	-0.65140673	3.84597843	3.39045778
C	-1.19094504	4.11544486	4.80906135
C	0.31581941	4.98220382	2.97711180
C	-1.82886493	3.82000565	2.38906891
H	-1.87644212	3.32263985	5.12882493
H	-0.37850897	4.17412004	5.54701517
H	-1.72726810	5.07682849	4.82047266
C	-1.40375790	0.57676530	4.10747416
C	-4.31712617	-1.69790652	4.07906176
C	-5.02939704	-0.47950505	4.71142506
C	-4.69682471	-1.78575876	2.58561544
C	-4.75034498	-2.98222188	4.81944892
H	-4.82825672	-0.42358478	5.79163799
H	-6.11669932	-0.55851953	4.56372774
H	-4.15105105	-2.59765228	2.09166715
H	-5.77812488	-1.96715986	2.48762289
H	-4.46595591	-0.84479683	2.07027673
H	-5.84531027	-3.08282574	4.78611170
H	-4.30318104	-3.87166939	4.35848554
H	-4.44523881	-2.94477142	5.87694365

C	-1.85611073	-2.66750152	3.89448367
O	-2.13782149	-3.55197366	3.07078827
N	-0.62605217	-2.56113861	4.48885923
H	-0.48242429	-1.70349511	5.03718048
H	-2.32362410	4.80188979	2.37042325
H	-1.47487923	3.58893702	1.37302260
H	0.73630543	4.79542193	1.97873167
H	-0.22761055	5.93800650	2.94703754
C	0.91046693	-2.21559451	2.59346117
H	1.49350705	-3.79002444	5.67494577
C	1.73641329	-3.13419266	4.82703508
H	1.94732394	-2.12904140	5.21459734
H	1.77002799	-2.62246998	2.04377090
H	1.15314456	-1.19562443	2.91482491
H	0.04673328	-2.15184045	1.92000399
C	0.25926790	-0.00123779	-0.35332183
C	0.76227956	1.29534266	-0.39046607
H	3.15617326	3.39153967	2.10236820
H	4.21669462	2.93236257	0.74704299
H	0.81714896	-0.72055216	0.24164646
C	1.84963521	1.69922275	0.41938904
H	-0.66771559	-1.63477587	-1.40026522
C	-2.00506224	0.07223655	-1.37174362
O	2.51695309	0.99312101	1.21718796
O	2.16590408	3.05243201	0.26726254
C	3.32319162	3.50114579	1.02645262
H	3.44201519	4.55818987	0.77209156
C	-0.59506303	-0.53887129	-1.47860230
H	-0.13273306	-0.29675663	-2.44842631
H	0.30824979	2.04677574	-1.03533025
H	-1.94820898	1.16164330	-1.52417723
H	-2.69181082	-0.34565041	-2.12480040
C	-2.45634627	-0.24665617	0.06537783
O	-1.34859789	-0.12338421	0.93360426
H	-3.28267650	0.42863340	0.36309491
H	-2.85676770	-1.28235210	0.08407126

TS–Mode B–LA2–RA2–*S*

COSMO(THF)-ZORA-M06-2X/TZ2P//COSMO(THF)-ZORA-BLYP-D3(BJ)/DZP

E = -20669.37

G = -20152.38

COSMO(THF)-ZORA-BLYP-D3(BJ)/DZP

E = -14452.42

G = -13935.43

$\boldsymbol{N}_{\mathbf{imag}}$ = 1, 264*i* cm^{-1}

C	-3.63395531	-2.82618838	-1.52810662
N	-2.76339357	-2.77351137	-0.33335579
H	-2.49014030	-1.80971527	-0.10233595
C	-6.38474280	-5.22292886	-0.26361967
S	-2.14825032	-5.39713165	0.07285596
N	-0.96332666	-3.12176624	0.98952821
H	-0.89843294	-2.09388205	0.89046080
C	0.00179449	-3.67601811	1.94969053
H	0.72702619	-2.85383960	2.05336239
C	-6.37163627	-5.83768059	1.14184069
C	0.80354964	-4.85968737	1.37596618
N	1.42734867	-4.40983615	0.11709214
H	0.14261689	-5.70524507	1.17045894
P	2.01619342	-5.41494826	-1.01868198
C	0.80551574	-6.66456813	-1.49940353
C	0.53452271	-7.73843496	-0.63352315
C	0.06580172	-6.50088221	-2.68051735
C	-0.49383385	-8.62798581	-0.94198421
H	1.11553497	-7.87429783	0.27686463
C	-0.94806633	-7.40727869	-2.98911599
H	0.27516126	-5.66769987	-3.34603608
C	-1.23558239	-8.46103346	-2.11711980
H	-0.71769576	-9.45110225	-0.26476870
H	-1.52510249	-7.28115120	-3.90278084
H	-2.03904519	-9.15766385	-2.35422190
C	3.50759349	-6.31096452	-0.48734107
C	3.86253627	-7.55931855	-1.02396843
C	4.33360573	-5.69014516	0.46536909
C	5.04563089	-8.17789924	-0.61237033
H	3.21841873	-8.04719061	-1.75303539
C	5.51248556	-6.31595009	0.87401960
H	4.05172652	-4.72549840	0.88265920
C	5.86893144	-7.55767381	0.33427564
H	5.32239496	-9.14621925	-1.02719529
H	6.15119115	-5.83754071	1.61530351
H	6.78810821	-8.04547060	0.65654909
C	2.45491318	-4.37162529	-2.42821575
C	3.42008689	-4.82091842	-3.34641734
C	1.82612201	-3.12839994	-2.60607677

C	3.74667193	-4.02673495	-4.44626203
H	3.92653144	-5.77225788	-3.19626446
C	2.16893396	-2.33964321	-3.70388607
H	1.11185405	-2.76052074	-1.88081438
C	3.12079063	-2.78613153	-4.62490561
H	1.68891721	-1.37119974	-3.83334197
H	3.38387439	-2.16535968	-5.48101569
H	1.56769535	-5.15991791	2.10716747
H	4.49578909	-4.37130976	-5.15799246
H	1.19622618	-4.93420167	4.20696104
H	1.85173280	-3.42102579	0.14585171
H	-7.20627604	-4.49599341	-0.34324267
H	-4.41967742	-4.89637602	-3.38010604
H	-3.85972700	-1.76716985	-1.68637085
H	-2.27920853	-2.53968334	3.12651968
C	-0.57319722	-3.89444284	3.39344624
C	-1.46792545	-5.14585034	3.50680644
C	0.61273987	-4.01981424	4.37986634
C	-1.39769159	-2.64268633	3.77273241
H	-2.32695302	-5.08117592	2.83006047
H	-0.91372910	-6.06120216	3.25653001
H	-1.83370395	-5.24122414	4.54095173
C	-1.94093505	-3.70539530	0.25263979
C	-2.98149319	-3.31127562	-2.89905439
C	-3.33592276	-4.77350919	-3.24747575
C	-3.53589628	-2.39124386	-4.01396733
C	-1.45305749	-3.14131196	-2.83420224
H	-2.99672910	-5.45794390	-2.46455636
H	-2.84777463	-5.04675907	-4.19501276
H	-3.22694799	-1.34868176	-3.84620602
H	-3.14525500	-2.71205266	-4.99151646
H	-4.63287223	-2.42994730	-4.04167973
H	-1.01785066	-3.36281631	-3.81947442
H	-1.17872081	-2.11289601	-2.56450572
H	-1.00781373	-3.82560803	-2.10611805
C	-5.04103807	-3.42868656	-1.31214361
O	-6.01131453	-2.93587404	-1.92920089
N	-5.13789678	-4.46772971	-0.46293464
H	-4.25049200	-4.90616108	-0.14974408
H	-1.73817292	-2.72048373	4.81539508
H	-0.79450825	-1.72715107	3.67440633
H	1.28972759	-3.15578906	4.29754948

H	0.23385050	-4.05727306	5.41180838
C	-6.55316985	-6.28495521	-1.36507484
H	-5.52687193	-6.53458282	1.24973543
H	-7.30175898	-6.39396967	1.32254696
H	-6.27367029	-5.05480110	1.90652495
H	-5.71738192	-6.99945540	-1.33379797
H	-6.56843553	-5.80824770	-2.35379850
H	-7.49389026	-6.83802623	-1.22685798
C	1.80153953	-0.27355150	0.16810421
C	0.84573729	-0.03804047	-0.84791738
H	-3.22864126	0.03708611	-0.95664514
H	-2.60453130	1.69774755	-0.80125235
H	1.38670643	-0.35329746	1.17440380
C	-0.53566365	-0.06244107	-0.61180747
H	3.62599626	0.48220841	1.05581435
C	4.05457326	-0.50640152	-0.82899568
O	-1.15334733	-0.38260244	0.44836551
O	-1.28064911	0.35498708	-1.73432957
C	-2.62730109	0.80840883	-1.44703220
H	-3.06668084	1.05827369	-2.41705866
C	3.16967182	0.39173212	0.05780664
H	3.06746210	1.40144870	-0.36397333
H	1.17975807	0.25807189	-1.84129727
H	3.69447617	-0.47678641	-1.86898559
H	5.11086703	-0.19878806	-0.81614150
C	3.84232877	-1.91135210	-0.24599064
O	2.47778626	-2.00010750	0.14399831
H	4.07695987	-2.68335740	-1.00053024
H	4.51761687	-2.06393389	0.62172715

10. NMR Spectra

^{1}H NMR: (400 MHz, $(CD_3)_2SO$, 298K) of **S1**

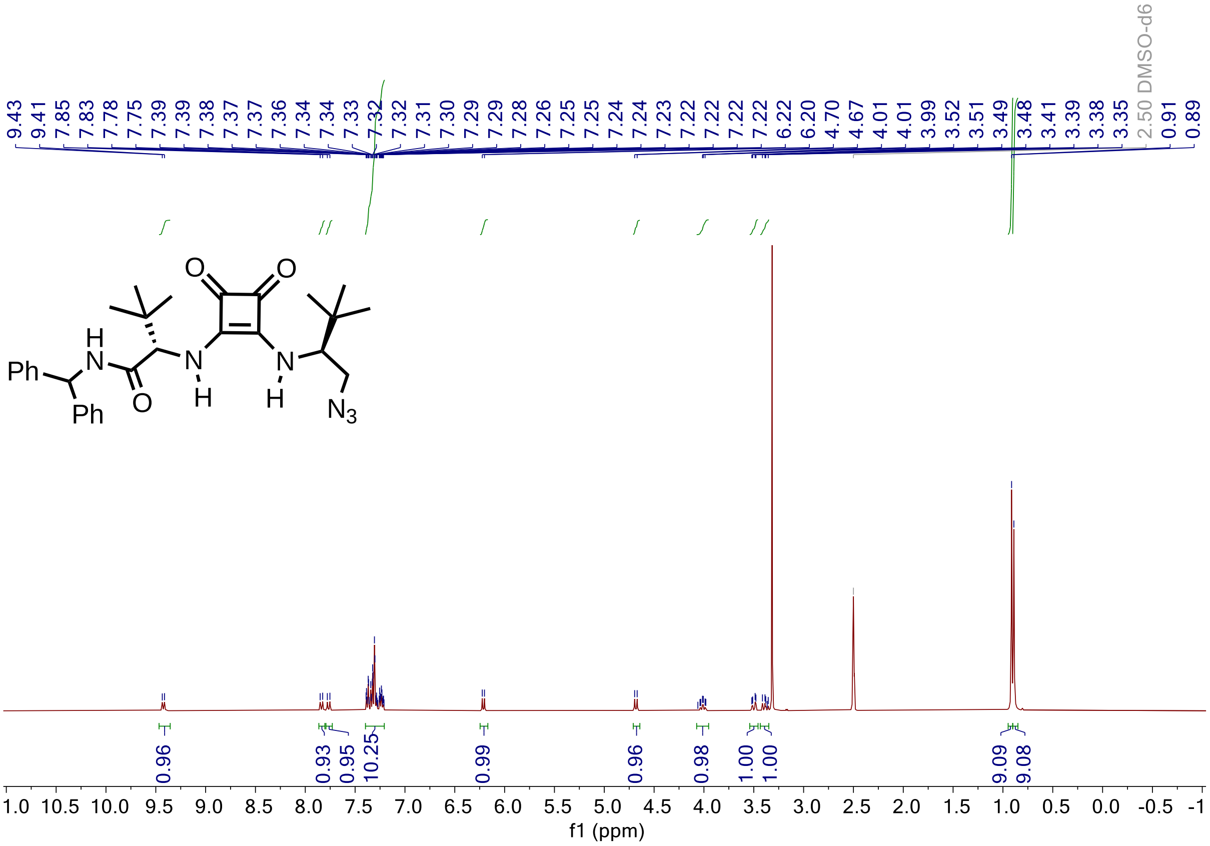

^{13}C NMR: (101 MHz, $(CD_3)_2SO$, 298K) of **S1**

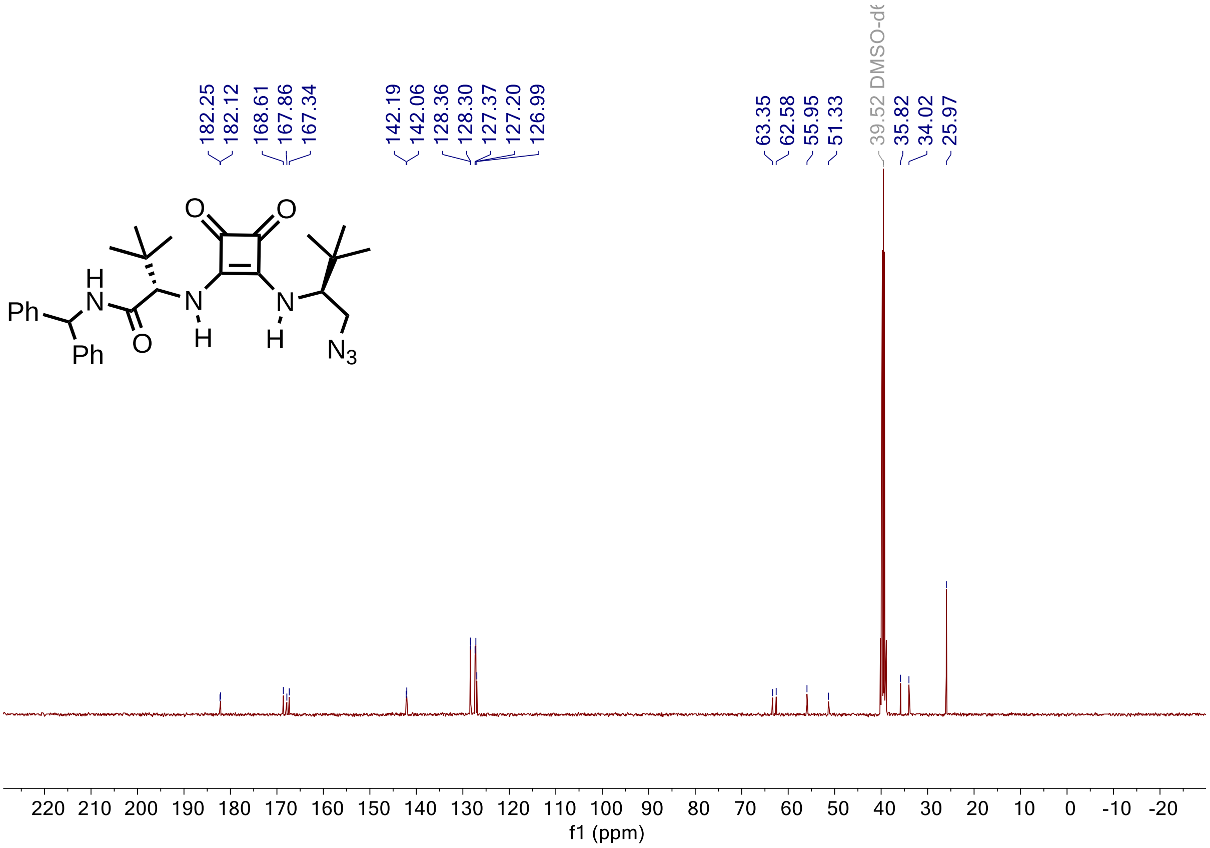

^{1}H NMR: (400 MHz, $CDCl_3$, 298K) of **3b**

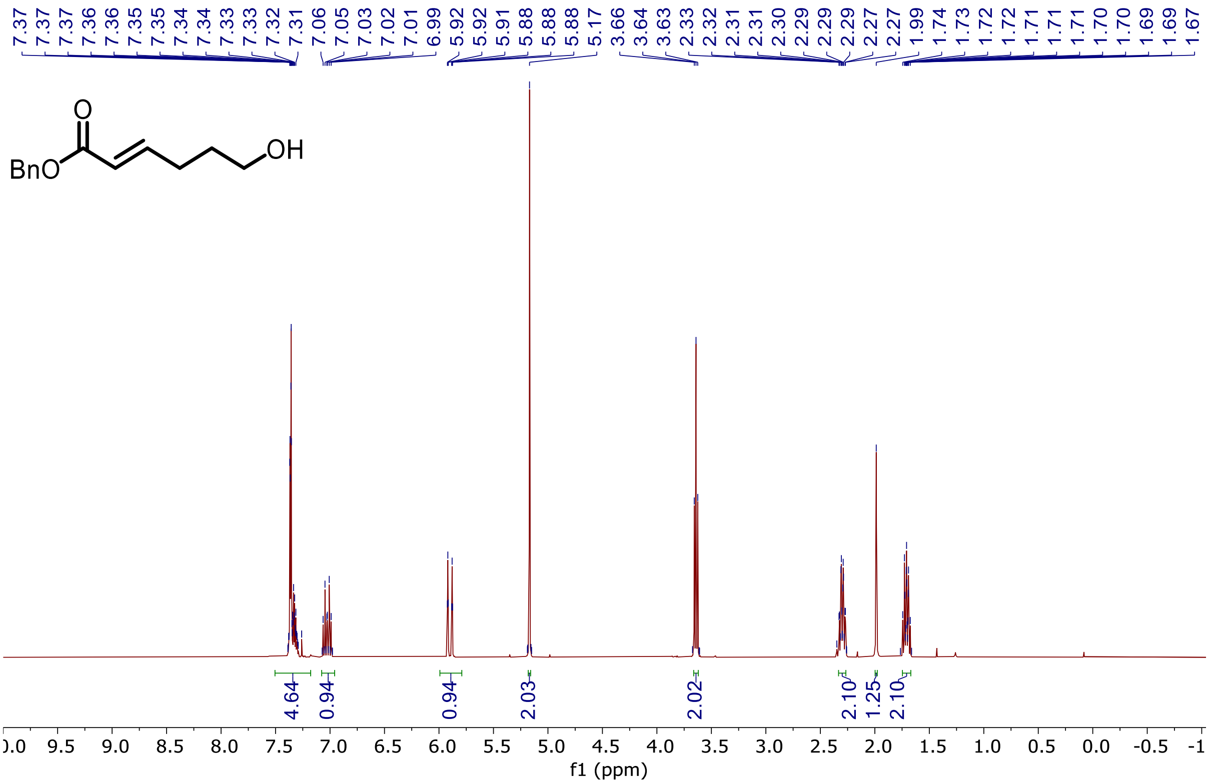

^{13}C NMR: (101 MHz, $CDCl_3$, 298K) of **3b**

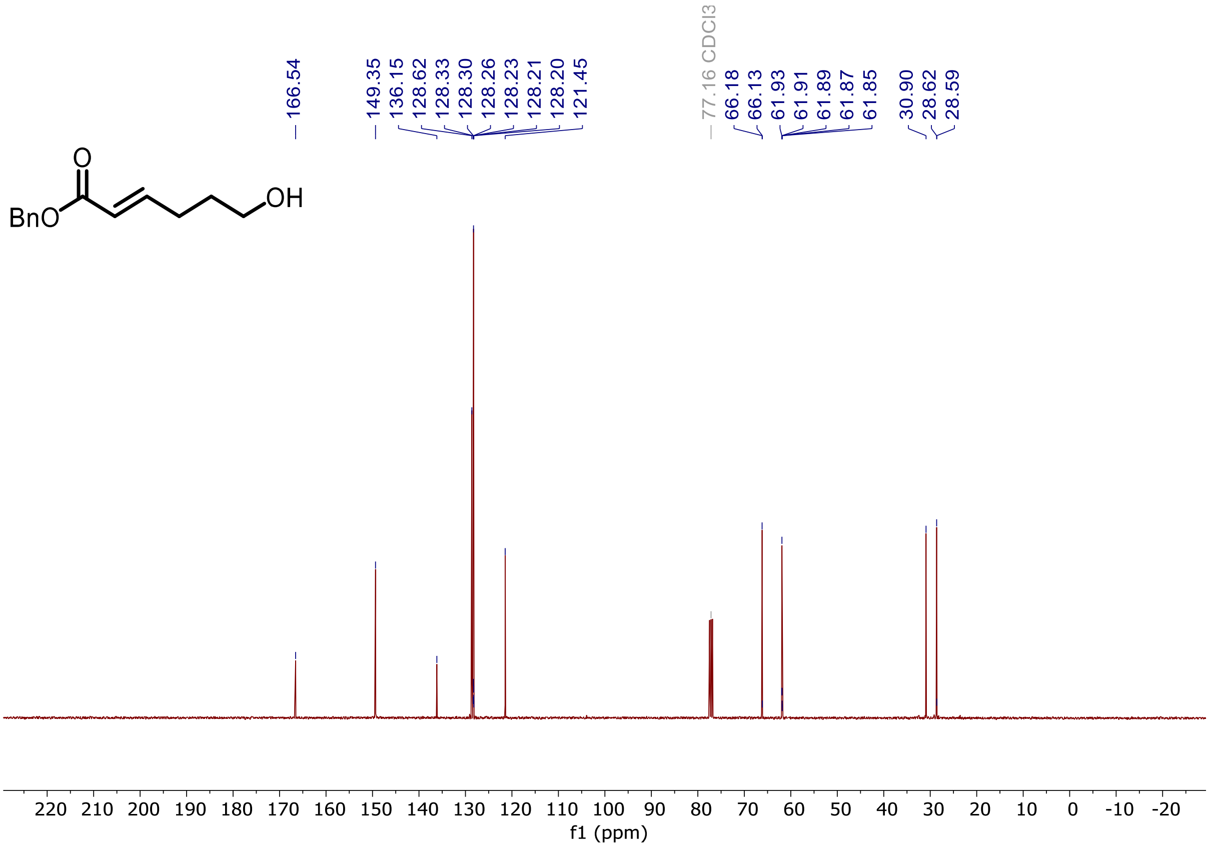

^{1}H NMR: (400 MHz, $CDCl_3$, 298K) of **S11**

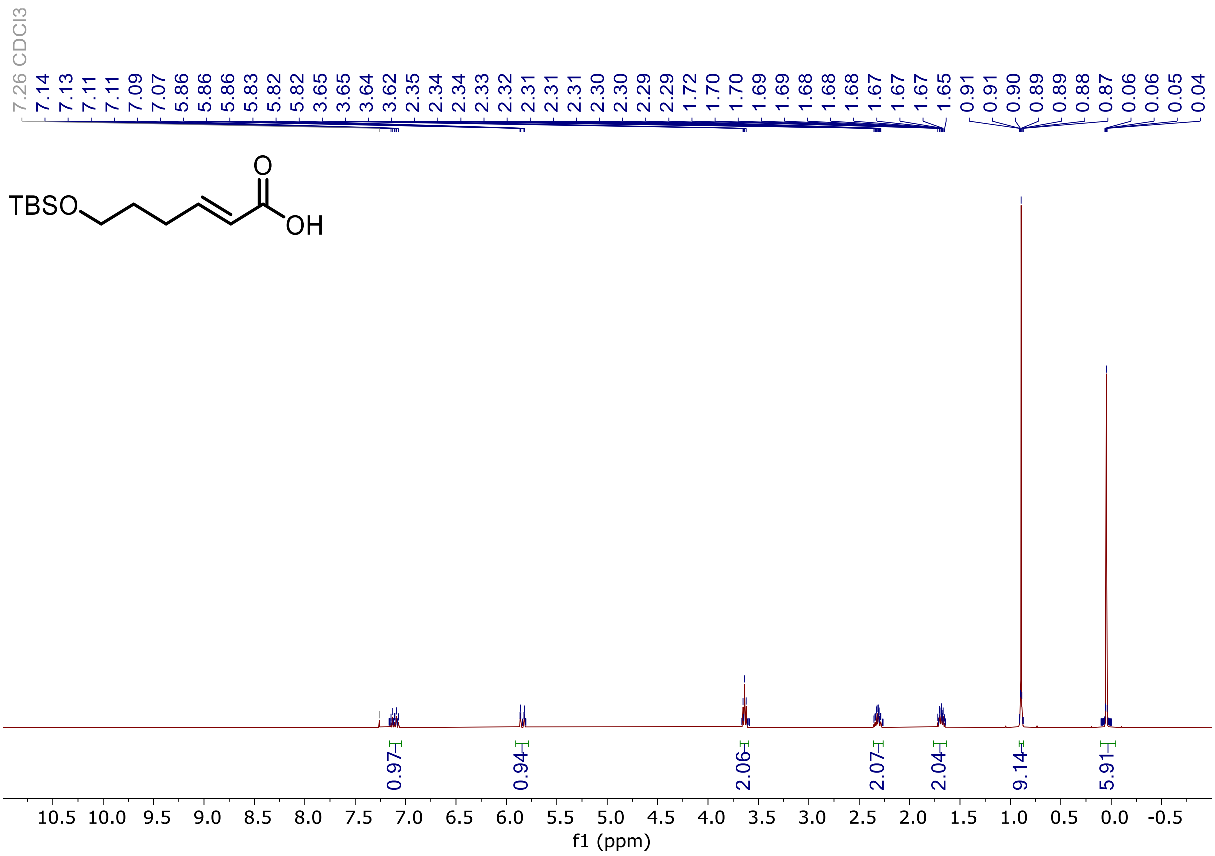

^{13}C NMR: (101 MHz, $CDCl_3$, 298K) of **S11**

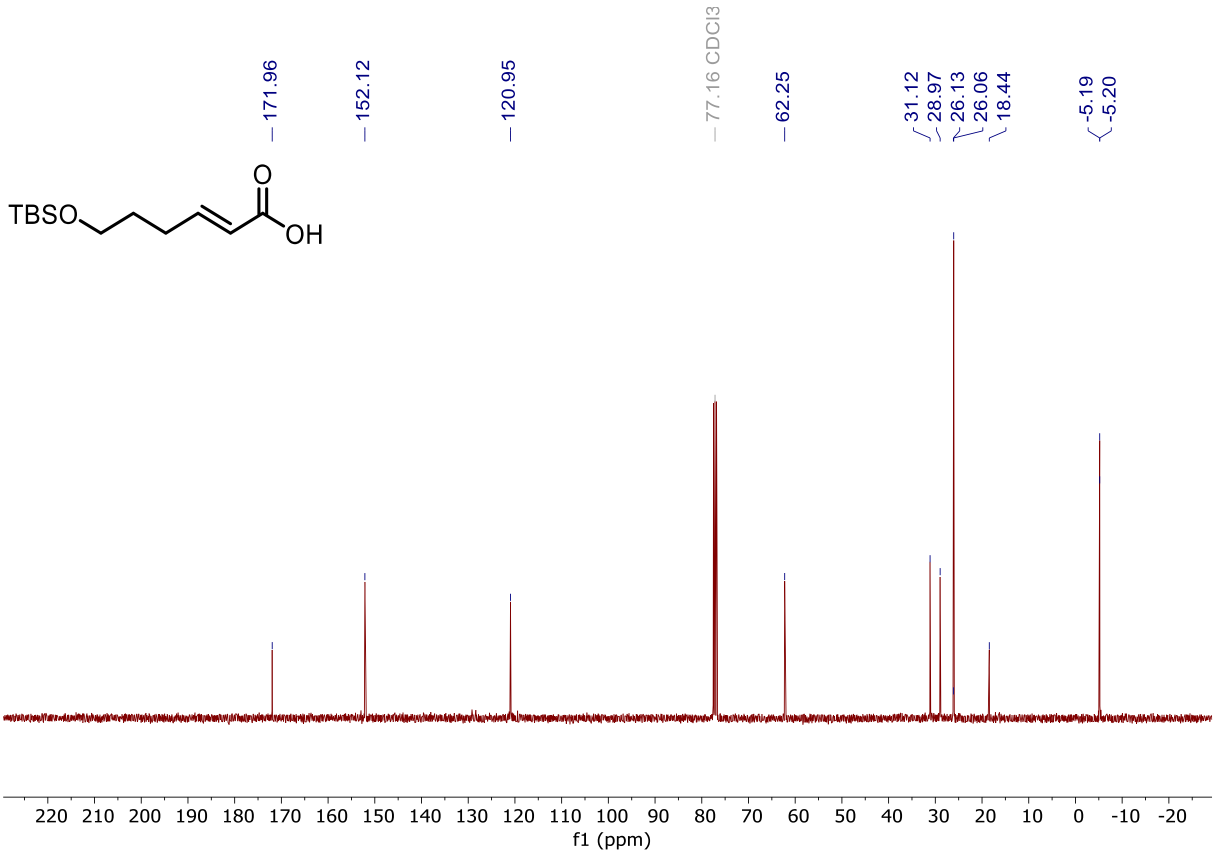

^{1}H NMR: (400 MHz, $CDCl_3$, 298K) of **3c**

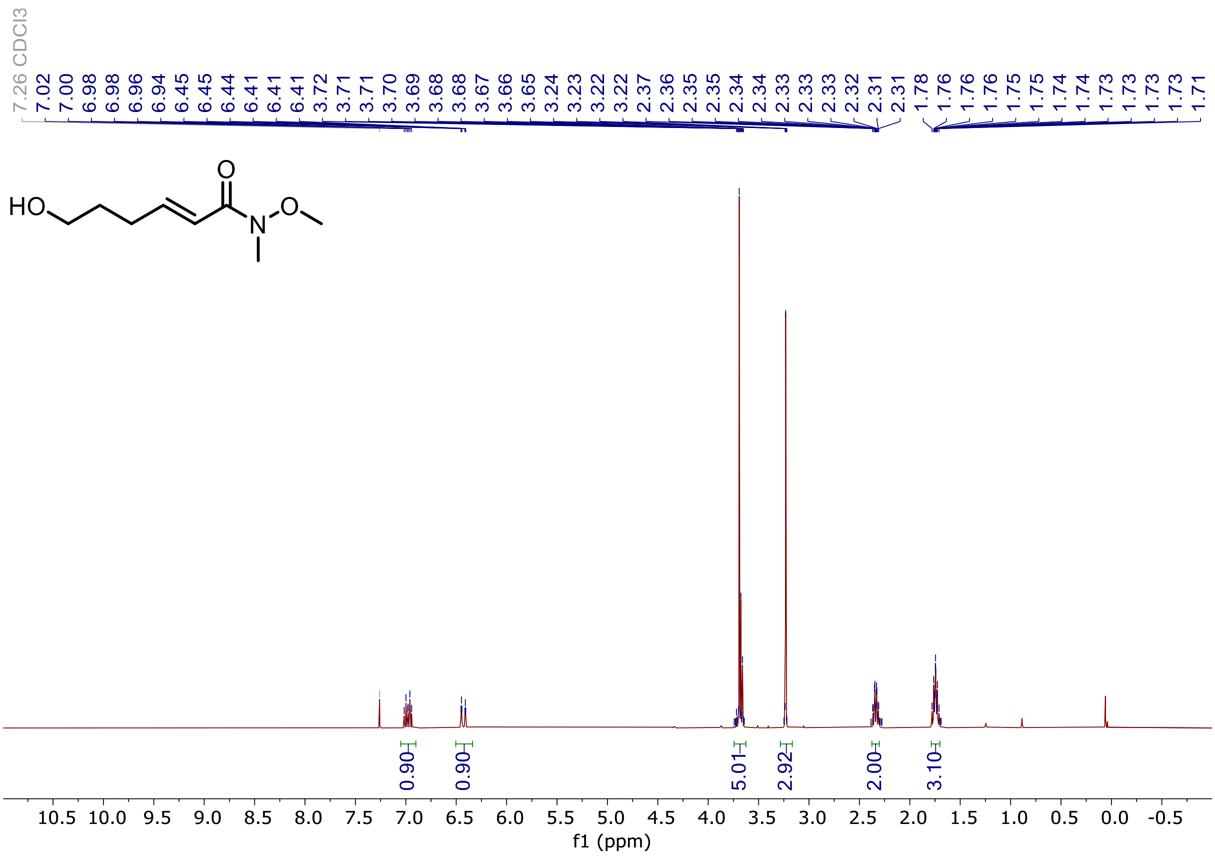

^{13}C NMR: (101 MHz, $CDCl_3$, 298K) of **3c**

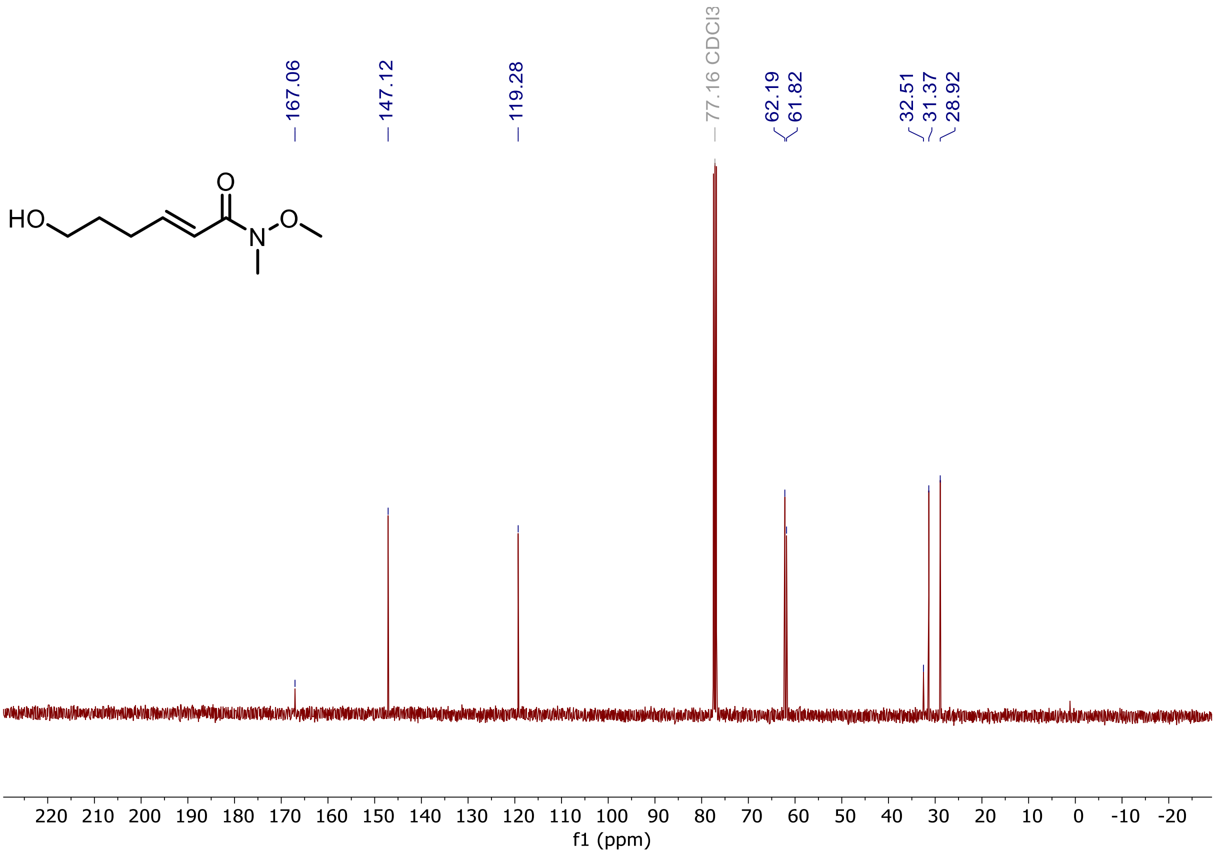

^{1}H NMR: (400 MHz, $CDCl_3$, 298K) of **S13**

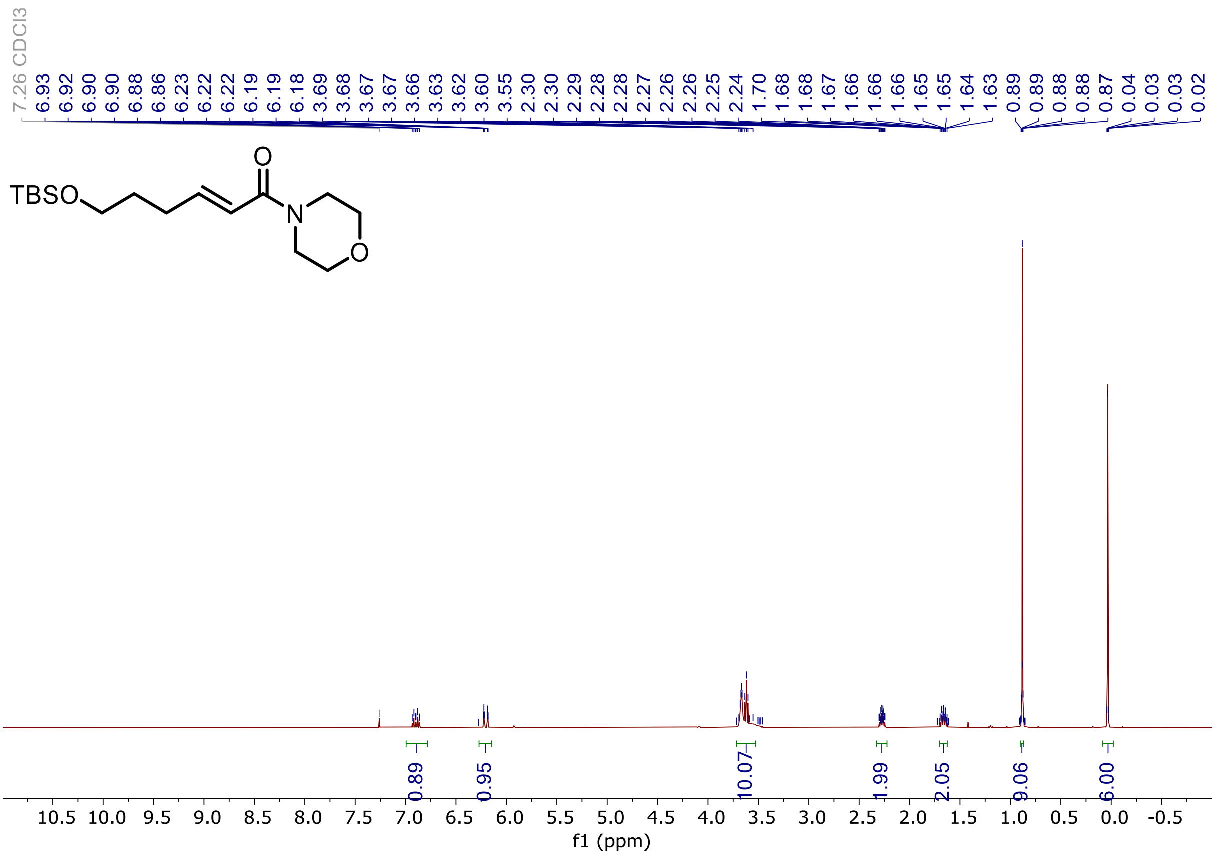

^{13}C NMR: (101 MHz, $CDCl_3$, 298K) of **S13**

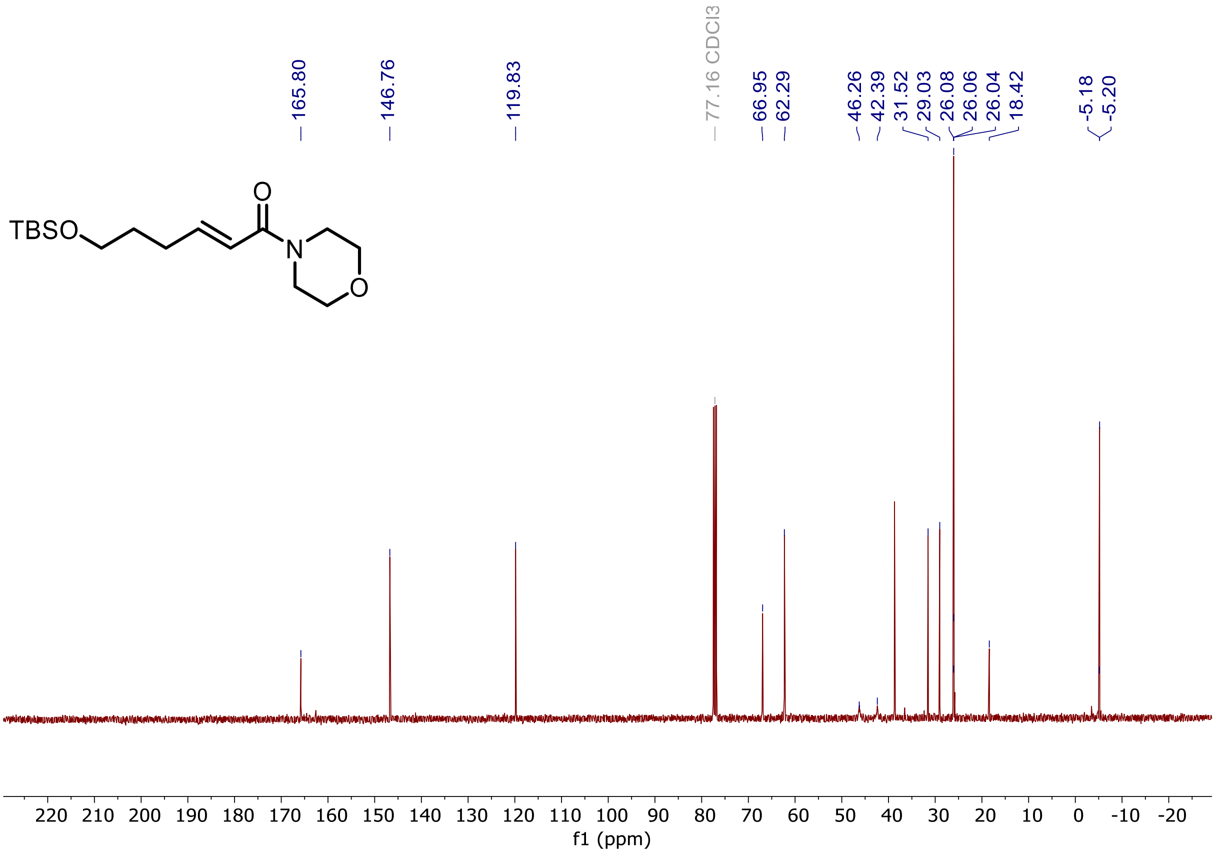

^{1}H NMR: (400 MHz, $CDCl_3$, 298K) of **3d**

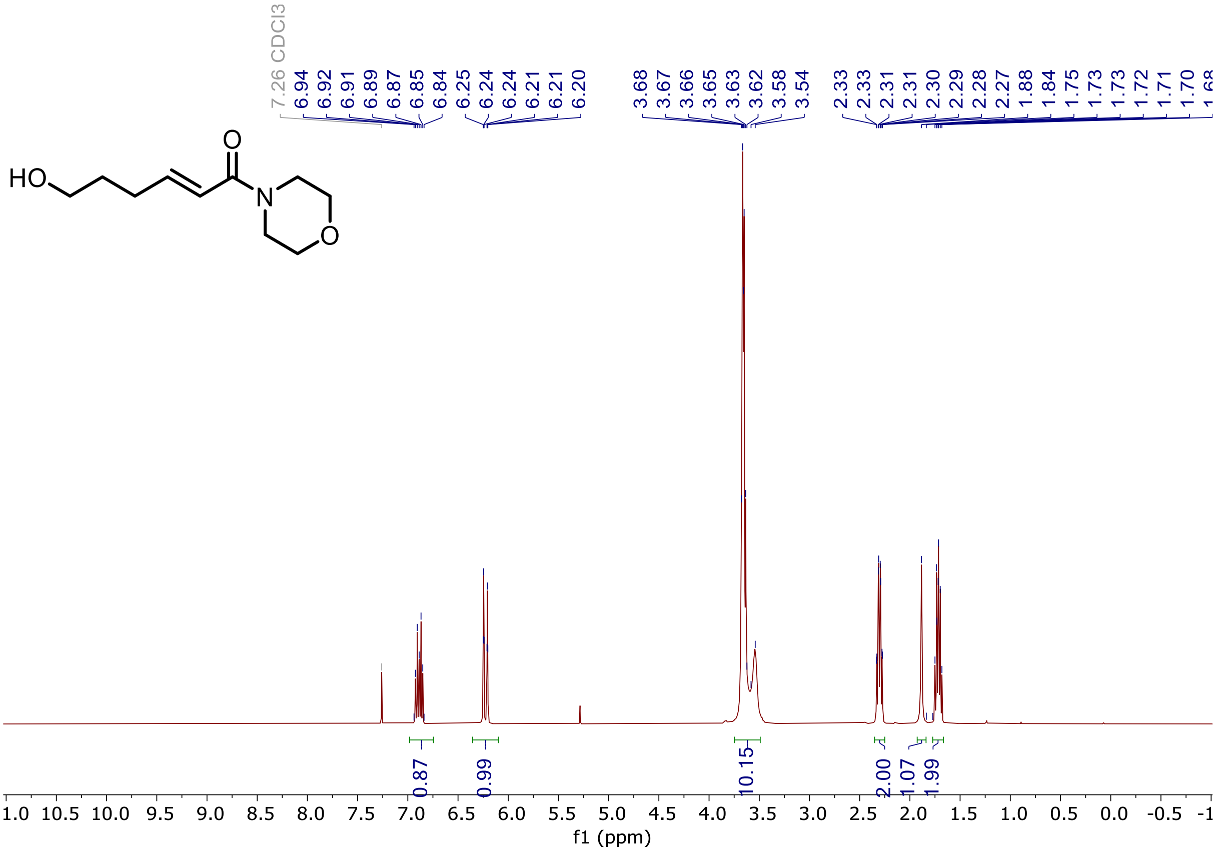

^{13}C NMR: (101 MHz, $CDCl_3$, 298K) of **3d**

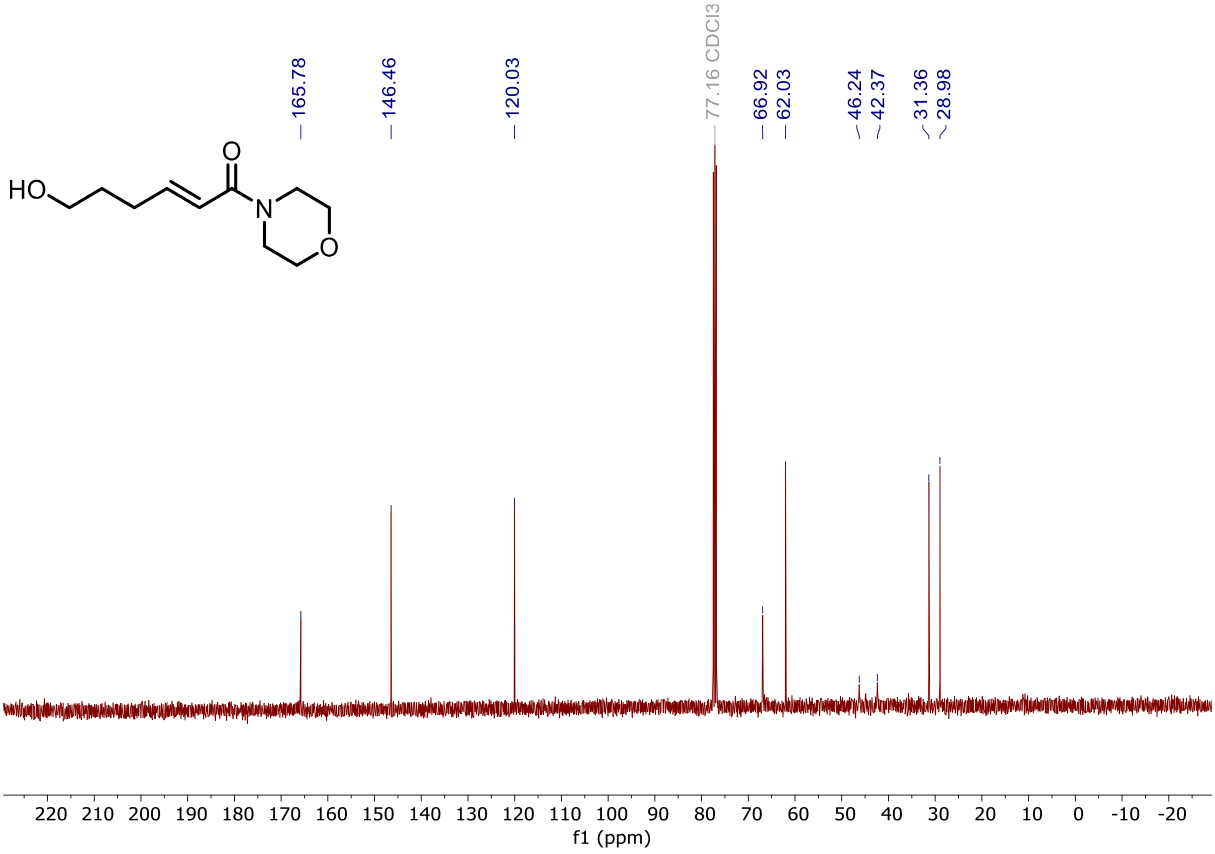

^{1}H NMR: (400 MHz, $CDCl_3$, 298K) of **3e**

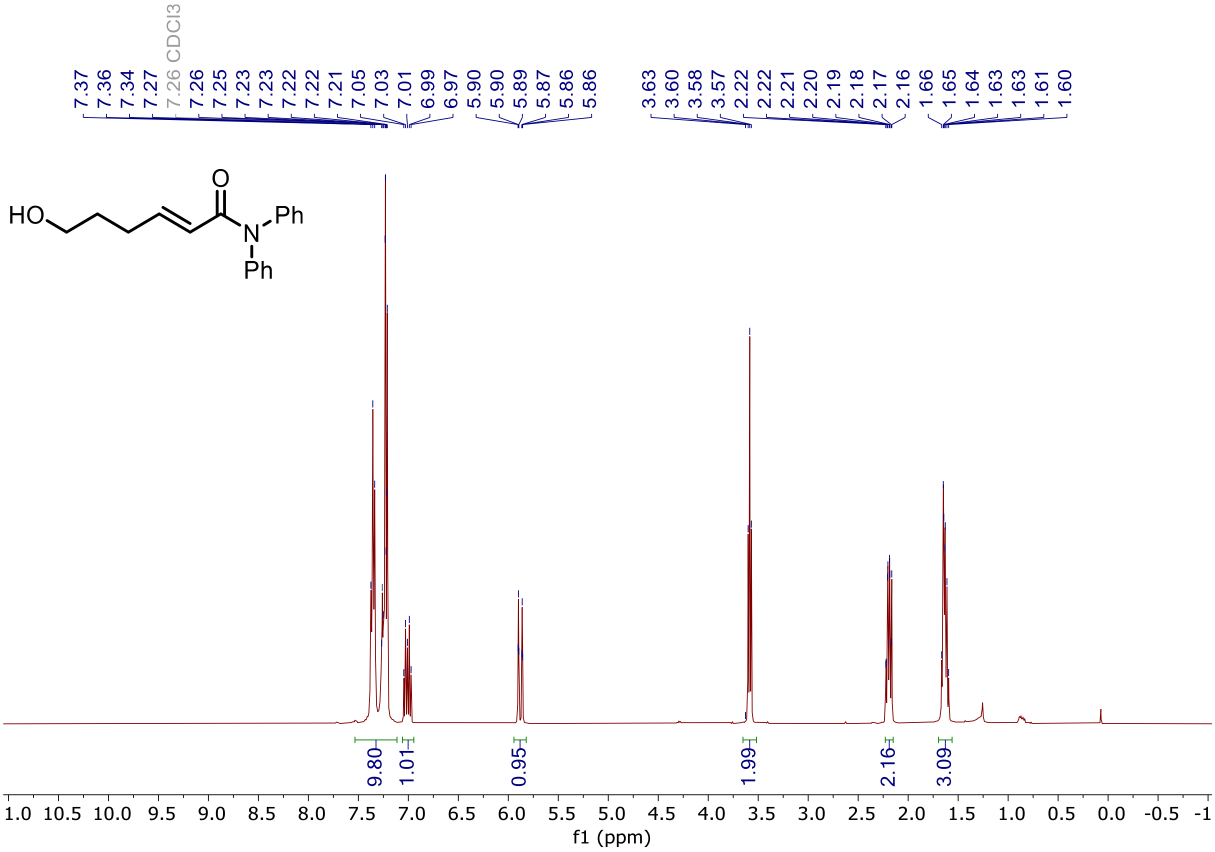

^{13}C NMR: (101 MHz, $CDCl_3$, 298K) of **3e**

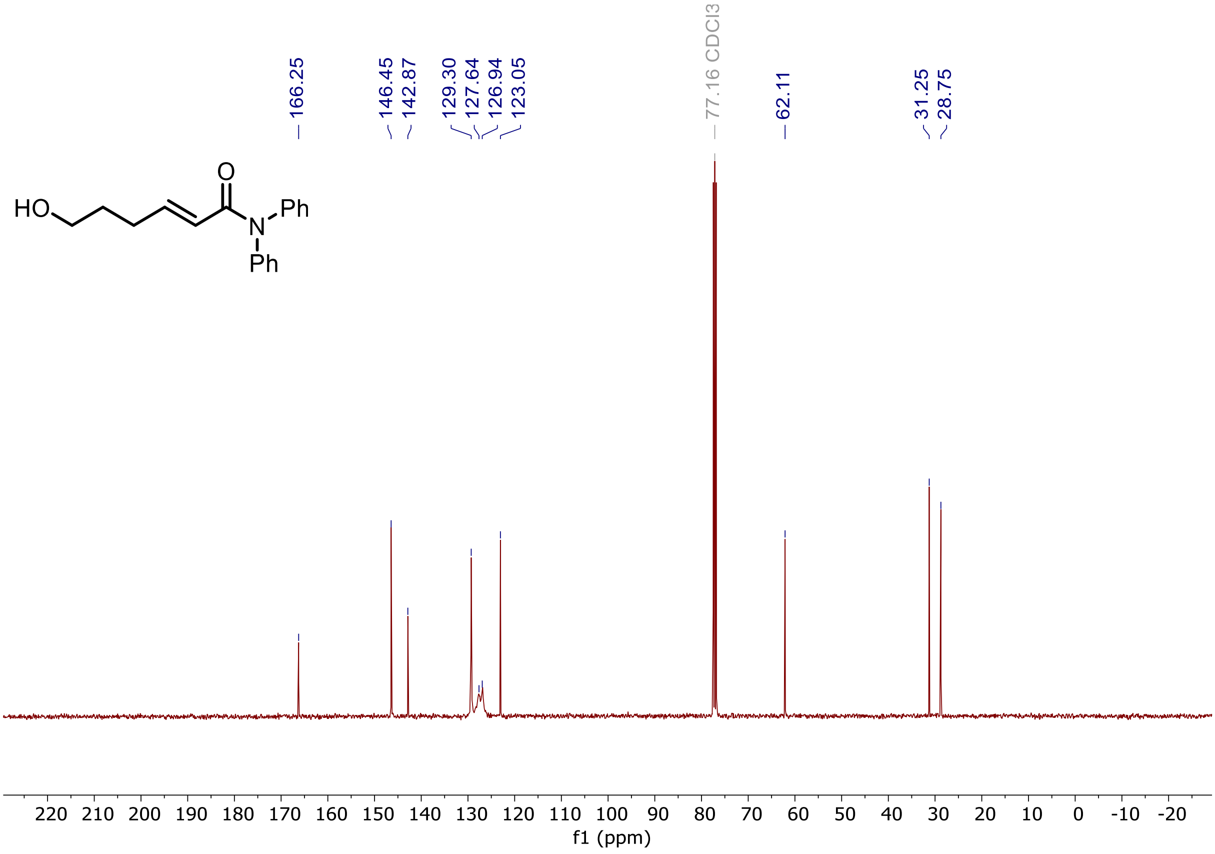

^{1}H NMR: (400 MHz, $CDCl_3$, 298K) of **3f**

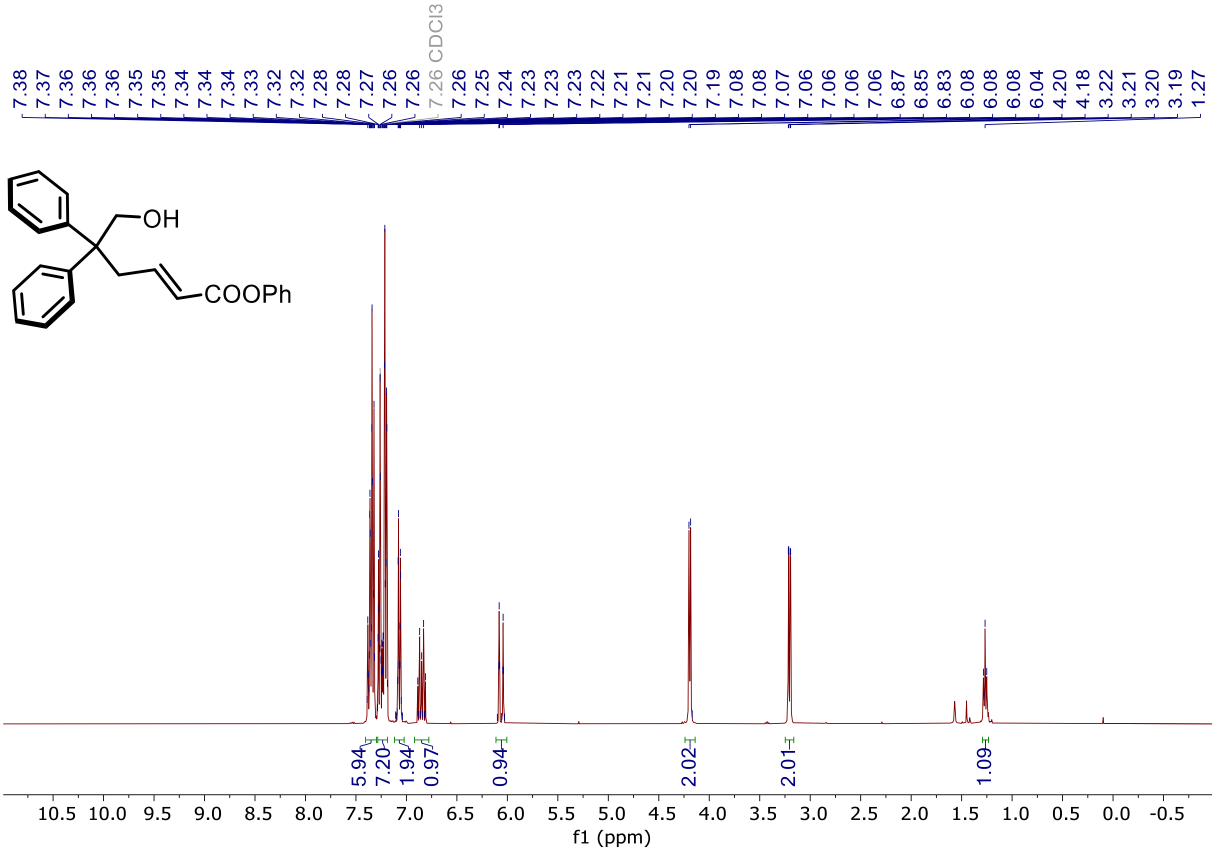

^{13}C NMR: (101 MHz, $CDCl_3$, 298K) of **3f**

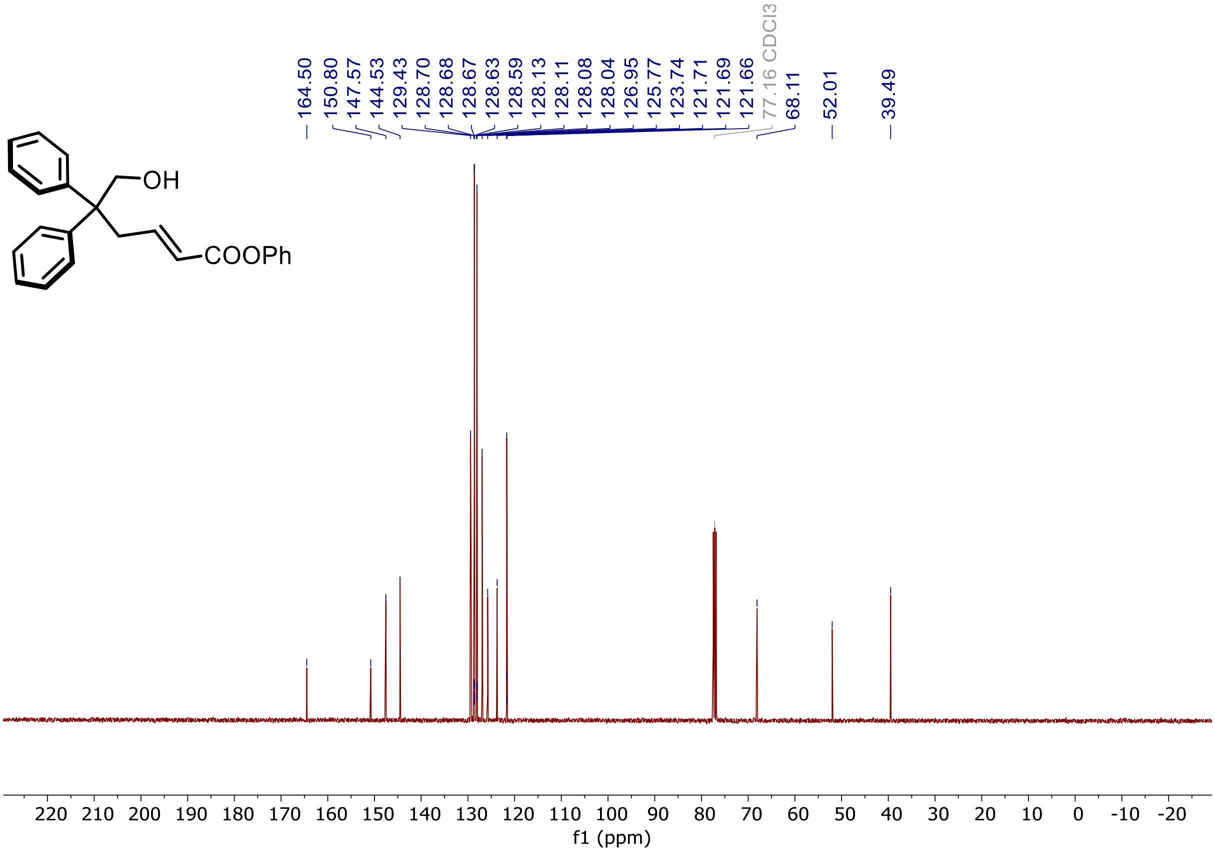

^{1}H NMR: (400 MHz, $CDCl_3$, 298K) of **3g**

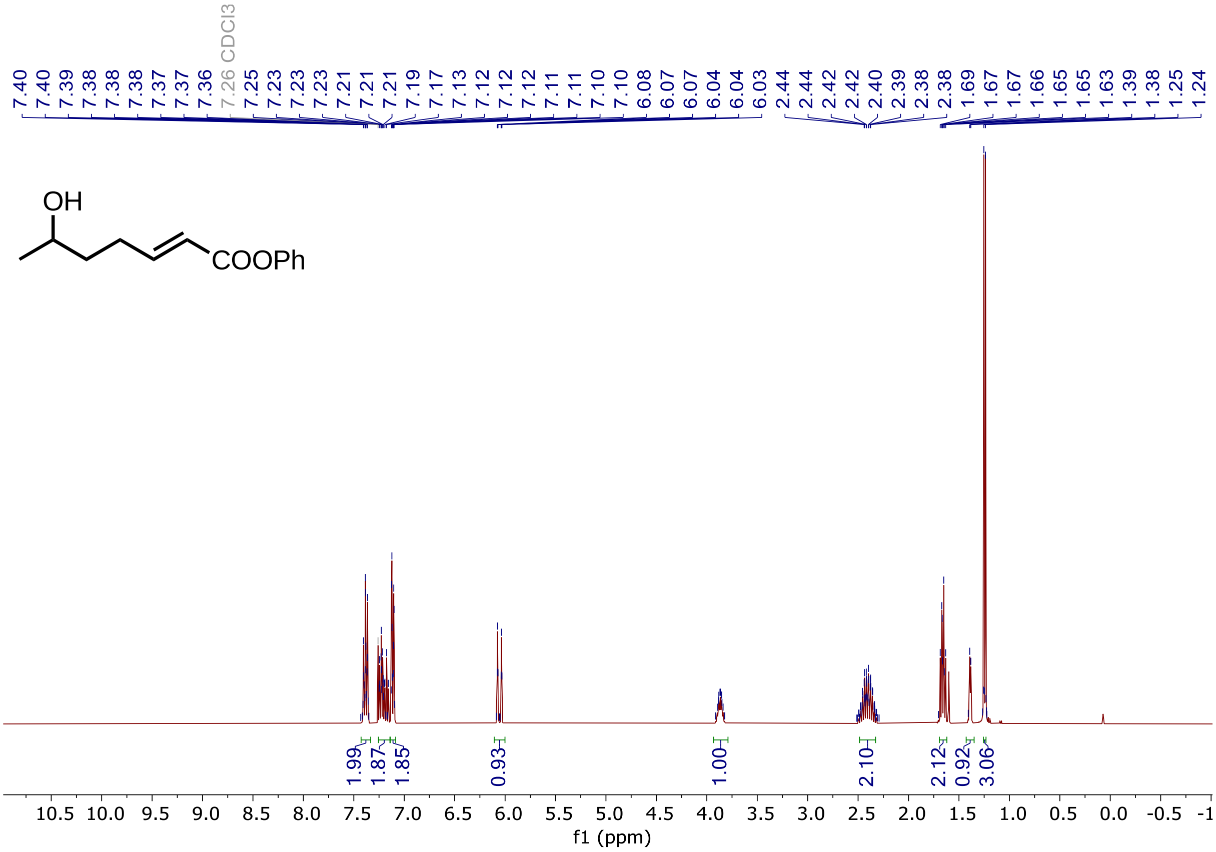

^{13}C NMR: (101 MHz, $CDCl_3$, 298K) of **3g**

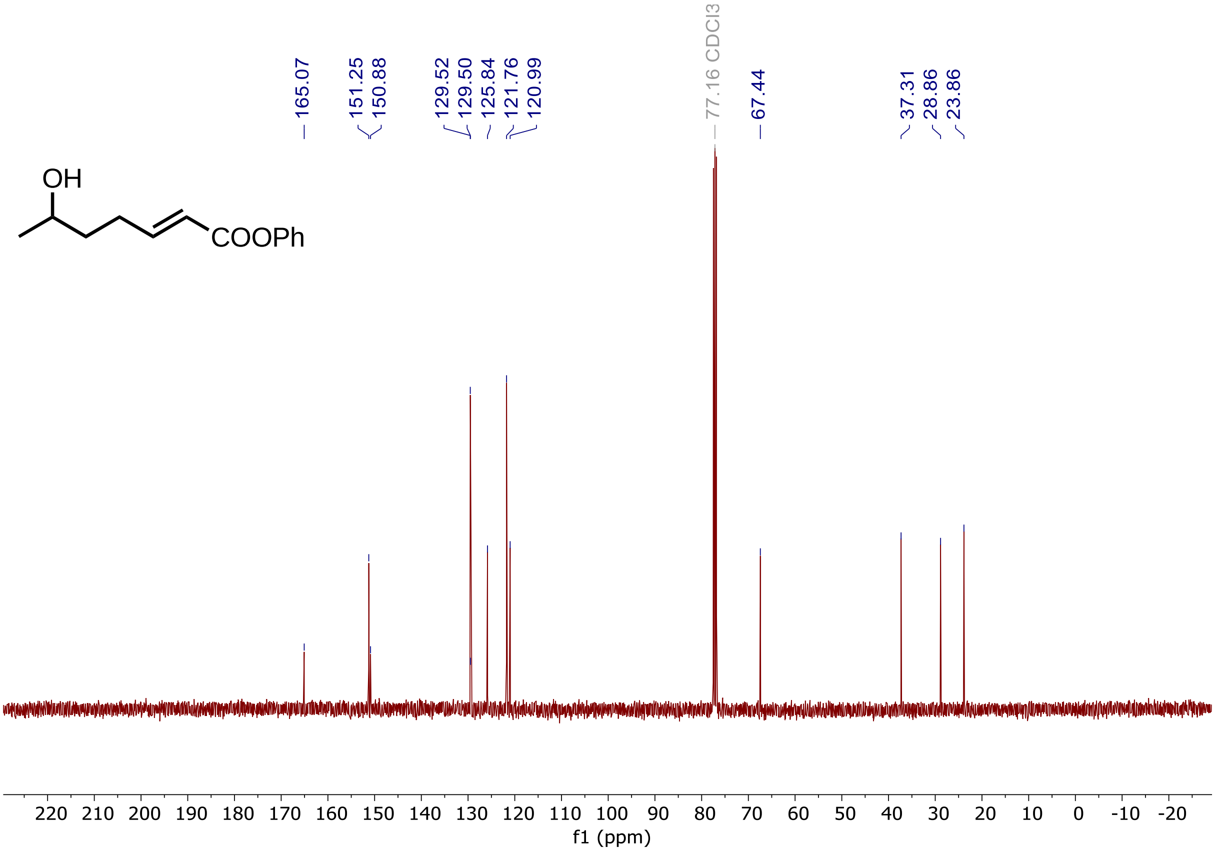

^{1}H NMR: (400 MHz, $CDCl_3$, 298K) of **3h**

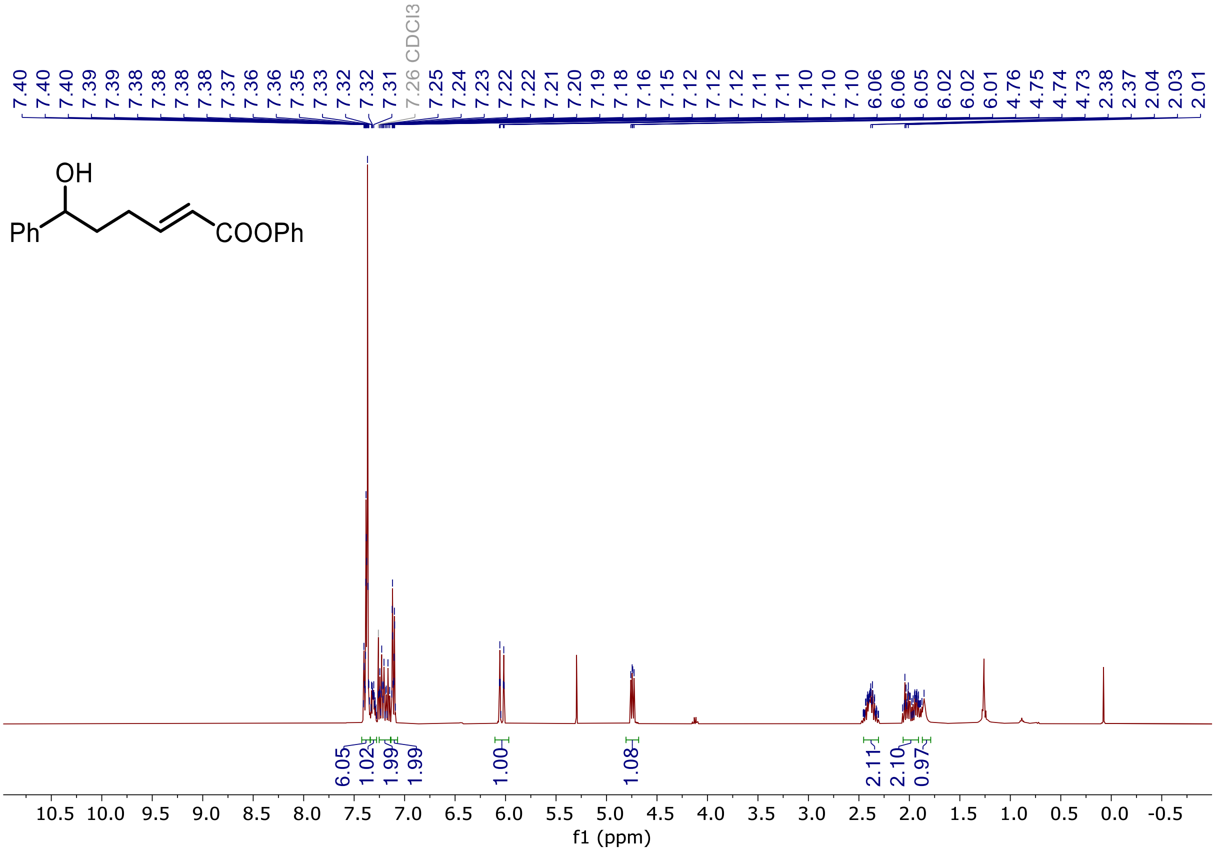

^{13}C NMR: (101 MHz, $CDCl_3$, 298K) of **3h**

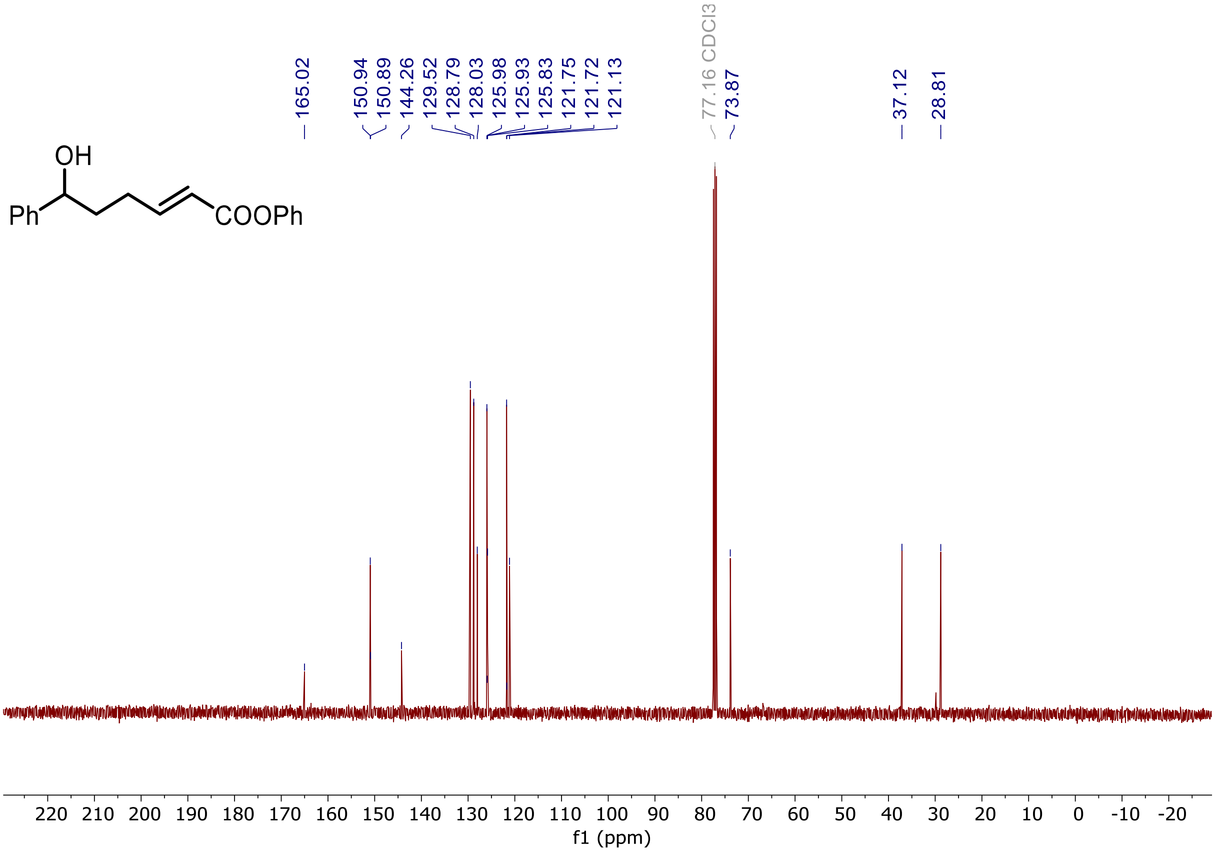

^{1}H NMR: (400 MHz, $CDCl_3$, 298K) of **3i**

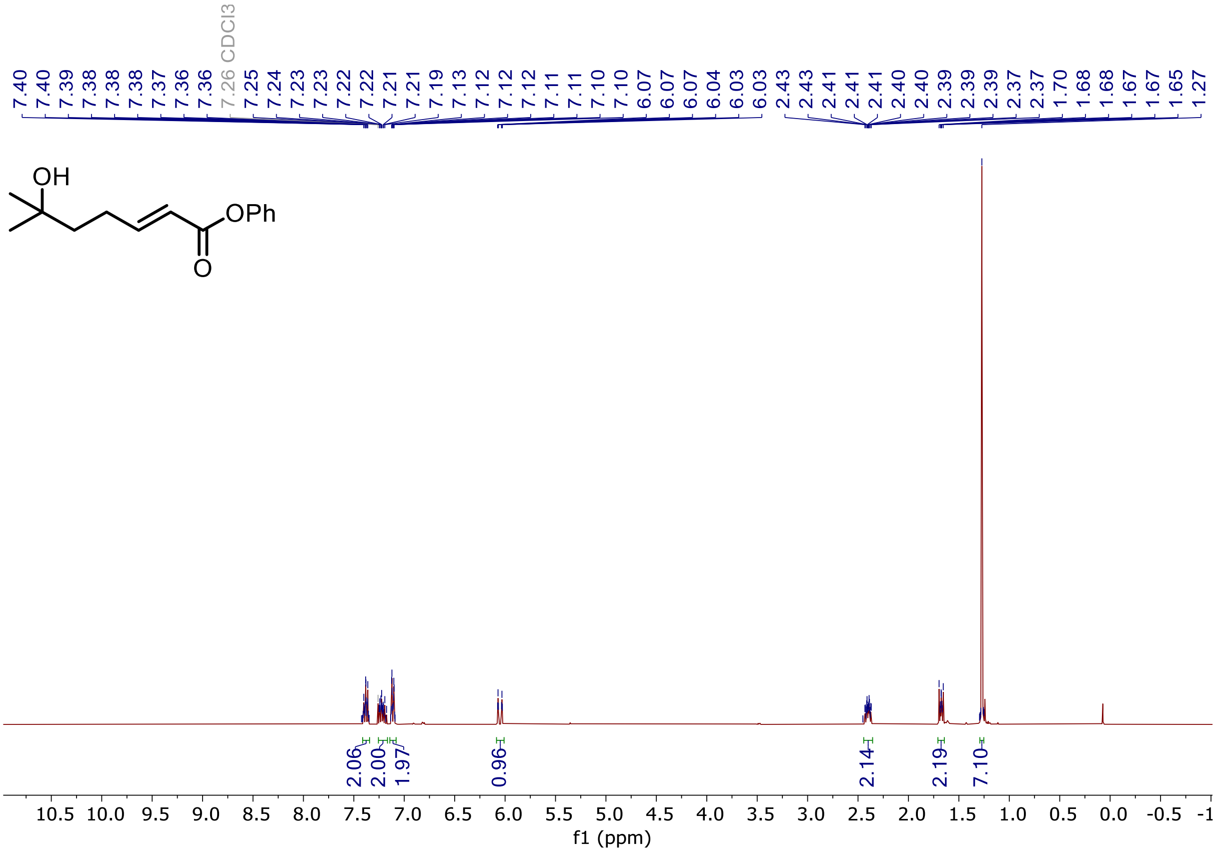

^{13}C NMR: (101 MHz, $CDCl_3$, 298K) of **3i**

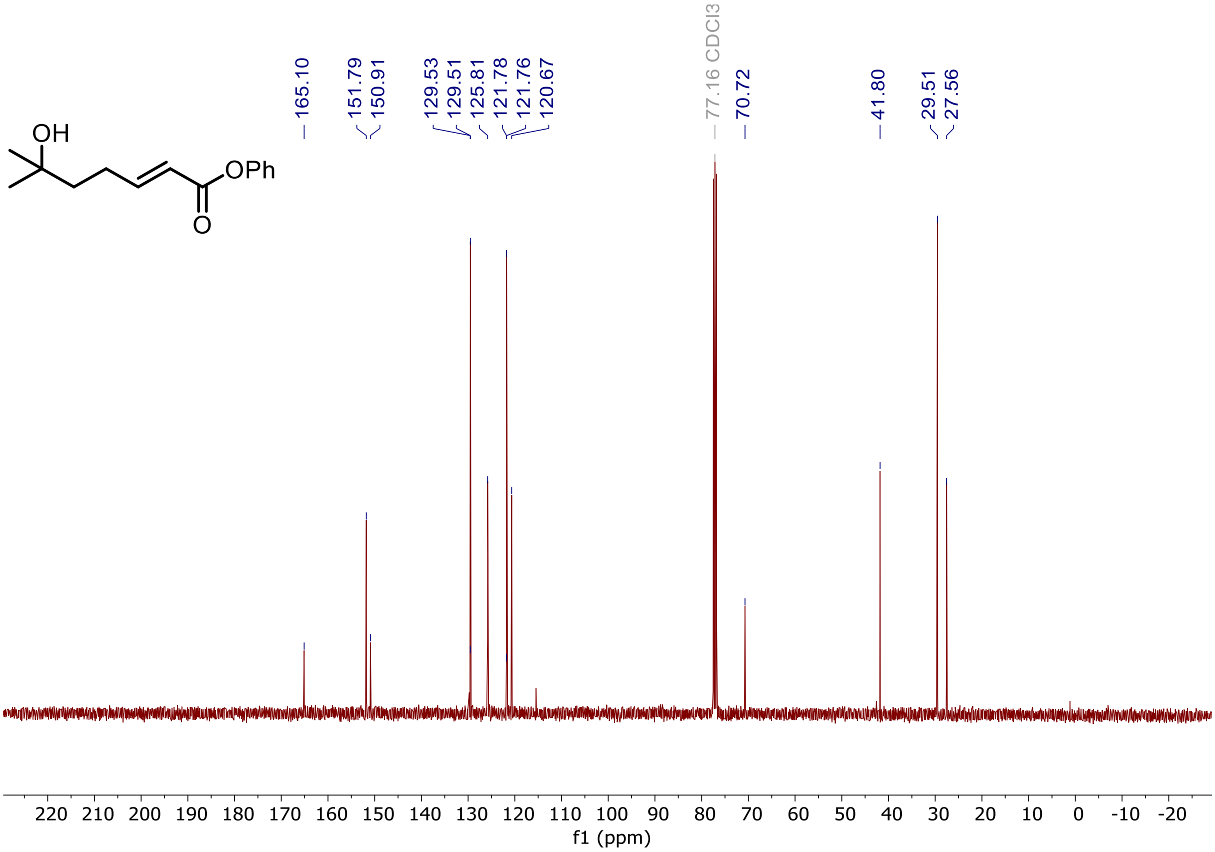

^{1}H NMR: (400 MHz, $CDCl_3$, 298K) of **3j**

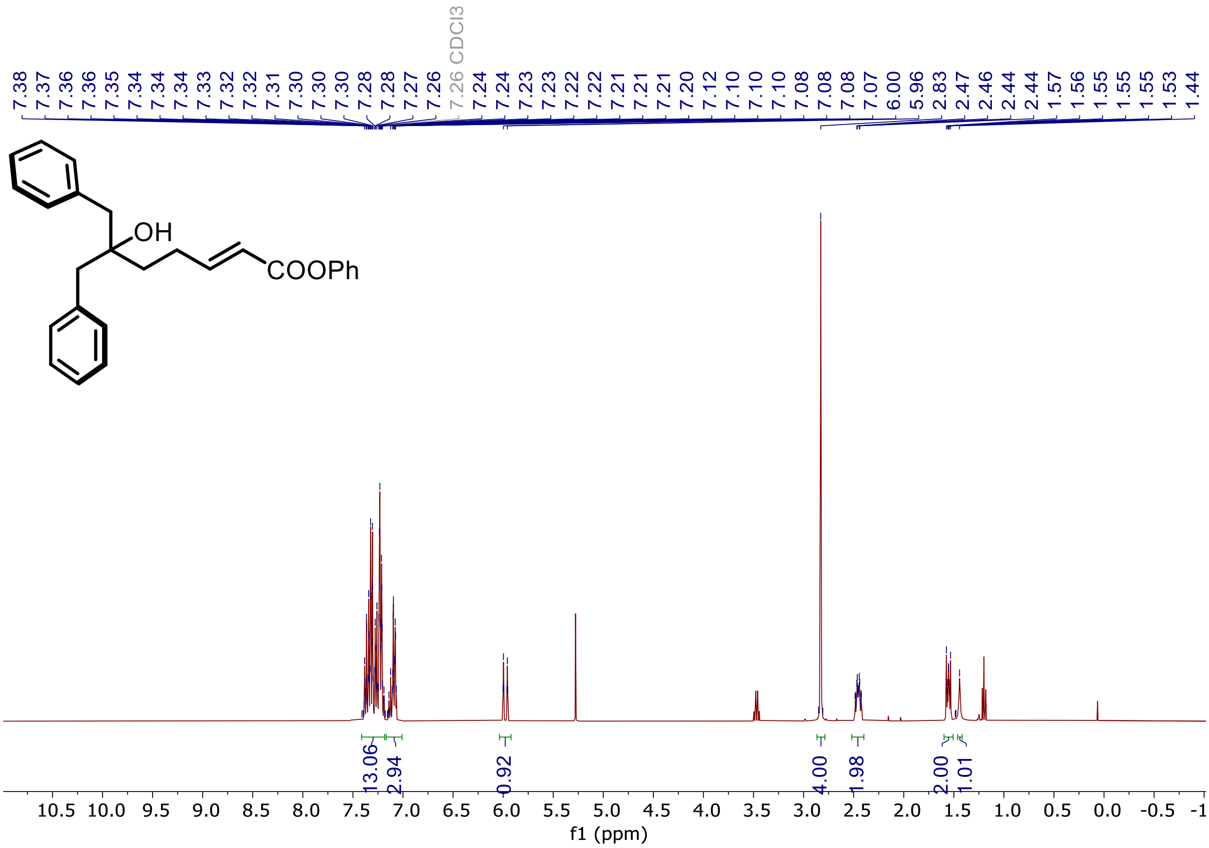

^{13}C NMR: (101 MHz, $CDCl_3$, 298K) of **3j**

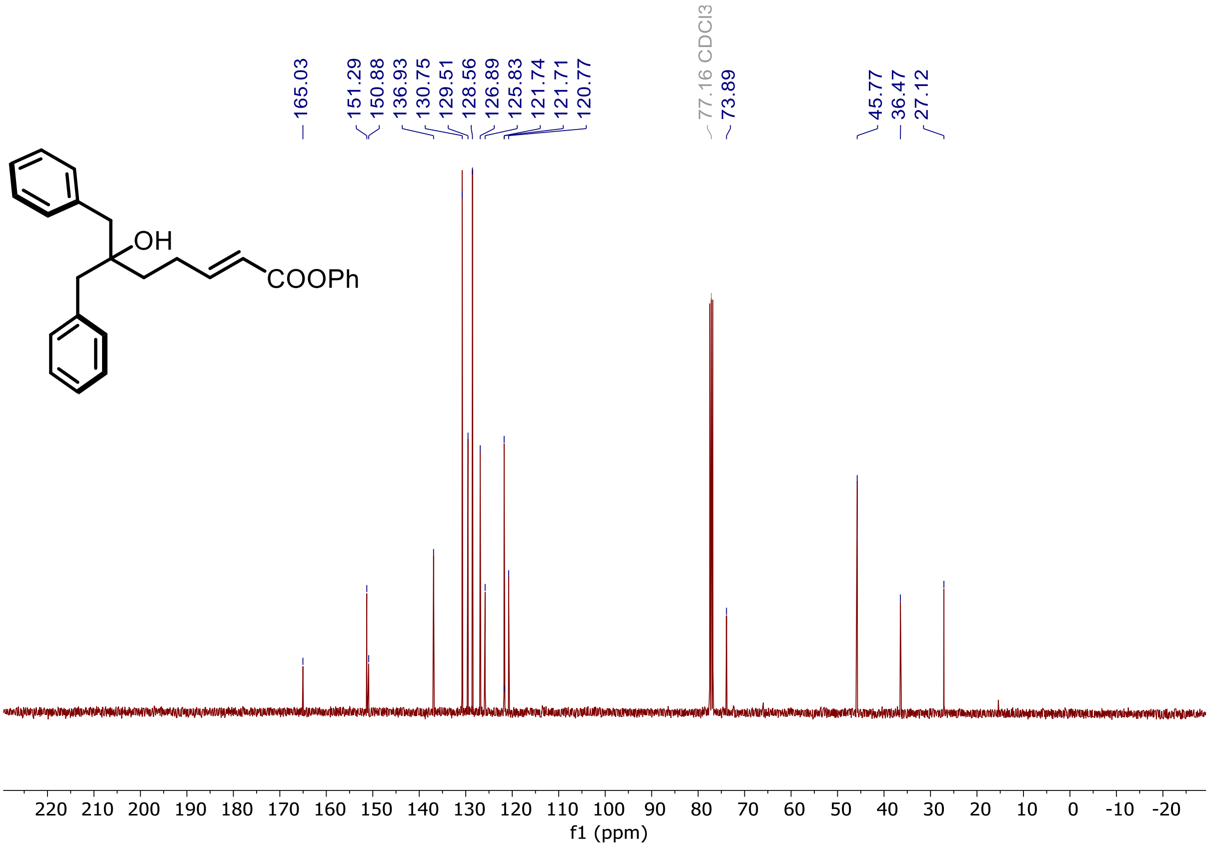

^{1}H NMR: (400 MHz, $CDCl_3$, 298K) of **S16**

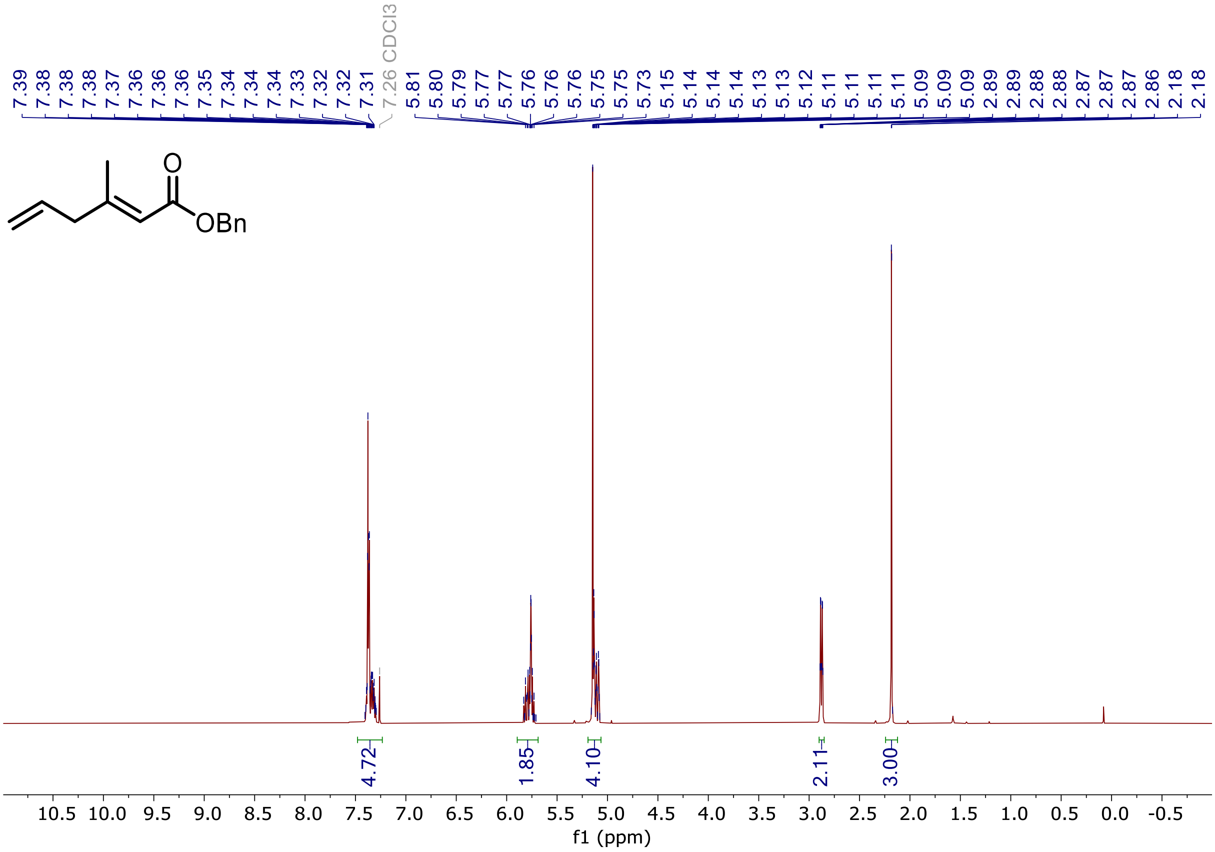

^{13}C NMR: (101 MHz, $CDCl_3$, 298K) of **S16**

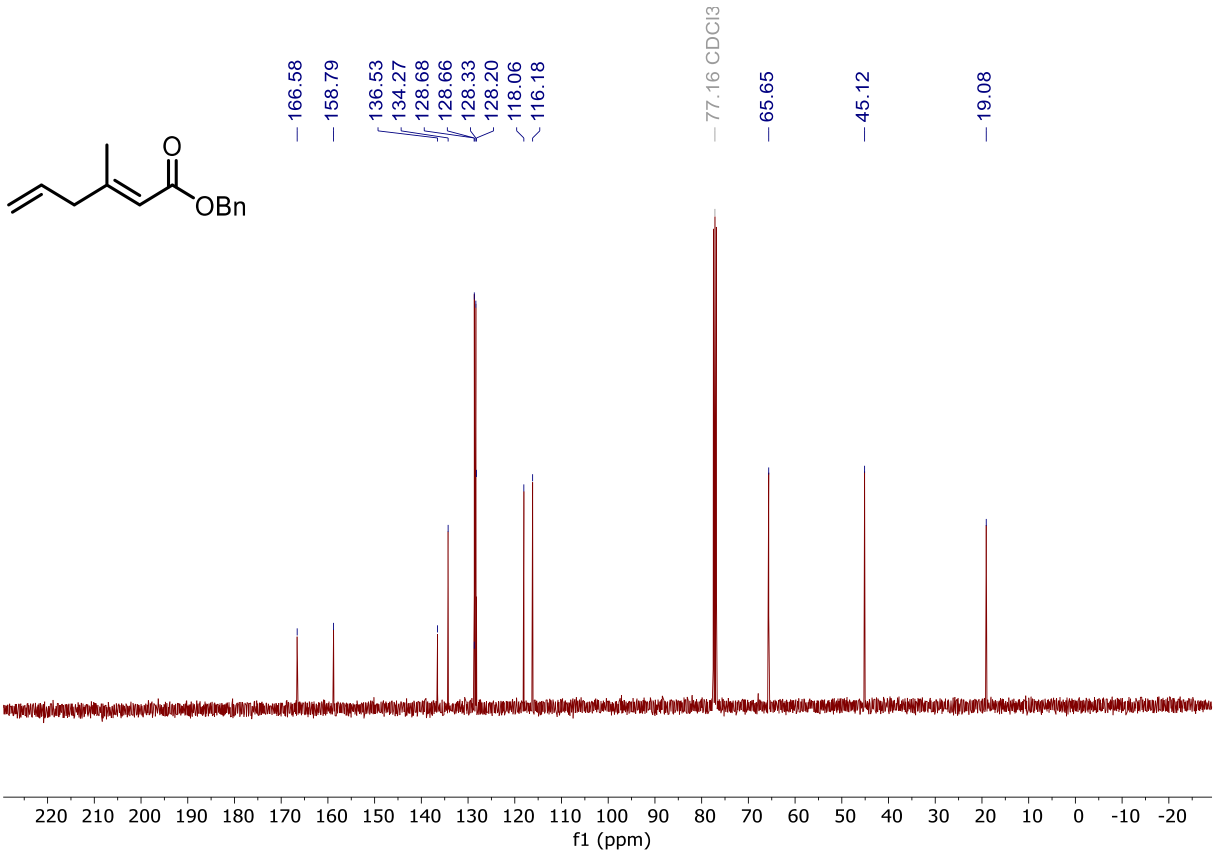

^1H NMR: (400 MHz, $CDCl_3$, 298K) of **3k**

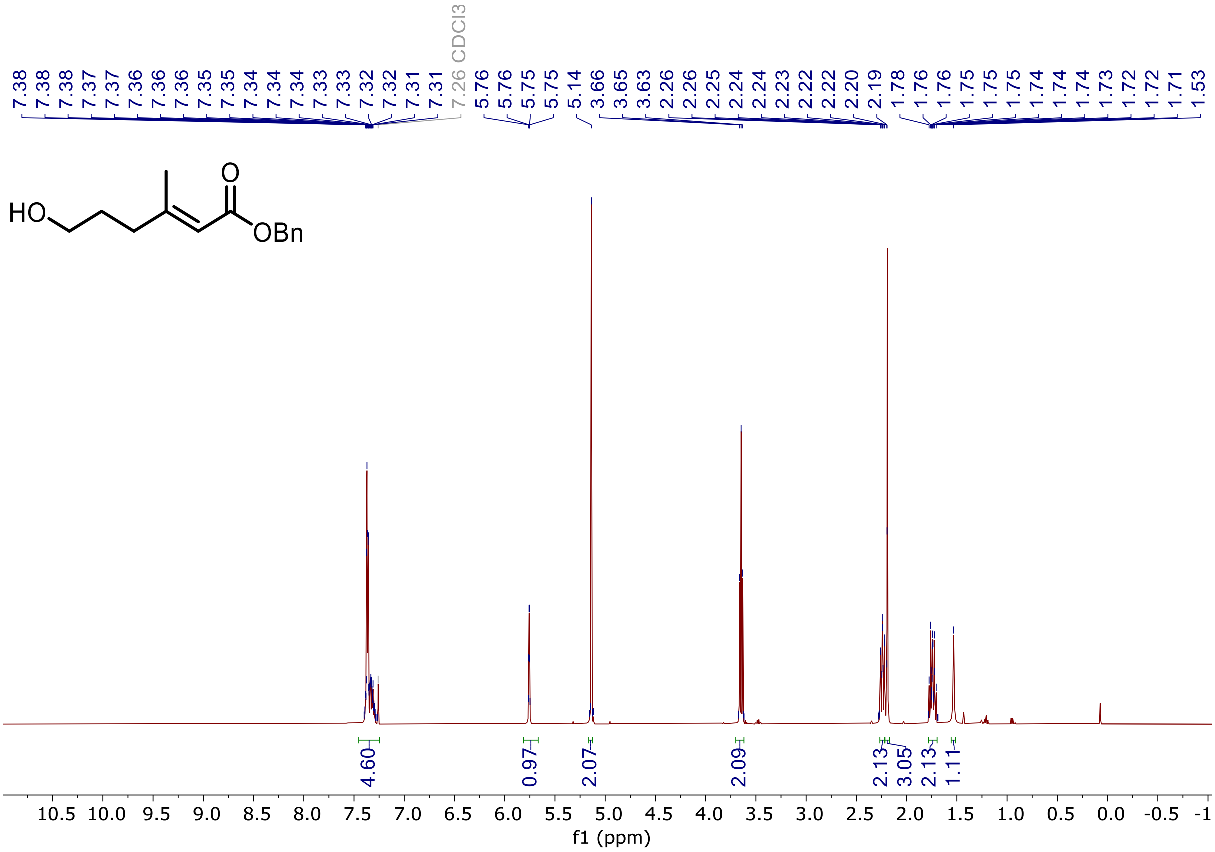

^{13}C NMR: (101 MHz, $CDCl_3$, 298K) of **3k**

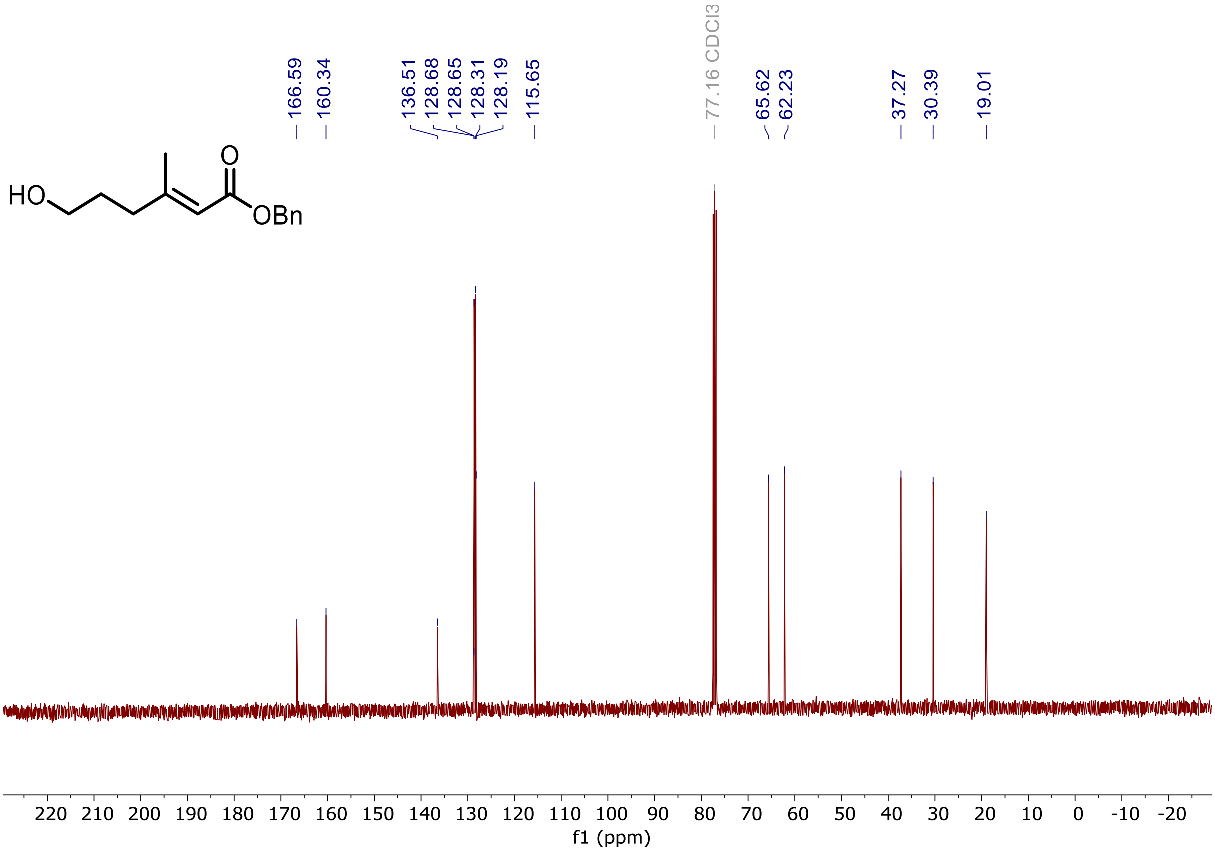

^{1}H NMR: (400 MHz, $CDCl_3$, 298K) of **3l**

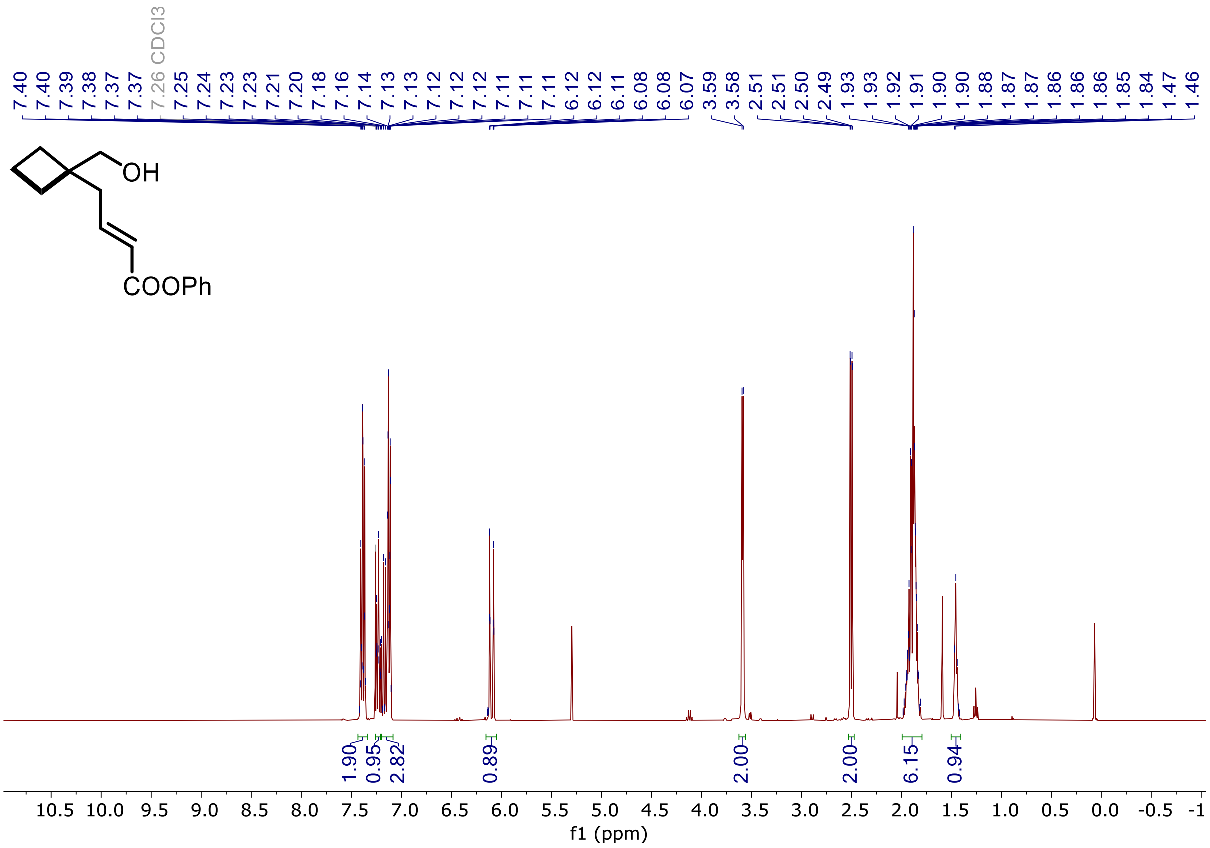

^{13}C NMR: (101 MHz, $CDCl_3$, 298K) of **3l**

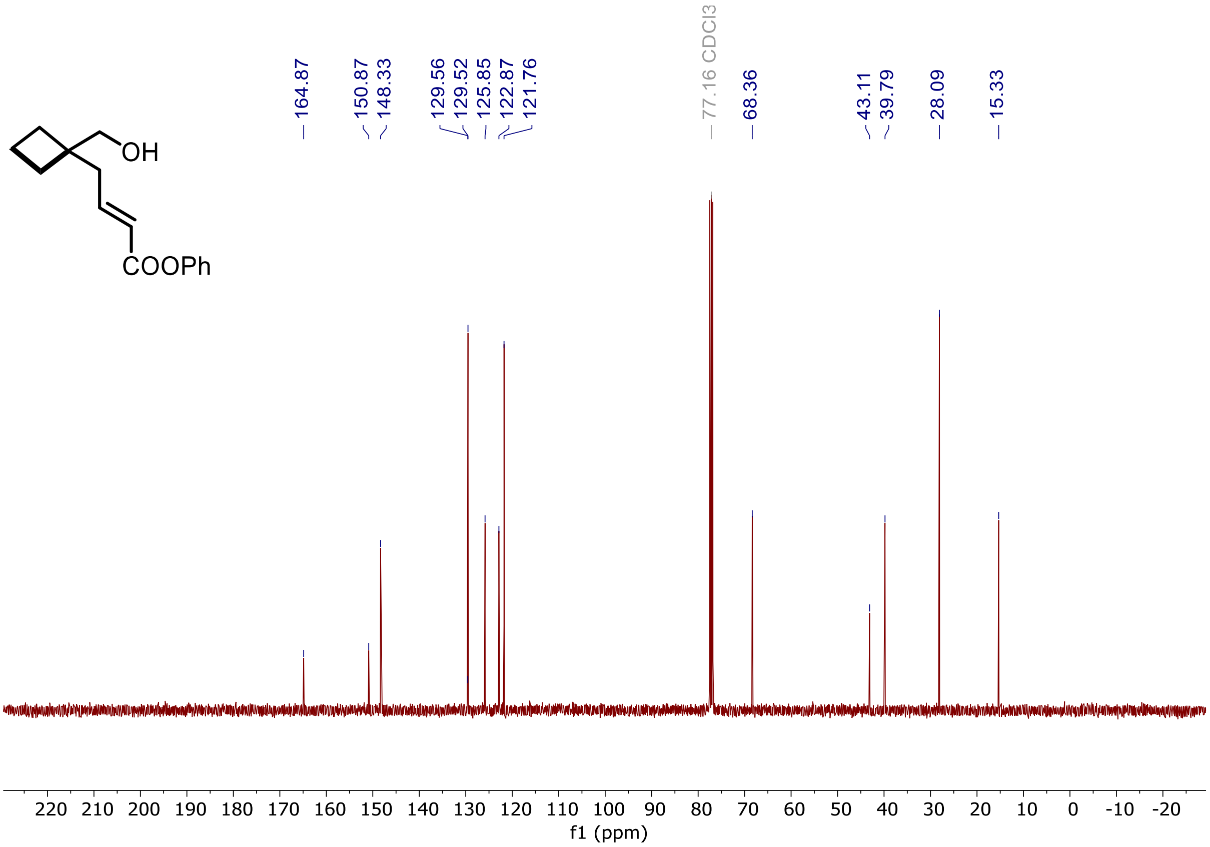

^{1}H NMR: (400 MHz, $CDCl_3$, 298K) of **3m**

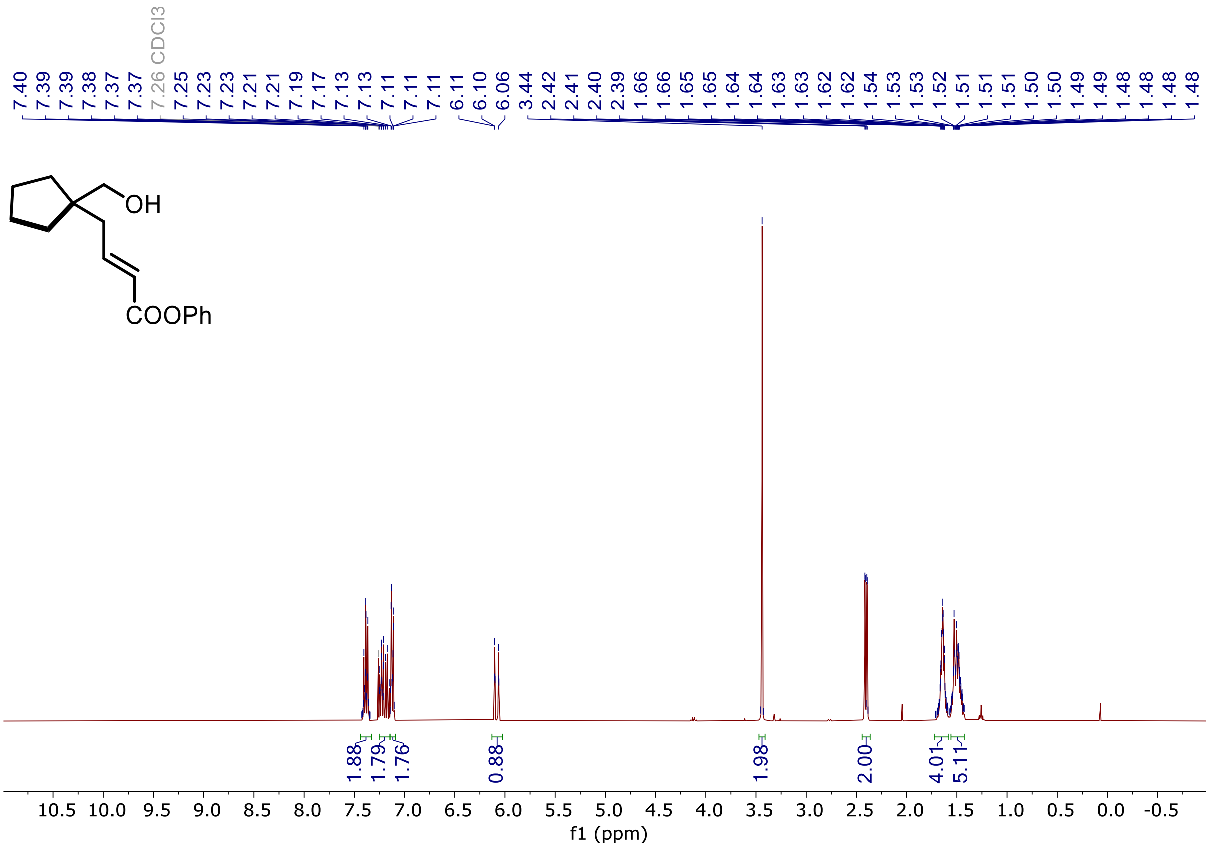

^{13}C NMR: (101 MHz, $CDCl_3$, 298K) of **3m**

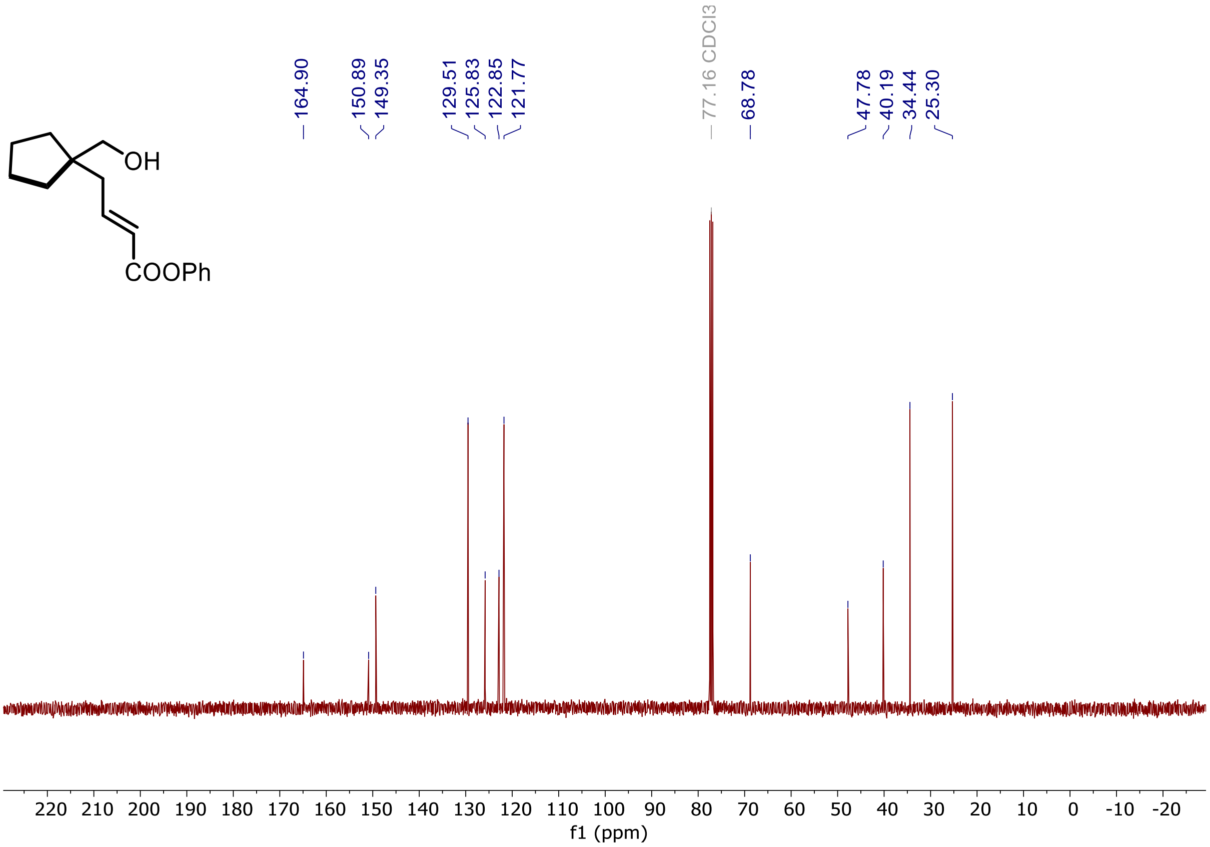

^{1}H NMR: (400 MHz, $CDCl_3$, 298K) of **3n**

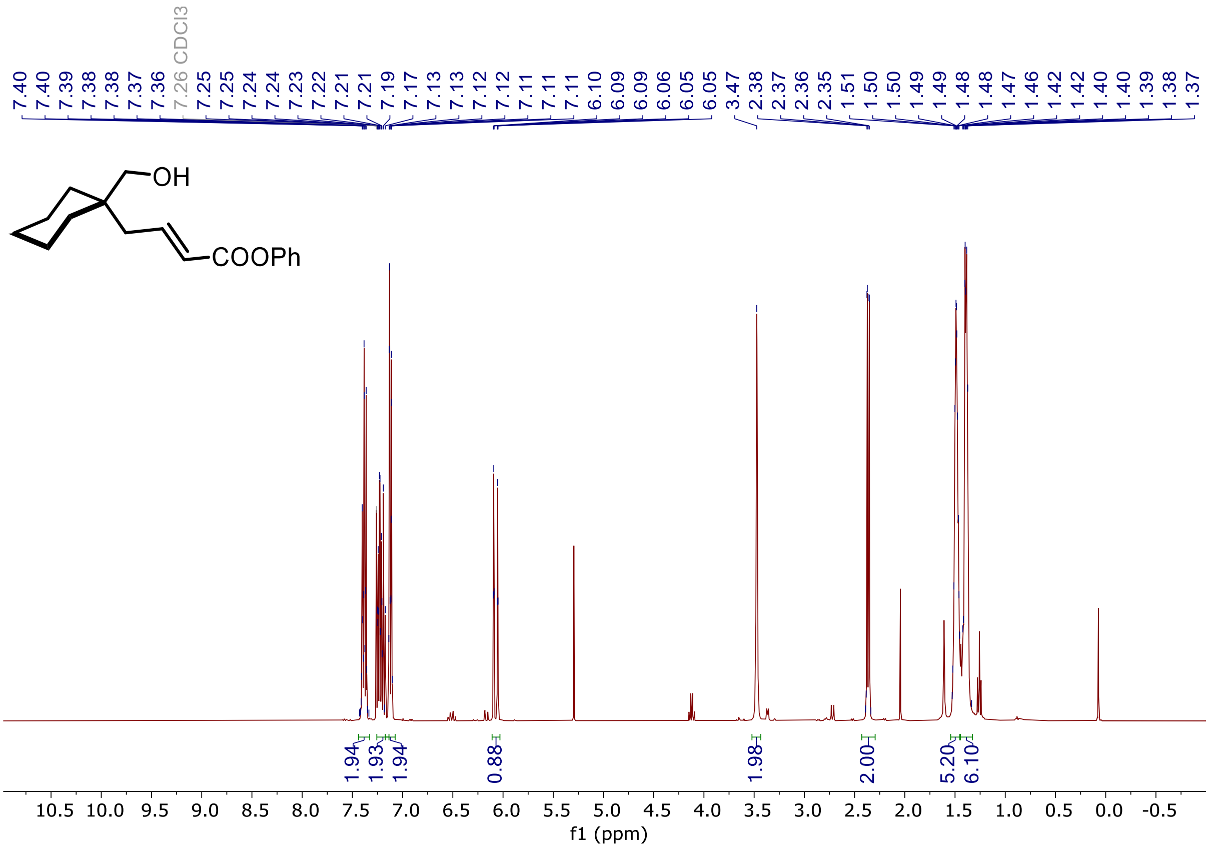

^{13}C NMR: (101 MHz, $CDCl_3$, 298K) of **3n**

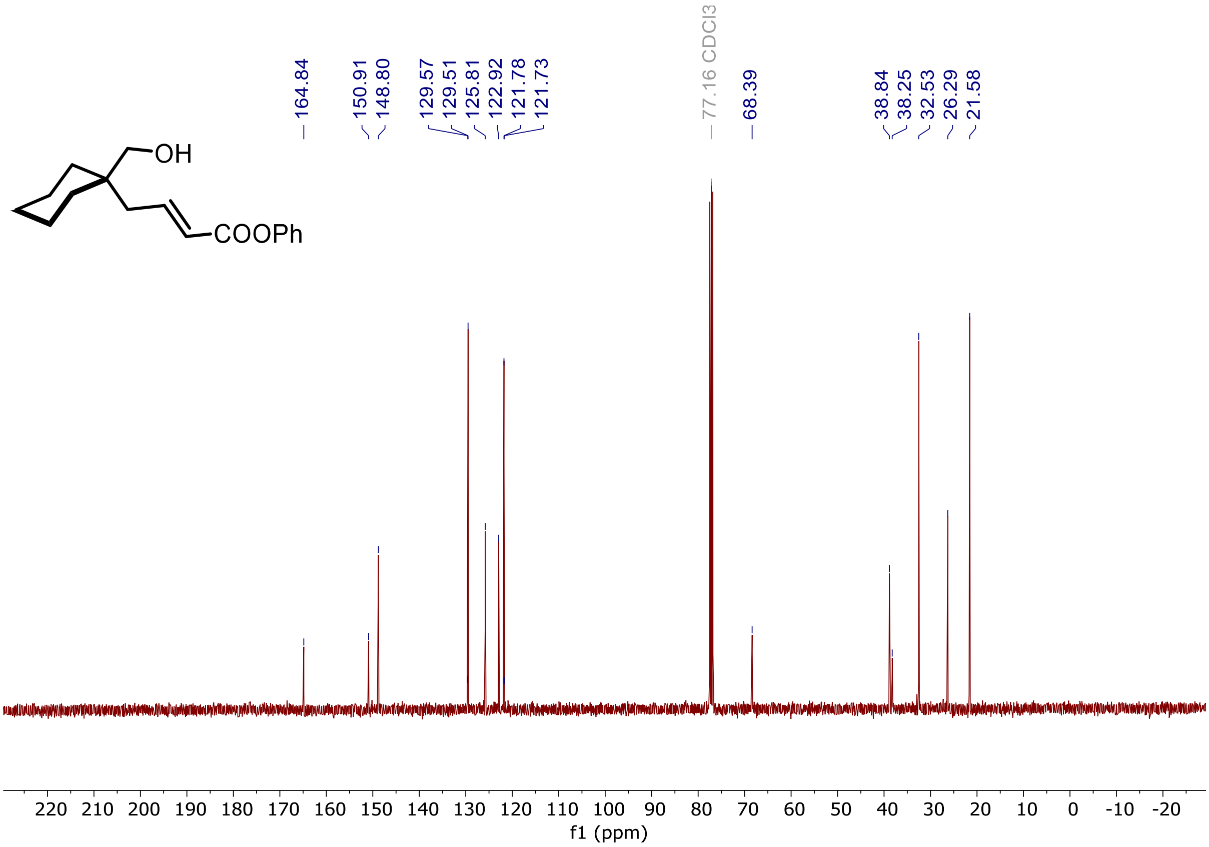

^{1}H NMR: (400 MHz, $CDCl_3$, 298K) of **3o**

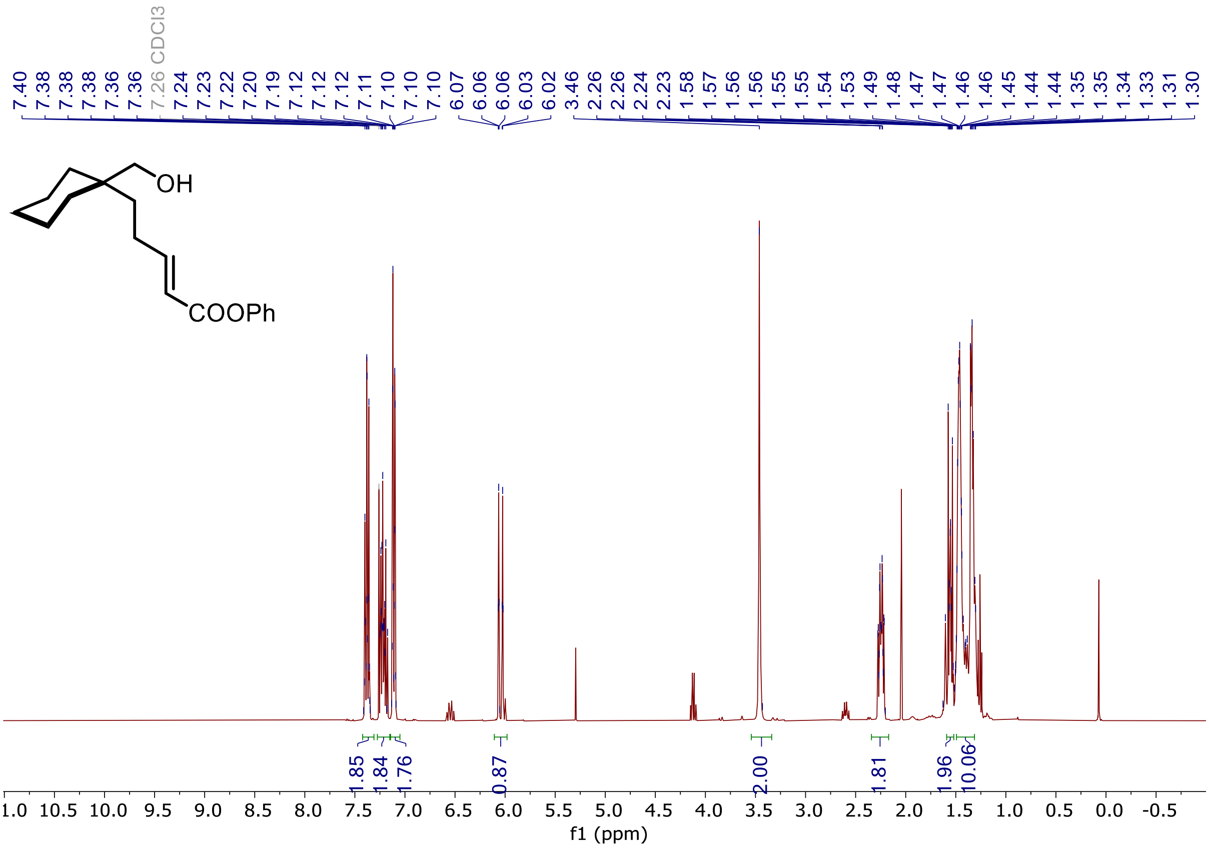

^{13}C NMR: (101 MHz, $CDCl_3$, 298K) of **3o**

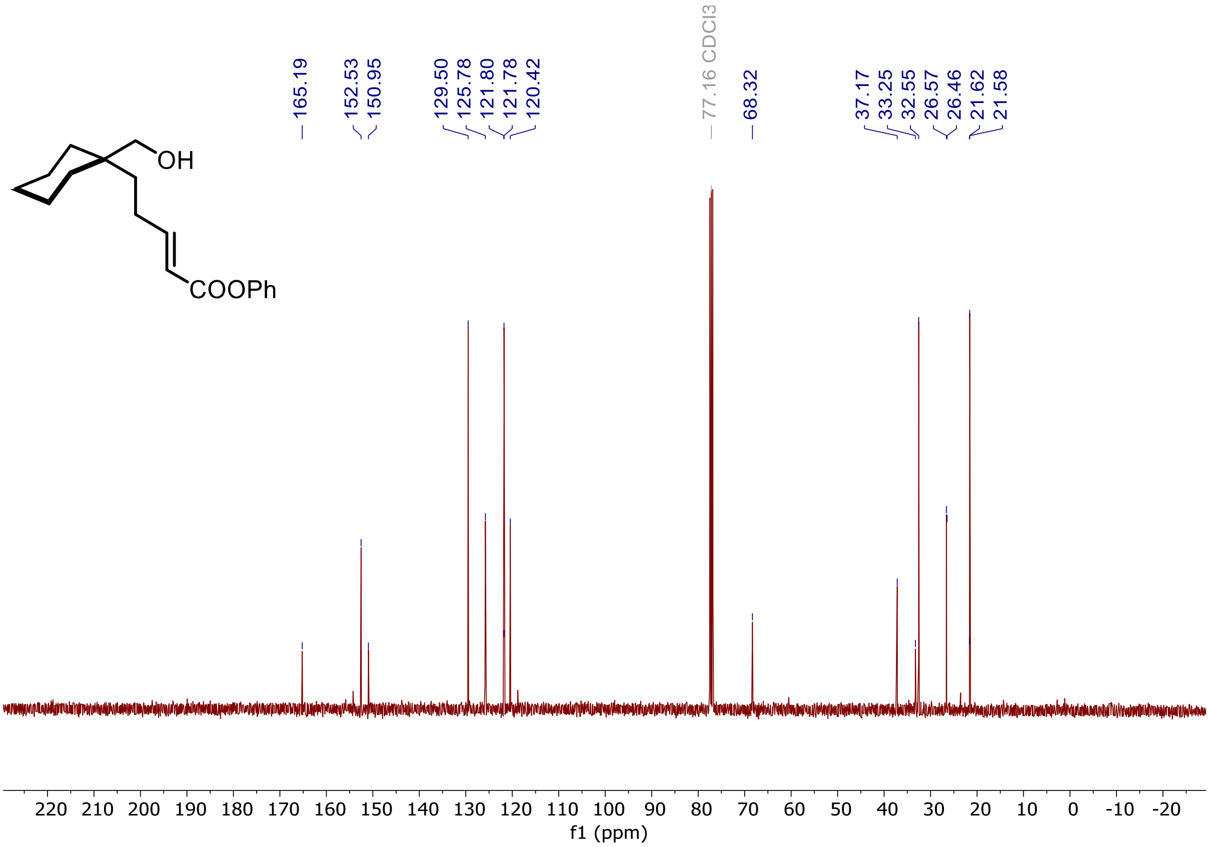

^{1}H NMR: (400 MHz, $CDCl_3$, 298K) of **S23**

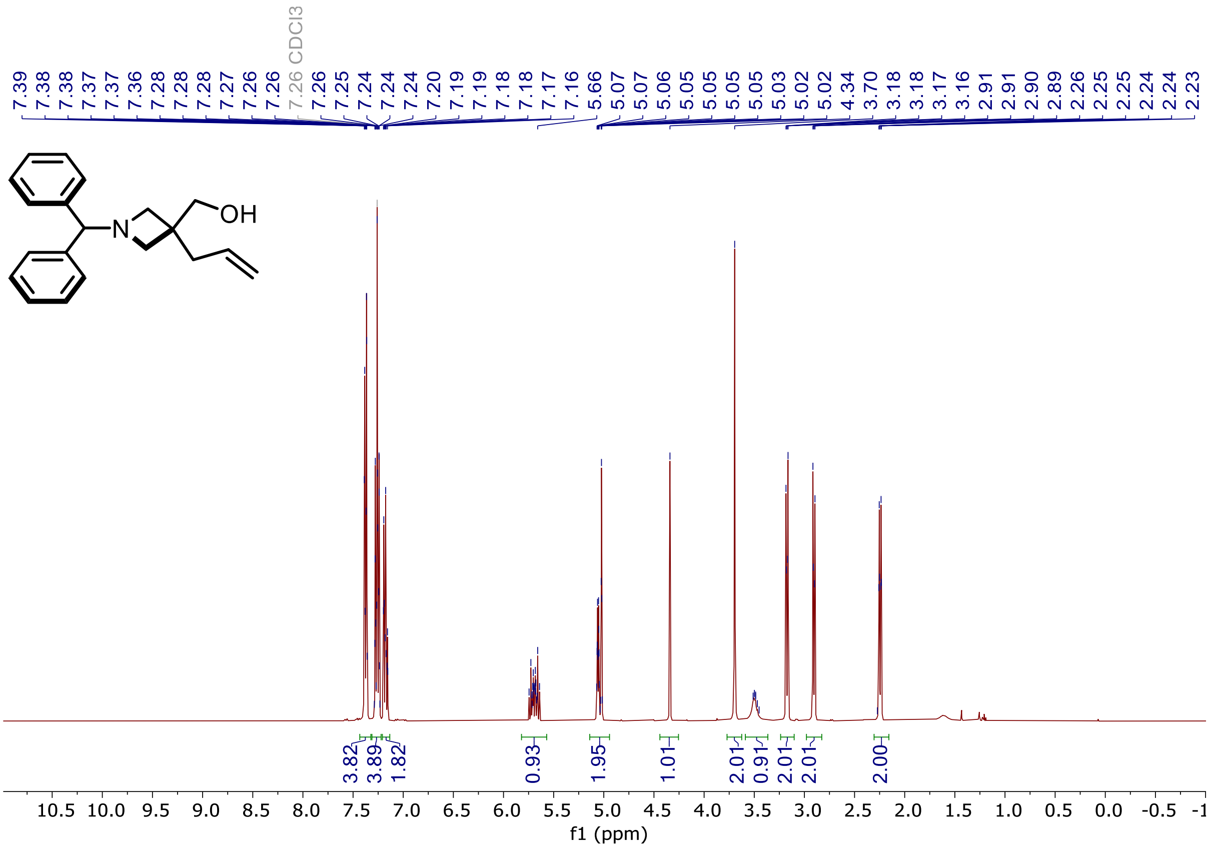

^{13}C NMR: (101 MHz, $CDCl_3$, 298K) of **S23**

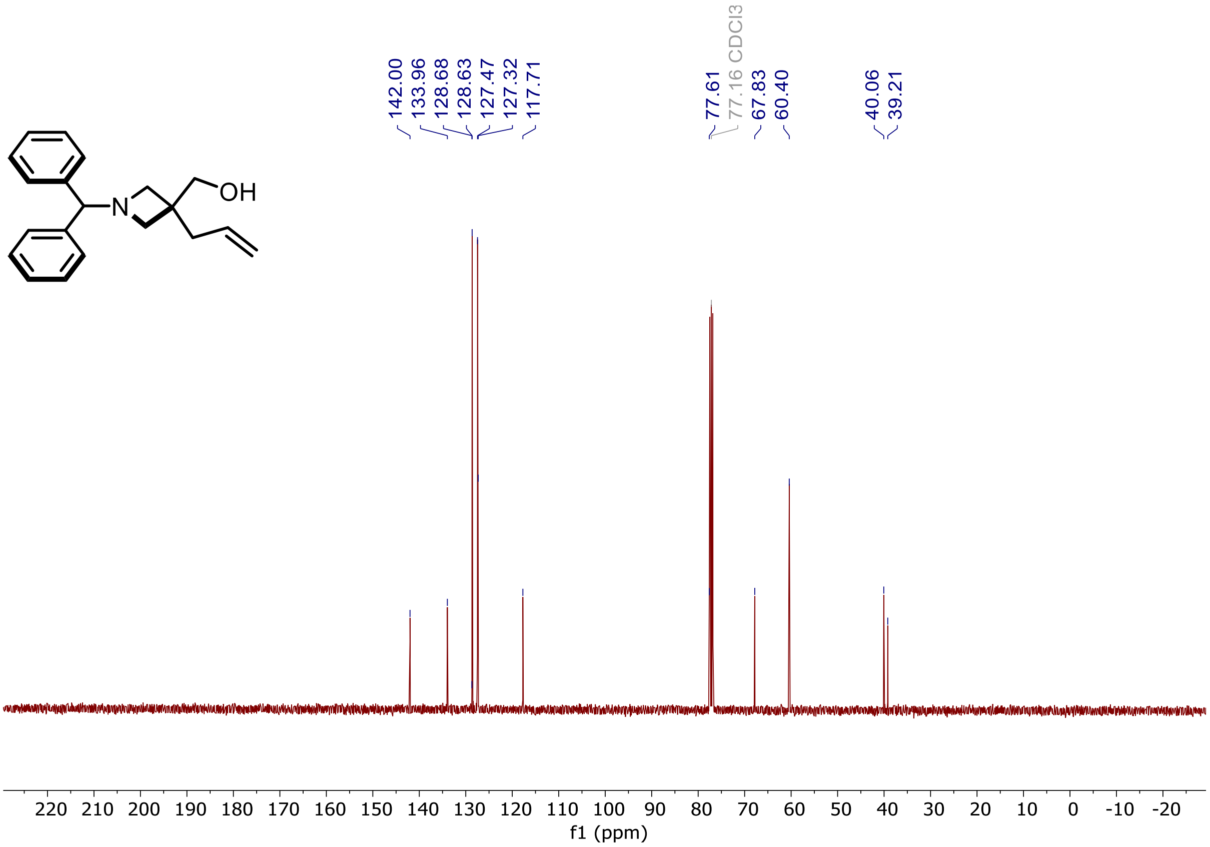

^{1}H NMR: (400 MHz, $CDCl_3$, 298K) of **3p**

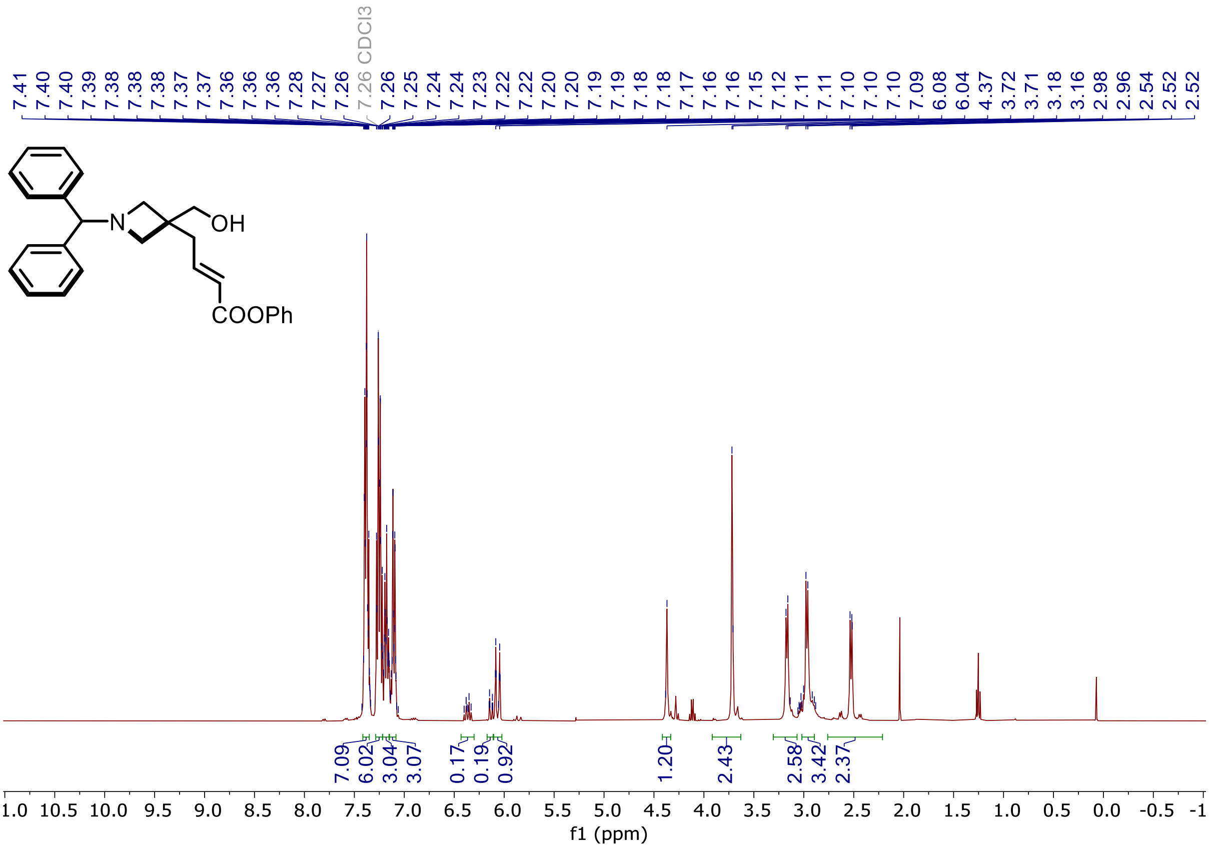

^{13}C NMR: (101 MHz, $CDCl_3$, 298K) of **3p**

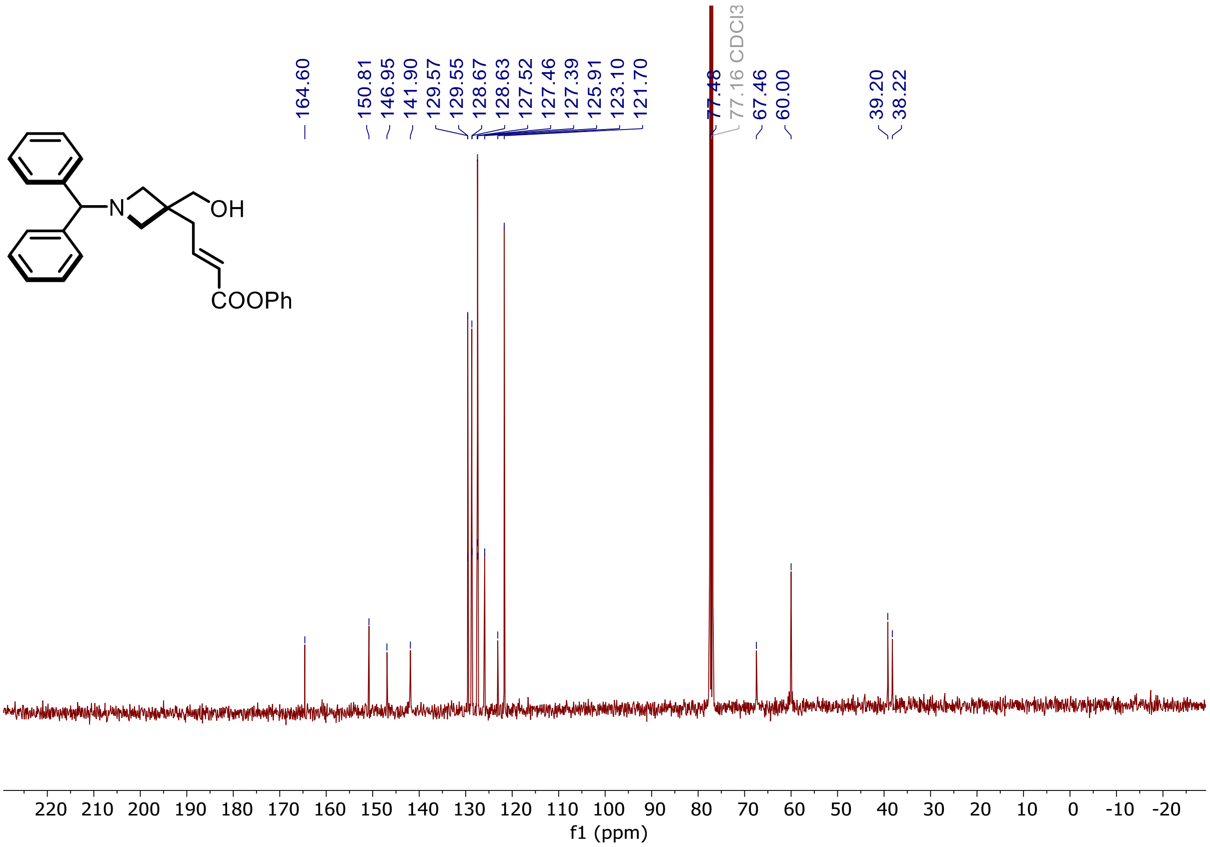

^{1}H NMR: (400 MHz, $CDCl_3$, 298K) of **3q**

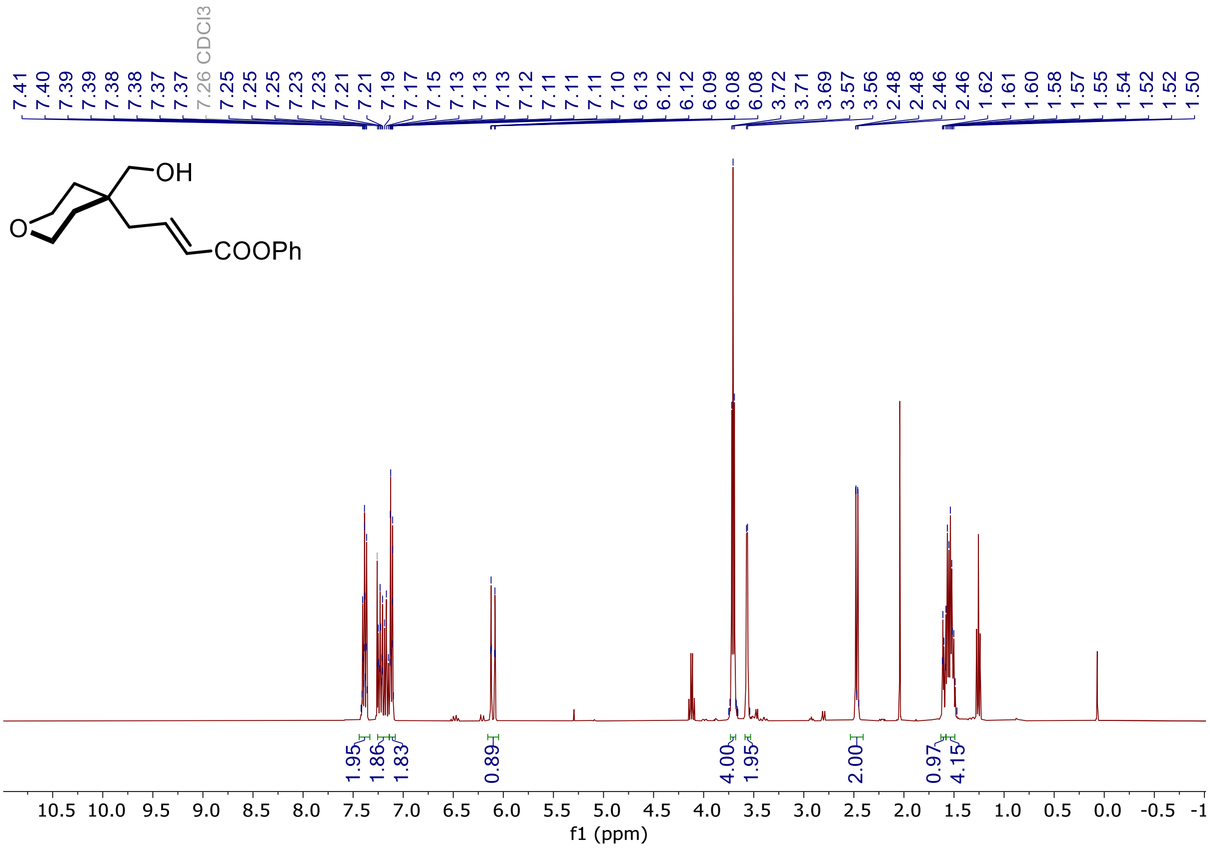

^{13}C NMR: (101 MHz, $CDCl_3$, 298K) of **3q**

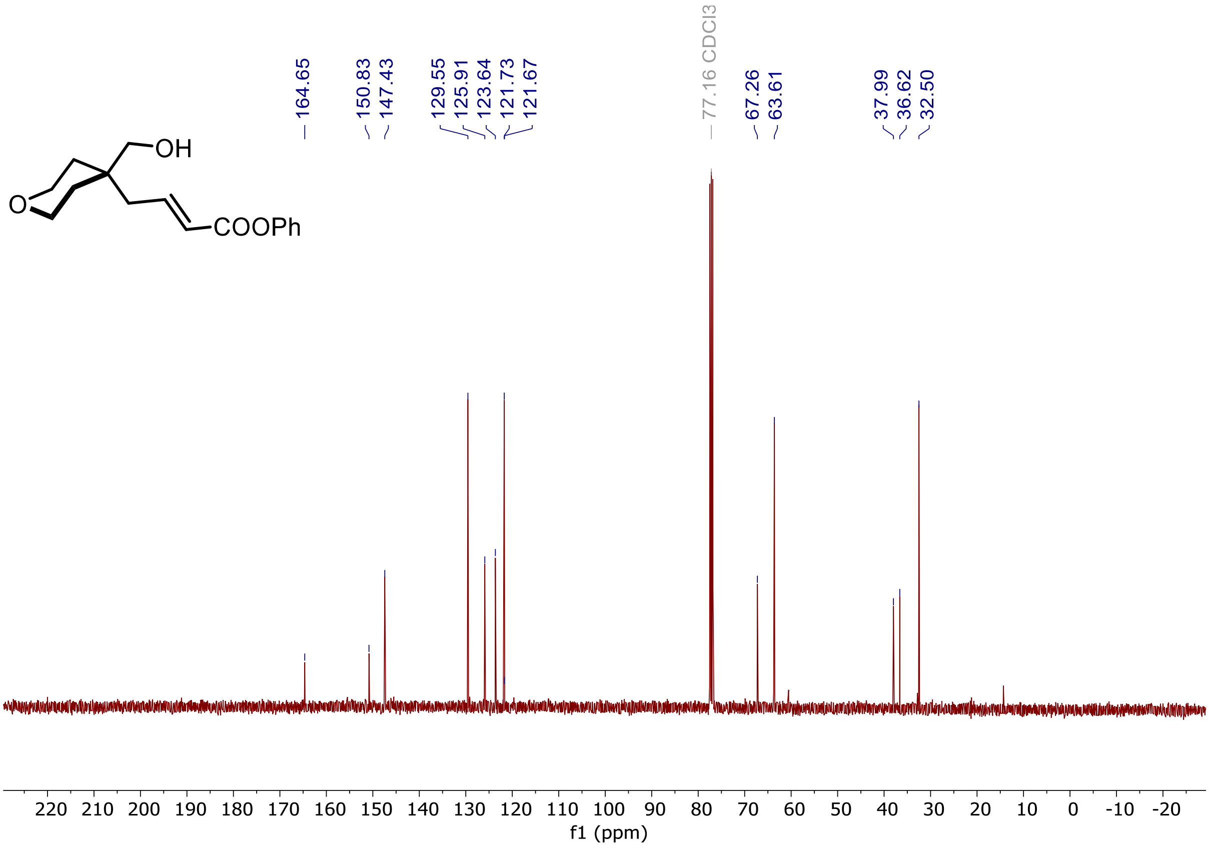

^{1}H NMR: (400 MHz, $CDCl_3$, 298K) of **3r**

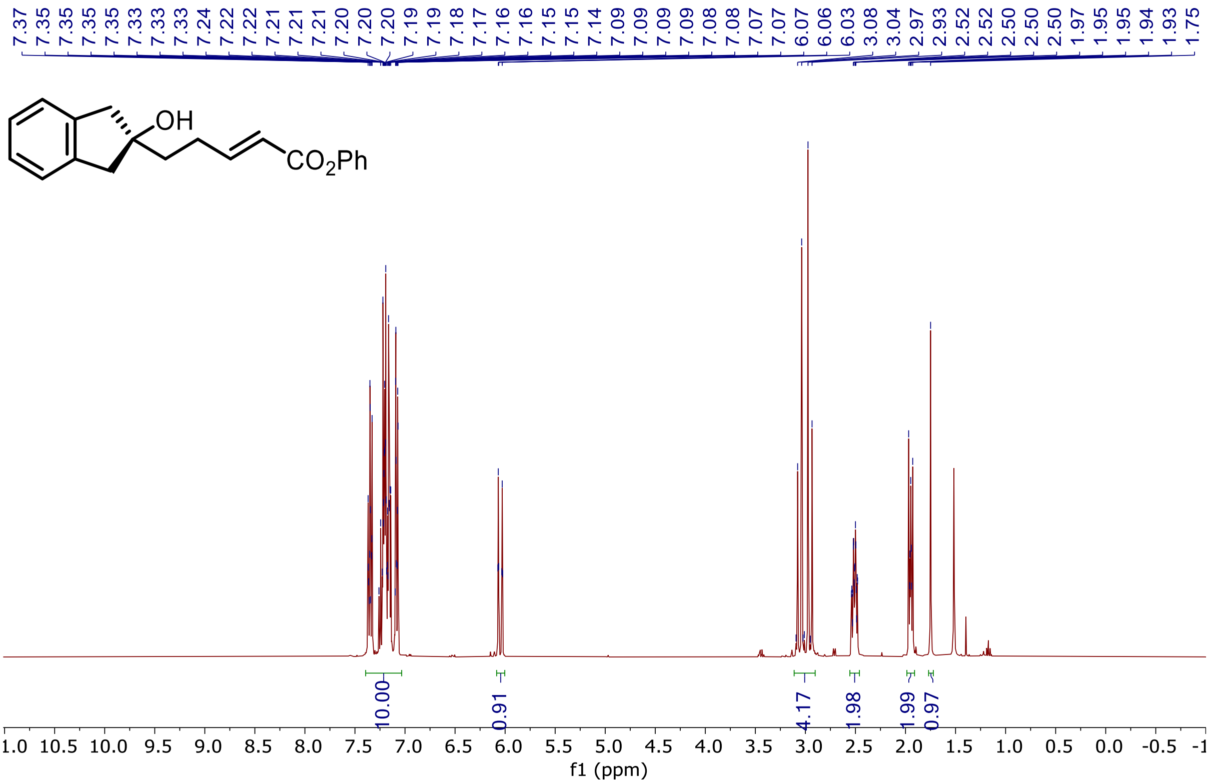

^{13}C NMR: (101 MHz, $CDCl_3$, 298K) of **3r**

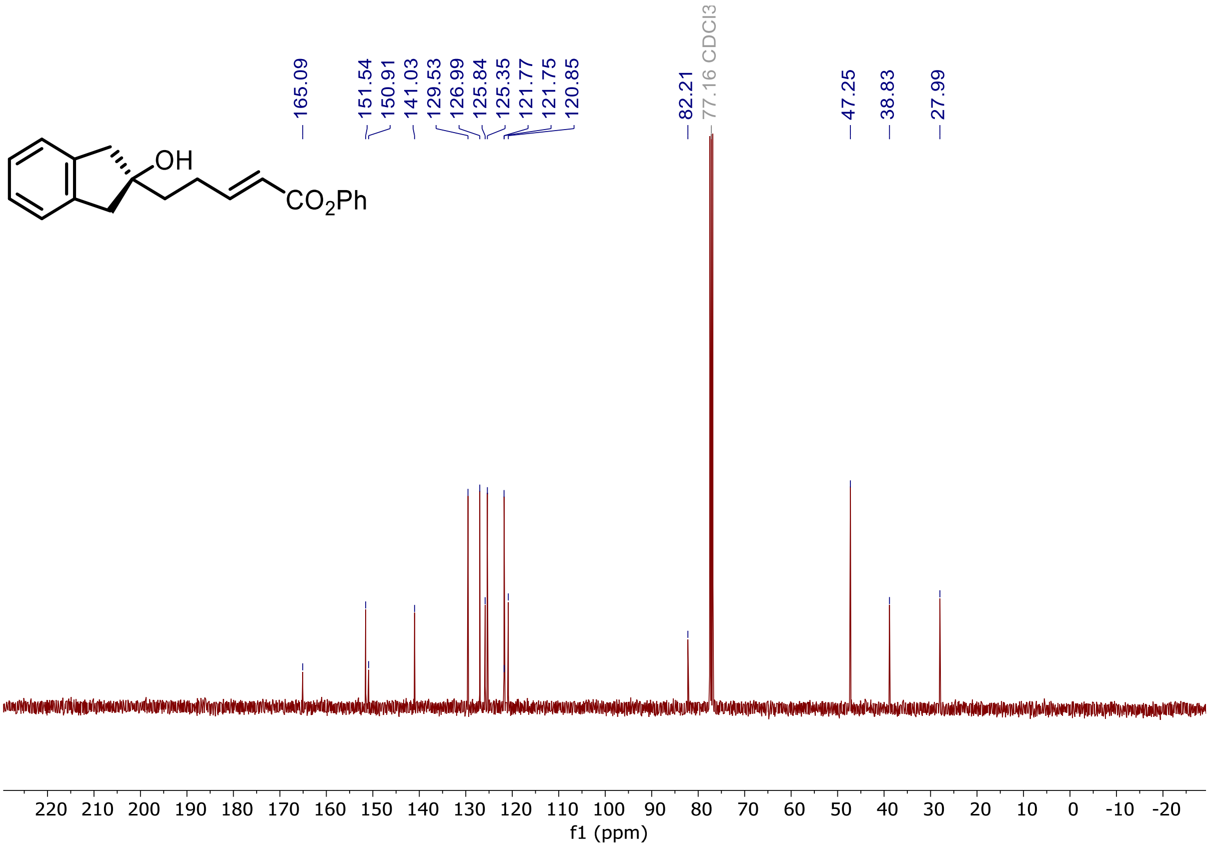

^{1}H NMR: (400 MHz, $CDCl_3$, 298K) of **3s**

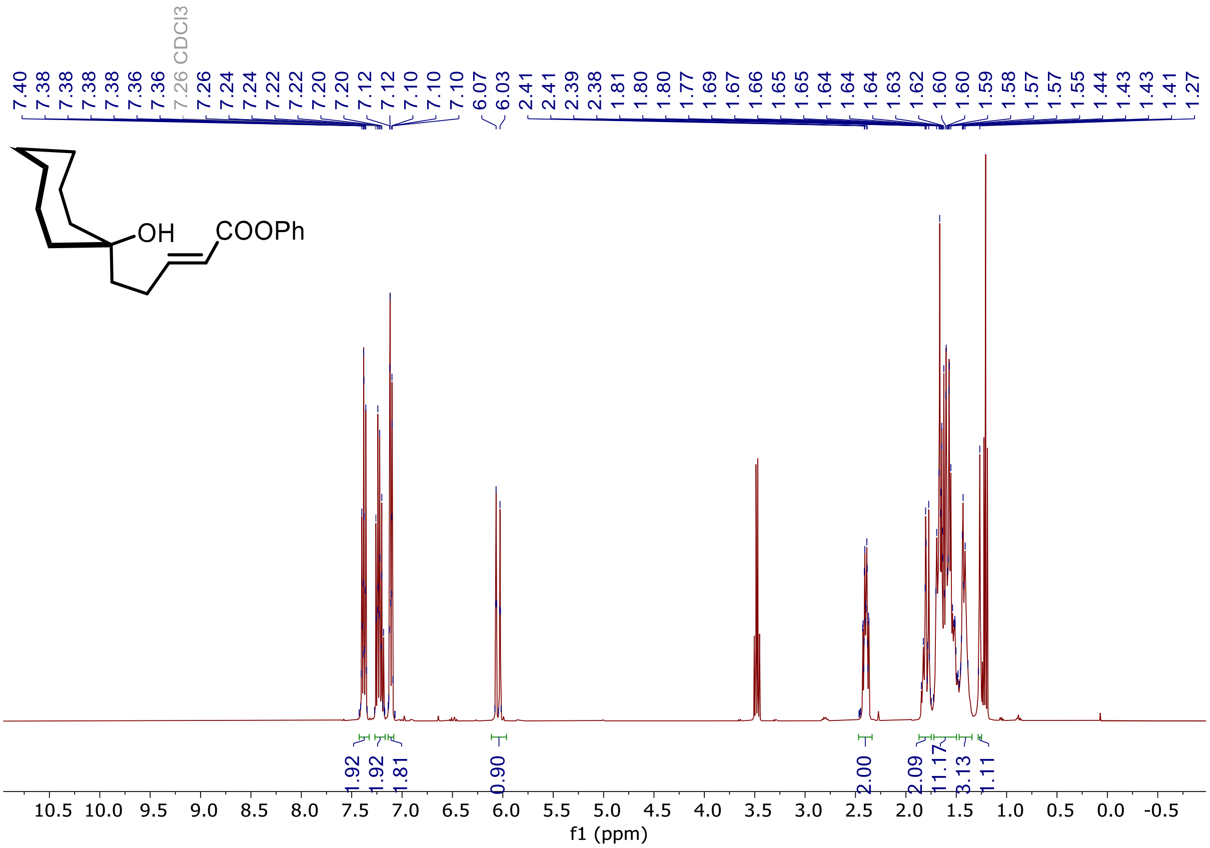

^{13}C NMR: (101 MHz, $CDCl_3$, 298K) of **3s**

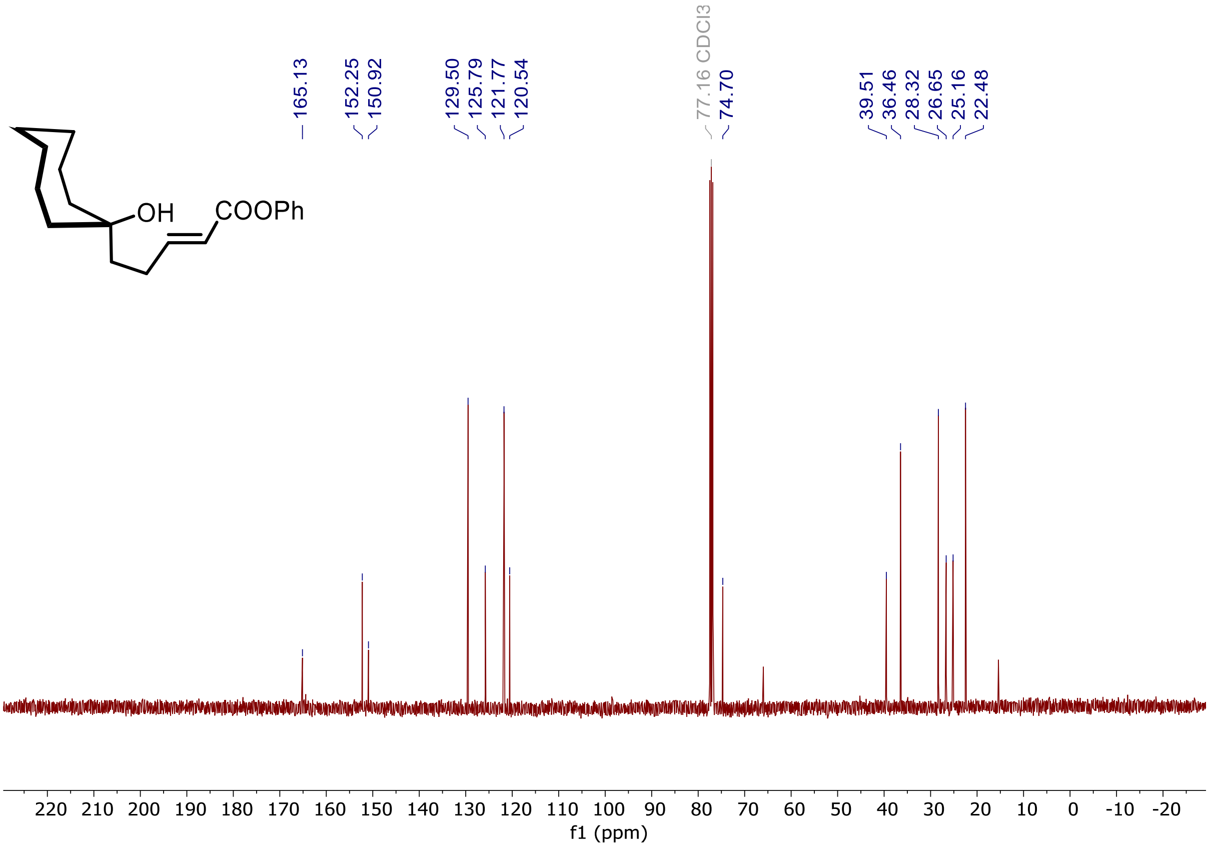

^{1}H NMR: (400 MHz, $CDCl_3$, 298K) of **3t**

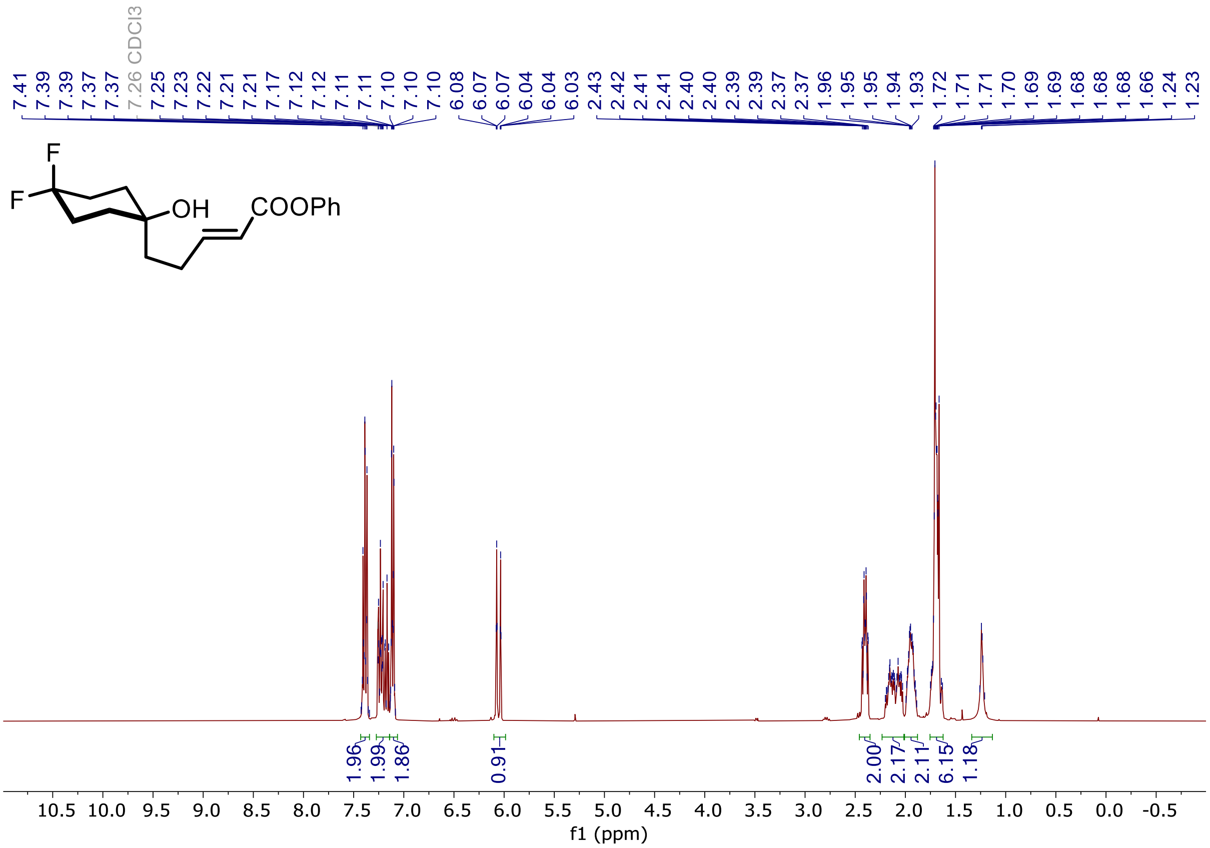

^{13}C NMR: (101 MHz, $CDCl_3$, 298K) of **3t**

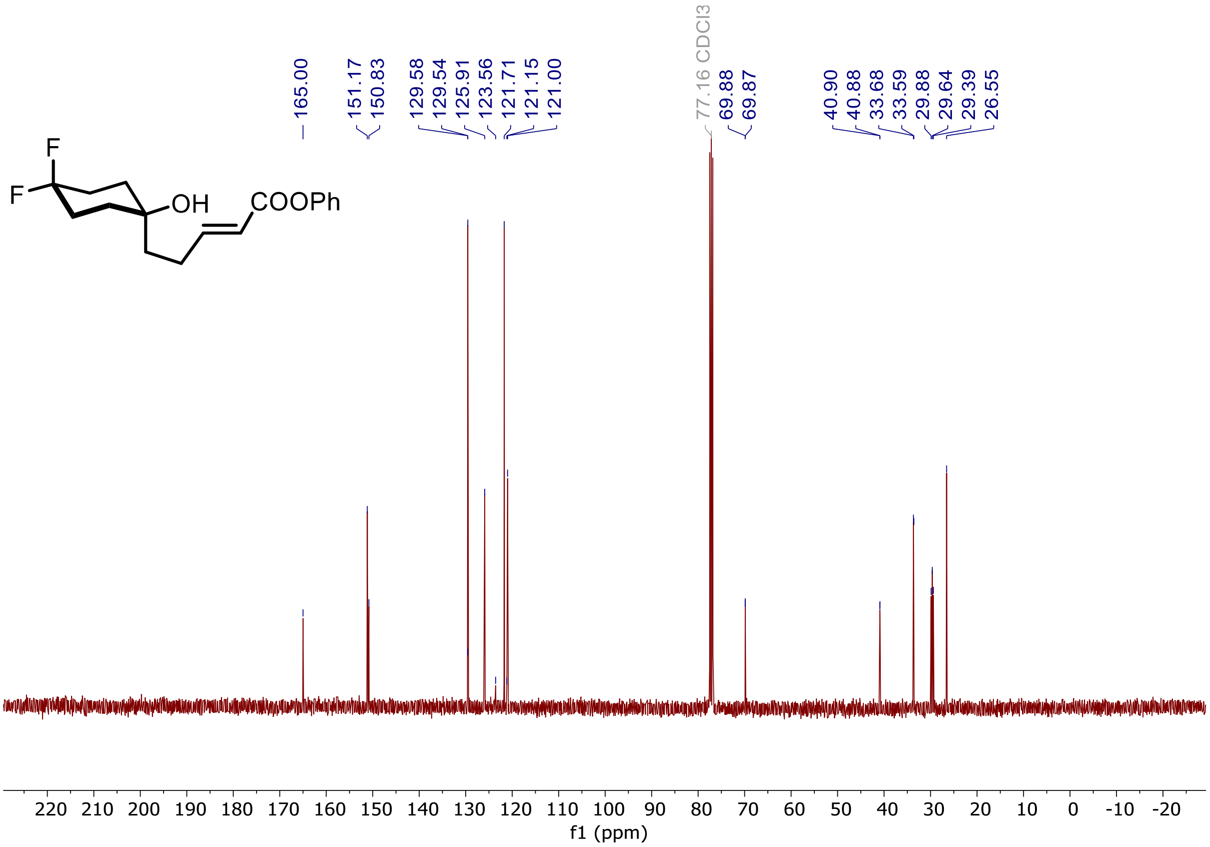

^{19}F NMR: (377 MHz, $CDCl_3$, 298K) of **3t**

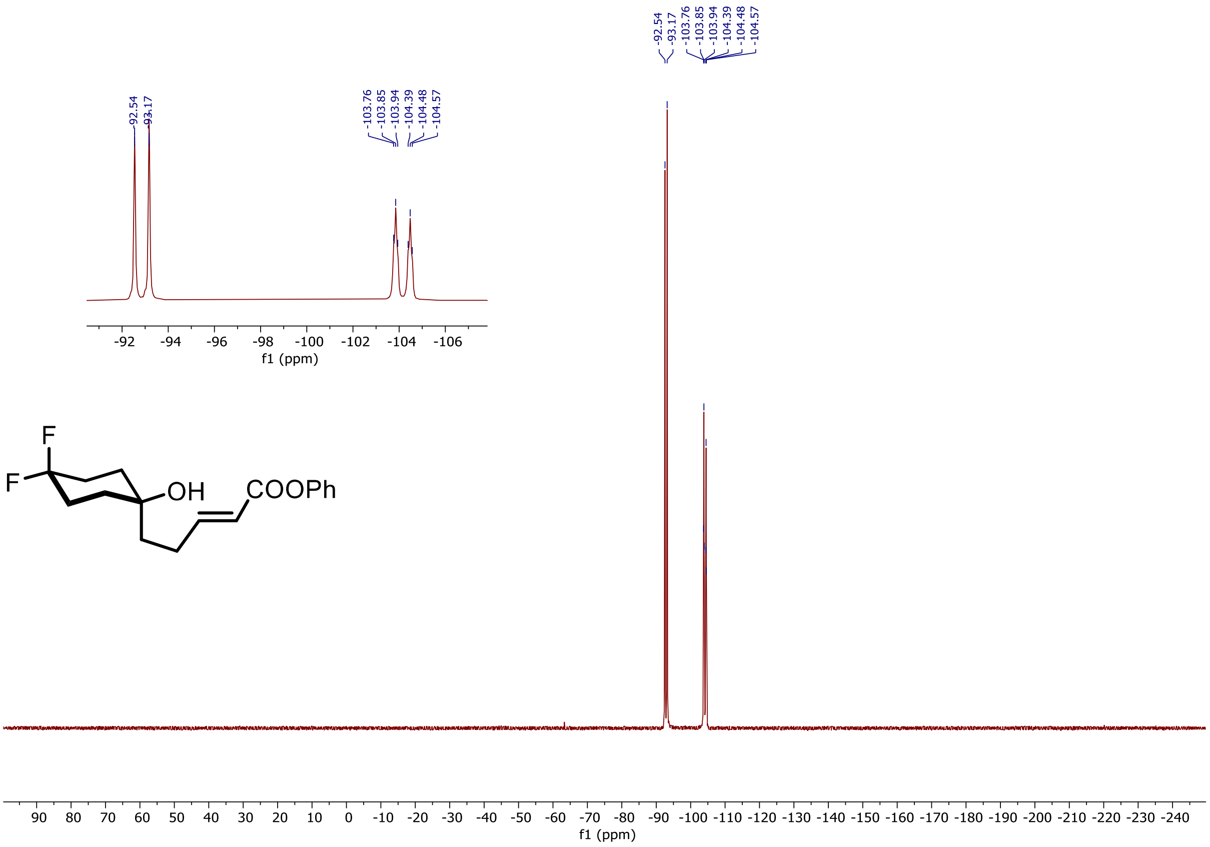

^{1}H NMR: (400 MHz, $CDCl_3$, 298K) of **3u**

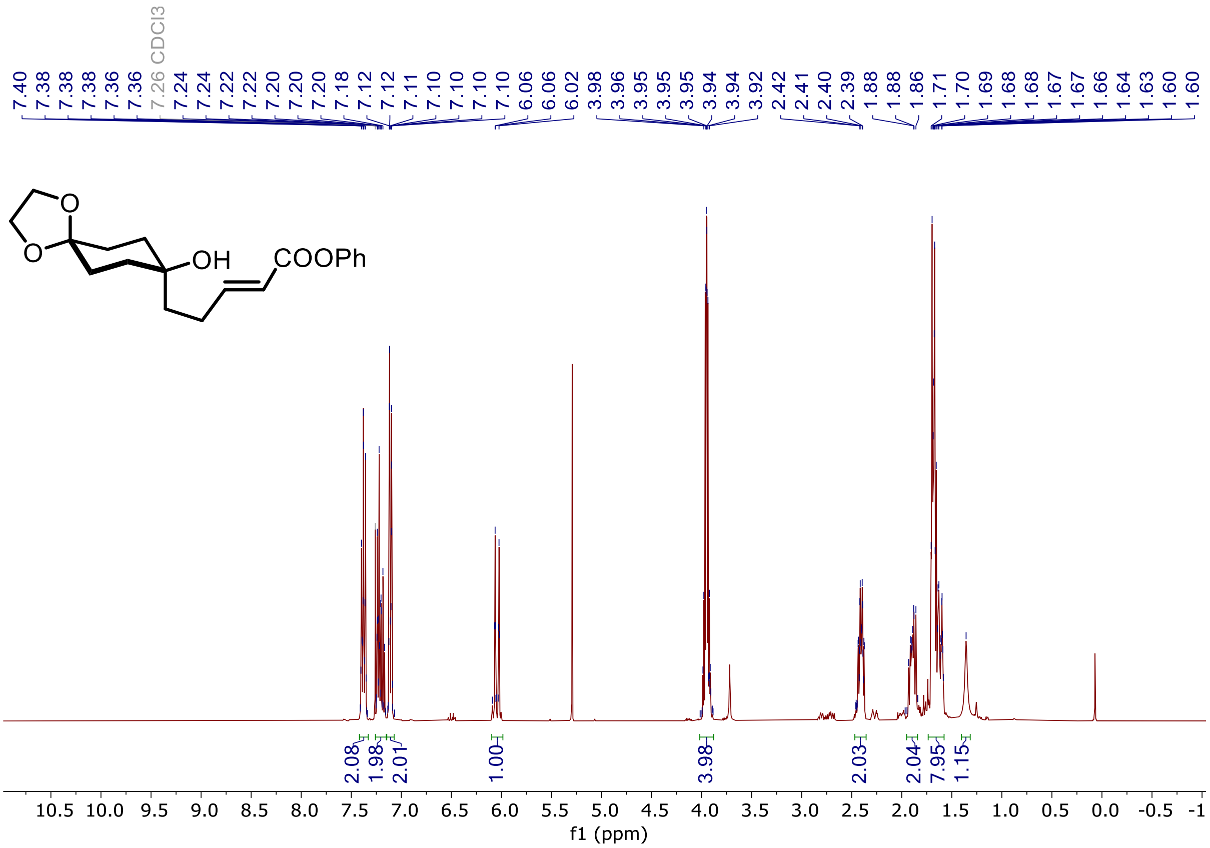

^{13}C NMR: (101 MHz, $CDCl_3$, 298K) of **3u**

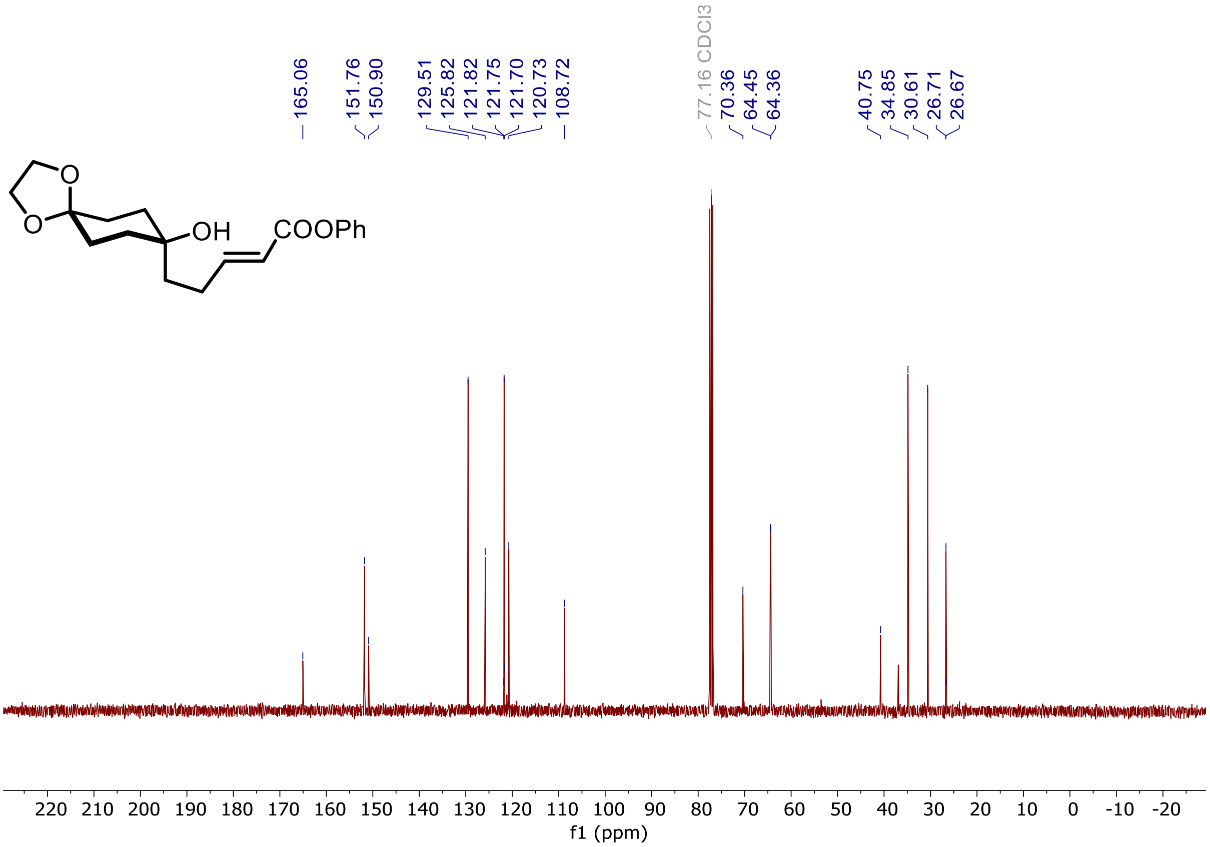

^{1}H NMR: (400 MHz, $CDCl_3$, 298K) of **3v**

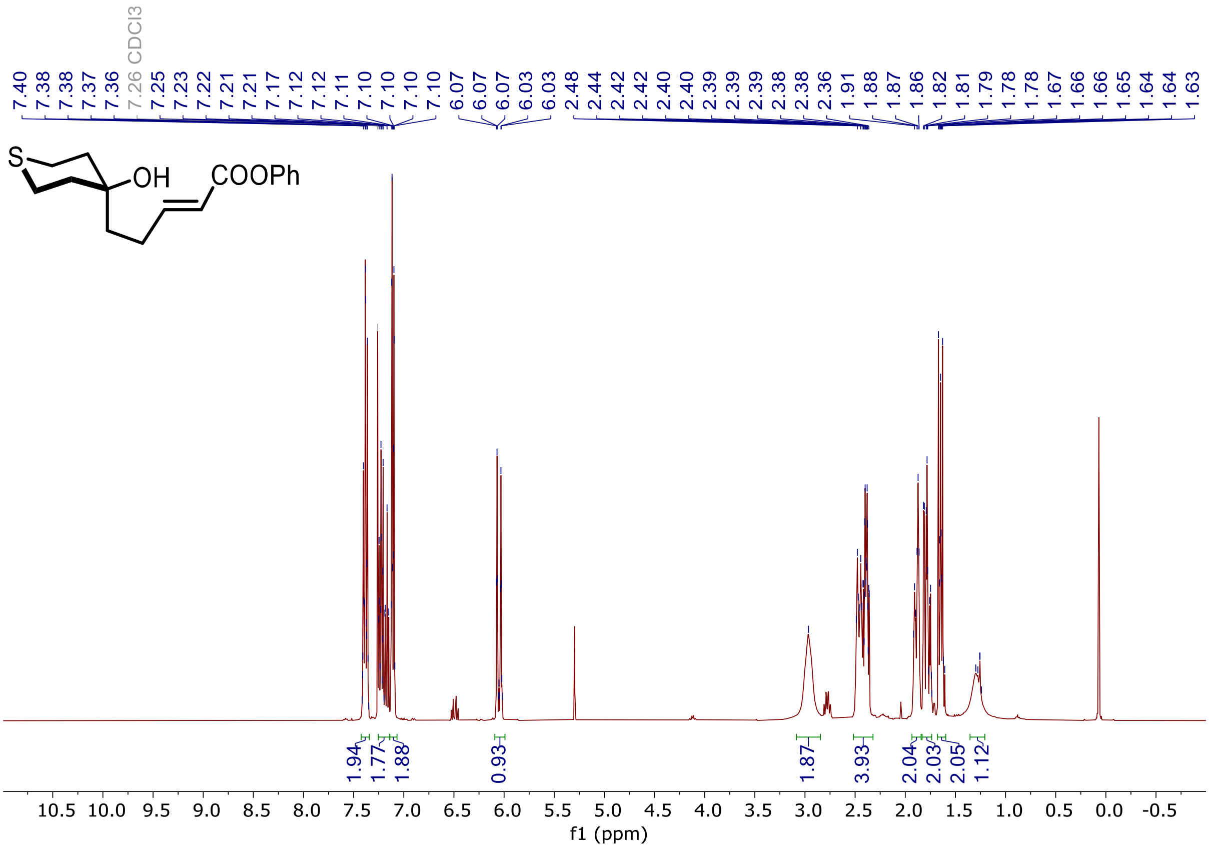

^{13}C NMR: (101 MHz, $CDCl_3$, 298K) of **3v**

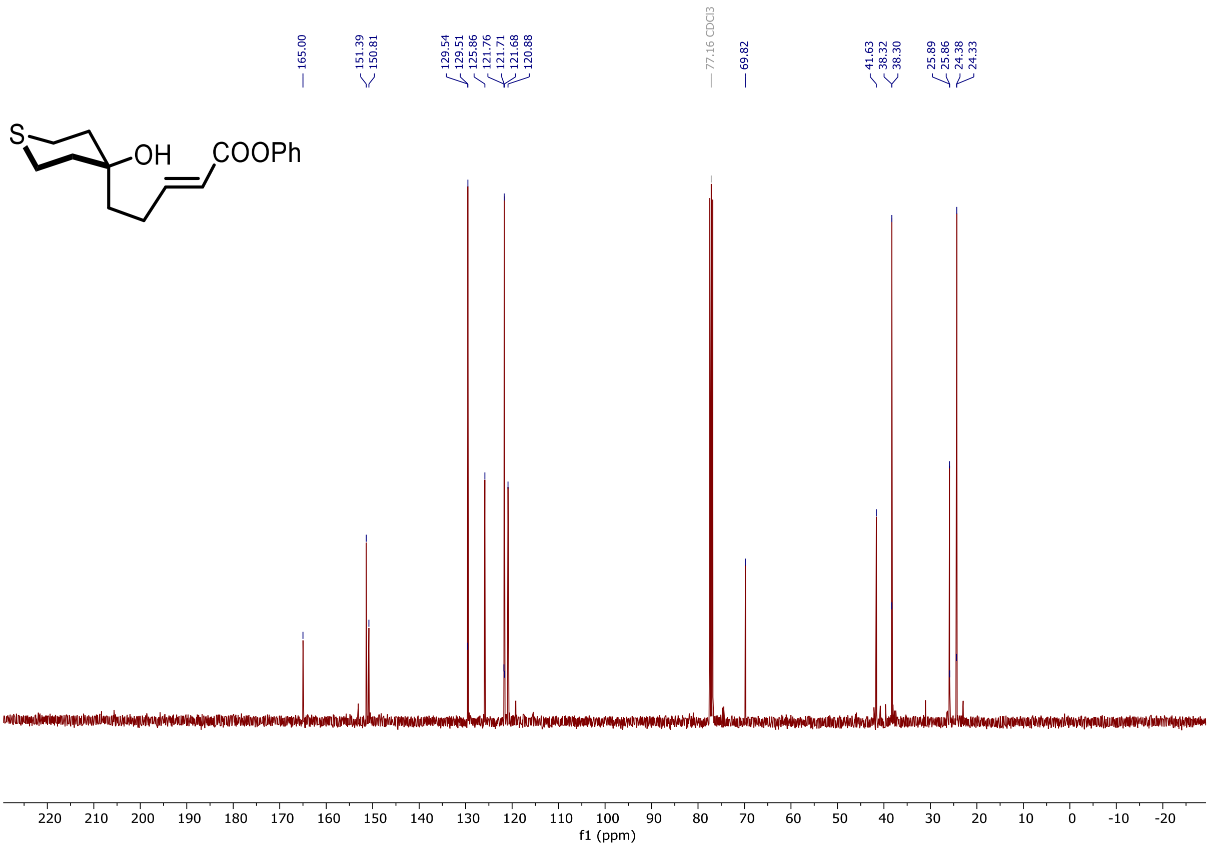

^{1}H NMR: (400 MHz, $CDCl_3$, 298K) of **3w**

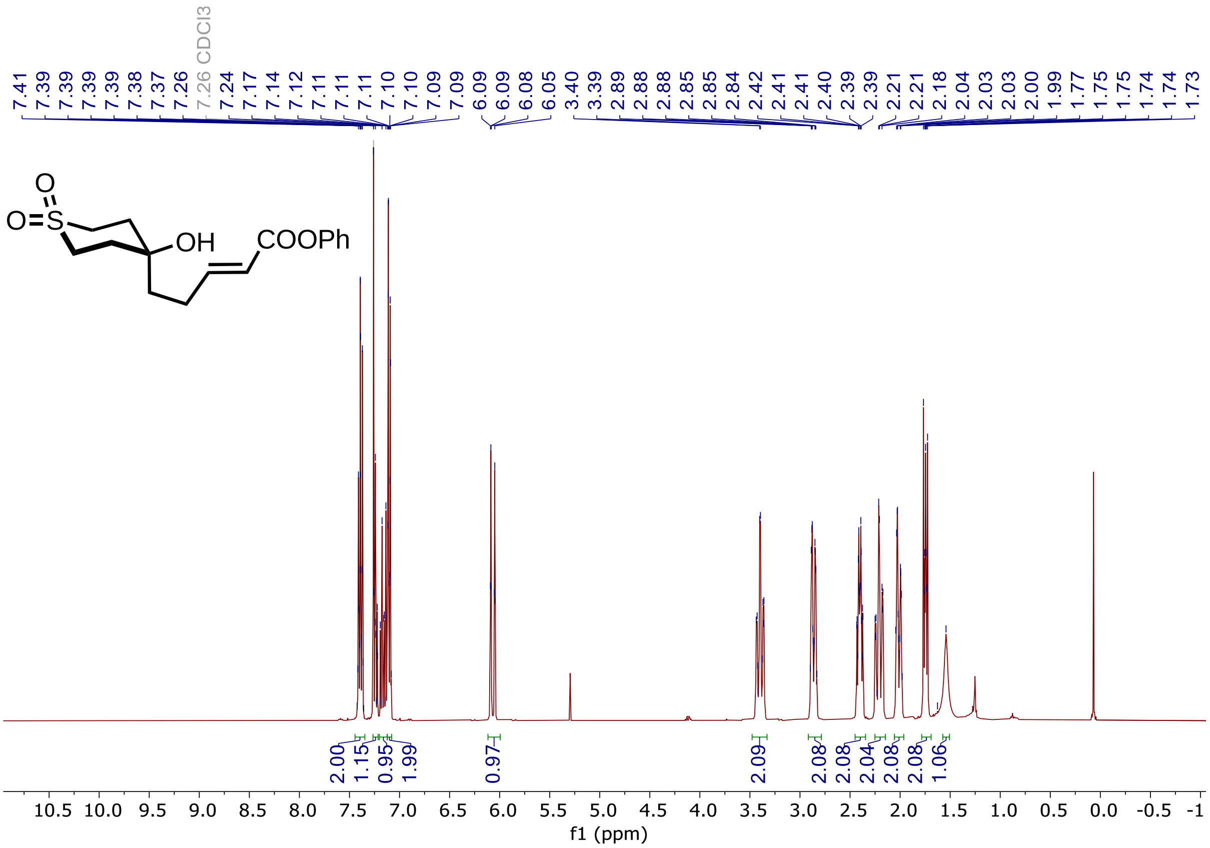

^{13}C NMR: (101 MHz, $CDCl_3$, 298K) of **3w**

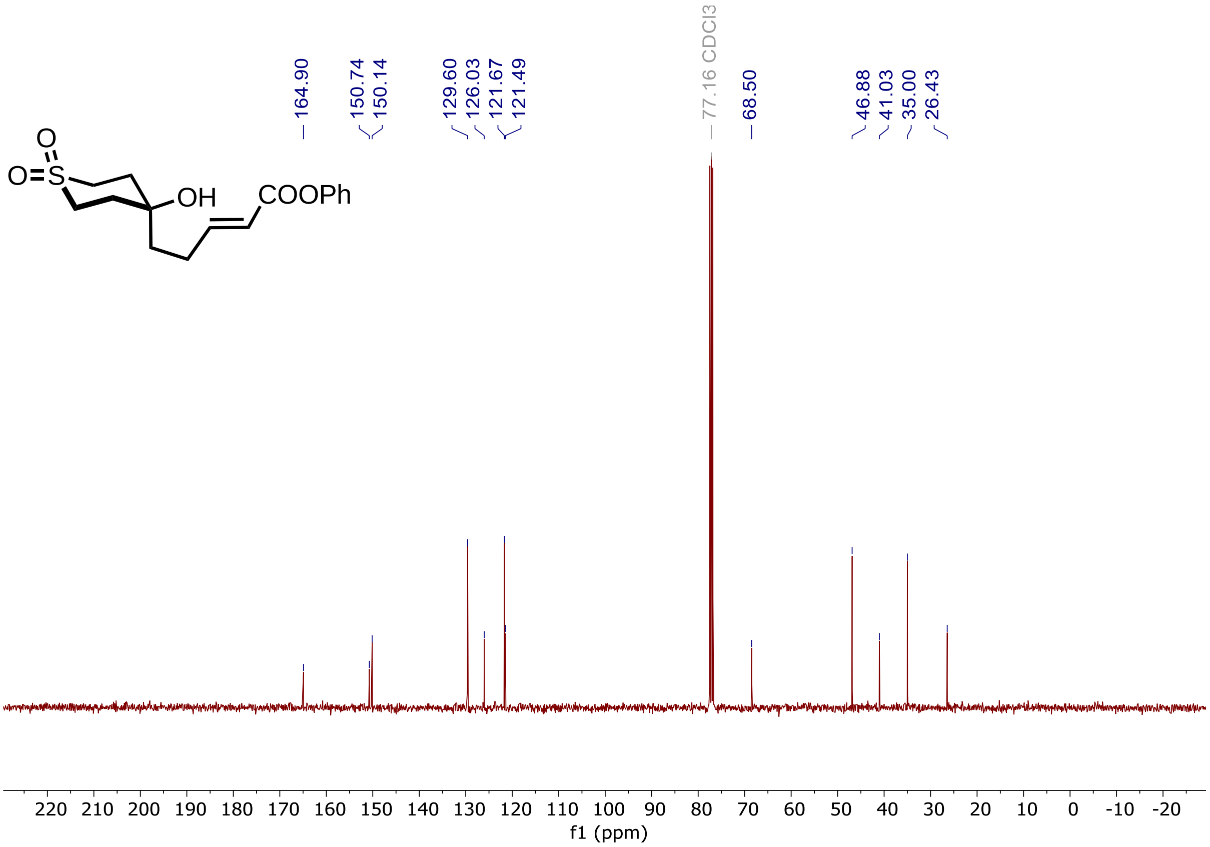

^{1}H NMR: (400 MHz, $CDCl_3$, 298K) of **3x**

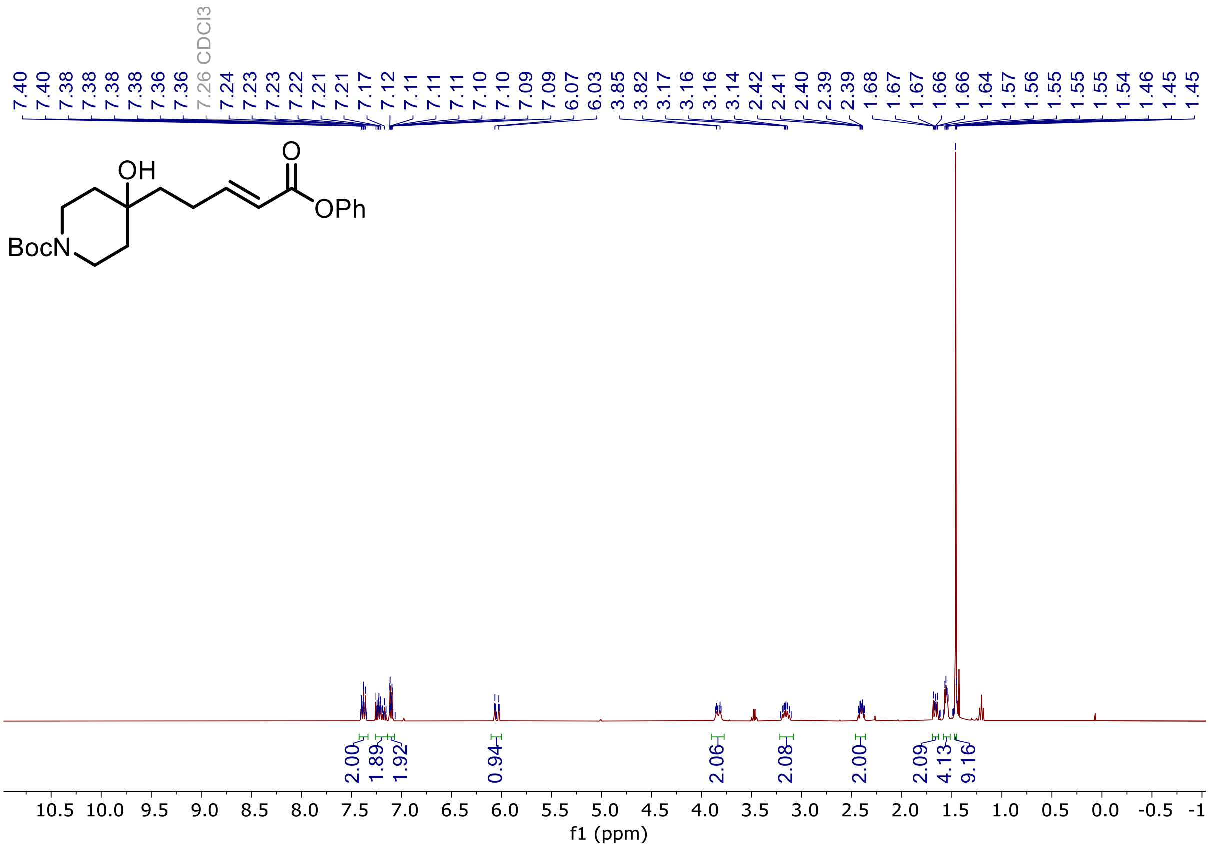

^{13}C NMR: (101 MHz, $CDCl_3$, 298K) of **3x**

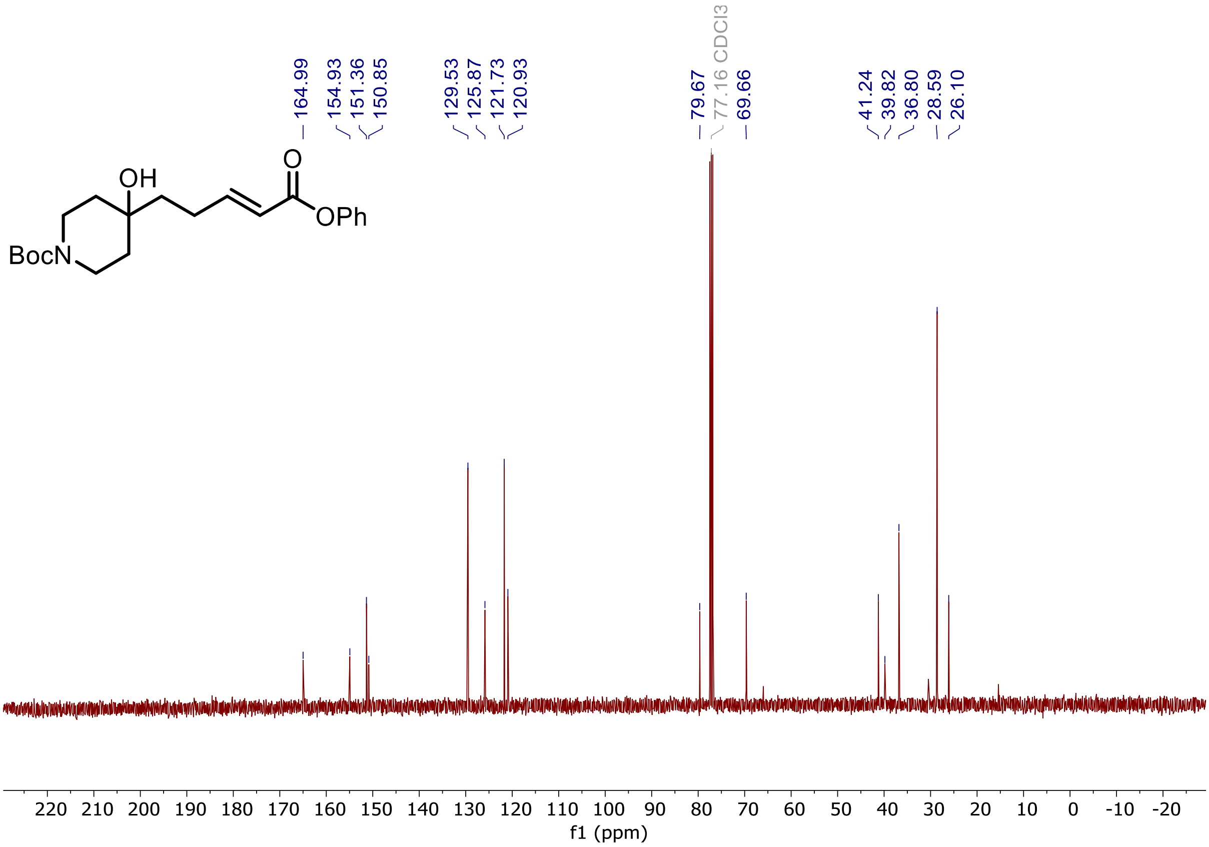

^{1}H NMR: (400 MHz, $CDCl_3$, 298K) of **3y**

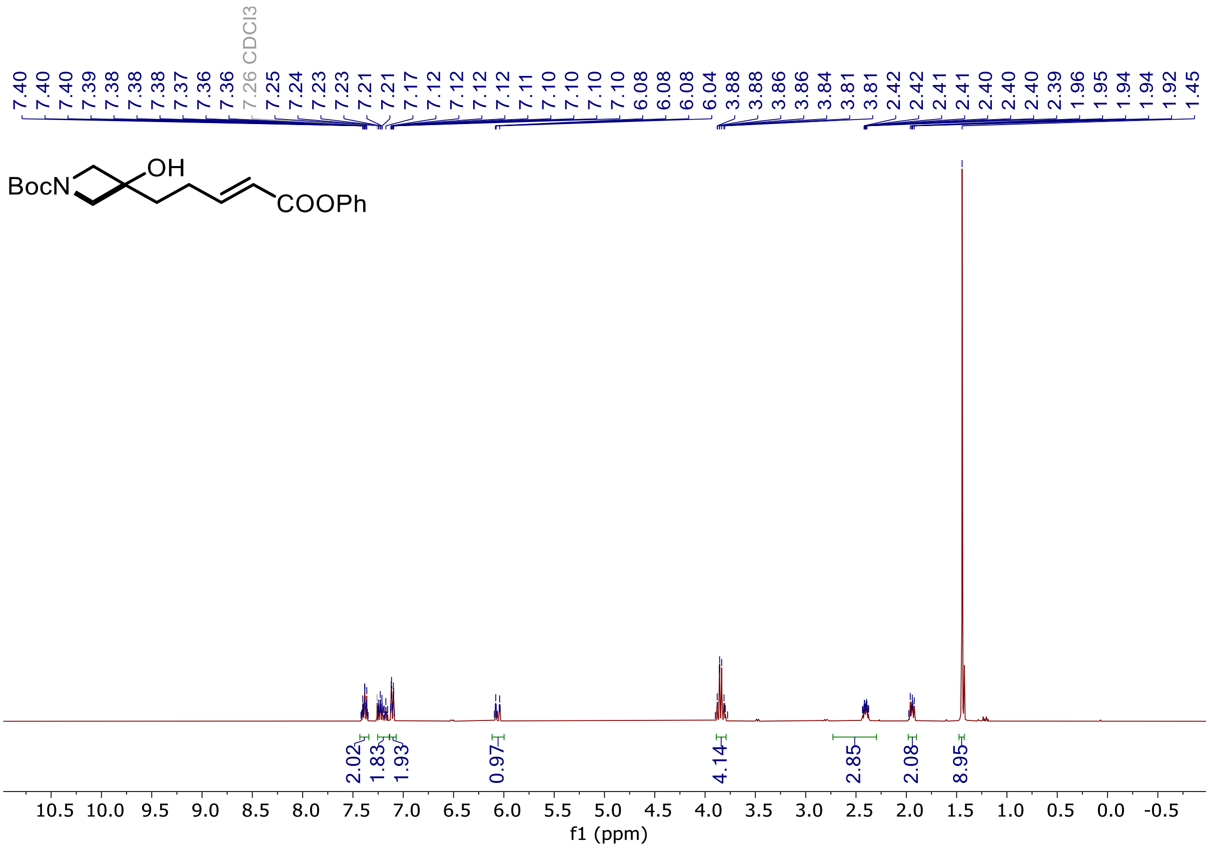

^{13}C NMR: (101 MHz, $CDCl_3$, 298K) of **3y**

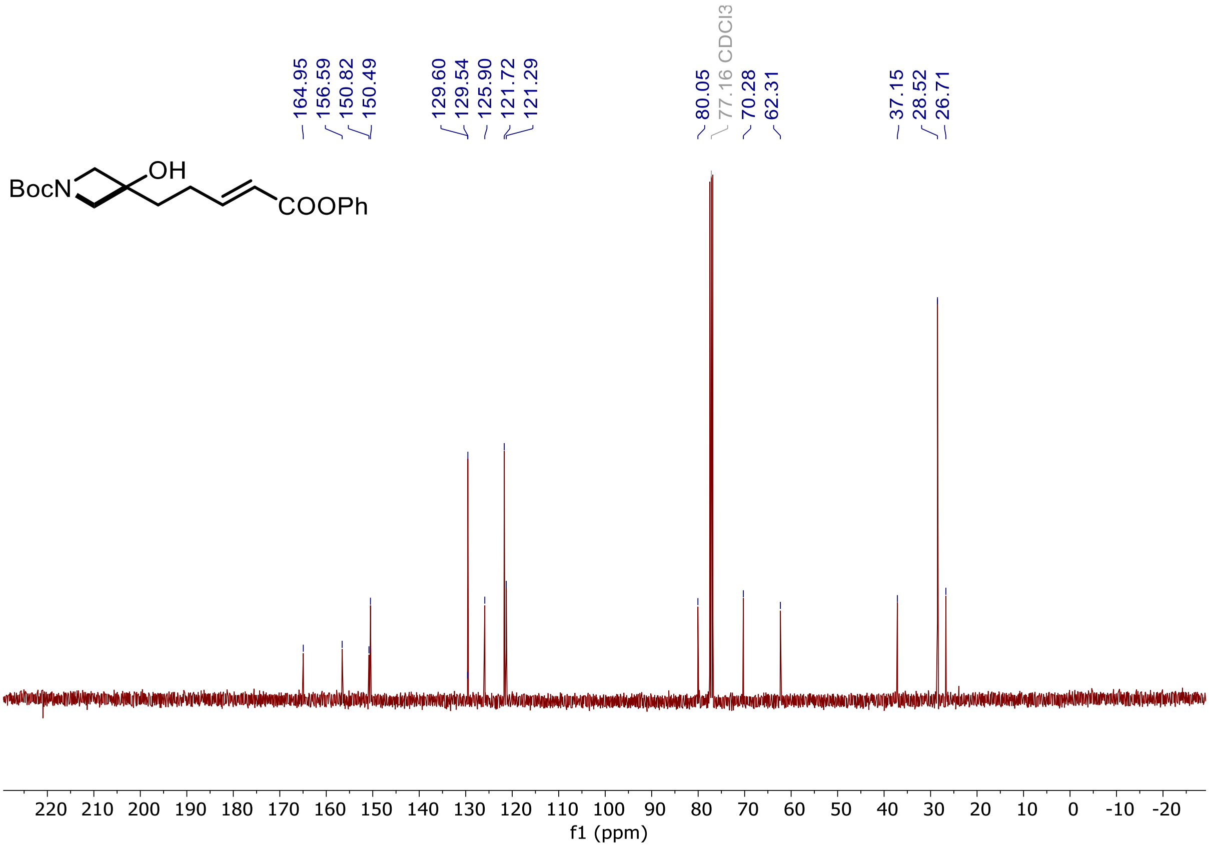

^{1}H NMR: (400 MHz, $CDCl_3$, 298K) of **3z**

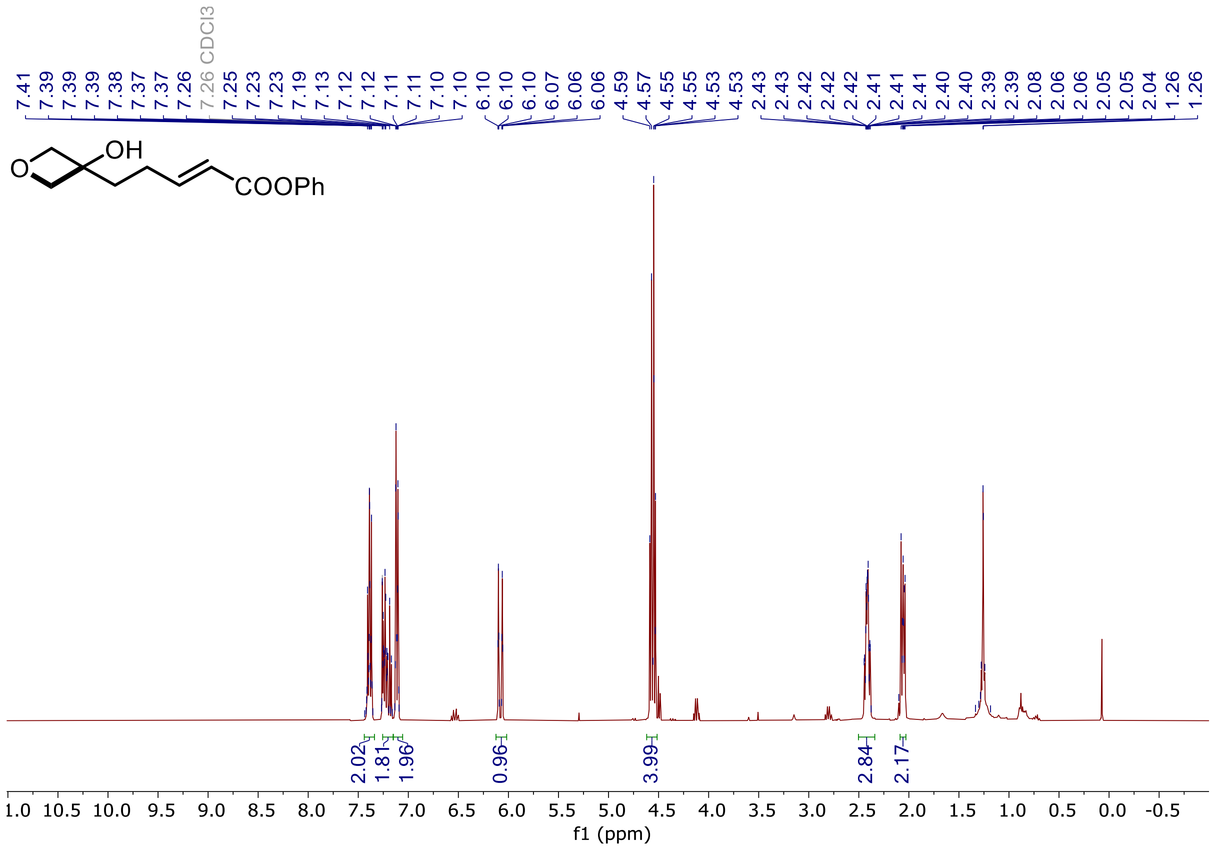

^{13}C NMR: (101 MHz, $CDCl_3$, 298K) of **3z**

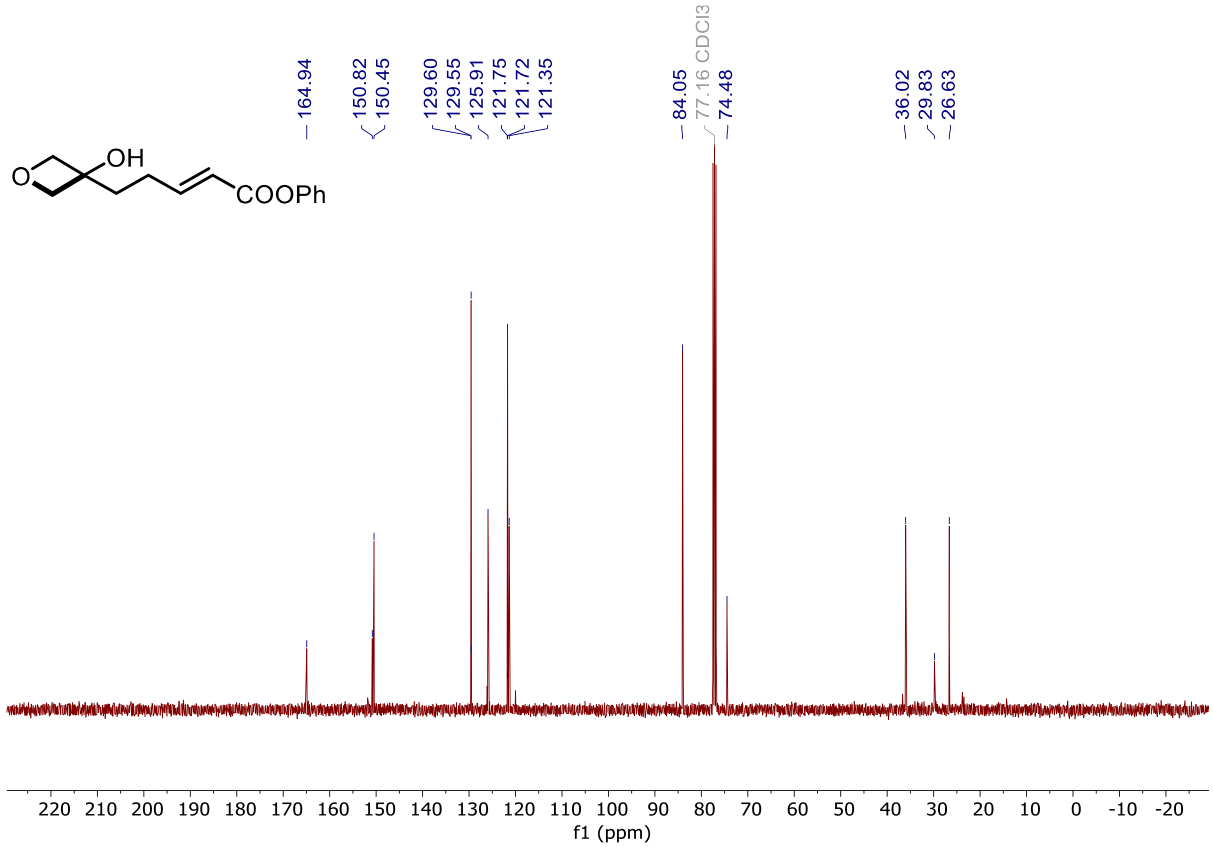

^{1}H NMR: (400 MHz, $CDCl_3$, 298K) of **3aa**

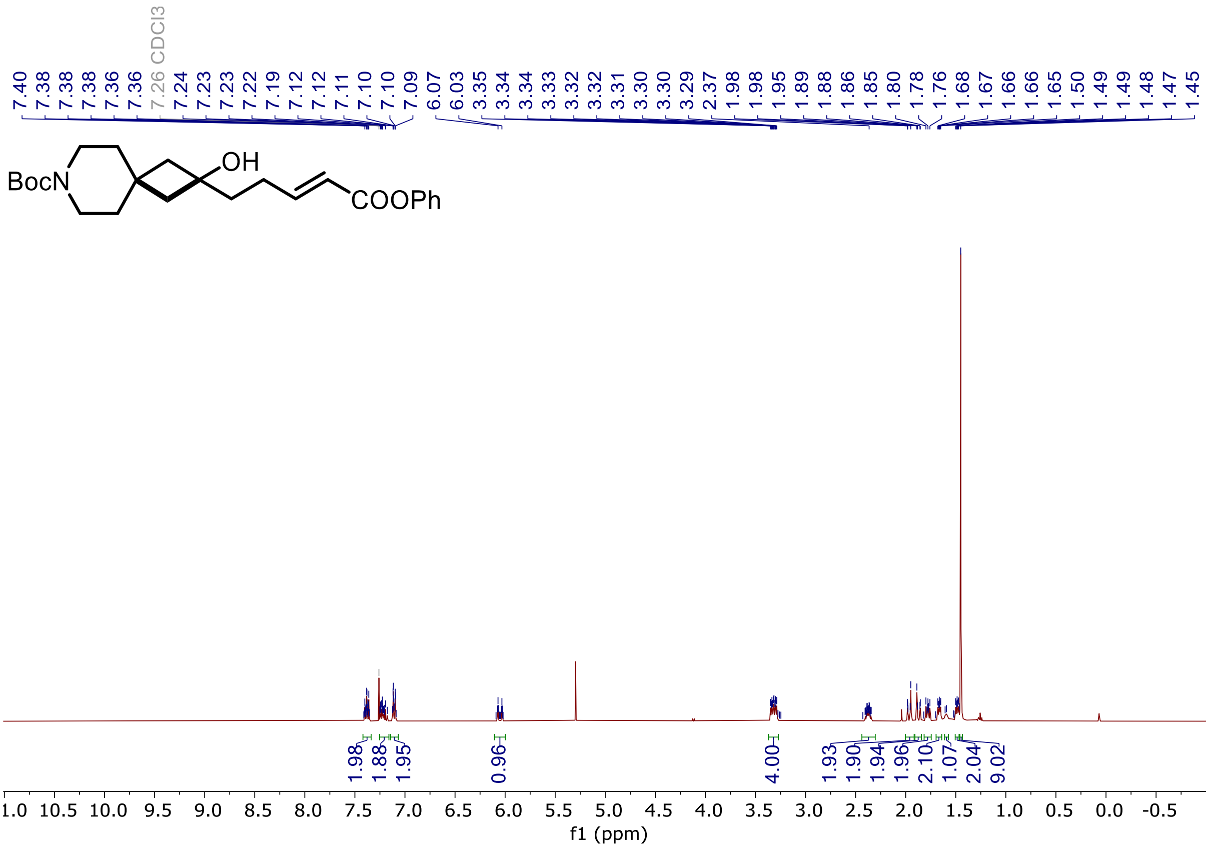

^{13}C NMR: (101 MHz, $CDCl_3$, 298K) of **3aa**

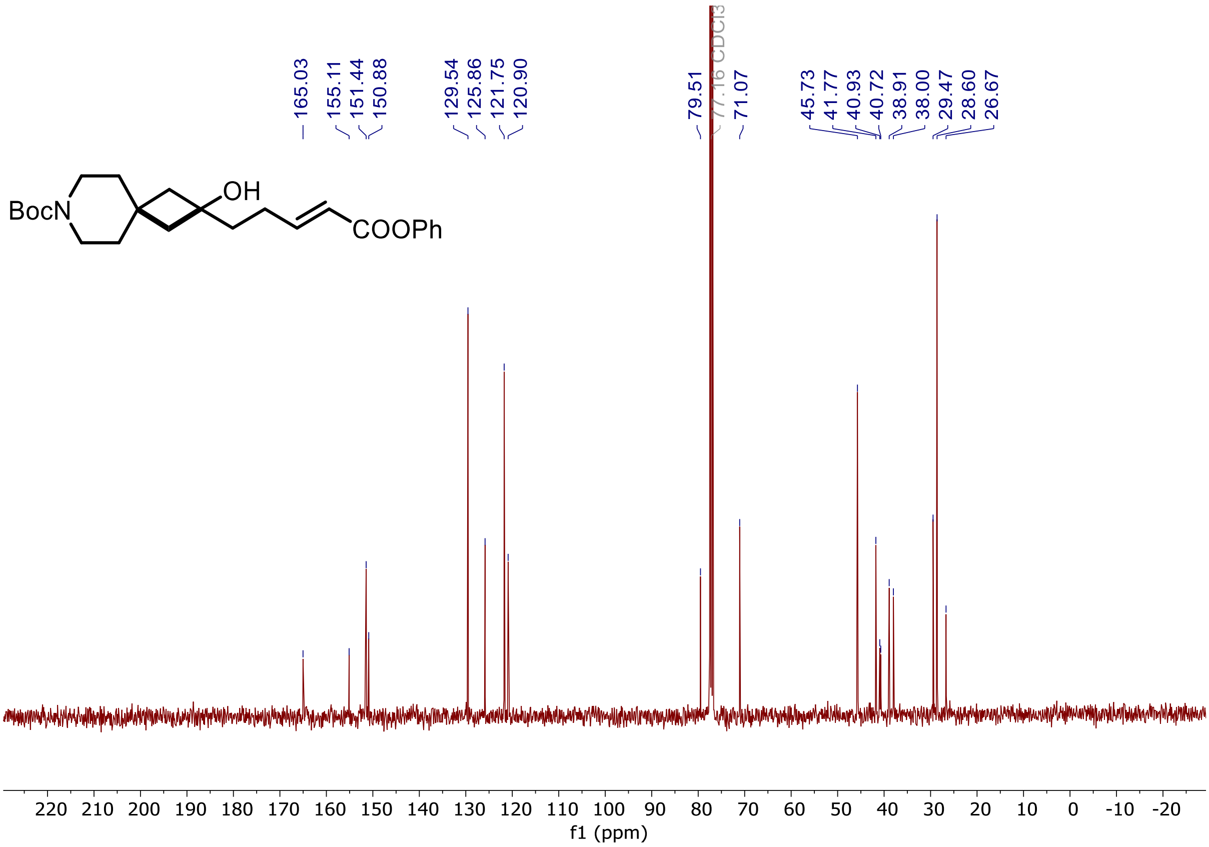

^{1}H NMR: (400 MHz, $CDCl_3$, 298K) of **3ab**

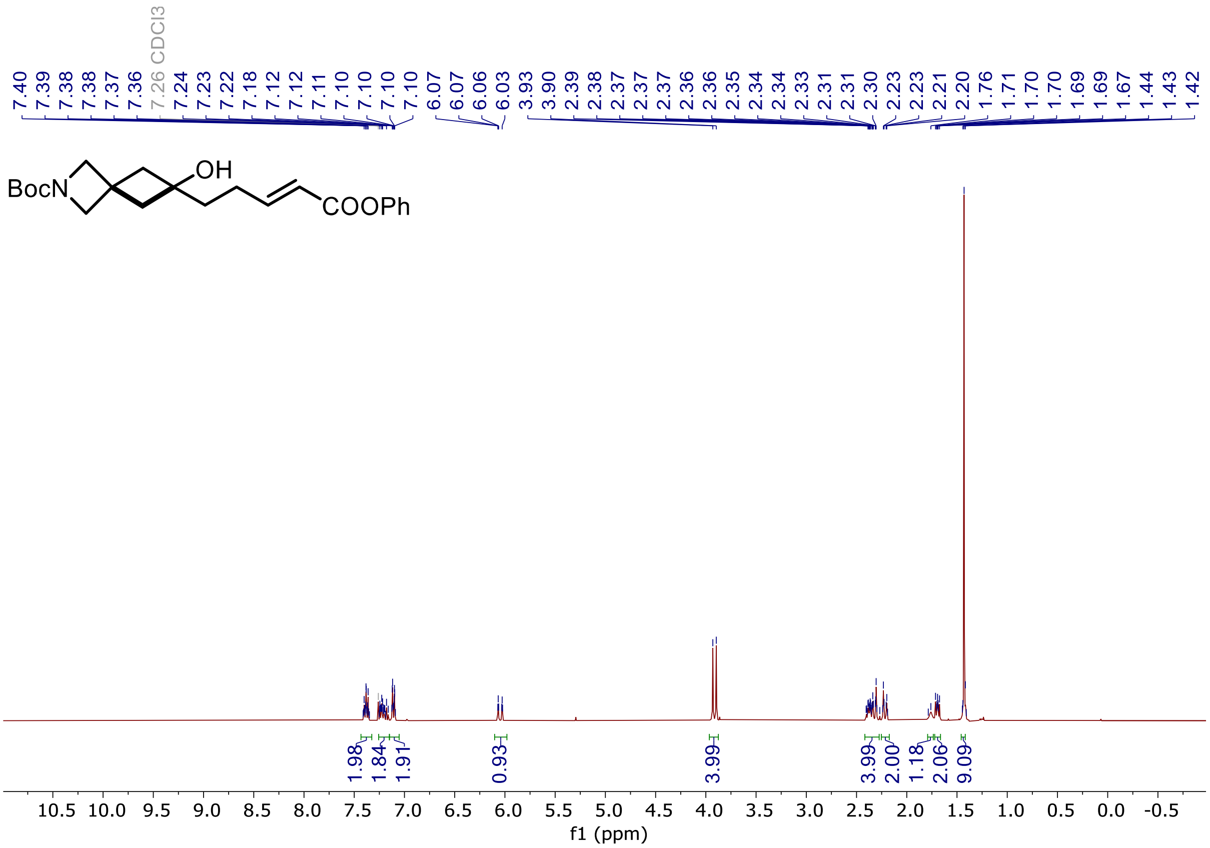

^{13}C NMR: (101 MHz, $CDCl_3$, 298K) of **3ab**

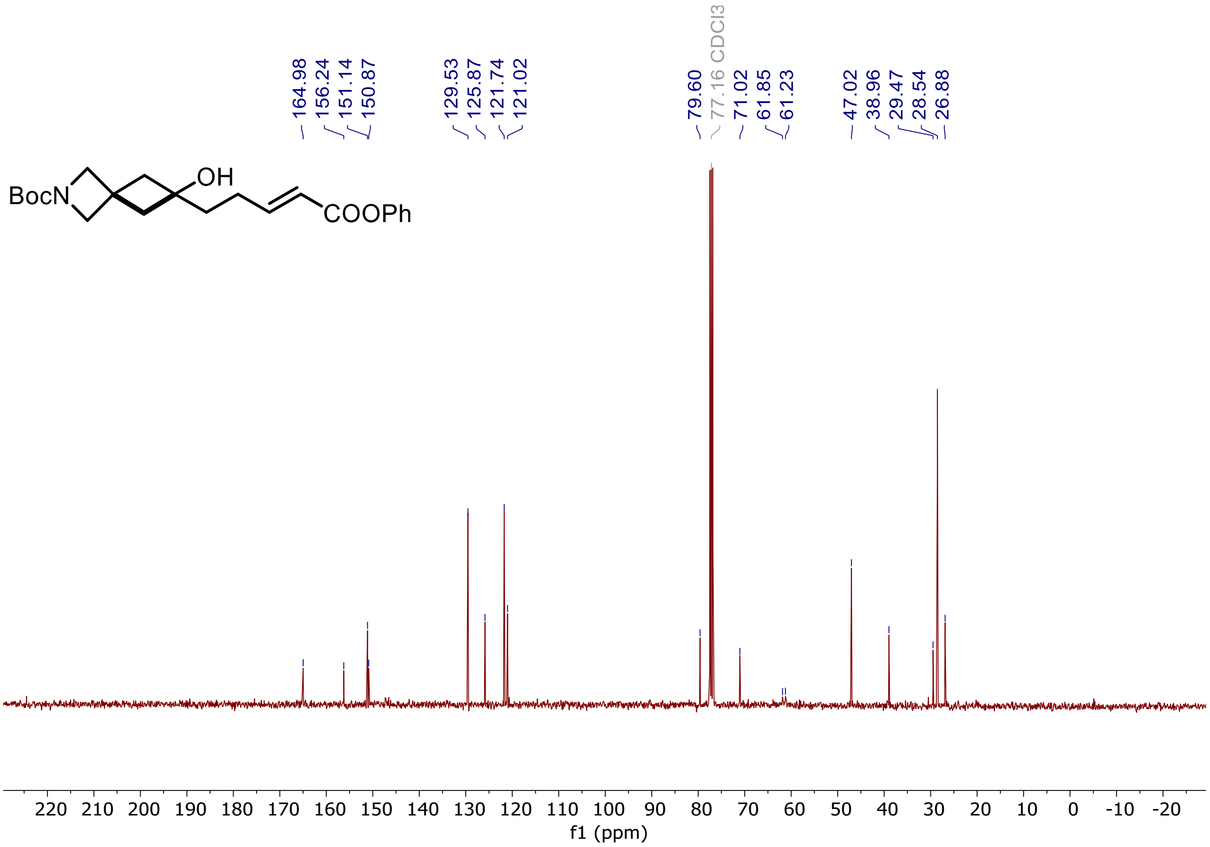

^{1}H NMR: (400 MHz, CDCl$_3$, 298K) of **3ac**

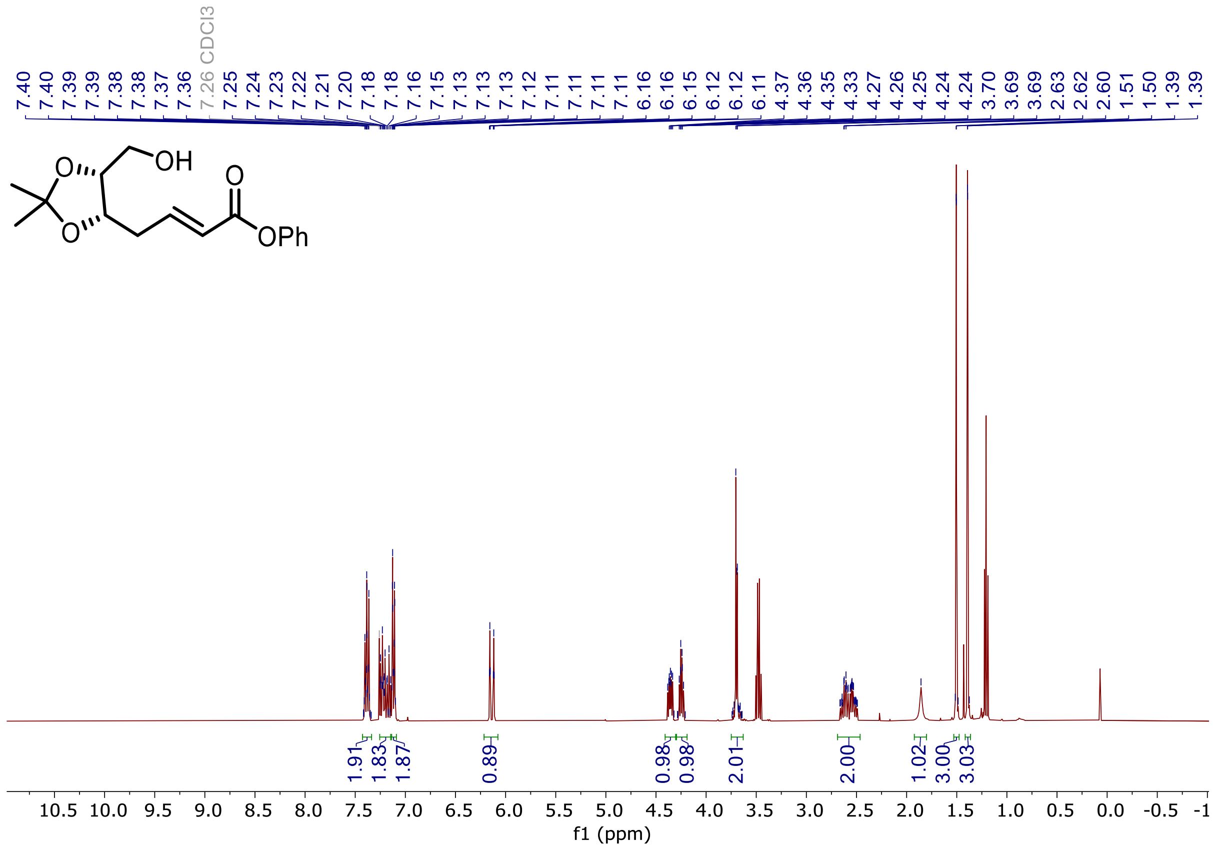

^{13}C NMR: (101 MHz, CDCl$_3$, 298K) of **3ac**

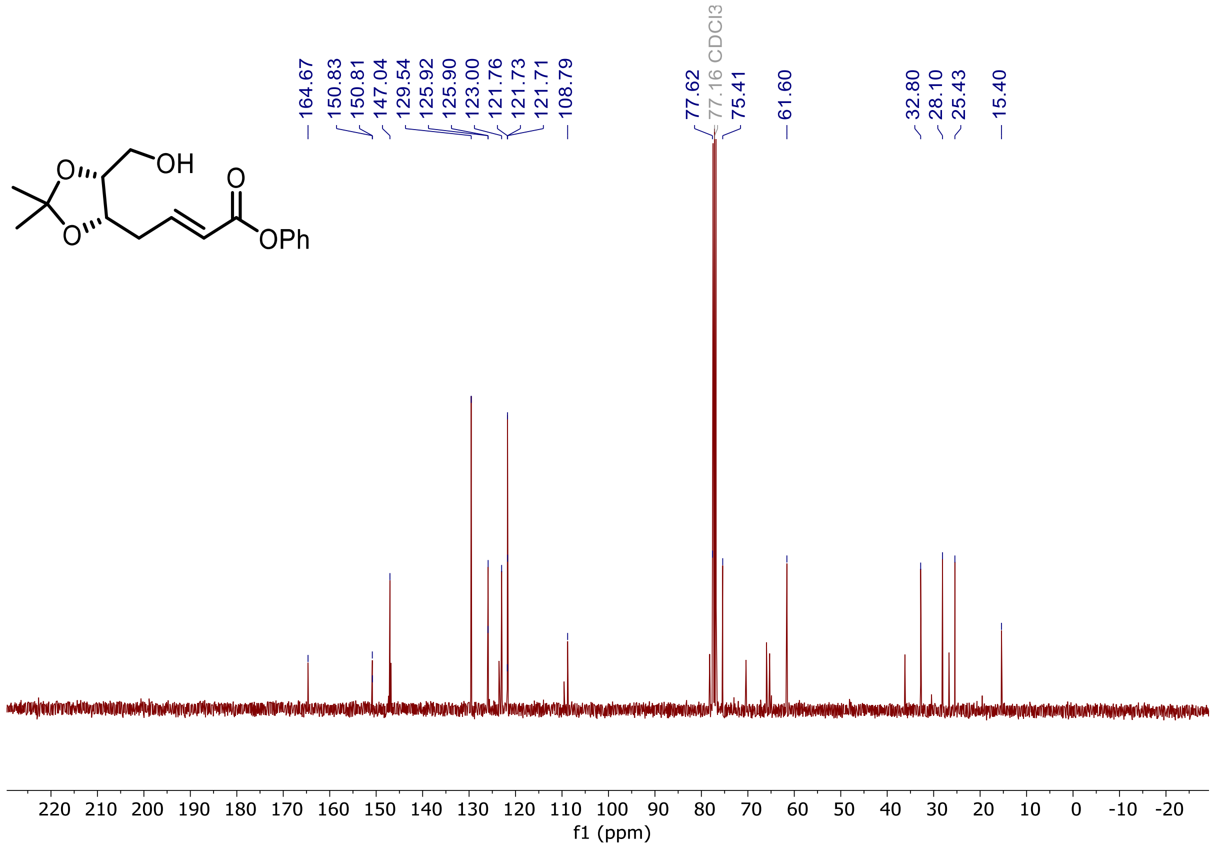

^{1}H NMR: (400 MHz, $CDCl_3$, 298K) of **3ad**

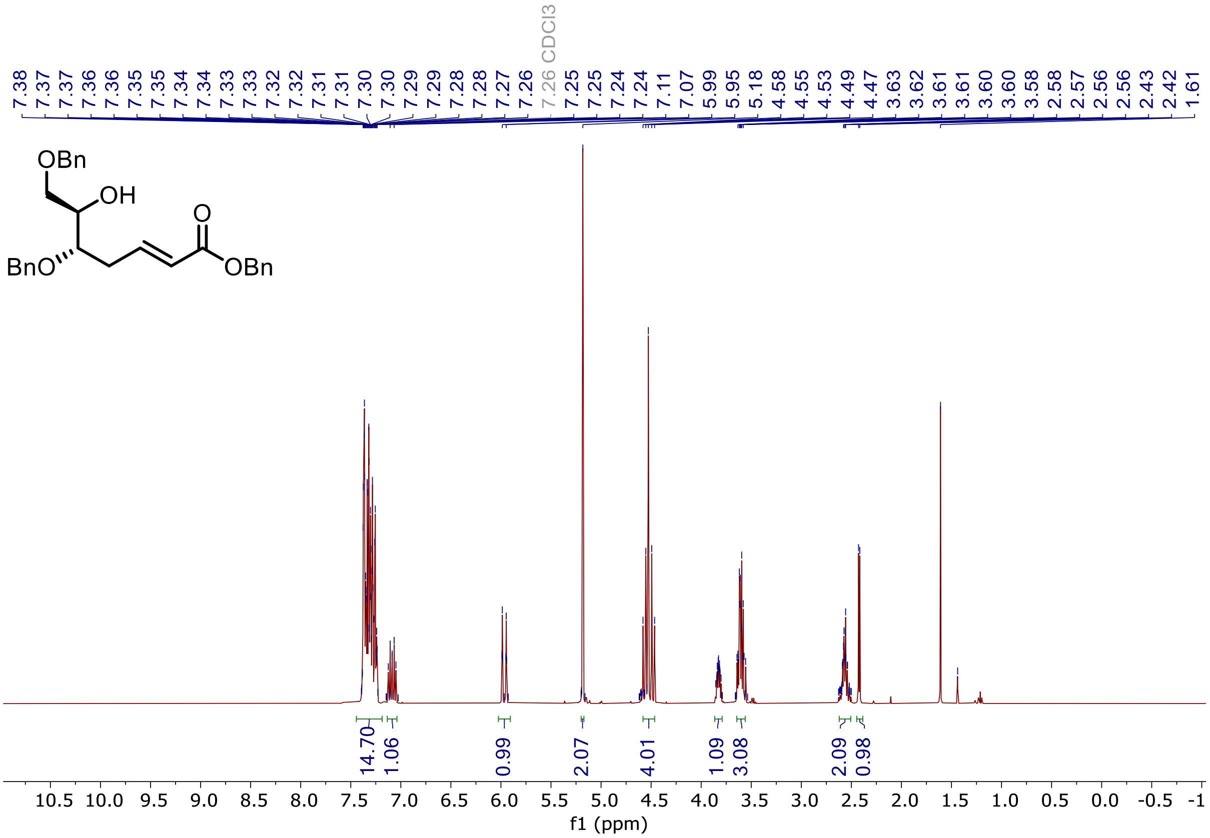

^{13}C NMR: (101 MHz, $CDCl_3$, 298K) of **3ad**

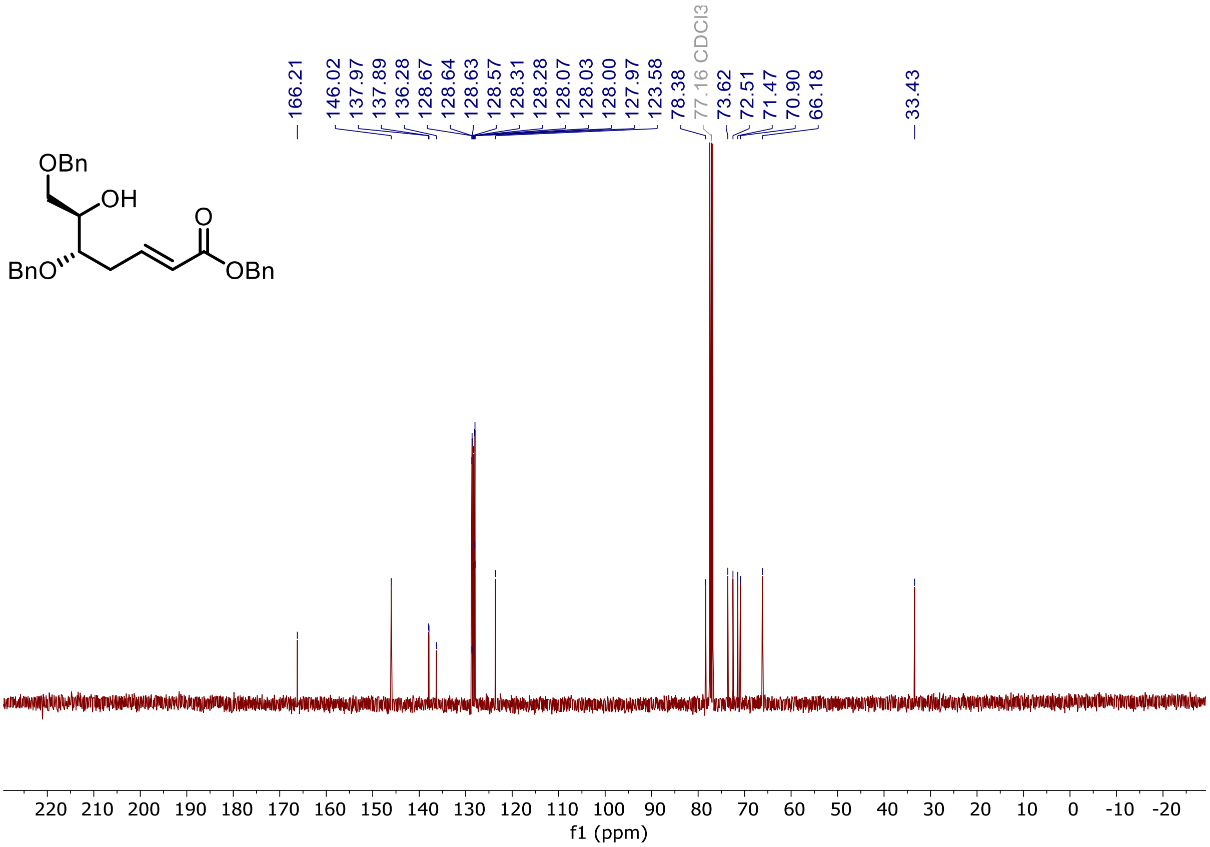

^{1}H NMR: (400 MHz, $CDCl_3$, 298K) of **3ae**

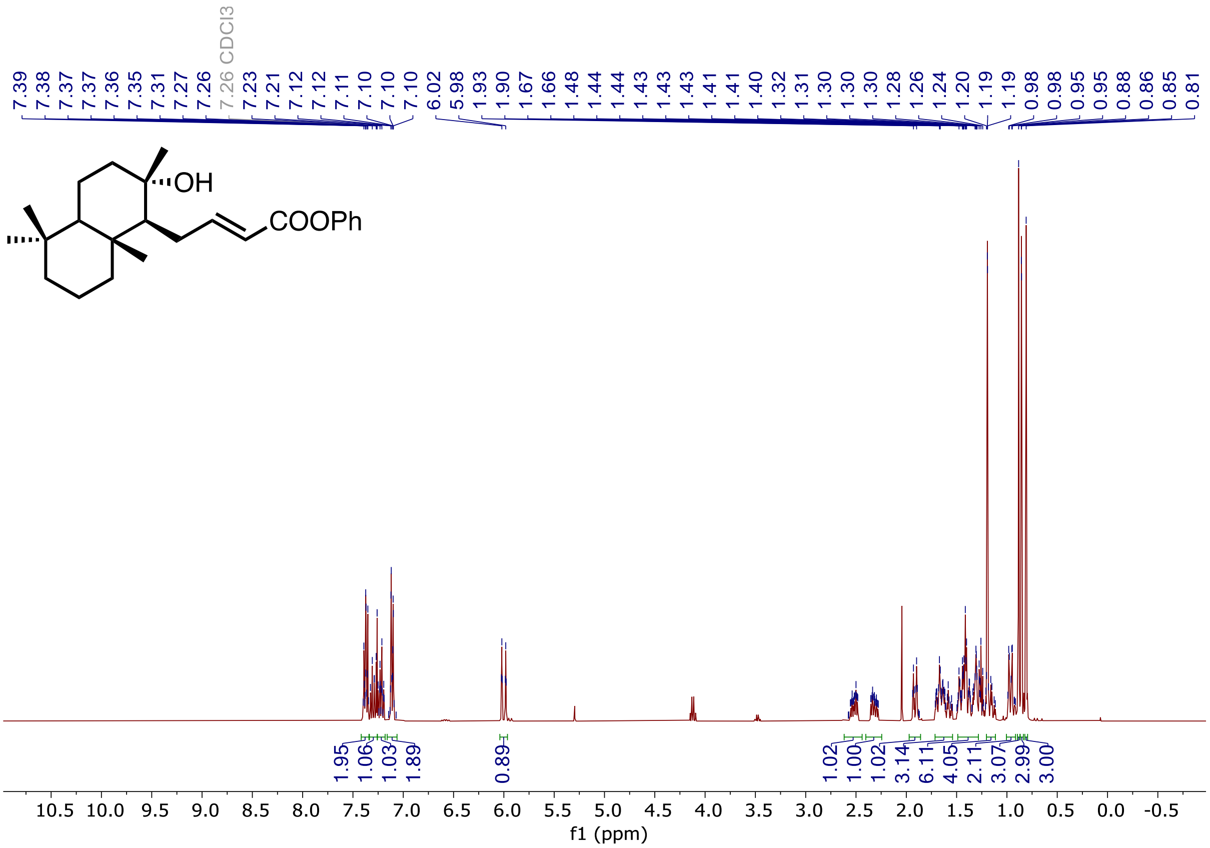

^{13}C NMR: (101 MHz, $CDCl_3$, 298K) of **3ae**

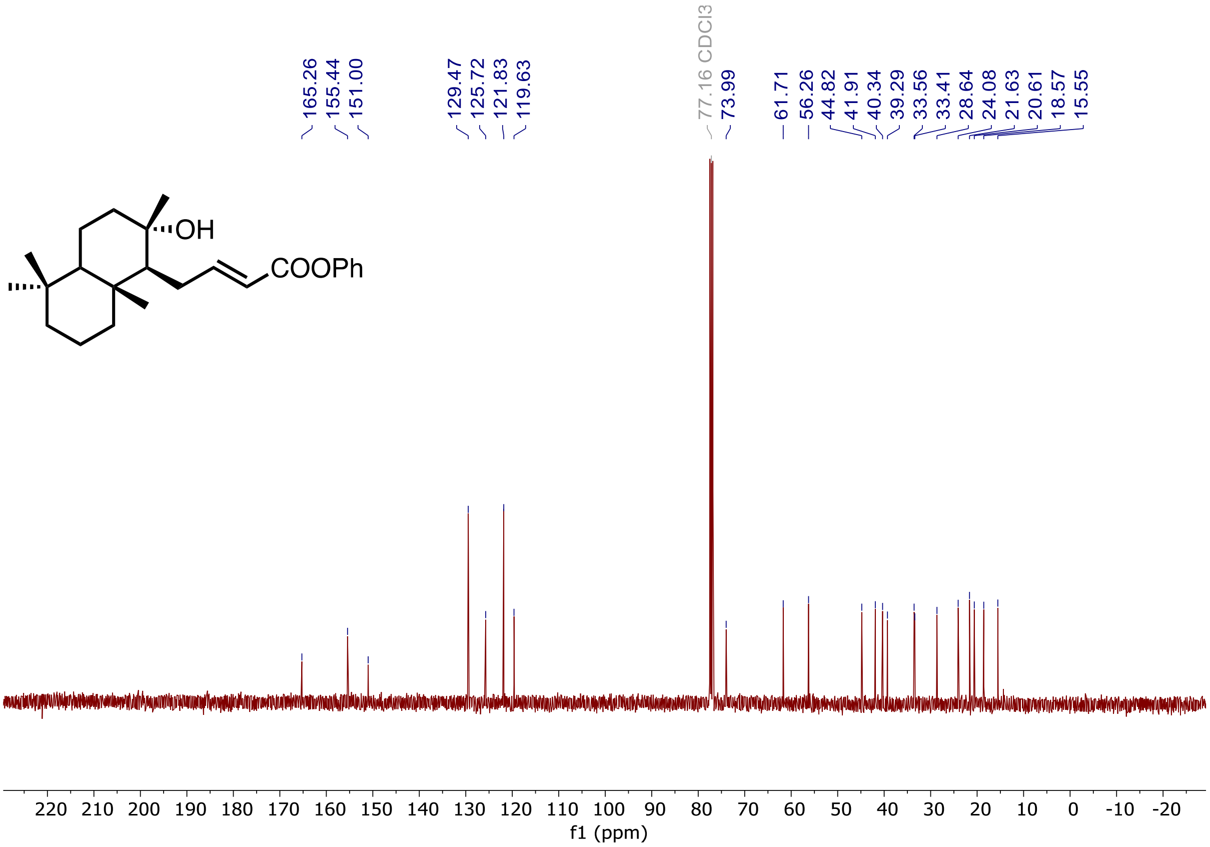

^{1}H NMR: (400 MHz, $CDCl_3$, 298K) of **3ag**

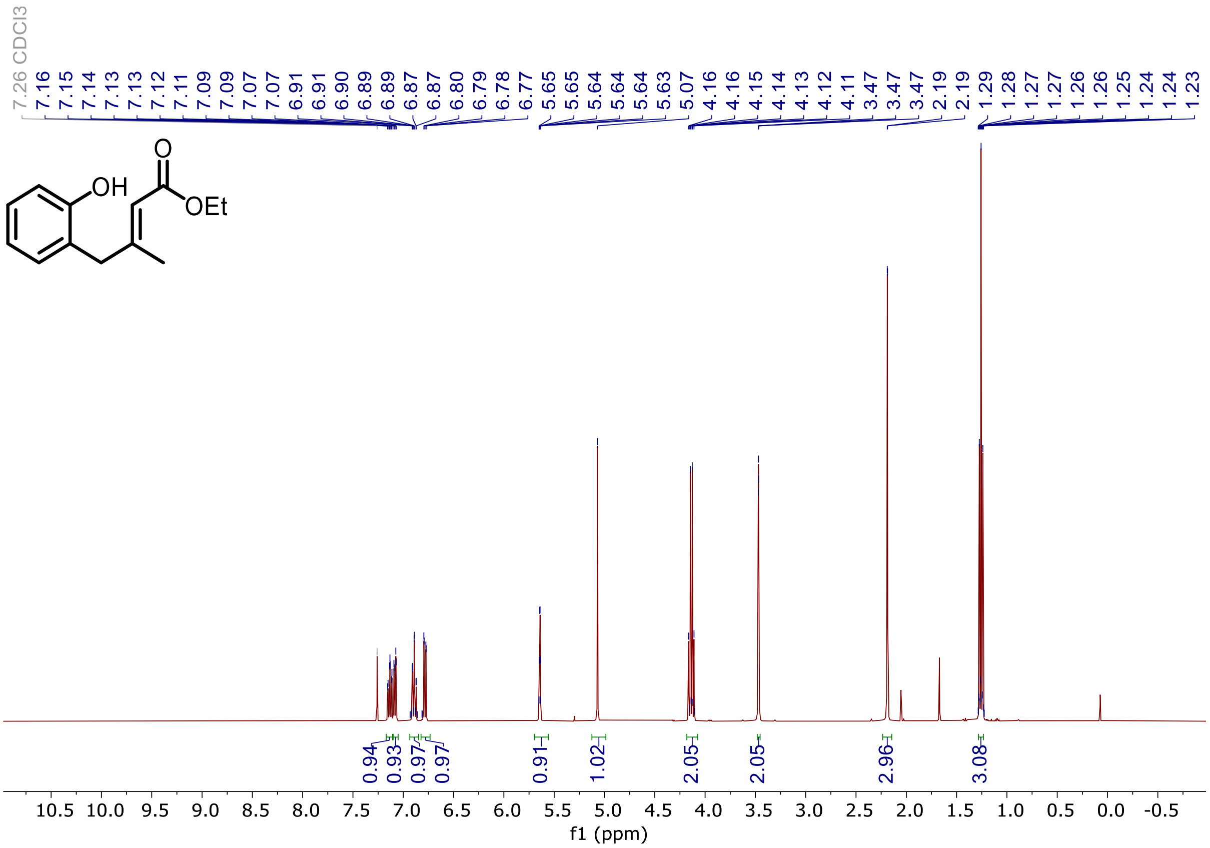

^{13}C NMR: (101 MHz, $CDCl_3$, 298K) of **3ag**

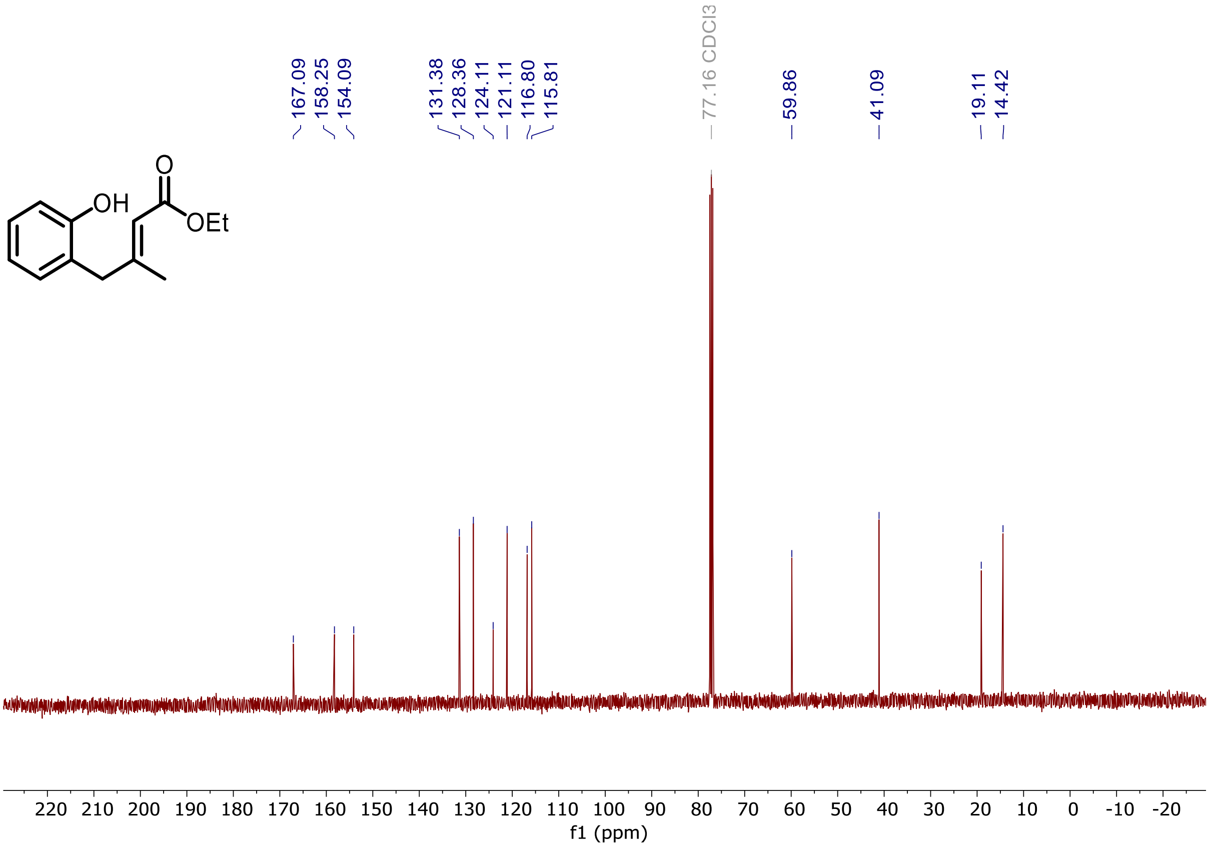

^{1}H NMR: (400 MHz, $CDCl_3$, 298K) of **3ah**

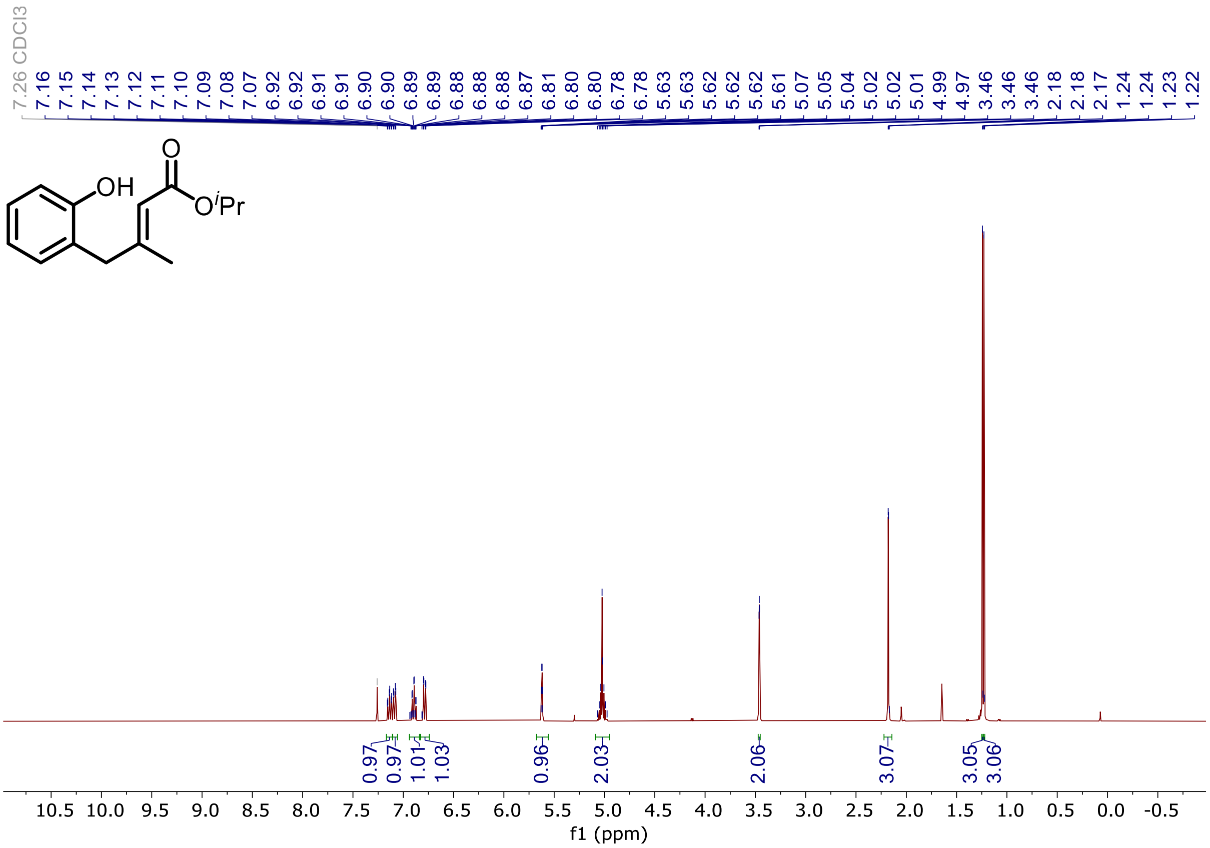

^{13}C NMR: (101 MHz, $CDCl_3$, 298K) of **3ah**

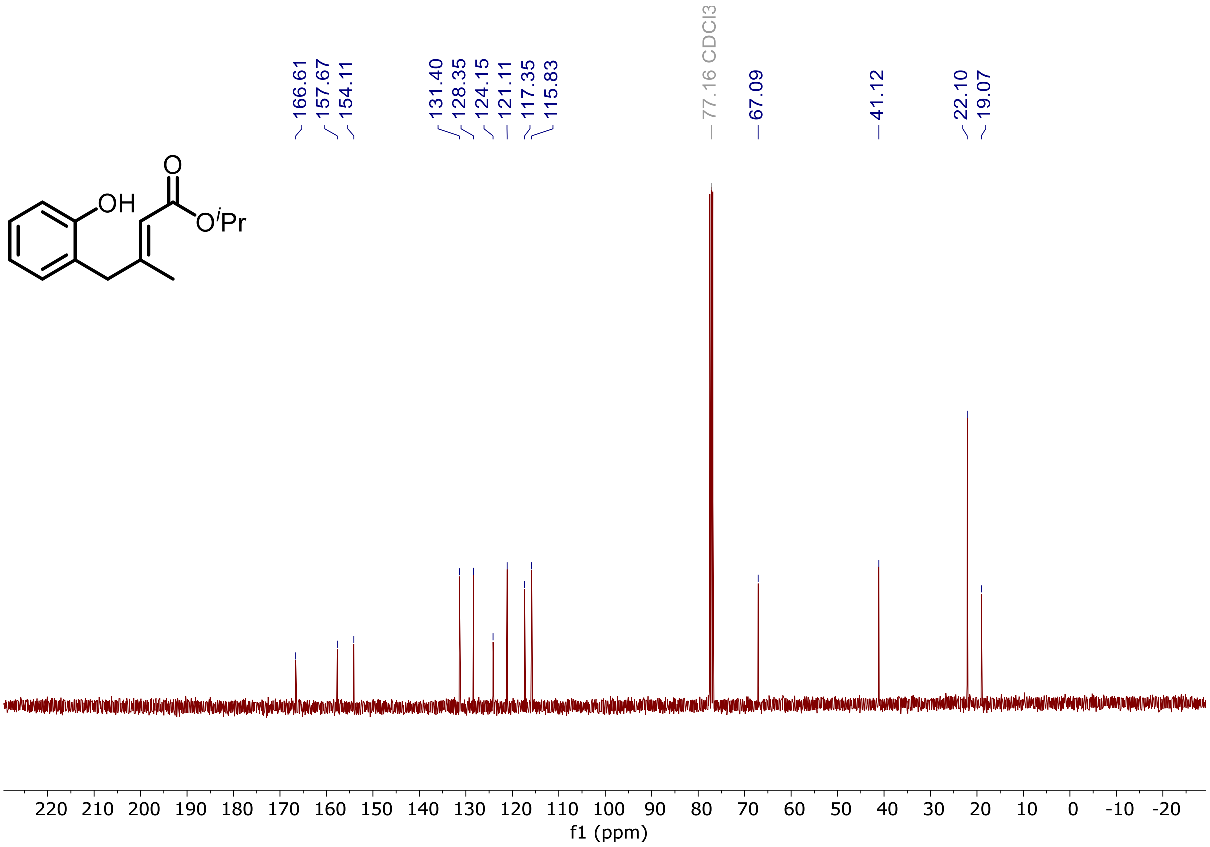

^{1}H NMR: (400 MHz, $CDCl_3$, 298K) of **3ai**

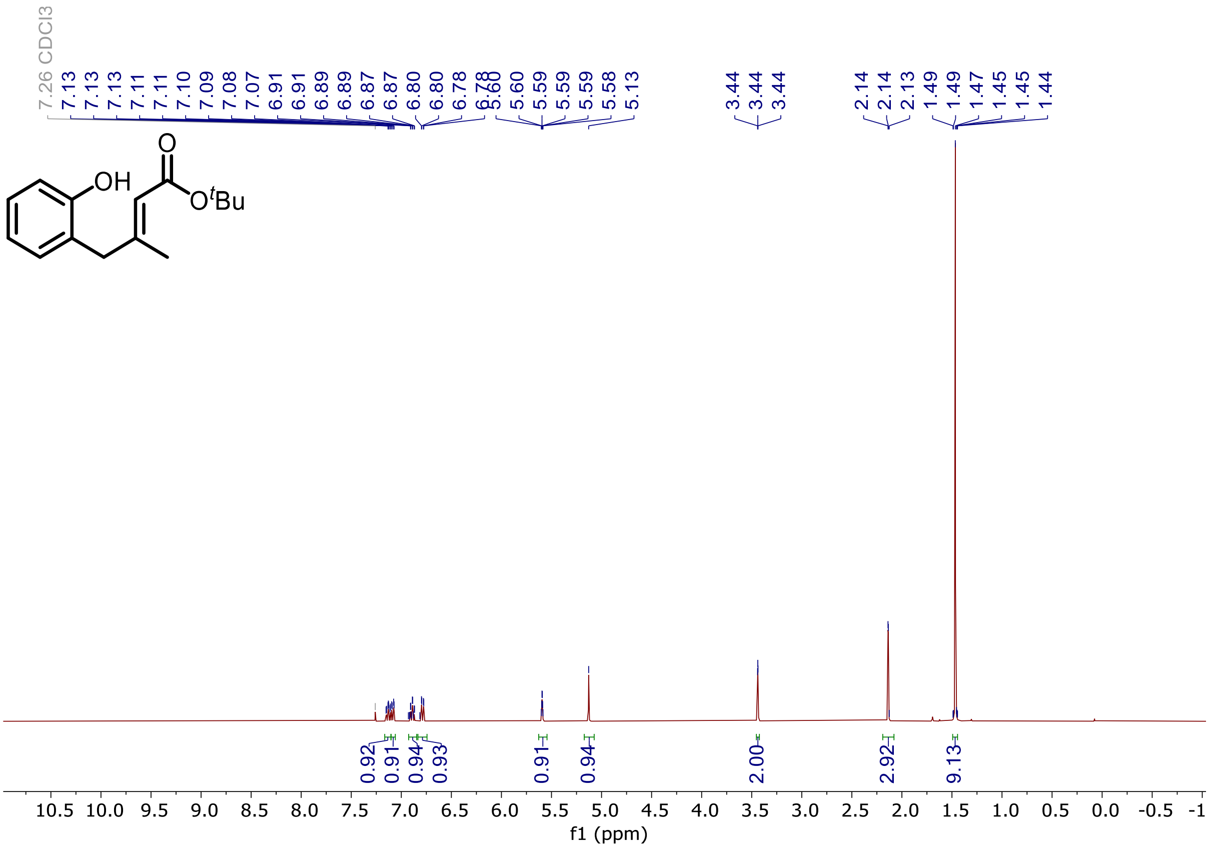

^{13}C NMR: (101 MHz, $CDCl_3$, 298K) of **3ai**

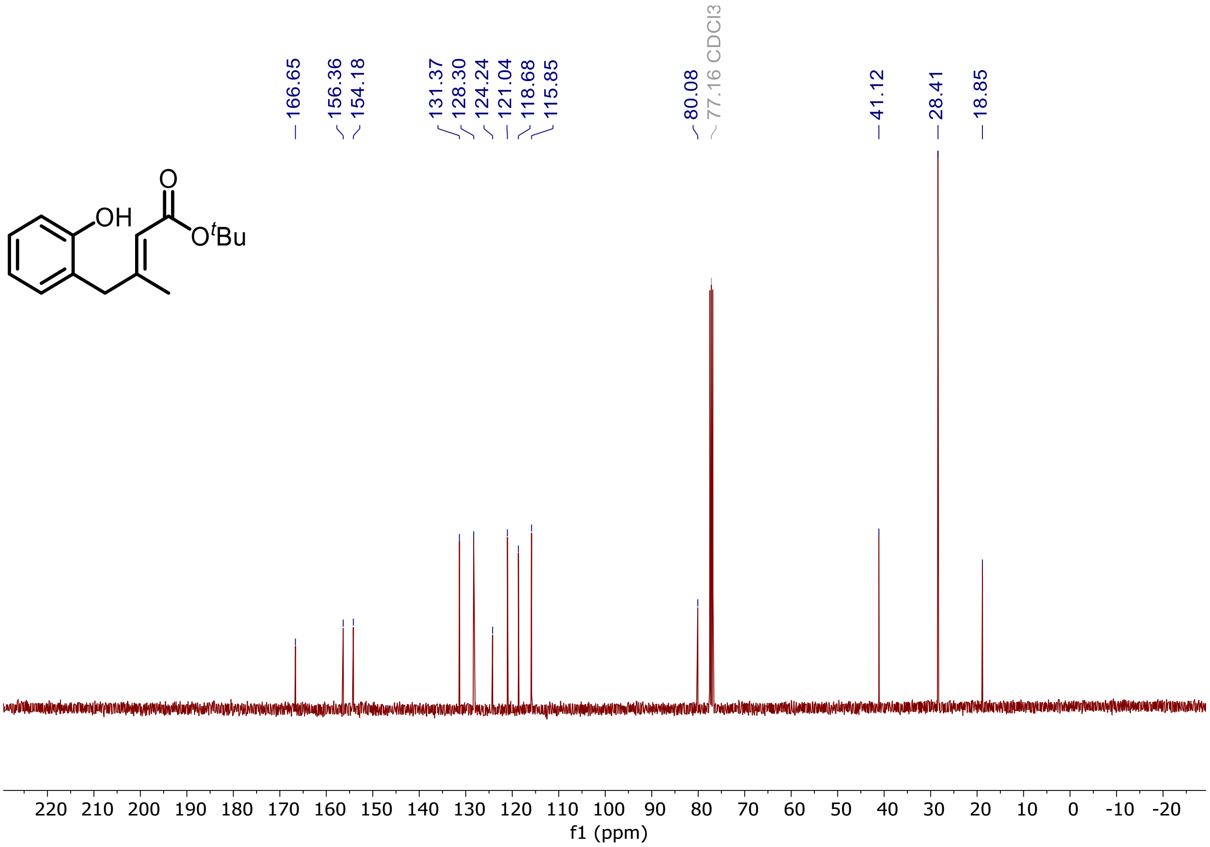

^{1}H NMR: (400 MHz, $CDCl_3$, 298K) of **3aj**

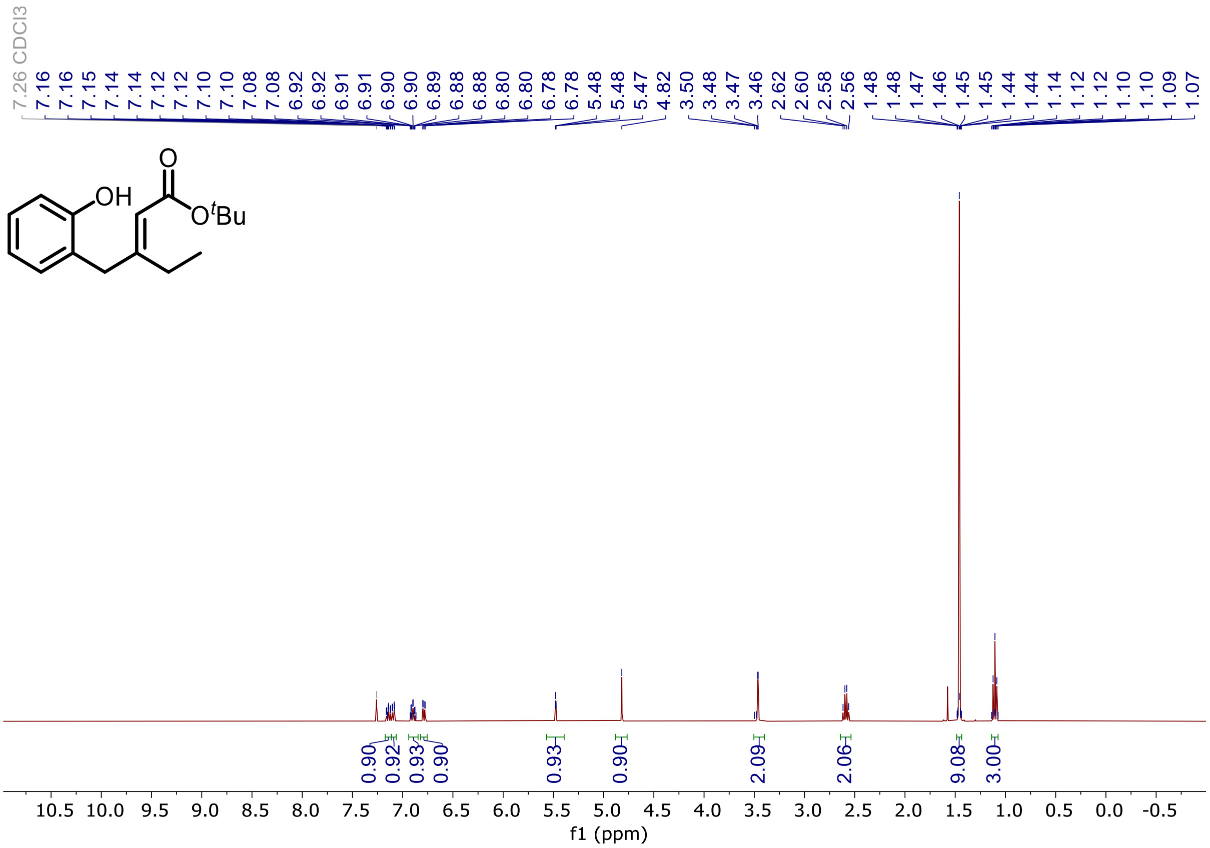

^{13}C NMR: (101 MHz, $CDCl_3$, 298K) of **3aj**

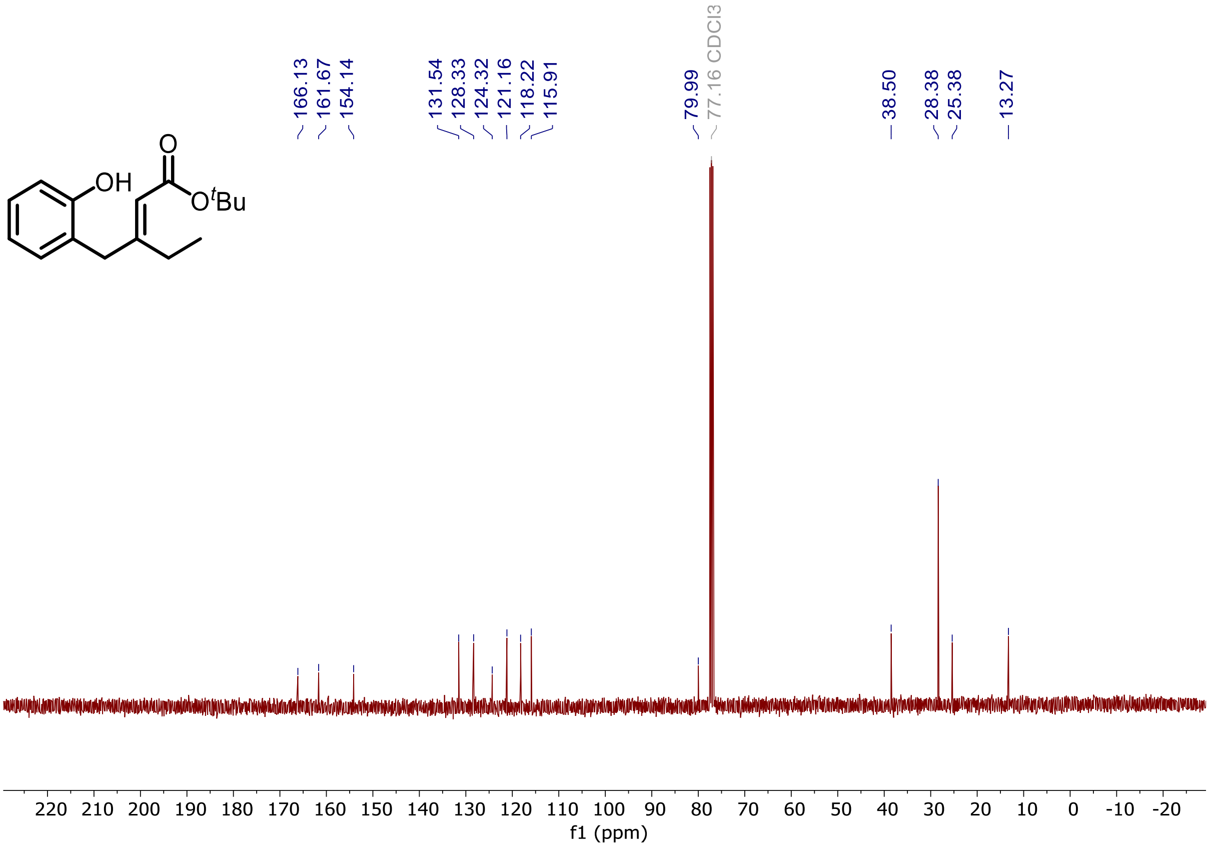

^{1}H NMR: (400 MHz, $CDCl_3$, 298K) of **3ak**

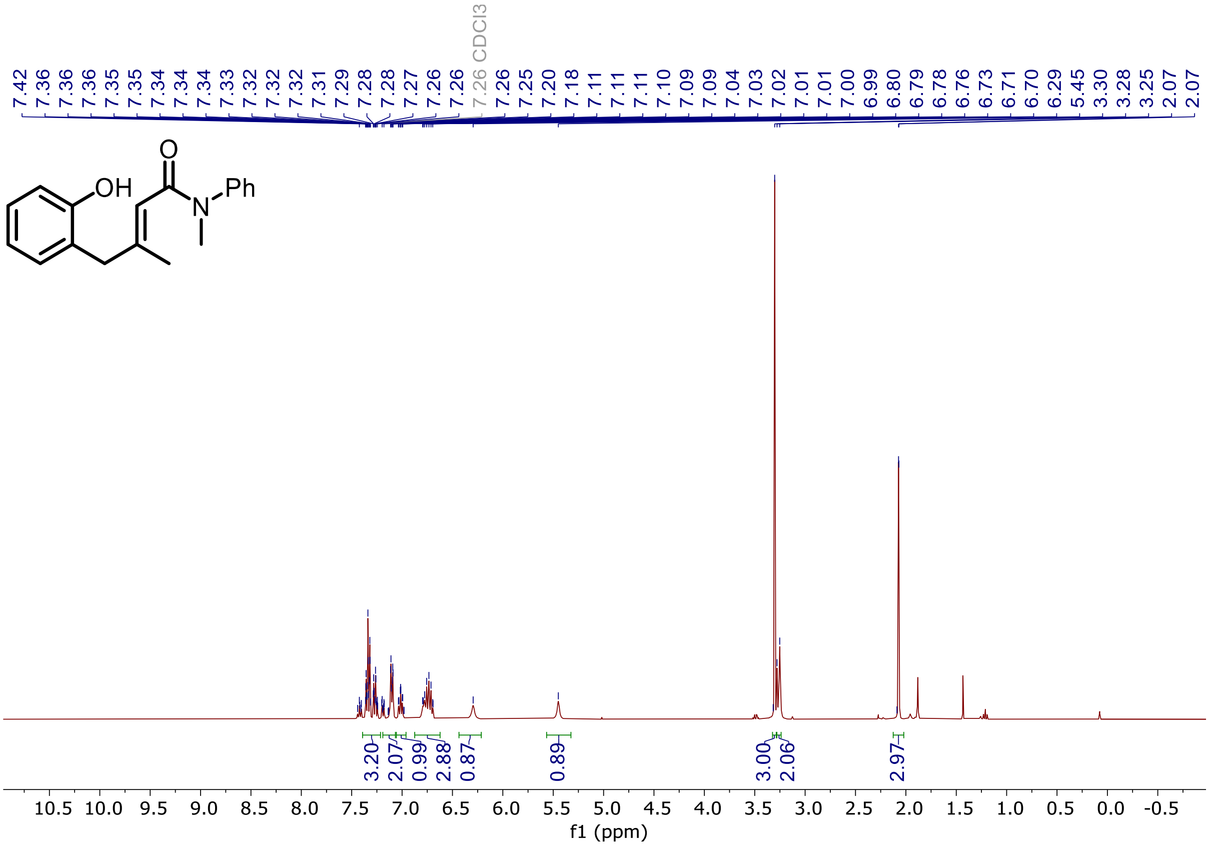

^{13}C NMR: (101 MHz, $CDCl_3$, 298K) of **3ak**

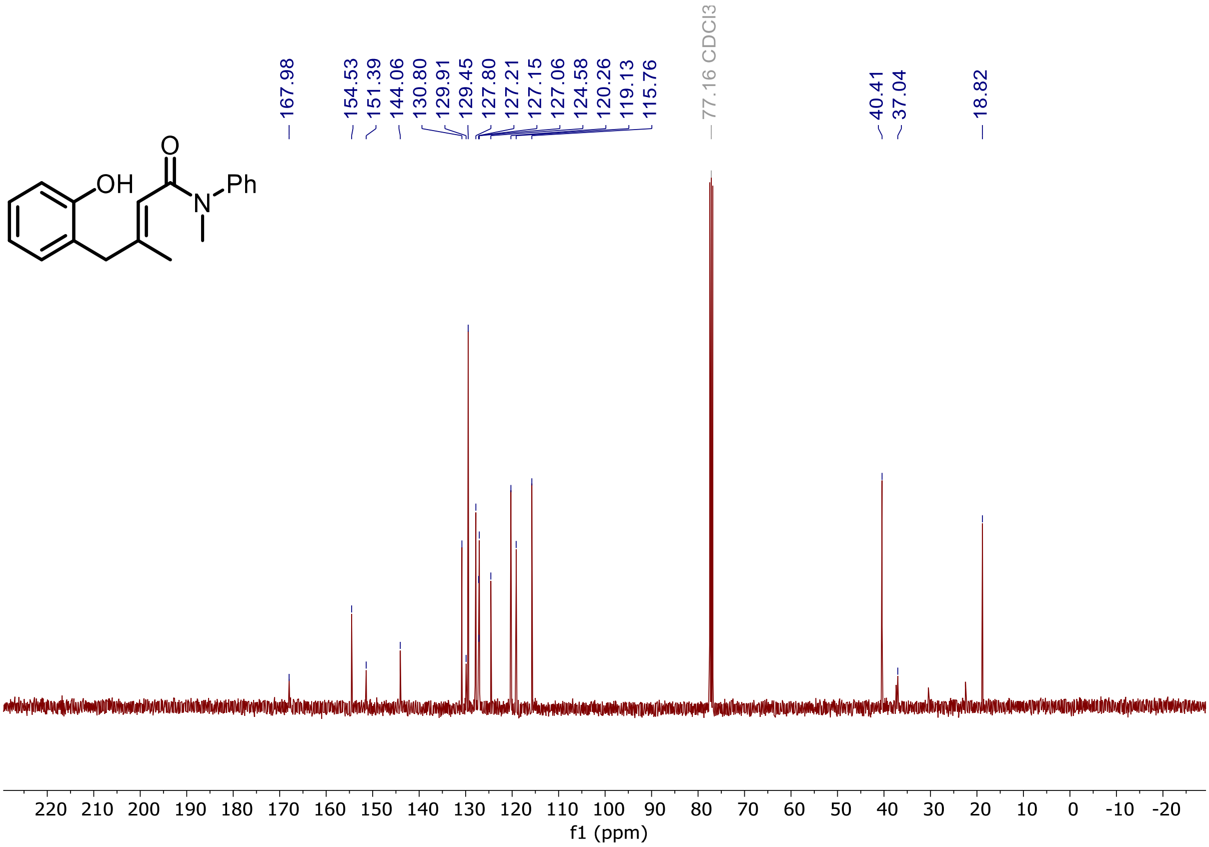

^{1}H NMR: (400 MHz, $(CD_3)_2SO$, 298K) of **3al**

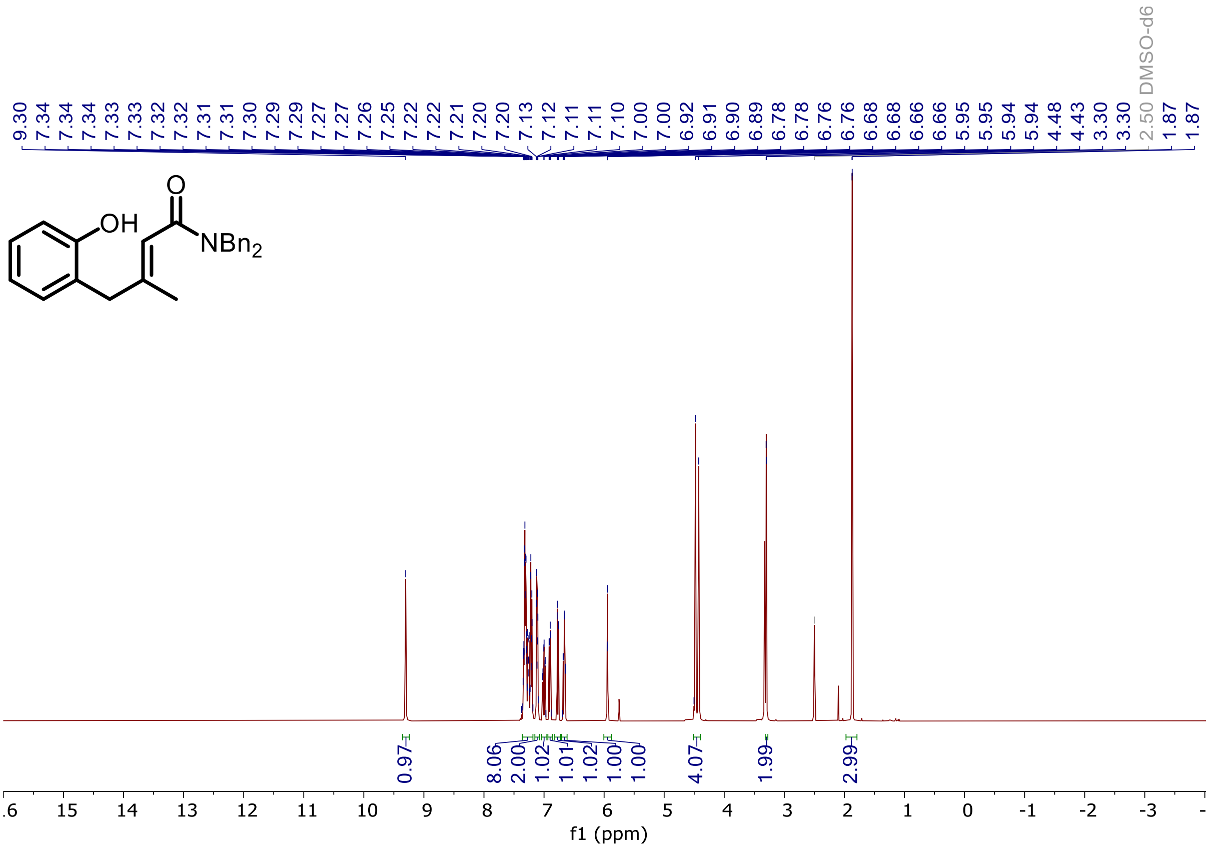

^{13}C NMR: (101 MHz, $(CD_3)_2SO$, 298K) of **3al**

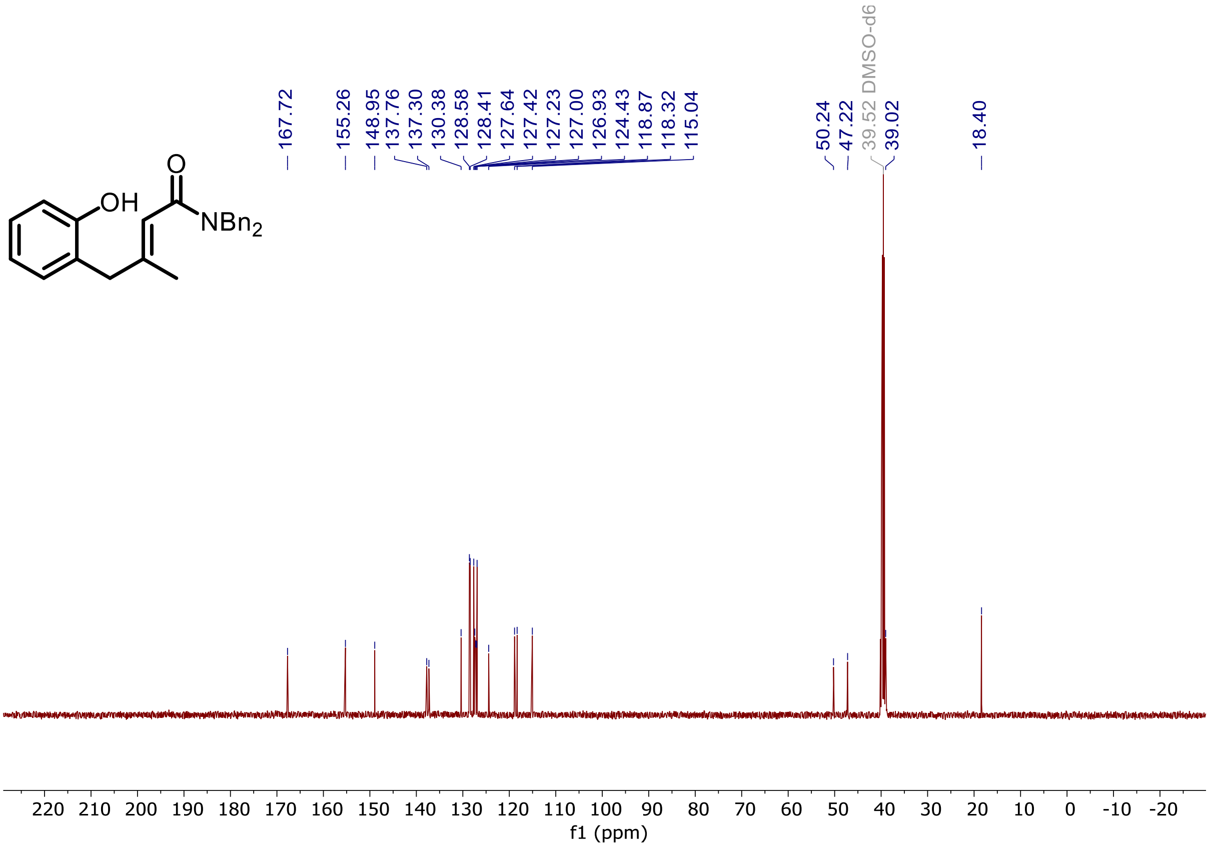

^{1}H NMR: (400 MHz, $(CD_3)_2SO$, 298K) of **S9**

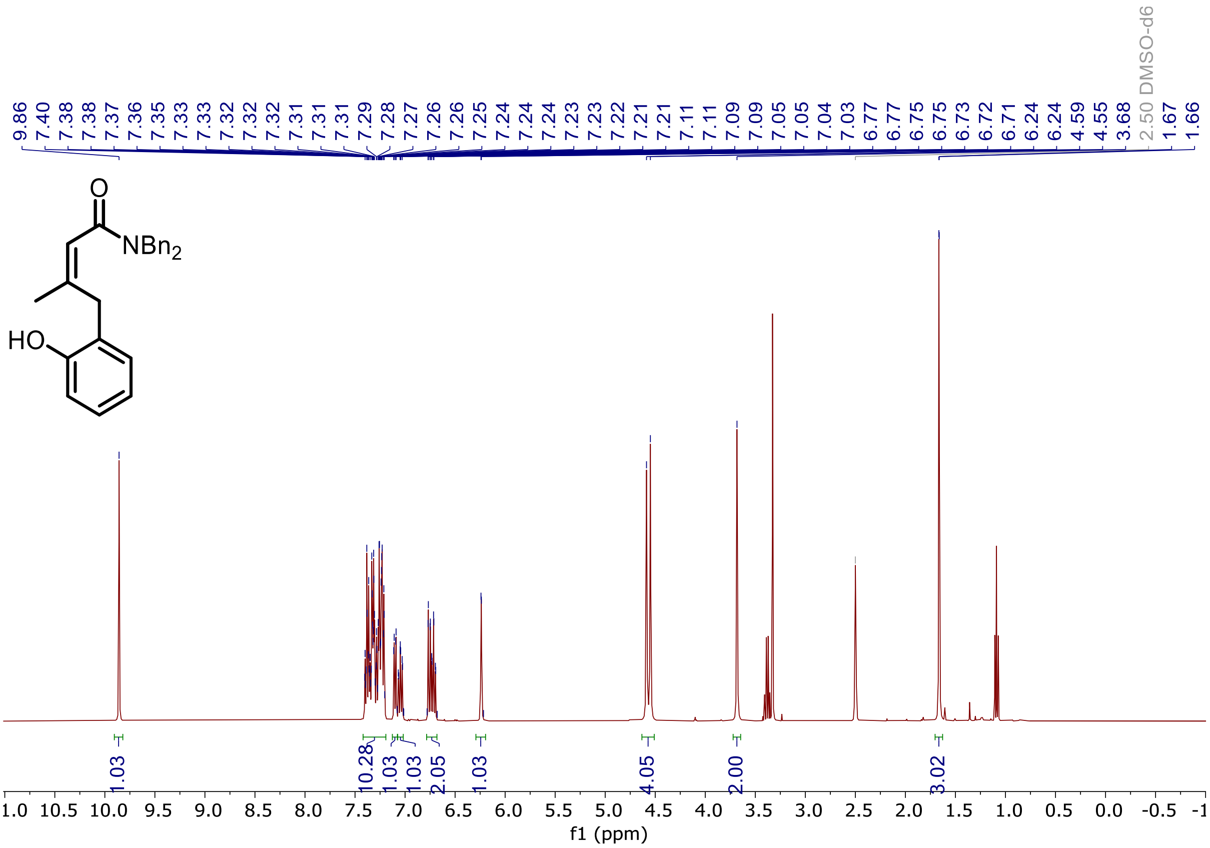

^{13}C NMR: (101 MHz, $(CD_3)_2SO$, 298K) of **S9**

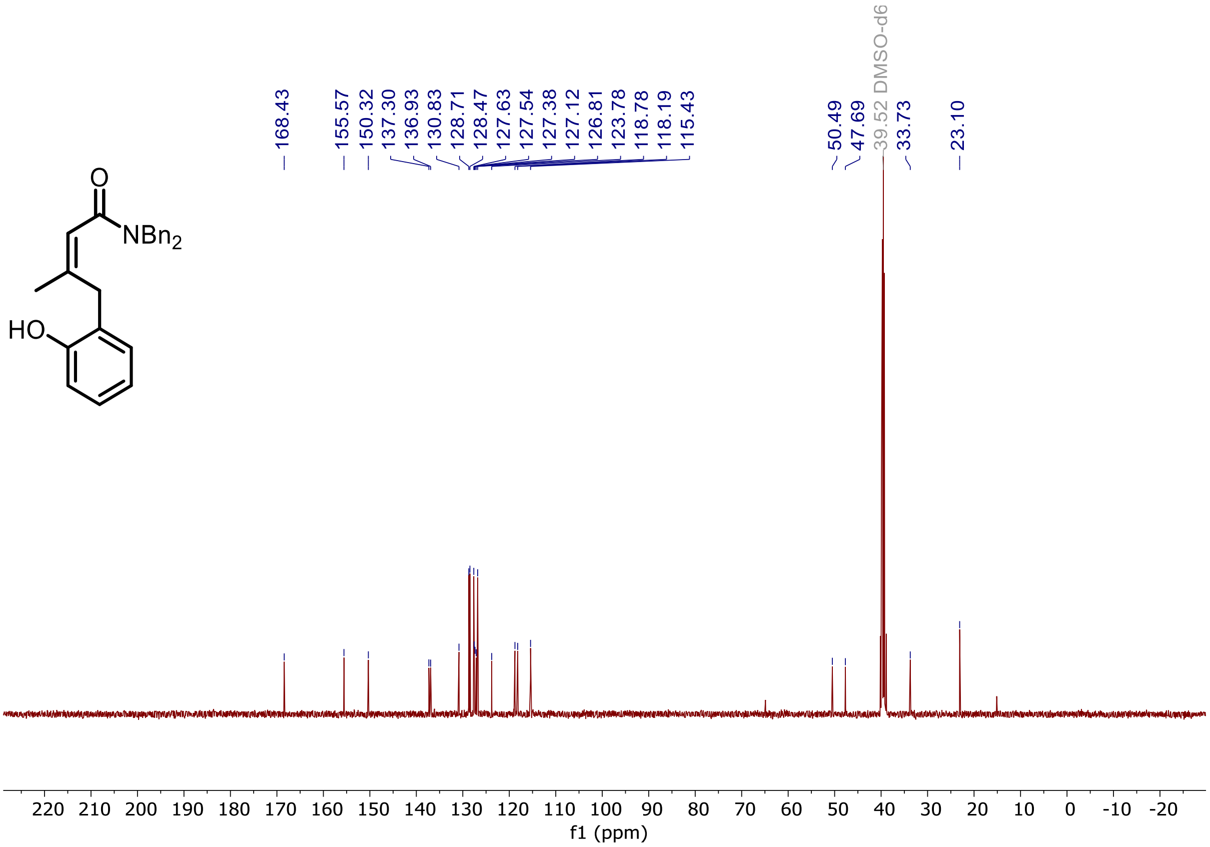

^{1}H NMR: (400 MHz, $CDCl_3$, 298K) of **3an**

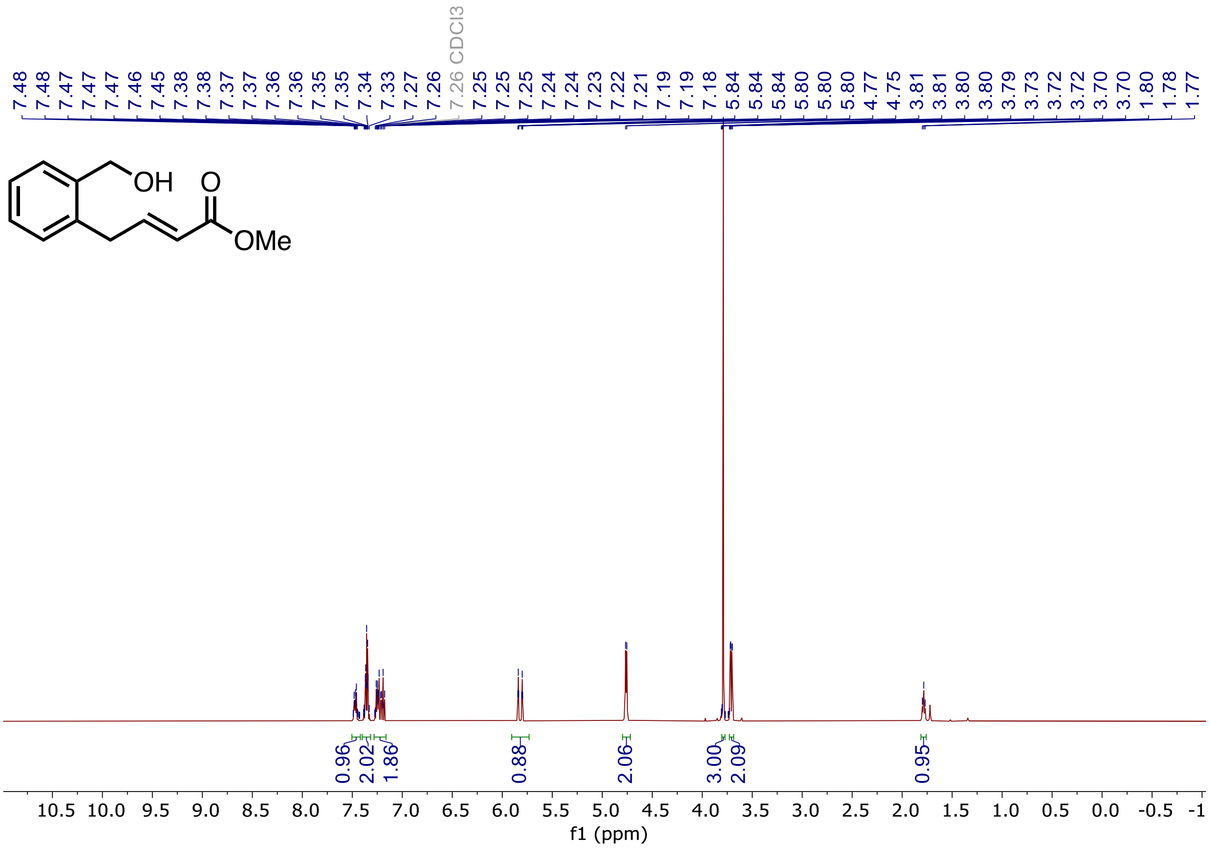

^{13}C NMR: (101 MHz, $CDCl_3$, 298K) of **3an**

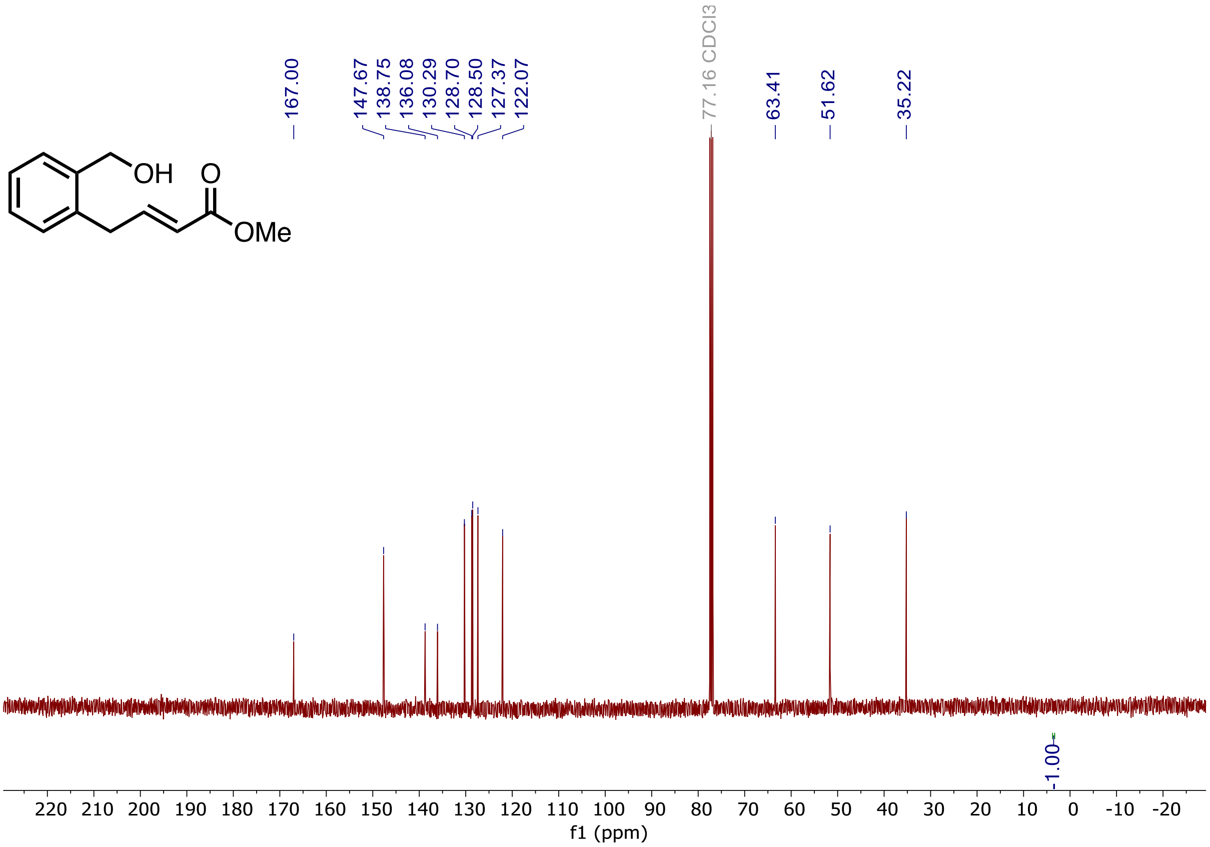

^{1}H NMR: (400 MHz, $CDCl_3$, 298K) of **3ao**

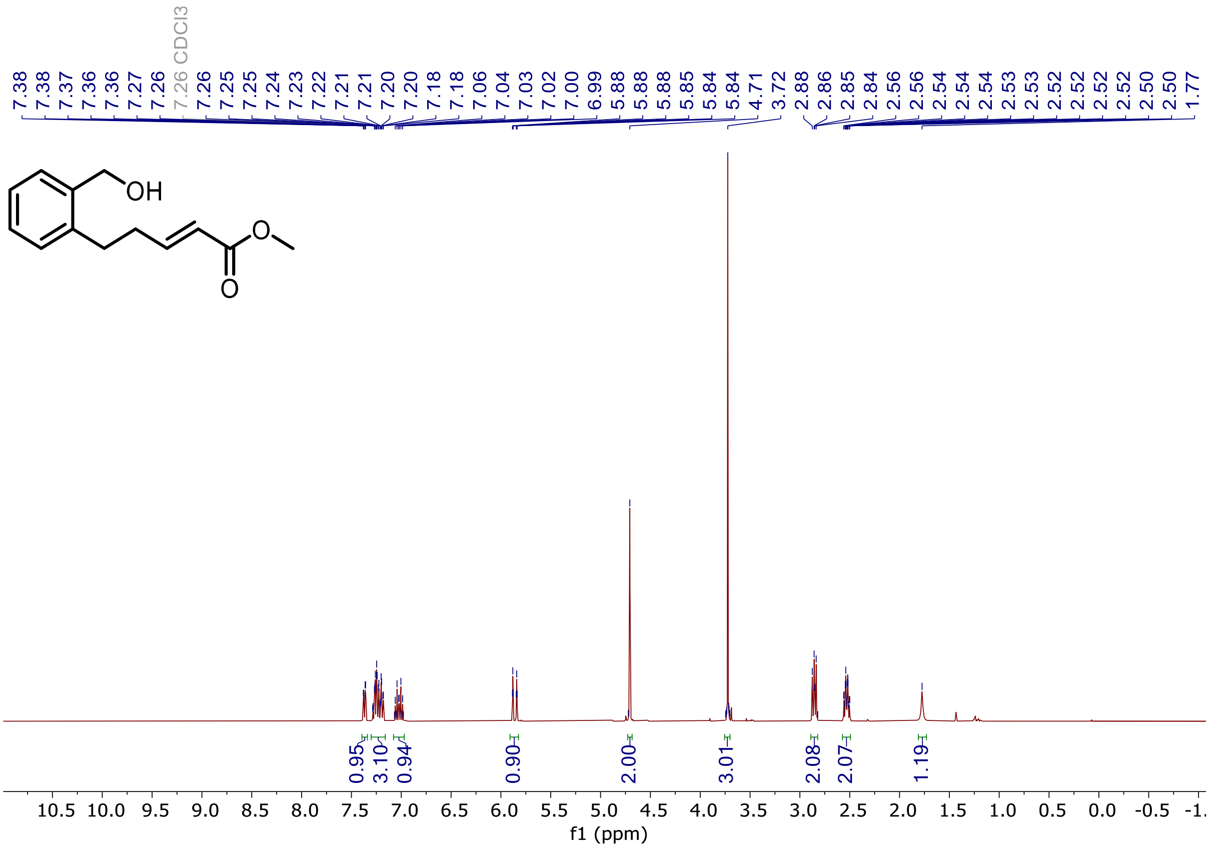

^{13}C NMR: (101 MHz, $CDCl_3$, 298K) of **3ao**

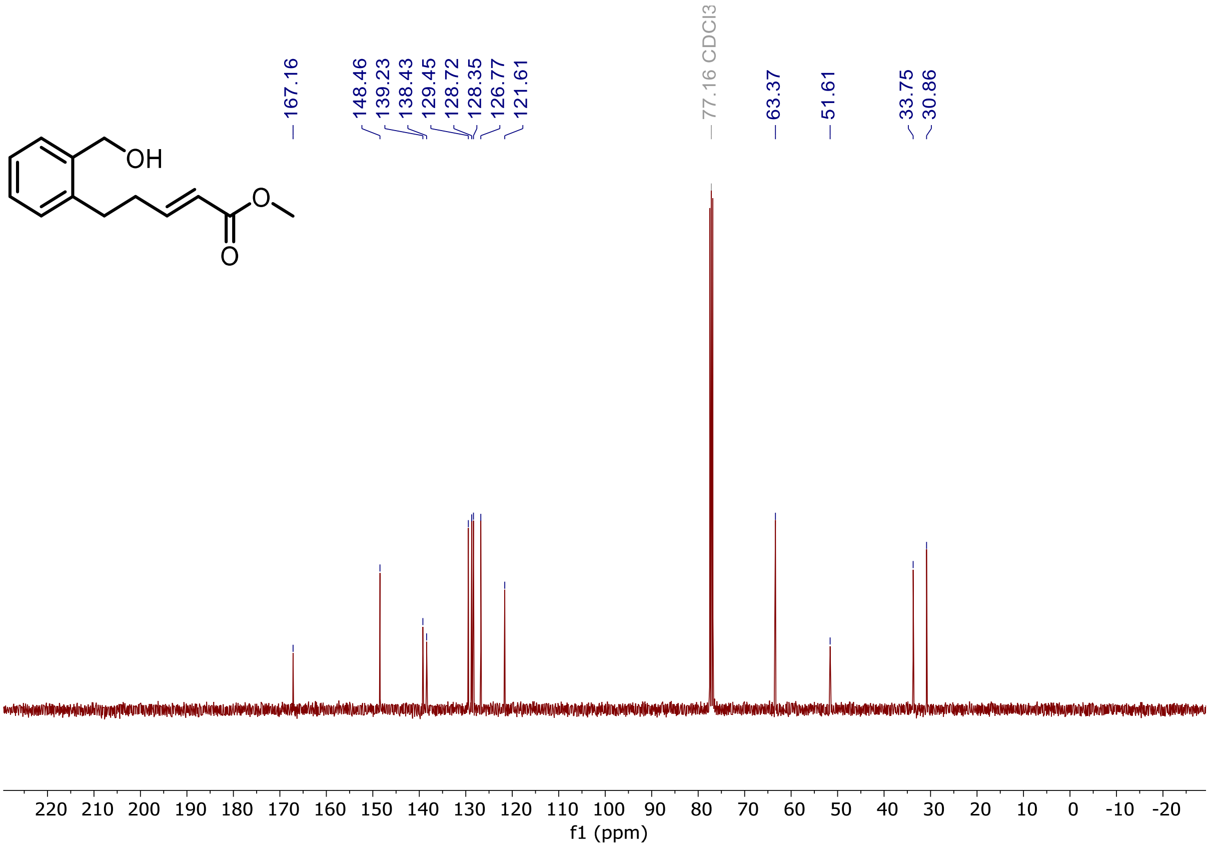

^{1}H NMR: (400 MHz, $CDCl_3$, 298K) of **3ap**

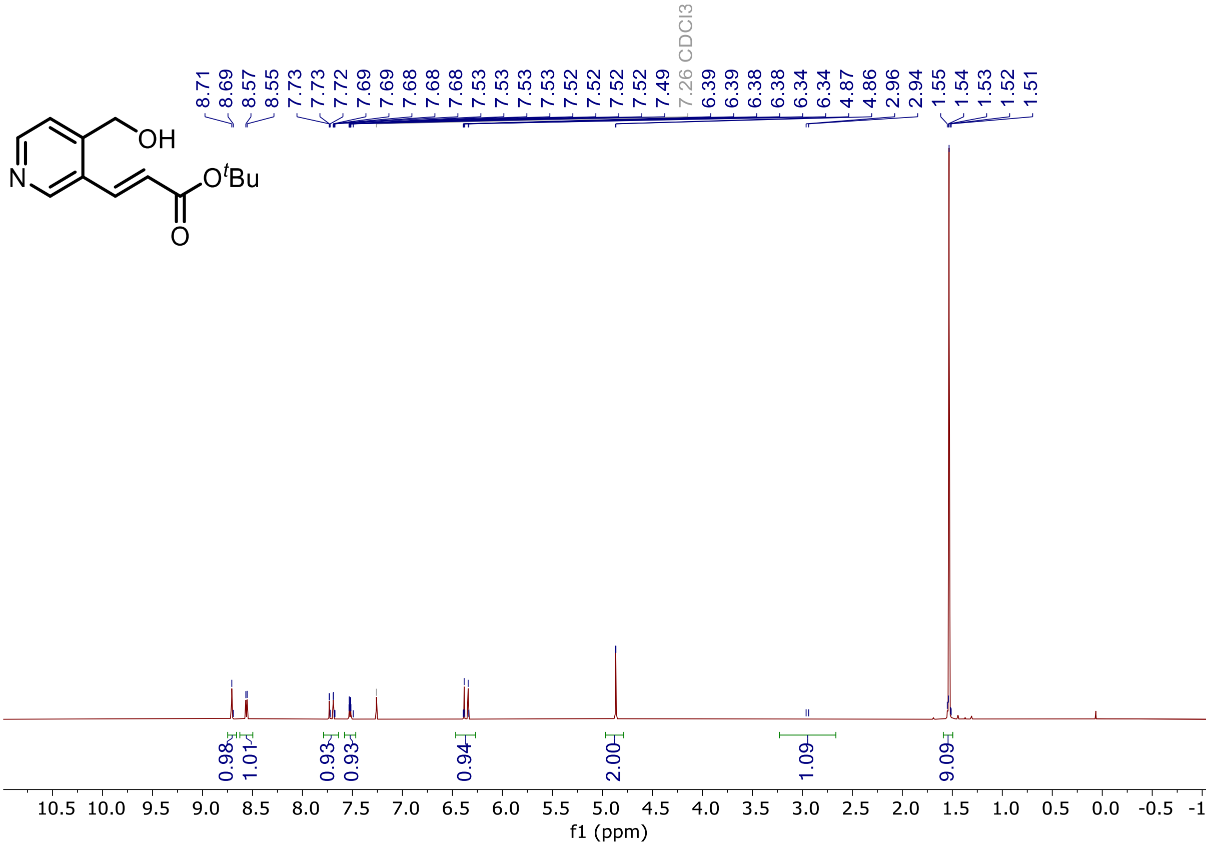

^{13}C NMR: (101 MHz, $CDCl_3$, 298K) of **3ap**

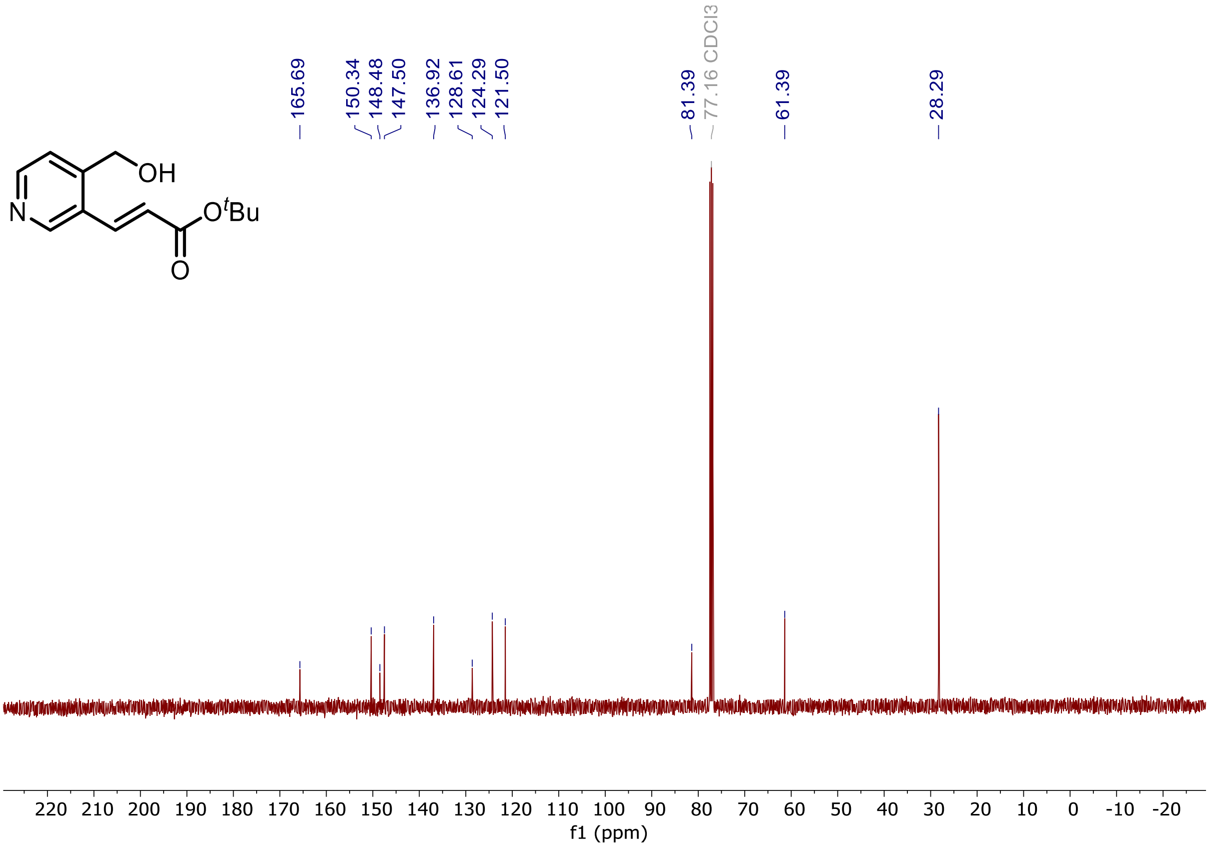

^{1}H NMR: (400 MHz, $CDCl_3$, 298K) of **4c**

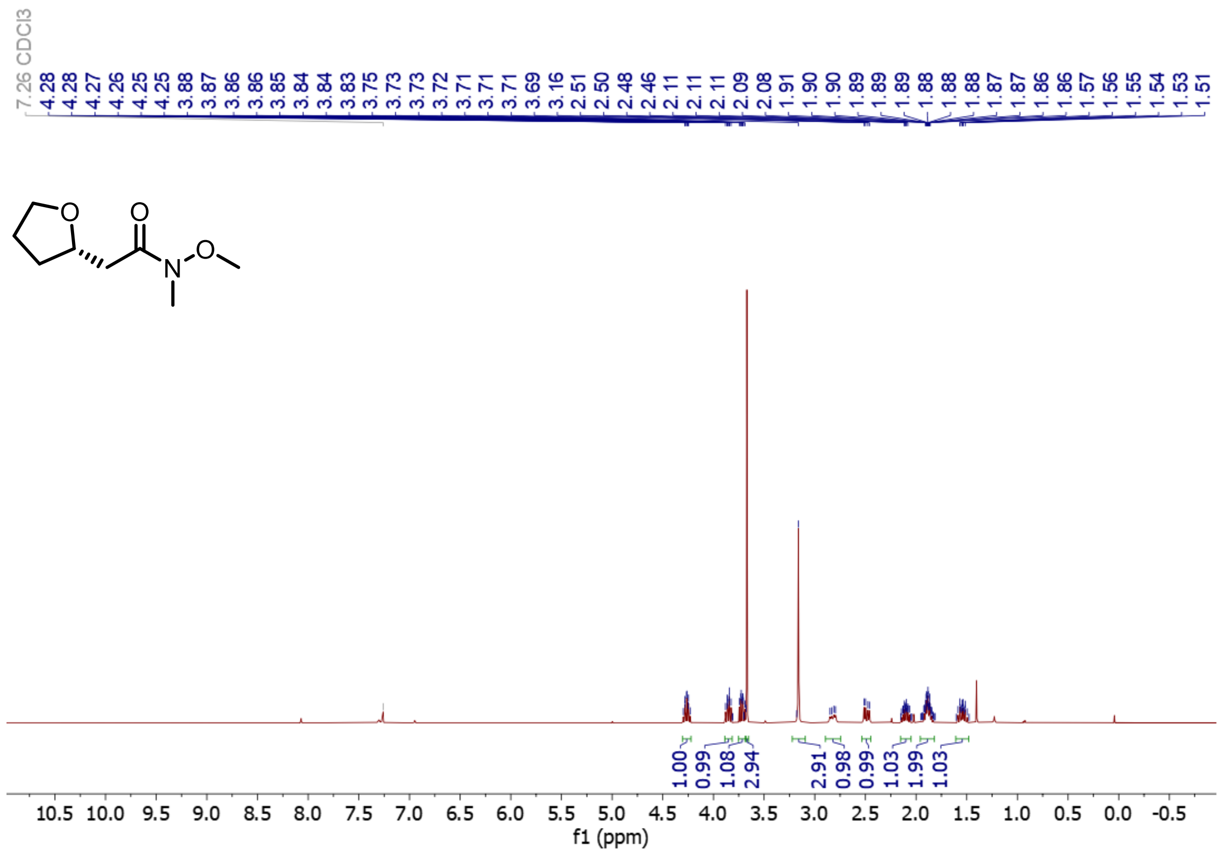

^{13}C NMR: (101 MHz, $CDCl_3$, 298K) of **4c**

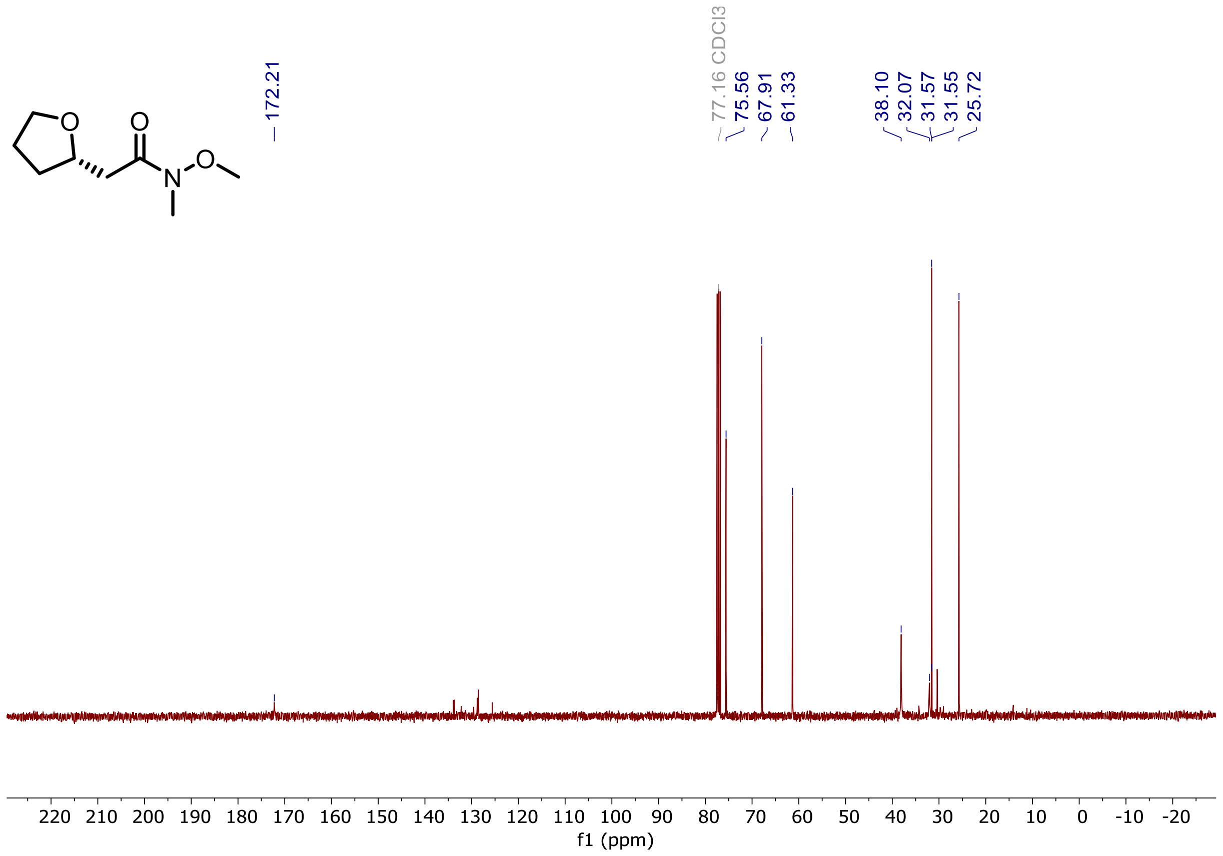

^{1}H NMR: (400 MHz, $CDCl_3$, 298K) of **4d**

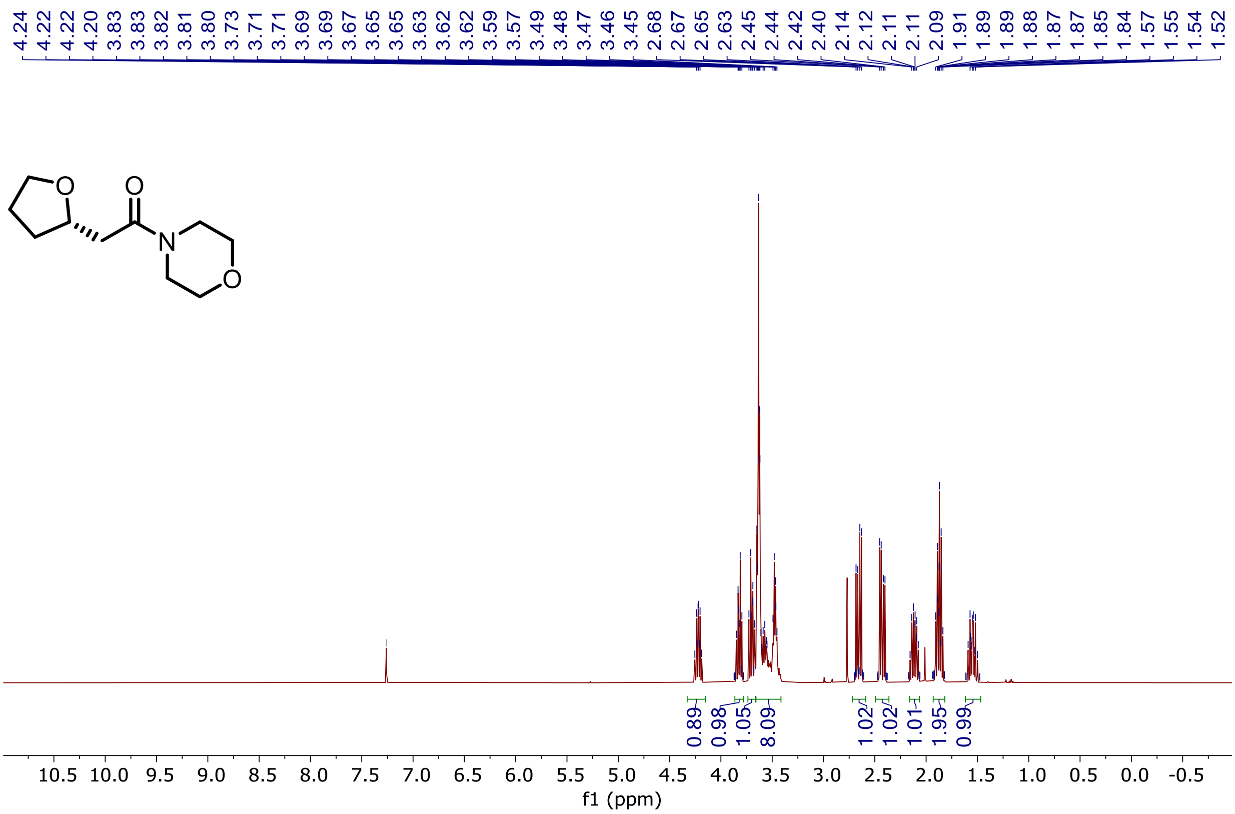

^{13}C NMR: (101 MHz, $CDCl_3$, 298K) of **4d**

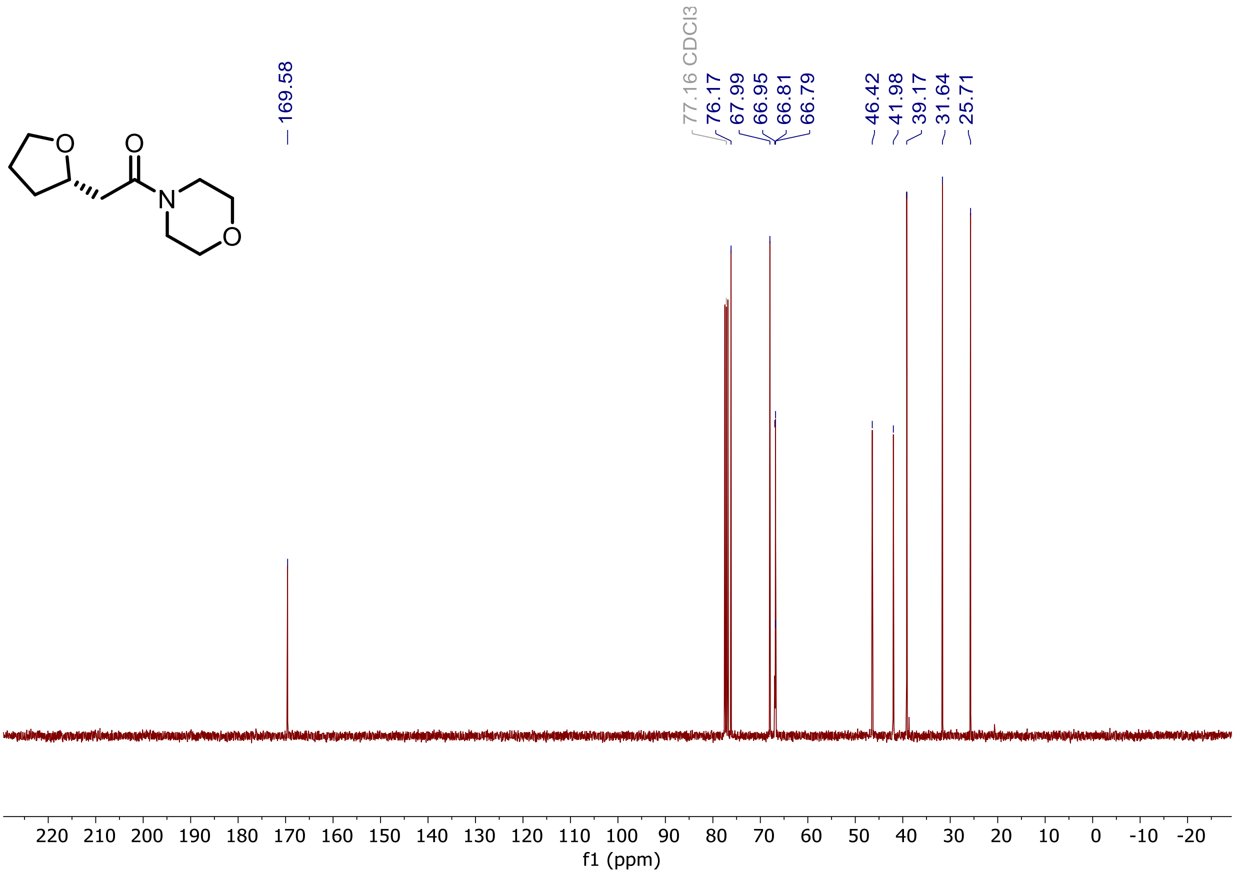

^{1}H NMR: (400 MHz, $CDCl_3$, 298K) of **4e**

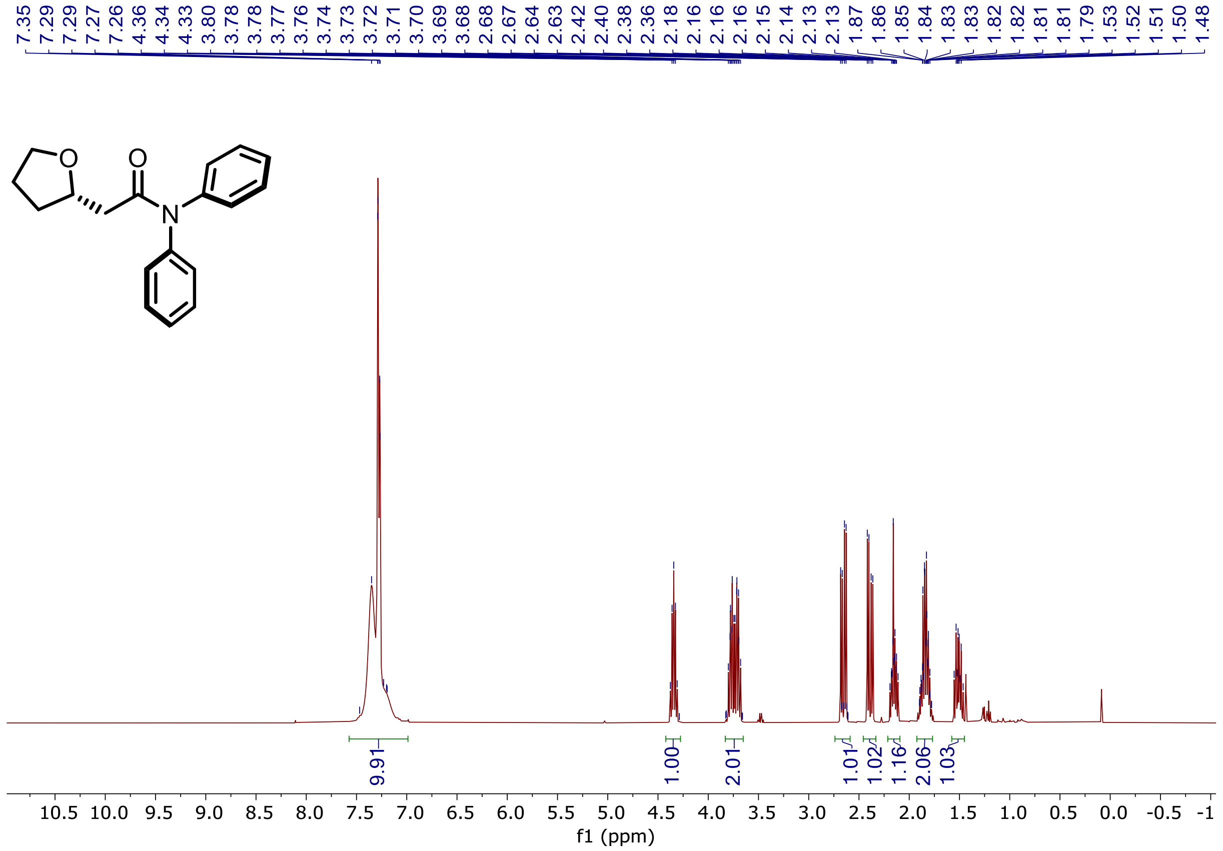

^{13}C NMR: (101 MHz, $CDCl_3$, 298K) of **4e**

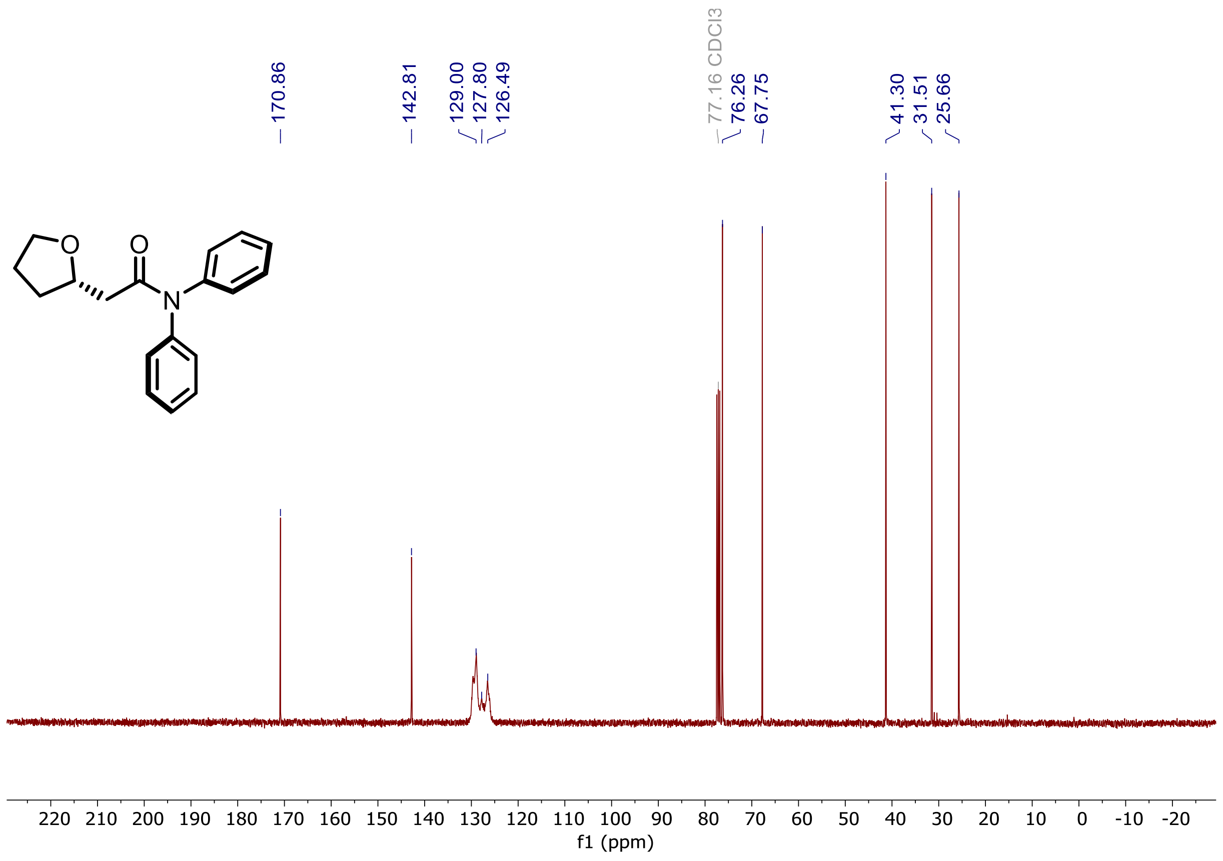

^{1}H NMR: (400 MHz, $CDCl_3$, 298K) of **4f**

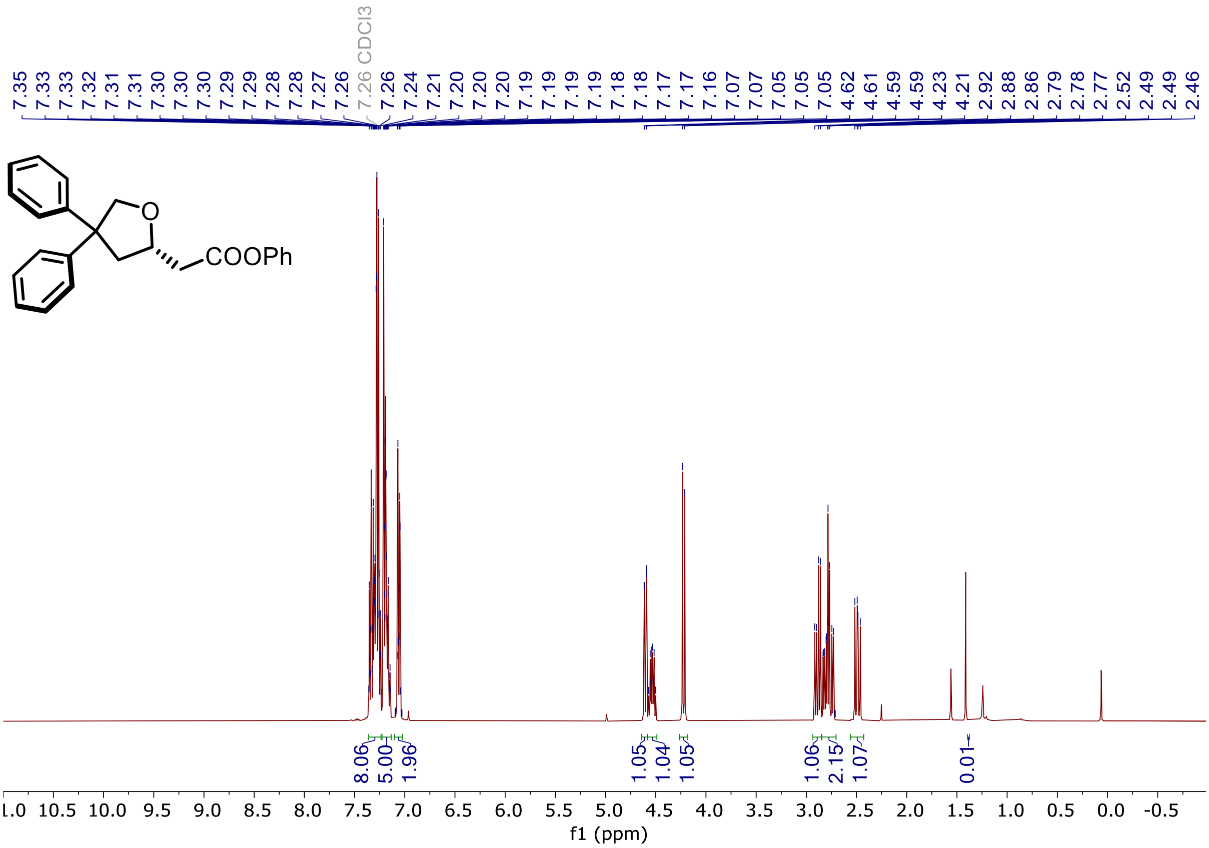

^{13}C NMR: (101 MHz, $CDCl_3$, 298K) of **4f**

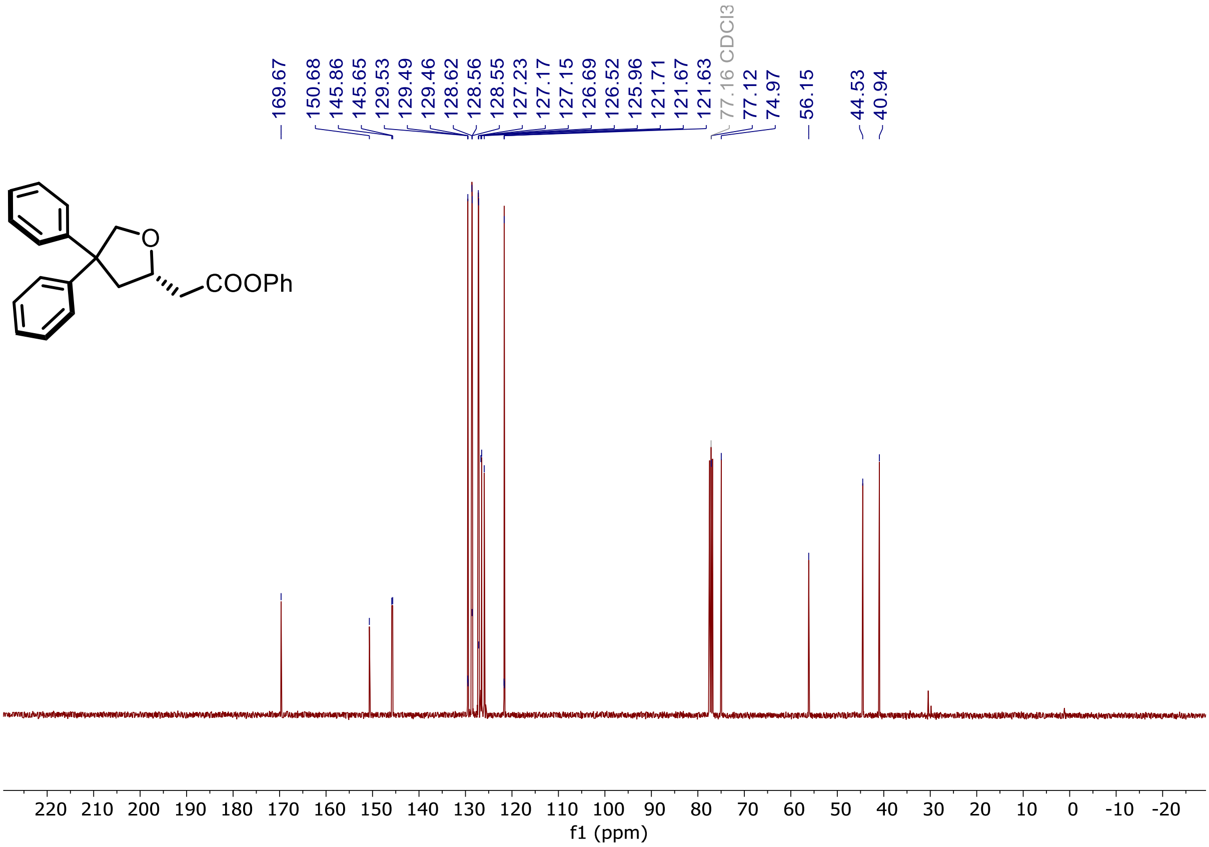

^{1}H NMR: (400 MHz, $CDCl_3$, 298K) of **4g**

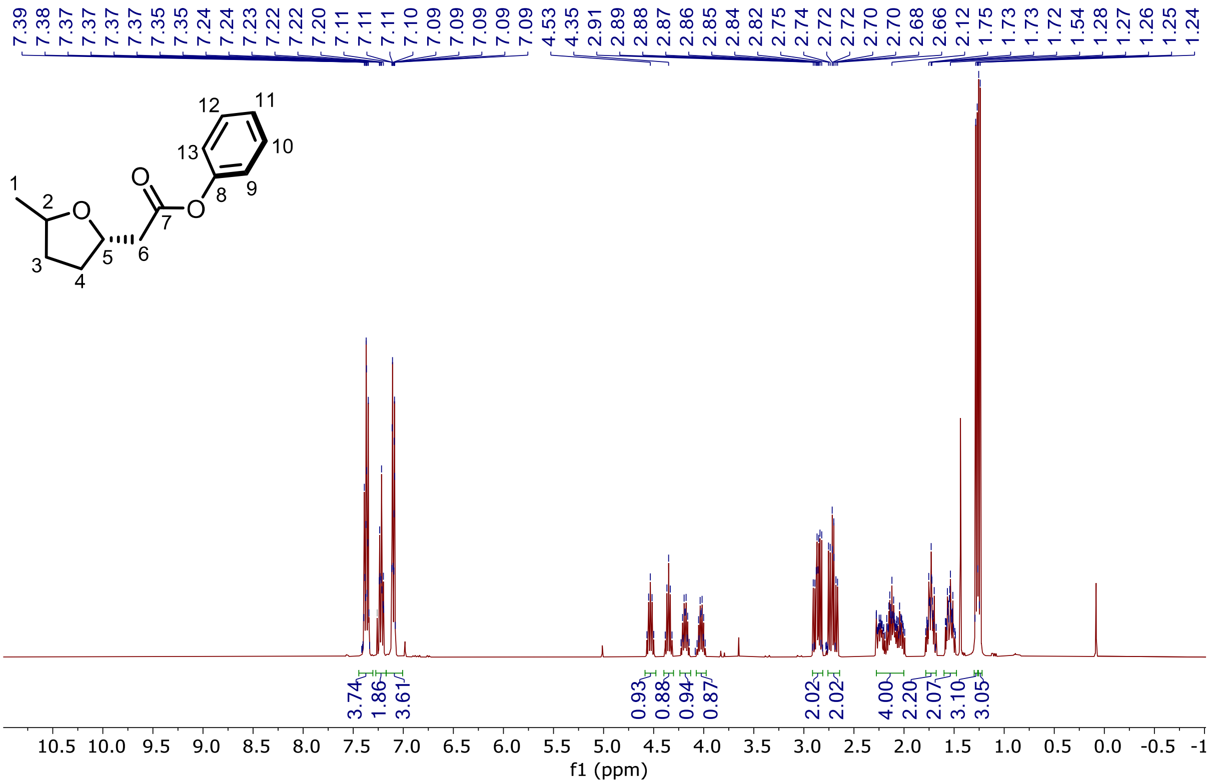

^{13}C NMR: (101 MHz, $CDCl_3$, 298K) of **4g**

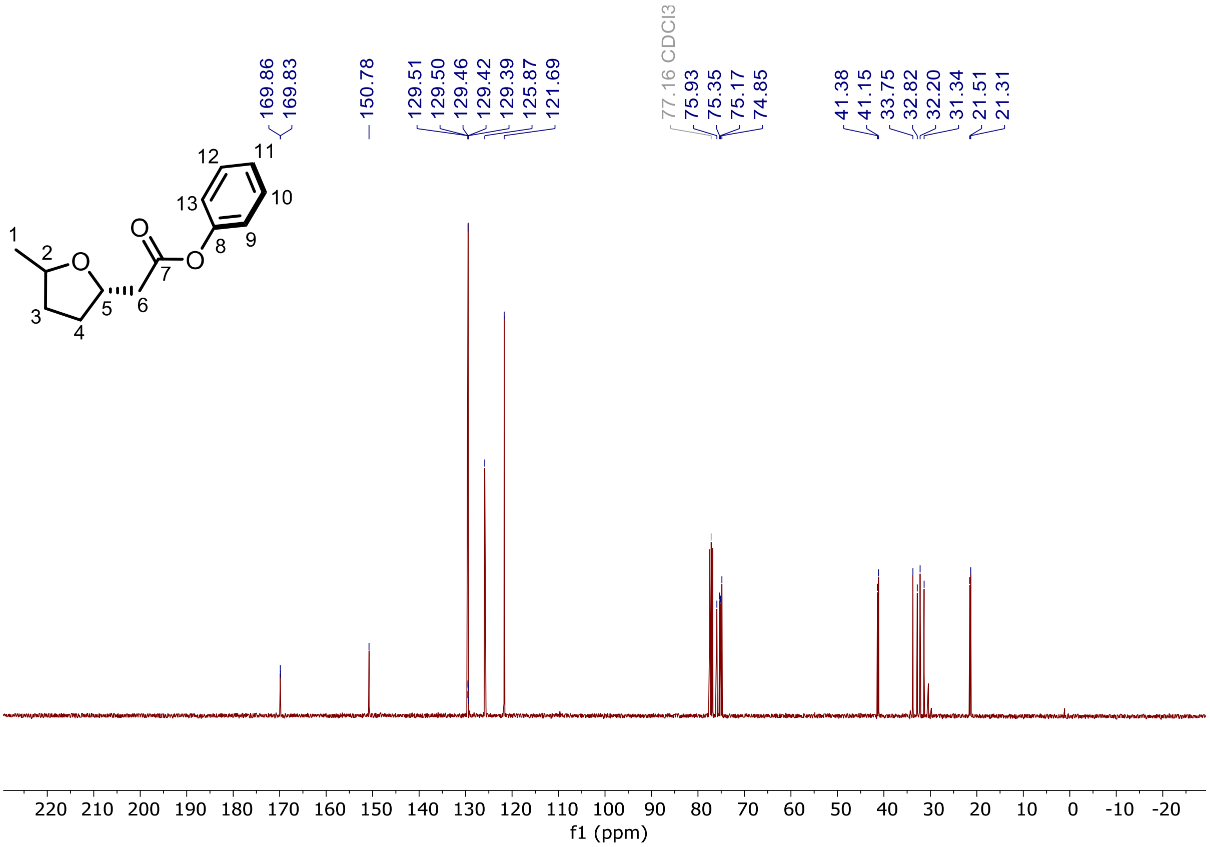

^{1}H NMR: (400 MHz, $CDCl_3$, 298K) of **4h**

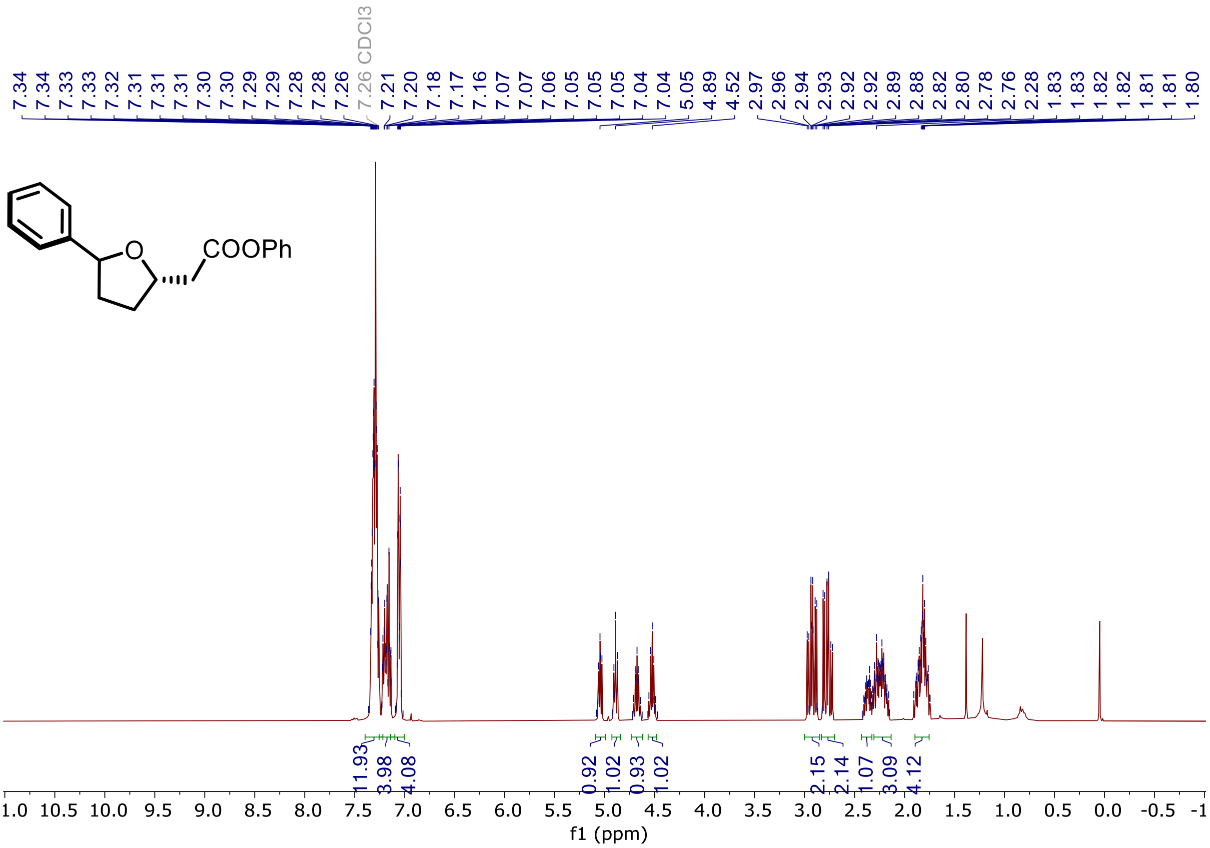

^{13}C NMR: (101 MHz, $CDCl_3$, 298K) of **4h**

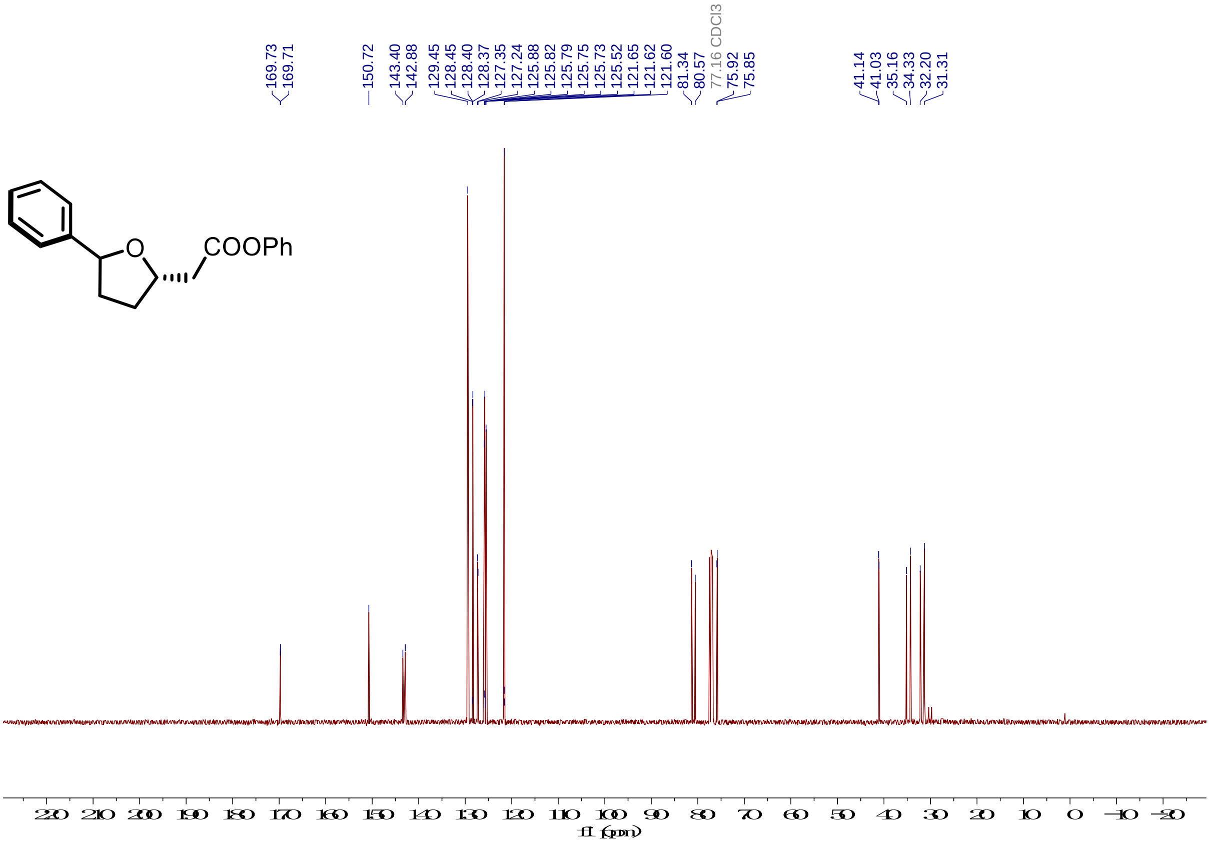

^{1}H NMR: (400 MHz, $CDCl_3$, 298K) of **4i**

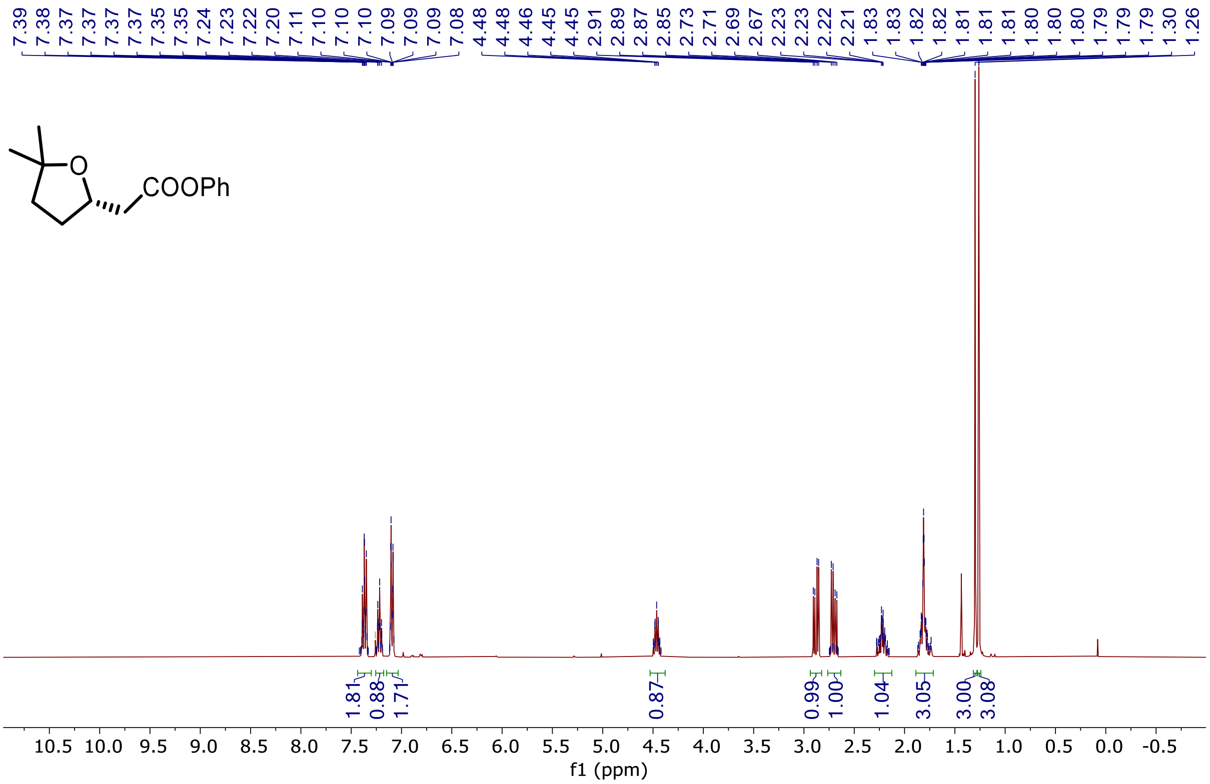

^{13}C NMR: (101 MHz, $CDCl_3$, 298K) of **4i**

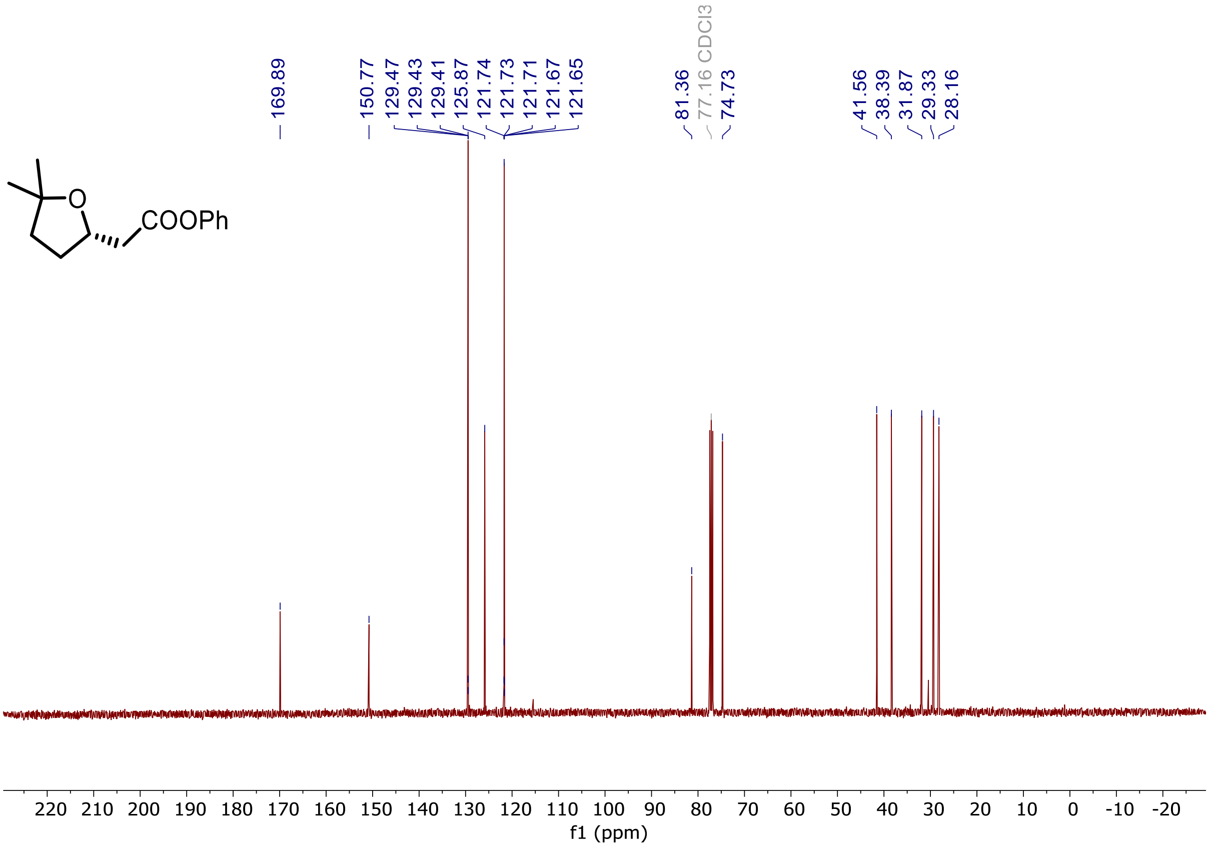

^{1}H NMR: (400 MHz, $CDCl_3$, 298K) of **4j**

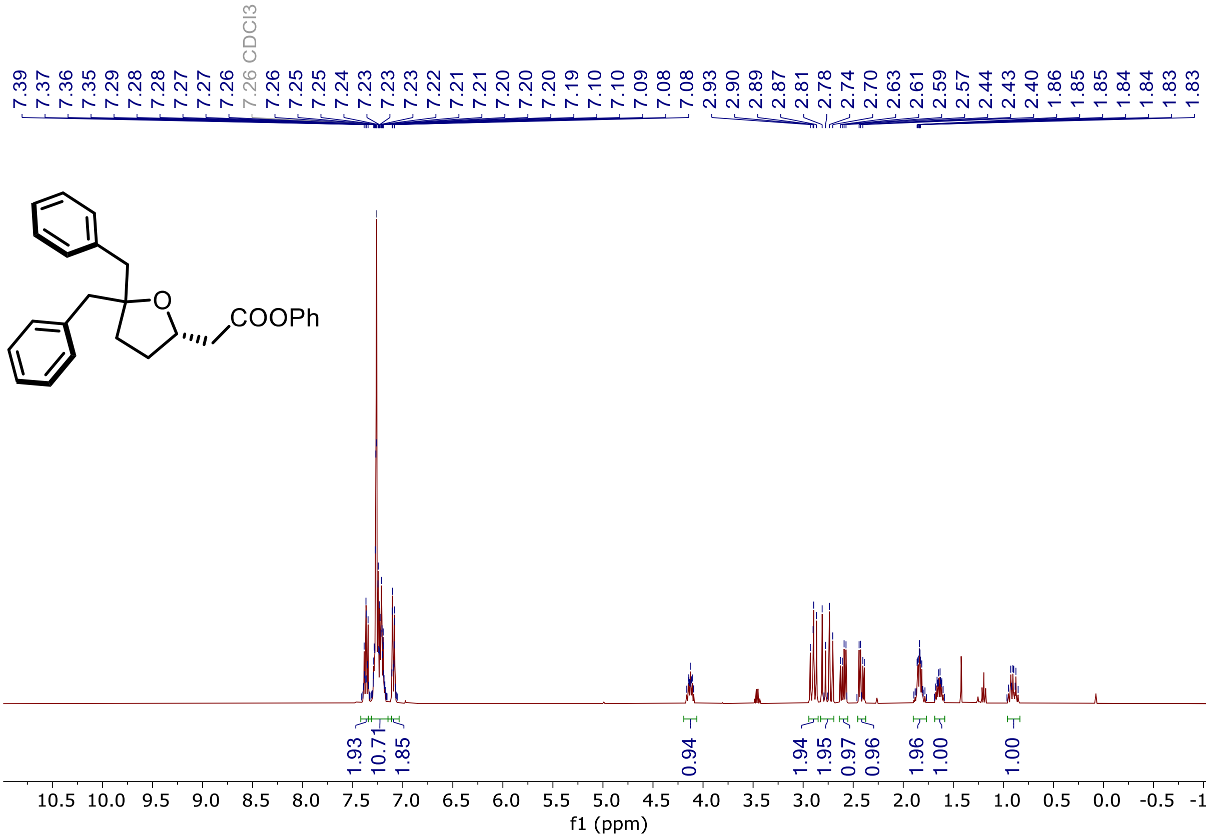

^{13}C NMR: (101 MHz, $CDCl_3$, 298K) of **4j**

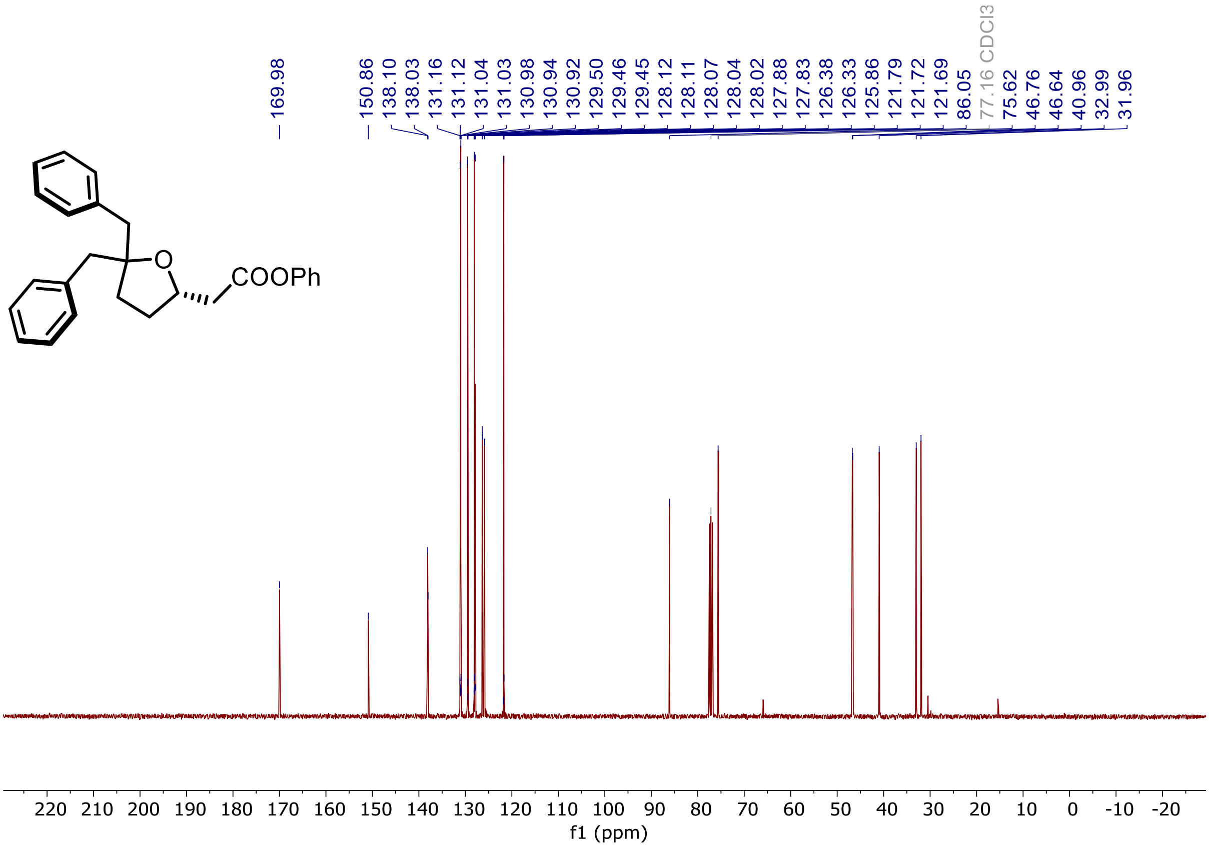

^{1}H NMR: (400 MHz, $CDCl_3$, 298K) of **4k**

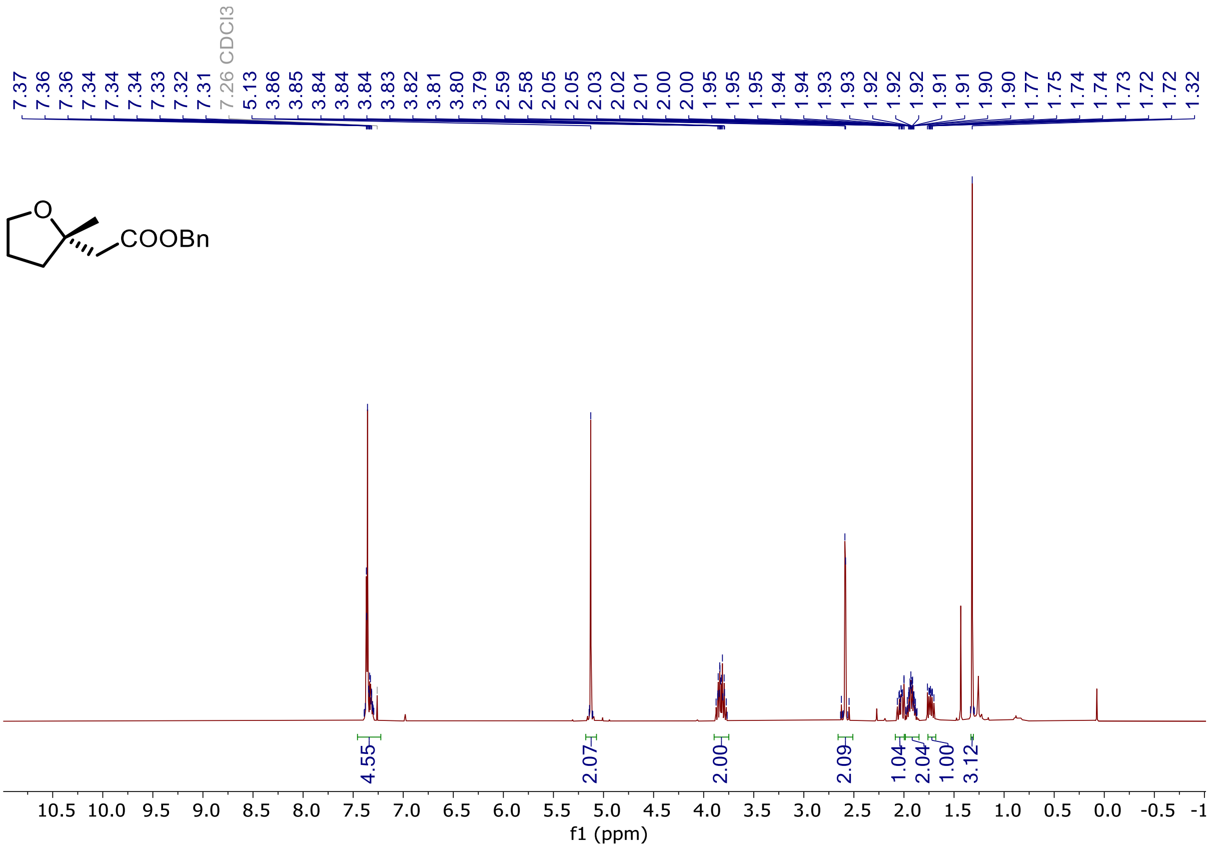

^{13}C NMR: (101 MHz, $CDCl_3$, 298K) of **4k**

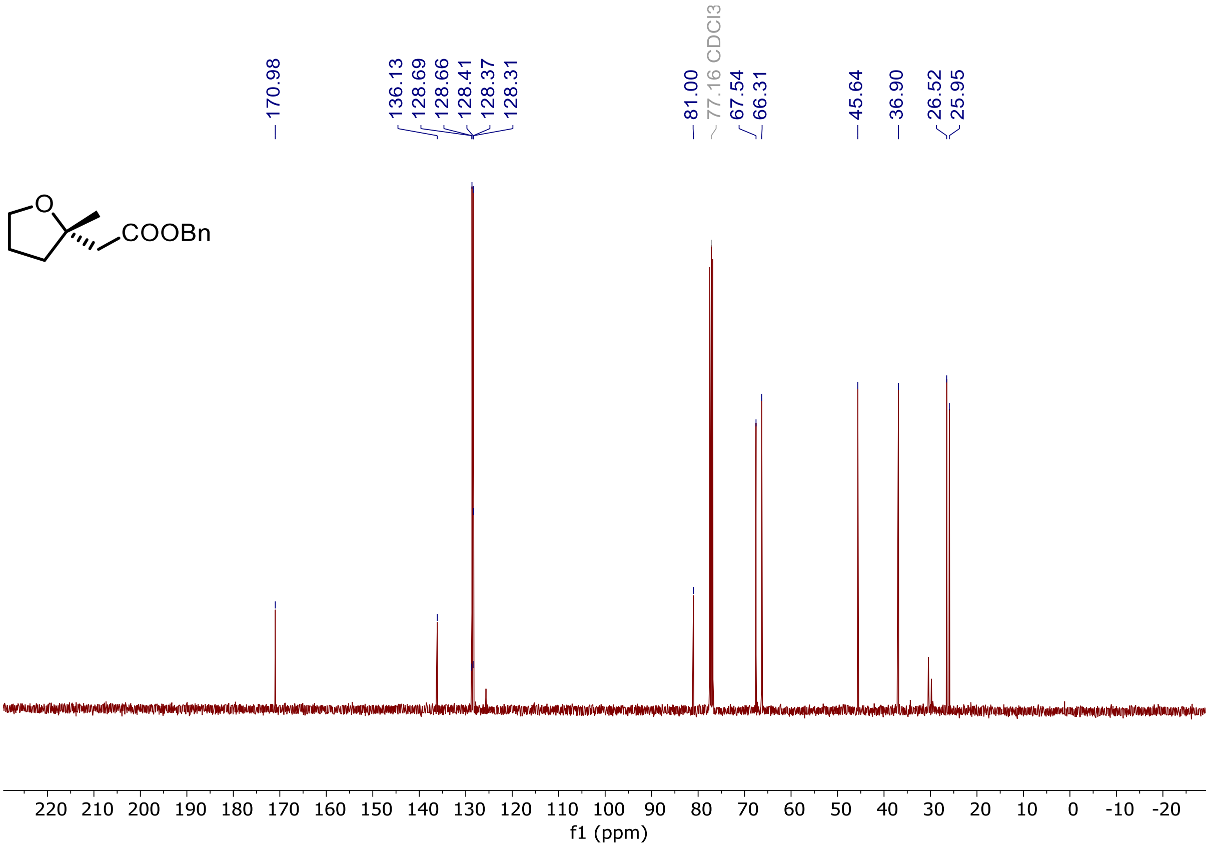

^{1}H NMR: (400 MHz, $CDCl_3$, 298K) of **41**

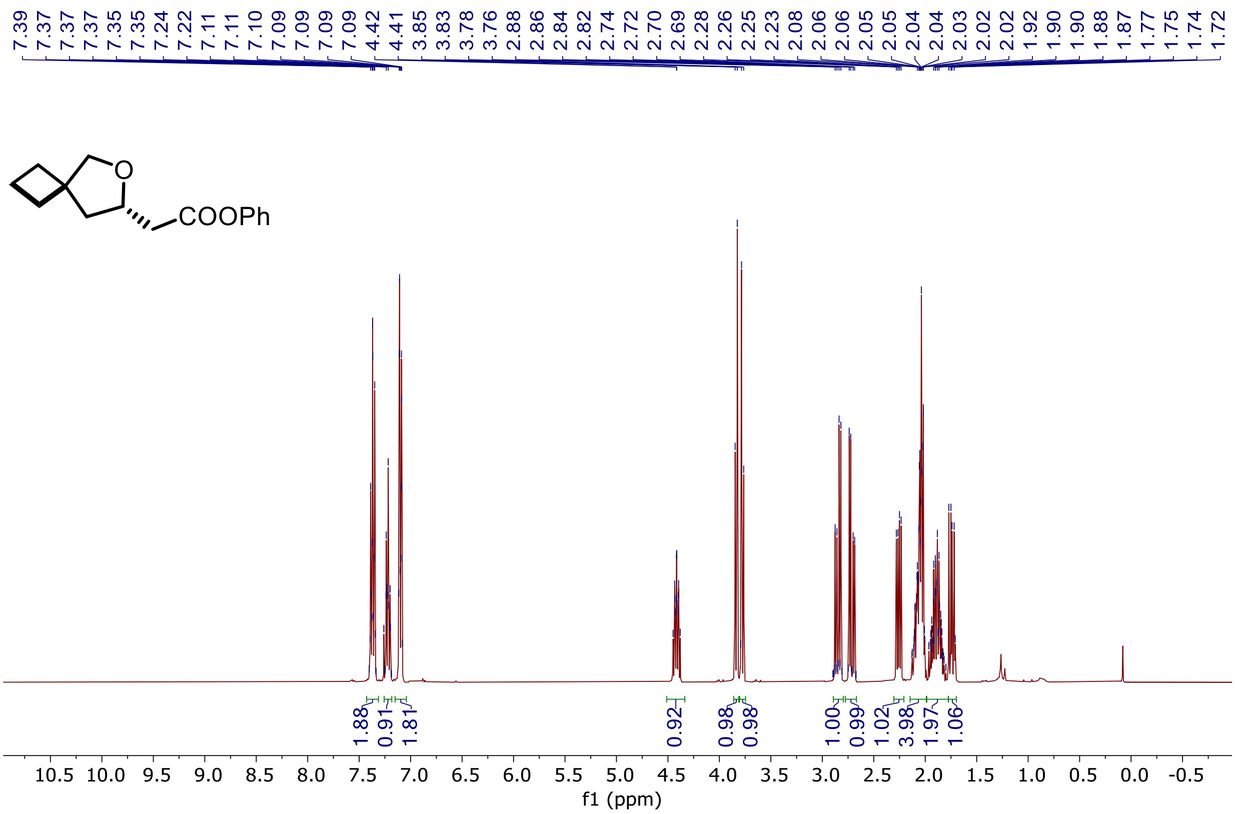

^{13}C NMR: (101 MHz, $CDCl_3$, 298K) of **41**

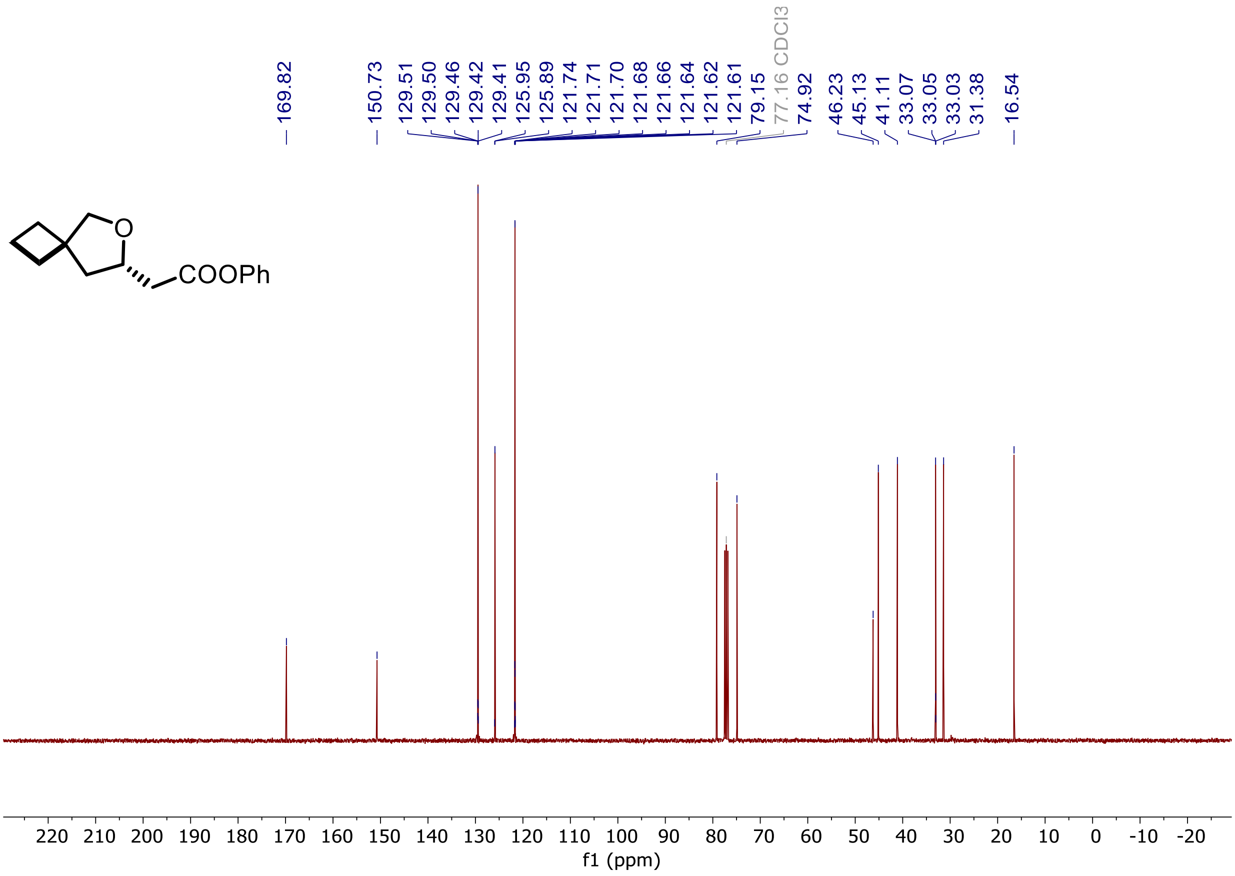

^{1}H NMR: (400 MHz, $CDCl_3$, 298K) of **4m**

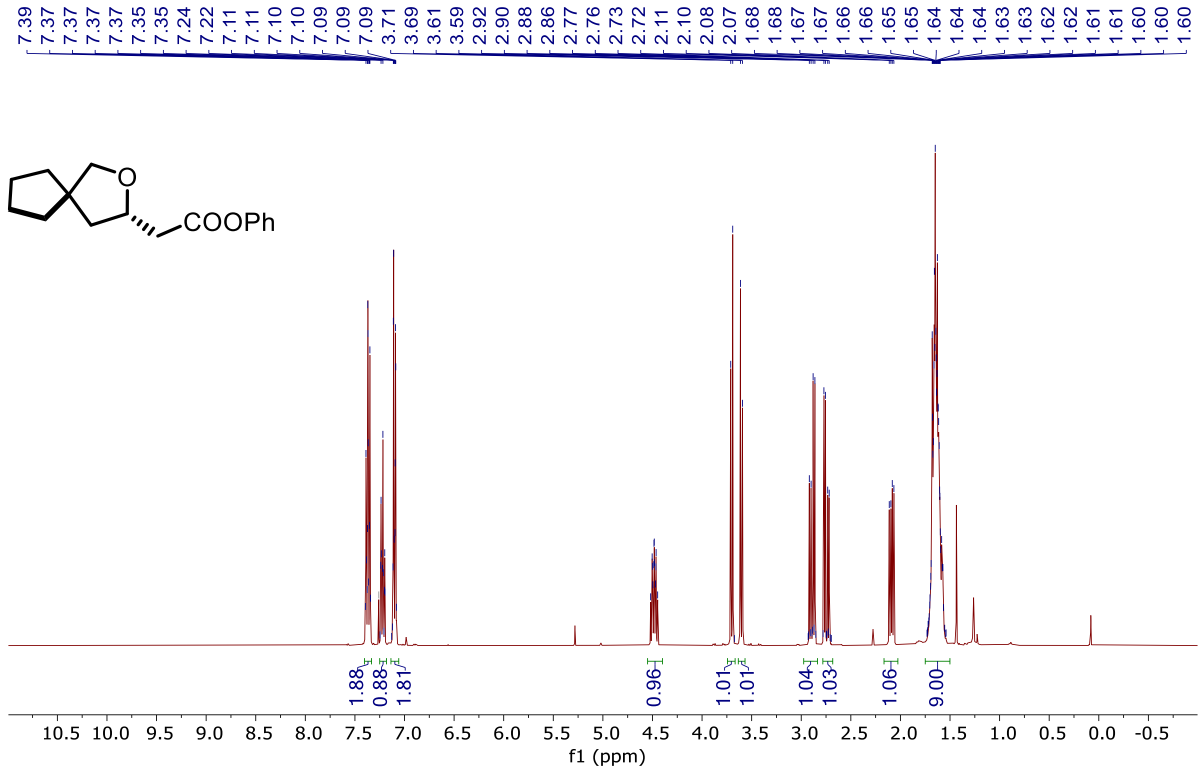

^{13}C NMR: (101 MHz, $CDCl_3$, 298K) of **4m**

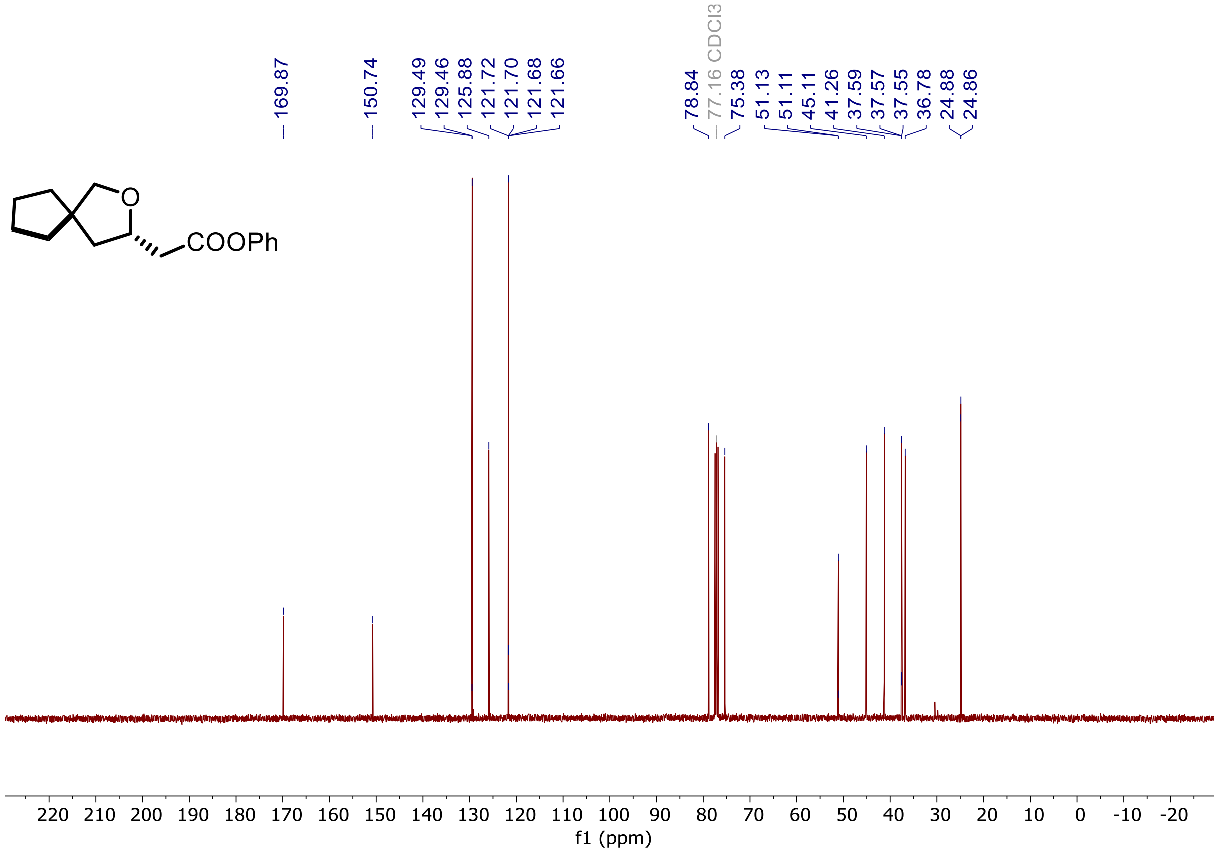

^{1}H NMR: (400 MHz, $CDCl_3$, 298K) of **4n**

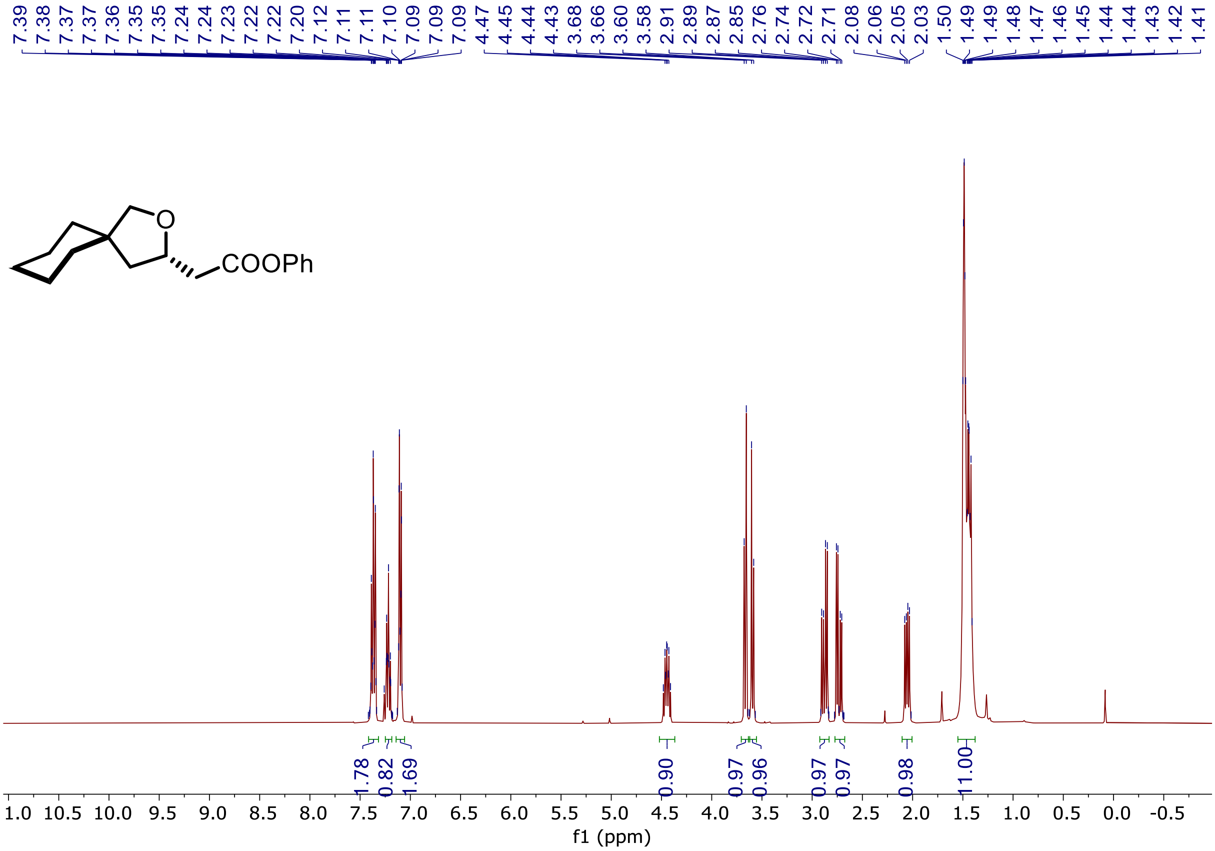

^{13}C NMR: (101 MHz, $CDCl_3$, 298K) of **4n**

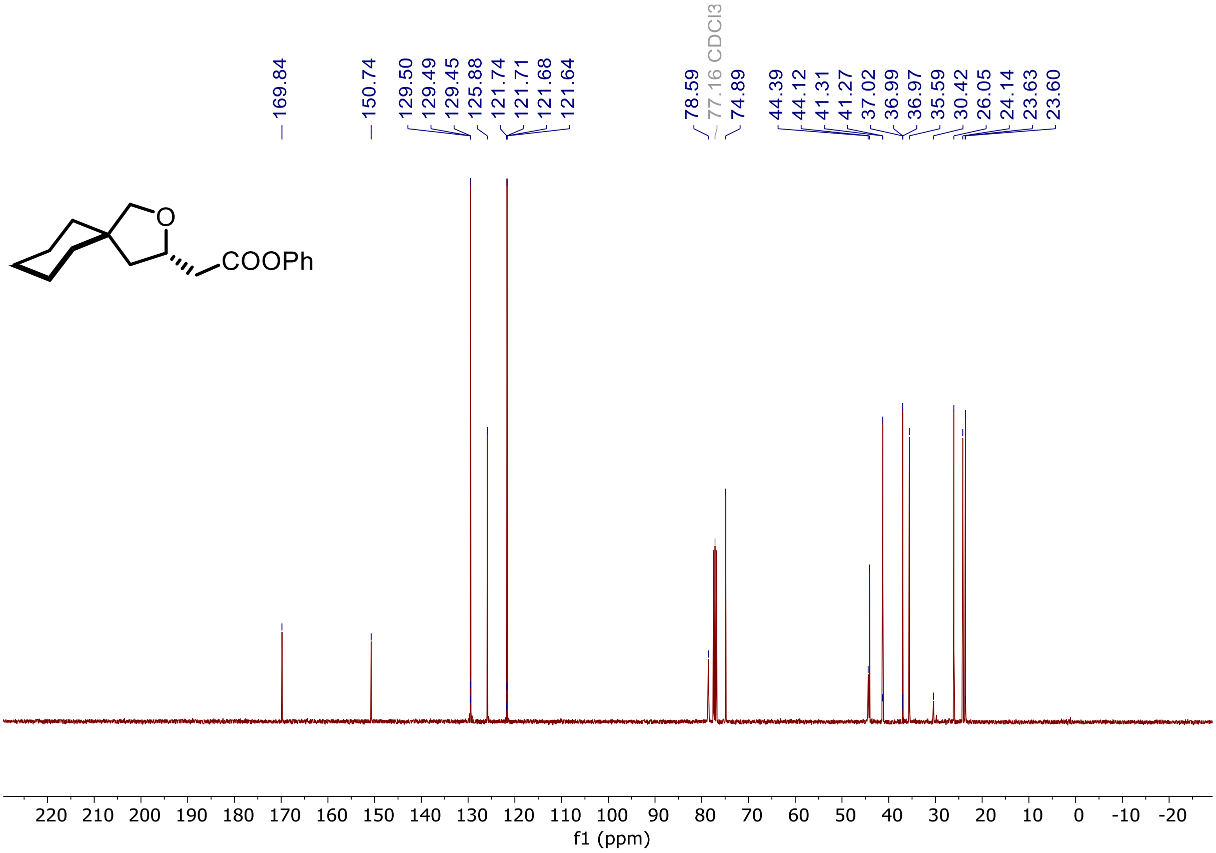

^{1}H NMR: (400 MHz, $CDCl_3$, 298K) of **4o**

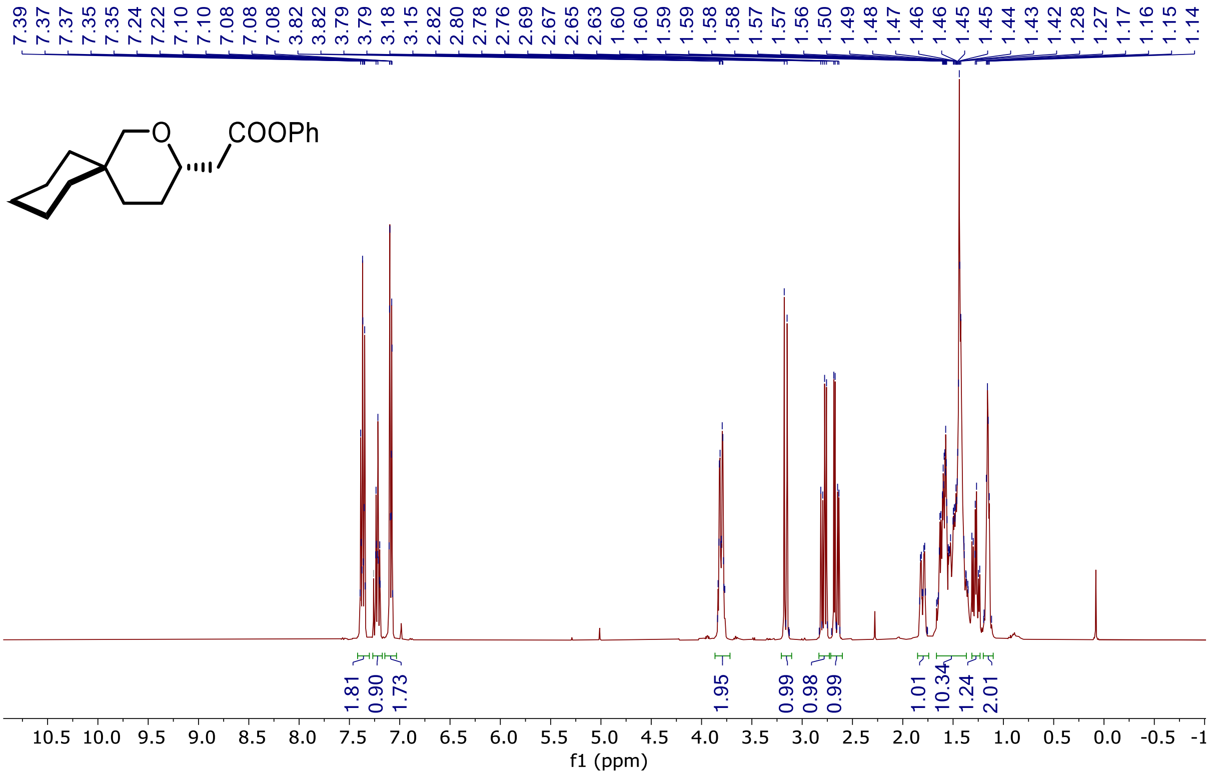

^{13}C NMR: (101 MHz, $CDCl_3$, 298K) of **4o**

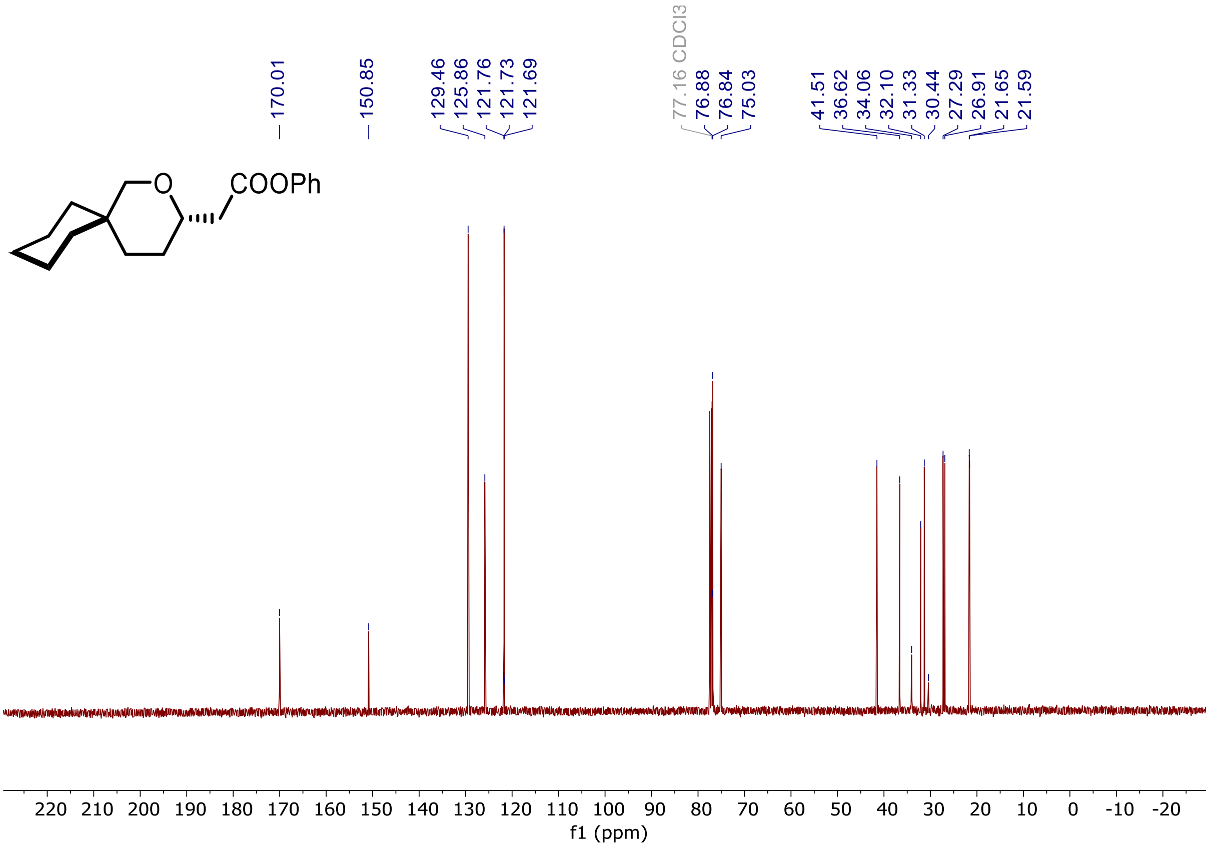

^{1}H NMR: (400 MHz, $CDCl_3$, 298K) of **4p**

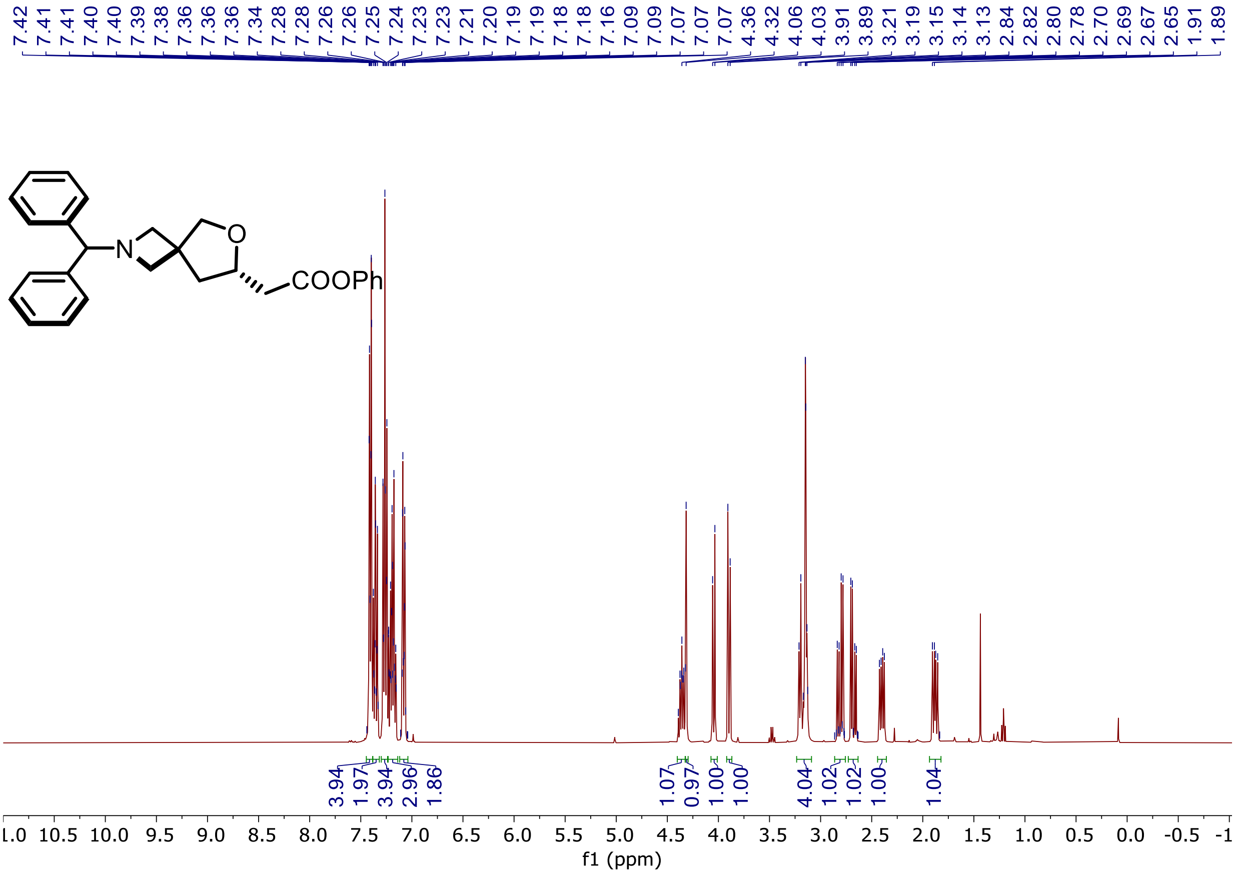

^{13}C NMR: (101 MHz, $CDCl_3$, 298K) of **4p**

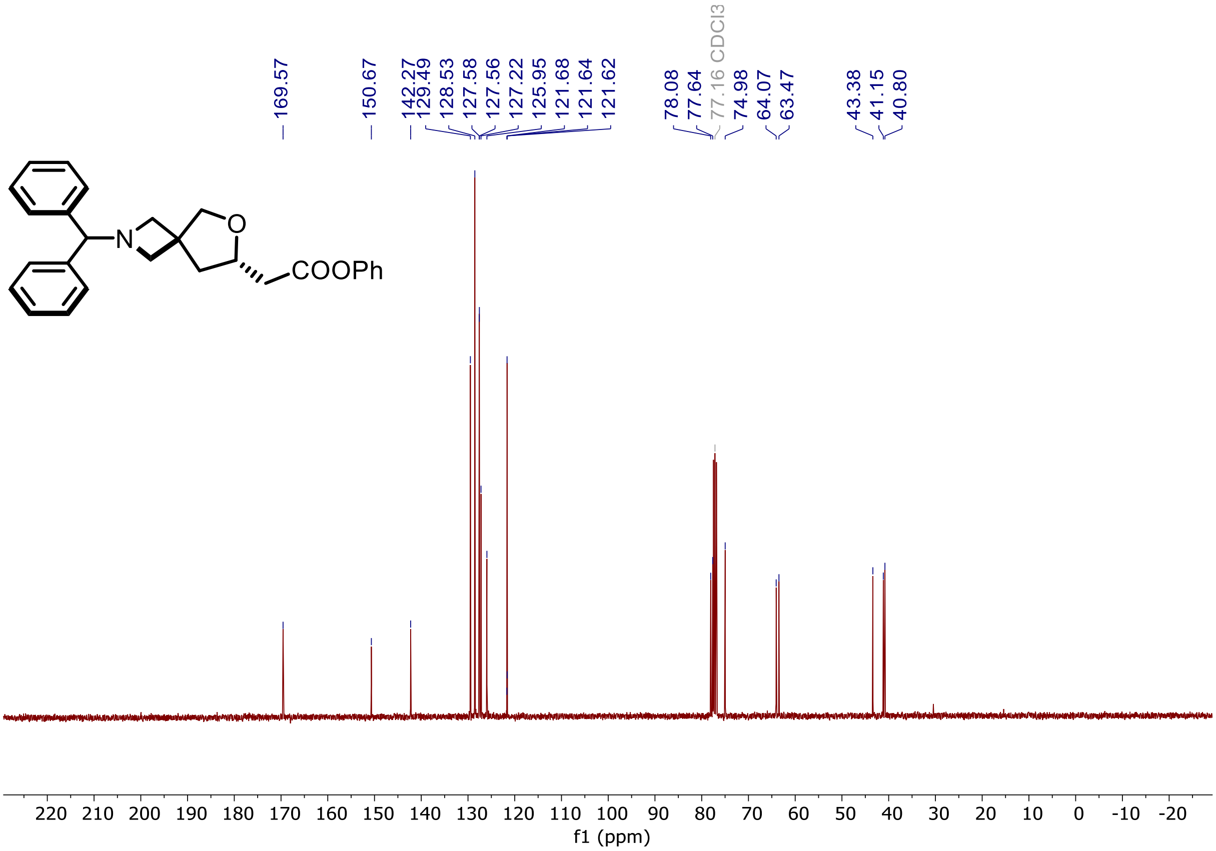

^{1}H NMR: (400 MHz, $CDCl_3$, 298K) of **4q**

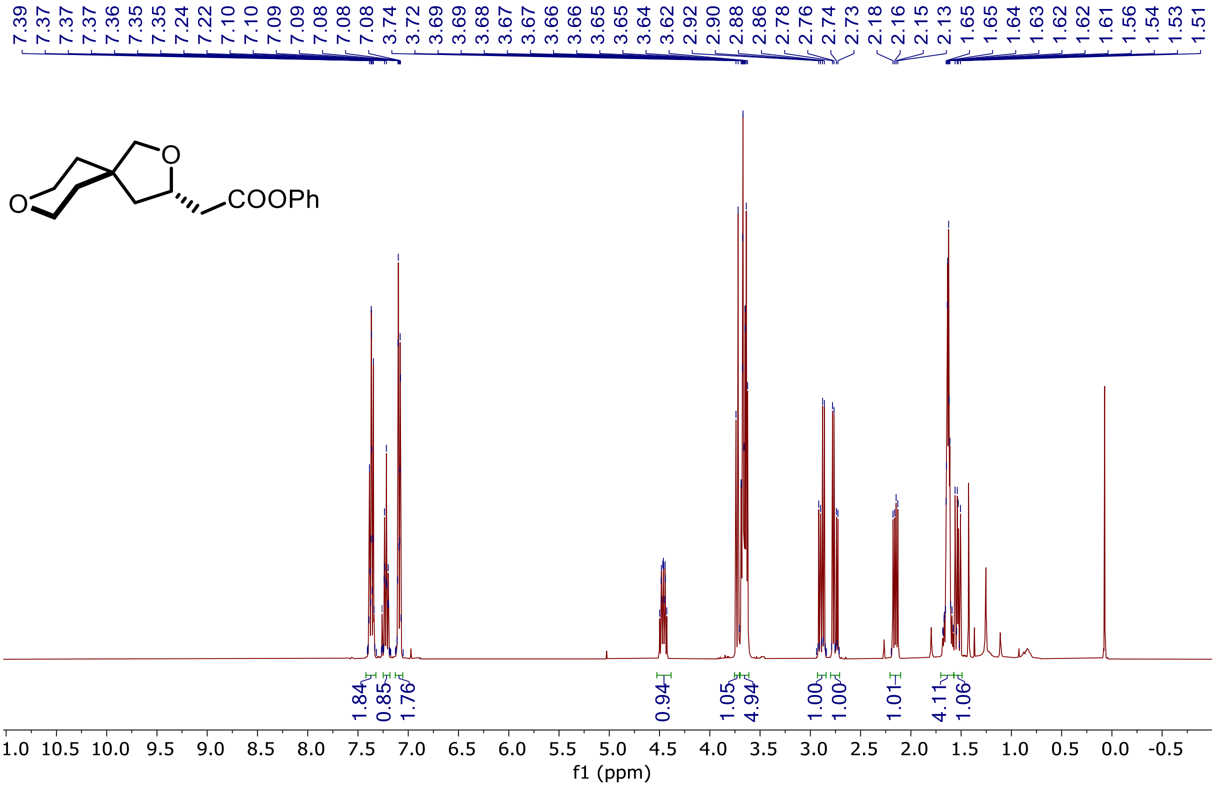

^{13}C NMR: (101 MHz, $CDCl_3$, 298K) of **4q**

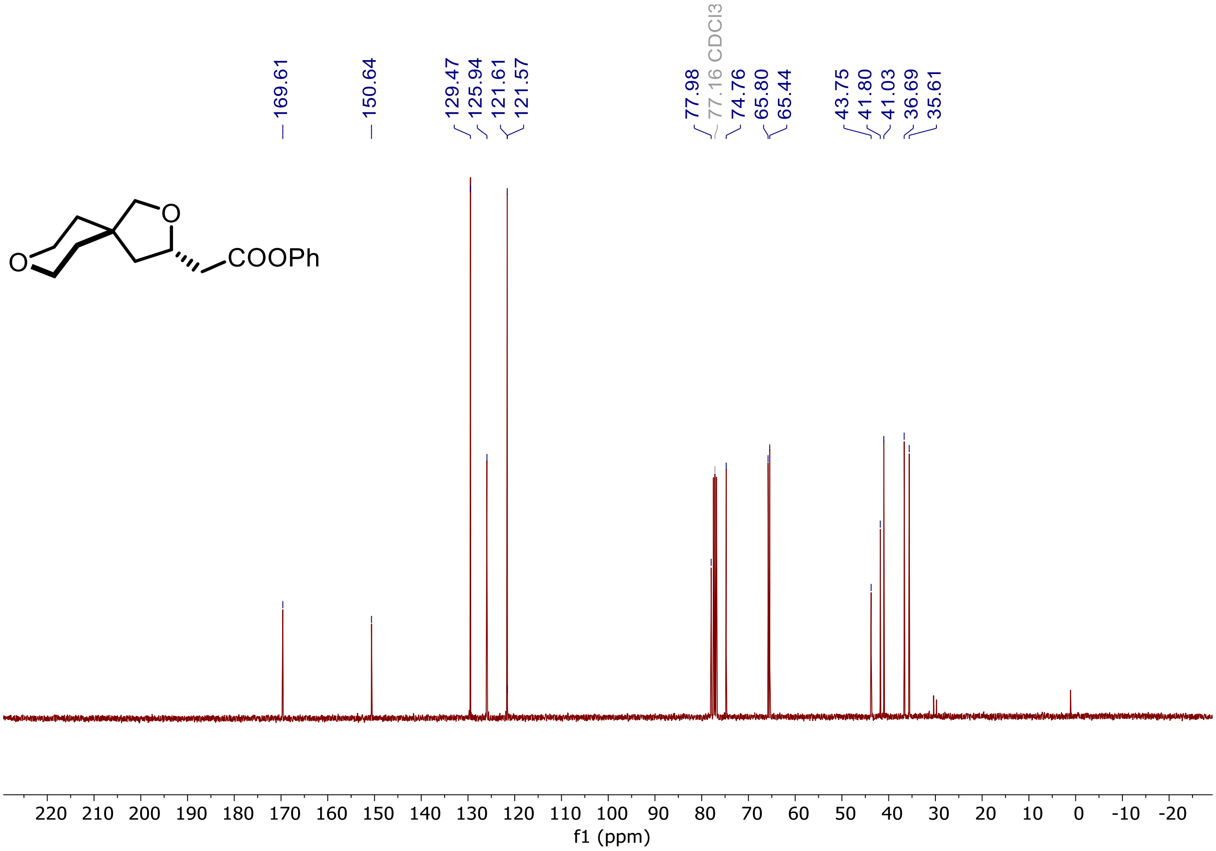

^{1}H NMR: (400 MHz, $CDCl_3$, 298K) of **4r**

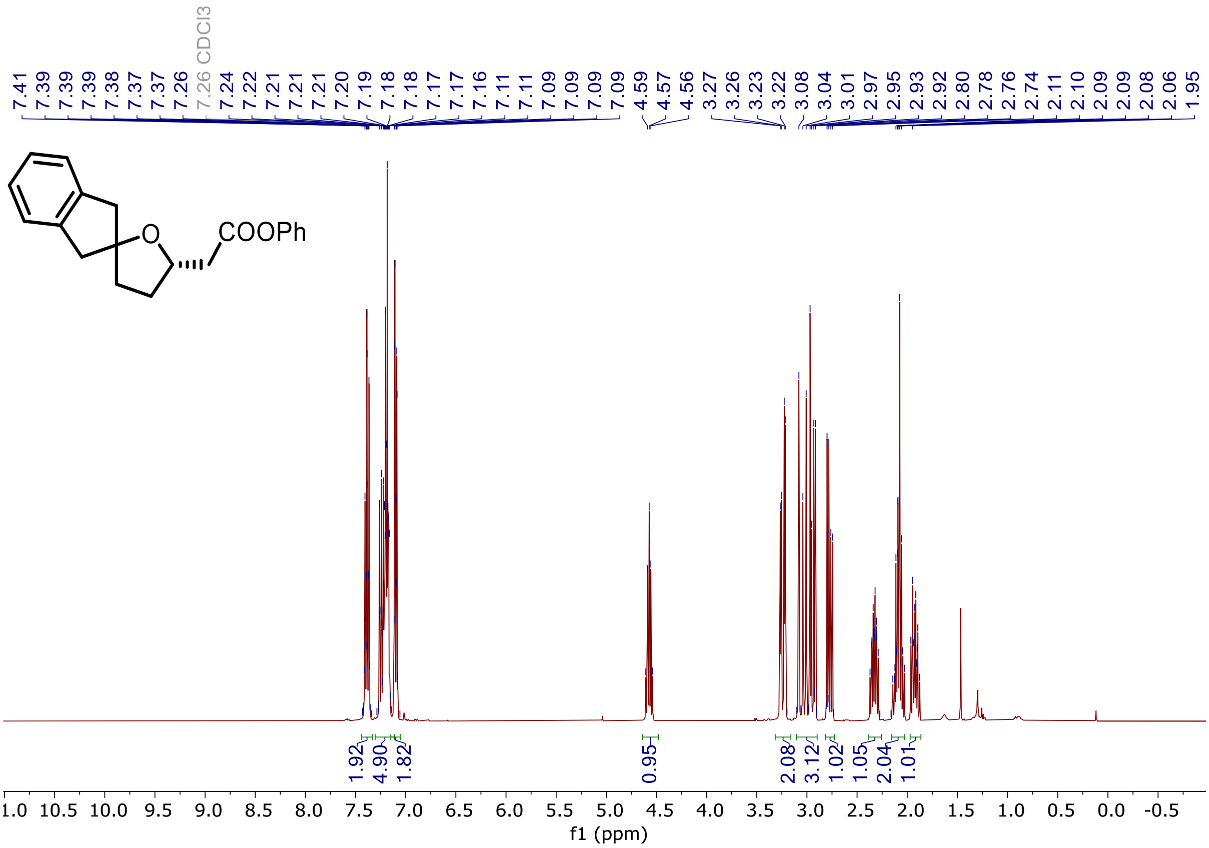

^{13}C NMR: (101 MHz, $CDCl_3$, 298K) of **4r**

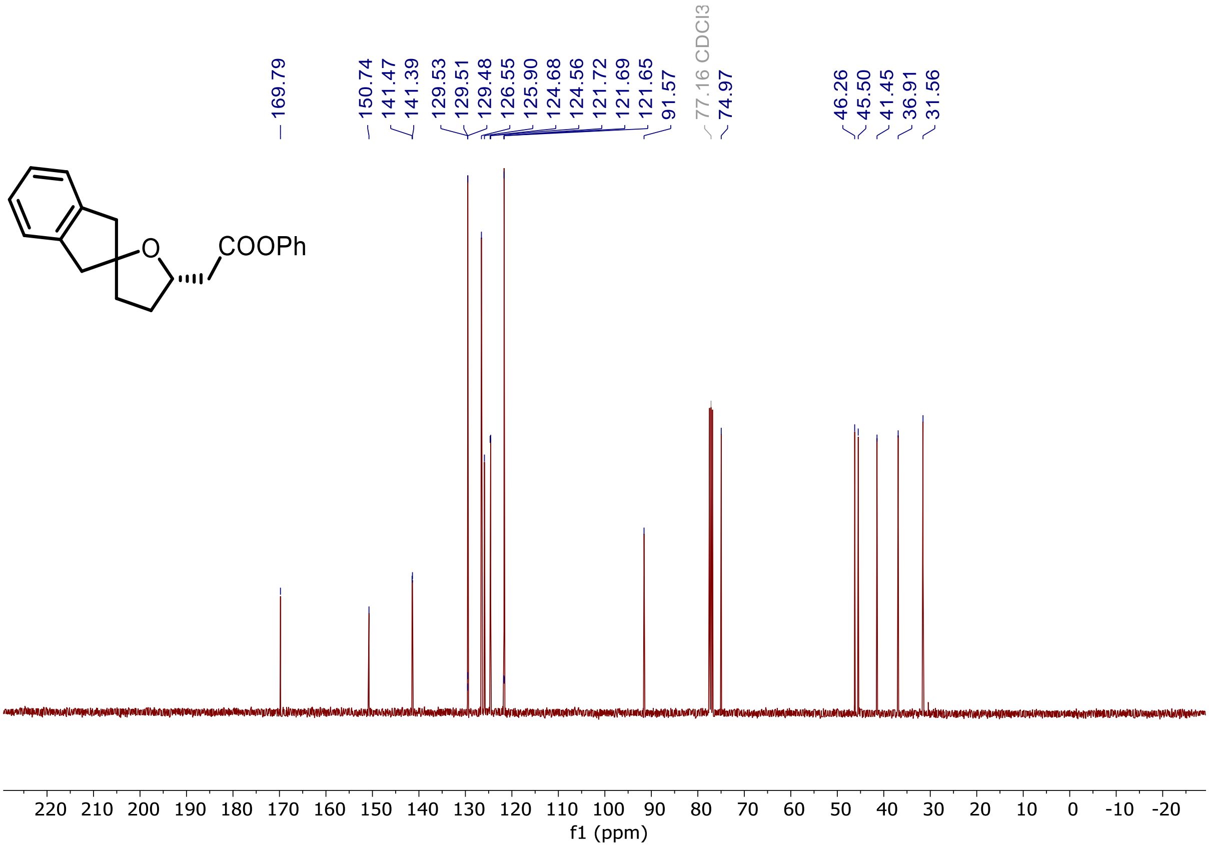

^{1}H NMR: (400 MHz, $CDCl_3$, 298K) of **4s**

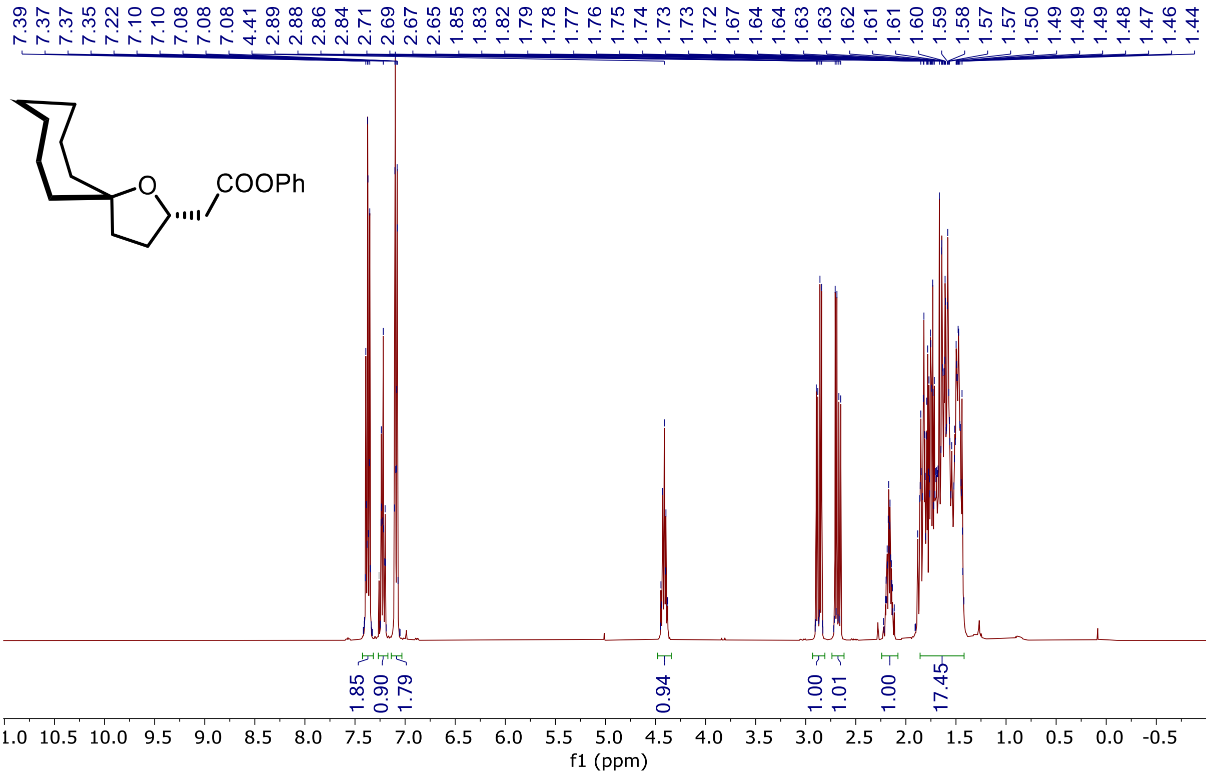

^{13}C NMR: (101 MHz, $CDCl_3$, 298K) of **4s**

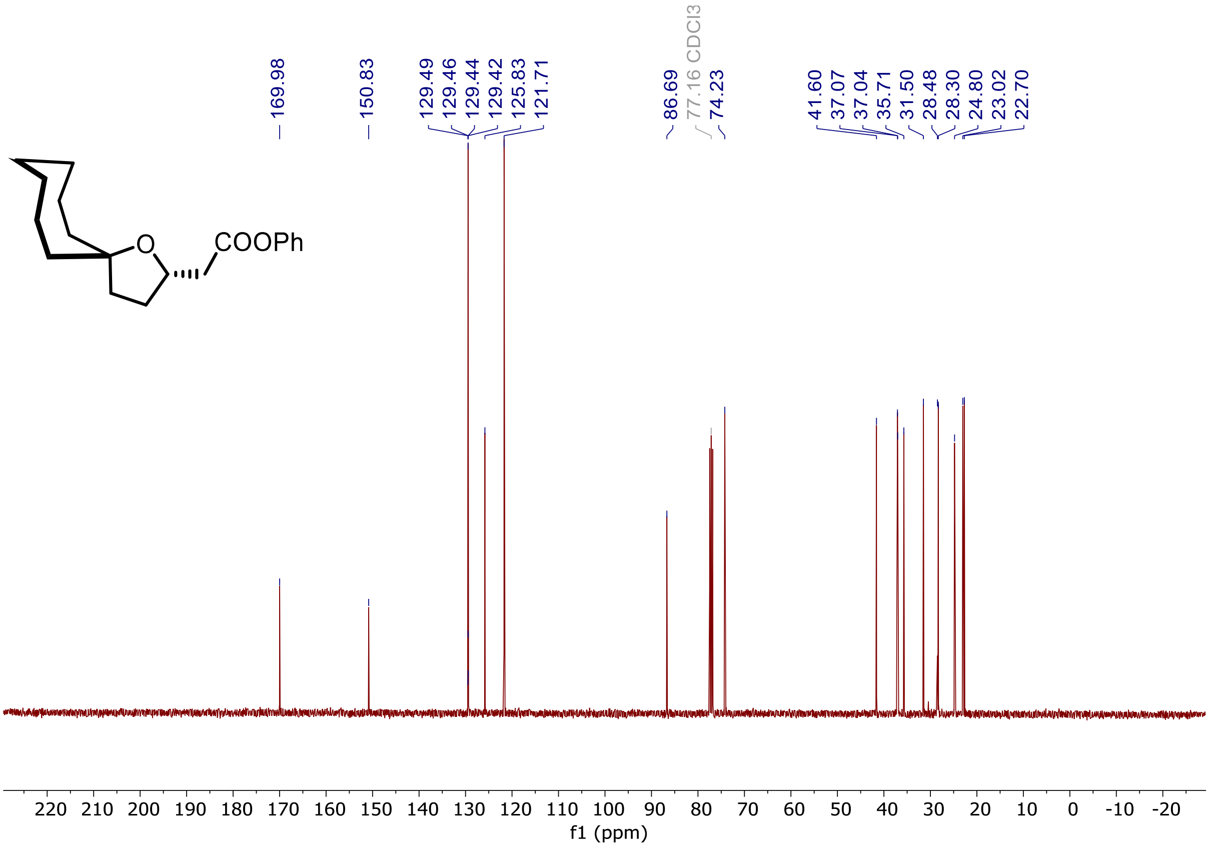

^{1}H NMR: (400 MHz, $CDCl_3$, 298K) of **4t**

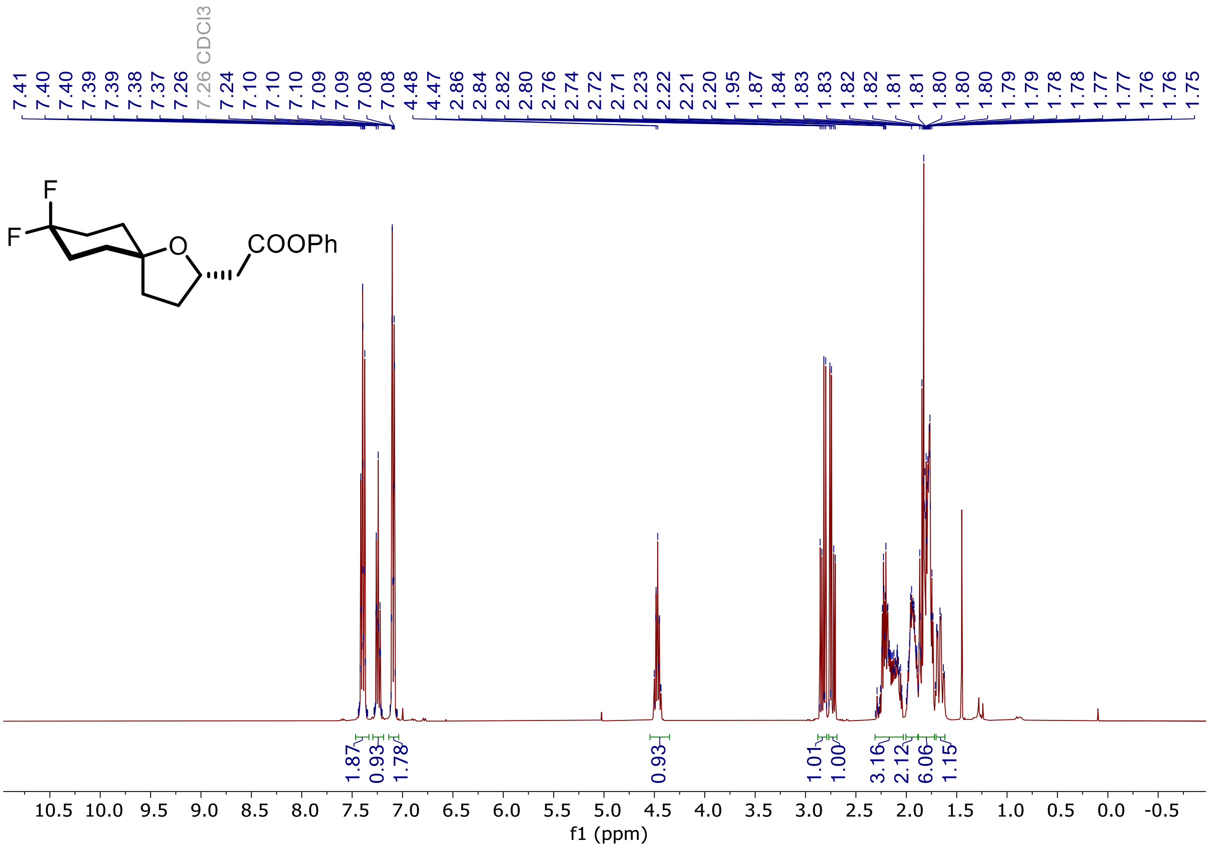

^{13}C NMR: (101 MHz, $CDCl_3$, 298K) of **4t**

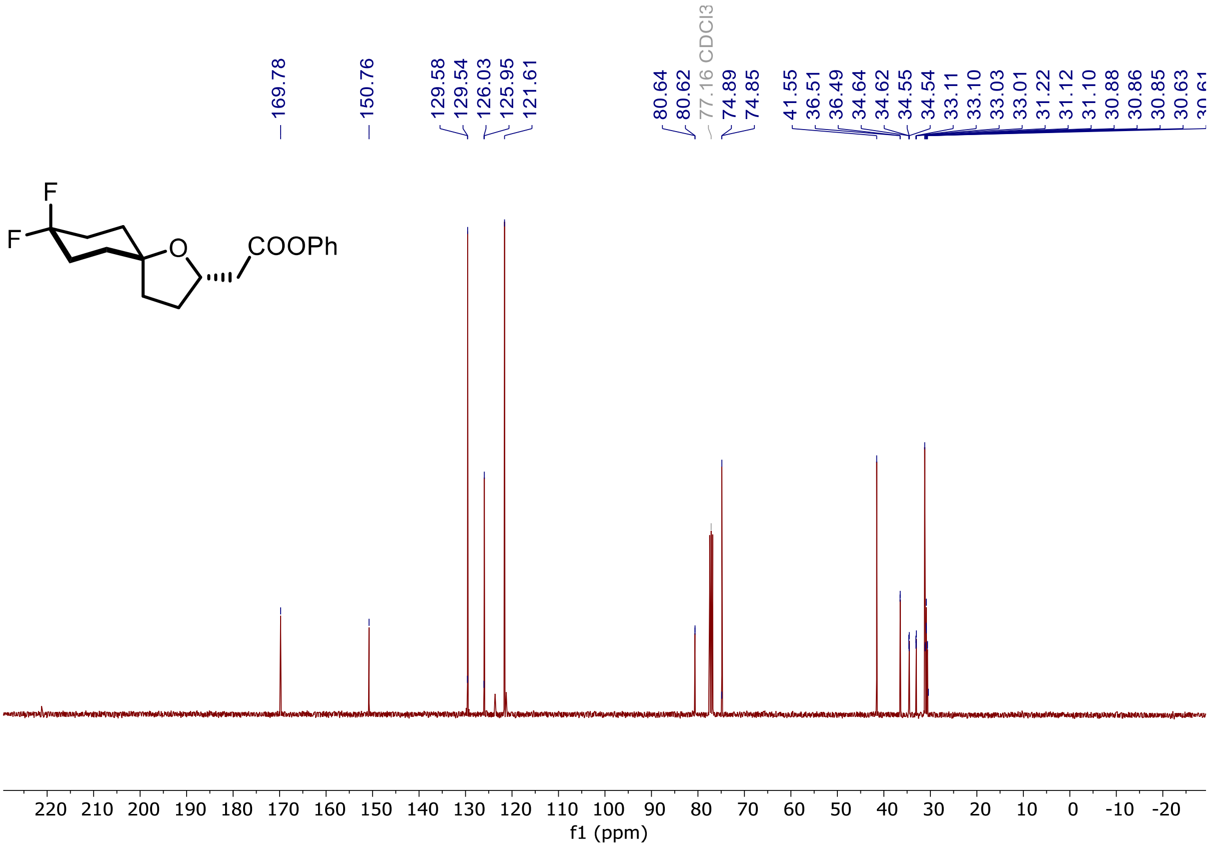

^{19}F NMR: (377 MHz, $CDCl_3$, 298K) of **4t**

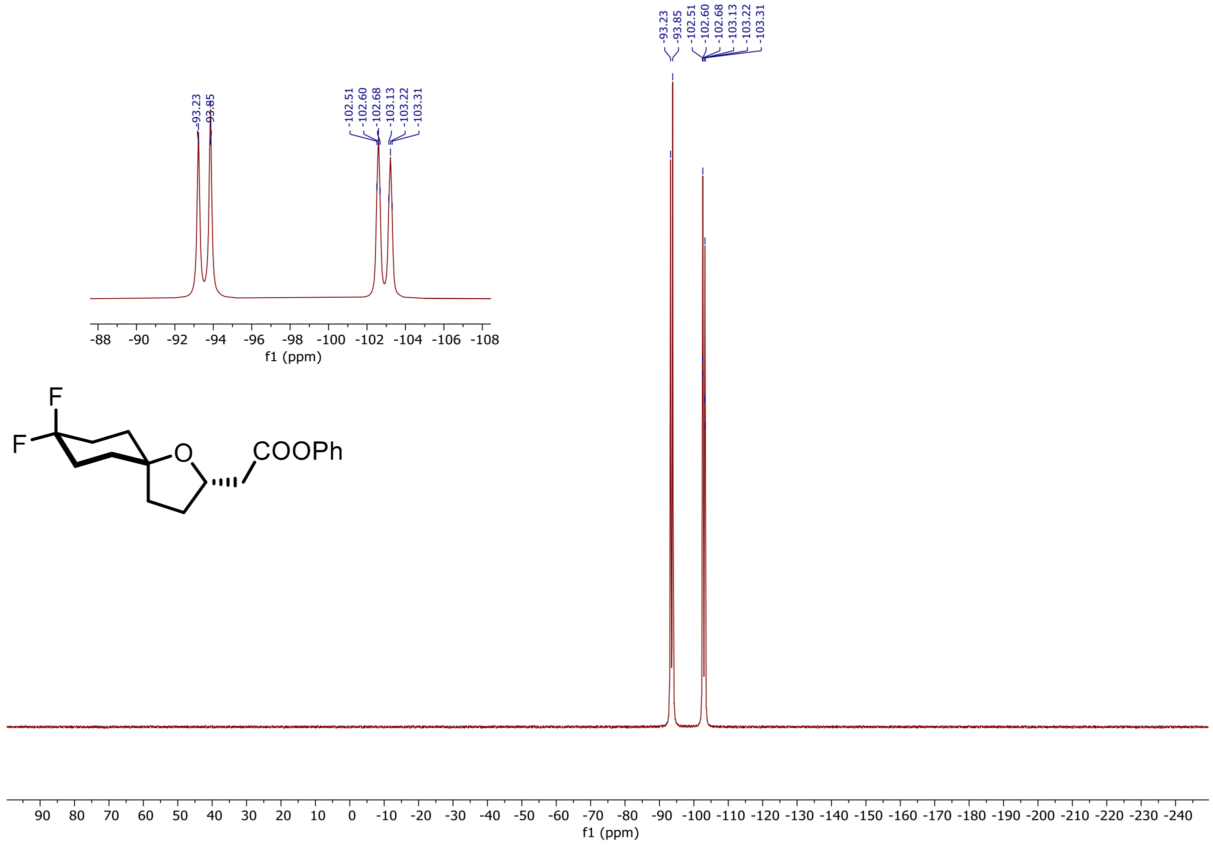

^{1}H NMR: (400 MHz, $CDCl_3$, 298K) of **4u**

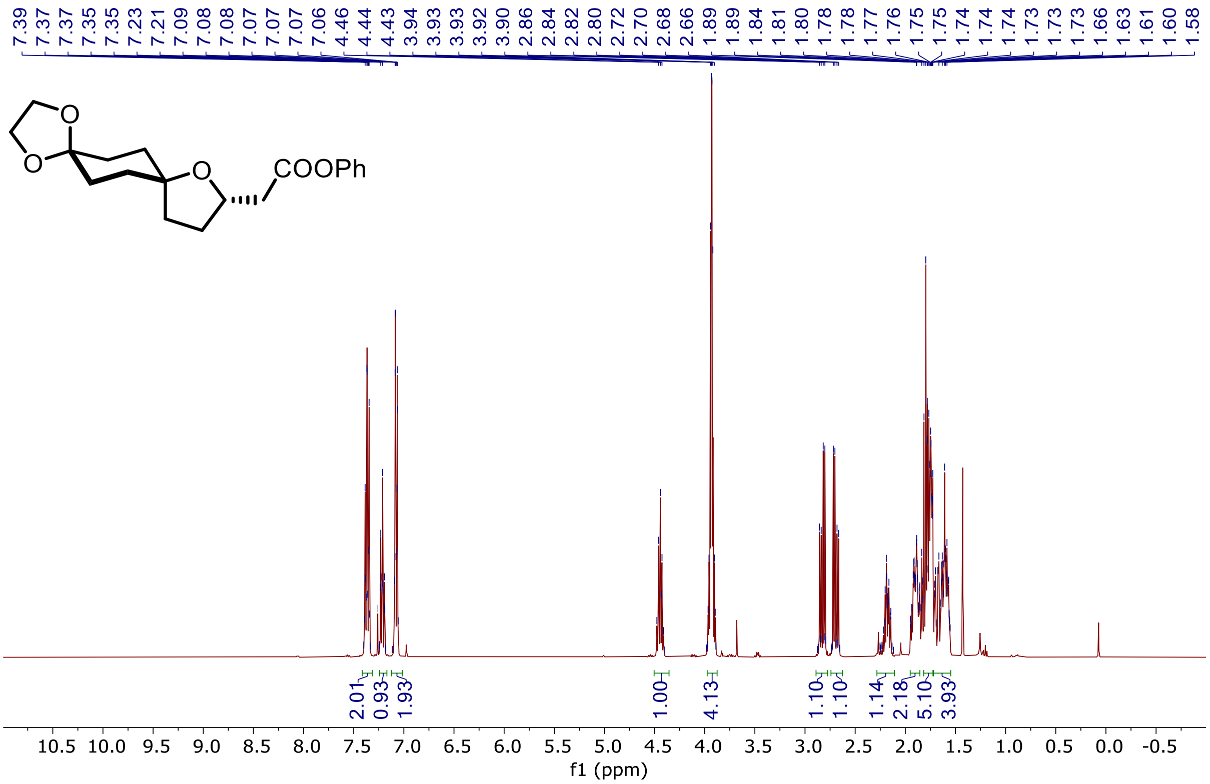

^{13}C NMR: (101 MHz, $CDCl_3$, 298K) of **4u**

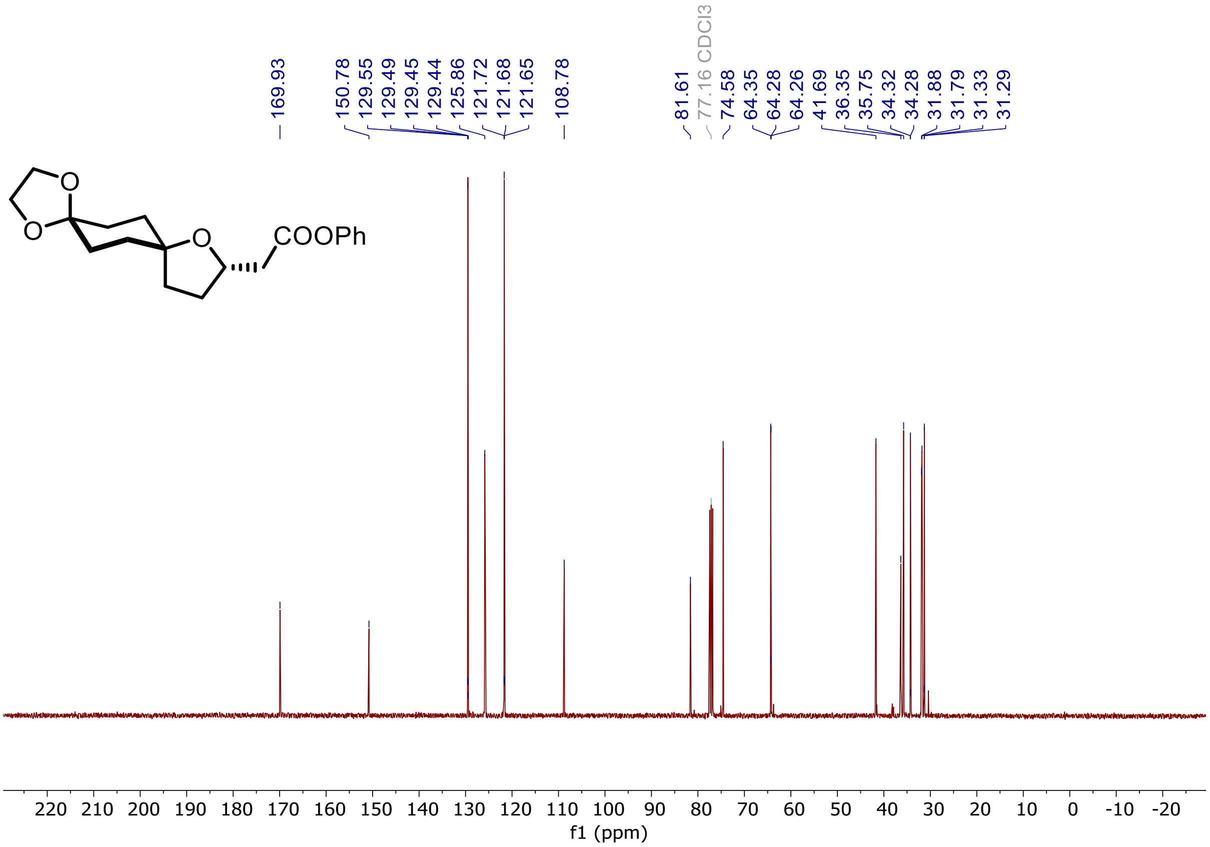

^{1}H NMR: (400 MHz, $CDCl_3$, 298K) of **4v**

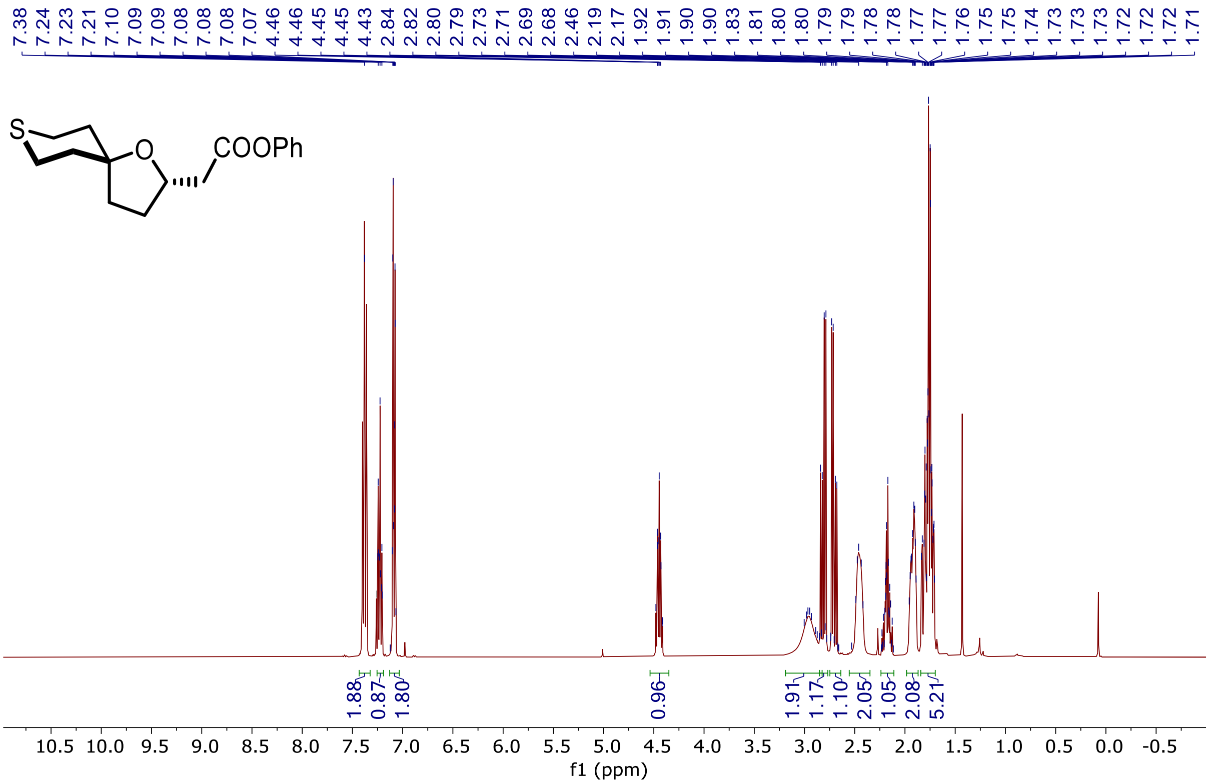

^{13}C NMR: (101 MHz, $CDCl_3$, 298K) of **4v**

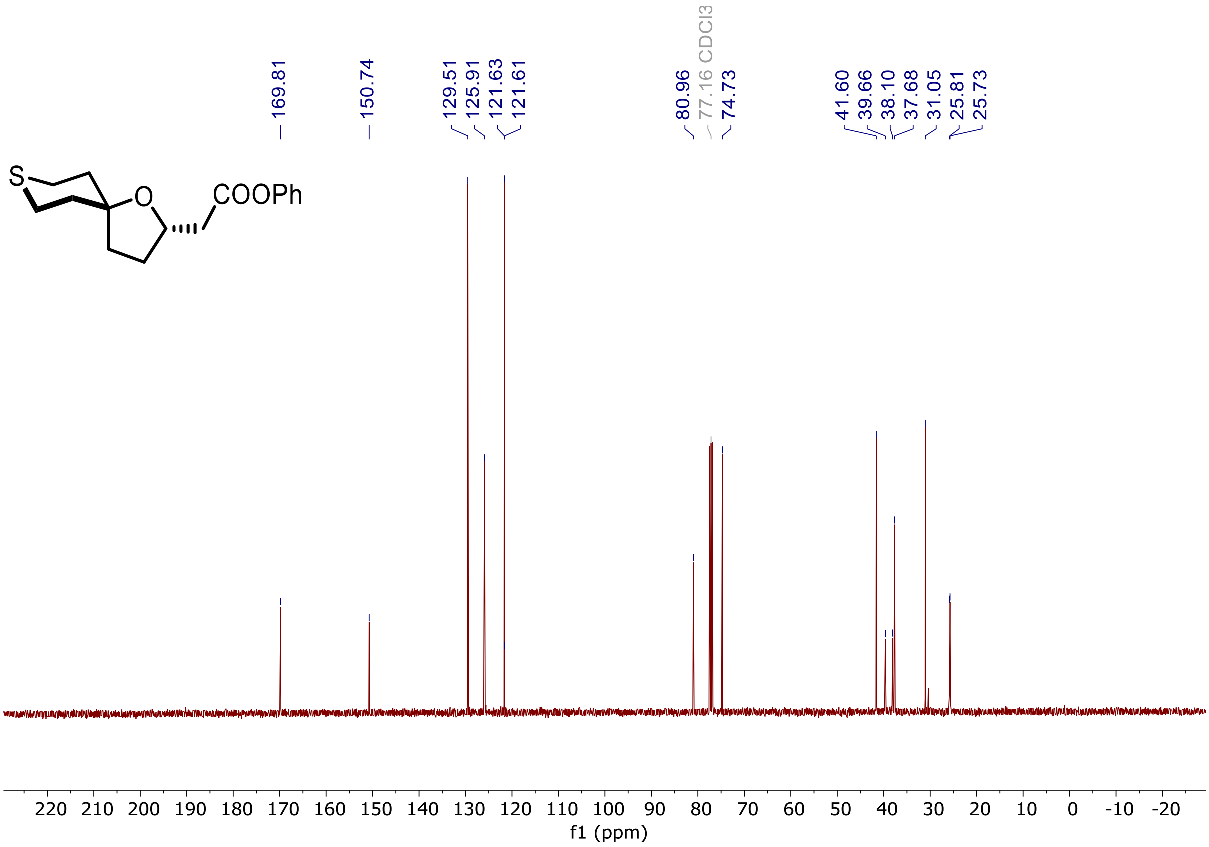

^{1}H NMR: (400 MHz, $CDCl_3$, 298K) of **4w**

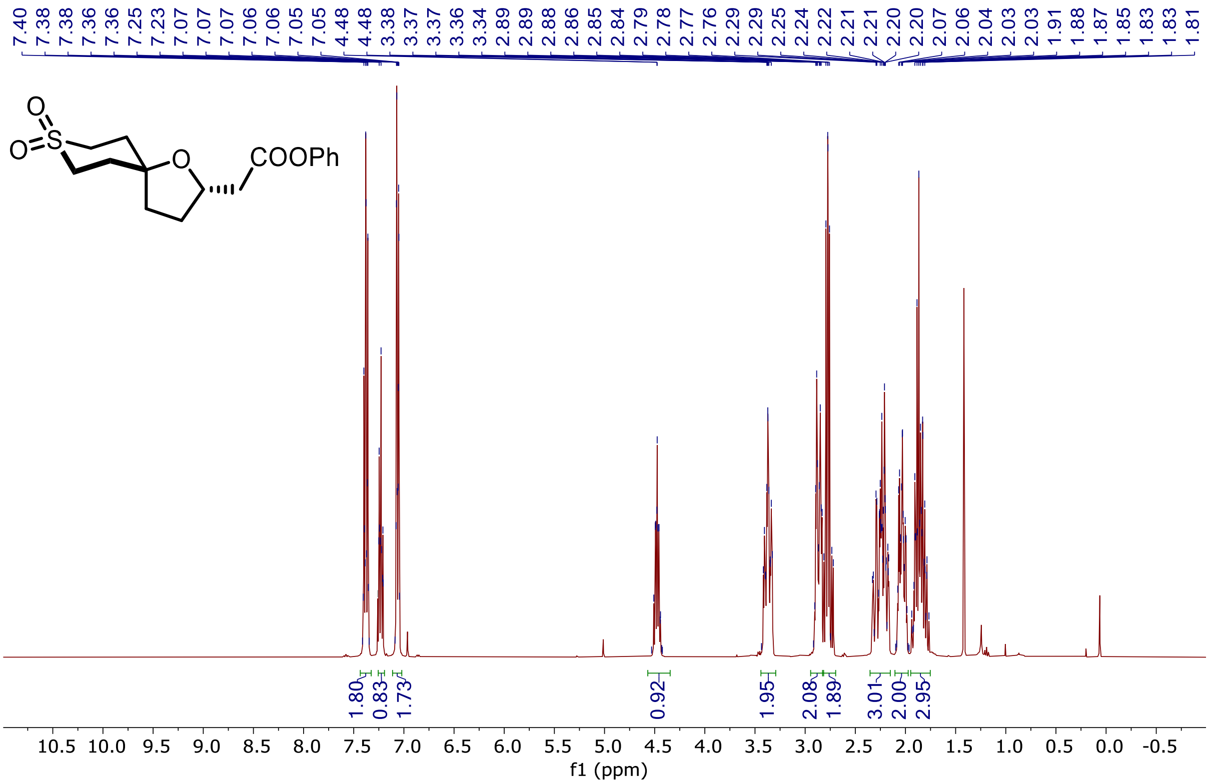

^{13}C NMR: (101 MHz, $CDCl_3$, 298K) of **4w**

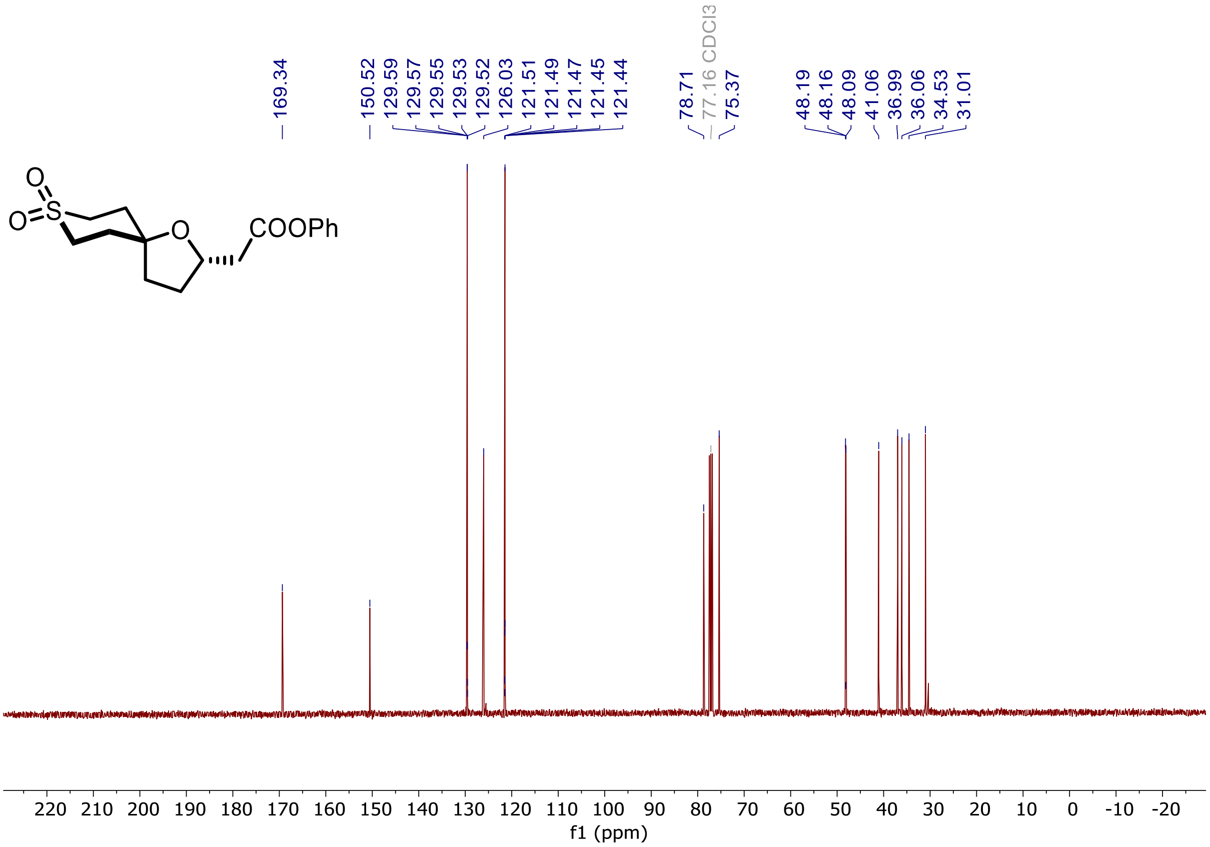

^{1}H NMR: (400 MHz, $CDCl_3$, 298K) of **4x**

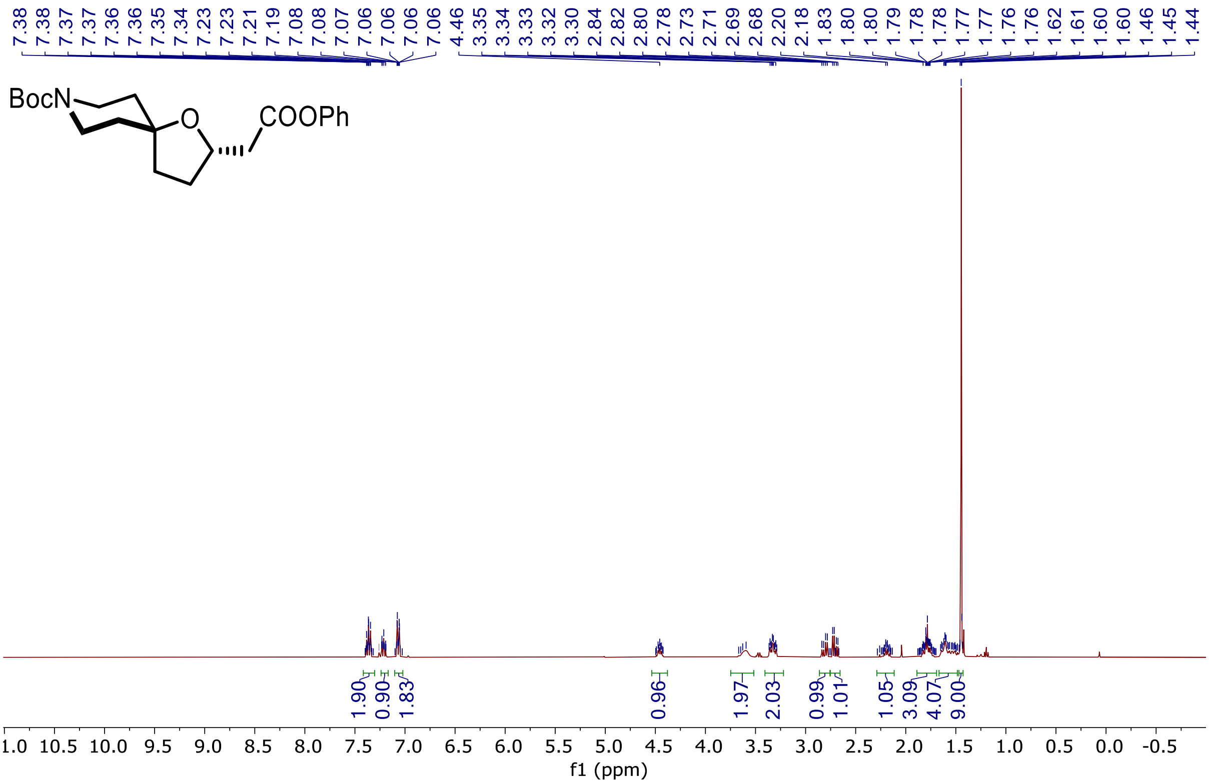

^{13}C NMR: (101 MHz, $CDCl_3$, 298K) of **4x**

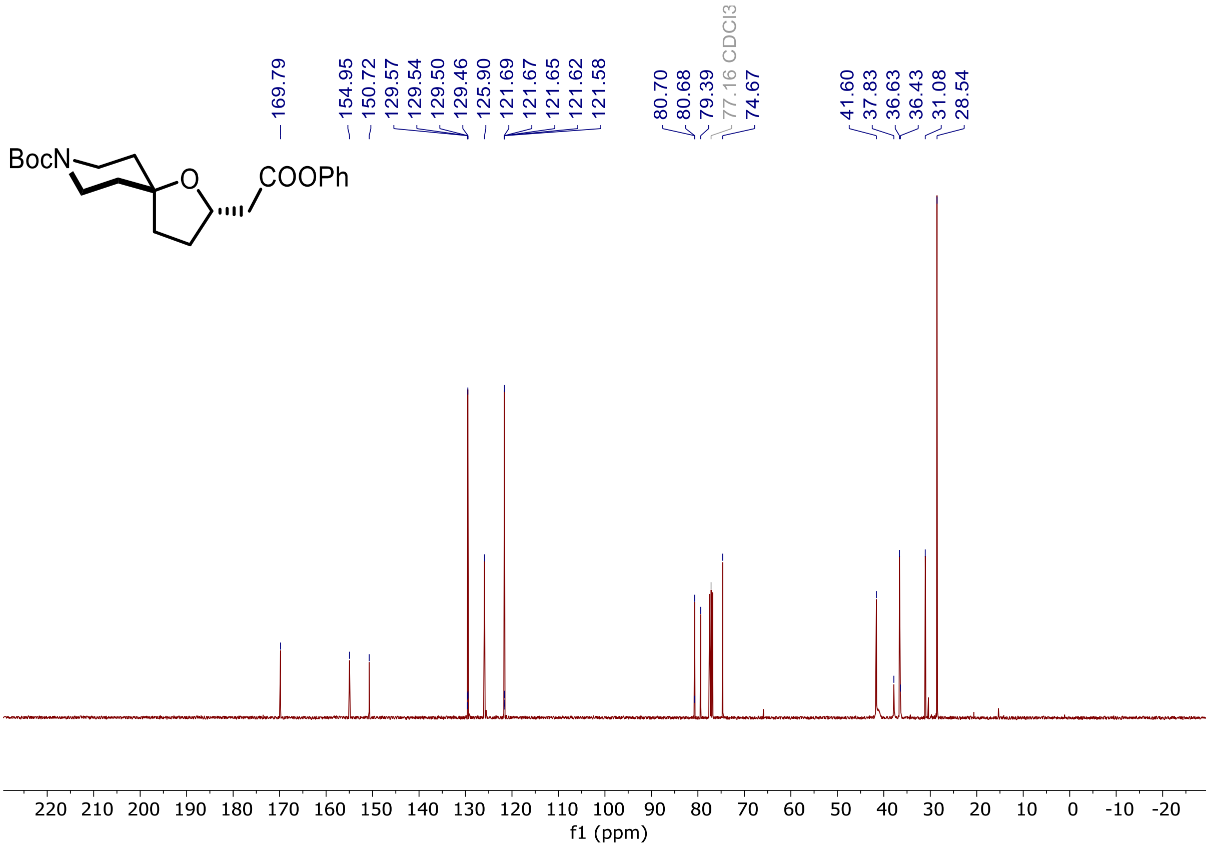

^{1}H NMR: (400 MHz, $CDCl_3$, 298K) of **4y**

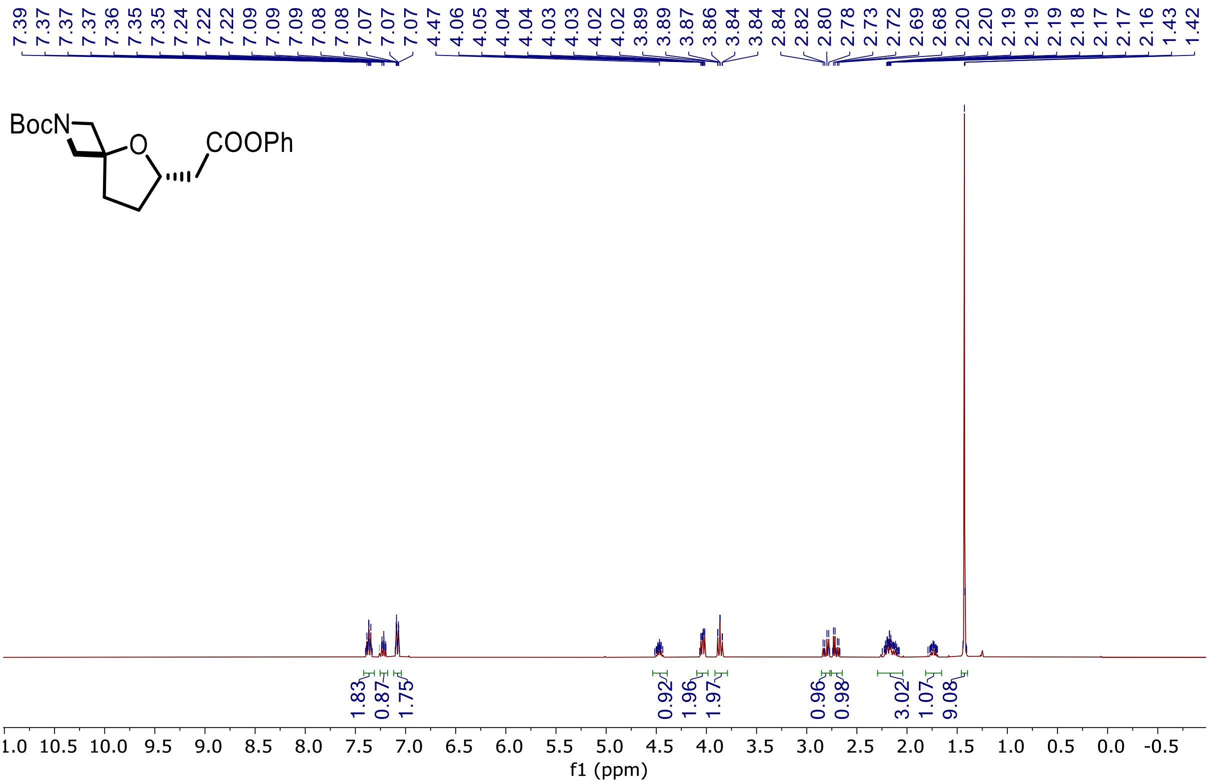

^{13}C NMR: (101 MHz, $CDCl_3$, 298K) of **4y**

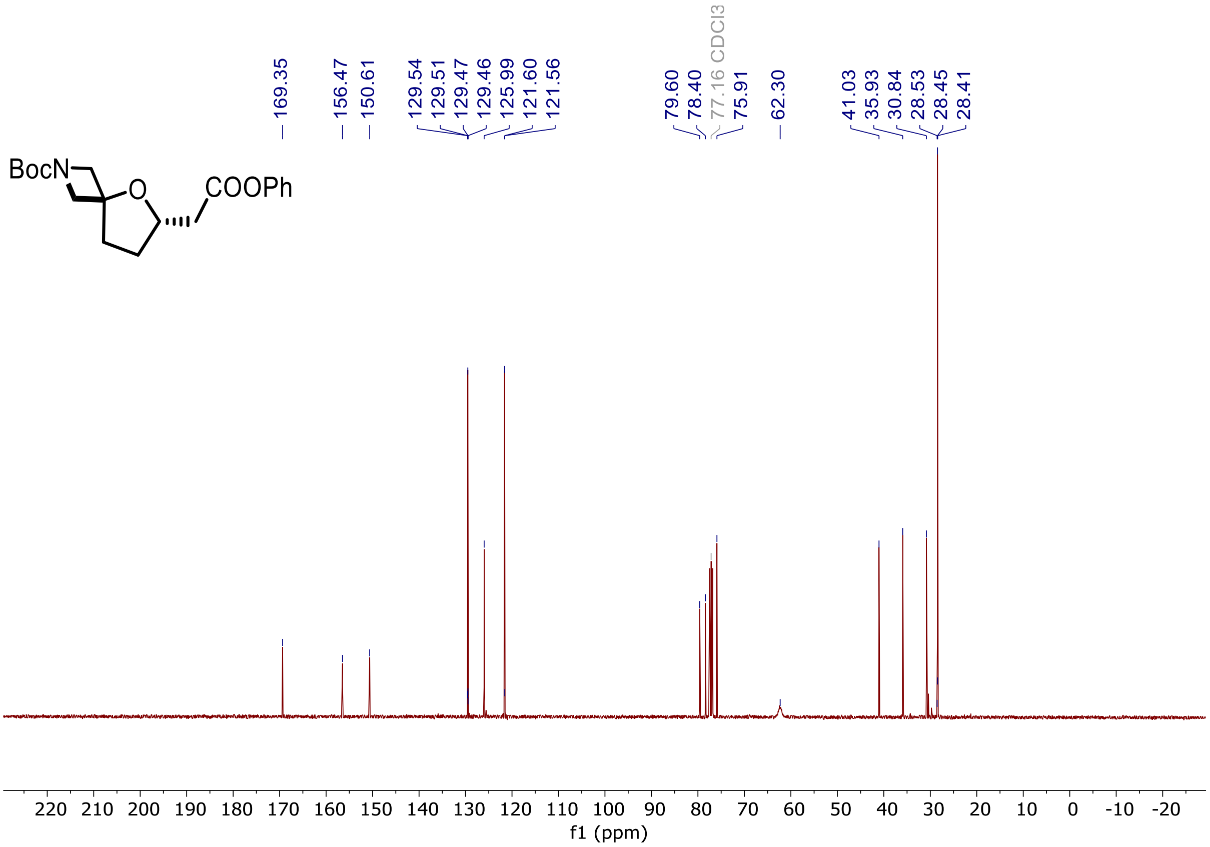

^{1}H NMR: (400 MHz, $CDCl_3$, 298K) of **4z**

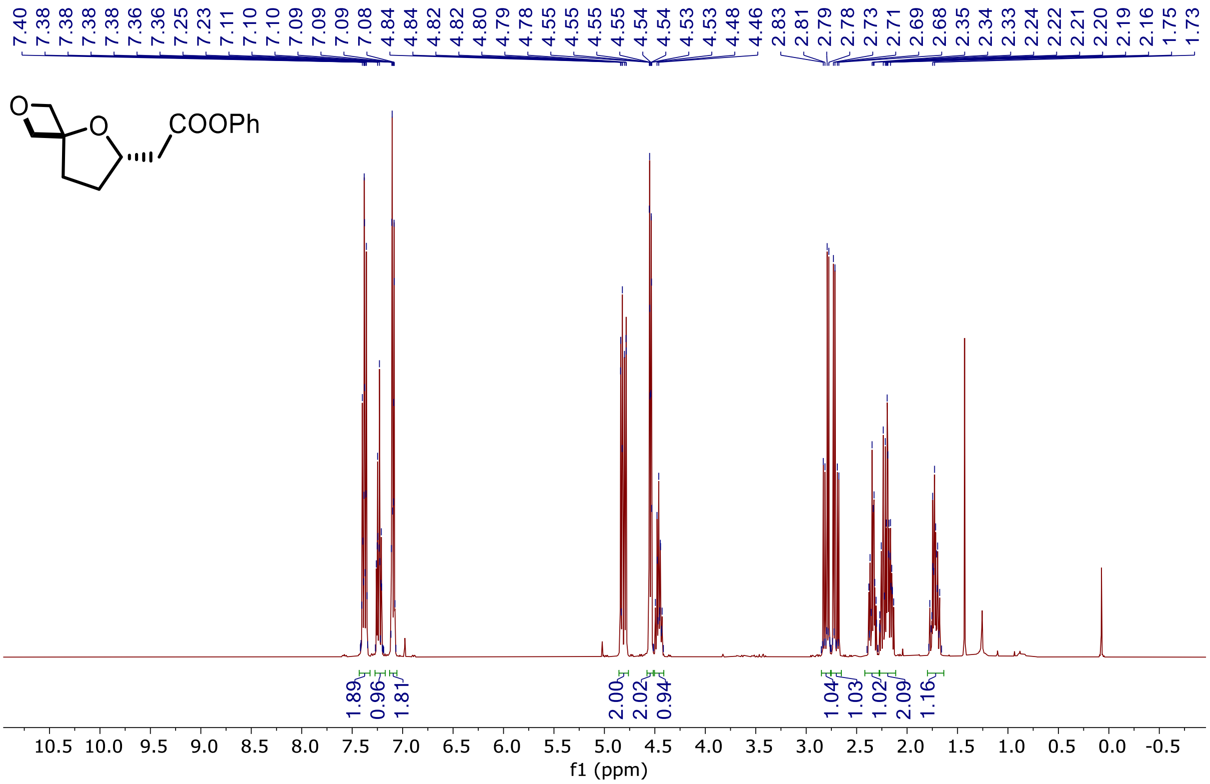

^{13}C NMR: (101 MHz, $CDCl_3$, 298K) of **4z**

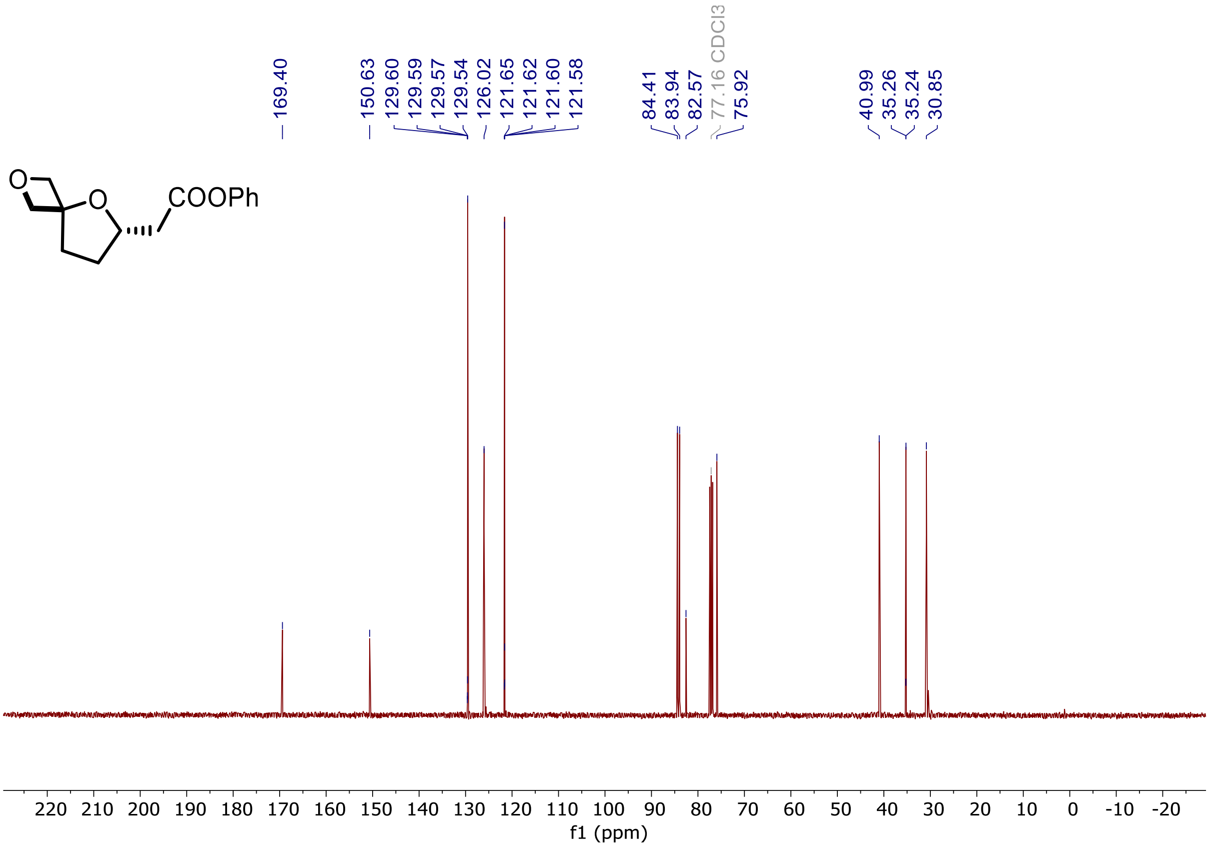

^{1}H NMR: (400 MHz, $CDCl_3$, 298K) of **4aa**

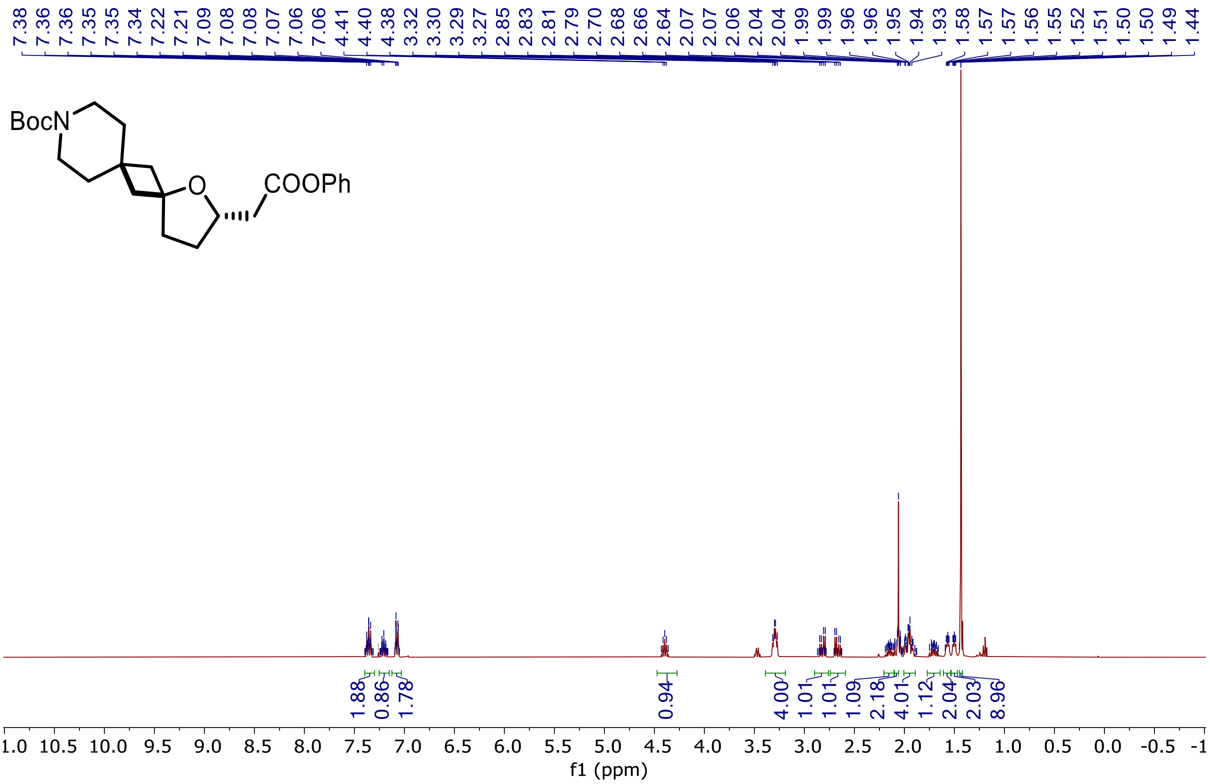

^{13}C NMR: (101 MHz, $CDCl_3$, 298K) of **4aa**

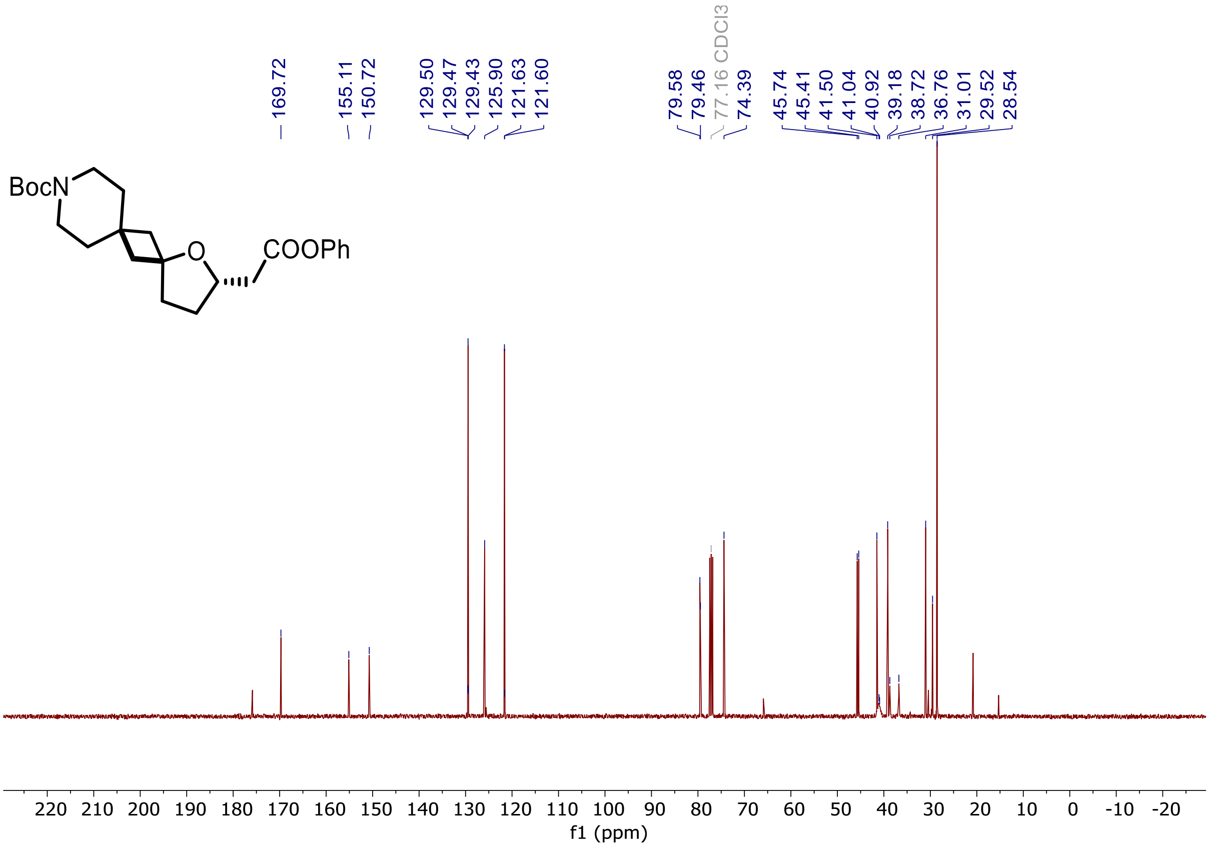

^{1}H NMR: (400 MHz, $CDCl_3$, 298K) of **4ab**

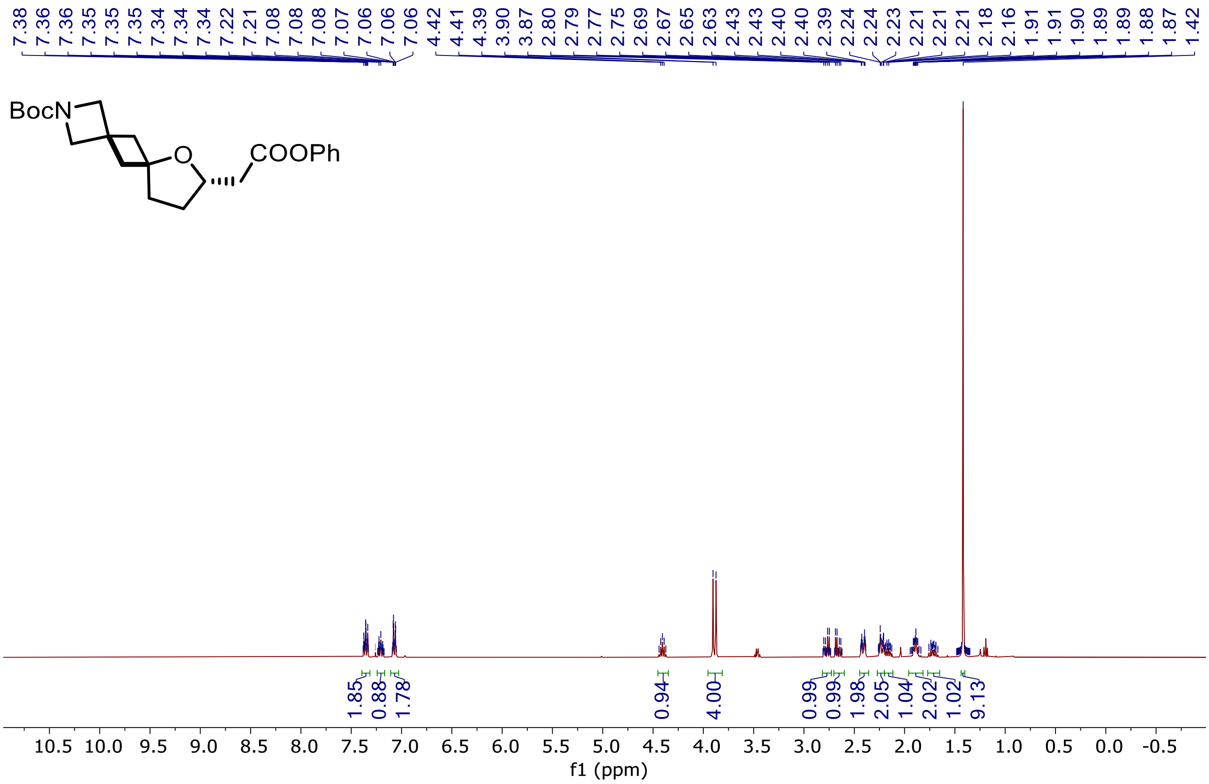

^{13}C NMR: (101 MHz, $CDCl_3$, 298K) of **4ab**

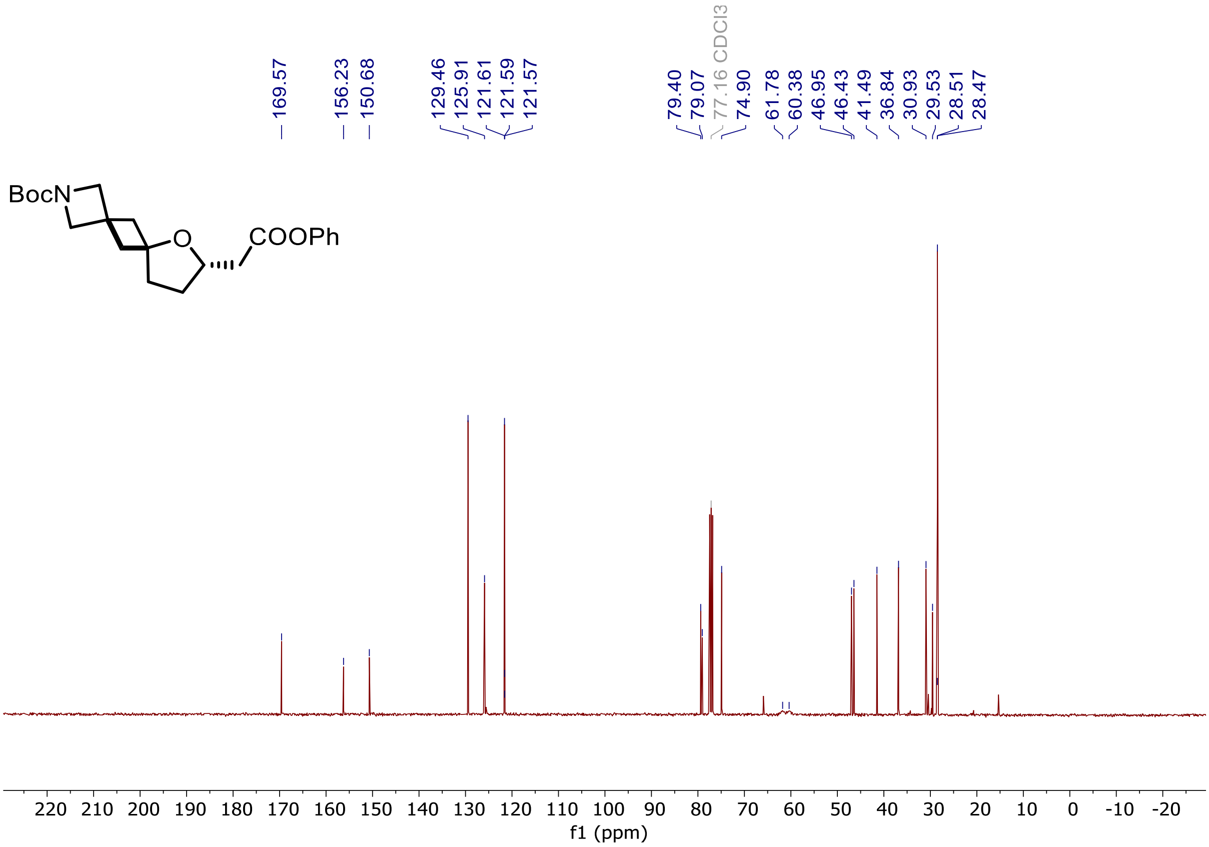

^{1}H NMR: (400 MHz, $CDCl_3$, 298K) of **4ac**

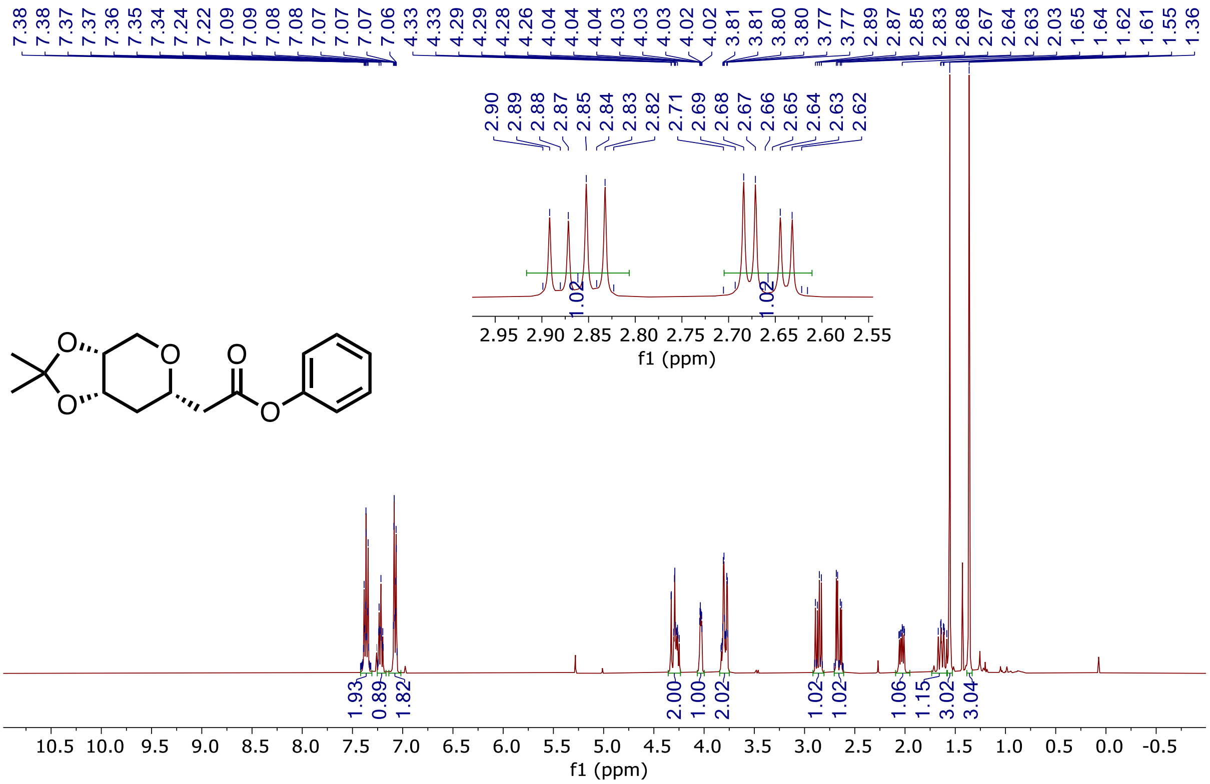

^{13}C NMR: (101 MHz, $CDCl_3$, 298K) of **4ac**

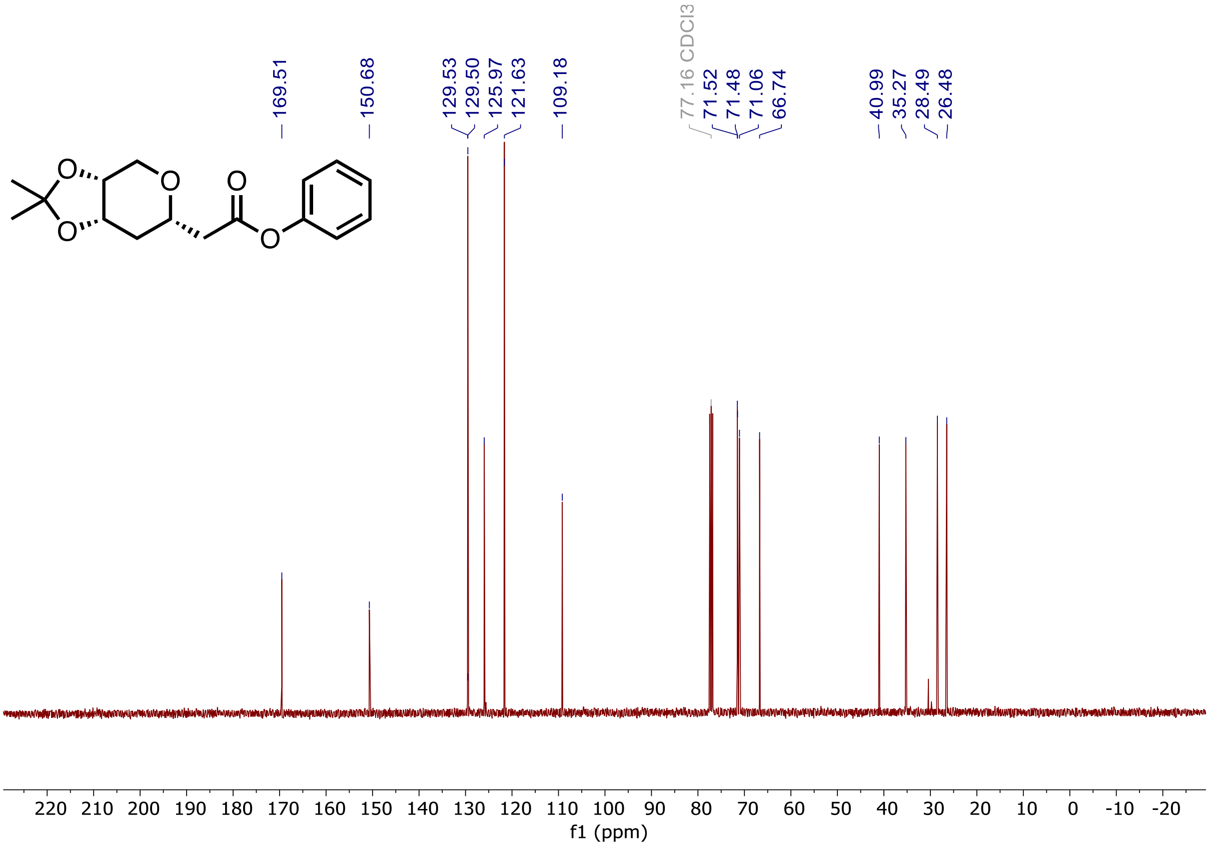

^{1}H NMR: (400 MHz, $CDCl_3$, 298K) of **4ac crude**

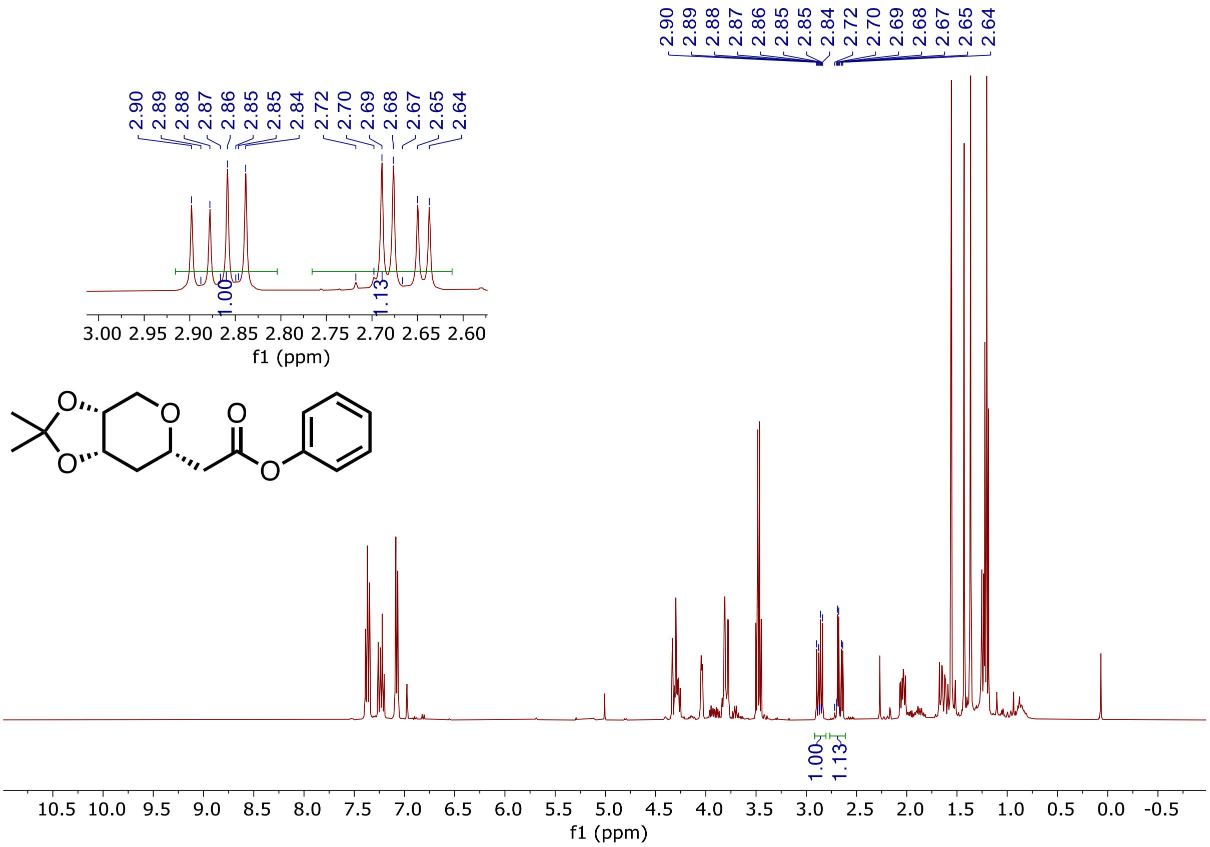

^{1}H NMR: (400 MHz, $CDCl_3$, 298K) of **S30**

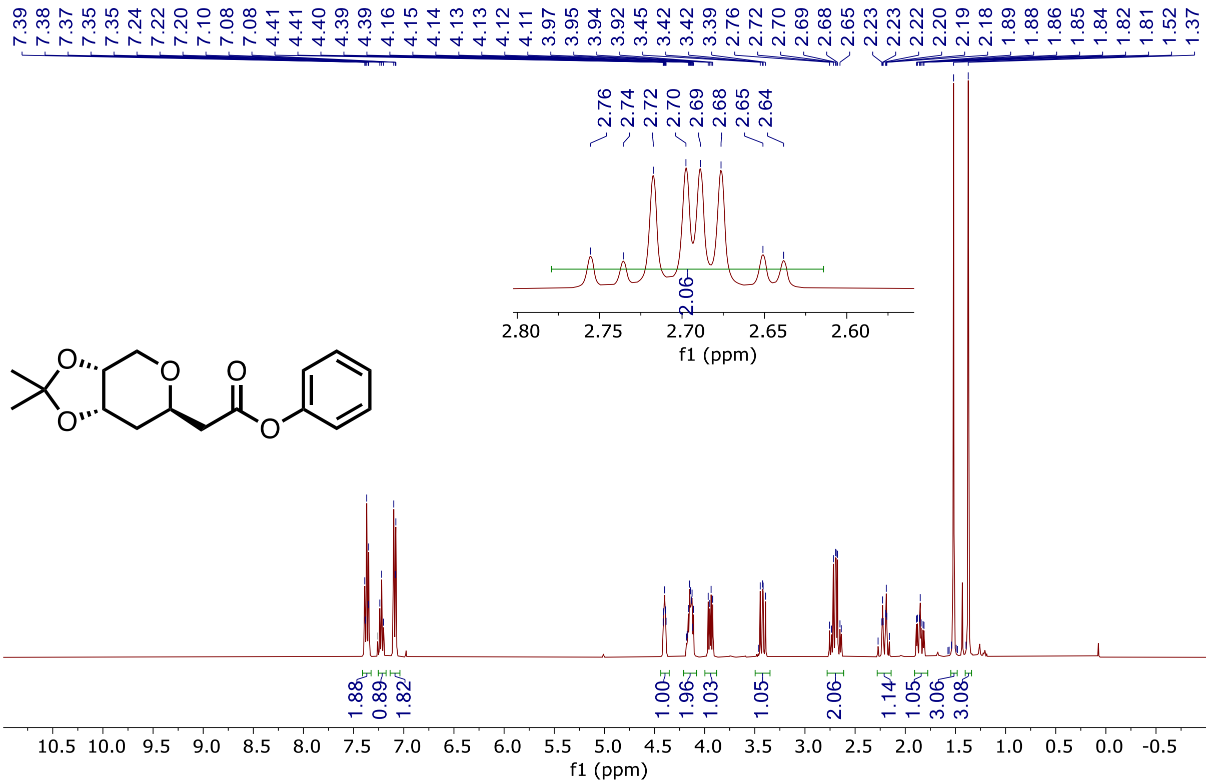

^{13}C NMR: (101 MHz, $CDCl_3$, 298K) of **S30**

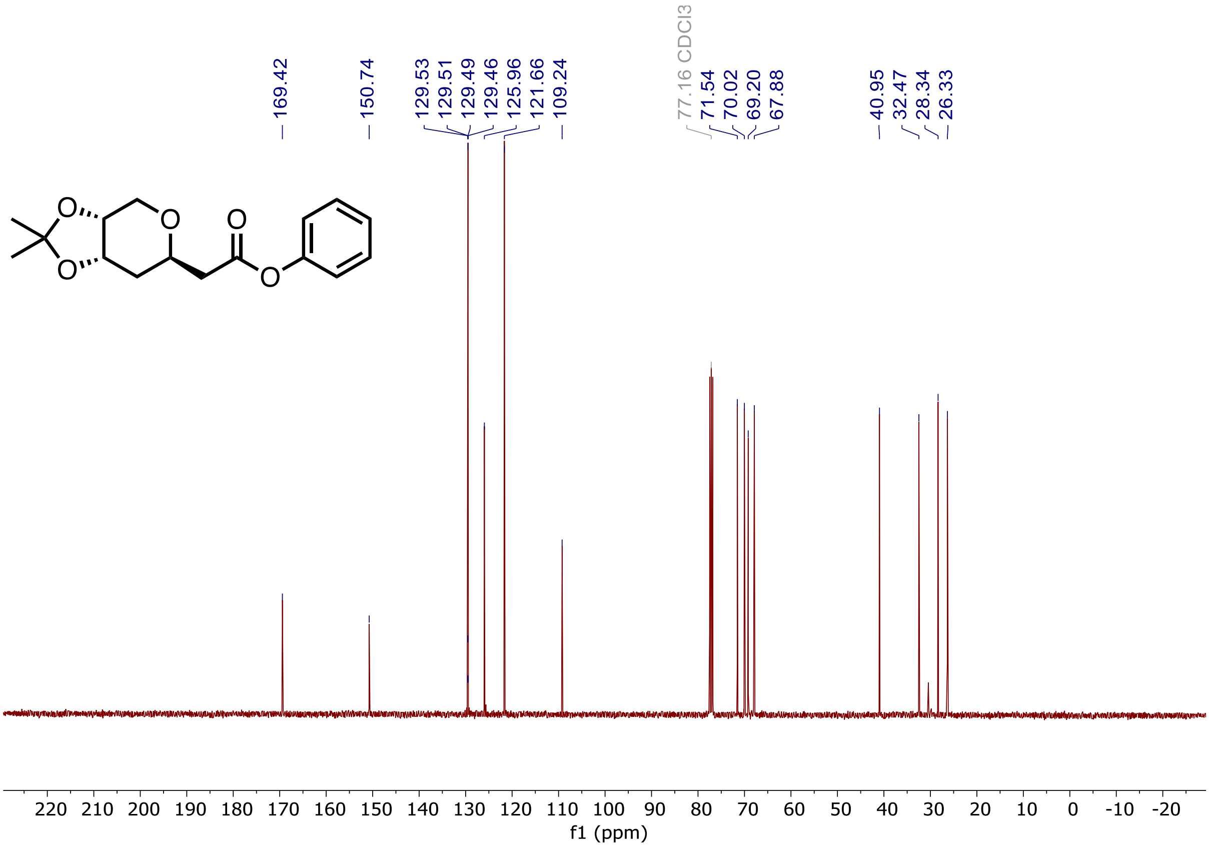

^{1}H NMR: (400 MHz, $CDCl_3$, 298K) of **S30 crude**

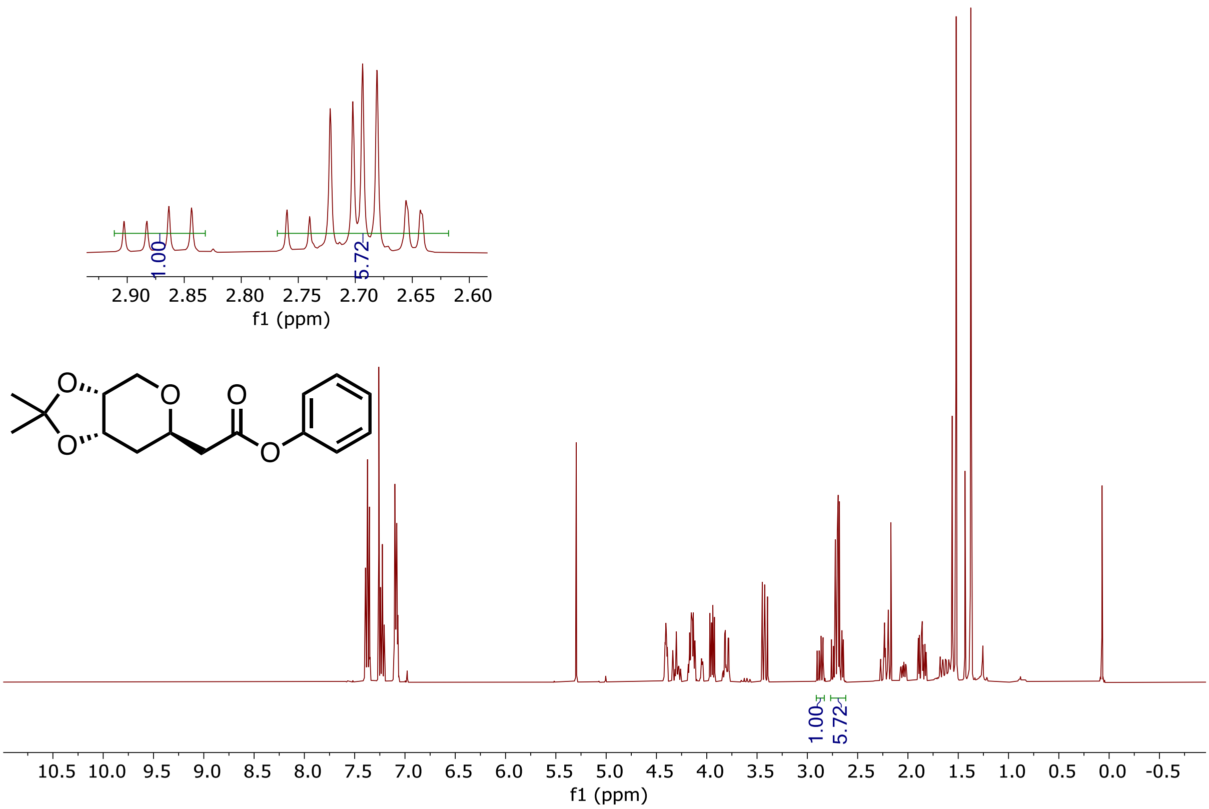

^{1}H NMR: (400 MHz, $CDCl_3$, 298K) of **4ad**

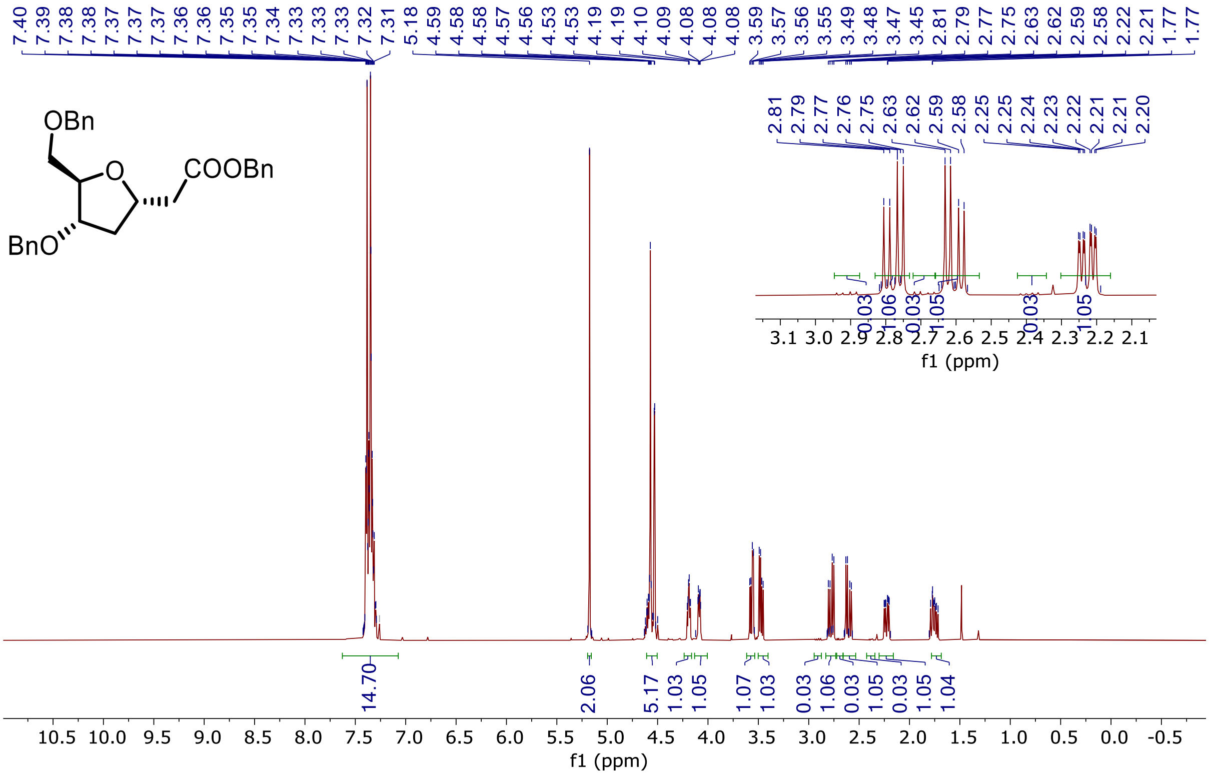

^{13}C NMR: (101 MHz, $CDCl_3$, 298K) of **4ad**

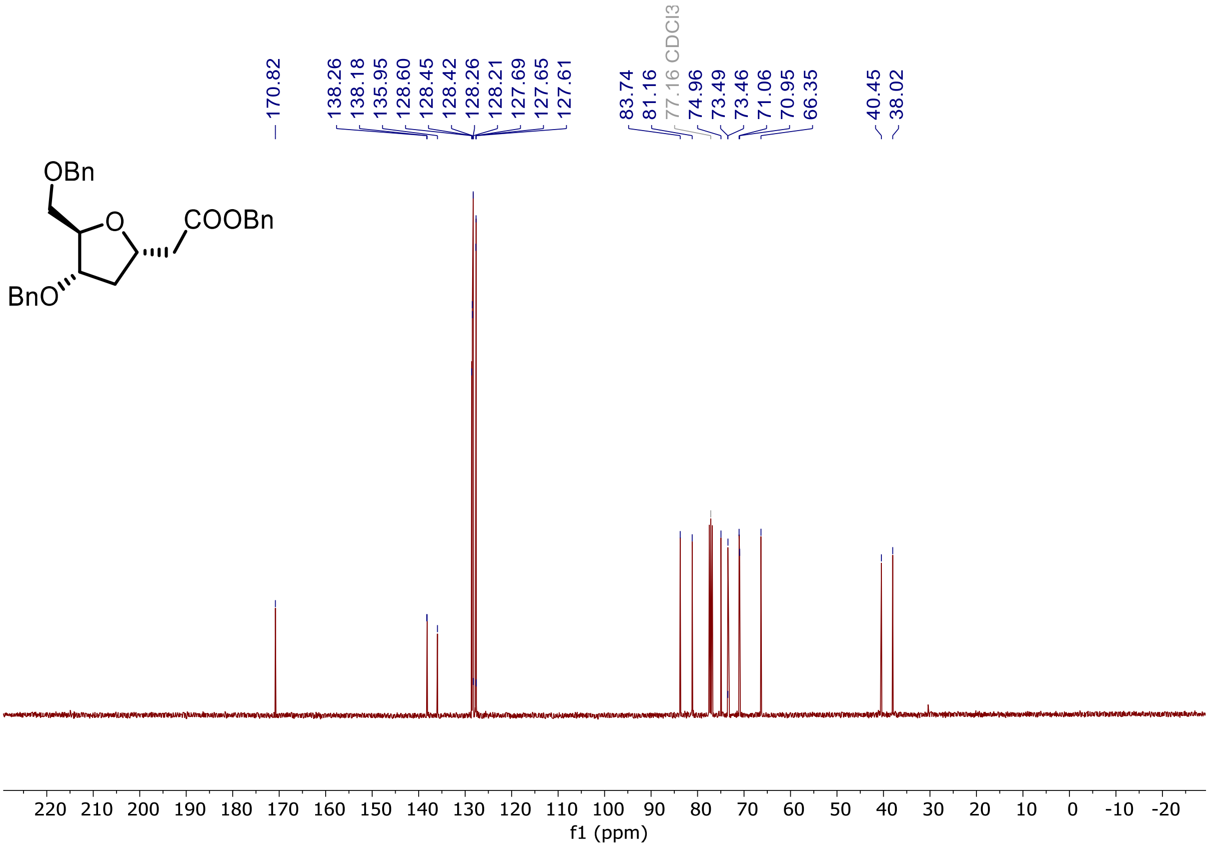

^{1}H NMR: (400 MHz, $CDCl_3$, 298K) of **4ae**

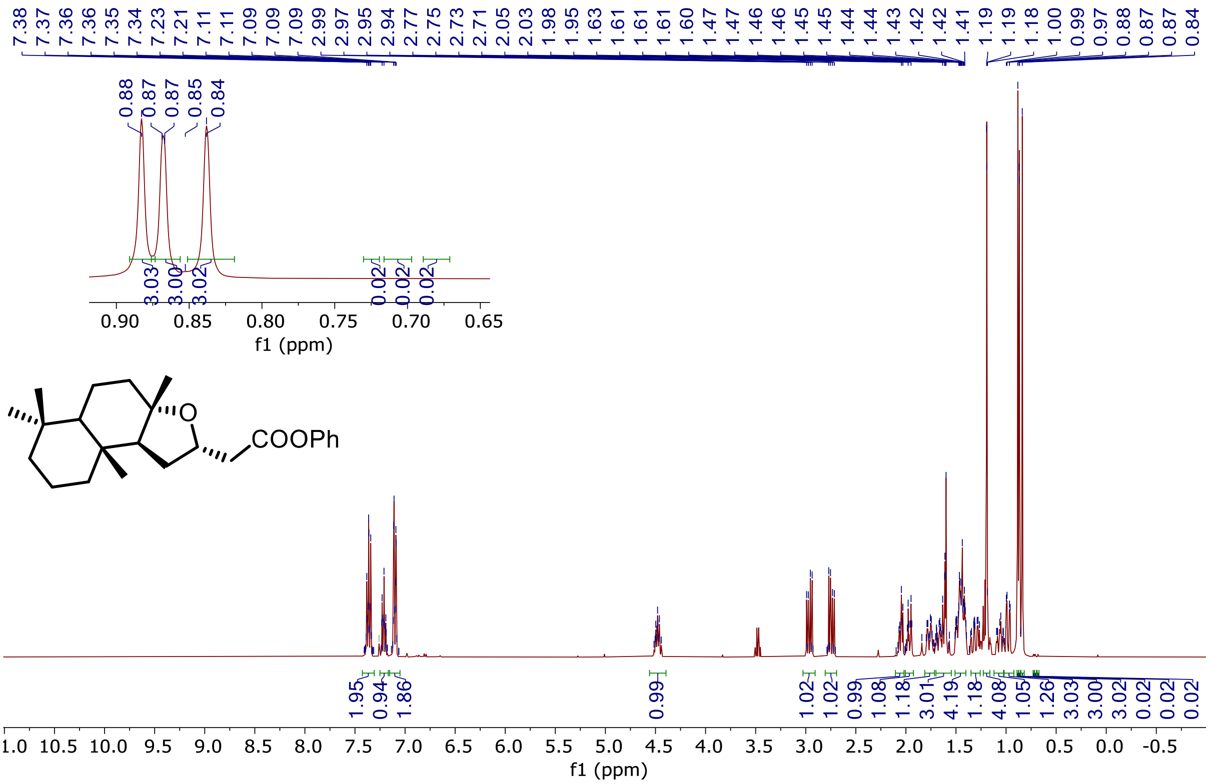

^{13}C NMR: (101 MHz, $CDCl_3$, 298K) of **4ae**

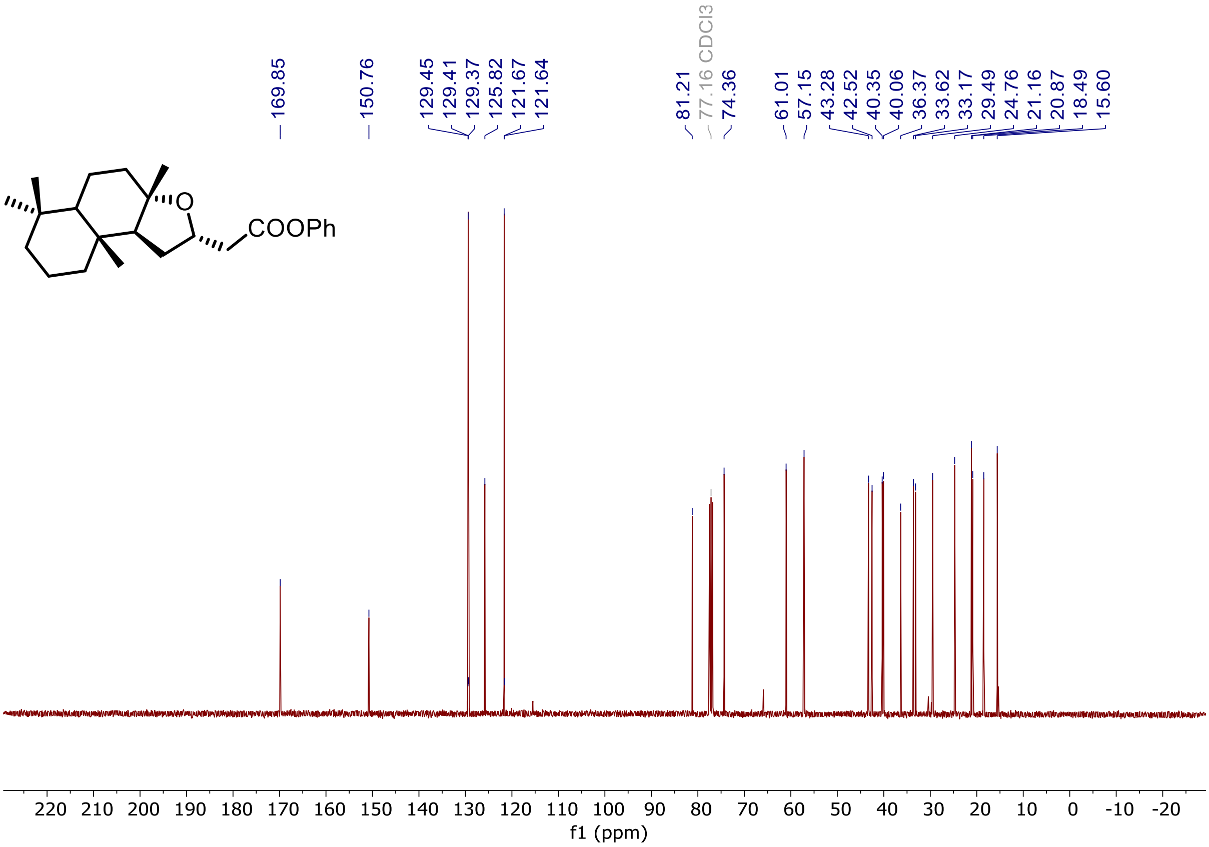

^{1}H NMR: (400 MHz, $CDCl_3$, 298K) of **4ag**

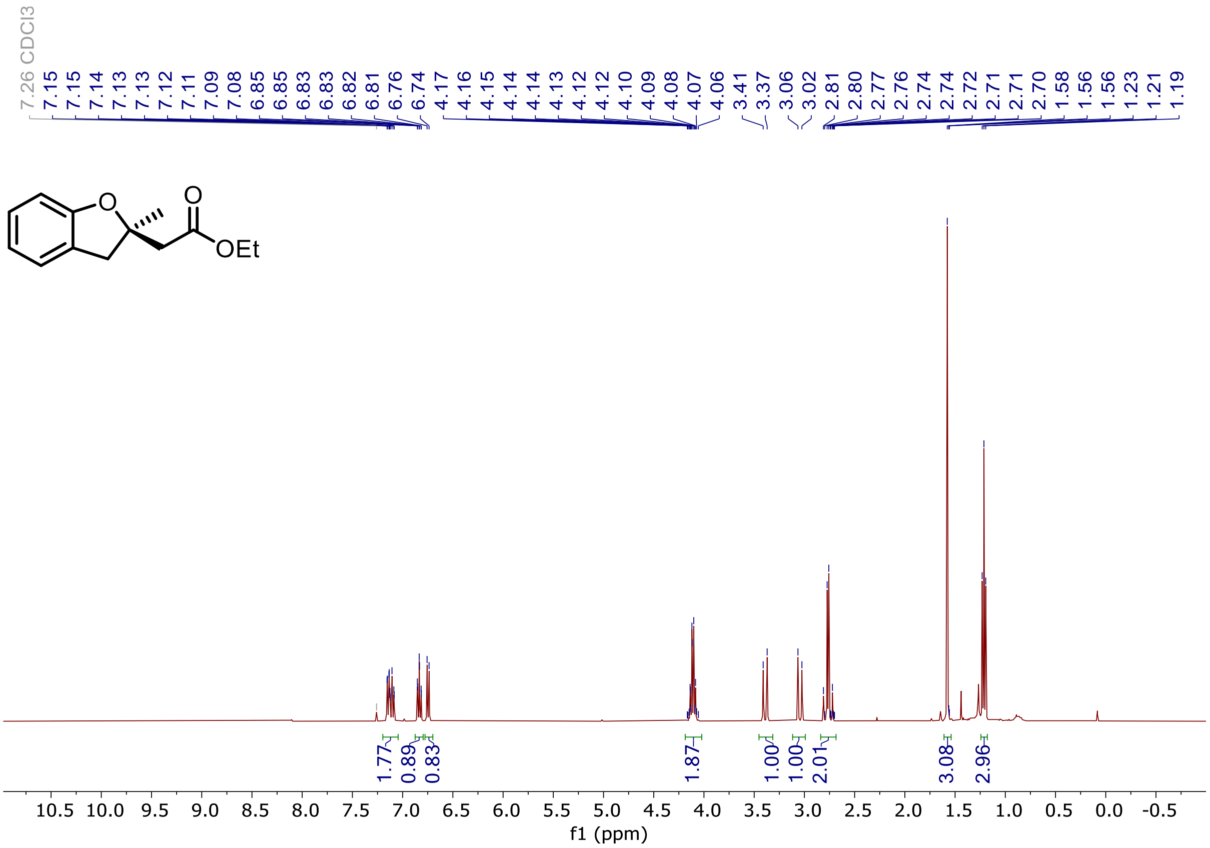

^{13}C NMR: (101 MHz, $CDCl_3$, 298K) of **4ag**

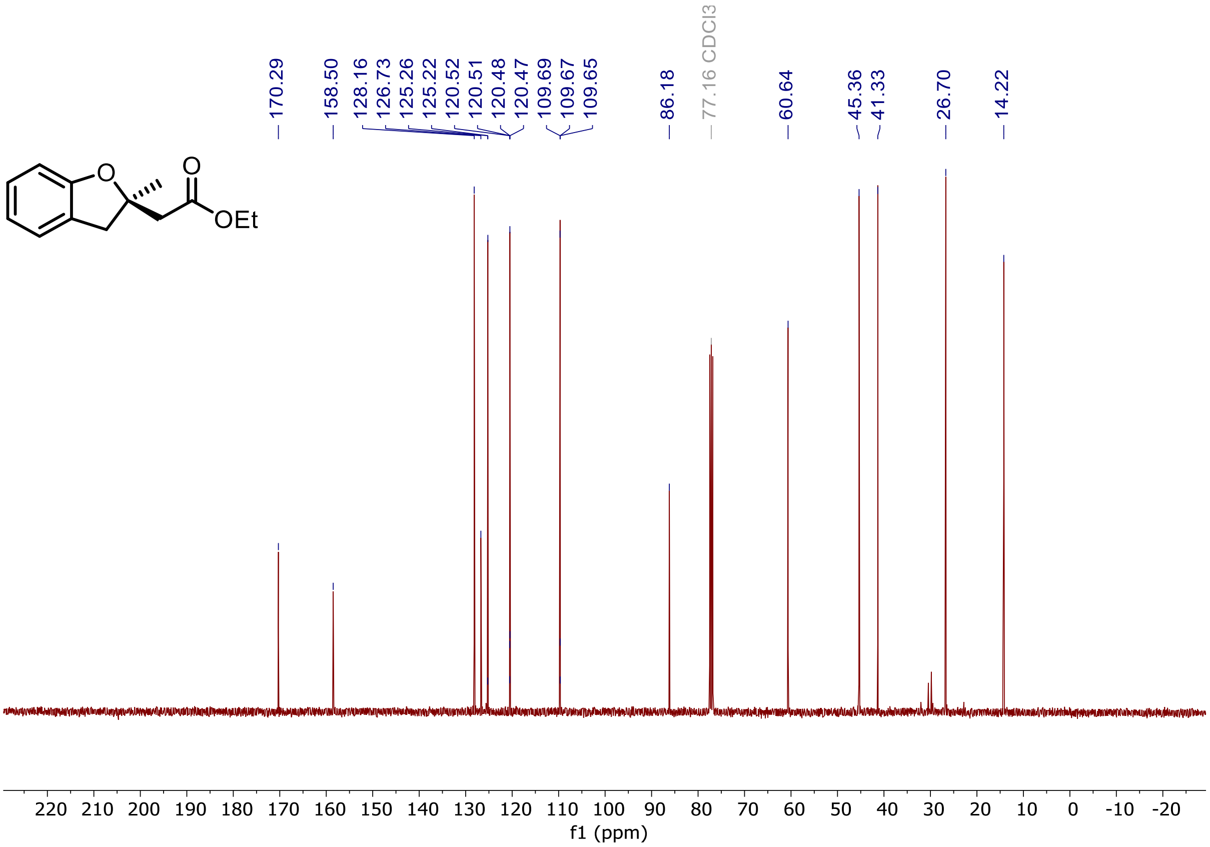

^{1}H NMR: (400 MHz, $CDCl_3$, 298K) of **4ah**

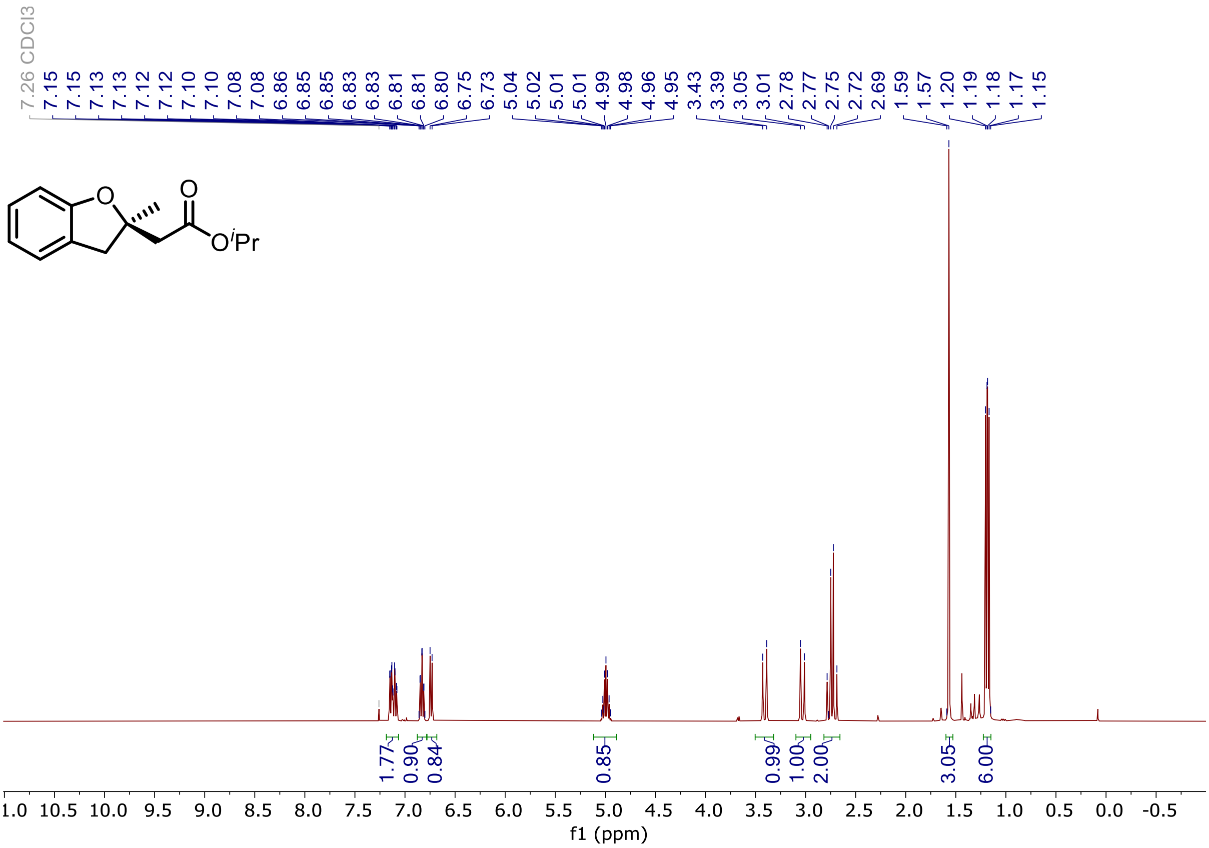

^{13}C NMR: (101 MHz, $CDCl_3$, 298K) of **4ah**

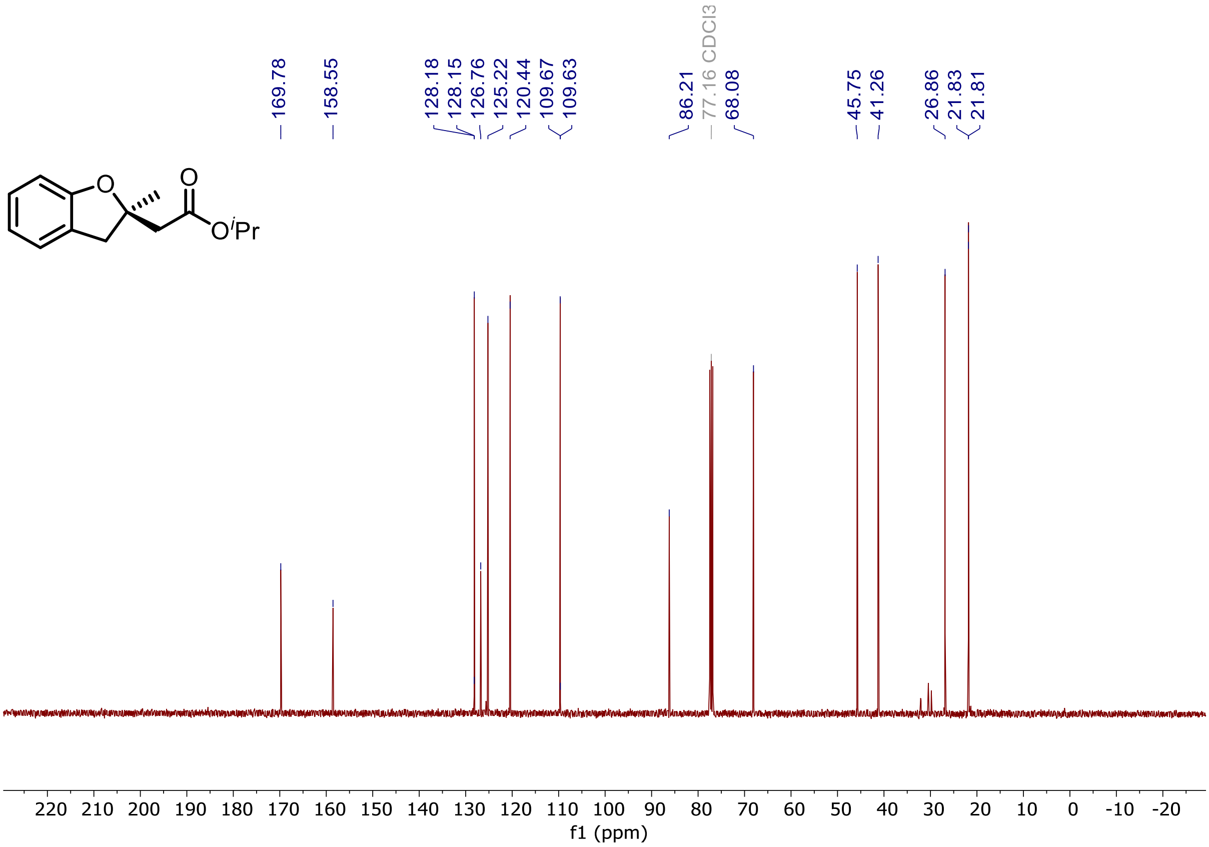

^{1}H NMR: (400 MHz, $CDCl_3$, 298K) of **4ai**

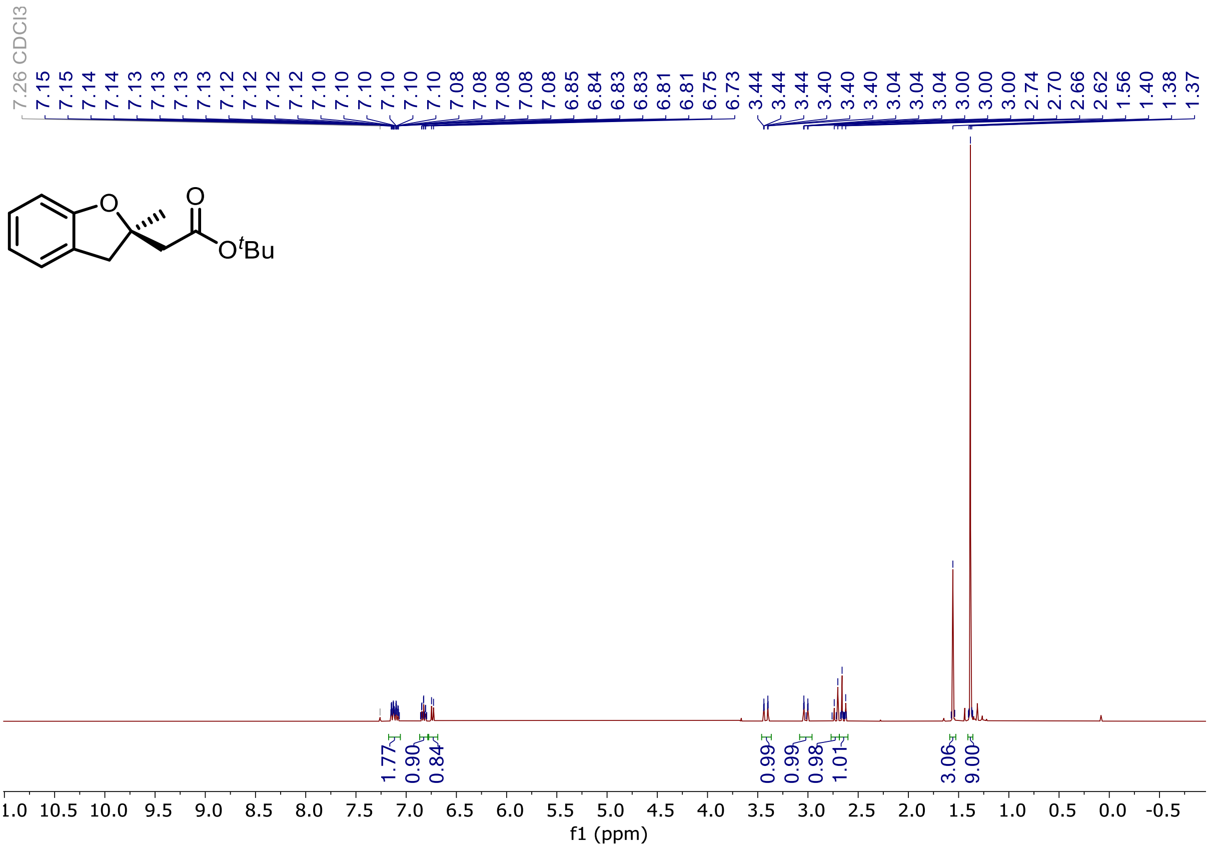

^{13}C NMR: (101 MHz, $CDCl_3$, 298K) of **4ai**

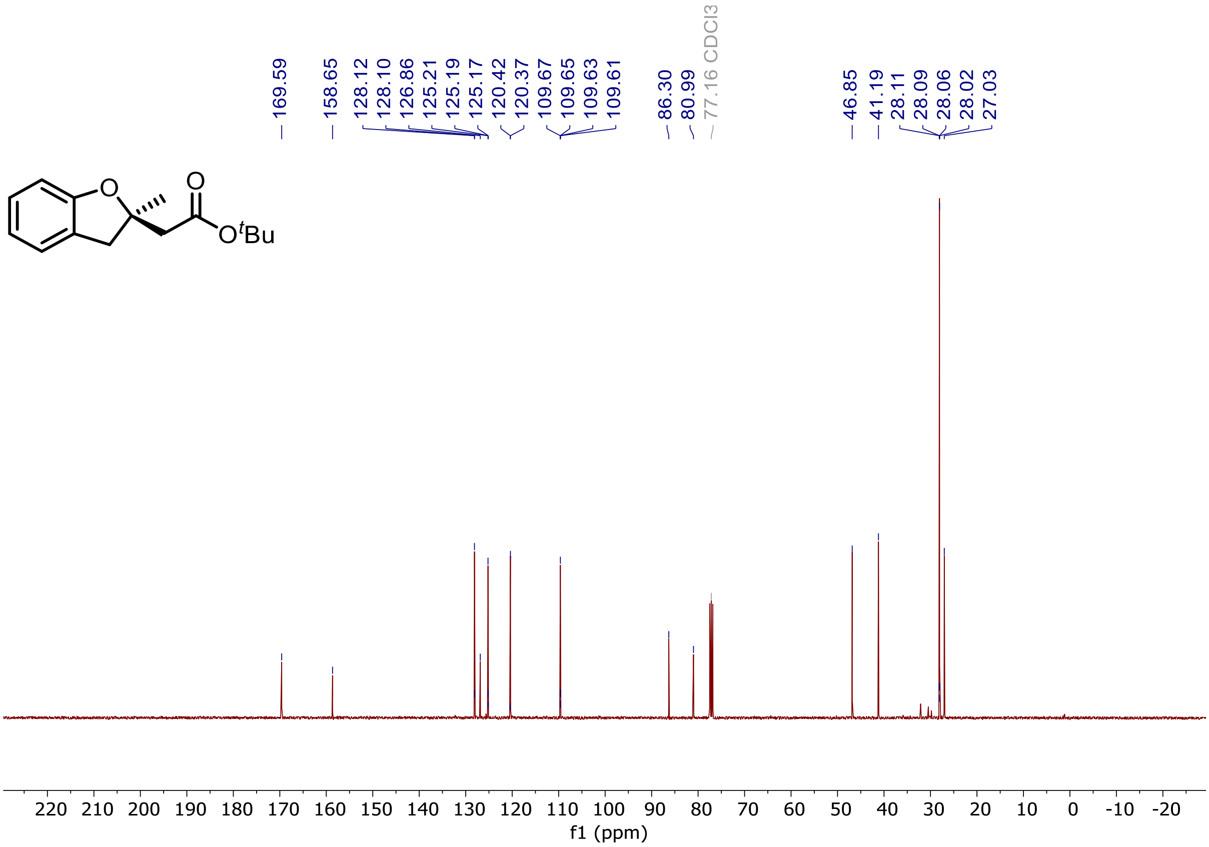

^{1}H NMR: (400 MHz, $CDCl_3$, 298K) of **4aj**

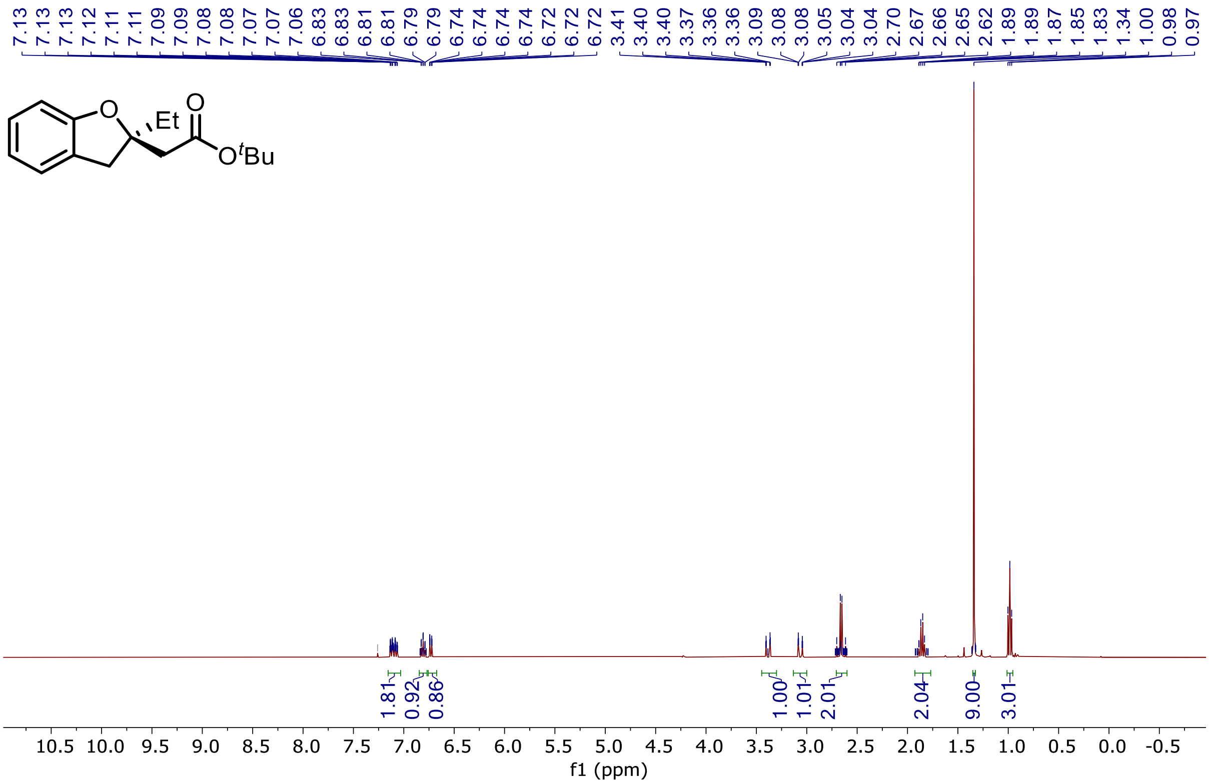

^{13}C NMR: (101 MHz, $CDCl_3$, 298K) of **4aj**

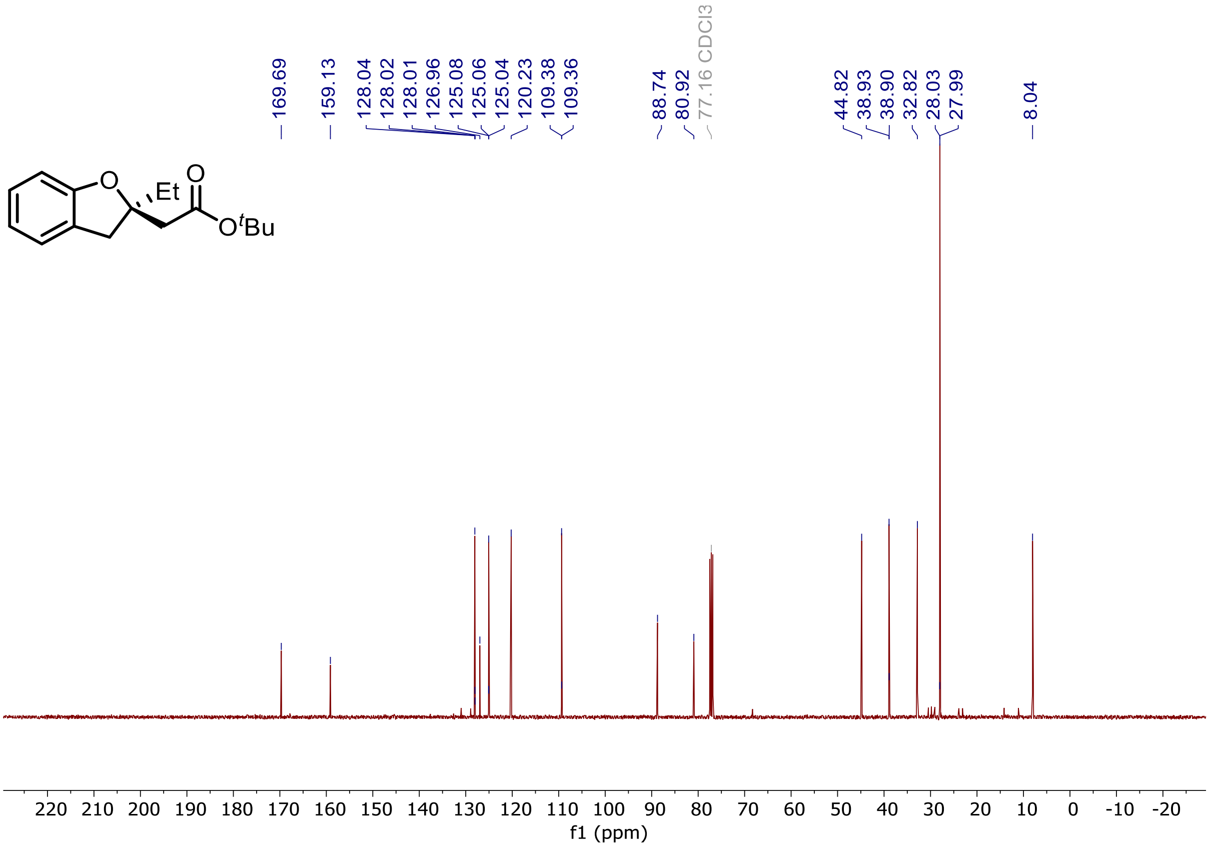

^{1}H NMR: (400 MHz, $CDCl_3$, 298K) of **4ak**

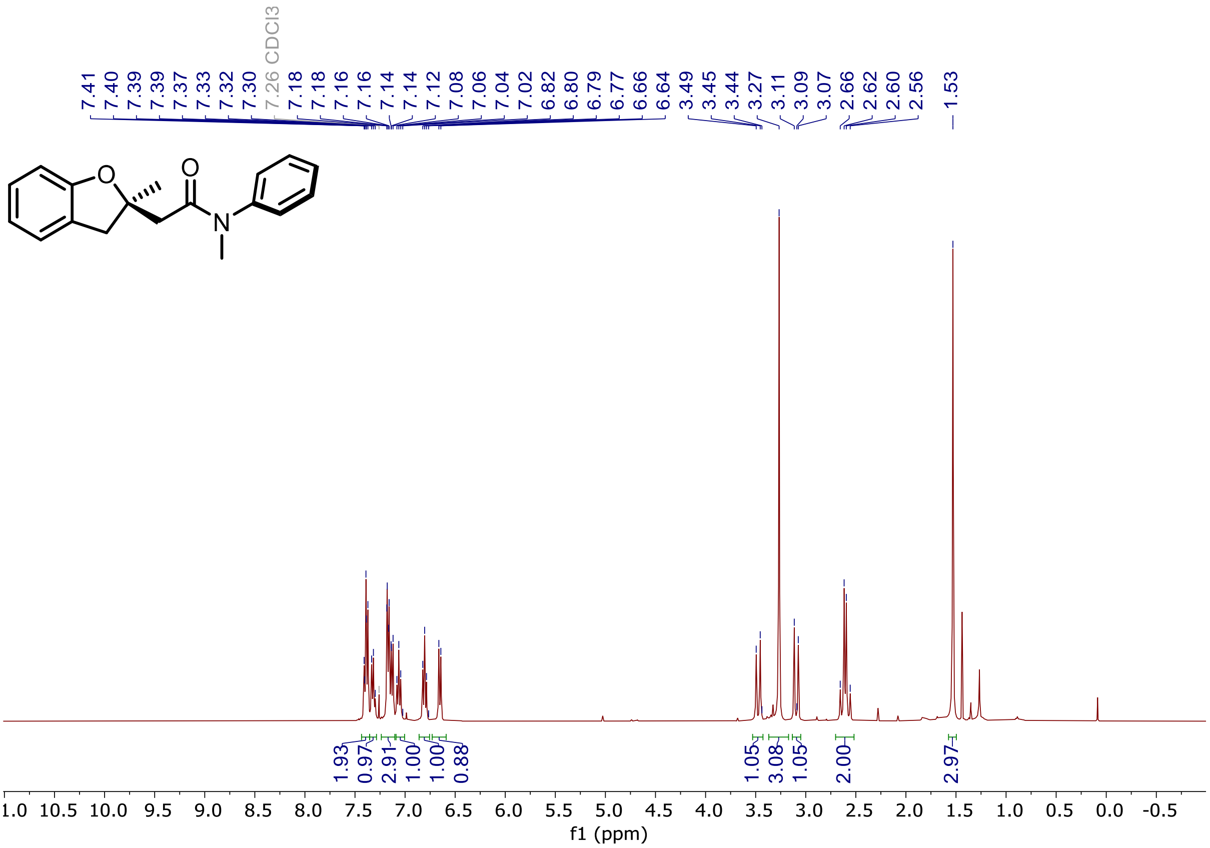

^{13}C NMR: (101 MHz, $CDCl_3$, 298K) of **4ak**

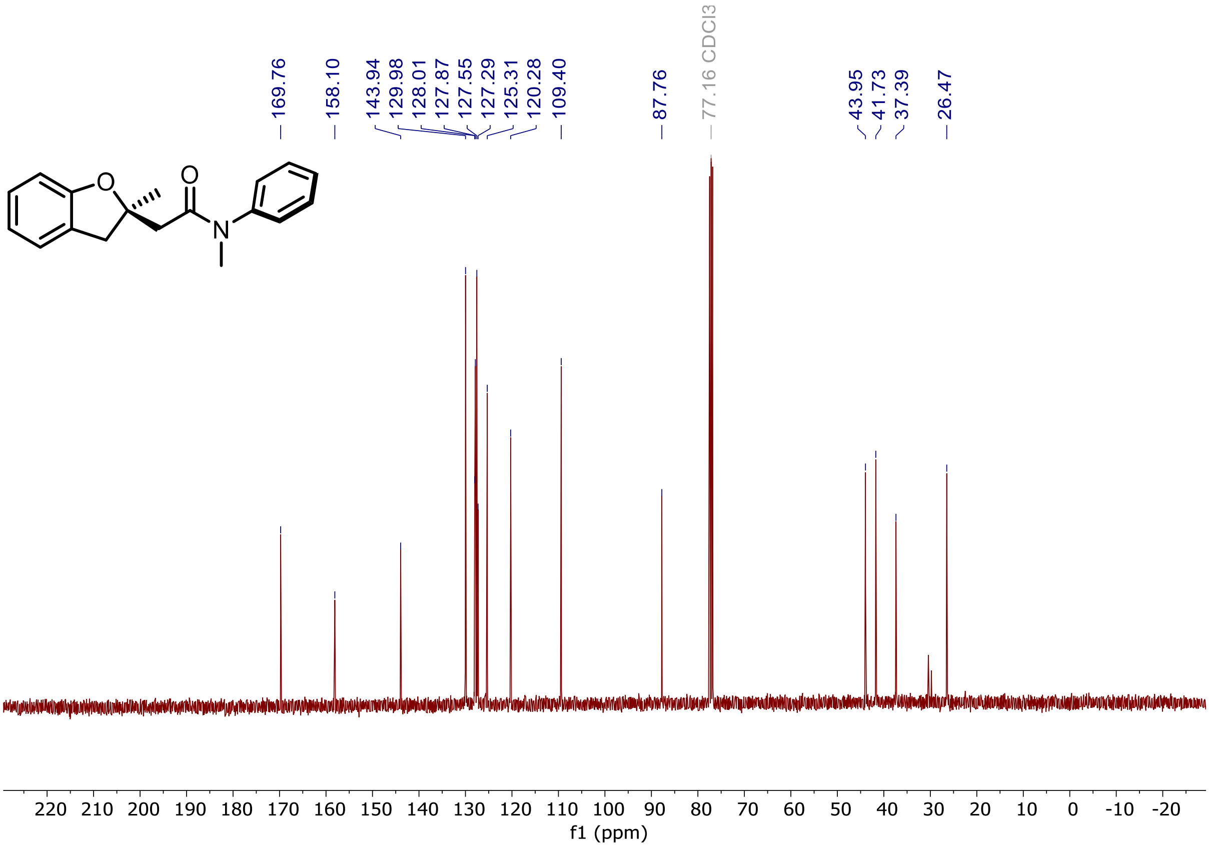

^{1}H NMR: (400 MHz, $CDCl_3$, 298K) of **4al**

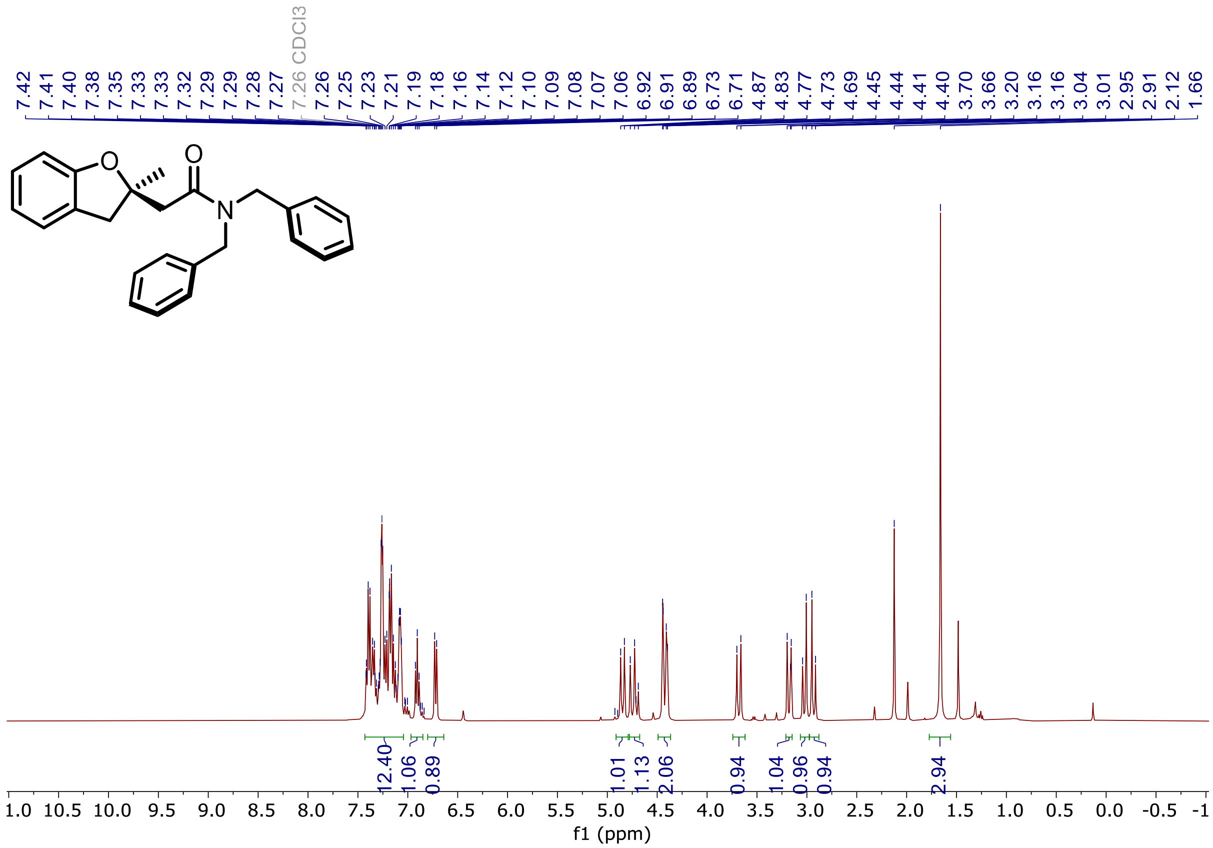

^{13}C NMR: (101 MHz, $CDCl_3$, 298K) of **4al**

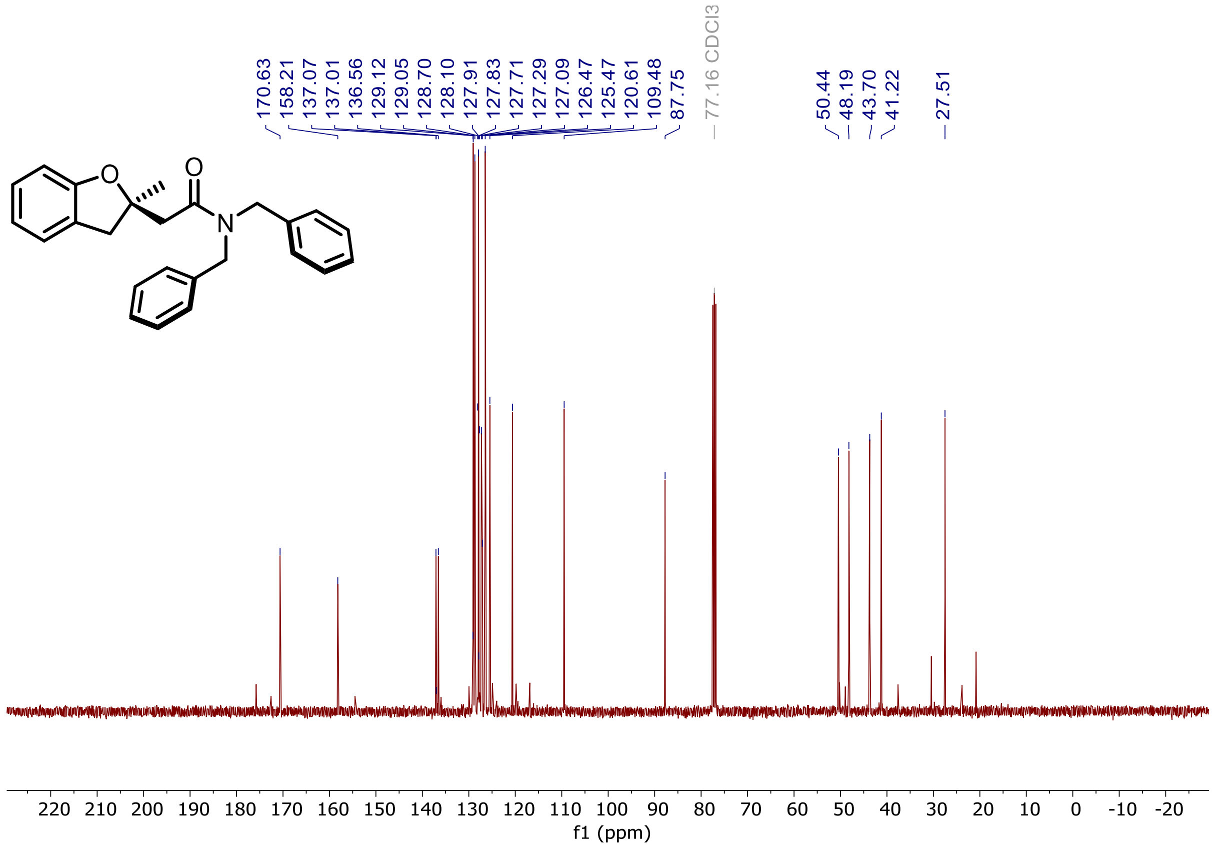

^{1}H NMR: (400 MHz, $CDCl_3$, 298K) of **4an**

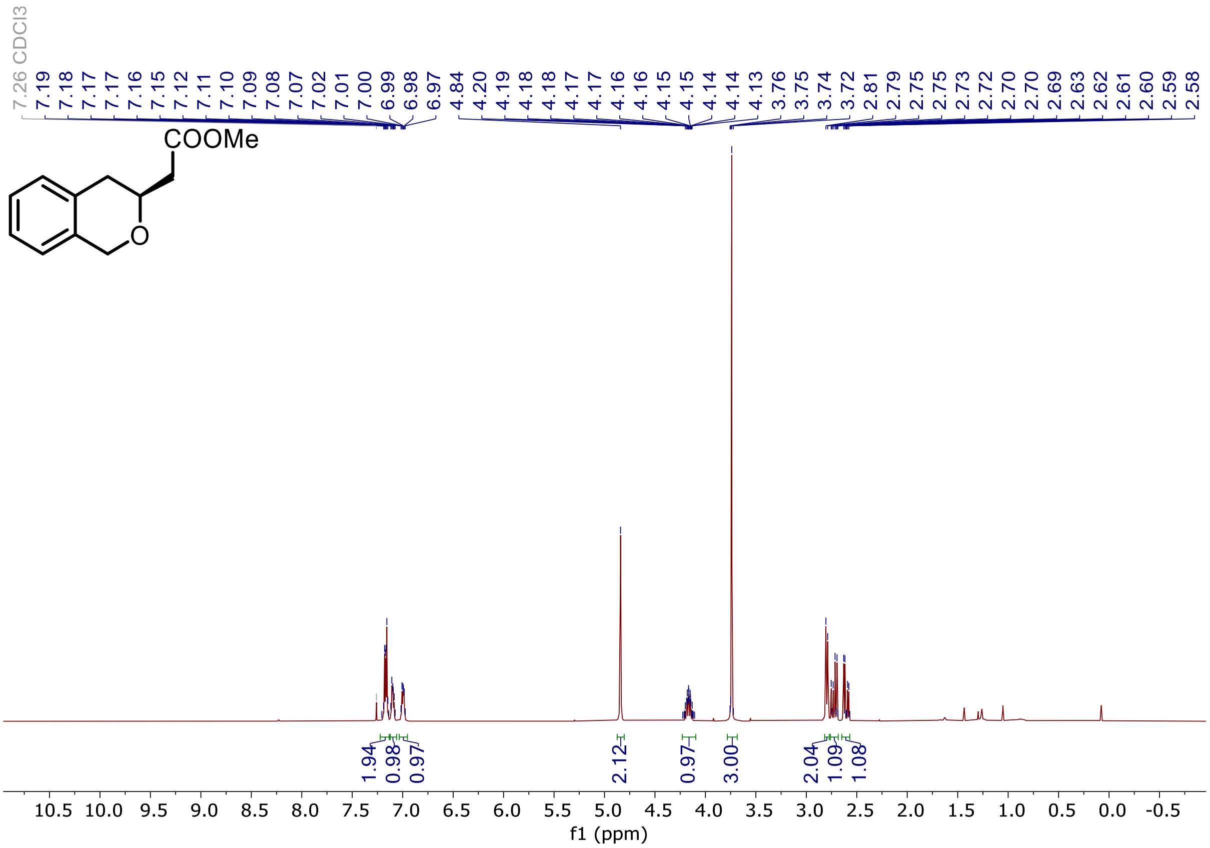

^{13}C NMR: (101 MHz, $CDCl_3$, 298K) of **4an**

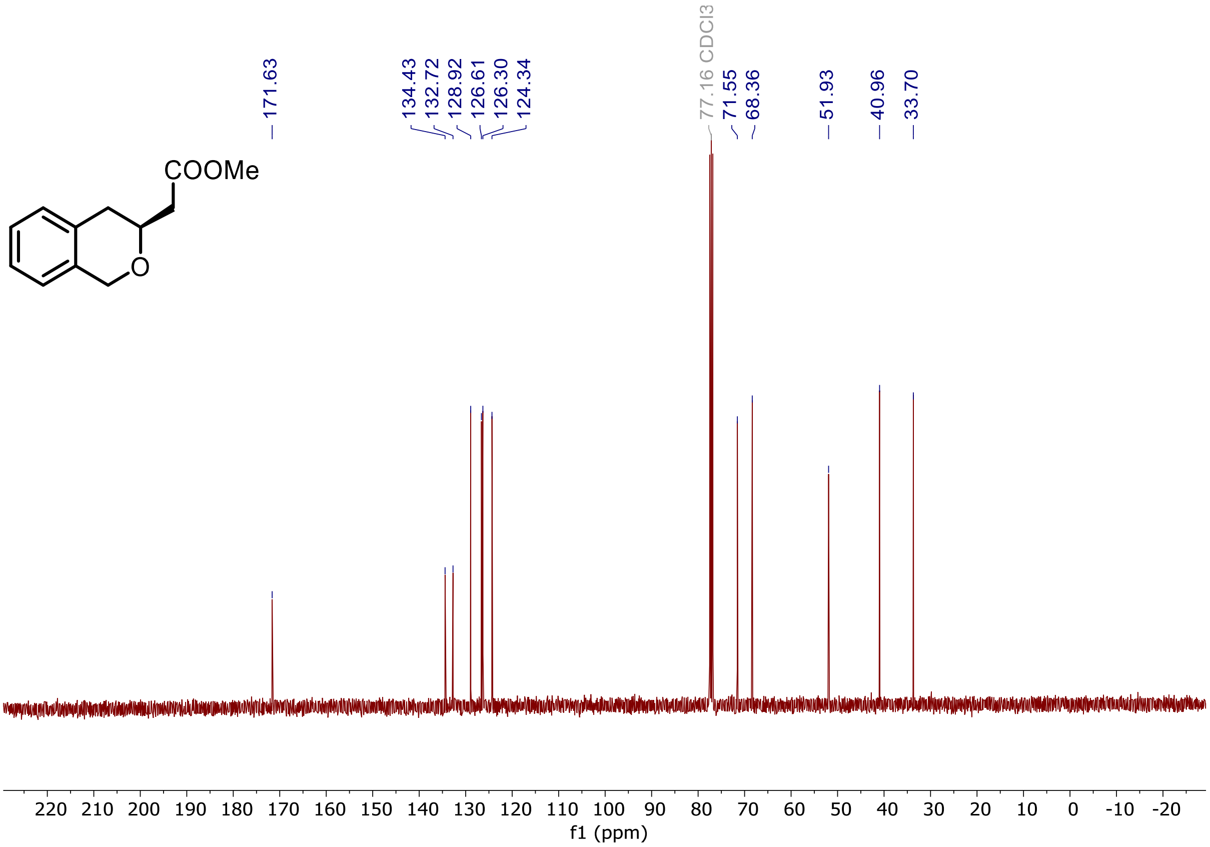

^{1}H NMR: (400 MHz, $CDCl_3$, 298K) of **4ao**

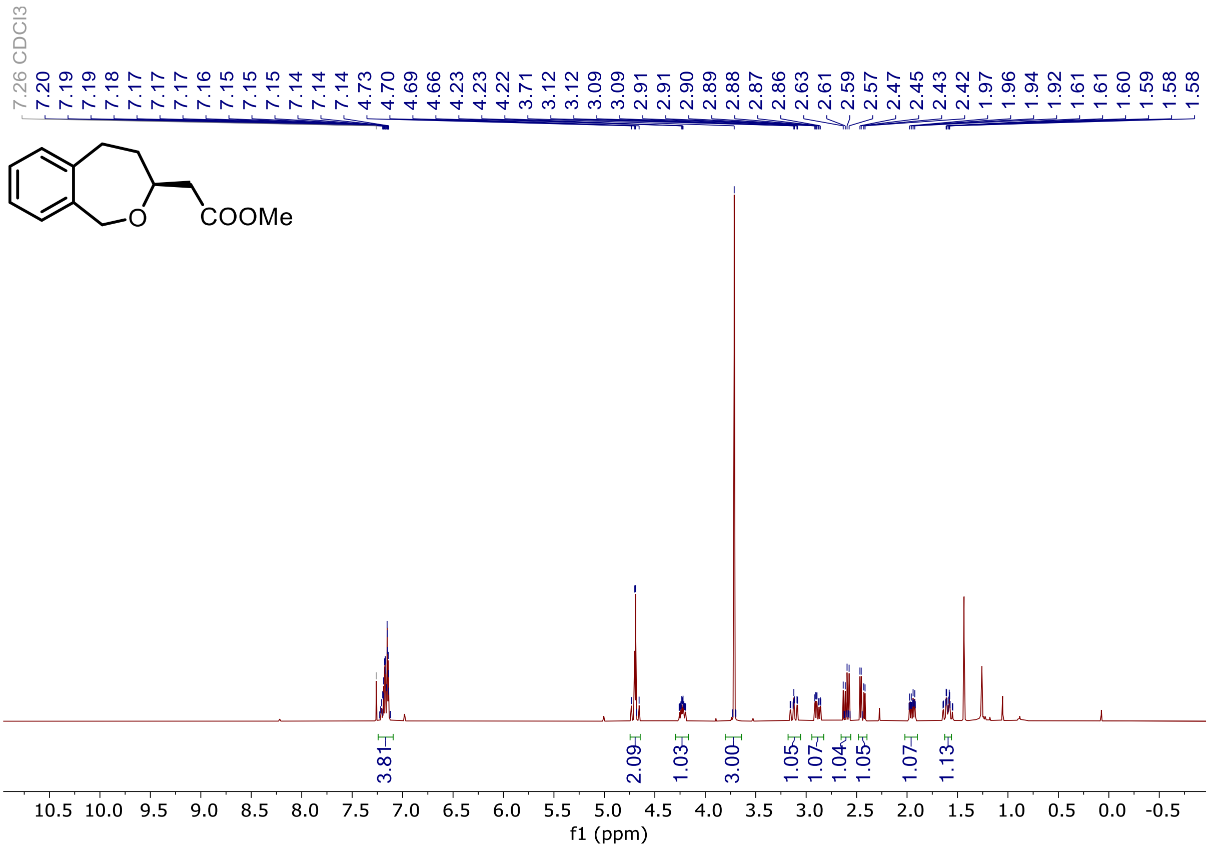

^{13}C NMR: (101 MHz, $CDCl_3$, 298K) of **4ao**

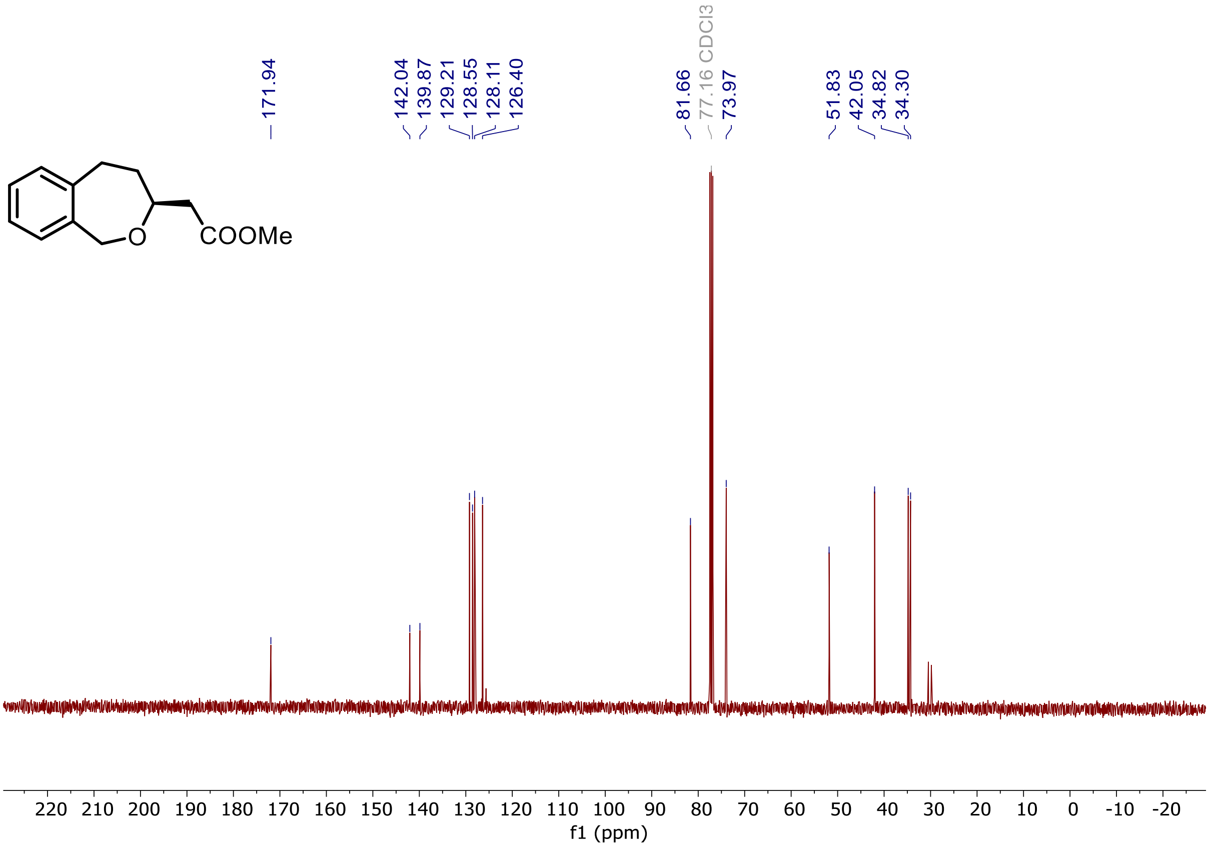

^{1}H NMR: (400 MHz, $CDCl_3$, 298K) of **4ap**

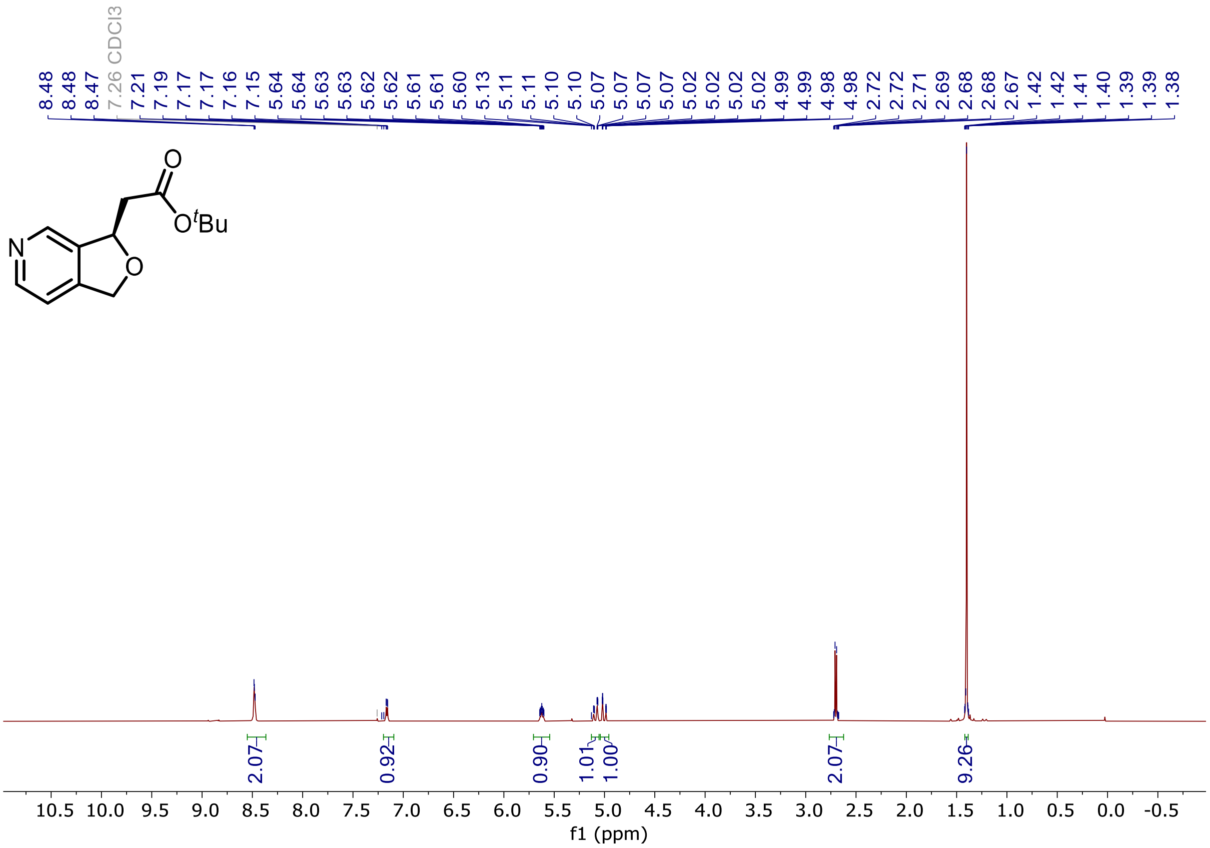

^{13}C NMR: (101 MHz, $CDCl_3$, 298K) of **4ap**

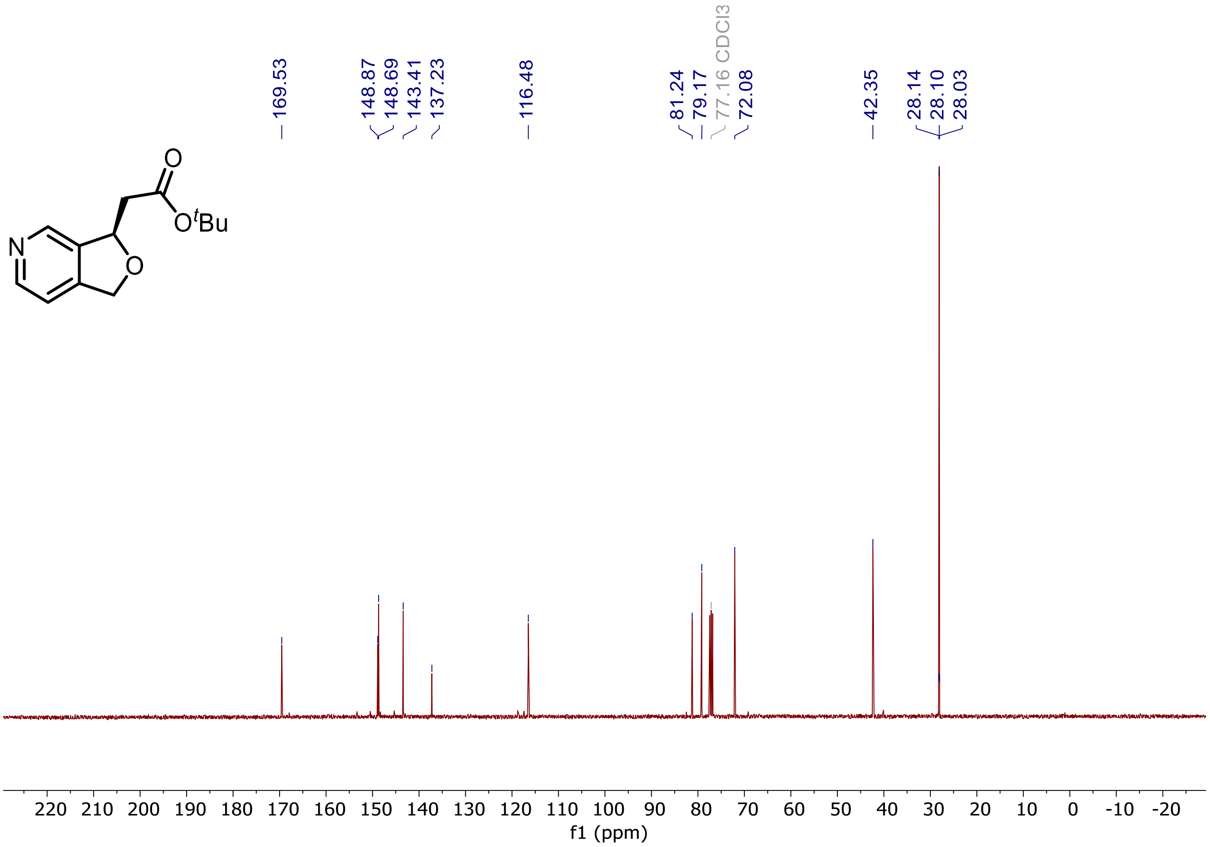

^{1}H NMR: (400 MHz, $CDCl_3$, 298K) of **4ar**

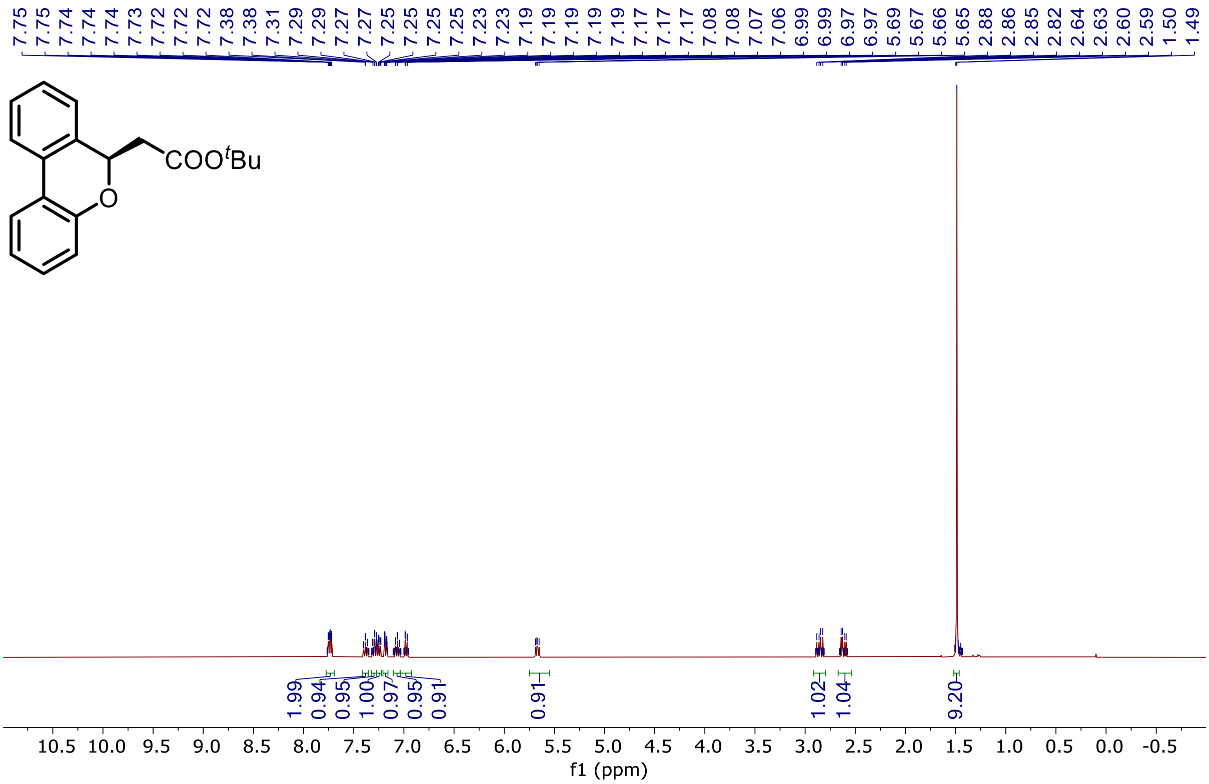

^{13}C NMR: (101 MHz, $CDCl_3$, 298K) of **4ar**

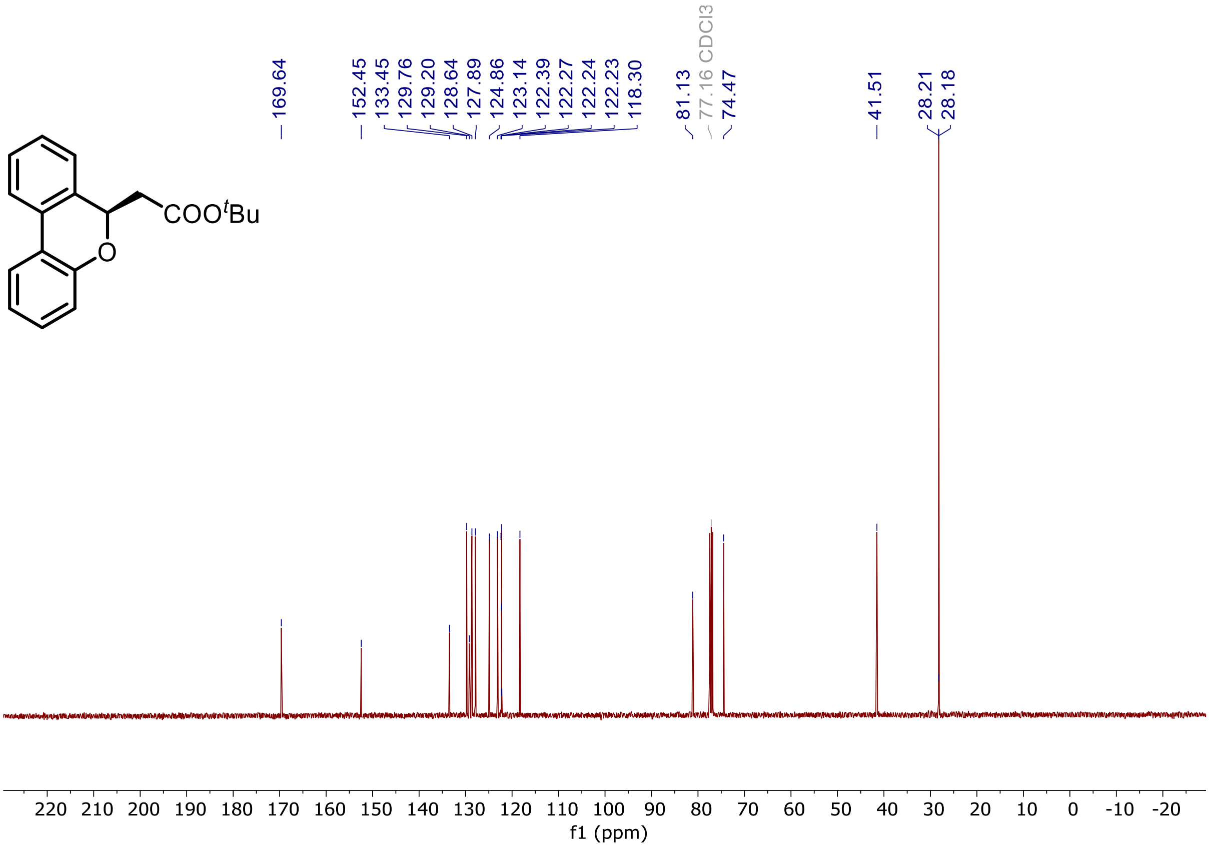

^{1}H NMR: (400 MHz, $CDCl_3$, 298K) of **5b**

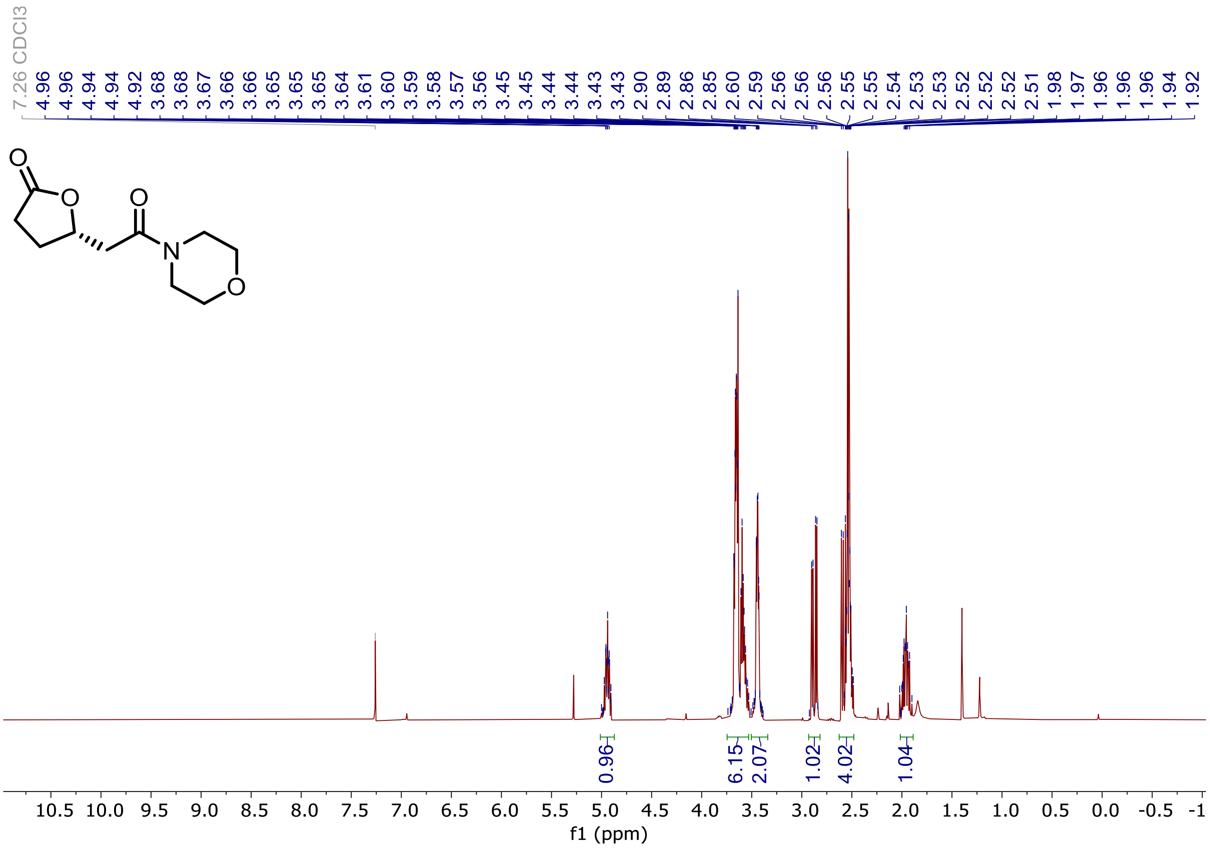

^{13}C NMR: (101 MHz, $CDCl_3$, 298K) of **5b**

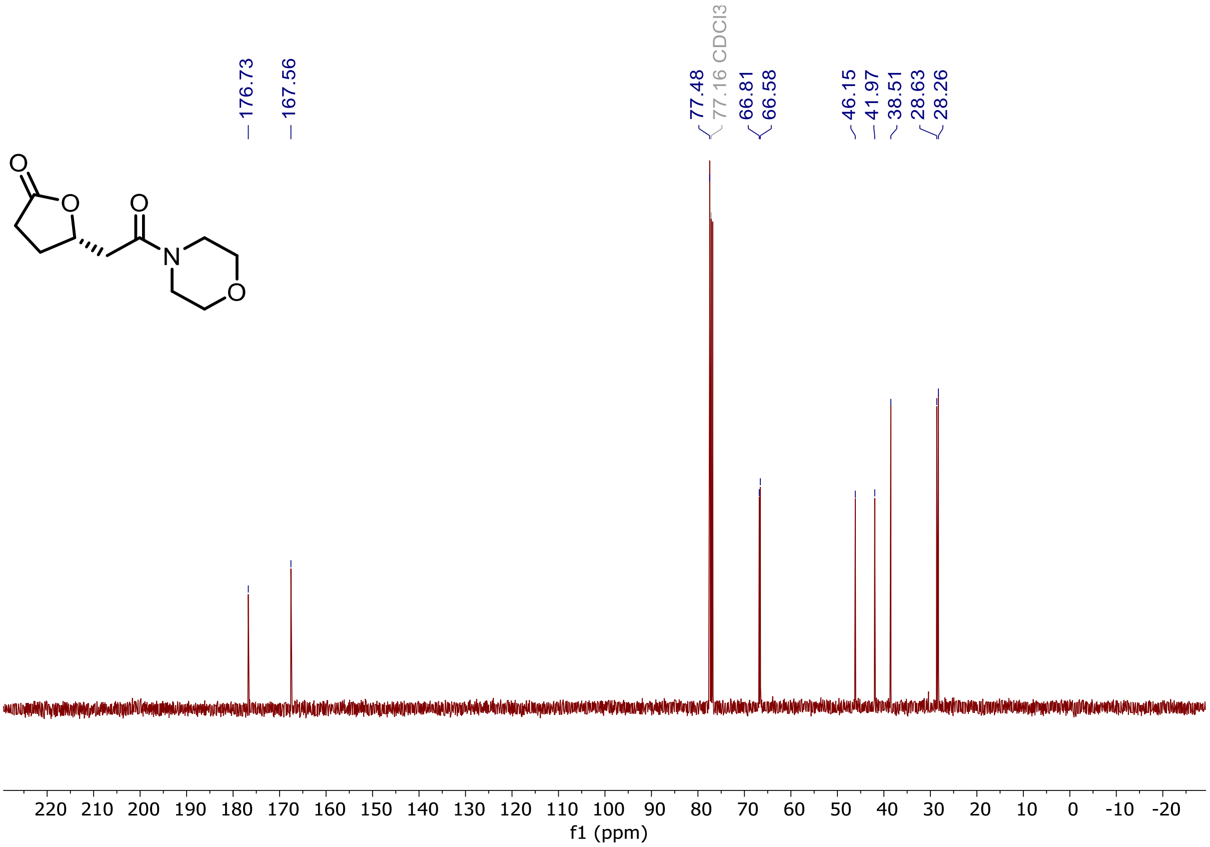

^{1}H NMR: (400 MHz, $CDCl_3$, 298K) of **5c**

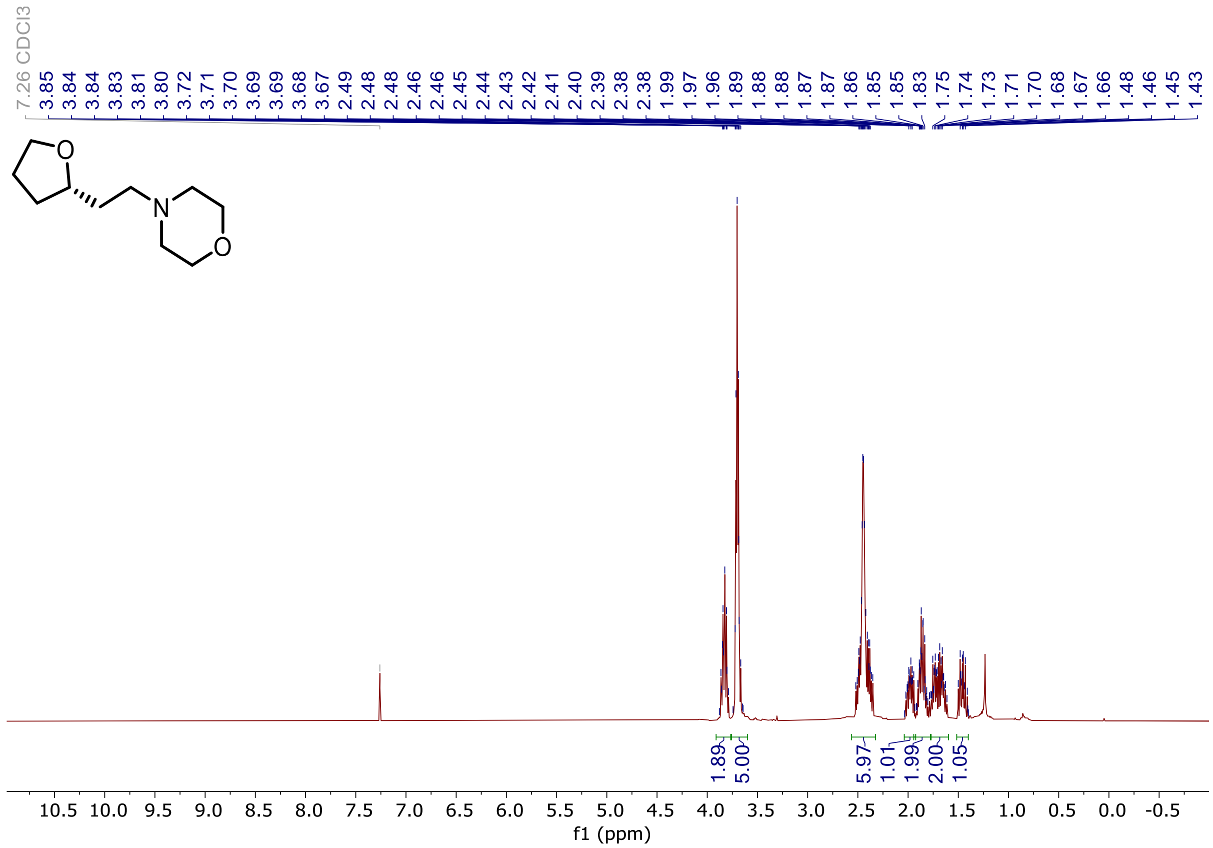

^{13}C NMR: (101 MHz, $CDCl_3$, 298K) of **5c**

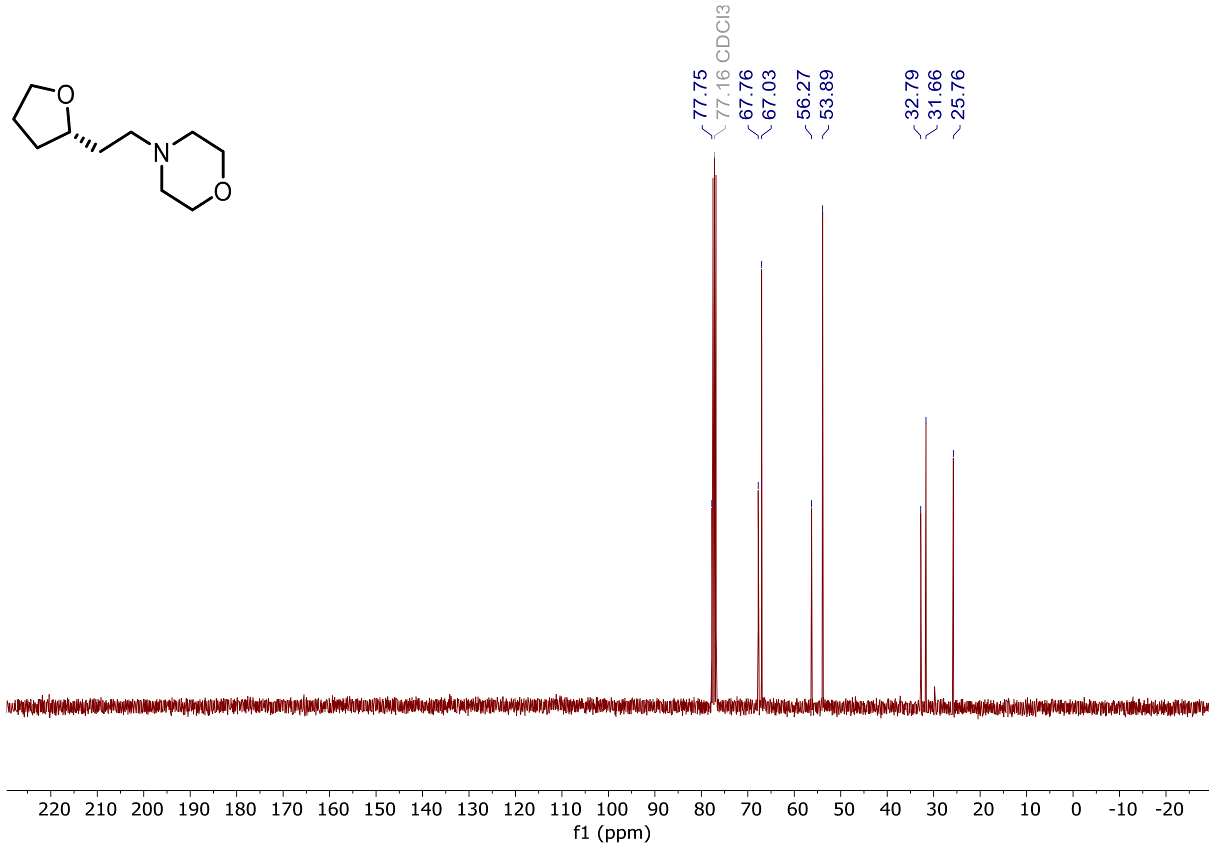

^{1}H NMR: (400 MHz, $CDCl_3$, 298K) of **5d**

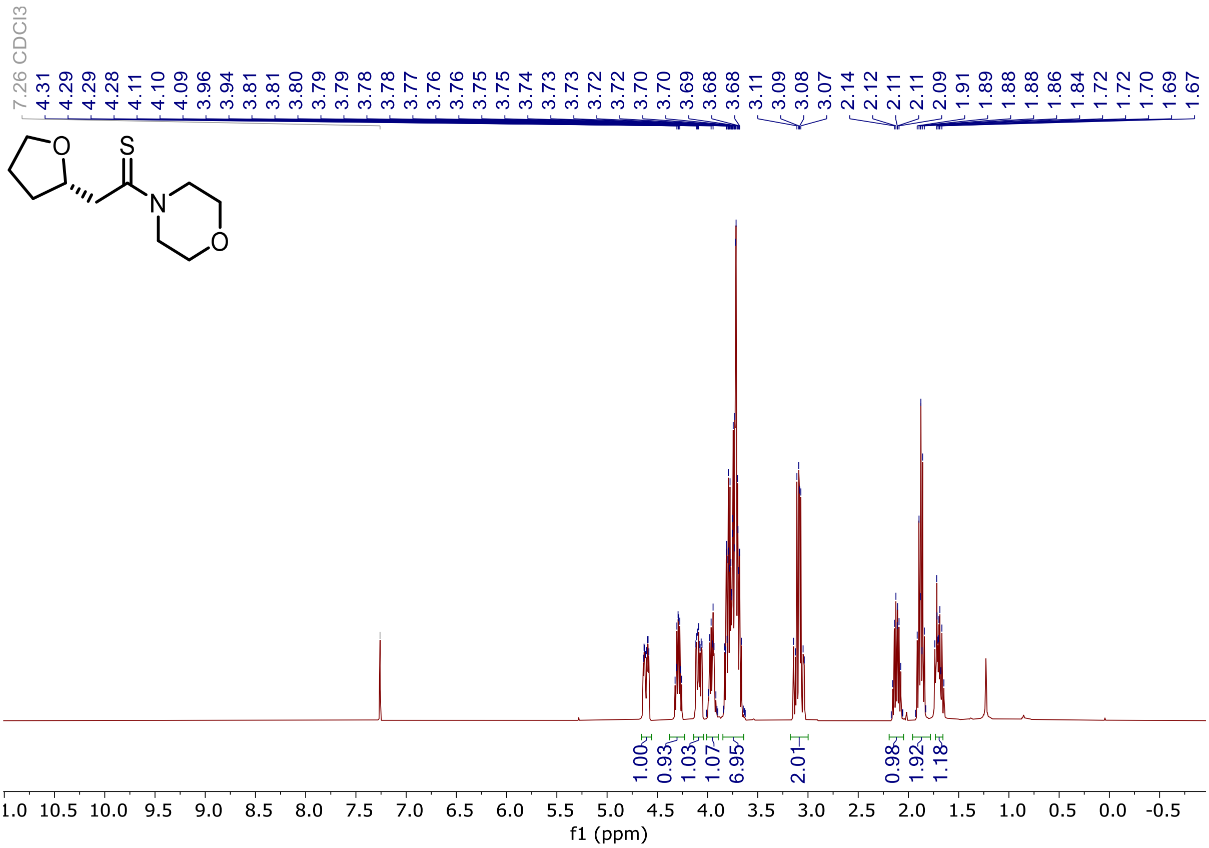

^{13}C NMR: (101 MHz, $CDCl_3$, 298K) of **5d**

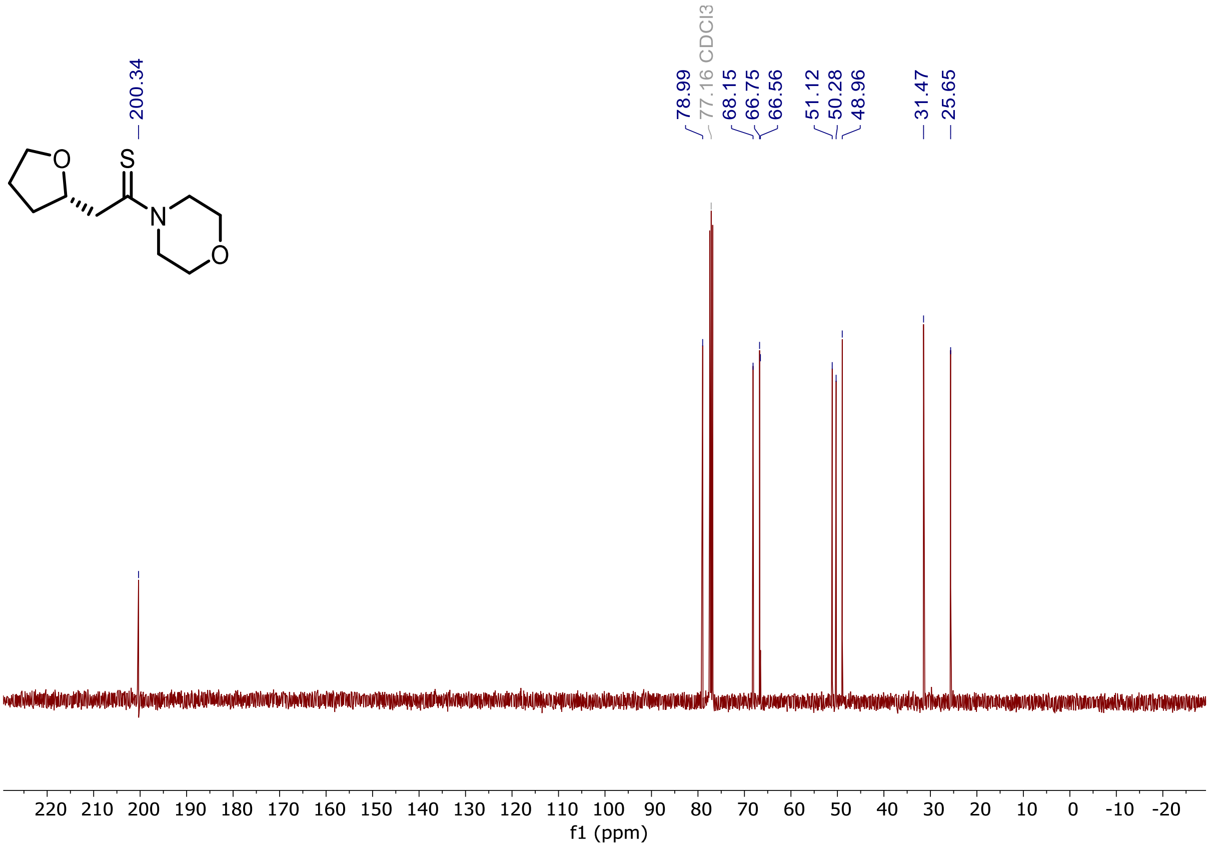

^{1}H NMR: (400 MHz, $CDCl_3$, 298K) of **5e**

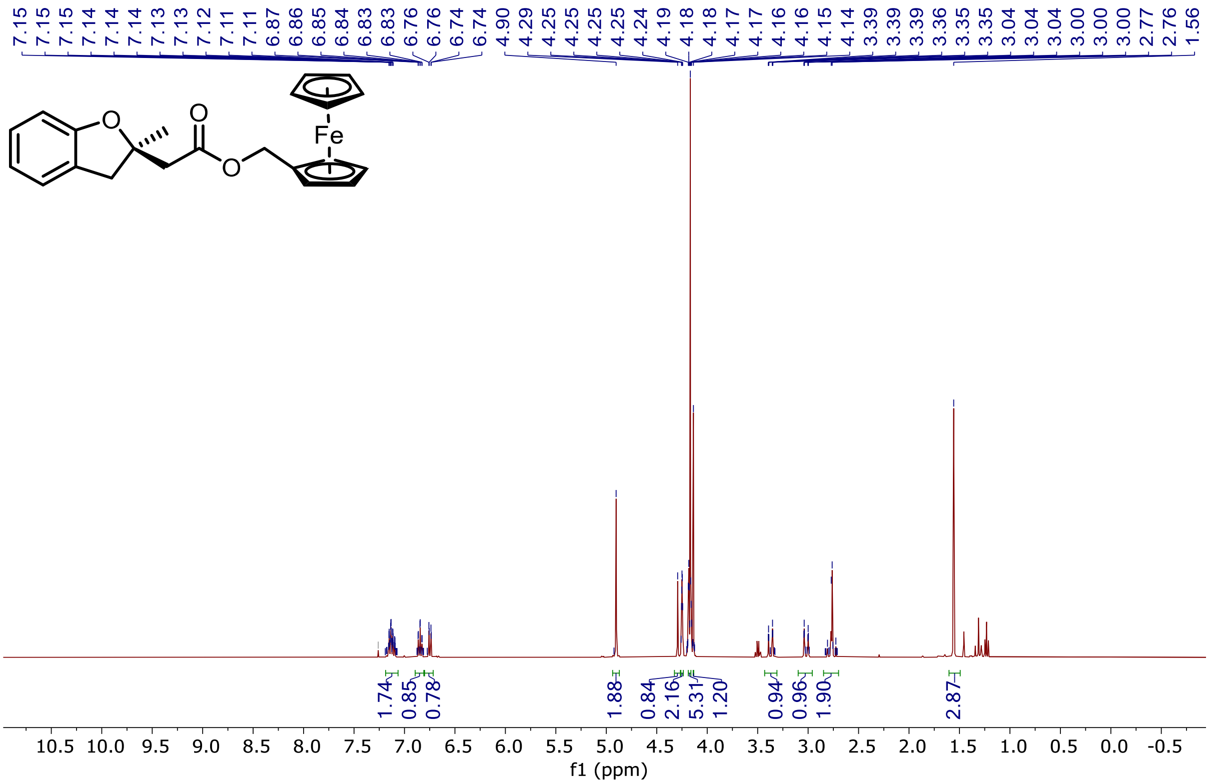

^{13}C NMR: (101 MHz, $CDCl_3$, 298K) of **5e**

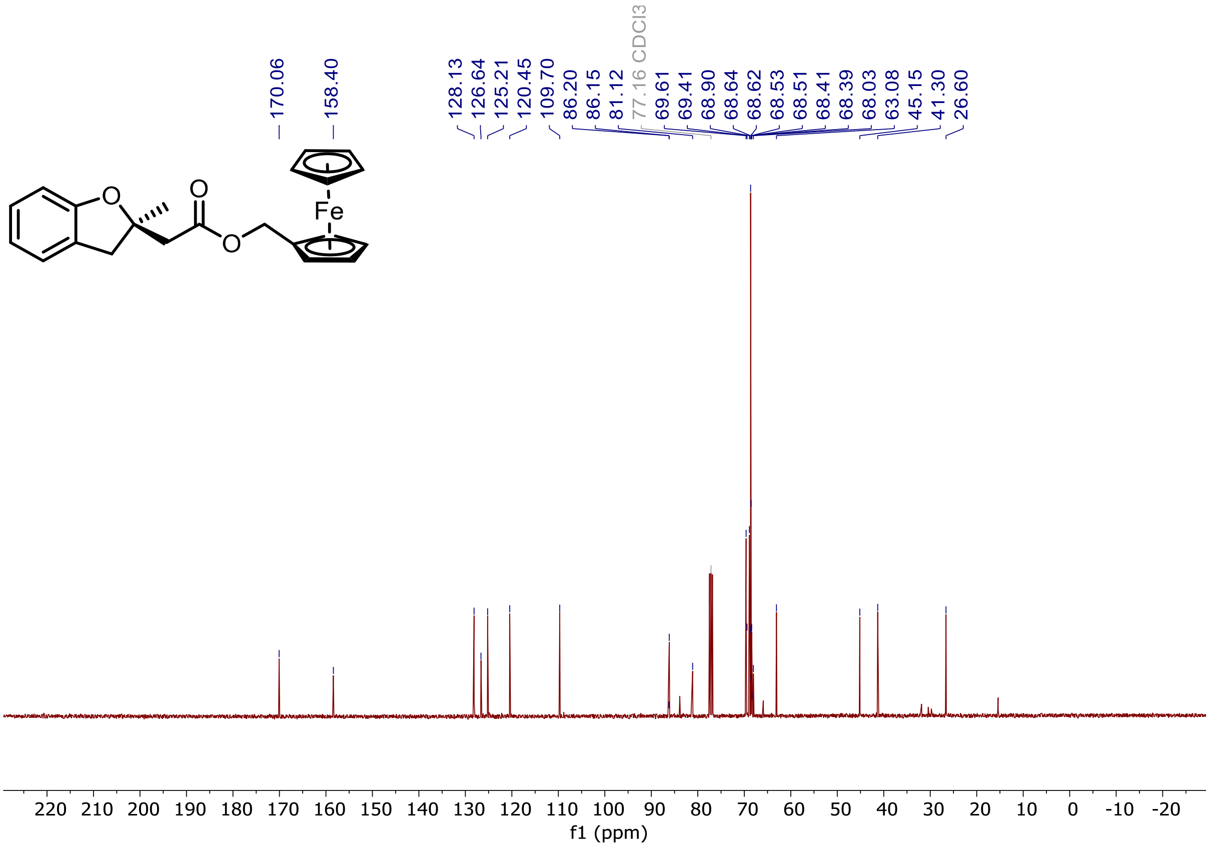

^{1}H NMR: (400 MHz, $CDCl_3$, 298K) of **5f**

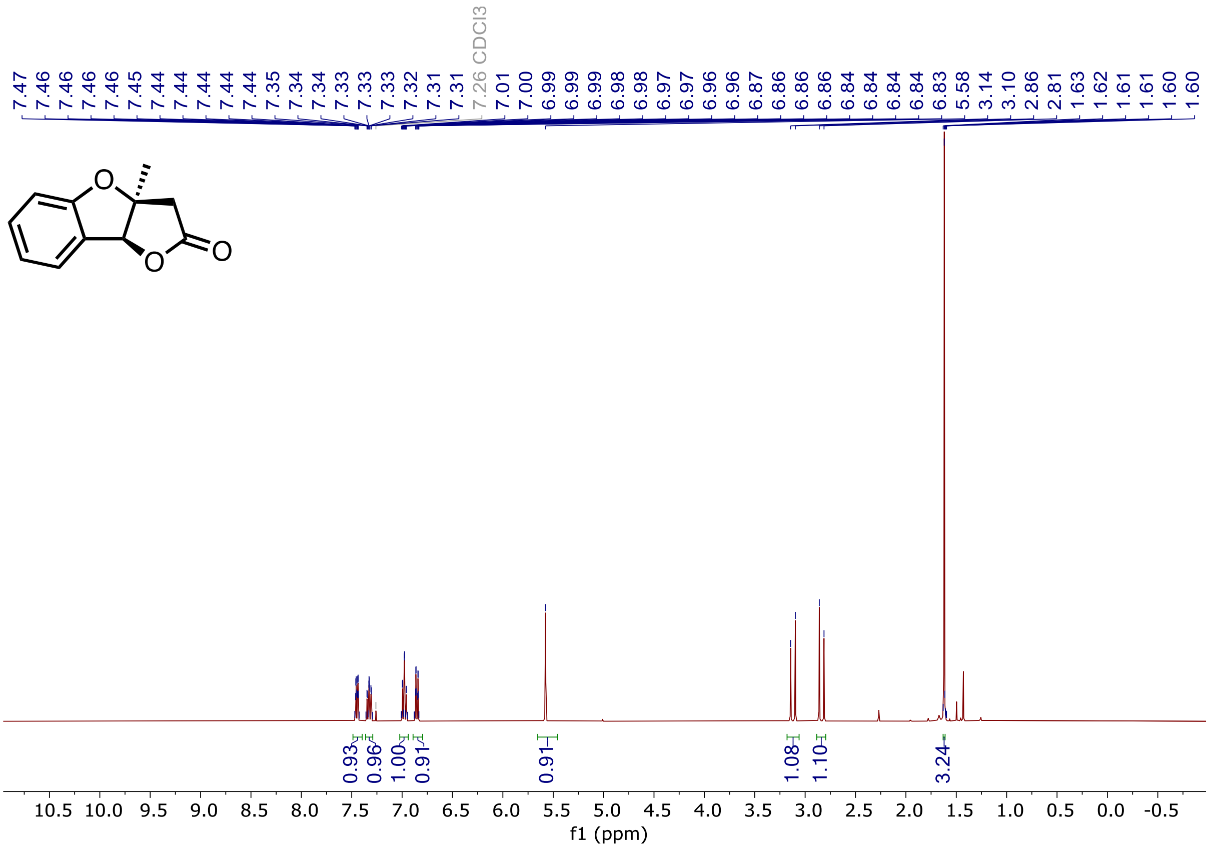

^{13}C NMR: (101 MHz, $CDCl_3$, 298K) of **5f**

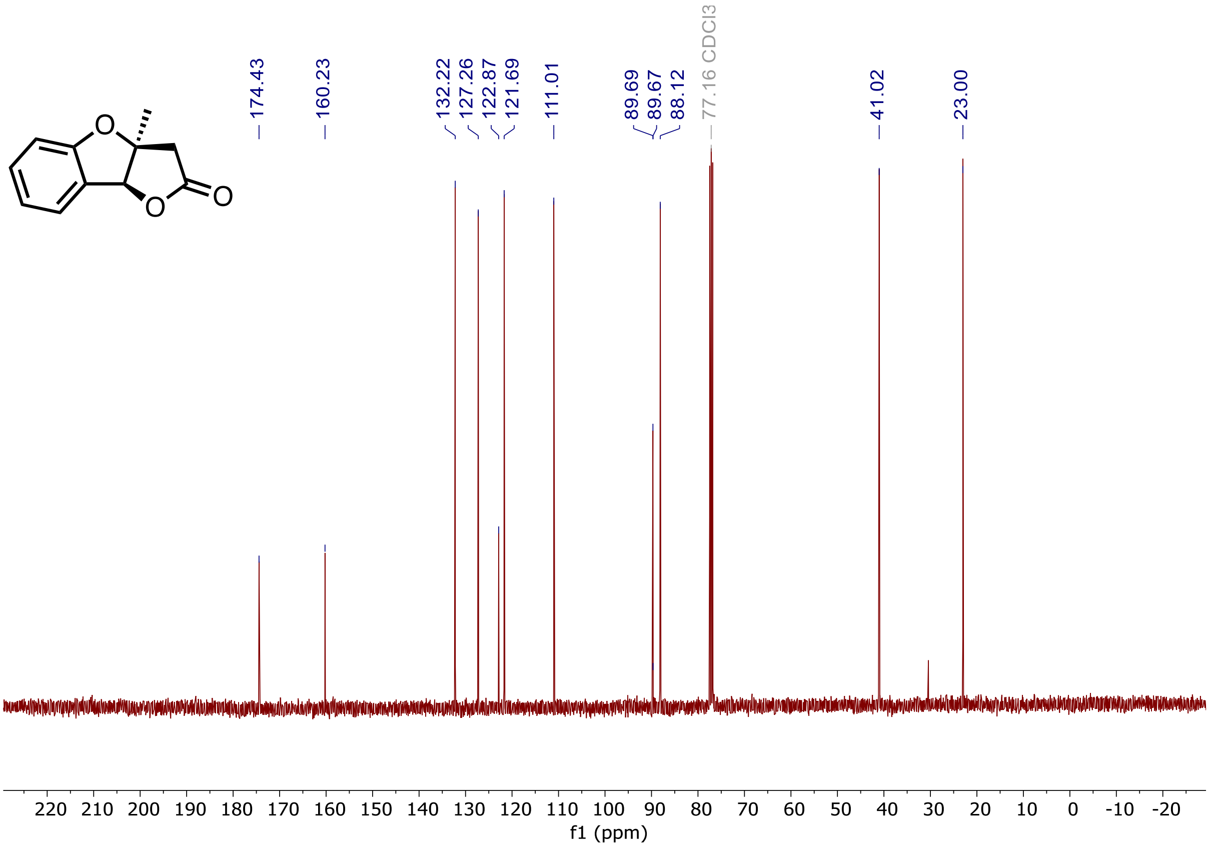

^{1}H NMR: (400 MHz, $CDCl_3$, 298K) of **5g**

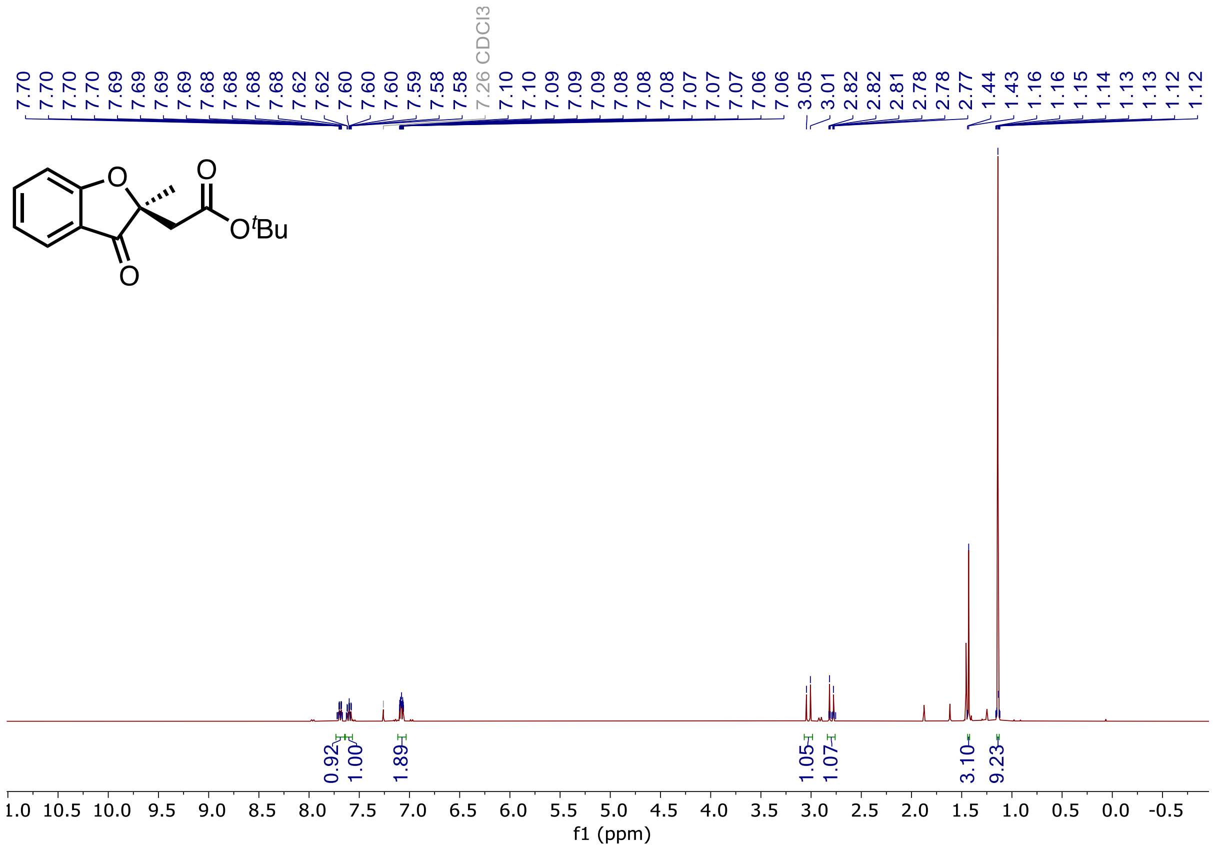

^{13}C NMR: (101 MHz, $CDCl_3$, 298K) of **5g**

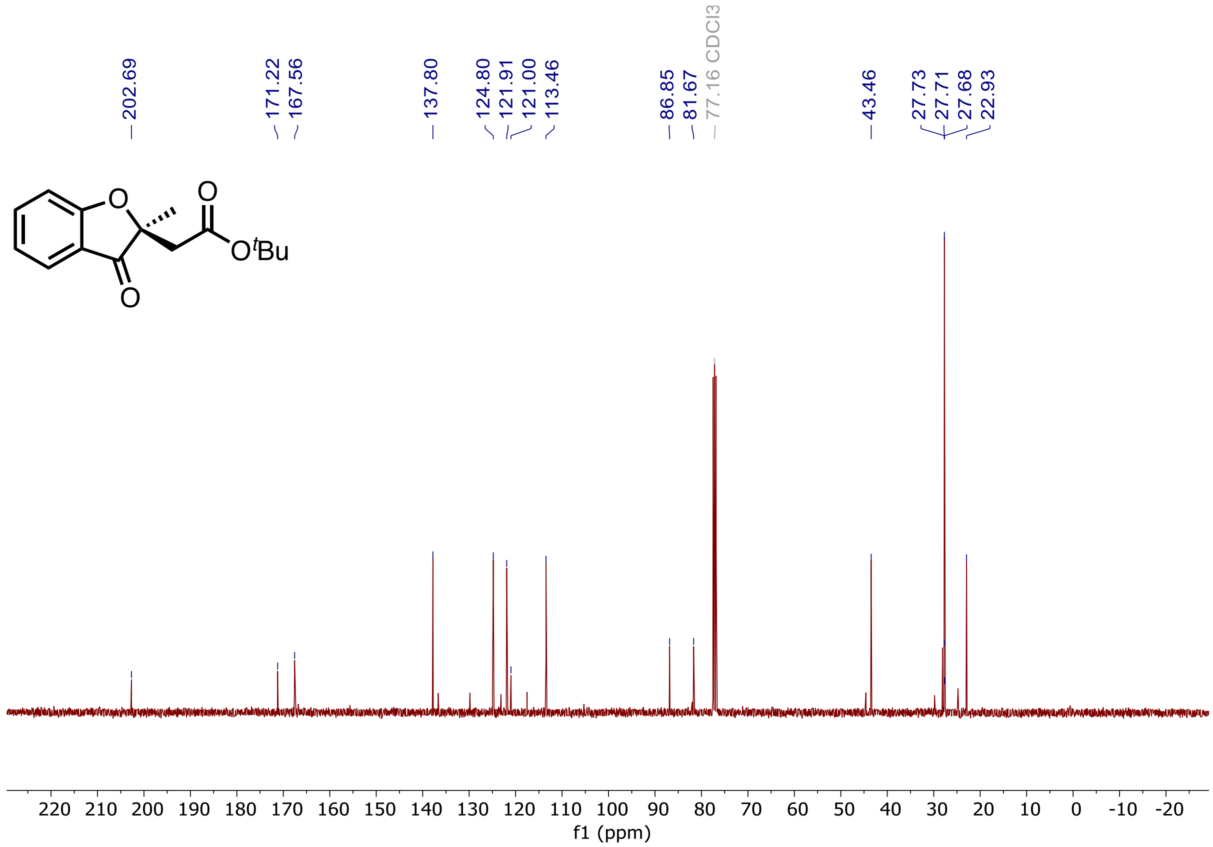

^{1}H NMR: (400 MHz, $CDCl_3$, 298K) of **5h**

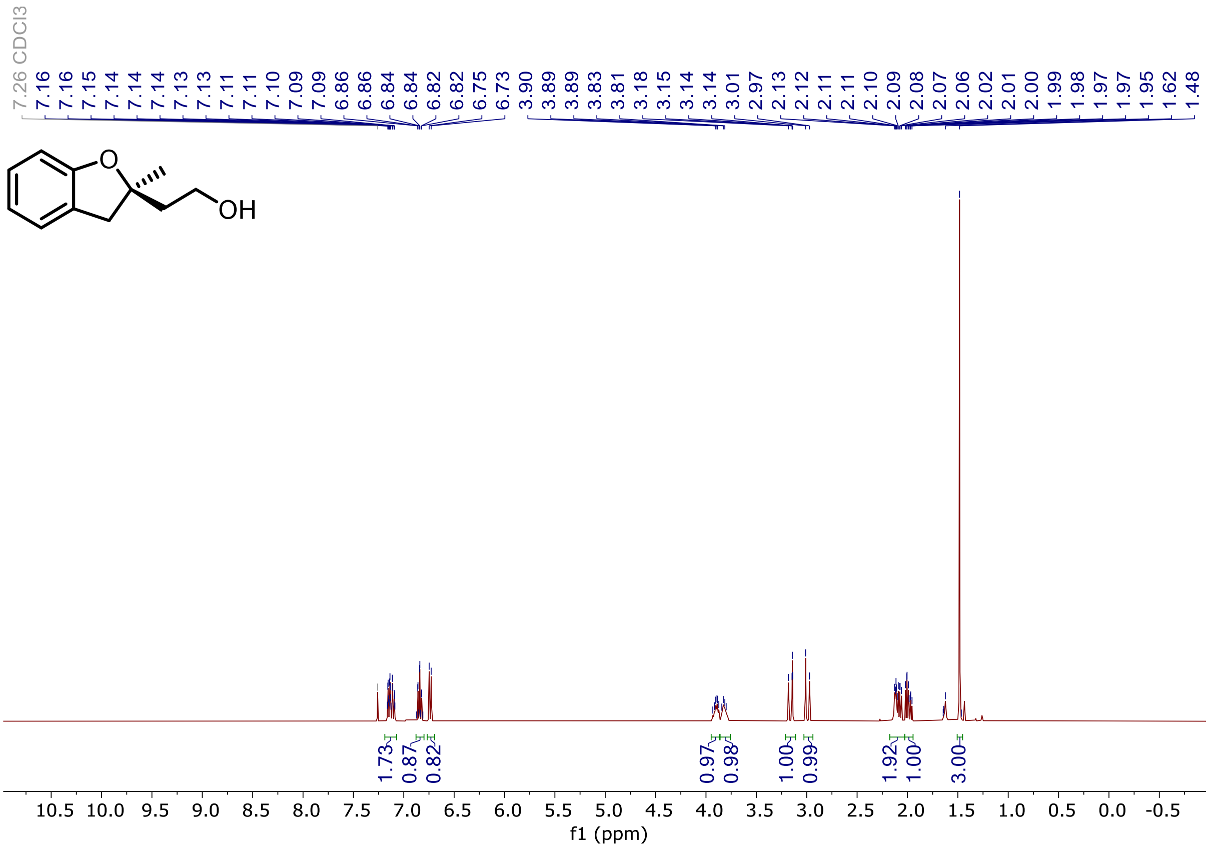

^{13}C NMR: (101 MHz, $CDCl_3$, 298K) of **5h**

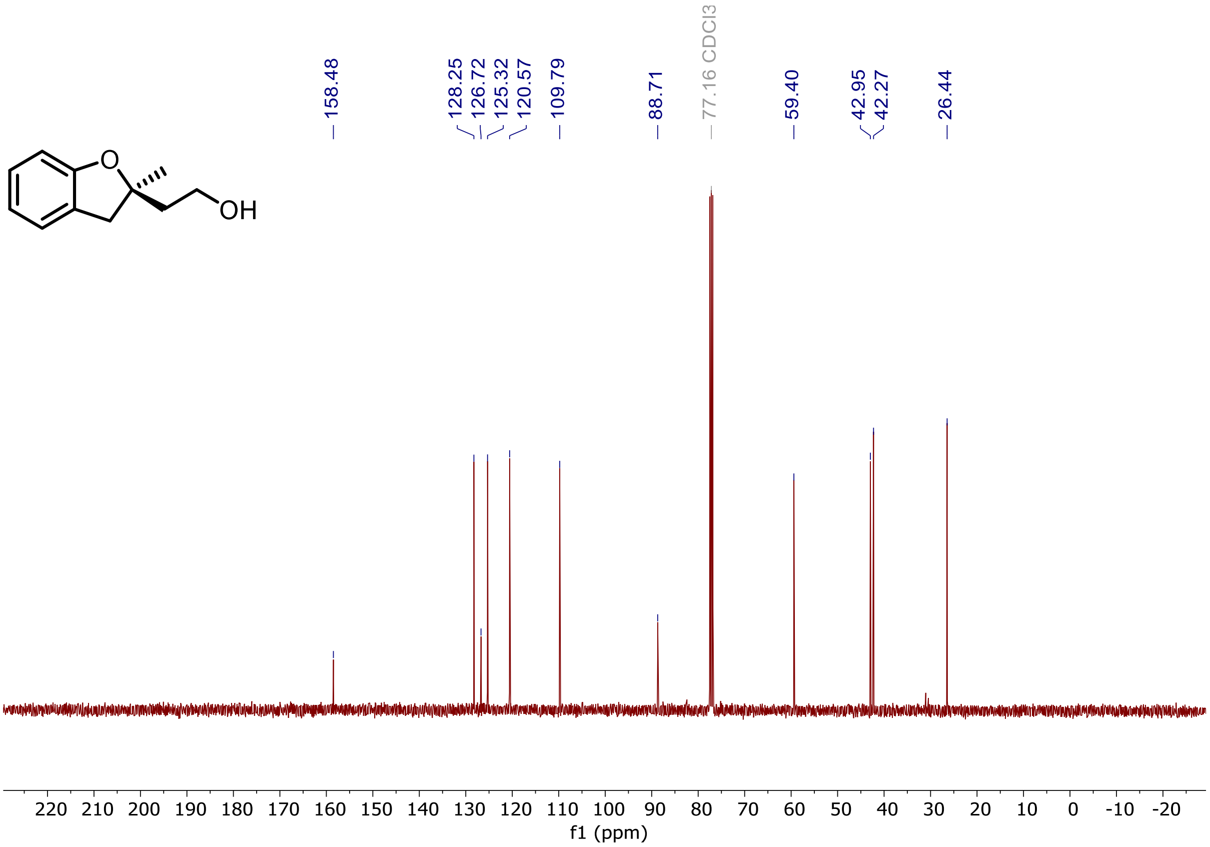

11. HPLC Traces

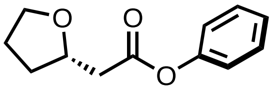

4a

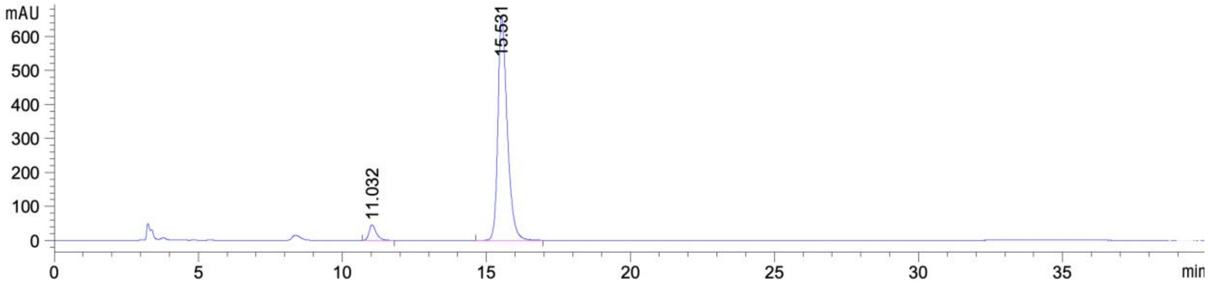

Peak #	RetTime [min]	Type	Width [min]	Area [mAU*s]	Height [mAU]	Area %
1	11.032	BB	0.2578	812.56311	46.51872	5.1595
2	15.531	BB	0.3373	1.49364e4	661.80457	94.8405

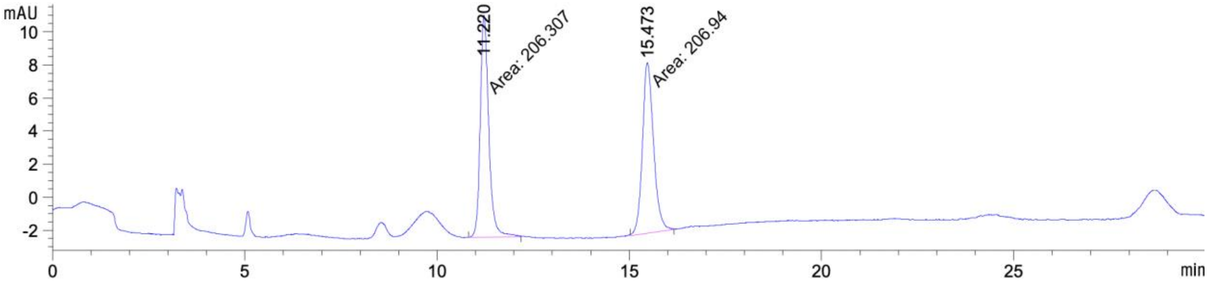

Peak #	RetTime [min]	Type	Width [min]	Area [mAU*s]	Height [mAU]	Area %
1	11.220	MM	0.2559	206.30656	13.43618	49.9233
2	15.473	MM	0.3346	206.94022	10.30703	50.0767

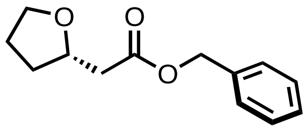

4b

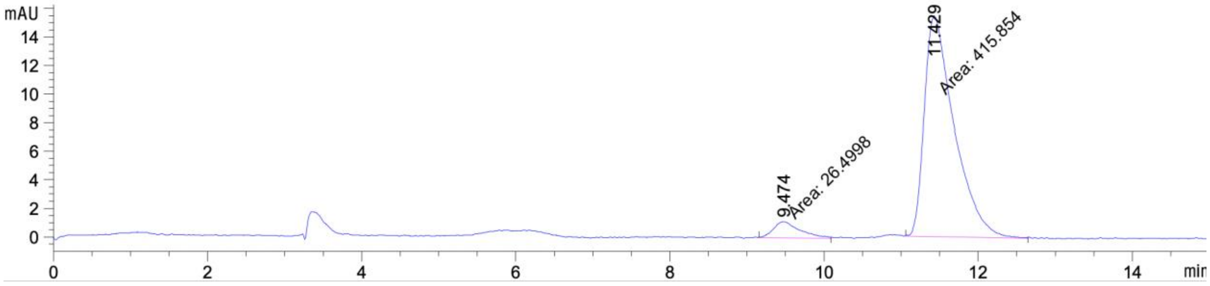

Peak #	RetTime [min]	Type	Width [min]	Area [mAU*s]	Height [mAU]	Area %
1	9.474	MM	0.3967	26.49980	1.11326	5.9906
2	11.429	MM	0.4478	415.85446	15.47890	94.0094

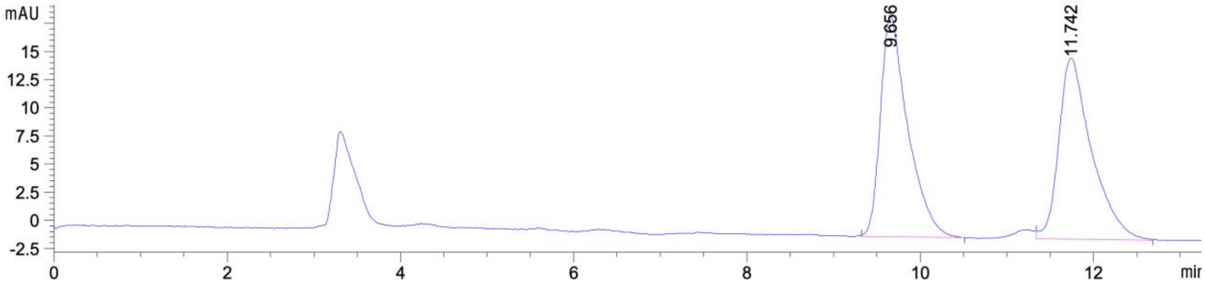

Peak #	RetTime [min]	Type	Width [min]	Area [mAU*s]	Height [mAU]	Area %
1	9.656	BB	0.3196	430.16431	19.63502	50.3959
2	11.742	VB	0.3791	423.40656	16.08400	49.6041

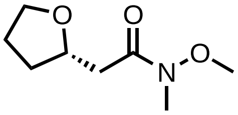

4c

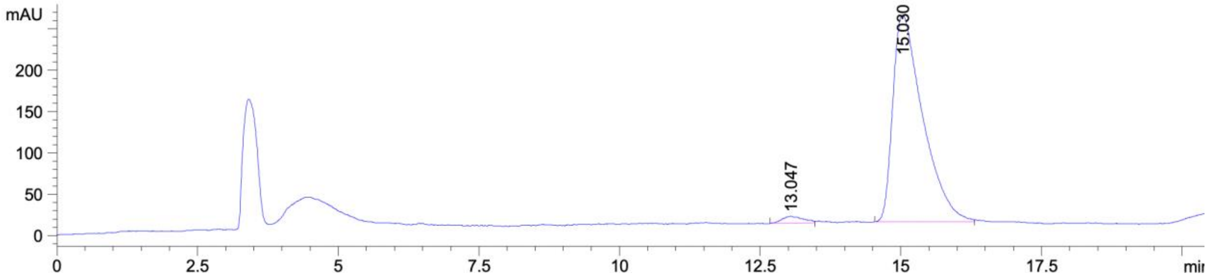

Peak #	RetTime [min]	Type	Width [min]	Area [mAU*s]	Height [mAU]	Area %
1	13.047	BB	0.3122	211.34895	8.32561	2.2300
2	15.030	BB	0.5402	9266.32910	250.13722	97.7700

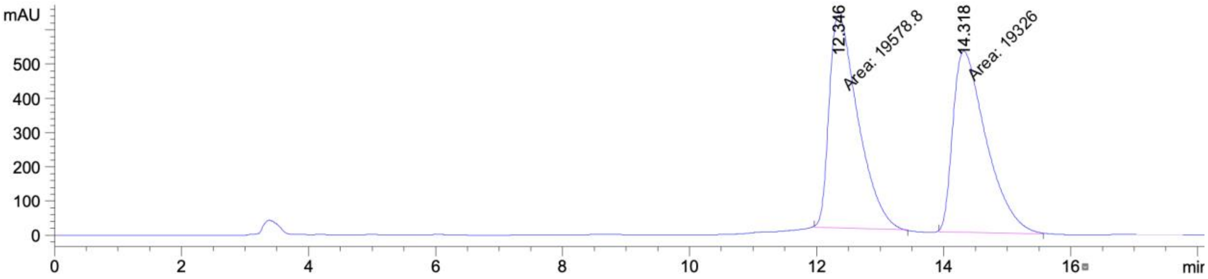

Peak #	RetTime [min]	Type	Width [min]	Area [mAU*s]	Height [mAU]	Area %
1	12.346	MM	0.5261	1.95788e4	620.21643	50.3248
2	14.318	MM	0.6085	1.93260e4	529.33557	49.6752

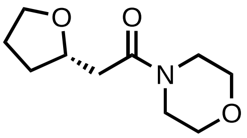

4d

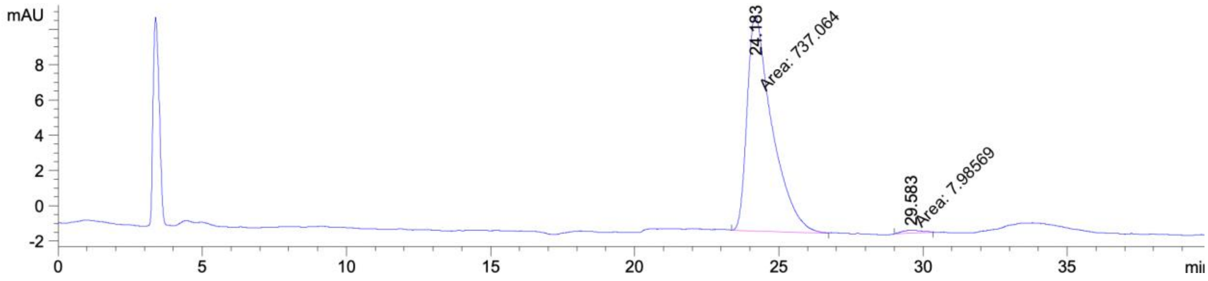

Peak #	RetTime [min]	Type	Width [min]	Area [mAU*s]	Height [mAU]	Area %
1	24.183	MM	1.0063	737.06409	12.20693	98.9282
2	29.583	MM	0.7735	7.98569	1.72060e-1	1.0718

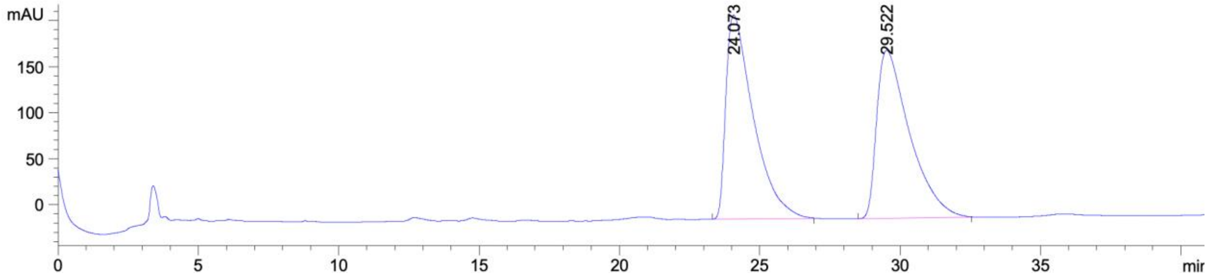

Peak #	RetTime [min]	Type	Width [min]	Area [mAU*s]	Height [mAU]	Area %
1	24.073	BB	1.0201	1.51308e4	221.75090	50.3785
2	29.522	BB	1.2045	1.49034e4	182.55247	49.6215

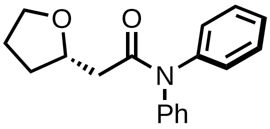

4e

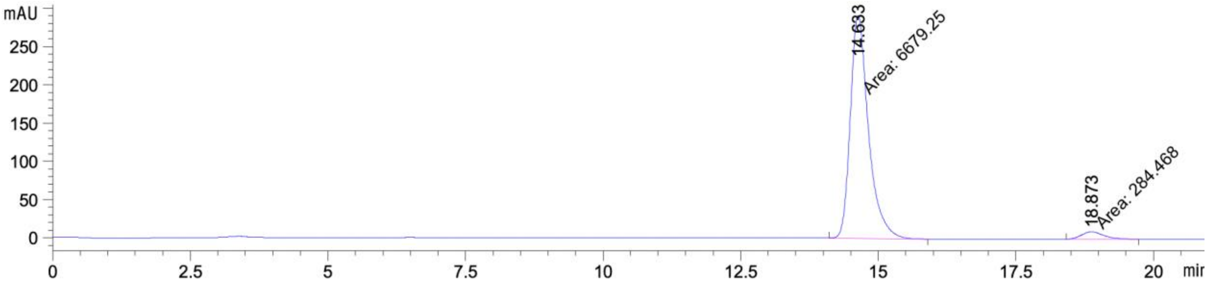

Peak #	RetTime [min]	Type	Width [min]	Area [mAU*s]	Height [mAU]	Area %
1	14.633	MM	0.3823	6679.25439	291.21591	95.9150
2	18.873	MM	0.4786	284.46783	9.90658	4.0850

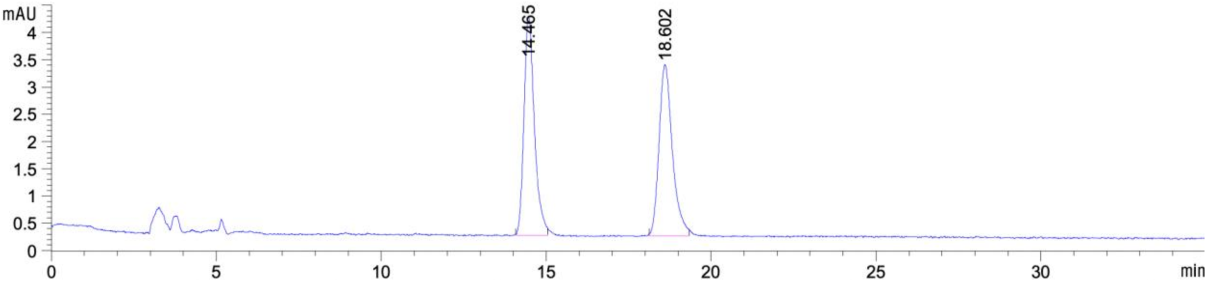

Peak #	RetTime [min]	Type	Width [min]	Area [mAU*s]	Height [mAU]	Area %
1	14.465	BB	0.3330	88.65073	4.02488	50.1595
2	18.602	BB	0.4118	88.08691	3.13511	49.8405

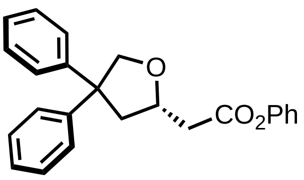

4f

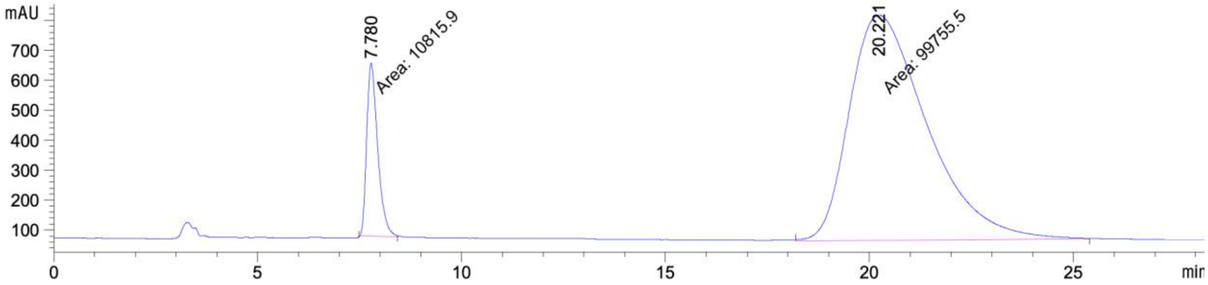

Peak #	RetTime [min]	Type	Width [min]	Area [mAU*s]	Height [mAU]	Area %
1	7.780	MM	0.3122	1.08159e4	577.47351	9.7818
2	20.221	MM	2.2100	9.97555e4	752.30743	90.2182

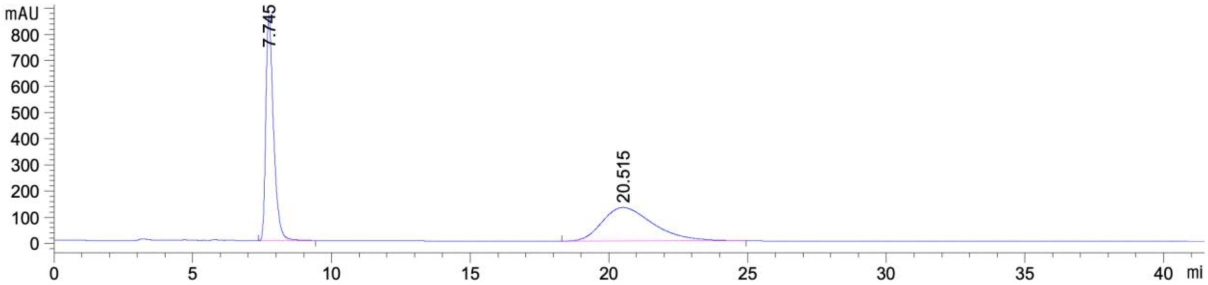

Peak #	RetTime [min]	Type	Width [min]	Area [mAU*s]	Height [mAU]	Area %
1	7.745	BB	0.2913	1.66370e4	861.60095	50.3208
2	20.515	BB	1.9245	1.64249e4	128.04399	49.6792

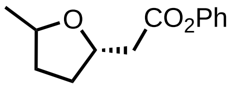

4g

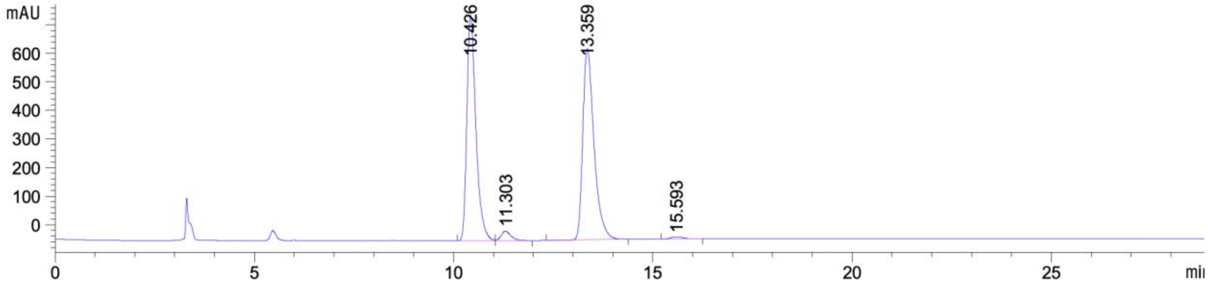

Peak #	RetTime [min]	Type	Width [min]	Area [mAU*s]	Height [mAU]	Area %
1	10.426	BV	0.2427	1.25092e4	790.04211	47.2857
2	11.303	VB	0.2576	563.59021	33.28087	2.1304
3	13.359	BB	0.3000	1.32083e4	670.02808	49.9287
4	15.593	BB	0.3376	173.32010	7.72929	0.6552

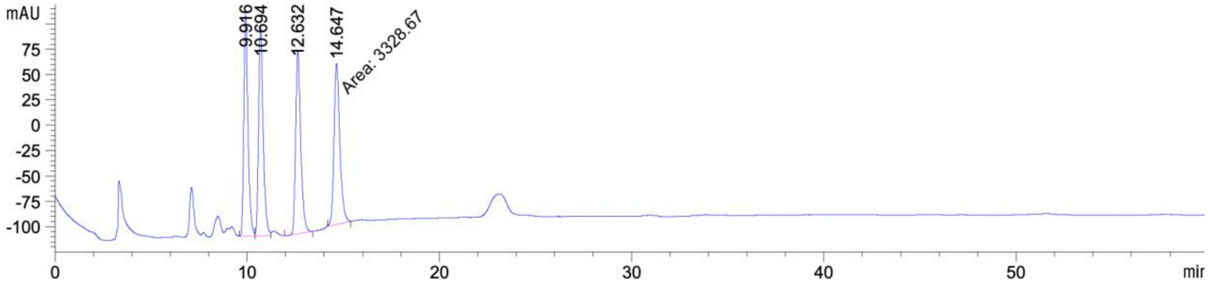

Peak #	RetTime [min]	Type	Width [min]	Area [mAU*s]	Height [mAU]	Area %
1	9.916	VV	0.2285	3297.65918	217.98547	24.6764
2	10.694	VV	0.2534	3411.34277	205.73596	25.5271
3	12.632	VB	0.2786	3325.93701	180.82787	24.8880
4	14.647	MM	0.3495	3328.66553	158.73523	24.9084

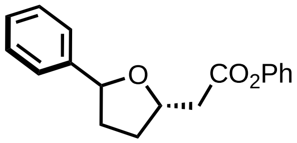

4h

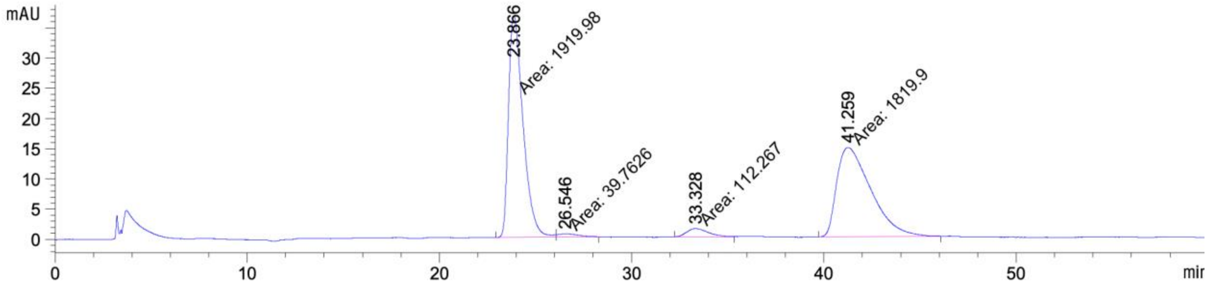

Peak #	RetTime [min]	Type	Width [min]	Area [mAU*s]	Height [mAU]	Area %
1	23.866	MF	0.8735	1919.97632	36.63522	49.3326
2	26.546	FM	1.1858	39.76258	5.58849e-1	1.0217
3	33.328	MM	1.3265	112.26680	1.41061	2.8846
4	41.259	MM	2.0568	1819.89917	14.74681	46.7611

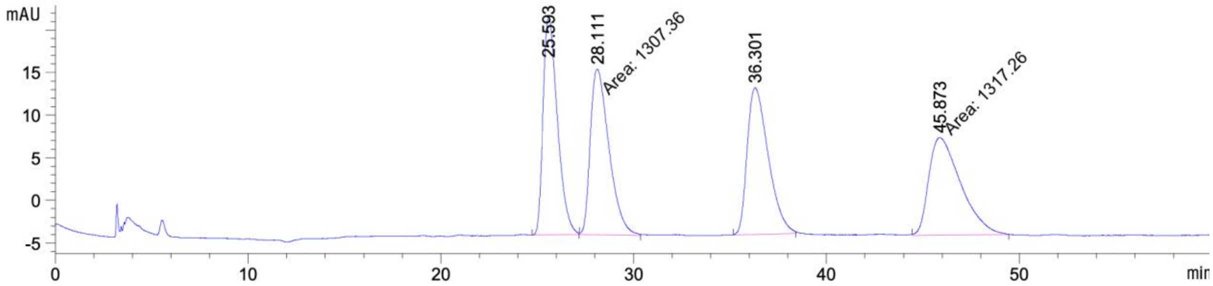

Peak #	RetTime [min]	Type	Width [min]	Area [mAU*s]	Height [mAU]	Area %
1	25.593	BB	0.7870	1326.72205	25.67041	25.2094
2	28.111	MM	1.1204	1307.35876	19.44813	24.8415
3	36.301	BB	1.1131	1311.45288	17.20872	24.9193
4	45.873	MM	1.9189	1317.26318	11.44087	25.0297

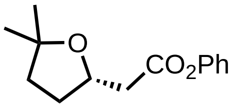

4i

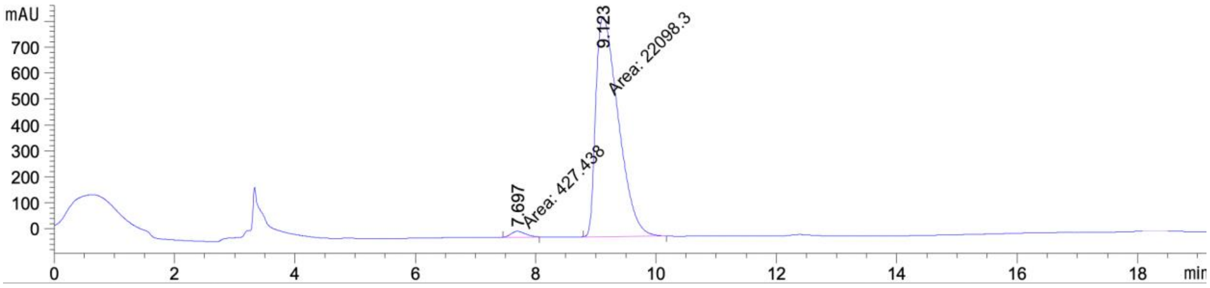

Peak #	RetTime [min]	Type	Width [min]	Area [mAU*s]	Height [mAU]	Area %
1	7.697	MM	0.2975	427.43784	23.94761	1.8976
2	9.123	MM	0.4335	2.20983e4	849.58716	98.1024

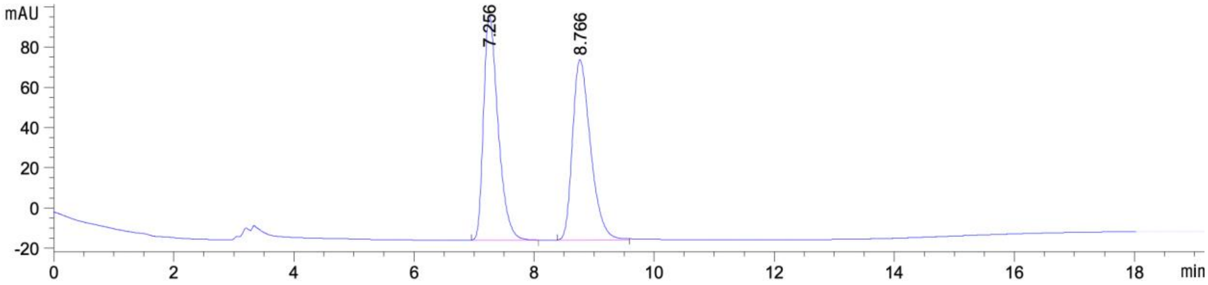

Peak #	RetTime [min]	Type	Width [min]	Area [mAU*s]	Height [mAU]	Area %
1	7.256	BB	0.2551	1869.66199	111.81320	50.0508
2	8.766	BB	0.3211	1865.86487	89.53719	49.9492

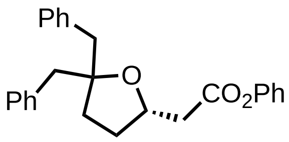

4j

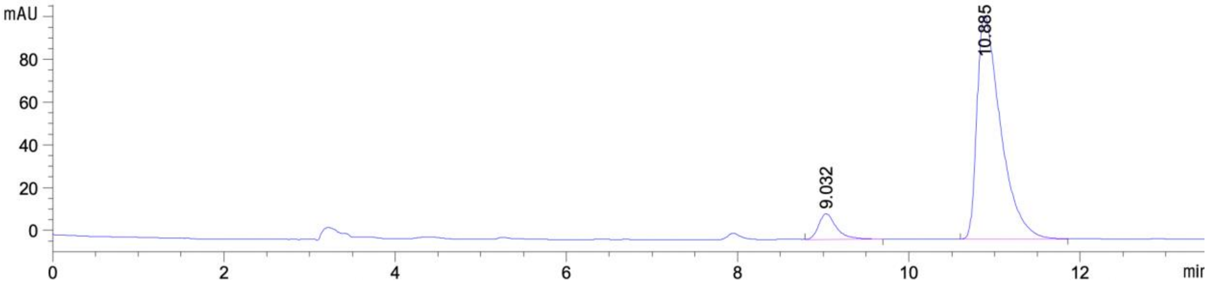

Peak #	RetTime [min]	Type	Width [min]	Area [mAU*s]	Height [mAU]	Area %
1	9.032	BB	0.2217	174.82506	12.01899	8.0282
2	10.885	BB	0.2917	2002.81482	104.44561	91.9718

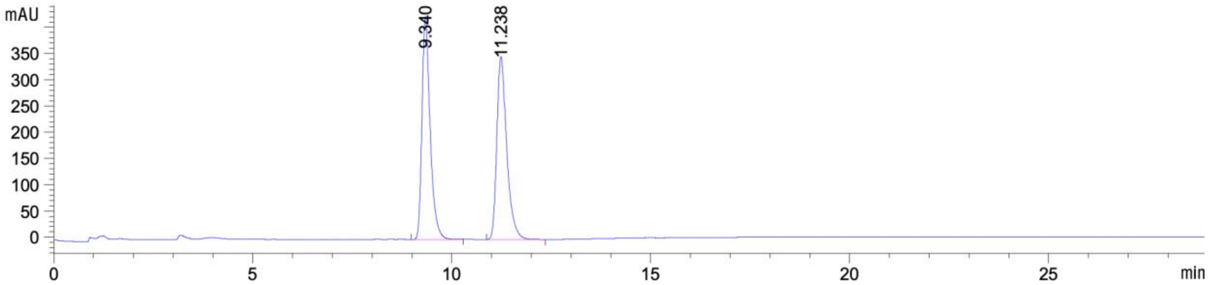

Peak #	RetTime [min]	Type	Width [min]	Area [mAU*s]	Height [mAU]	Area %
1	9.340	VB	0.2137	6036.35449	425.10031	50.1422
2	11.238	BB	0.2610	6002.11230	348.34943	49.8578

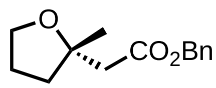

4k

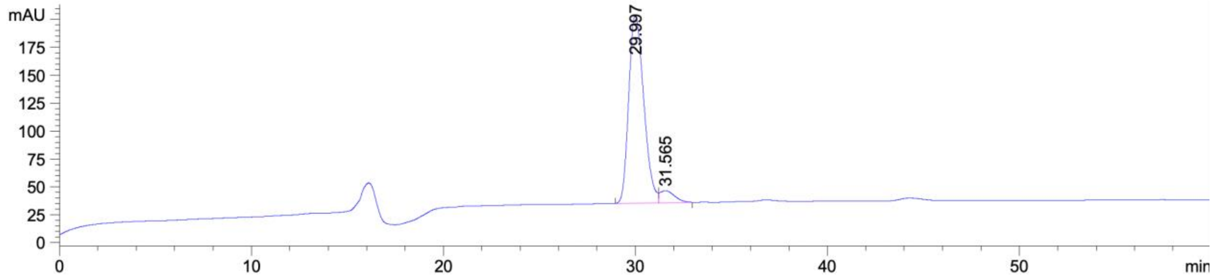

Peak #	RetTime [min]	Type	Width [min]	Area [mAU*s]	Height [mAU]	Area %
1	29.997	BV	0.8508	9164.63086	168.42009	94.0702
2	31.565	VB	0.7950	577.69922	10.81812	5.9298

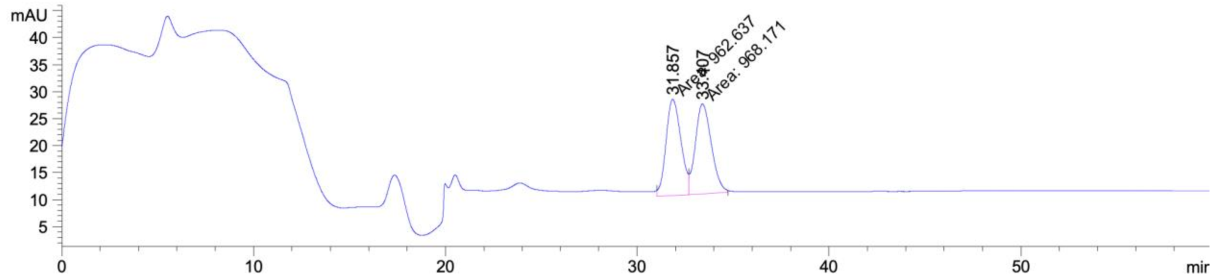

Peak #	RetTime [min]	Type	Width [min]	Area [mAU*s]	Height [mAU]	Area %
1	31.857	MF	0.9002	962.63739	17.82213	49.8567
2	33.407	FM	0.9715	968.17139	16.60993	50.1433

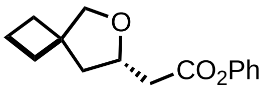

4l

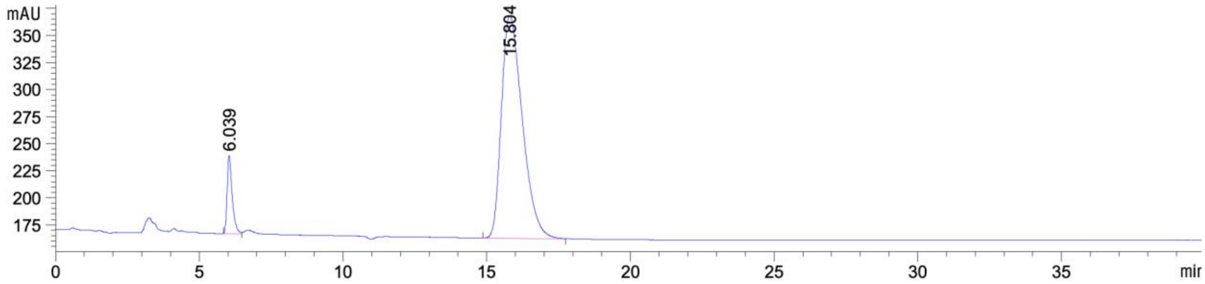

Peak #	RetTime [min]	Type	Width [min]	Area [mAU*s]	Height [mAU]	Area %
1	6.039	BV	0.1840	889.78375	72.07682	7.8771
2	15.804	BB	0.7892	1.04061e4	205.36037	92.1229

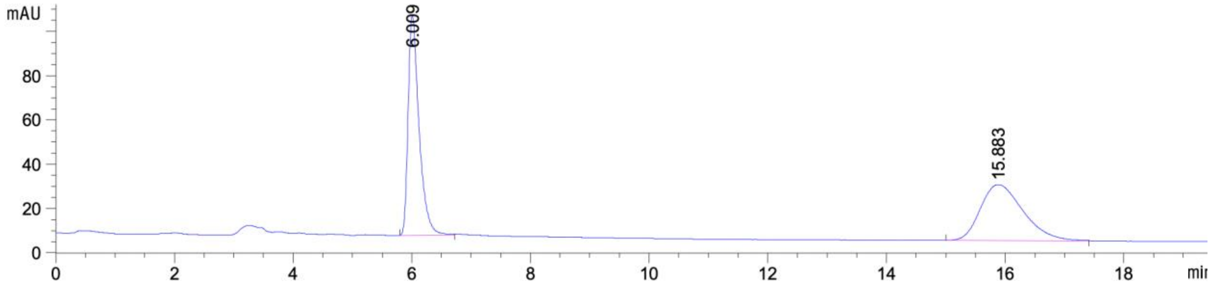

Peak #	RetTime [min]	Type	Width [min]	Area [mAU*s]	Height [mAU]	Area %
1	6.009	BB	0.1916	1274.22571	99.37149	50.1842
2	15.883	BB	0.7711	1264.87415	25.30827	49.8158

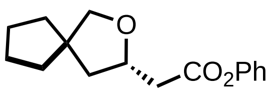

4m

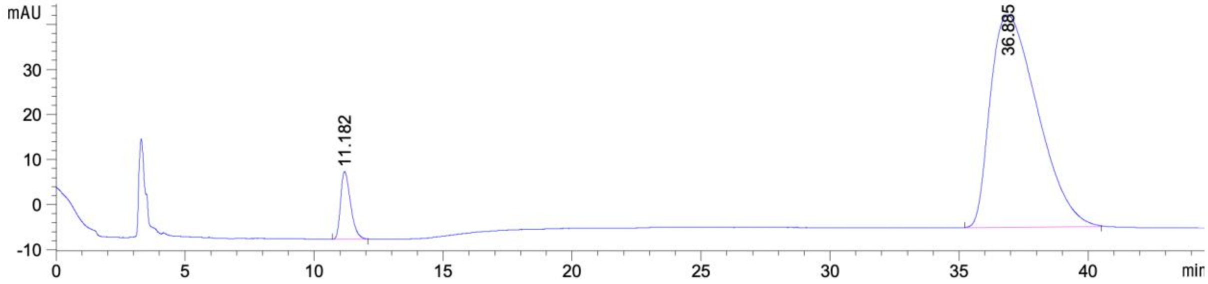

Peak #	RetTime [min]	Type	Width [min]	Area [mAU*s]	Height [mAU]	Area %
1	11.182	BB	0.4206	404.92725	15.01679	6.3277
2	36.885	BB	1.8637	5994.33691	47.13342	93.6723

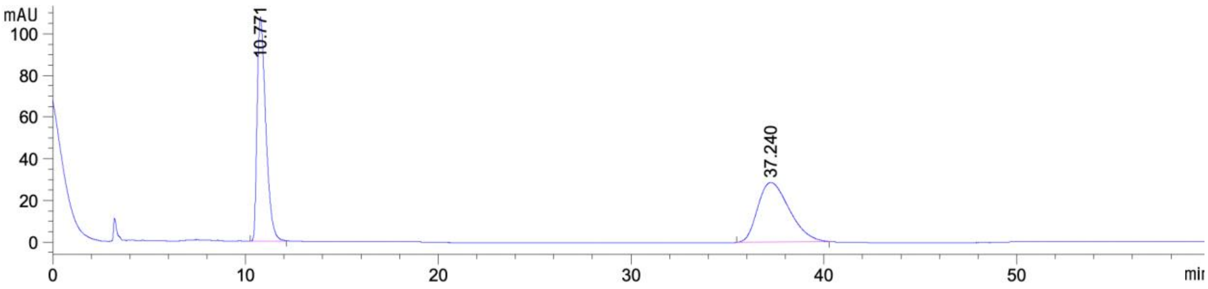

Peak #	RetTime [min]	Type	Width [min]	Area [mAU*s]	Height [mAU]	Area %
1	10.771	BB	0.4830	3370.47485	107.73637	50.6010
2	37.240	BB	1.6395	3290.41675	28.74343	49.3990

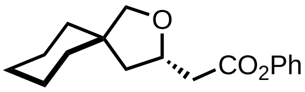

4n

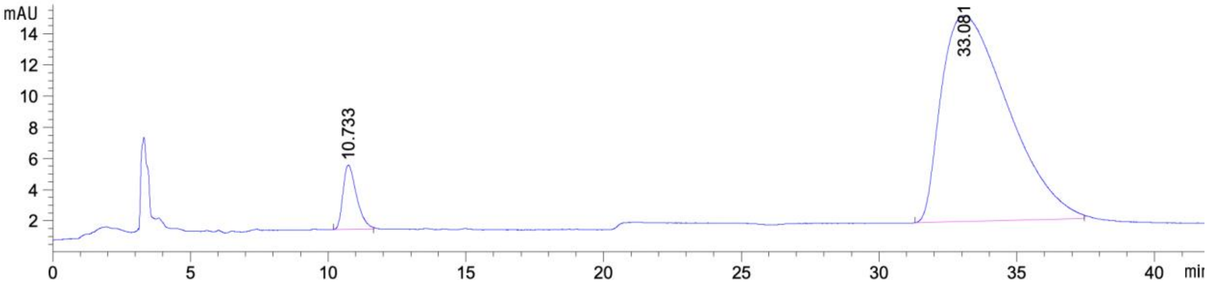

Peak #	RetTime [min]	Type	Width [min]	Area [mAU*s]	Height [mAU]	Area %
1	10.733	BB	0.5211	141.72000	4.12253	5.9757
2	33.081	BB	2.0673	2229.87427	13.22403	94.0243

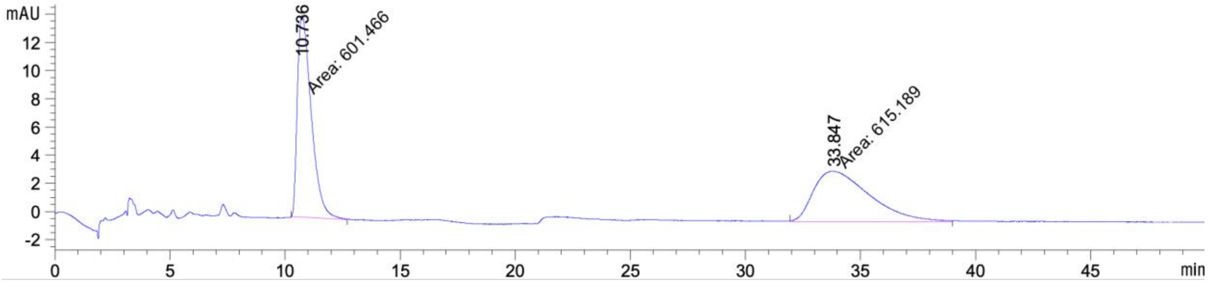

Peak #	RetTime [min]	Type	Width [min]	Area [mAU*s]	Height [mAU]	Area %
1	10.736	MM	0.7006	601.46625	14.30866	49.4361
2	33.847	MM	2.8565	615.18866	3.58939	50.5639

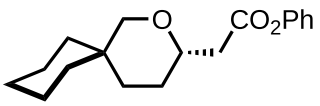

4o

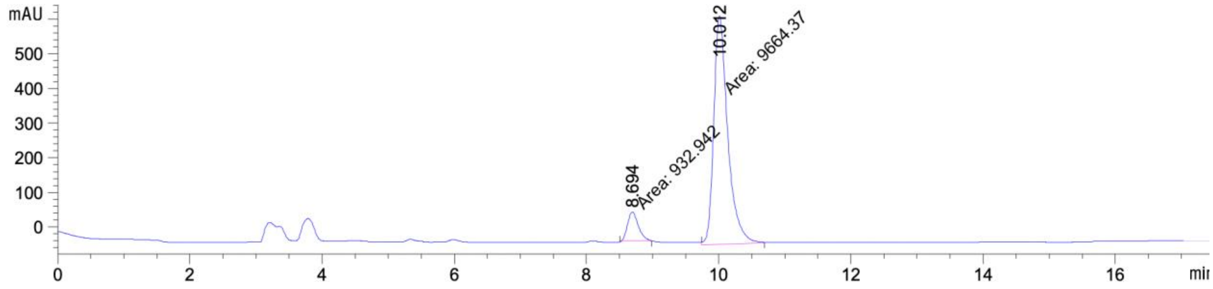

Peak #	RetTime [min]	Type	Width [min]	Area [mAU*s]	Height [mAU]	Area %
1	8.694	MM	0.1866	932.94232	83.31873	8.8036
2	10.012	MM	0.2444	9664.37305	659.11115	91.1964

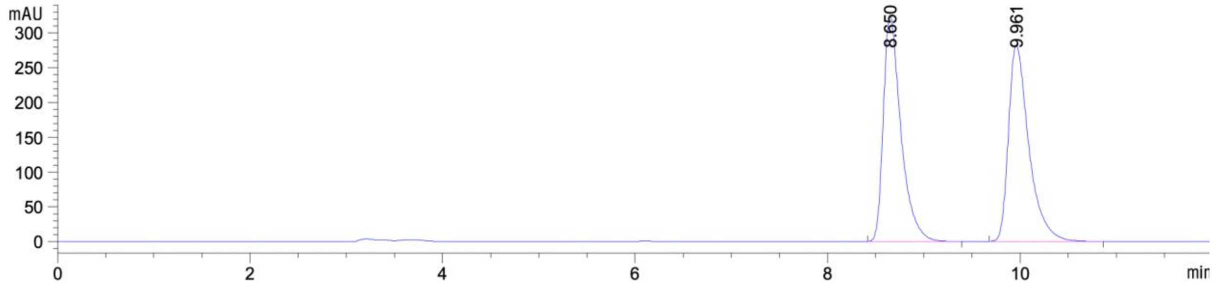

Peak #	RetTime [min]	Type	Width [min]	Area [mAU*s]	Height [mAU]	Area %
1	8.650	BB	0.1924	4132.32959	324.86829	49.8538
2	9.961	BB	0.2214	4156.55908	282.94901	50.1462

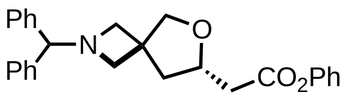

4p

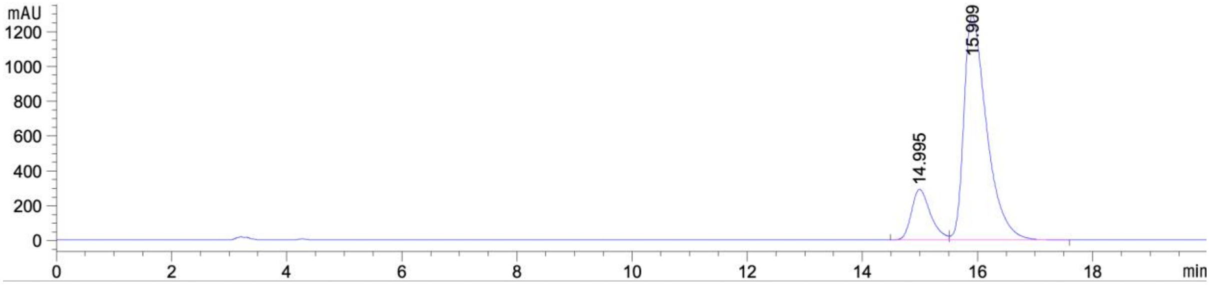

Peak #	RetTime [min]	Type	Width [min]	Area [mAU*s]	Height [mAU]	Area %
1	14.995	BV	0.3506	6676.46973	290.00919	15.8851
2	15.909	VB	0.4162	3.53533e4	1288.24622	84.1149

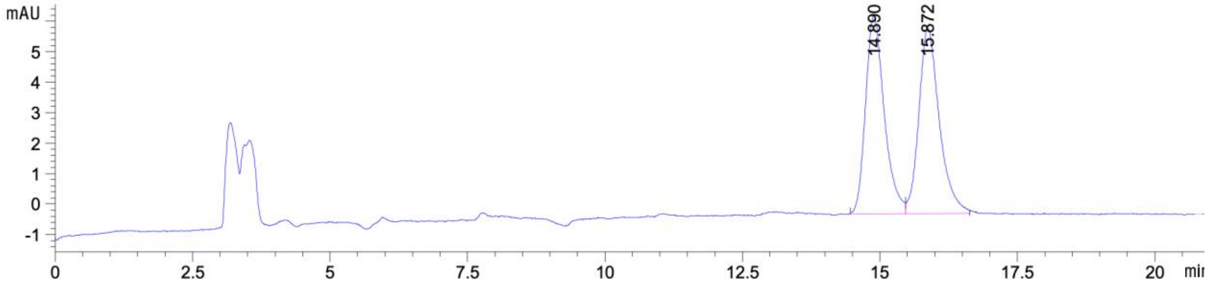

Peak #	RetTime [min]	Type	Width [min]	Area [mAU*s]	Height [mAU]	Area %
1	14.890	BV	0.3566	154.87320	6.53120	49.3708
2	15.872	VB	0.4006	158.82079	6.08343	50.6292

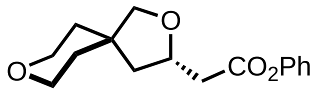

4q

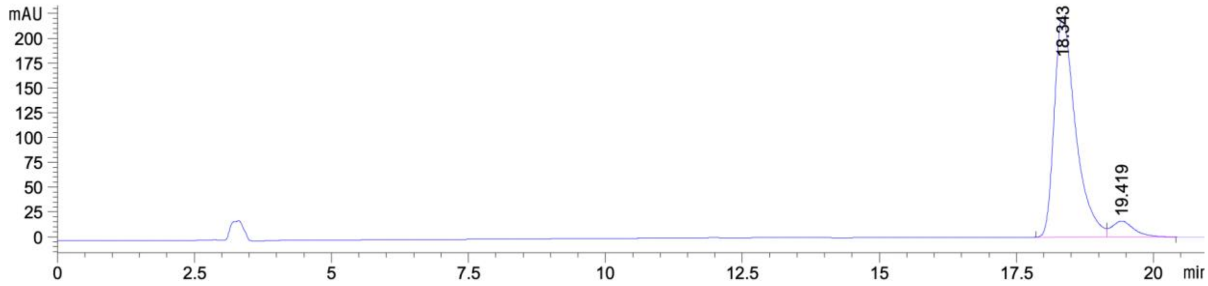

Peak #	RetTime [min]	Type	Width [min]	Area [mAU*s]	Height [mAU]	Area %
1	18.343	BV	0.4084	6038.37451	222.67833	92.7107
2	19.419	VB	0.4246	474.76178	16.35882	7.2893

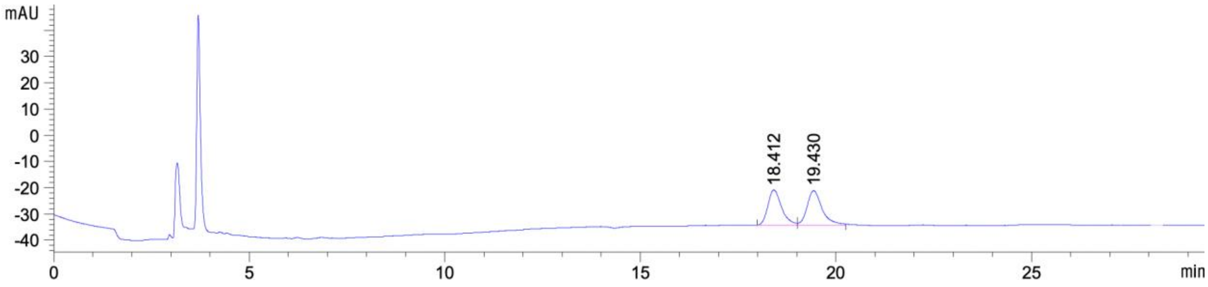

Peak #	RetTime [min]	Type	Width [min]	Area [mAU*s]	Height [mAU]	Area %
1	18.412	BV	0.3740	329.80307	13.45267	48.3033
2	19.430	VB	0.3962	352.97235	13.18843	51.6967

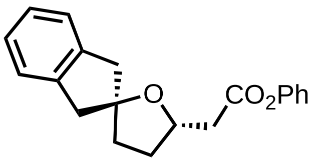

4r

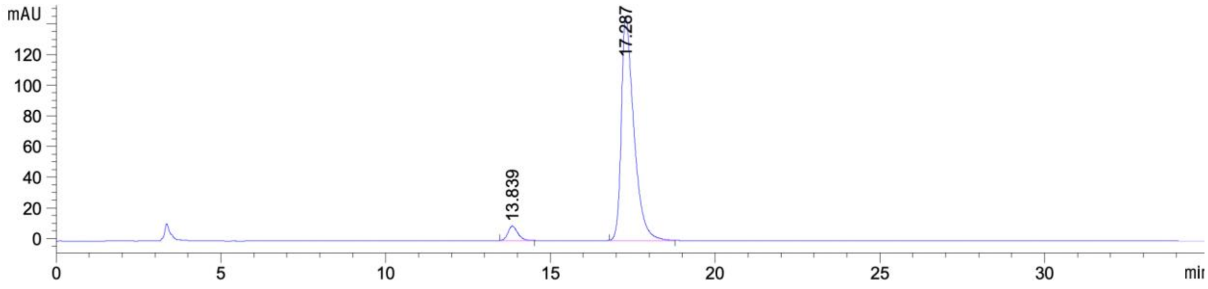

Peak #	RetTime [min]	Type	Width [min]	Area [mAU*s]	Height [mAU]	Area %
1	13.839	BB	0.3159	199.85324	9.48337	4.8999
2	17.287	BB	0.3912	3878.88403	146.35667	95.1001

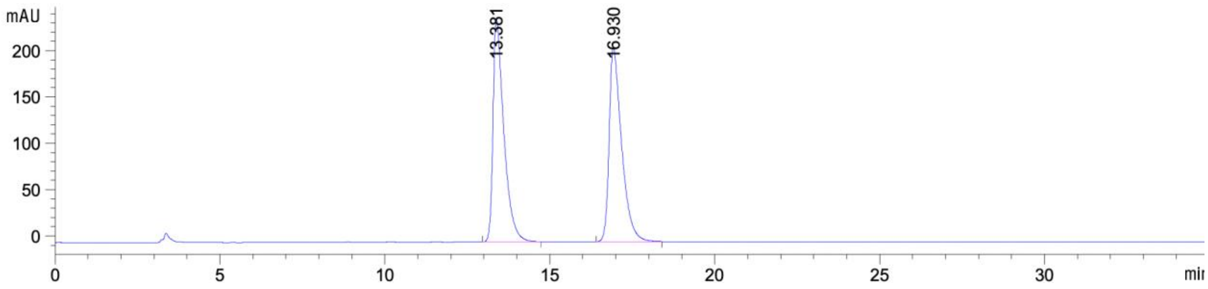

Peak #	RetTime [min]	Type	Width [min]	Area [mAU*s]	Height [mAU]	Area %
1	13.381	BB	0.3313	5498.09814	241.74213	49.9378
2	16.930	BB	0.3858	5511.79785	210.30171	50.0622

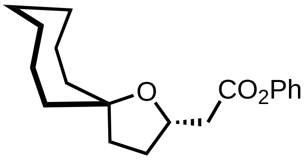

4s

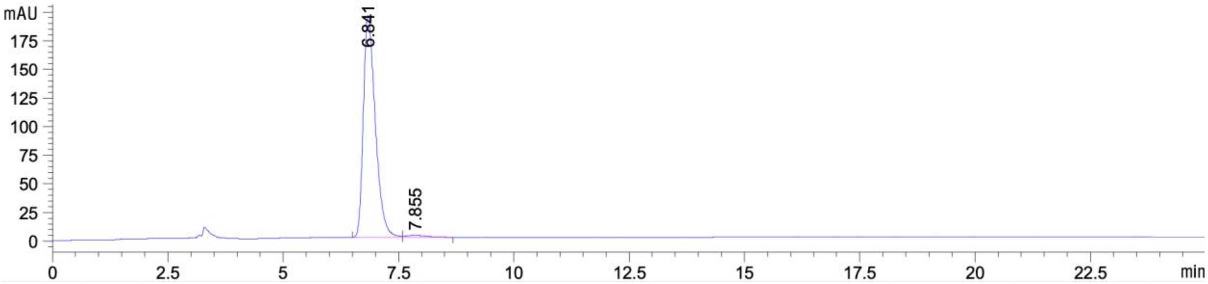

Peak #	RetTime [min]	Type	Width [min]	Area [mAU*s]	Height [mAU]	Area %
1	6.841	BV	0.2749	3505.45801	193.87785	98.3492
2	7.855	VB	0.4455	58.83789	1.80891	1.6508

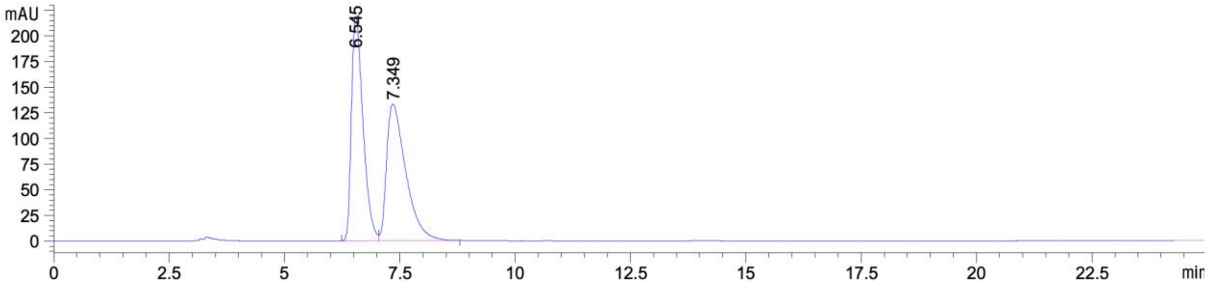

Peak #	RetTime [min]	Type	Width [min]	Area [mAU*s]	Height [mAU]	Area %
1	6.545	BV	0.2679	3827.83521	218.97209	49.6824
2	7.349	VB	0.4393	3876.78149	133.31686	50.3176

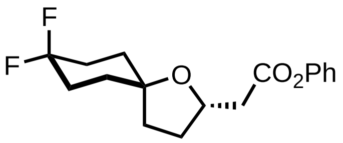

4t

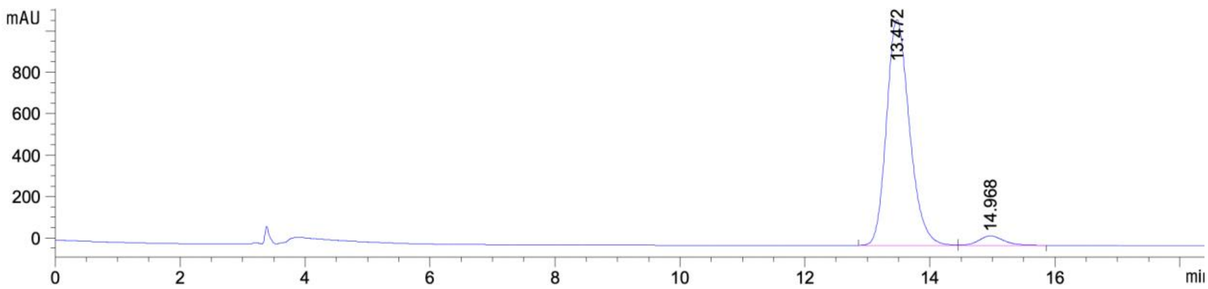

Peak #	RetTime [min]	Type	Width [min]	Area [mAU*s]	Height [mAU]	Area %
1	13.472	BV	0.3942	2.78296e4	1088.67493	95.7448
2	14.968	VB	0.4196	1236.82214	45.15331	4.2552

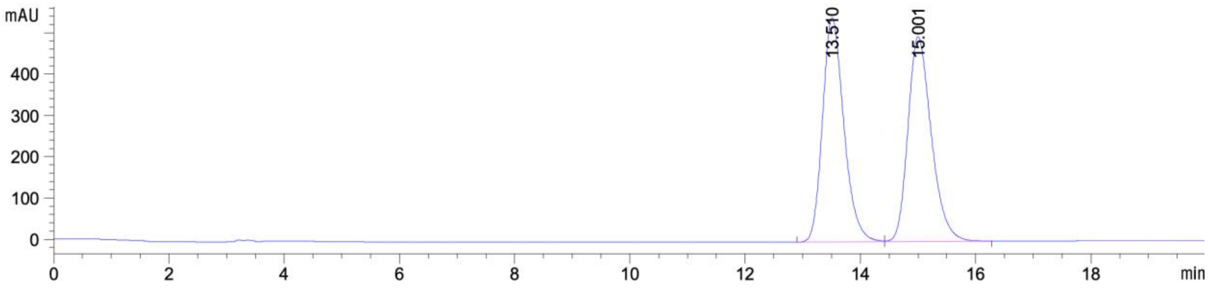

Peak #	RetTime [min]	Type	Width [min]	Area [mAU*s]	Height [mAU]	Area %
1	13.510	BV	0.3960	1.39687e4	543.24188	49.9484
2	15.001	VB	0.4331	1.39976e4	496.21054	50.0516

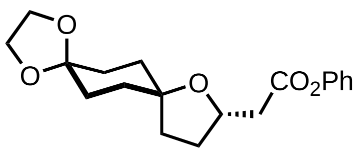

4u

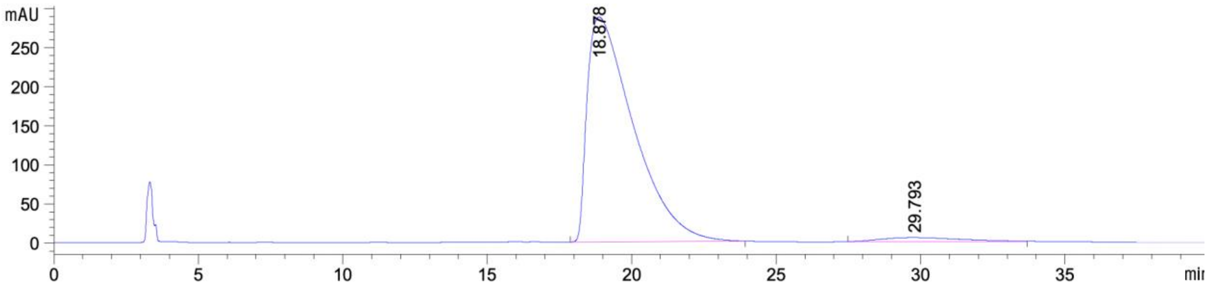

Peak #	RetTime [min]	Type	Width [min]	Area [mAU*s]	Height [mAU]	Area %
1	18.878	BB	1.6527	3.20739e4	287.67780	97.0253
2	29.793	BB	2.2681	983.36536	5.10330	2.9747

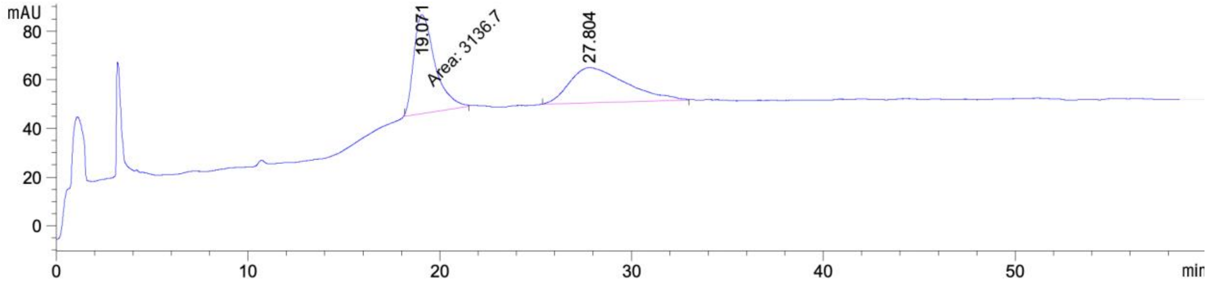

Peak #	RetTime [min]	Type	Width [min]	Area [mAU*s]	Height [mAU]	Area %
1	19.071	MM	1.2888	3136.69751	40.56311	51.1581
2	27.804	BB	2.4184	2994.67993	14.53082	48.8419

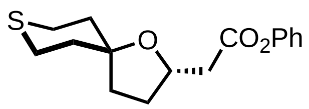

4v

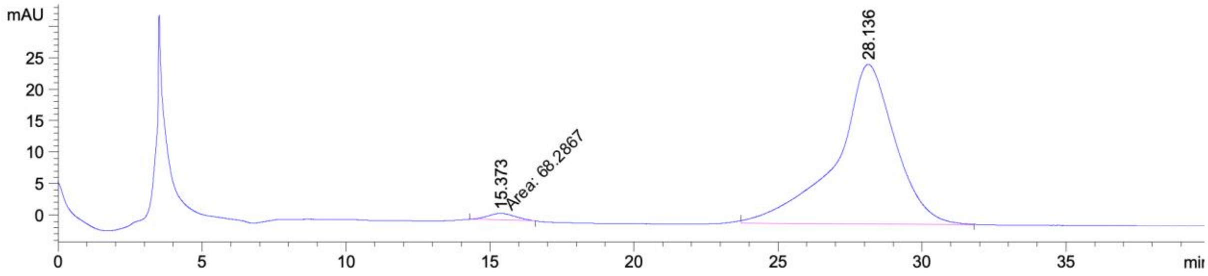

Peak #	RetTime [min]	Type	Width [min]	Area [mAU*s]	Height [mAU]	Area %
1	15.373	MM	1.0937	68.28665	1.04062	1.8630
2	28.136	BB	1.9935	3597.10205	25.31894	98.1370

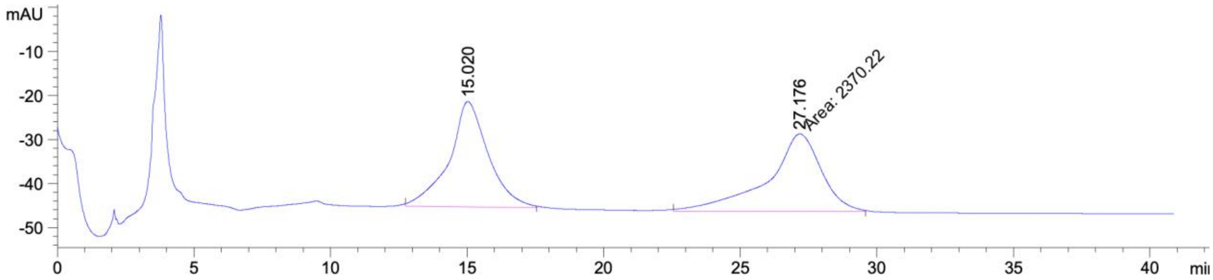

Peak #	RetTime [min]	Type	Width [min]	Area [mAU*s]	Height [mAU]	Area %
1	15.020	BB	1.4013	2367.48584	23.86714	49.9711
2	27.176	MM	2.2583	2370.22021	17.49274	50.0289

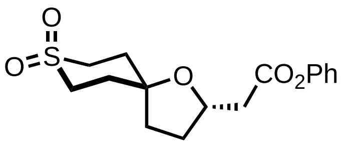

4w

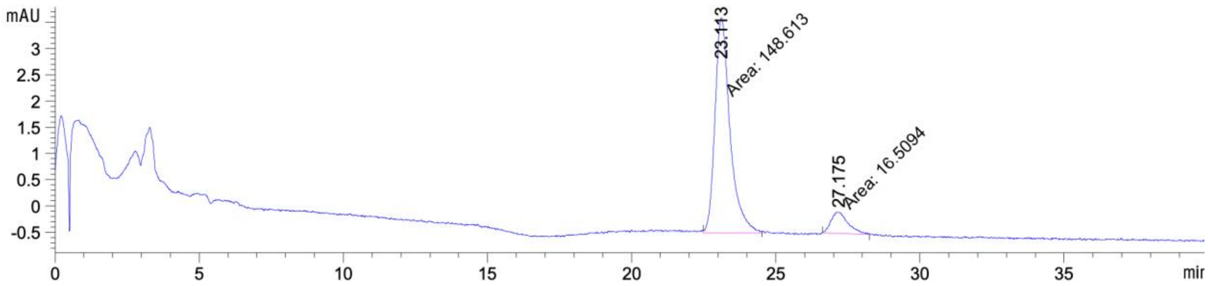

Peak #	RetTime [min]	Type	Width [min]	Area [mAU*s]	Height [mAU]	Area %
1	23.113	MM	0.6045	148.61314	4.09722	90.0017
2	27.175	MM	0.6884	16.50941	3.99696e-1	9.9983

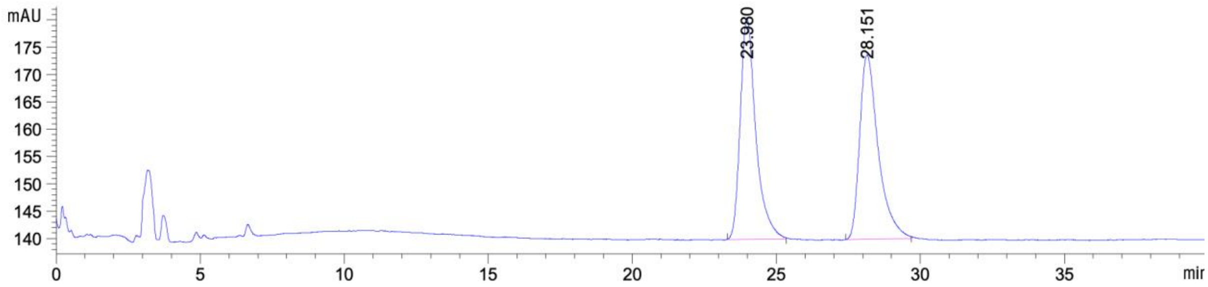

Peak #	RetTime [min]	Type	Width [min]	Area [mAU*s]	Height [mAU]	Area %
1	23.980	BB	0.5718	1532.26465	40.64712	50.1813
2	28.151	BB	0.6671	1521.19214	33.79866	49.8187

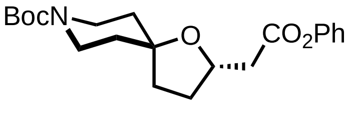

4x

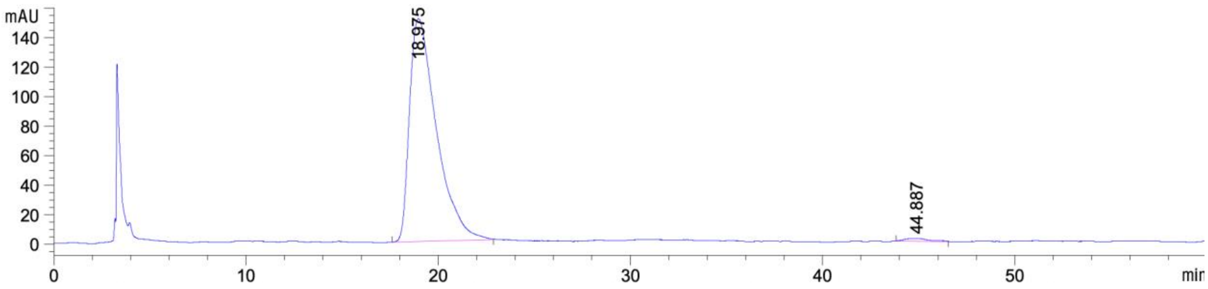

Peak #	RetTime [min]	Type	Width [min]	Area [mAU*s]	Height [mAU]	Area %
1	18.975	BB	1.4070	1.47271e4	151.10843	98.7490
2	44.887	BB	1.0537	186.56622	2.09356	1.2510

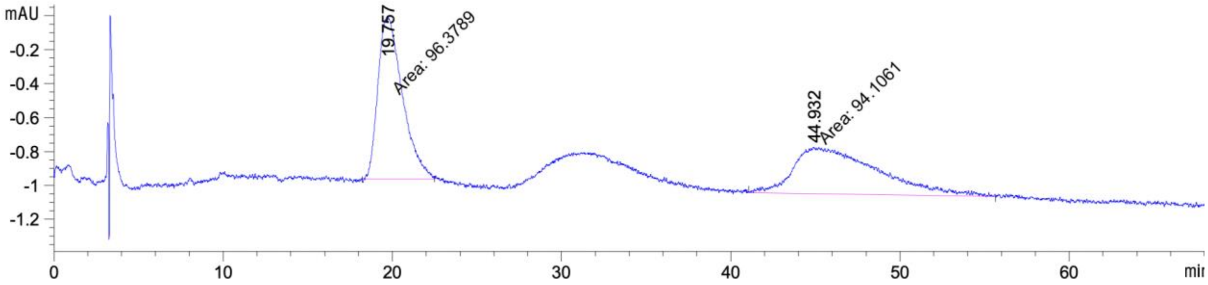

Peak #	RetTime [min]	Type	Width [min]	Area [mAU*s]	Height [mAU]	Area %
1	19.757	MM	1.6895	96.37894	9.50752e-1	50.5966
2	44.932	MM	5.6930	94.10606	2.75504e-1	49.4034

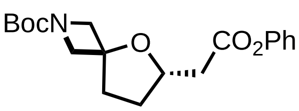

4y

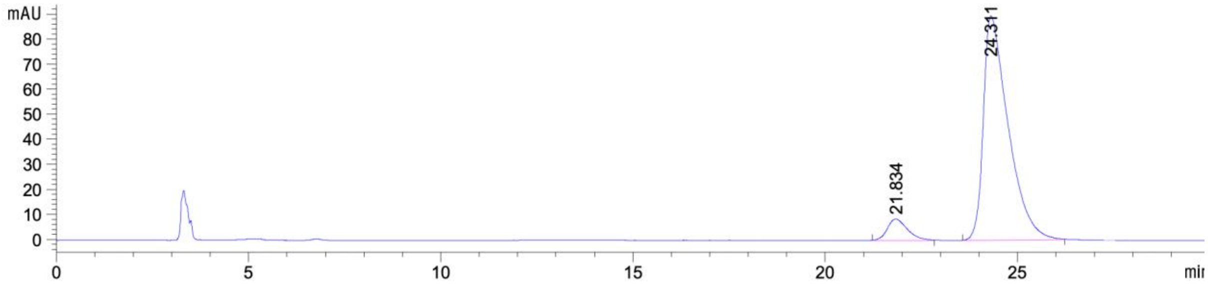

Peak #	RetTime [min]	Type	Width [min]	Area [mAU*s]	Height [mAU]	Area %
1	21.834	BB	0.5621	315.92633	8.60938	7.2680
2	24.311	BB	0.6662	4030.89185	89.70876	92.7320

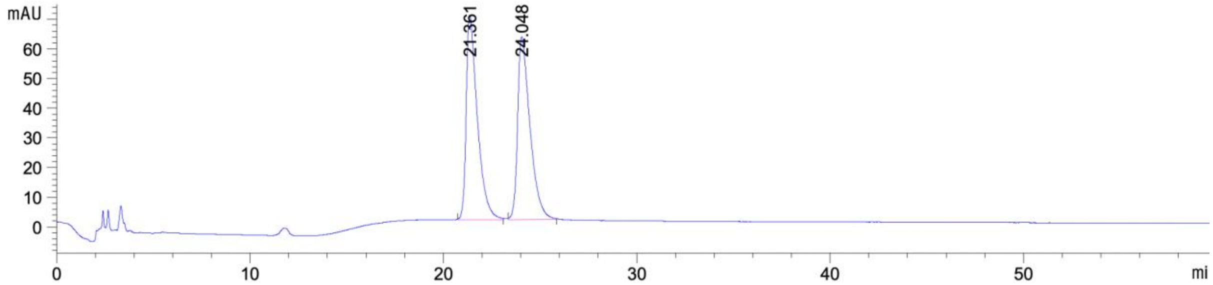

Peak #	RetTime [min]	Type	Width [min]	Area [mAU*s]	Height [mAU]	Area %
1	21.361	BB	0.5875	2745.25757	68.79700	50.1348
2	24.048	BB	0.6570	2730.49121	61.61629	49.8652

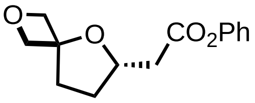

4z

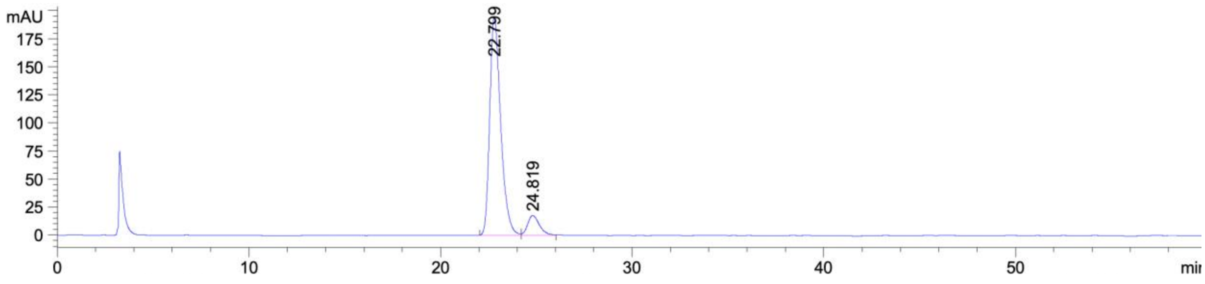

Peak #	RetTime [min]	Type	Width [min]	Area [mAU*s]	Height [mAU]	Area %
1	22.799	BB	0.6038	7729.92334	194.42815	91.0312
2	24.819	BB	0.6584	761.58209	17.68704	8.9688

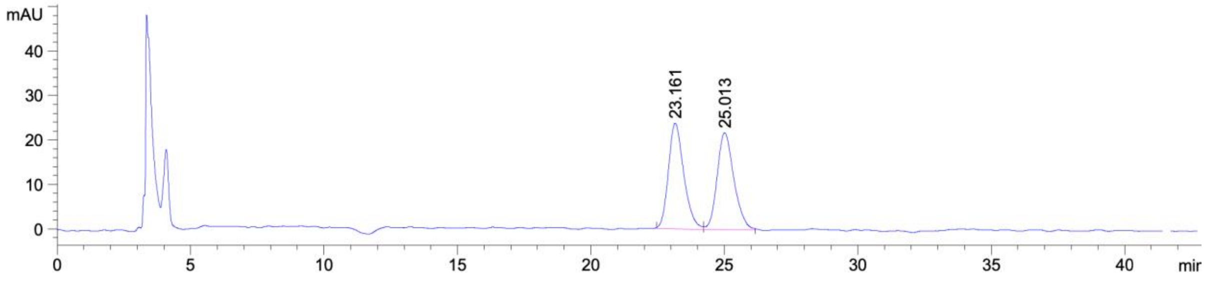

Peak #	RetTime [min]	Type	Width [min]	Area [mAU*s]	Height [mAU]	Area %
1	23.161	BB	0.6053	958.91498	23.83799	50.0146
2	25.013	BB	0.6683	958.35614	21.82643	49.9854

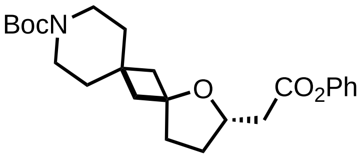

4aa

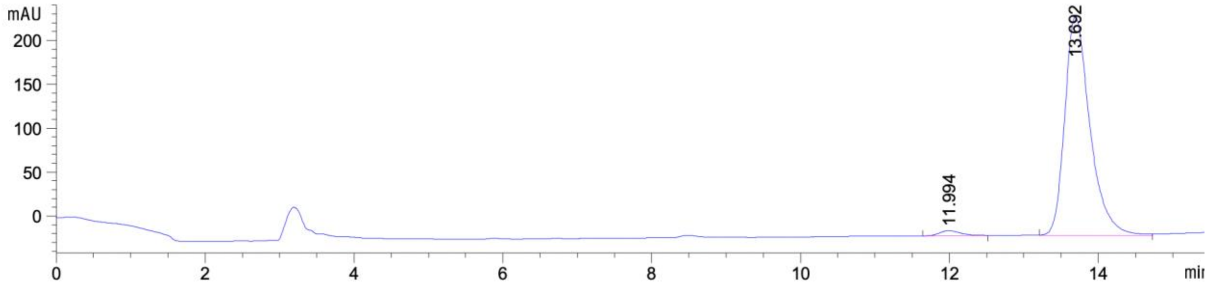

Peak #	RetTime [min]	Type	Width [min]	Area [mAU*s]	Height [mAU]	Area %
1	11.994	BB	0.3026	117.93710	5.86502	1.9725
2	13.692	BB	0.3581	5861.24268	249.50497	98.0275

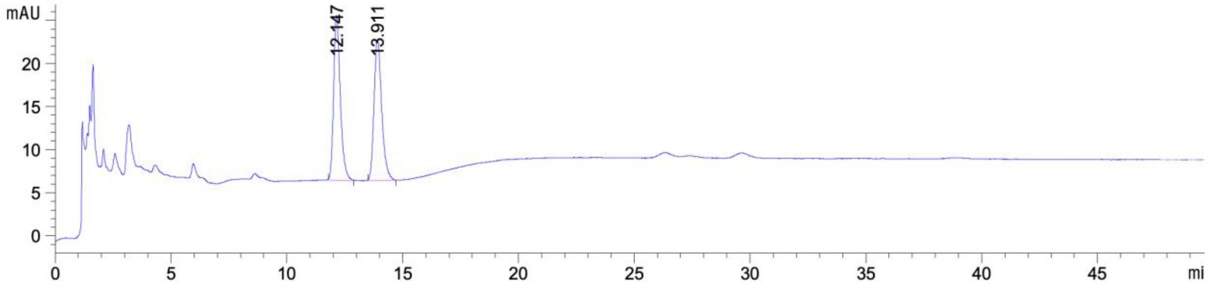

Peak #	RetTime [min]	Type	Width [min]	Area [mAU*s]	Height [mAU]	Area %
1	12.147	BB	0.3053	382.62088	19.13159	50.5501
2	13.911	BB	0.3511	374.29385	16.35173	49.4499

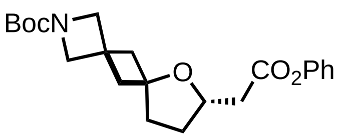

4ab

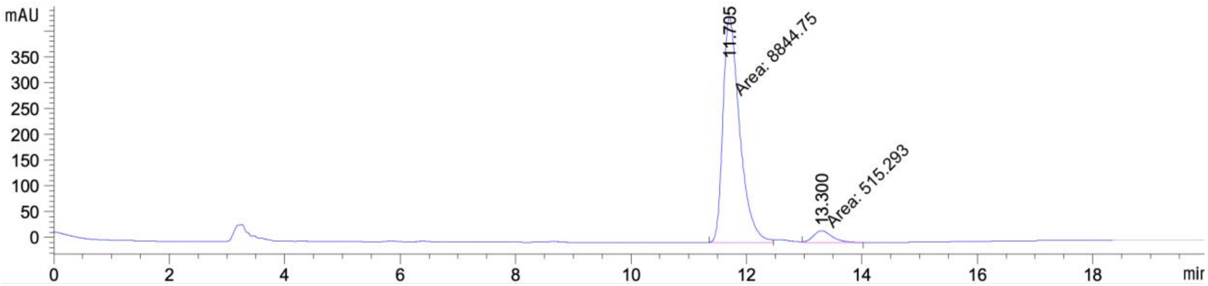

Peak #	RetTime [min]	Type	Width [min]	Area [mAU*s]	Height [mAU]	Area %
1	11.705	MM	0.3377	8844.74609	436.54654	94.4948
2	13.300	MM	0.3768	515.29346	22.79379	5.5052

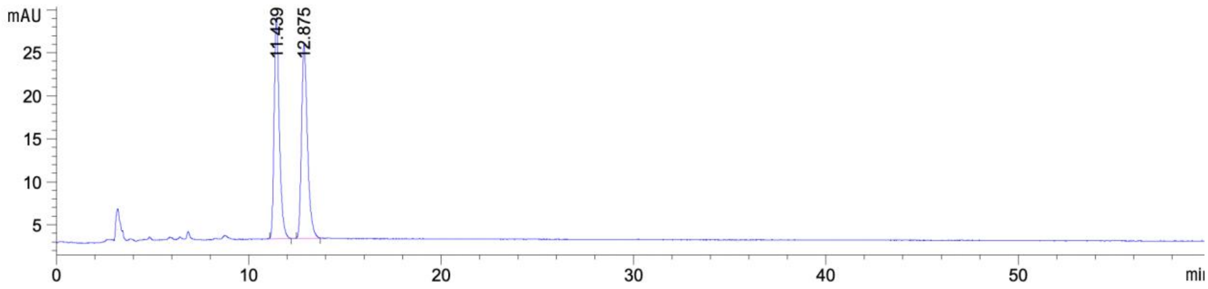

Peak #	RetTime [min]	Type	Width [min]	Area [mAU*s]	Height [mAU]	Area %
1	11.439	BB	0.2862	485.24509	25.70958	49.9949
2	12.875	BB	0.3271	485.34479	22.55262	50.0051

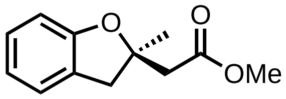

4af

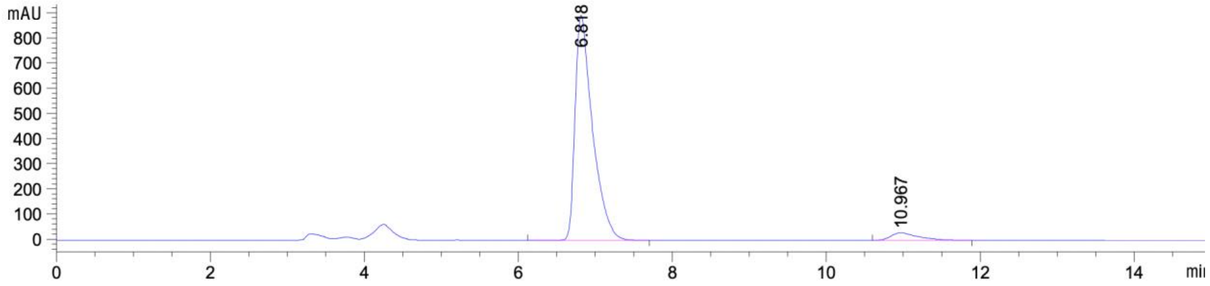

Peak #	RetTime [min]	Type	Width [min]	Area [mAU*s]	Height [mAU]	Area %
1	6.818	BB	0.2267	1.40038e4	894.33063	95.0467
2	10.967	BB	0.3528	729.79688	29.89680	4.9533

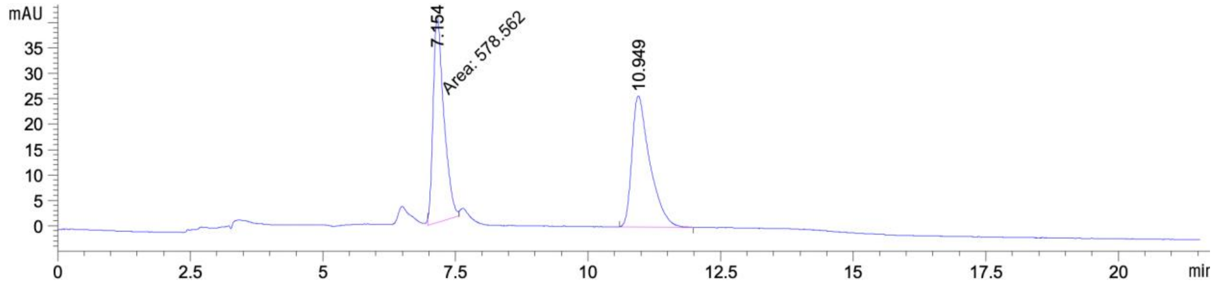

Peak #	RetTime [min]	Type	Width [min]	Area [mAU*s]	Height [mAU]	Area %
1	7.154	MM	0.2360	578.56219	40.86252	48.8740
2	10.949	BB	0.3417	605.22095	25.79529	51.1260

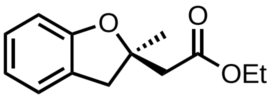

4ag

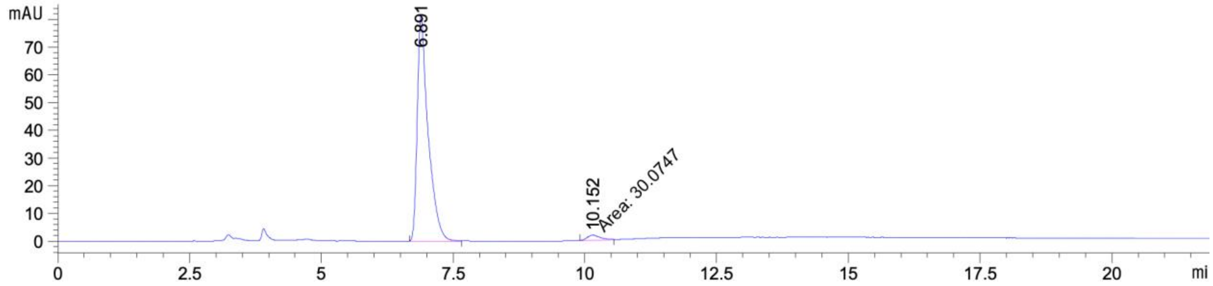

Peak #	RetTime [min]	Type	Width [min]	Area [mAU*s]	Height [mAU]	Area %
1	6.891	BB	0.2092	1183.45862	81.61973	97.5217
2	10.152	MM	0.2757	30.07473	1.81833	2.4783

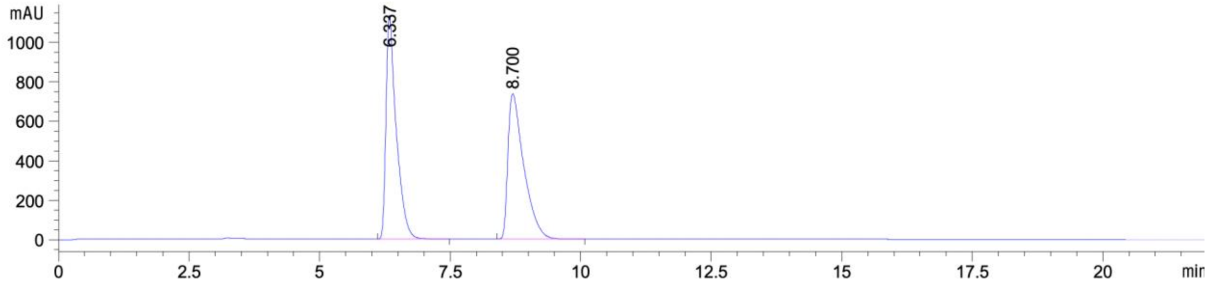

Peak #	RetTime [min]	Type	Width [min]	Area [mAU*s]	Height [mAU]	Area %
1	6.337	BB	0.1958	1.55329e4	1134.37524	49.9536
2	8.700	BB	0.3103	1.55618e4	737.05780	50.0464

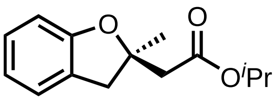

4ah

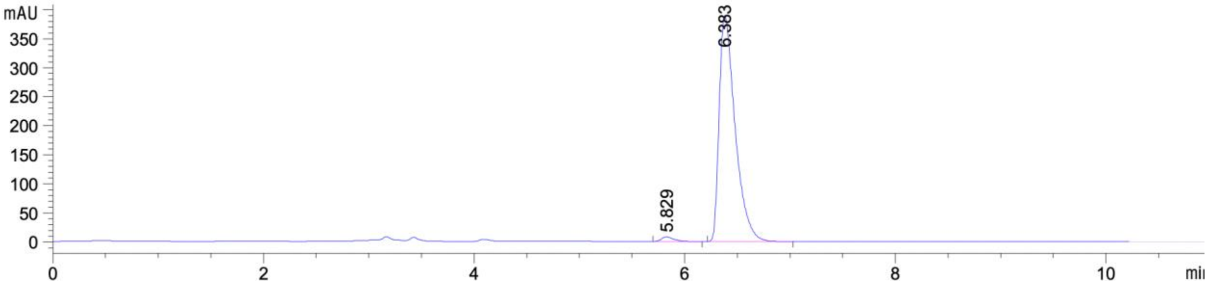

Peak #	RetTime [min]	Type	Width [min]	Area [mAU*s]	Height [mAU]	Area %
1	5.829	BB	0.1266	69.35152	8.20855	1.6657
2	6.383	BB	0.1606	4094.22241	389.63034	98.3343

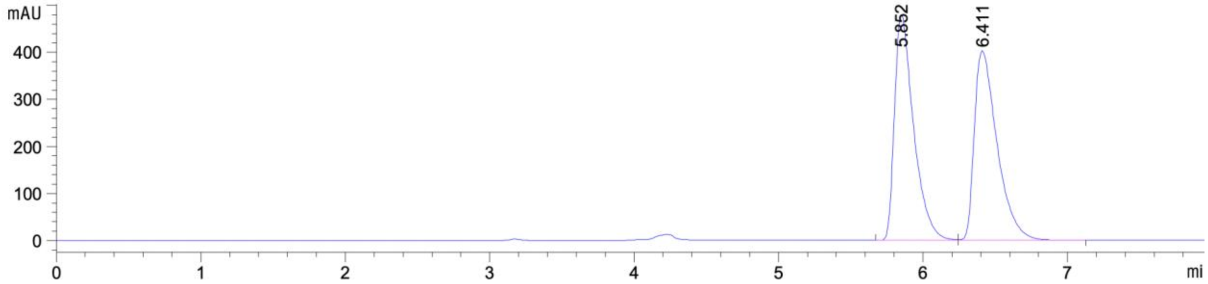

Peak #	RetTime [min]	Type	Width [min]	Area [mAU*s]	Height [mAU]	Area %
1	5.852	VV	0.1373	4401.22510	478.44495	49.8929
2	6.411	VB	0.1681	4420.12500	402.62119	50.1071

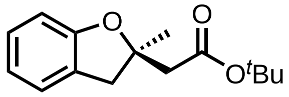

4ai

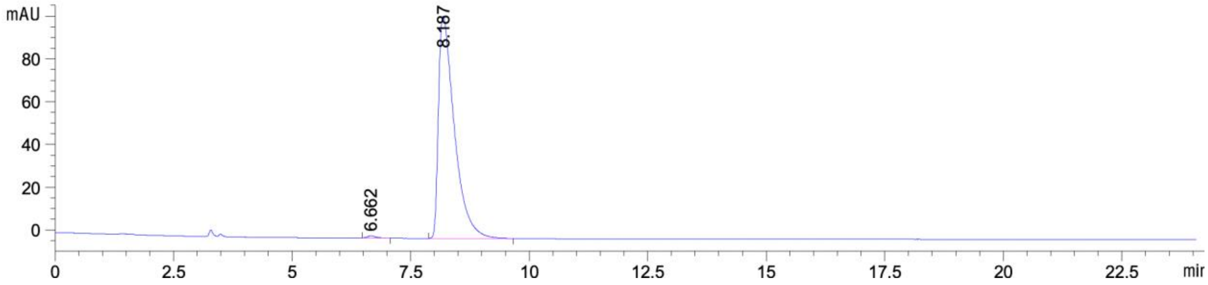

Peak #	RetTime [min]	Type	Width [min]	Area [mAU*s]	Height [mAU]	Area %
1	6.662	BB	0.1978	13.66202	1.01003	0.5632
2	8.187	BB	0.3469	2412.31323	103.90549	99.4368

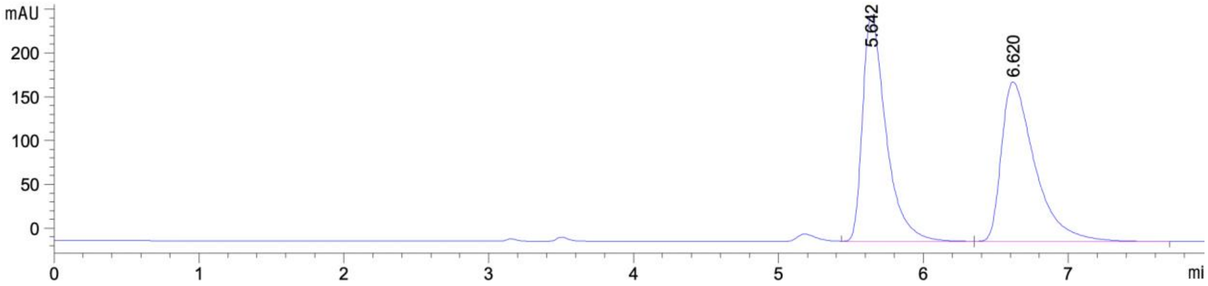

Peak #	RetTime [min]	Type	Width [min]	Area [mAU*s]	Height [mAU]	Area %
1	5.642	VV	0.1715	2911.48901	258.28906	49.9252
2	6.620	VB	0.2412	2920.21313	182.03874	50.0748

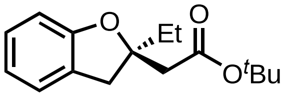

4aj

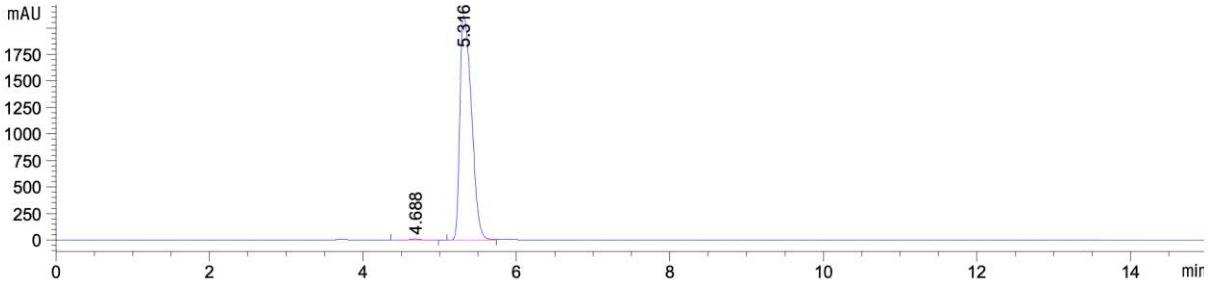

Peak #	RetTime [min]	Type	Width [min]	Area [mAU*s]	Height [mAU]	Area %
1	4.688	BB	0.1410	84.39632	8.55905	0.3807
2	5.316	BV	0.1676	2.20851e4	2119.12671	99.6193

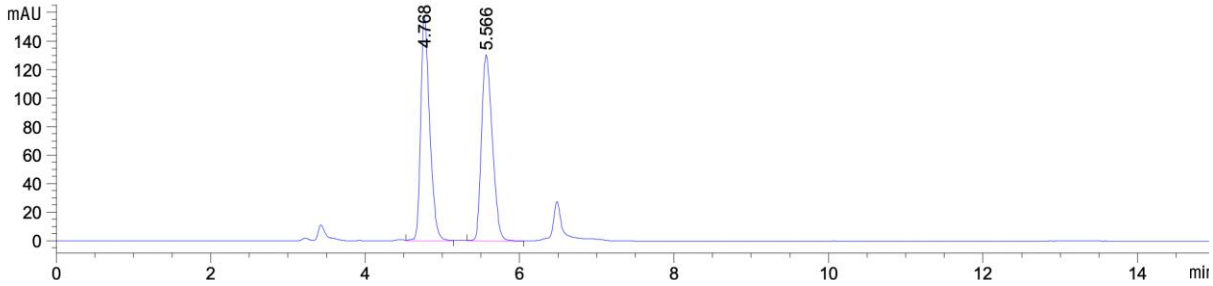

Peak #	RetTime [min]	Type	Width [min]	Area [mAU*s]	Height [mAU]	Area %
1	4.768	VV	0.1188	1255.65527	157.82893	49.8908
2	5.566	VB	0.1510	1261.15137	130.25391	50.1092

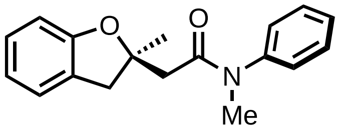

4ak

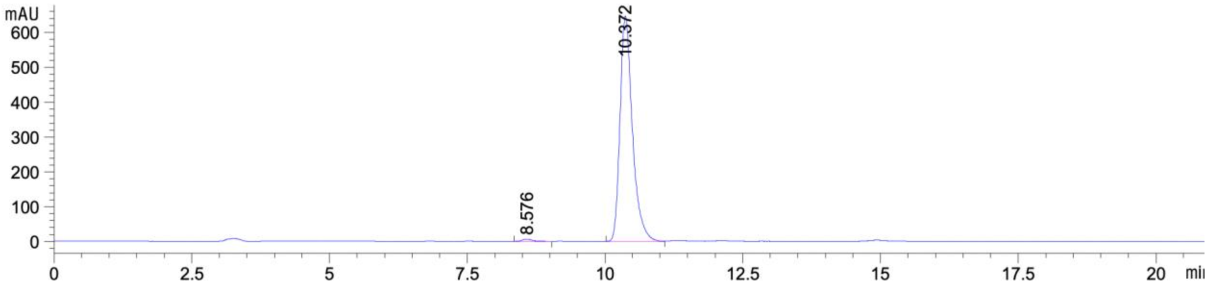

Peak #	RetTime [min]	Type	Width [min]	Area [mAU*s]	Height [mAU]	Area %
1	8.576	BB	0.1982	80.18137	6.14592	0.7881
2	10.372	BV	0.2380	1.00939e4	646.90863	99.2119

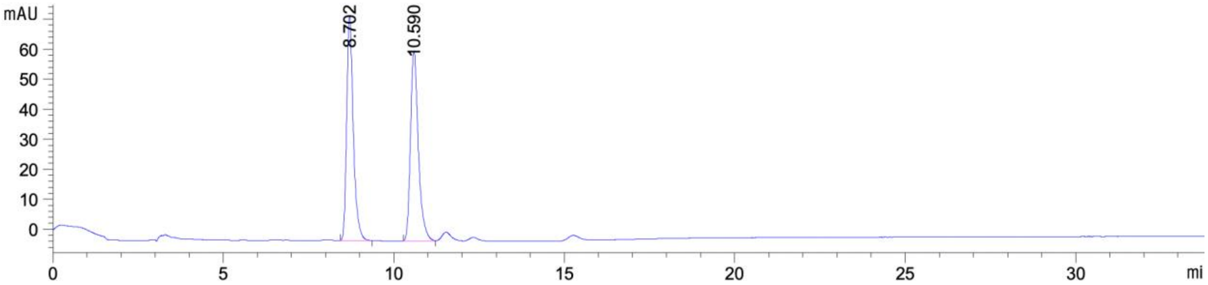

Peak #	RetTime [min]	Type	Width [min]	Area [mAU*s]	Height [mAU]	Area %
1	8.702	BB	0.2060	1013.67969	74.85977	50.0975
2	10.590	BV	0.2422	1009.73206	63.27227	49.9025

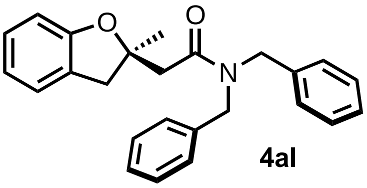

4al

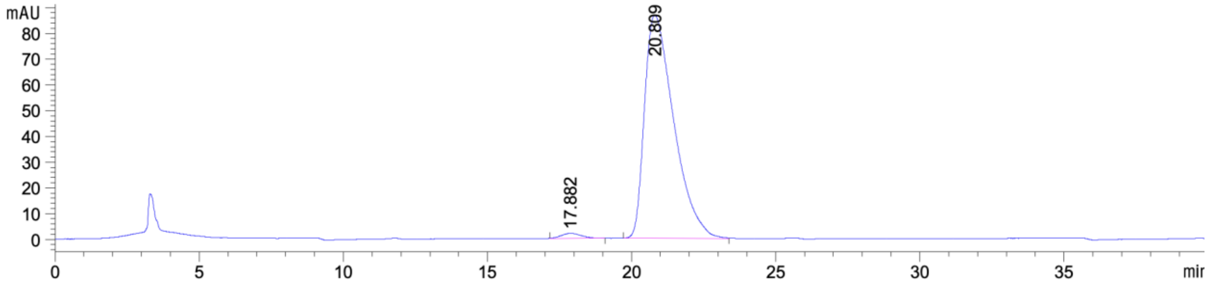

Peak #	RetTime [min]	Type	Width [min]	Area [mAU*s]	Height [mAU]	Area %
1	17.882	BB	0.6678	94.50056	1.94850	1.5171
2	20.809	BB	1.0758	6134.70654	86.66422	98.4829

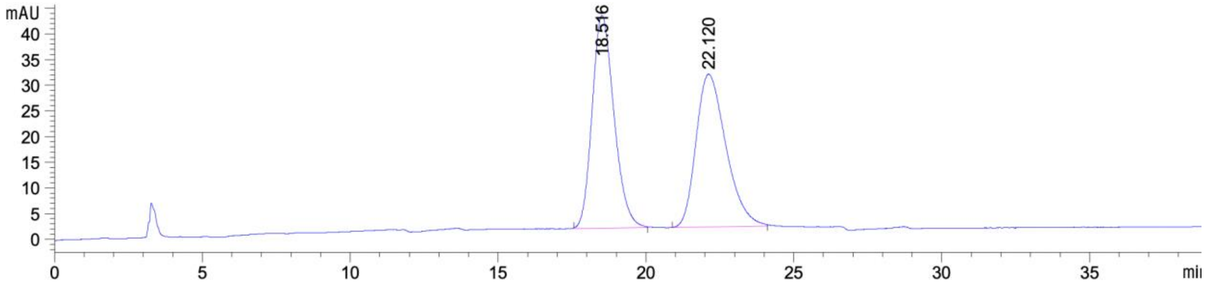

Peak #	RetTime [min]	Type	Width [min]	Area [mAU*s]	Height [mAU]	Area %
1	18.516	BB	0.7933	2136.45703	41.45103	50.4968
2	22.120	BB	1.0716	2094.42188	29.74032	49.5032

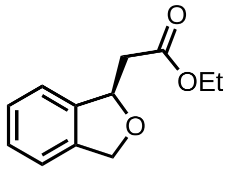

4am

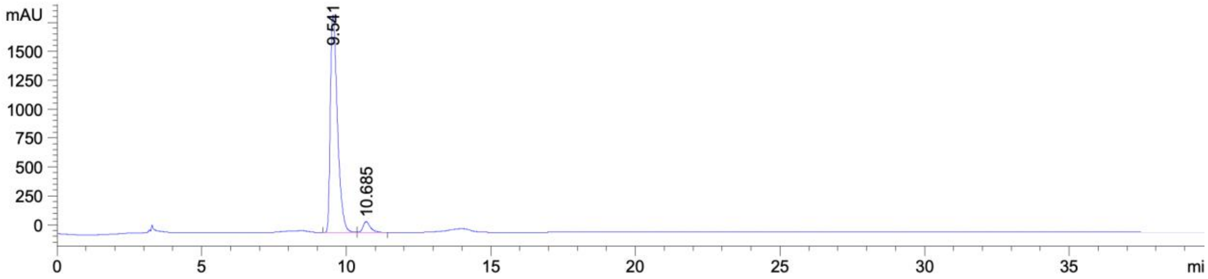

Peak #	RetTime [min]	Type	Width [min]	Area [mAU*s]	Height [mAU]	Area %
1	9.541	VV	0.2600	3.22551e4	1881.56311	95.0843
2	10.685	VB	0.2652	1667.54895	94.79186	4.9157

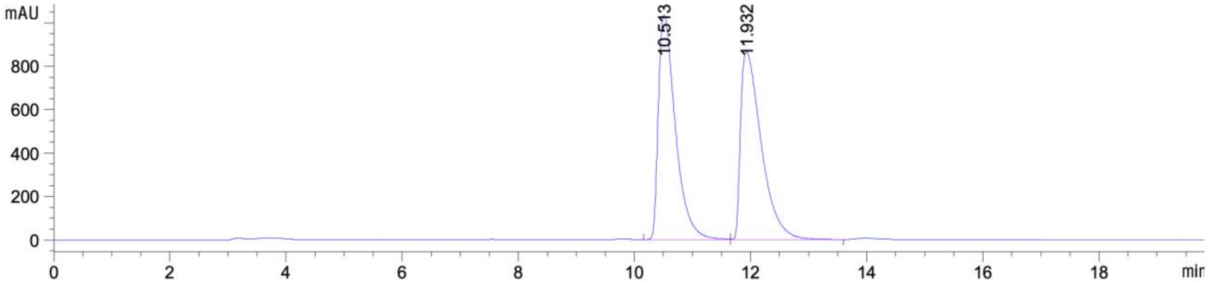

Peak #	RetTime [min]	Type	Width [min]	Area [mAU*s]	Height [mAU]	Area %
1	10.513	VV	0.3106	2.11711e4	1035.29224	49.6155
2	11.932	VV	0.3833	2.14993e4	866.86066	50.3845

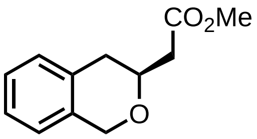

4an

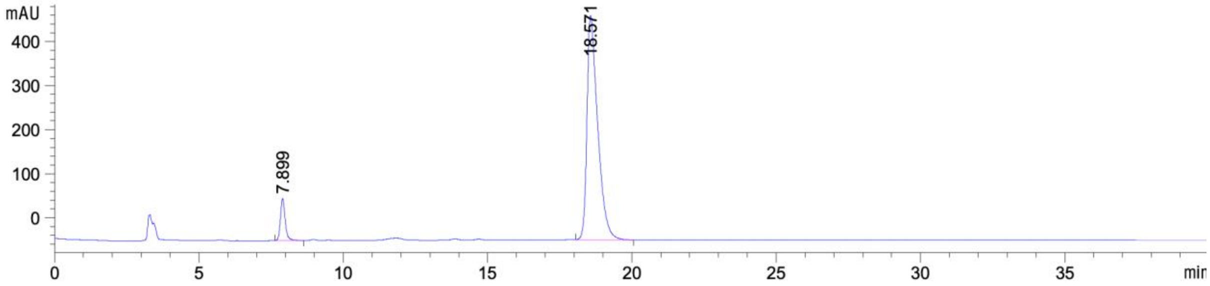

Peak #	RetTime [min]	Type	Width [min]	Area [mAU*s]	Height [mAU]	Area %
1	7.899	BB	0.1743	1105.24243	95.99947	7.7354
2	18.571	VB	0.3817	1.31829e4	509.83185	92.2646

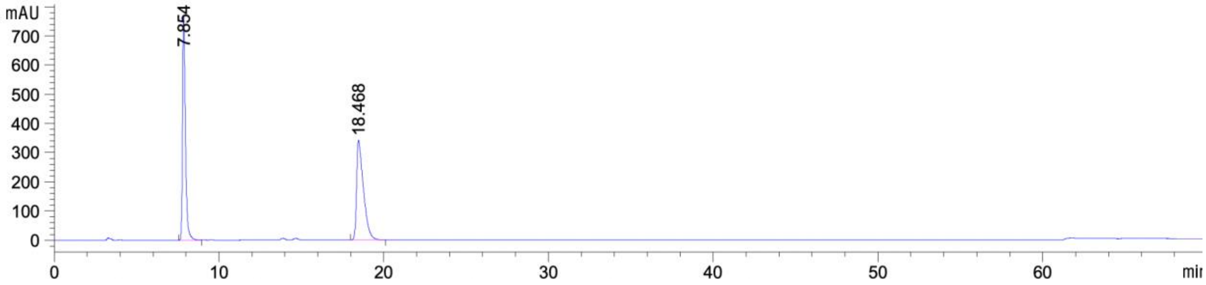

Peak #	RetTime [min]	Type	Width [min]	Area [mAU*s]	Height [mAU]	Area %
1	7.854	BB	0.1850	9448.12500	770.64429	49.0022
2	18.468	BB	0.4196	9832.89355	341.80624	50.9978

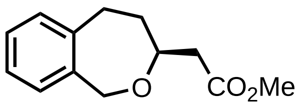

4ao

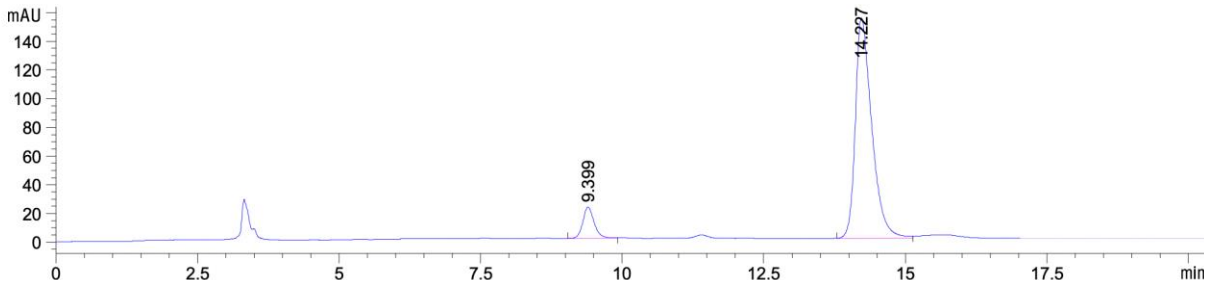

Peak #	RetTime [min]	Type	Width [min]	Area [mAU*s]	Height [mAU]	Area %
1	9.399	BB	0.2135	305.77008	21.81654	8.9529
2	14.227	BB	0.3020	3109.55737	153.69724	91.0471

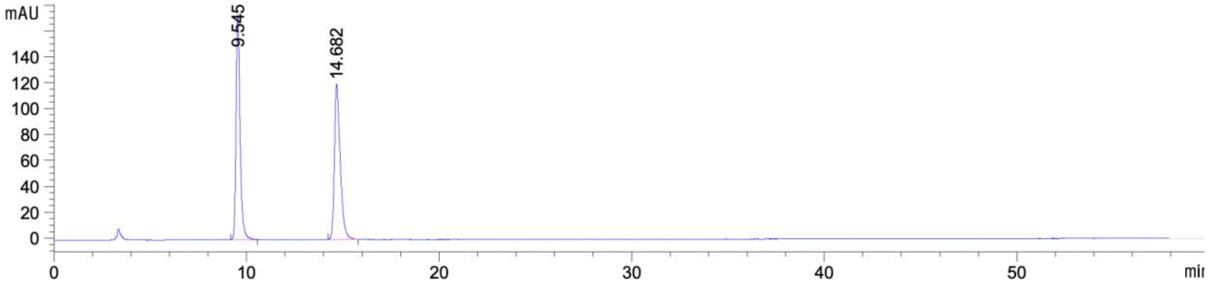

Peak #	RetTime [min]	Type	Width [min]	Area [mAU*s]	Height [mAU]	Area %
1	9.545	BB	0.2173	2517.09814	173.44472	50.0129
2	14.682	BB	0.3147	2515.80176	119.95415	49.9871

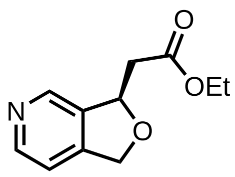

4ap

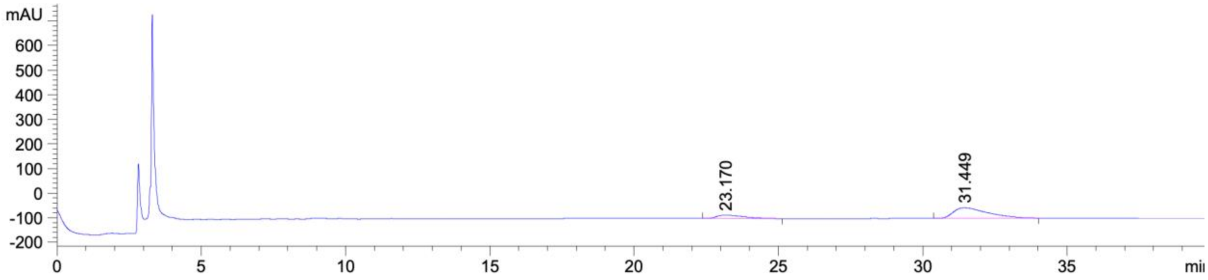

Peak #	RetTime [min]	Type	Width [min]	Area [mAU*s]	Height [mAU]	Area %
1	23.170	BB	0.9445	903.92322	13.86671	19.8692
2	31.449	BB	1.2484	3645.44727	43.37950	80.1308

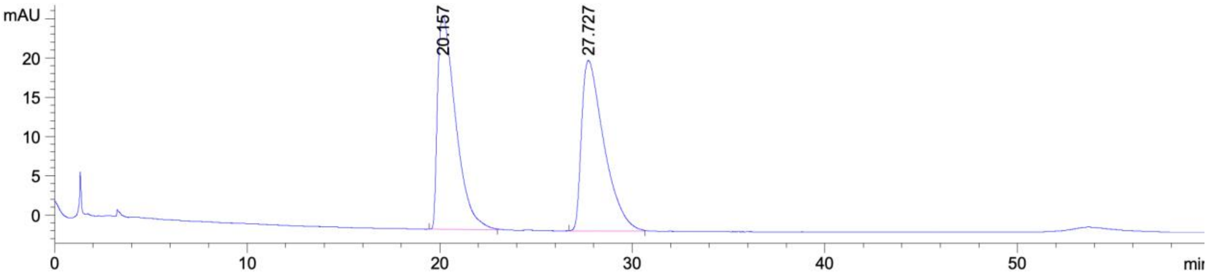

Peak #	RetTime [min]	Type	Width [min]	Area [mAU*s]	Height [mAU]	Area %
1	20.157	BB	1.0049	1783.37610	27.20021	50.3587
2	27.727	BB	1.1890	1757.97278	21.70398	49.6413

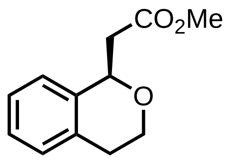

4aq

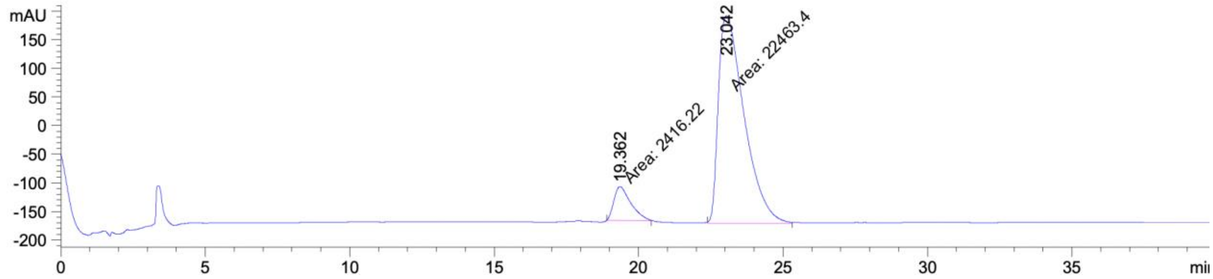

Peak #	RetTime [min]	Type	Width [min]	Area [mAU*s]	Height [mAU]	Area %
1	19.362	MM	0.6822	2416.21558	59.03285	9.7116
2	23.042	MM	1.0325	2.24634e4	362.60742	90.2884

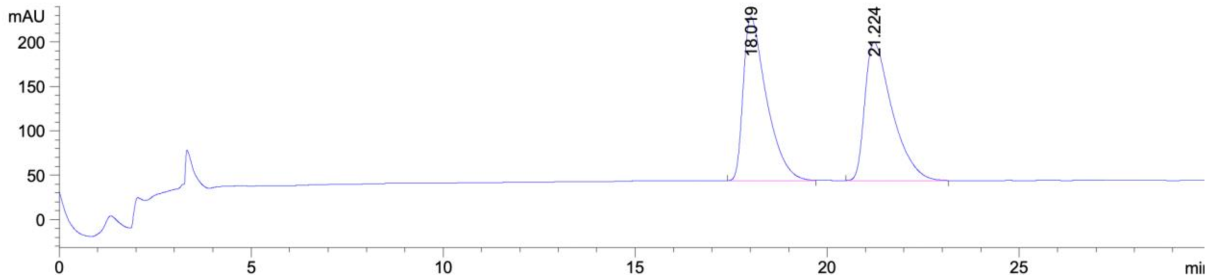

Peak #	RetTime [min]	Type	Width [min]	Area [mAU*s]	Height [mAU]	Area %
1	18.019	BB	0.6177	7730.66992	184.16956	50.0957
2	21.224	BB	0.7364	7701.13086	155.88680	49.9043

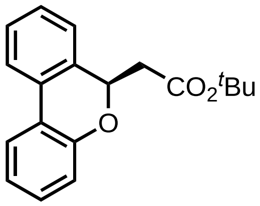

4ar

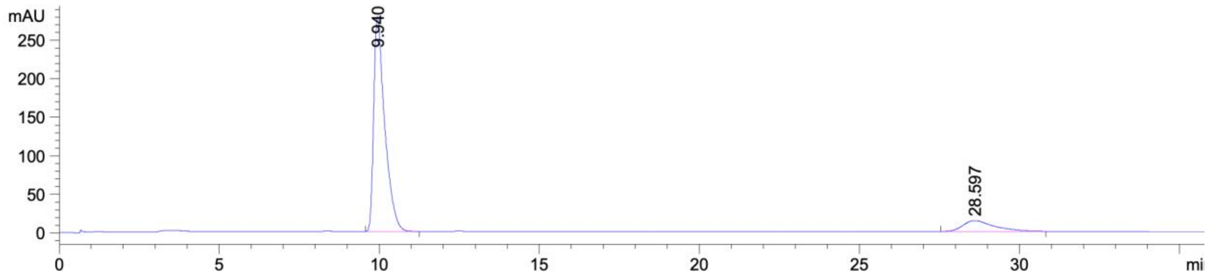

Peak #	RetTime [min]	Type	Width [min]	Area [mAU*s]	Height [mAU]	Area %
1	9.940	BB	0.3483	6709.39600	279.27539	87.5926
2	28.597	BB	0.9837	950.37567	14.07632	12.4074

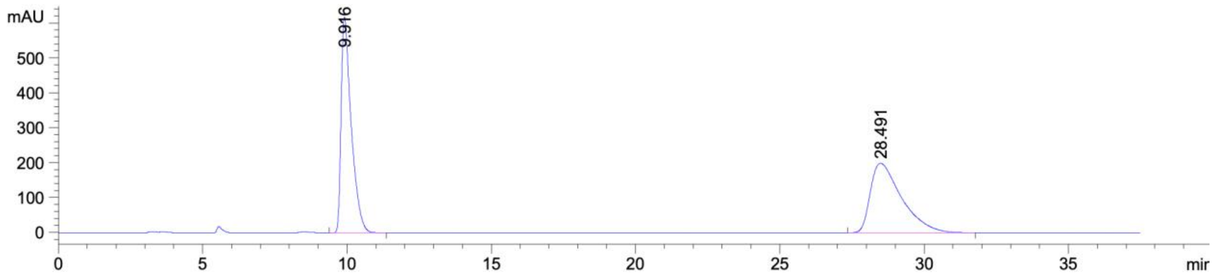

Peak #	RetTime [min]	Type	Width [min]	Area [mAU*s]	Height [mAU]	Area %
1	9.916	VB	0.3557	1.50543e4	619.16113	50.0073
2	28.491	BB	1.1332	1.50499e4	199.25960	49.9927

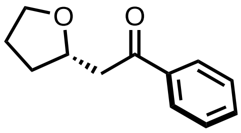

5a

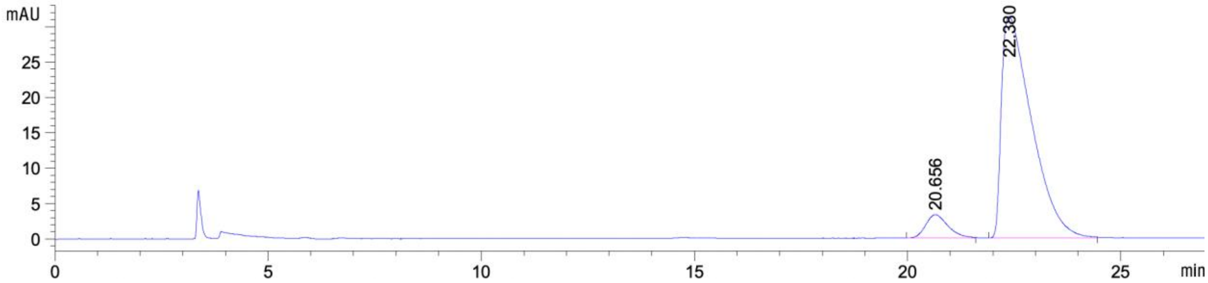

Peak #	RetTime [min]	Type	Width [min]	Area [mAU*s]	Height [mAU]	Area %
1	20.656	BB	0.5355	118.97023	3.27766	7.1841
2	22.380	BB	0.7204	1537.04895	31.32752	92.8159

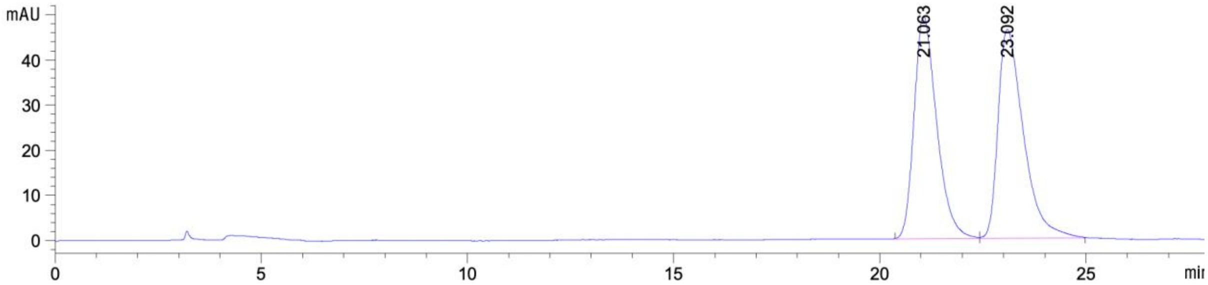

Peak #	RetTime [min]	Type	Width [min]	Area [mAU*s]	Height [mAU]	Area %
1	21.063	BB	0.5841	1884.18176	49.27311	48.6670
2	23.092	BB	0.6574	1987.40039	46.24489	51.3330

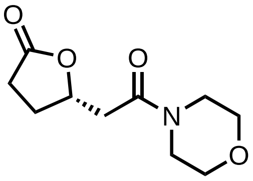

5b

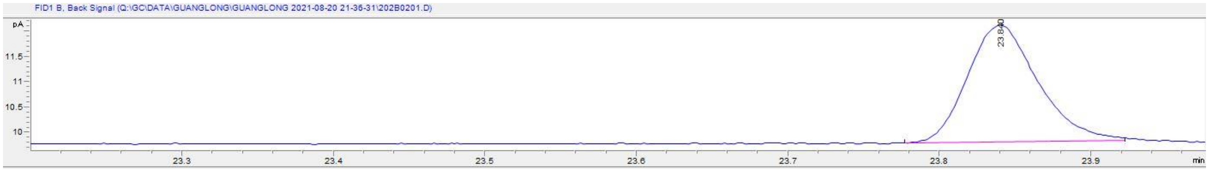

#	Time	Area	Height	Width	Area%	Symmetry
1	23.84	7.5	2.3	0.0499	100.000	0.814

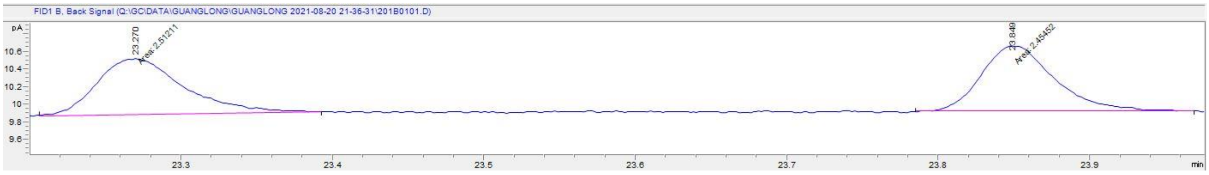

#	Time	Area	Height	Width	Area%	Symmetry
1	23.27	2.5	6.3E-1	0.0663	50.580	0.806
2	23.849	2.5	7.4E-1	0.0555	49.420	0.74

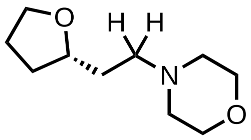

5c

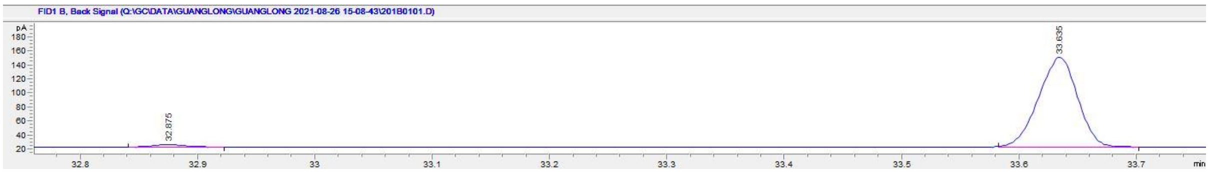

#	Time	Area	Height	Width	Area%	Symmetry
1	32.875	5.7	3.2	0.0279	1.879	0.953
2	33.635	295.1	127.7	0.036	98.121	1.145

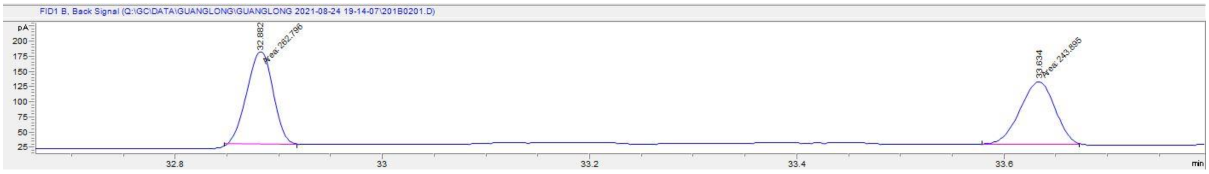

#	Time	Area	Height	Width	Area%	Symmetry
1	32.882	262.8	152.7	0.0287	51.865	1.027
2	33.634	243.9	103.3	0.0393	48.135	1.143

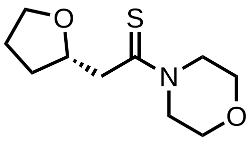

5d

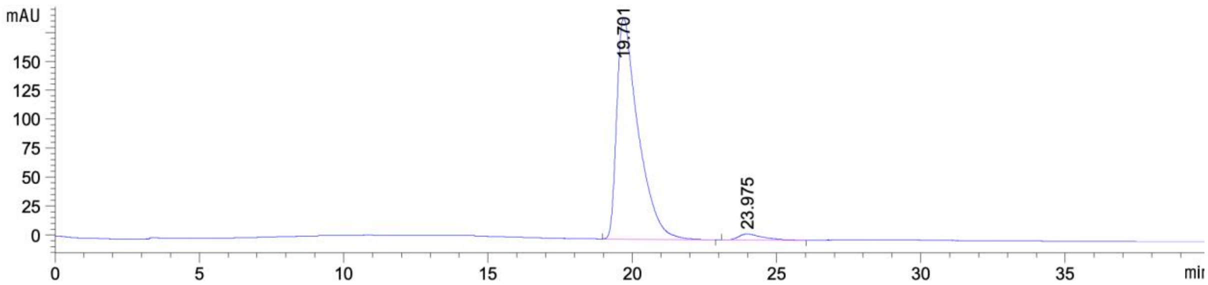

Peak #	RetTime [min]	Type	Width [min]	Area [mAU*s]	Height [mAU]	Area %
1	19.701	BB	0.7467	9893.28223	191.52293	97.1237
2	23.975	BB	0.8123	292.98795	5.24046	2.8763

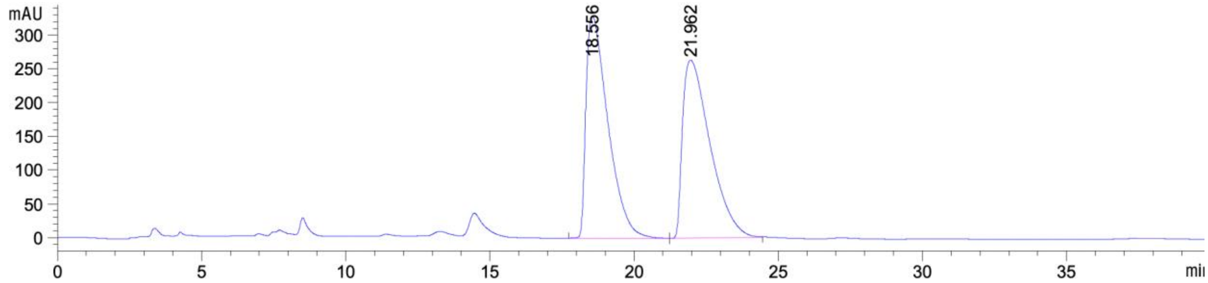

Peak #	RetTime [min]	Type	Width [min]	Area [mAU*s]	Height [mAU]	Area %
1	18.556	BB	0.8106	1.74855e4	329.75934	49.9071
2	21.962	BB	1.0116	1.75507e4	263.98917	50.0929

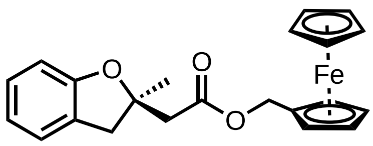

5e

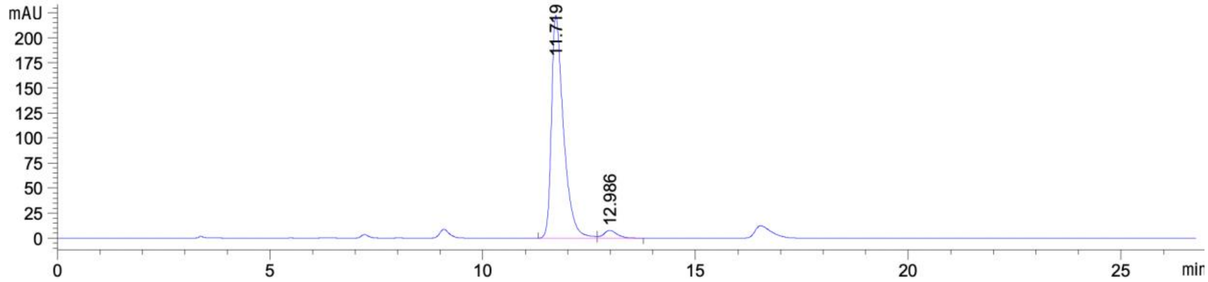

Peak #	RetTime [min]	Type	Width [min]	Area [mAU*s]	Height [mAU]	Area %
1	11.719	BV	0.2903	4393.60010	222.61356	96.1914
2	12.986	VB	0.3235	173.96124	7.94482	3.8086

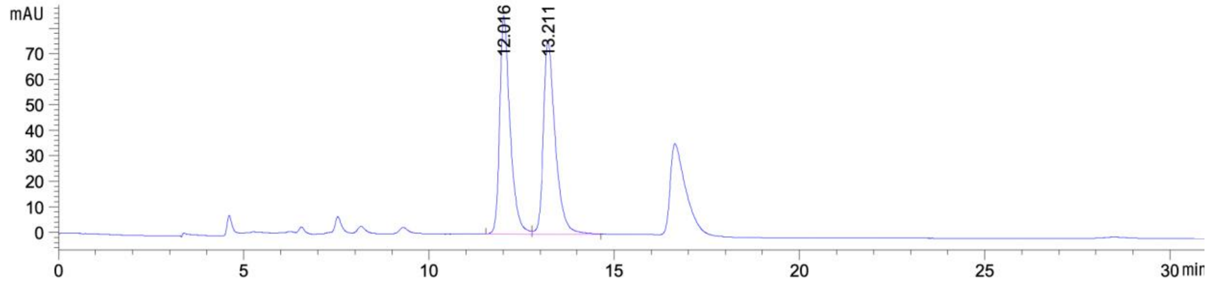

Peak #	RetTime [min]	Type	Width [min]	Area [mAU*s]	Height [mAU]	Area %
1	12.016	VV	0.2921	1673.84558	85.60327	49.6309
2	13.211	VB	0.3335	1698.73901	75.79695	50.3691

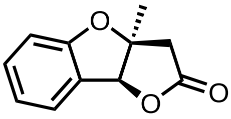

5f

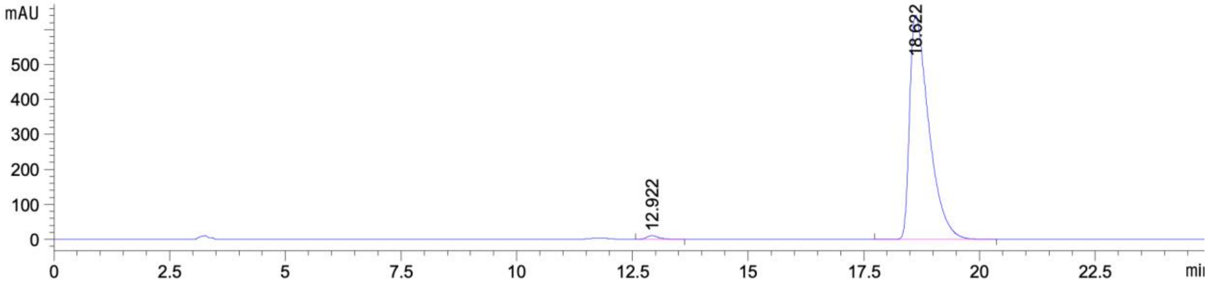

Peak #	RetTime [min]	Type	Width [min]	Area [mAU*s]	Height [mAU]	Area %
1	12.922	VB	0.2653	185.09251	10.51958	0.9932
2	18.622	BB	0.4358	1.84505e4	641.10901	99.0068

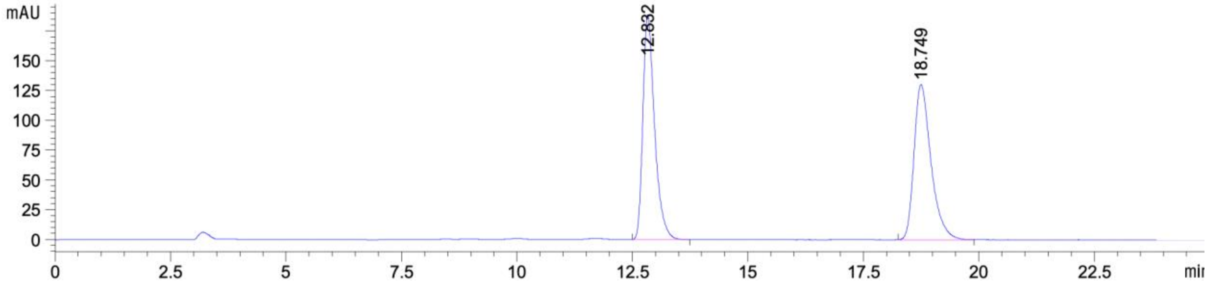

Peak #	RetTime [min]	Type	Width [min]	Area [mAU*s]	Height [mAU]	Area %
1	12.832	BB	0.2677	3327.64111	188.70679	49.9927
2	18.749	BB	0.3859	3328.61475	130.35880	50.0073

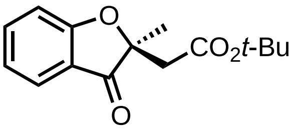

5g

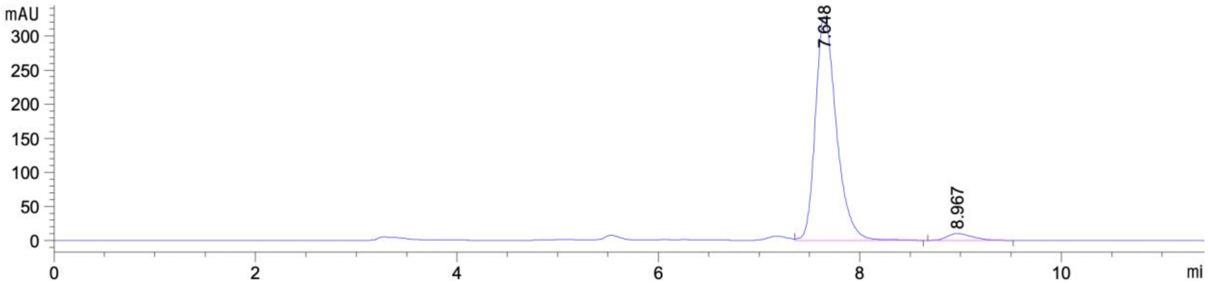

Peak #	RetTime [min]	Type	Width [min]	Area [mAU*s]	Height [mAU]	Area %
1	7.648	VB	0.2205	4742.28955	328.39786	96.4648
2	8.967	BB	0.2672	173.79512	9.98076	3.5352

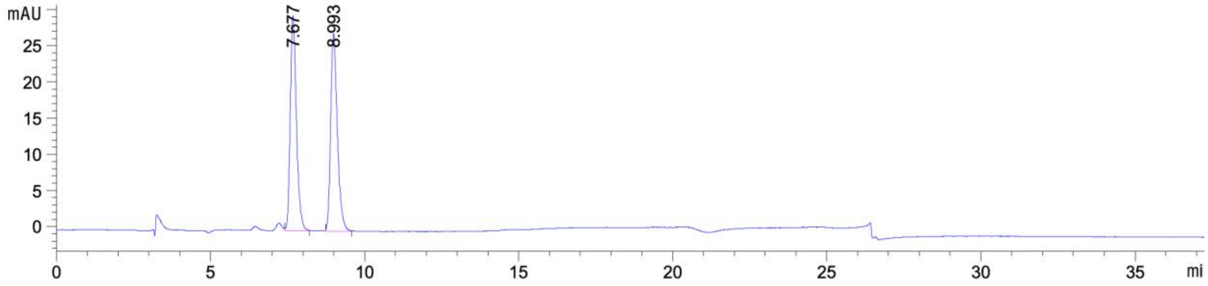

Peak #	RetTime [min]	Type	Width [min]	Area [mAU*s]	Height [mAU]	Area %
1	7.677	VB	0.2102	413.01266	29.70541	50.0976
2	8.993	BB	0.2283	411.40366	27.22878	49.9024

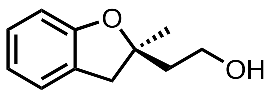

5h

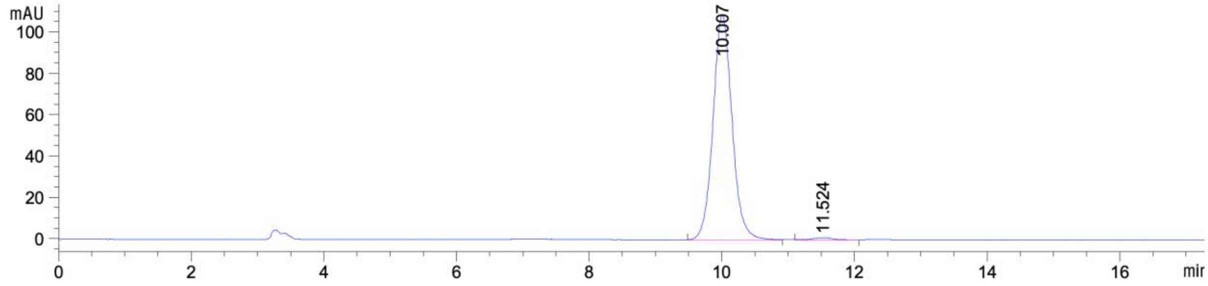

Peak #	RetTime [min]	Type	Width [min]	Area [mAU*s]	Height [mAU]	Area %
1	10.007	BB	0.3093	2169.39648	108.50298	99.0373
2	11.524	BB	0.3168	21.08857	9.73112e-1	0.9627

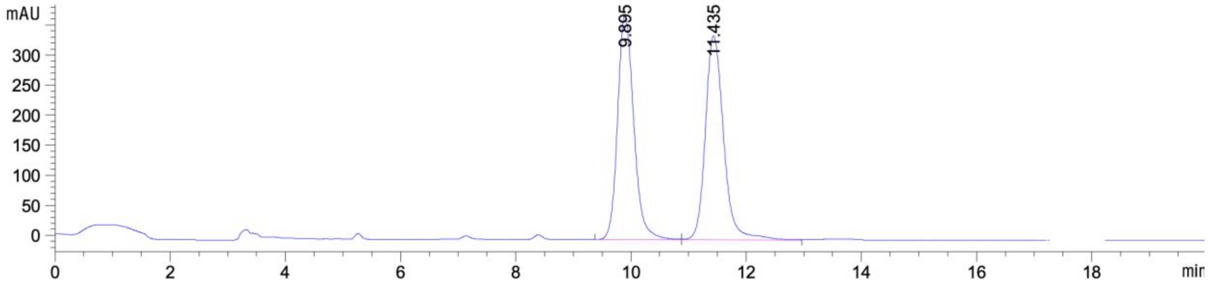

Peak #	RetTime [min]	Type	Width [min]	Area [mAU*s]	Height [mAU]	Area %
1	9.895	BV	0.3051	7331.03809	373.28925	49.4261
2	11.435	VB	0.3414	7501.29590	340.00787	50.5739

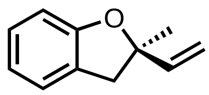

5i

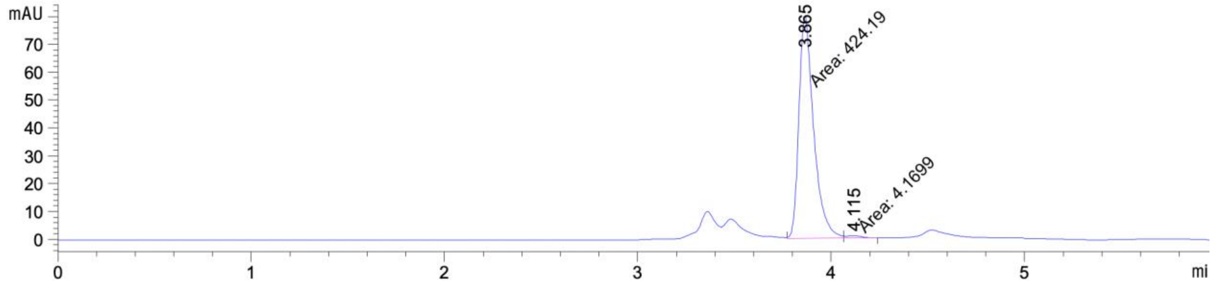

Peak #	RetTime [min]	Type	Width [min]	Area [mAU*s]	Height [mAU]	Area %
1	3.865	MF	0.0877	424.18954	80.64874	99.0265
2	4.115	FM	0.0859	4.16990	8.08948e-1	0.9735

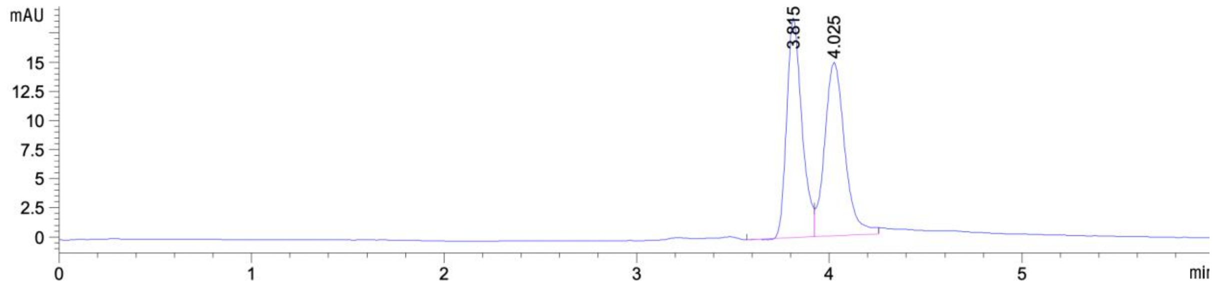

Peak #	RetTime [min]	Type	Width [min]	Area [mAU*s]	Height [mAU]	Area %
1	3.815	VV	0.0844	103.64482	18.76944	48.7422
2	4.025	VB	0.1136	108.99380	14.84916	51.2578

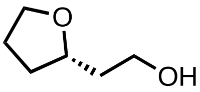

5j

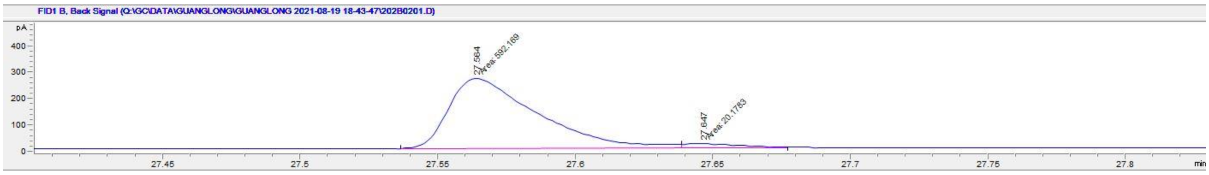

#	Time	Area	Height	Width	Area%	Symmetry
1	27.564	592.2	265.3	0.0372	96.705	0.424
2	27.647	20.2	14.9	0.0226	3.295	0

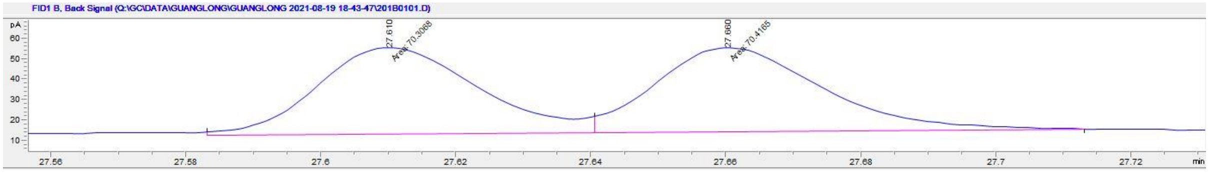

#	Time	Area	Height	Width	Area%	Symmetry
1	27.61	70.3	42.2	0.0278	49.961	0.772
2	27.66	70.4	41.3	0.0284	50.039	0.712

12. References

[1]. Farley, A. J. M.; Sandford, C.; Dixon, D. J. Bifunctional Iminophosphorane Catalysed Enantioselective Sulfa-Michael Addition to Unactivated α-Substituted Acrylate Esters, *J. Am. Chem. Soc.* **2015**, *137*, 15992-15995.

[2]. Nuñez, M. G.; Farley, A. J. M.; Dixon, D. J. Bifunctional Iminophosphorane Organocatalysts for Enantioselective Synthesis: Application to the Ketimine Nitro-Mannich Reaction, *J. Am. Chem. Soc.* **2013**, *135*, 16348-16351.

[3]. Rotstein, D. M.; Gabriel, S. D.; Manser, N.; Filonova, L.; Padilla, F.; Sankuratri, S.; Ji, C.; Rosier, A. de; Dioszegi, M.; Heilek, G.; Jekle, A.; Weller, P.; Berry, P. Synthesis, SAR and evaluation of [1,4']-bipiperidinyl-4-yl-imidazolidin-2-one derivatives as novel CCR5 antagonists, *Bioorg. Med. Chem. Lett.,* **2010**, *20*, 3219-3222.

[4]. Rozsar, D.; Formica, M.; Yamazaki, K.; Hamlin, T.; Dixon, D. J. Bifunctional Iminophosphorane Catalysed Enantioselective Sulfa-Michael Addition to Unactivated α,β-Unsaturated Amides, 10.26434/chemrxiv.14355422.

[5]. Matsuda, Y.; Tsuji, Y.; Fujihara, T. Cu-Catalysed Three-Component Coupling Reactions Using Nitriles, 1,3-Dienes and Silylboranes, *Chem. Commun.,* **2020**, *56*, 4648-4651.

[6]. Cowen, B. J.; Saunders, L. B.; Miller, S. J. Pyridylalanine (Pal)-Peptide Catalysed Enantioselective Allenoate Additions to *N*-Acyl Imines, *J. Am. Chem. Soc.,* **2009**, *131*, 17, 6105-6107.

[7]. Lee, D.; Kim, D.; Lee, S.; Kim, T.; Kim, J.; Kim, S.; Liu, K. H.; Lee, S.; Bae, J. S.; Song, K. S.; Cho, C. W.; Son, Y. K.; Baek, D. J.; Lee, T. Efficient Syntheses of 1,2,3-Triazoloamide Derivatives Using Solid- and Solution-Phase Synthetic Approaches, *Molecules.* **2015**, *20*, 11, 19984-20013.

[8]. Diehl, K.; Himbert, G.; Henn, L. Intramolekulare Diels-Alder-Reaktionen bei Allencarboxaniliden; Variation der Substituenten in p-Position des Anilinkerns, *Chem. Ber.* **1986**, *119*, 2430-2443.

[9]. Kobayashi, Y.; Taniguchi, Y.; Hayama, N.; Inokuma, T.; Takemoto, Y. A Powerful Hydrogen-Bond-Donating Organocatalyst for the Enantioselective Intramolecular Oxa-Michael Reaction of α,β-Unsaturated Amides and Esters. *Angew. Chem. Int. Ed.,* **2013**, *52*, 11114-11118.

[10]. Stang, E. M.; White, M. C. Molecular Complexity *via* C–H Activation: A Dehydrogenative Diels–Alder Reaction. *J. Am. Chem. Soc.* **2011**, *133*, 38, 14892–14895.

[11]. Prasad, K. R.; Pawar, A. B. Enantioselective Formal Synthesis of Palmerolide A. *Org. Lett.,* **2011**, *13*, 16, 4252–4255.

[12]. Lauberteaux, J.; Crévisy, C.; Baslé, O.; Figueiredo, R. M. de; Mauduit, M.; Campagne, J. M. Copper-Catalysed Asymmetric Conjugate Additions of Bis(pinacolato)diboron and Dimethylzinc to Acyl-

N-methylimidazole Michael Acceptors: A Highly Stereoselective Unified Strategy for 1,3,5,...n (OH, Me) Motif Synthesis. *Org. Lett.,* **2019**, *21*, 6, 1872–1876.

[13]. Venkateshwarlu, R.; Chinnababu, B.; Ramulu, U.; Reddy, K. P.; Reddy, M. D.; Sowjanya, P.; Rao, P. V.; Aravind, S. Synthesis and Biological Evaluation of (−)-Kunstleramide and Its Derivatives. *Med. Chem. Commun.,* **2017**, *8*, 394-404.

[14]. Hamaguchi, T.; Takahashi, Y.; Tsuji, H.; Kawatsura, M. Nickel-Catalysed Hydroarylation of *in Situ* Generated 1,3-Dienes with Arylboronic Acids Using a Secondary Homoallyl Carbonate as a Surrogate for the 1,3-Diene and Hydride Source. *Org. Lett.,* **2020**, *22*, 3, 1124–1129.

[15]. Barton, D. H. R.; Gokturk, A. K. Functionalization of Saturated Hydrocarbons. Part 3. The Oxidation of 3β,5α,6β-Triacetoxycholestane using the Gif System. *J. Chem. Soc., Perkin Trans.* **1985**, *1*, 2109-2117.

[16]. Yamamoto, Y.; Yamada, S.; Nishiyama, H. Copper-Catalysed Regio- and Stereoselective Conjugate Allylation of Electron-Deficient Alkynes with Allylboronates under Mild Conditions. *Chem. Eur. J.* **2012**, *18*, 3153–3156.

[17]. Escudero, J.; Bellosta, V.; Cossy, J. Rhodium-Catalysed Cyclization of O,ω-Unsaturated Alkoxyamines: Formation of Oxygen-Containing Heterocycles. *Angew. Chem. Int. Ed.,* **2018**, *57*, 574–578.

[18]. Yip, S. Y. Y.; Aïssa, C. Isomerization of Olefins Triggered by Rhodium-Catalysed C–H Bond Activation: Control of Endocyclic β-Hydrogen Elimination *Angew. Chem. Int. Ed.* **2015**, *54*, 6870–6873.

[19]. Frantz, D. E.; Singleton, D. A. Carbometalations of Simple Alkenes with Allyldibromoborane. *Org. Let.,* **1999**, *1*, 3, 485–486.

[20]. Clive, D. L. J.; M. P.; Pham, Subedi, R. Carbocyclization by Radical Closure onto O-Trityl Oximes: Dramatic Effect of Diphenyl Diselenide. *J. Am. Chem. Soc.* **2007**, *129*, 9, 2713–2717.

[21]. Wang, D.; Lichtenfeld, C. M.; Daniliuc, C. G.; Studer, A. Radical Aryl Migration from Boron to Carbon. *J. Am. Chem. Soc.* **2021**, *143*, 25, 9320–9326.

[22]. Jakubec, P.; Hawkins, A.; Felzmann, W.; Dixon, D. J. Total Synthesis of Manzamine A and Related Alkaloids. *J. Am. Chem. Soc.* **2012**, *134*, 42, 17482–17485.

[23]. Huy, P. H.; Koskinen, A. M. P. Efficient, Stereodivergent Access to 3-Piperidinols by Traceless $P(OEt)_3$ Cyclodehydration. *Org. Lett.,* **2013**, *15*, 20, 5178–5181.

[24]. Cooper, L. C.; Carlson, E. J.; Castro, J. L.; Chicchi, G. G.; Dinnell, K.; Salvo, J. D.; Elliott, J. M.; Hollingworth, G. J.; Kurtz, M. M.; Ridgill, M. P.; Rycroft, W.; Tsao, K. L.; Swain, C. J. 4,4-Disubstituted cyclohexylamine NK1 receptor antagonists II. *Bioorg. Med. Chem. Lett.,* **2002**, *12*, 13, 1759-1762.

[25]. Davis, R. W.; Allweil, A.; Tian, J.; Brash, A. R.; Sulikowski, G. A. Stereocontrolled Synthesis of Four Isomeric Linoleate Triols of Relevance to Skin Barrier Formation and Function. *Tetrahedron Lett.,* **2018**, *59*, 4571-4573.

[26]. Hurtak, J. A.; McDonald, F. E. Synthesis of the ABC Substructure of Brevenal by Sequential exo-Mode Oxacyclizations of Acyclic Polyene Precursors. *Org. Lett.,* **2017**, *19*, 22, 6036–6039.

[27]. Adamo, M. F. A.; Pergoli, R. Studies on the Generation of Unnatural C-Nucleosides with 1-Alkynyl-2-deoxy-d-riboses. *Org. Lett.,* **2007**, *9*, 22, 4443–4446.

[28]. Wang, J. L.; Li, H. J.; Wang, H. S.; Wu, Y. C. Regioselective 1,2-Diol Rearrangement by Controlling the Loading of $BF_3{\cdot}Et_2O$ and Its Application to the Synthesis of Related Nor-Sesquiterene- and Sesquiterene-Type Marine Natural Products. *Org. Lett.,* **2017**, *19*, 14, 3811–3814.

[29]. Lattanzi, A.; Senatore, A.; Massa, A.; Scettri, A. Novel Highly Regioselective $VO(acac)_2$/TBHP Mediated Oxidation of o-Alkenyl Phenols to o-Hydroxybenzyl Ketones. *J. Org. Chem.* **2003**, *68*, 9, 3691–3694.

[30]. Nicholson, W. I.; Barreteau, F.; Leitch, J. A.; Payne, R.; Priestley, I.; Godineau, E.; Battilocchio, C.; Browne, D. L. Direct Amidation of Esters by Ball Milling. *Angew. Chem. Int. Ed.,* **2021**, *60*, 21868–21874.

[31]. Ledoussal, B.; Gorgues, A.; Coq, A. L. Reduction Par Les Sels Chromeux De Bromures Benzyliques Ortho-O-Acyles Avec Transposition Du Groupement Acyle: De L' Ester : Acces Aux Ortho-Hydroxybenzyl Cetones Non Masquees Et Une Nouvelle Preparation De Benzo¦B¦Furannes Substitues En Position-2. *Tetrahedron.,* **1987**, *43*, 5841-5852.

[32]. Son, E. C.; Kim, S. Y.; Kim, S. G. Squaramide-Catalysed Asymmetric Intramolecular Oxa-Michael Reaction of α,β-Unsaturated Carbonyls Containing Benzyl Alcohol: Construction of Chiral 1-Substituted Phthalans. *J. Org. Chem.* **2021**, *86*, 9, 6826-6839.

[33] Miyata, K.; Kutsuna, H.; Kawakami, S.; Kitamura, M. A Chiral Bidentate sp2-N Ligand, Naph-diPIM: Application to CpRu-Catalysed Asymmetric Dehydrative C-, N-, and O-Allylation. *Angew. Chem. Int. Ed.* **2011**, *50*, 4649–4653.

[34]. Chen, D.; Berhane, I. A.; Chemler, S. R. Copper-Catalysed Enantioselective Hydroalkoxylation of Alkenols for the Synthesis of Cyclic Ethers. *Org. Lett.* **2020**, *22*, 19, 7409–7414.

[35]. Verma, A.; Jana, S.; Prasad, C. D.; Yadava, A.; Kumar, S. Organoselenium and DMAP co-catalysis: regioselective synthesis of medium-sized halolactones and bromooxepanes from unactivated alkenes. *Chem. Commun.,* **2016**, *52*, 4179-4182.

[36]. Zhang, C.; Ji, J.; Sun, P. Palladium-Catalysed Alkenylation via sp2 C–H Bond Activation Using Phenolic Hydroxyl as the Directing Group. *J. Org. Chem.* **2014**, *79*, 7, 3200–3205.

[37]. Lee, S.; Kaib, P. S. J.; List, B. Asymmetric Catalysis via Cyclic, Aliphatic Oxocarbenium Ions. *J. Am. Chem. Soc.* **2017**, *139*, 6, 2156-2159.

[38]. Lee, S.; Bae, H. Y.; List, B. Can a Ketone Be More Reactive than an Aldehyde? Catalytic Asymmetric Synthesis of Substituted Tetrahydrofurans. *Angew. Chem. Int. Ed.* **2018**, *57*, 12162–12166.

[39]. Trend, R. M.; Ramtohul, Y. K.; Ferreira, E. M.; Stoltz, B. M. Palladium-Catalysed Oxidative Wacker Cyclizations in Nonpolar Organic Solvents with Molecular Oxygen: A Stepping Stone to Asymmetric Aerobic Cyclizations. *Angew. Chem. Int. Ed.* **2003**, *42*, 2892-2895.

[40]. Asano, K.; Matsubara, S. Asymmetric Catalytic Cycloetherification Mediated by Bifunctional Organocatalysts. *J. Am. Chem. Soc.,* **2011**, *133*, 42, 16711-16713.

[41]. Kwon, H. Y.; Park, C. M.; Lee, S. B.; Youn, J. H.; Kang, S. H. Asymmetric Iodocyclization Catalysed by Salen–CrIIICl: Its Synthetic Application to Swainsonine. *Chem. Eng. J.,* **2008**, *14*, 1023-1028.

[42]. Barbe, G.; Charette, A. B. Highly Chemoselective Metal-Free Reduction of Tertiary Amides. *J. Am. Chem. Soc.* **2008**, *130*, 1, 18–19.

[43]. Ransborg, L. K.; Albrecht, Ł.; Weise, C. F.; Bak, J. R.; Jørgensen, K. A. Optically Active Thiophenes *via* an Organocatalytic One-Pot Methodology. *Org. Lett.,* **2012**, *14*, 3, 724-727.

[44]. Szostak, M.; Spain, M.; Eberhart, A. J.; Procter, D. J. Highly Chemoselective Reduction of Amides (Primary, Secondary, Tertiary) to Alcohols using SmI_2/Amine/H_2O under Mild Conditions. *J. Am. Chem. Soc.* **2014**, *136*, 6, 2268-2271.

[45]. Tietze, L. F.; Jackenkroll, S.; Hierold, J.; Ma, L.; Waldecker, B. A Domino Approach to the Enantioselective Total Syntheses of Blennolide C and Gonytolide C. *Chem. Eng. J.,* **2014**, *20*, 8628-8635.

[46] (a) te Velde, G.; Bickelhaupt, F. M.; Baerends, E. J.; Fonseca Guerra, C.; van Gisbergen, S. J. A.; Snijders, J. G.; Ziegler, T. Chemistry with ADF. *J. Comput. Chem.* **2001**, *22*, 931–967. (b) Fonseca Guerra, C.; Snijders, J. G.; te Velde, G.; Baerends, E. J. Towards an Order-N DFT Method. *Theor. Chem. Acc.* **1998**, *99*, 391–403. ADF2018.105, SCM Theoretical Chemistry, Vrije Universiteit: Amsterdam (The Netherlands), 2017. http://www.scm.com.

[47] (a) Slater, J. C. Quantum Theory of Molecules and Solids. (McGraw-Hill, New York, 1974). (b) Becke, A. D. Density Functional Calculations of Molecular Bond Energies. *J. Chem. Phys.* **1986**, *84*, 4524–4529. (c) Becke, A. D. Density-Functional Exchange-Energy Approximation with Correct Asymptotic Behavior. *Phys. Rev. A* **1988**, *38*, 3098–3100.

[48] Lee, C.; Yang, W.; Parr, R. G. Development of the Colle-Salvetti Correlation-Energy Formula into a Functional of the Electron Density. *Phys. Rev. B* **1988**, 37, 785–789.

[49] van Lenthe, E.; Baerends, E. J. Optimized Slater-Type Basis Sets for the Elements 1-118. *J. Comput. Chem.* **2003**, *24,* 1142–1156.

[50] (a) Klamt, A.; Schüürmann, G. COSMO: A New Approach to Dielectric Screening in Solvents with Explicit Expressions for the Screening Energy and its Gradient. *J. Chem. Soc. Perkin Trans. 2* **1993**, 799–805. (b) Klamt, A. Conductor-like Screening Model for Real Solvents: A New Approach to the Quantitative Calculation of Solvation Phenomena. *J. Phys. Chem.* **1995**, *99*, 2224–2235. (c) Klamt, A.; Jonas, V. Treatment of the Outlying Charge in Ccontinuum Solvation Models. *J. Chem. Phys.* **1996**, *105*, 9972–9981. (d) Pye, C. C.; Ziegler, T. An Implementation of the Conductor-like Screening Model of Solvation within the Amsterdam Density Functional Package. *Theor. Chem. Acc.* **1999**, *101*, 396–408.

[51] (a) Grimme, S.; Antony, J.; Ehrlich, S.; Krieg, H. A Consistent and Accurate *ab initio* Parametrization of Density Functional Dispersion Correction (DFT-D) for the 94 Elements H-Pu. *J. Chem. Phys.* **2010**, *132*, 154104. (b) Becke, A. D.; Johnson, E. R. A Density-Functional Model of the Dispersion Interaction. *J. Chem. Phys.* **2005**, *123*, 154101.

[52] (a) van Lenthe, E.; Baerends, E. J.; Snijders, J. G. Relativistic Regular Two-Component Hamiltonians. *J. Chem. Phys.* **1993**, *99*, 4597–4610. (b) van Lenthe, E.; Baerends, E. J.; Snijders, J. G. Relativistic Total Energy using Regular Approximations. *J. Chem. Phys.* **1994**, *101*, 9783–9792.

[53] CYLview20; Legault, C. Y., Unversité de Sherbrooke: Sherbrooke, 2020 (http://www.cylview.org).

[54] Zhao, Y.; Truhlar, D. G. The M06 suite of density functionals for main group thermochemistry, thermochemical kinetics, noncovalent interactions, excited states, and transition elements: two new functionals and systematic testing of four M06-class functionals and 12 other functionals. *Theor. Chem. Acc.* **2008**, *120*, 215–241.

[55] (a) Vermeeren, P.; van der Lubbe, S. C. C.; Fonseca Guerra, C.; Bickelhaupt, F. M.; Hamlin, T. A. Under-standing chemical reactivity using the activation strain model. *Nature Protoc,* **2020**, *15*, 649–667. (b) Bickelhaupt, F. M.; Houk, K. N. Analyzing Reaction Rates with the Distortion/Interaction-Activation Strain Model. *Angew. Chem. Int. Ed.* **2017**, *56*, 10070–10086. (c) Wolters, L. P.; Bickelhaupt, F. M. The activation strain model and molecular orbital theory. *WIRES Comput. Mol. Sci.* **2015**, *5*, 324–343. (d) Fernández, I.; Bickelhaupt, F. M. The activation strain model and molecular orbital theory: understanding and design-ing chemical reactions. *Chem. Soc. Rev.* **2014**, *43*, 4953–4967. (e) van Zeist, W.-J.; Bickelhaupt, F. M. The activation strain model of chemical reactivity. *Org. Biomol. Chem.* **2010**, *8*, 3118–3127. (f) Vermeeren, P.; Hamlin, T. A.; Bickelhaupt, F. M. *Chem. Comm.* **2021**, *57*, 5880–5896.

[56] Bickelhaupt, F. M.; Baerends, E. J. in Reviews in Computational Chemistry (Eds.: K. B. Lipkowitz, D. B. Boyd), Wiley, Hoboken, 2000, pp. 1–86.

[57] (a) Okino, T.; Hoashi, Y.; Takemoto, Y. Enantioselective Michael Reaction of Malonates to Nitroolefins Catalyzed by Bifunctional Organocatalysts. *J. Am. Chem. Soc.* **2003**, *125*, 12672–12673. (b) Okino, T.; Hoashi, Y.; Furukawa, T.; Xu, X.; Takemoto, Y. Enantio- and Diastereoselective Michael Reaction of 1,3-Dicarbonyl Compounds to Nitroolefins Catalyzed by a Bifunctional Thiourea. *J. Am. Chem. Soc.* **2005**, *127*, 119–125. (c) Hamza, A.; Schubert, G.; Soós, T.; Pápai, I.

Theoretical Studies on the Bifunctionality of Chiral Thiourea-Based Organocatalysts: Competing Routes to C–C Bond Formation. *J. Am. Chem. Soc.* **2006**, *128*, 13151–13160. (d) Kótai, B.; Kardos, G.; Hamza, A.; Farkas, V.; Pápai, I.; Soós, T. On the Mechanism of Bifunctional Squaramide Catalyzed Organocatalytic Michael Addition: A Protonated Catalyst as an Oxyanion Hole. *Chem. Eur. J.* **2014**, *20*, 5631–5639. (e) Su, G.; Thompson, C. J.; Yamazaki, K.; Rozsar, D.; Christensen, K.; Hamlin, T. A.; Dixon, D. J. A Bifunctional Iminophosphorane Squaramide Catalyzed Enantioselective Synthesis of Hydroquinazolines via Intramolecular Aza-Michael Reaction to α,β-Unsaturated Esters, *Chem. Sci.* **2021**, *12*, 6064–6072.
